# Enantioselective synthesis and characterization of [*m*.*n*]paracyclophanes

**DOI:** 10.1039/d6sc05207j

**Published:** 2026-08-05

**Authors:** Yilu Wen, Yaqi Yao, Yujun Zhong, Chenyang Li, Changgui Zhao

**Affiliations:** a Key Laboratory of Radiopharmaceuticals, Ministry of Education, College of Chemistry, Beijing Normal University Beijing 100875 China cgzhao@bnu.edu.cn; b Key Laboratory of Theoretical and Computational Photochemistry, Ministry of Education, College of Chemistry, Beijing Normal University Beijing 100875 China chenyang.li@bnu.edu.cn

## Abstract

[*m*.*n*]Paracyclophanes ([*m*.*n*]PCPs) are widely used in asymmetric catalysis, energy materials, and π-stacked polymers. To date, however, most studies have focused on [2.2]PCPs. The atroposelective synthesis and structural characterization of [*m*.*n*]PCPs with variable ansa chain lengths remain underexplored. In this study, we report an N-heterocyclic carbene-catalyzed enantioselective macrocyclization. This method enables the efficient construction of a diverse array of [*m*.*n*]PCPs with excellent enantioselectivities. A comprehensive investigation was conducted, including ring strain energy calculations, aromatic interaction energy analysis, racemization barrier measurements, local strain visualization, and systematic geometric analysis. These studies provide detailed insights into the relationship between the macrocyclic structure and properties. The synthetic utility of [*m*.*n*]PCPs is demonstrated by a [6.5]PCP-derived thiourea organocatalyst that effectively promotes the asymmetric amination reaction.

## Abstract 2

An NHC-catalyzed enantioselective macrocyclization is developed to access diverse [*m*.*n*]PCPs in up to 97% ee. Subsequent structural interrogation of strain energy, aromatic stacking, and local strain establishes clear structure–property correlations.

## Graphical abstract

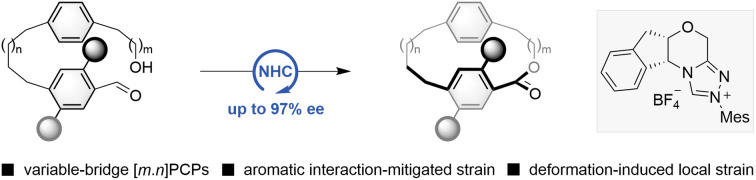

## Introduction

**Scheme 1:**
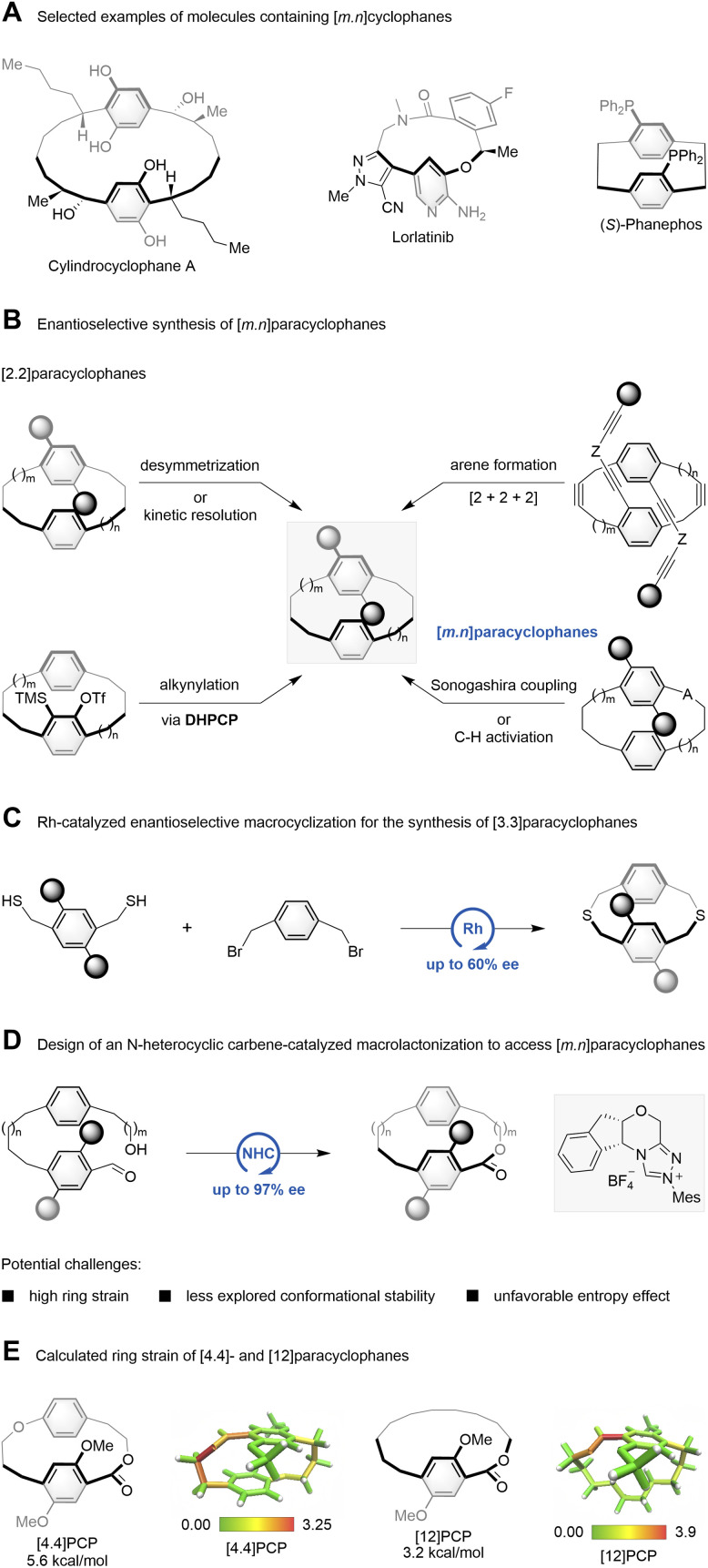
Overview of the [*m*.*n*]cyclophanes and design of an N-heterocyclic carbene (NHC)-catalyzed macrolactonization to access [*m*.*n*]paracyclophanes. DHPCP = dehydro-[2.2]-paracyclophane.

[*m*.*n*]Cyclophanes form the cores of many bioactive natural products, such as cylindrocyclophane A, which contains a [7.7]paracyclophane and exhibits cytotoxic, antifungal, and antibacterial activities (Scheme 1A).^1,2^ In addition, these strained macrocycles are known to be prevalent scaffolds in drug discovery. For example, lorlatinib, which contains a [5.2]metaorthocyclophane, has been approved by the United States Food and Drug Administration (US FDA) for the treatment of anaplastic lymphoma kinase-positive non-small cell lung cancer.^3^ Notably, [2.2]paracyclophanes are attractive molecules due to the intriguing structural and electronic properties,^4^ which have been widely used in asymmetric catalysis,^5–7^ energy materials,^8^ π-stacked polymers,^9,10^ and functional parylene coatings.^11,12^

Recently, catalytic enantioselective synthesis of planar-chiral [*m*.*n*]paracyclophanes ([*m*.*n*]PCPs) has attracted increasing attention (Scheme 1B).^13^ In this context, most efforts have been confined to late-stage asymmetric modification of [2.2]PCPs with strategies including transition metal- or organo-catalyzed desymmetrization and kinetic resolution.^14–22^ Notably, Tanaka and co-workers developed Rh- or Ni-catalyzed intramolecular double [2 + 2 + 2] cycloadditions for the enantio- and diastereoselective construction of [2.2]triphenylenophanes bearing two planar-chiral elements.^23^ More recently, Yu, Xu and co-workers described an enantioconvergent synthesis of [2.2]PCPs *via in situ* generation of dehydro-[2.2]paracyclophane followed by nucleophilic addition or cycloaddition.^24^ Despite these advances, the atroposelective synthesis and structural characterization of [*m*.*n*]PCPs with varying ansa chain lengths remain underexplored.^25–30^ In 2009, Shibata reported a Pd-catalyzed Sonogashira cross-coupling for the preparation of [6.6]- and [7.7]PCPs.^31^ In 2023, Zhao and co-workers achieved Pd/pGlu-catalyzed late-stage C–H olefination of cyclophanes, affording [6.6]PCPs with moderate enantioselectivity.^32^

Asymmetric macrocyclization of an appropriate linear precursor represents a promising strategy for the synthesis of [*m*.*n*]PCPs, as it enables the simultaneous formation of the macrocyclic framework and planar chirality in a single step. To date, only one enantioselective macrocyclization for the synthesis of [3.3]PCPs has been developed (Scheme 1C).^33^ This route involved the Rh-catalyzed S_N_2 nucleophilic addition and was developed by Tanaka. In contrast, asymmetric macrolactonization has been well established for the construction of planar-chiral [*n*]cyclophanes. Pioneering work by Collins demonstrated an enzymatic atroposelective macrolactonization,^34^ followed by organocatalytic macrocyclizations reported by Zhao,^35^ Wang,^36^ and Chi/Wu,^37^ affording cyclophanes, indolophanes, and naphthalenophanes, respectively. Subsequently, our group further developed dynamic kinetic resolution/oxidative esterification^38^ and desymmetrization macrocyclization^39^ for the enantioselective synthesis of cyclophanes. Building on these advances, we envisioned that N-heterocyclic carbene-catalyzed macrocyclization of aryl-tethered precursors could offer a viable route to [*m*.*n*]PCPs (Scheme 1D). However, several challenges must be addressed: (1) the inherently high ring strain associated with [*m*.*n*]PCPs,^29,40,41^ (2) the limited understanding of the relationship between the structure and conformational stability,^2,42^ and (3) the unfavorable entropic effects and often unpredictable efficiency of macrocyclization reactions.^43,44^ Notably, incorporating a rigid benzene ring into the ansa chain can substantially alter the macrocyclic strain compared to purely aliphatic-bridged analogs, a structural effect that remains poorly understood.^29^ For instance, computational analysis of [4.4]PCP revealed a 2.4 kcal mol^−1^ increase in ring strain relative to the corresponding [12]PCP (Scheme 1E). Herein, we report the enantioselective synthesis and systematic characterization of [*m*.*n*]PCPs.

## Results and discussion

### Reaction optimization and substrate scope

The NHC-catalyzed macrolactonization was investigated using readily accessible linear precursor 1 as the model substrate (Tables 1, S1 and S2). Initial screening of carbene precatalysts was conducted under standard conditions: K_2_CO_3_ as the base, 3,3′,5,5′-tetra-*tert*-butyldiphenoquinone (DQ) as the oxidant, 4 Å molecular sieves as the additive, and THF as the solvent. Indanol-derived NHC precatalysts bearing electron-deficient *N*-substituents generally provided slightly diminished enantioselectivity (entries 1 and 2). In contrast, precatalysts with *N*-Mes, *N*-2,4,6-(^*i*^Pr)_3_C_6_H_2_, *N*-Ph, and *N*-2,6-(Et)_2_C_6_H_3_ substituents delivered [6.5]PCP 2 with improved enantioselectivity (entries 3–6). Pyrrolotriazolium- and amino-alcohol-derived precatalysts resulted in lower reaction efficiencies (entries 7–9). Notably, the relatively low yield was partly attributed to the formation of a dimeric byproduct 3. Replacement of K_2_CO_3_ with organic bases such as DMAP or DBU led to diminished yields (entries 10 and 11). Solvent screening revealed that toluene and hexanes afforded both lower yields and enantioselectivities (entries 12 and 13). Increasing the reaction concentration to 20 mM reduced the yield, while dropwise addition of 1 did not improve the outcome (entries 14 and 15). Interestingly, increasing the reaction temperature led to higher yields, likely due to increased conformational flexibility of the ansa chain facilitating the macrocyclization (entries 16 and 17). Accordingly, the conditions outlined in entry 17 were identified as optimal and employed for subsequent exploration of the substrate scope.

**Table 1:** Optimization of the reaction conditions

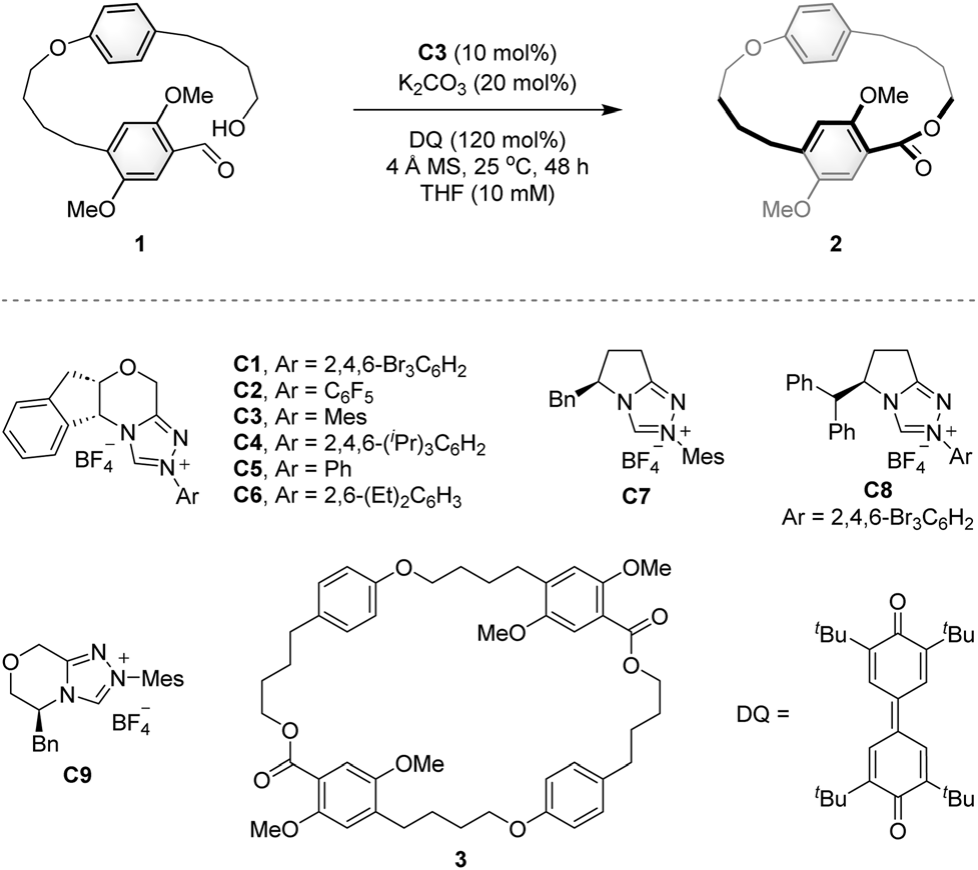			
Entry^a^	Variation from above conditions	Yield^b^ (%)	ee^c^ (%)
1	C1	39	82
2	C2	26	86
3	None	36	95
4	C4	28	96
5	C5	24	93
6	C6	19	96
7	C7	15	79^d^
8	C8	24	88^d^
9	C9	17	61^d^
10	DMAP	18	96
11	DBU	17	93
12	Toluene	26	78
13	Hexanes	15	47
14	THF (5.0 mL)	18	95
15	1 was added dropwise	36	96
16	THF (30.0 mL)	40	96
17^e^	THF (30.0 mL)	61	94

a Reaction conditions: a mixture of 1 (0.10 mmol), DQ (0.12 mmol), base (20 mol%), 4 Å MS (50 mg), and NHC (10 mol%) in THF (10.0 mL) was stirred at 25 °C for 48 h.

b Isolated yield of 2.

c The enantiomeric excess (ee) of 2 was determined by HPLC using a chiral stationary phase.

d The product was obtained with the opposite configuration.

e The reaction was conducted at 70 °C. DMAP = 4-dimethylaminopyridine; DBU = 1,8-diazabicyclo[5.4.0]undec-7-ene.

**Scheme 2:**
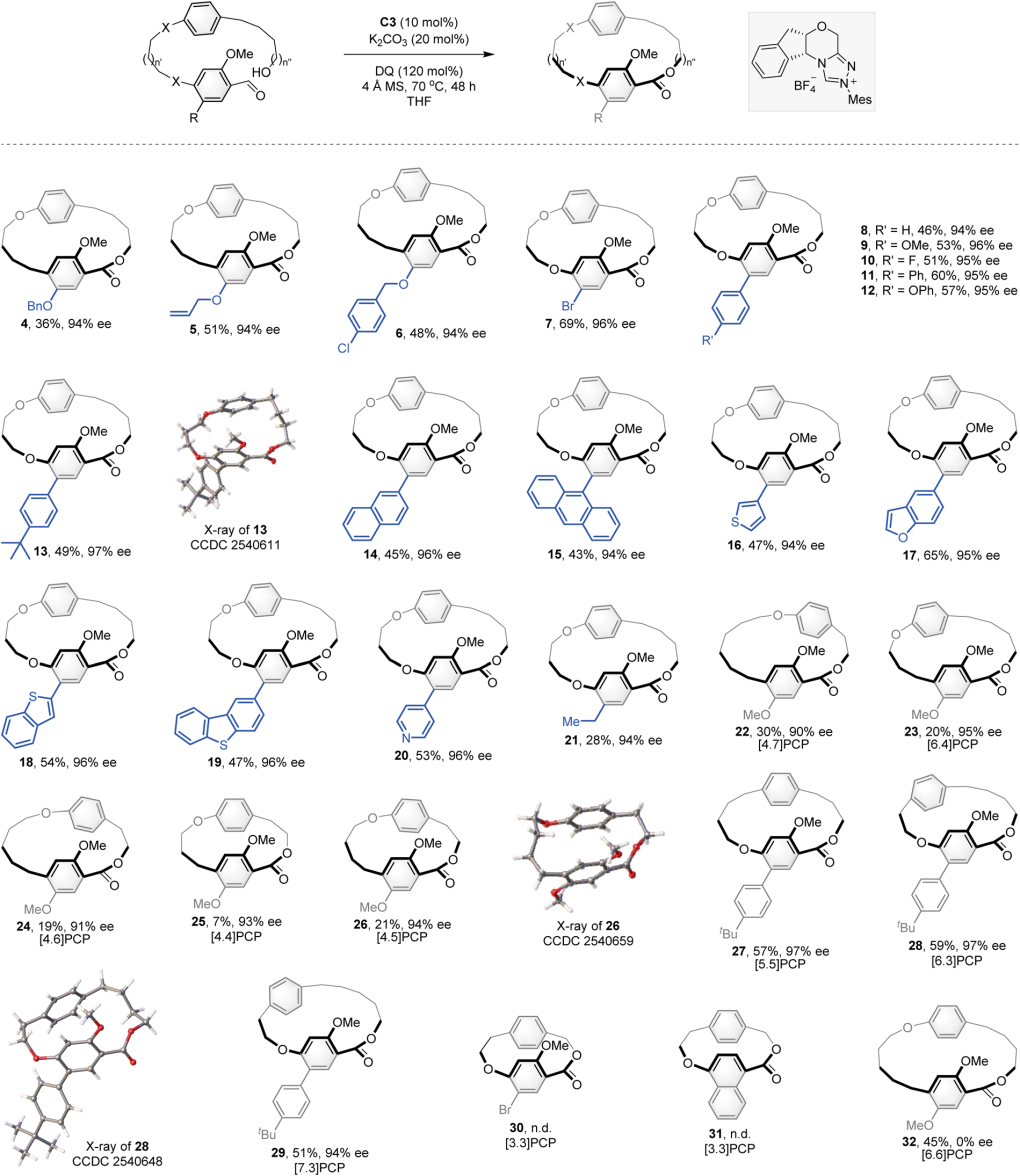
Scope of the substrate. n.d. = not detected.

With the optimized reaction conditions established, the scope of benzene ring substituents was explored (Scheme 2). Substrates bearing 2,5-dialkoxy groups on the benzene ring were well tolerated, affording the desired products 4–6 in moderate yields with excellent enantioselectivities. The bromo-substituted substrate was successfully converted to macrocycle 7 in 69% yield and 96% ee. A scale-up experiment on a 1.0 mmol scale delivered 7 in 23% yield while maintaining the enantioselectivity. Subsequently, the compatibility of aryl-substituted substrates was examined. A range of substituents including phenyl, 4-*tert*-butylphenyl, 4-methoxyphenyl, 4-fluorophenyl, 4-biphenyl, and 4-phenoxyphenyl proved to be viable, providing the planar-chiral macrolides 8–13 in excellent enantioselectivities. Substrates incorporating 2-naphthyl, 9-anthracenyl, and heteroaromatic rings were also compatible under the reaction conditions, delivering the corresponding products with outstanding stereocontrol. In addition, the ethyl-substituted substrate was well tolerated, yielding the [6.5]PCP 21 with 94% ee. Finally, the absolute configuration of 9 was confirmed by single-crystal X-ray crystallography, and the configurations of other related structures were assigned by analogy. Next, the scope of the ansa chain was evaluated for the synthesis of [*m*.*n*]PCPs. Substrates bearing different linkers were well tolerated, delivering a series of PCPs (22–29) with consistently high enantioselectivities. Notably, [4.4]PCP 25 was obtained in a low yield, which is largely due to the substantial conformational strain associated with macrocyclization and competing oligomerization that reduce the overall efficiency. Further attempts to access the corresponding [3.3]PCPs 30–31 were unsuccessful, presumably as a result of the dramatically enhanced ring strain.^45^ The [6.6]PCP product 32 obtained was racemic, indicating low conformational stability under the reaction conditions.

### Ring strain energy and configurational stability

**Scheme 3:**
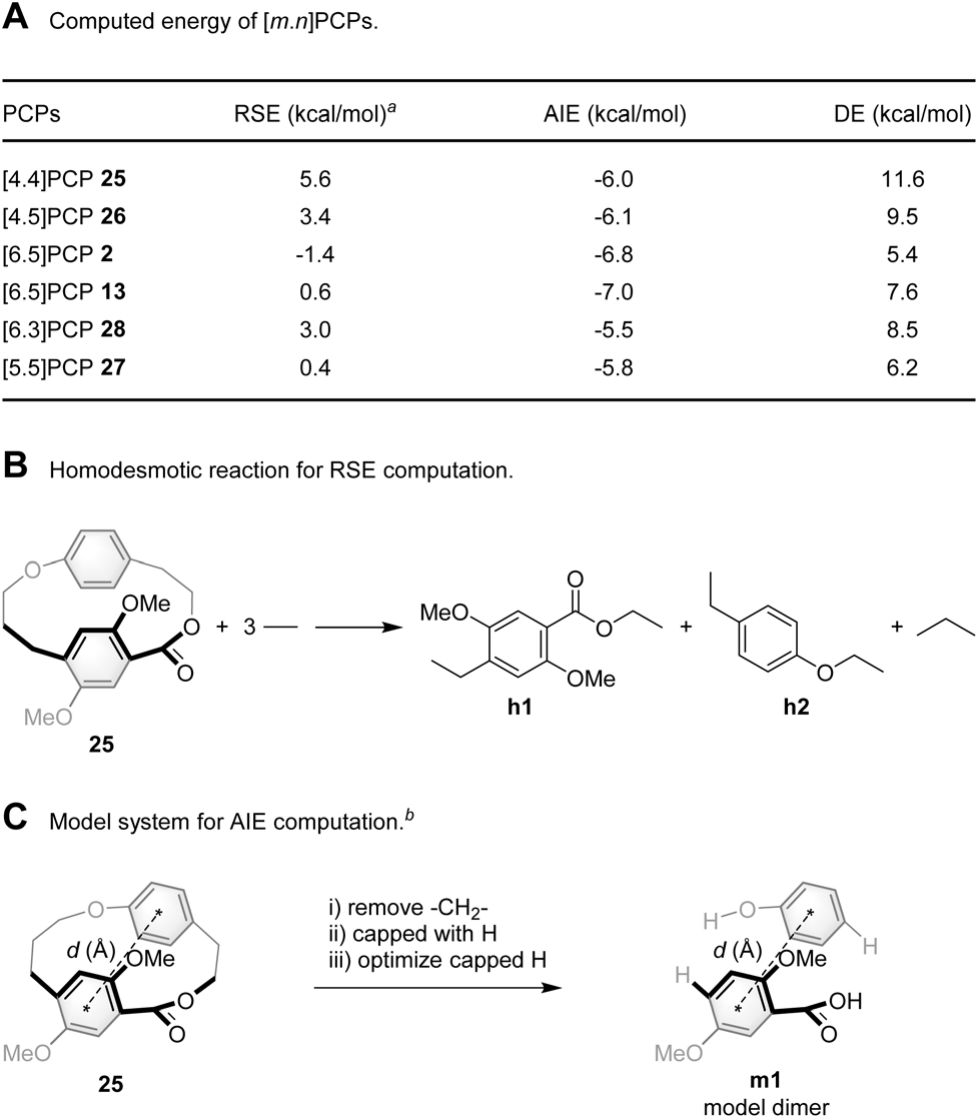
Ring strain, aromatic interaction, and deformation energy analysis. ^*a*^The RSE was calculated as the negative enthalpic change of the homodesmotic reaction: RSE_298K_ = *H*_298K_(reactants) − *H*_298K_(products). The enthalpy at 298.15 K was evaluated as *H*_298K_ = *E*_elec_(B2PLYP-D3) + *E*_ZPVE_(M06-2X) + Δ*H*_298K_(M06-2X), where the terms on the right hand side correspond to the electronic single-point energy, the zero-point vibrational energy (ZPVE) and the thermal correction, respectively. ^*b*^The AIE is the energy difference between the optimized structure and a non-interacting reference. Taking 25 as an example, AIE = *E*_model_(*d*) − *E*_model_(20 Å) = *E*_model_(3.49 Å) − *E*_model_(20 Å), where *d* represents the distance between the two aromatic rings in the PCP.

Ring strain energy (RSE) provides an overall thermodynamic measure of macrocyclization relative to a hypothetical strain-free reference structure.^29,46,47^ Accordingly, we employed homodesmotic reactions to evaluate the RSEs of several representative [*m*.*n*]PCPs with varying bridge lengths.^48^ An example for [4.4]PCP 25 is shown in Scheme 3B, and the complete dataset is provided in Table S11. In general, the RSE decreases with increasing ring size. [4.4]PCP 25 exhibits an RSE of 5.6 kcal mol^−1^, which is 2.4 kcal mol^−1^ higher than that of [12]PCP, a paracyclophane analog lacking a benzene ring in the ansa chain. The RSEs of [6.5]PCP 13 and [5.5]PCP 27 are close to zero, while [6.5]PCP 2 shows an unexpected negative value. These observations suggest that RSE not only reflects structural deformation of the C/O-based macrocyclic backbone but also includes stabilizing contributions that likely arise from noncovalent interactions between the two benzene rings.^49^

To separate these two contributions, a model system was constructed by removing the linker between the two benzene rings and capping the resulting dangling bonds with hydrogen atoms (Scheme 3C).^50,51^ The aromatic interaction energy (AIE) is negative for all [*m*.*n*]PCP-derived model dimers, confirming that the through-space aromatic interaction stabilizes the macrocyclic frameworks.^52^ We therefore define the deformation energy (DE) of the macrocyclic backbone as RSE minus AIE.^53^ The resulting DE values show a clearer dependence on ring size. For the R = OMe series, DE follows the order [4.4]PCP 25 (11.6 kcal mol^−1^) > [4.5]PCP 26 (9.5 kcal mol^−1^) > [5.5]PCP (5.7 kcal mol^−1^) ≈ [6.5]PCP 2 (5.4 kcal mol^−1^). Importantly, all DEs are positive, confirming that the low or negative RSE values of larger macrocycles arise in part from compensating aromatic stabilization rather than from an absence of skeletal deformation. Substituent and linking-atom effects further modulate the DE. Introducing the *tert*-butylphenyl group into [5.5]PCP increases the DE only modestly, from 5.7 to 6.2 kcal mol^−1^. In [6.5]PCP, the corresponding increase is larger, reflecting the additional local deformation associated with the replacement of CH_2_ with O at the C4 bridgehead (see StrainViz analysis below). Overall, shortening the ansa chain is the primary factor governing skeletal deformation, whereas bulky substituents play only a minor role.

**Scheme 4:**
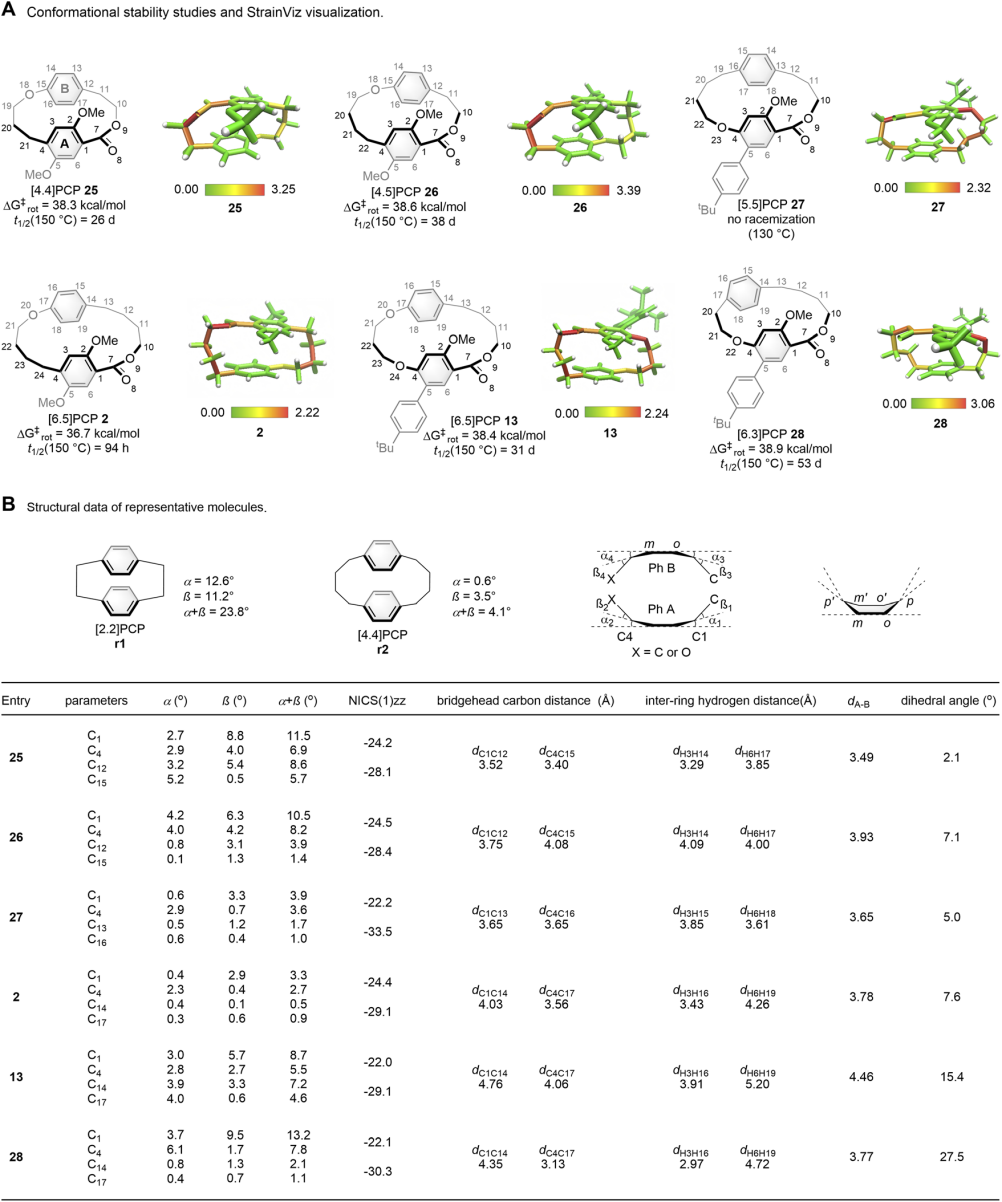
Conformational stability studies, StrainViz visualization, and structural data of representative molecules. The structures of 25, 2, and 27 were obtained from theoretical calculations, while those of 26, 13, and 28 were determined by single-crystal X-ray diffraction.

Although DE quantifies the thermodynamic cost of geometric distortion, it does not directly capture the kinetic configurational stability.^54^ To address this, we experimentally measured the racemization energy barriers for representative [*m*.*n*]PCPs (Scheme 4A).^55^ All examined compounds exhibit high configurational stability. The barrier for [6.5]PCP 2 (36.7 kcal mol^−1^) is the lowest, whereas [6.3]PCP 28 and [4.5]PCP 26 possess higher barriers of 38.9 and 38.6 kcal mol^−1^, respectively. Notably, these barrier heights correlate positively with the computed DE values, that is, greater skeletal deformation accompanies enhanced configurational persistence.^56^

### Local strain and geometric analyses

To determine how deformation is accommodated within the macrocyclic skeleton, we visualize the local strain distribution using StrainViz.^57,58^ As shown in Scheme 4A, the strain is localized along the side chains attached to the aromatic ring. For 25 and 26, the most pronounced strain is concentrated at the ester C(sp^3^)–O bond, while 28 exhibits notable strain at the C(sp^2^)–O–C(sp^3^) bridge linked to the C4 bridgehead of the 2,5-disubstituted benzene ring A. As the ring size increases to 27, 13, and 2, the local strain becomes weaker and more delocalized, indicating that larger macrocycles can more effectively relieve skeleton deformation. The comparison between 2 and 13 further suggests that the oxygen linkage at the C4 bridgehead contributes additional localized strain.

The observed strain can be rationalized by comparing the geometries of the macrocycle and the corresponding open-chain species used in the StrainViz protocol.^57^ In most structures, torsional deformation is the dominant strain-relief mode. For example, in 25 and 26, the largest strain occurs around the O9–C10 and C10–C11 linkages, where dihedral contributions account for 58–78% of the local strain energy. The corresponding C1–C7–O9–C10 and O9–C10–C11–C12 dihedral angles relax substantially upon bond cleavage, differing by ∼10° and 22°, respectively. In the larger macrocycles 27, 13, and 2, strain is distributed over a broader segment of the ansa chain rather than concentrated at a single bond. Compound 28 represents a distinct case, where bond-angle bending also makes a notable contribution to the local strain of the C21–O22 and C4–O22 bonds. Overall, ring closure imposes both torsional and angular strain on the ansa chain, and their relative contributions are governed by ring size and bridge asymmetry.

We next examined the bending angles *α* and *β* of the benzene rings (Scheme 4B). The quantity *α* + *β* is commonly used for small PCPs to measure the out-of-plane deformation of the phenyl rings.^29^ Ring A is generally more distorted than ring B, consistent with stronger geometric constraints near the ester-linked side. The compact [4.4]PCP 25 exhibits notable distortions in both rings (*α* + *β* = 5.7–11.5°), likely due to its short symmetric bridge and close inter-ring contact (3.49 Å). In contrast, the larger [5.5]PCP 27 shows much smaller distortions (1.0–3.9°), indicating that symmetry alone does not determine ring bending. Comparisons among 25, 26, and 28 suggest that bridge asymmetry promotes uneven distortion between the two rings, most strikingly in [6.3]PCP 28, which displays the largest inter-ring difference and a maximum *α* + *β* of 13.2° at C1. Despite identical bridge lengths, 2 and 13 exhibit different distortion patterns, highlighting the roles of substituents and linking atoms in modulating local deformation. Notably, the *α* + *β* values of these oxa-bridged cyclophanes are at least 10.6° smaller than that of all-carbon [2.2]PCP r1, while ring A in [5.5]PCP 27 shows a benzene distortion comparable to that of [4.4]PCP r2. Overall, smaller ring size and greater bridge asymmetry favour stronger and more uneven benzene-ring bending.

### Aromaticity and inter-ring interaction patterns

**Scheme 5:**
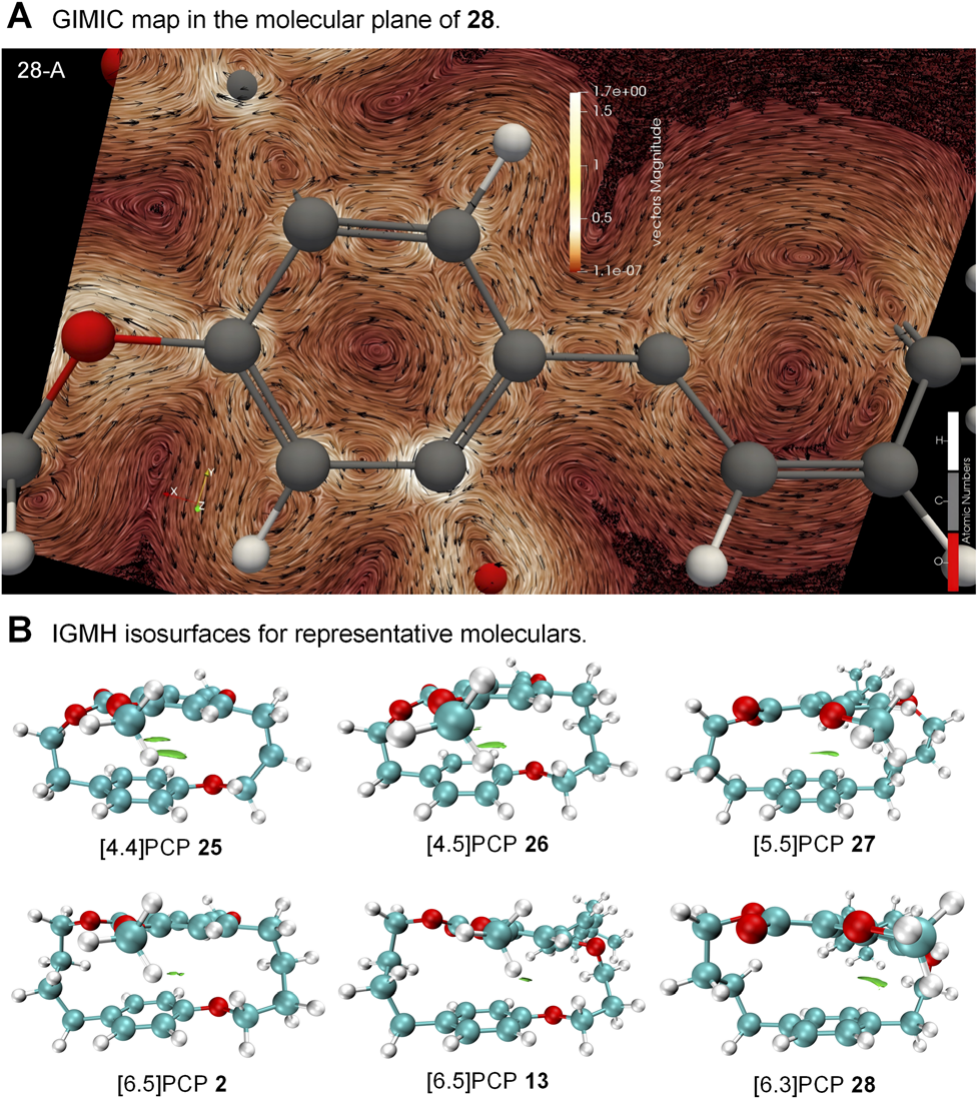
Visualization of aromaticity and non-covalent interactions. ^*a*^The arrows indicate the direction of the induced current, and the color corresponds to the magnitude of the induced current. The applied magnetic field is perpendicular to the plane of the benzene ring. ^*b*^IGMH colors range from blue (strong attractive) to green (weak van der Waals).

Scheme 4B also reports the out-of-plane magnetic shielding component evaluated 1 Å above the ring center [NICS(1)zz].^59^ The markedly negative values indicate that both benzene rings retain substantial aromatic character despite their bending deformation. This conclusion is further supported by the gauge—including magnetically induced current (GIMIC) analysis of 28—which shows a diatropic ring current in the more distorted ring A (Scheme 5A).^60,61^ Ring B generally exhibits more negative NICS(1)zz values than the substituted ring A, consistent with its smaller bending angles and suggesting slightly stronger aromaticity.

The above AIE analysis established that favorable intramolecular aromatic interactions contribute to the stabilization of these macrocycles. Herein, we examine the relative orientations of the two benzene rings using their dihedral angles (*τ*), bridgehead C⋯C distance differences (Δ*d*_C–C_), and inter-ring H⋯H distance differences (Δ*d*_H–H_). Symmetric [4.4]PCP 25 and [5.5]PCP 27 adopt nearly parallel ring arrangements (*τ* ≤ 5°), whereas the unsymmetrical analogues adopt increasingly tilted arrangements. This trend is most pronounced for [6.3]PCP 28, which exhibits both the largest dihedral angle (27.5°) and Δ*d*_C–C_ (1.22 Å). Notable lateral displacement is observed for 13 and 28, as reflected by their large Δ*d*_H–H_ values (≥1.29 Å). Overall, these geometric descriptors reveal two representative inter-ring motifs:^62–64^ a parallel-displaced geometry for 25, 26, 27 and 2, and a tilted T-shaped geometry for 13 and 28.

To visualize the noncovalent interactions associated with these stacking motifs, independent gradient model based on Hirshfeld partition (IGMH) analysis was performed on the corresponding macrocycles (Scheme 5B).^65^ The resulting isosurfaces are consistent with the geometric trends described above. The offset-stacked [4.4]PCP 25 displays an obvious attractive interaction region between the two benzene rings due to its nearly parallel arrangement and short ring separation. In contrast, the highly tilted [6.3]PCP 28 shows a more diffuse isosurface, consistent with the weaker noncovalent interaction in the T-shaped arrangement. These results show that the macrocycle-imposed ring orientation strongly influences the strength and nature of inter-ring interactions, providing a structural rationale for the observed variations in AIEs across the series.

### Spectroscopic signature and catalytic application

**Scheme 6:**
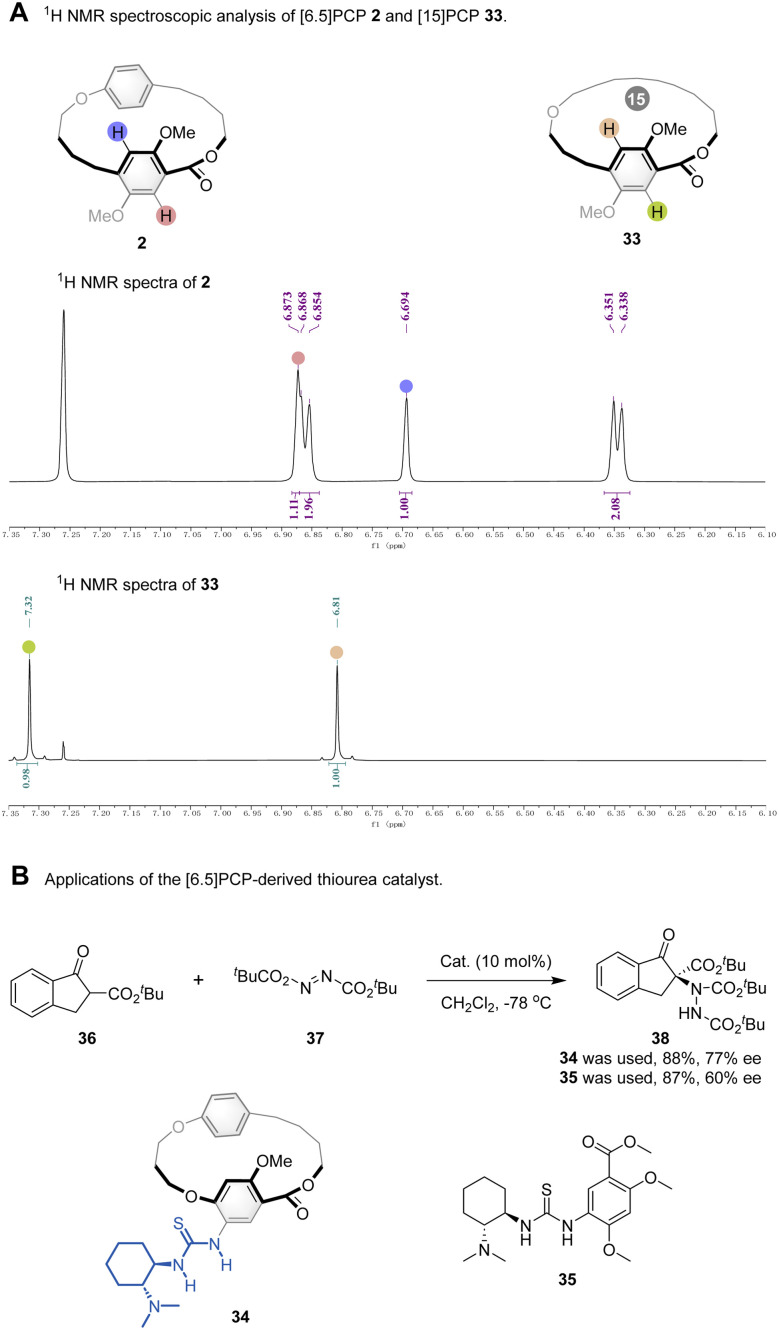
^1^H NMR spectroscopic analysis and applications of the [6.5]PCP-derived thiourea catalyst.

The aromatic interactions revealed in the spatial arrangement analysis are also observable in the experimental NMR data (Scheme 6A). ^1^H NMR spectroscopic analysis revealed upfield shifts of the aromatic proton signals relative to a reference macrocycle lacking the ansa-bridged benzene moiety.^35^ This diamagnetic shift arises from the shielding effect of the opposing aromatic ring current. The shift magnitude correlates with the average inter-ring distance and the degree of benzene ring deformation.^66^

To explore potential applications of this scaffold, we further functionalized the [*m*.*n*]PCP framework (Scheme 6B). Buchwald–Hartwig amination of compound 7 smoothly delivered the Boc-protected amine. Deprotection, followed by sequential treatment with thiophosgene (CSCl_2_) and (1*S*,2*S*)-*N*,*N*′-dimethylcyclohexane-1,2-diamine, afforded thiourea bifunctional catalyst 34. In the asymmetric amination reaction of β-ketoester 36 to di-*tert*-butyl azodicarboxylate (DBAD, 37), thiourea catalyst 34 proved more effective than its non-planar-chiral analogue 35, delivering product 38 in 88% yield and 77% ee. These results highlight the utility of the constructed atropisomeric scaffold in asymmetric catalysis.

## Conclusion

We have developed an NHC-catalyzed enantioselective macrocyclization that affords planar-chiral [*m*.*n*]paracyclophanes with variable ansa chain lengths and excellent enantioselectivities (up to 97% ee). Comprehensive computational and experimental investigations revealed that ring strain is mitigated by attractive aromatic interactions, with deformation energy increasing sharply as the ring size decreases. Racemization barrier measurements demonstrated high configurational stability (36.7–38.9 kcal mol^−1^), correlating positively with skeletal distortion. StrainViz visualization and geometric analysis identified the ester-adjacent C–O bond and aryl ether bridge as primary loci of localized strain, while NICS and GIMIC analyses confirmed retained aromaticity despite significant ring bending. The synthetic utility of this scaffold is validated by a [6.5]PCP-derived thiourea organocatalyst that effectively promoted the asymmetric amination reaction. This work provides fundamental insights into the interplay between macrocyclic topology, conformational stability, and aromatic interactions. Future directions include expanding the scope to heteroatom-bridged congeners, leveraging the unique spatial arrangement for enantioselective supramolecular recognition, and exploring the catalytic potential of these planar-chiral architectures in challenging asymmetric transformations.

## Author contributions

Y. W. conducted the main experiments and drafted the manuscript; Y. Z. assisted with the experiments; Y. Y. and C. L. performed the DFT computations; C. Z. and C. L. supervised the project and co-wrote the manuscript.

## Conflicts of interest

There are no conflicts to declare.

## Supplementary Material

SC-OLF-D6SC05207J-s001

SC-OLF-D6SC05207J-s002

## Data Availability

CCDC 2540611, 2540648 and 2540659 contain the supplementary crystallographic data for this paper.^67*a*–*c*^ The data supporting this article have been included as part of the supplementary information (SI). Supplementary information: details of the experimental procedure; ^1^H NMR, ^13^C NMR, and HPLC spectra; X-ray crystallographic data and computational data. See DOI: https://doi.org/10.1039/d6sc05207j.
