# Supplementary material for: Enantioselective synthesis and characterization of [m.n]paracyclophanes
Source: Chem Sci. 2026 Aug 5;17(38):18427–35. doi: 10.1039/d6sc05207j (PMC13470676; doi:10.1039/d6sc05207j)
Supplement: Supplementary file 1 [file SC-017-D6SC05207J-s001.pdf]

Supplementary Information (SI) for Chemical Science.
This journal is © The Royal Society of Chemistry 2026

Supporting Information

Enantioselective Synthesis and Characterization of [*m.n*]Paracyclophanes

Yilu Wen,[a,‡] Yaqi Yao,[b,‡] Yujun Zhong,[a] Chenyang Li[b,*] and Changgui Zhao[a,*]

[a]Key Laboratory of Radiopharmaceuticals, Ministry of Education, College of Chemistry, Beijing Normal University, Beijing 100875, China.
[b]Key Laboratory of Theoretical and Computational Photochemistry, Ministry of Education, College of Chemistry, Beijing Normal University, Beijing 100875, China.
[‡]These authors contributed equally.
*Corresponding Author(s): chenyang.li@bnu.edu.cn; cgzhao@bnu.edu.cn.

Table of Contents

1. General information S-2
2. The beforehand supplementary information of main text S-2
3. Methods and procedures for the synthesis of substrates S-3
4. Derivatization and conformational stability studies S-10
5. Characterization data S-19
6. References S-43
7. X-Ray report S-44
8. DFT simulations computational methods S-47
9. Copies of NMR spectra S-242
10. Copies of HPLC spectra S-275

1. General information

All reactions in non-aqueous media were conducted under a positive pressure of dry nitrogen in glassware that had been dried in oven prior to use unless noted otherwise. Anhydrous solutions of reaction mixtures were transferred *via* an oven dried syringe or cannula. Chemicals were purchased from commercial sources including dichloromethane (DCM), *n*-hexane, ethyl acetate (EA), methanol (MeOH), tetrahydrofuran (THF), acetonitrile (MeCN), acetone and petroleum ether (PE) were purchased from Beijing Chemical Factory (Beijing, China) and Beijing Tong Guang Fine Chemicals Company (Beijing, China). Column chromatography was performed using silica gal (normal phase, 200 – 300 mesh) from Yantai Xinnuo Chemical Co., Ltd., usually with petroleum ether (PE, bp 60 – 90 °C) and ethyl acetate (EA) as eluent. Reactions were monitored by thin-layer chromatography (TLC) on G254 silica gal plates (0.2 mm) from Yantai Xinnuo Chemical Co., Ltd. The plates were visualized by UV light. ^{1}H, ^{13}C, and ^{19}F NMR spectra were recorded on a JEOL Delta (400 MHz or 600 MHz) spectrometer with $CDCl_3$, DMSO-d_6 or acetone-d_6 as solvents and deuterated solvent residual peak or tetramethylsilane (TMS) as internal standard. Coupling constants (J) are reported in Hertz (Hz), and the chemical shifts (δ) were reported in parts per million (ppm). Multiplicities are indicated as singlet (s), doublet (d), triplet (t), quartet (q), pentet (p), doublet of doublets (dd), triplet of doublets (td), doublet of triplets (dt), quartet of doublets (qd), doublet of doublet of doublets (ddd), doublet of doublet of triplets (ddt), doublet of doublet of doublet of doublets (dddd), multiplet (m), and broad (br). HPLC analysis was conducted on SHIMADZU LC-20ADXR instrument with chiral columns (Chiralpak IB N-5, IC and IF, column 4.6 × 250 mm, (Daicel Chemical Ind., Ltd.)). High resolution mass spectra (HRMS) were recorded on a Waters LCT Premier XE mass spectrometer with TOF. The $[\alpha]_D$ were recorded on RUDOLPH Autopol III. Crystallographic data were collected using a Rigaku Oxford Diffraction XtaLAB Synergy diffractometer equipped with a HyPix-6000E area detector at 100 K using Cu Kα (λ = 1.54184 Å) from a PhotonJet micro-focus X-ray source.

2. The beforehand supplementary information of main text

Table S1. Optimization of the reaction conditions.

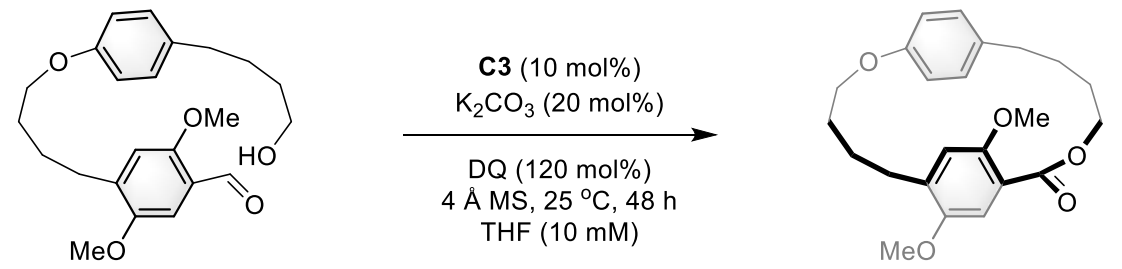

1 2

Entry[a]	Variation from above conditions	Yield(%)[b]	ee(%)[c]
1	**C10**	26	84
2	**C11**	20	95
3	**C12**	28	58[d]
4	Cs_2CO_3	23	96
5	DIPEA	trace	-
6	MTBE	17	82
7	1,4-Dioxane	20	91
8	DPTU	18	93
9	$Mg(OTf)_2$	N.R.	-
10	50 °C	40	94
11	60 °C	46	94
12	THF (30.0 mL), 60 °C	52	94
13	THF (20.0 mL), 70 °C	55	94
14	THF (30.0 mL), 80 °C	53	94

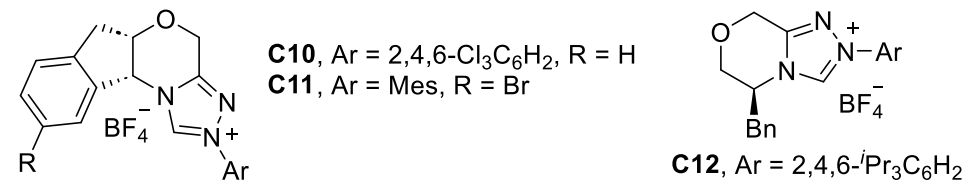

[a]Reaction conditions: A mixture of **1** (0.10 mmol), DQ (0.12 mmol), base (20 mol%), 4 Å MS (50 mg), and NHC (10 mol%) in THF (10.0 mL) was stirred at 25 °C for 48 h. [b]Isolated yield of **2**. [c]The enantiomeric excess (ee) of **2** was determined by HPLC using a chiral stationary phase. [d]The product was obtained as the opposite configuration. DIPEA = N,N'-diisopropylethylamine; MTBE = methyl tert-butyl ether; DPTU = N,N'-diphenyl thiourea.

Table S2. Effects of solvent volume and temperature on product and dimer yields.

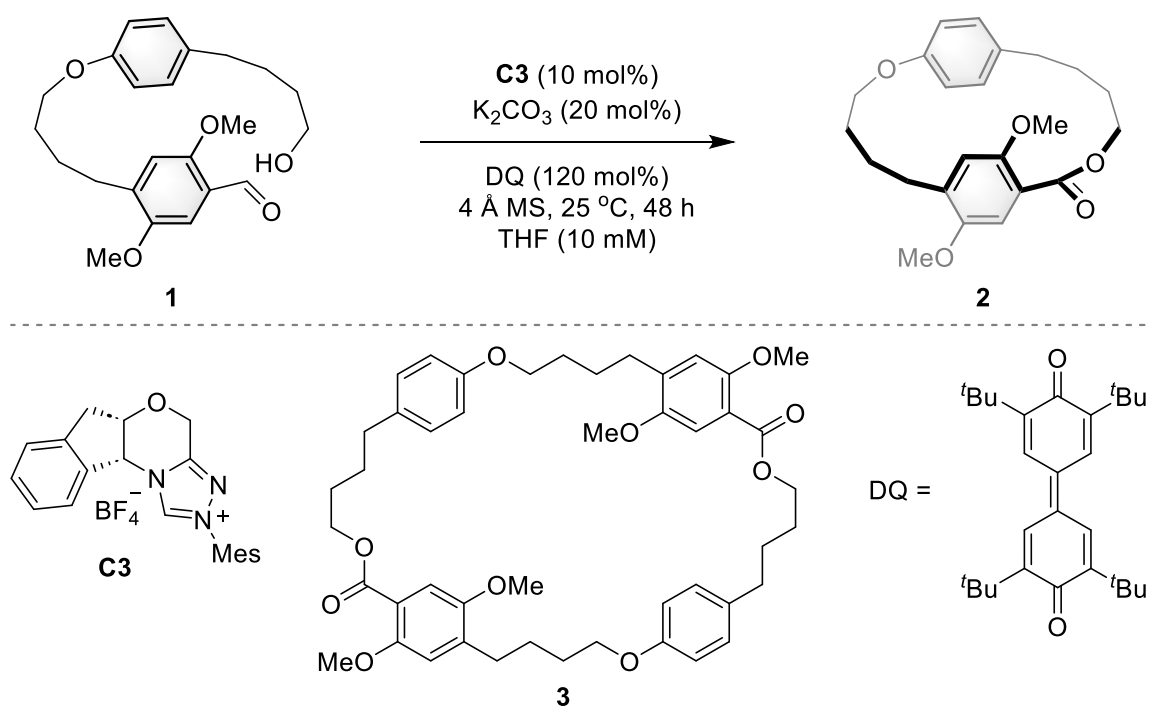

Entry	Variation from above conditions	Yield(%)[a]	ee(%)[b]	Yield(%)[c]
1	THF (5.0 mL)	18	95	17
2	THF (10.0 mL)	36	95	10
3	**1** was added dropwise	36	96	9
4	THF (20.0 mL)	37	95	8
5	THF (30.0 mL)	40	96	4
6	THF (10.0 mL), 70 °C	50	94	11
7	THF (20.0 mL), 70 °C	55	94	9
8	THF (30.0 mL), 70 °C	61	94	5

[a]Isolated yield of **2**. [b]The enantiomeric excess (ee) of **2** was determined by HPLC using a chiral stationary phase. [c]Isolated yield of **3**.

Concentration variation experiments show that the yield of dimeric byproduct **3** decreases markedly upon dilution: from 17% (5 mL) to 4% (30 mL) at room temperature, and from 11% (10 mL) to 5% (30 mL) at 70 °C, confirming that dilution effectively suppresses its formation.

3. Methods and procedures for the synthesis of substrates

1) General procedures used in synthesis

GP1: Phenol alkylation with alkyl bromide (Williamson Ether Synthesis)

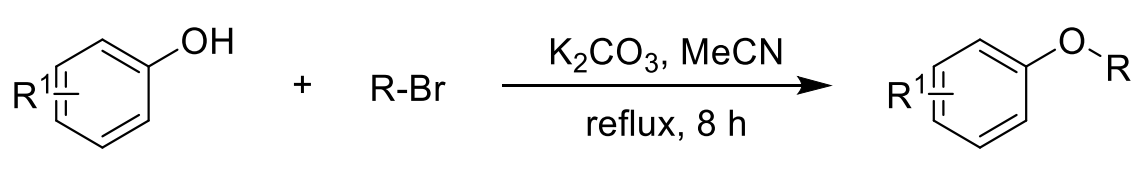

To a solution of phenol (10.0 mmol, 1.0 equiv.) in dry MeCN (50 mL) was added K_2CO_3 (2.76 g, 20.0 mmol, 2.0 equiv.) and RBr (20.0 mmol, 2.0 equiv.). The reaction mixture was heated to reflux for 8 h. After cooling to room temperature, the reaction mixture was filtered and washed with EtOAc (3 × 30 mL). The combined filtrate was concentrated *in vacuo*, and the residue was purified by flash column chromatography on silica gel (PE/EA = 5:1, *v*/*v*) to give the alkylated product.

GP2: TBS protection of alcohol

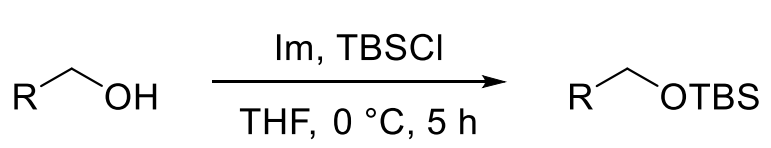

To a solution of alcohol (10.0 mmol, 1.0 equiv.) in dry THF (70 mL) at 0 °C was added imidazole (816 mg, 12.0 mmol, 1.2 equiv.) and TBSCl (1.81 g, 12.0 mmol, 1.2 equiv.). The reaction mixture was warmed to room temperature and stirred for 5 h. Then water was added and the organic phase was separated. The aqueous phase was extracted with EtOAc (3 × 30 mL). The combined organic layers were washed with brine, dried over anhydrous Na_2SO_4, filtered, and concentrated. Purification by flash column chromatography on silica gel (PE/EA = 15:1, *v*/*v*) afforded the TBS ether.

GP3: 9-BBN hydroboration of alkene and subsequent Suzuki-Miyaura coupling

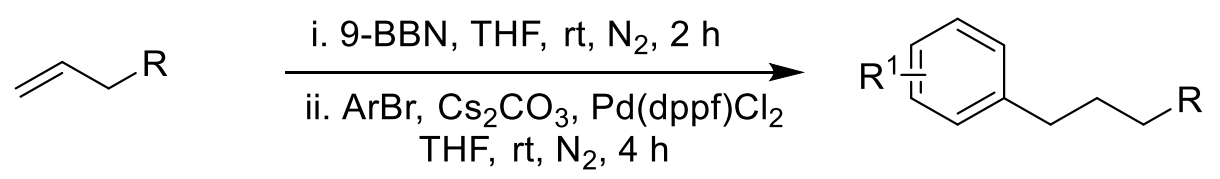

Step i. To a solution of alkene (10.0 mmol, 1.0 equiv.) in dry THF (10 mL) was added 9-BBN (12.0 mmol, 0.5 M in THF, 1.2 equiv.) under N_2 atmosphere. The reaction mixture was stirred at room temperature for 2 h and used directly for next step.

Step ii. In another flask, bromobenzene derivative (8.0 mmol, 1.0 equiv.), Pd(dppf)Cl_2 (146 mg, 0.20 mmol, 0.025 equiv.), and Cs_2CO_3 (7.82 g, 24.0 mmol, 3.0 equiv.) were placed under N_2 atmosphere. The above alkylborane solution was added *via* syringe, and the mixture was stirred at room temperature for 4 h. Then 1N NH_4Cl (30 mL) was slowly added and the organic phase was separated. The aqueous layer was extracted with EtOAc (3 × 30 mL), the combined organic layers were washed with brine, dried over anhydrous Na_2SO_4. After filtration and removing the solvent *in vacuum*, the residue was purified by flash column chromatography on silica gel (PE/EA = 6:1, *v*/*v*) to afford the coupled product.

GP4: Suzuki–Miyaura cross-coupling of aryl bromides and boronic acids

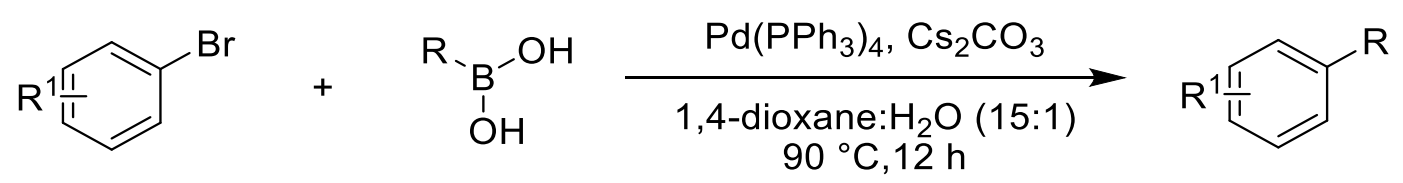

Aryl bromide (5.0 mmol, 1.0 equiv.), boronic acid (15.0 mmol, 3.0 equiv.), $Pd(PPh_3)_4$ (289 mg, 0.25 mmol, 0.05 equiv.) and Cs_2CO_3 (4.89 g, 15.0 mmol, 3.0 equiv.) were added to a dried flask. The flask was evacuated and backfilled with N_2 three times. Then the degassed 1,4-dioxane/H_2O (25 mL, 15:1, *v*/*v*) was added to the reaction, and the mixture was stirred at 90 °C for 12 h. After cooling to room temperature, mixture was diluted with water (30 mL) and extracted with EtOAc (3 × 30 mL). The combined organic layers were dried over anhydrous Na_2SO_4, concentrated, and purified by flash column chromatography on silica gel (PE/EA = 5:1, *v*/*v*) to afford the products.

GP5: TBS deprotection with TBAF

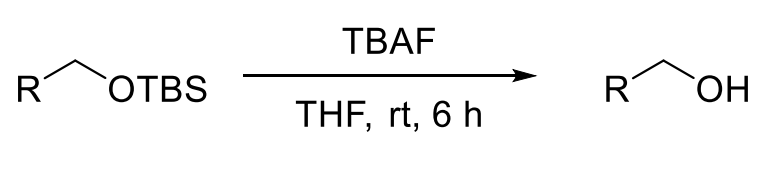

To a solution of TBS ether (6.0 mmol, 1.0 equiv.) in THF (20 mL) was added TBAF (3.14 g, 12.0 mmol, 2.0 equiv.). The reaction mixture was stirred at room temperature for 6 h. Then 1N HCl (10 mL) was added, the organic phase was separated, and the aqueous layer was extracted with EtOAc

(3 × 20 mL). The combined organic layers were washed with brine, dried over anhydrous Na_2SO_4, and concentrated. Purification by flash column chromatography on silica gel (PE/EA = 5:1, *v*/*v*) afforded the desilylated products.

GP6: Tosylation of alcohol

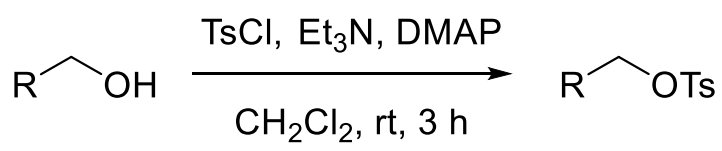

To a solution of alcohol (5.0 mmol, 1.0 equiv.) in dry CH_2Cl_2 (40 mL) was added TsCl (1.14 g, 6.0 mmol, 1.2 equiv.), Et_3N (0.92 mL, 6.5 mmol, 1.3 equiv.) and DMAP (61 mg, 0.5 mmol, 0.1 equiv.). The reaction mixture was stirred at room temperature for 3 h and then was filtered. The solid was washed by EtOAc (3 × 20 mL), and the filtrate concentrated. The residue was purified by flash column chromatography on silica gel (PE/EA = 5:1, *v*/*v*) to afford the product.

GP7: THP protection of alcohol

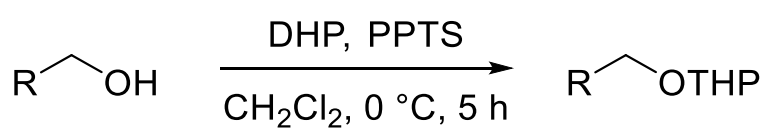

To a solution of alcohol (10.0 mmol, 1.0 equiv.) in dry CH_2Cl_2 (50 mL) was added 3,4-dihydro-2H-pyran (DHP, 1.26g, 15.0 mmol, 1.5 equiv.) and PPTS (126 mg, 0.50 mmol, 0.05 equiv.) at 0 °C. The mixture was stirred for 5 h, then dried and concentrated. And the residue was purified by flash column chromatography on silica gel (PE/EA = 10:1, *v*/*v*) to afford the THP ether product.

GP8: THP deprotection

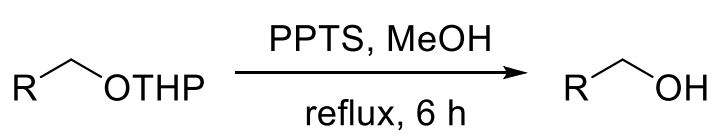

To a solution of THP ether (5.0 mmol, 1.0 equiv.) in MeOH (30 mL) was added PPTS (63 mg, 0.25 mmol, 0.05 equiv.). The reaction mixture was refluxed for 6 h, After cooling to room temperature, the mixture was diluted with water (30 mL) and extracted with EtOAc (3 × 30 mL). The combined organic layers were dried over anhydrous Na_2SO_4, concentrated, and purified by flash column chromatography on silica gel (PE/EA = 5:1, *v*/*v*) to afford the product.

2) Synthesis of specific intermediates and substrates

a) Procedure for the synthesis of aromatic aldehydes

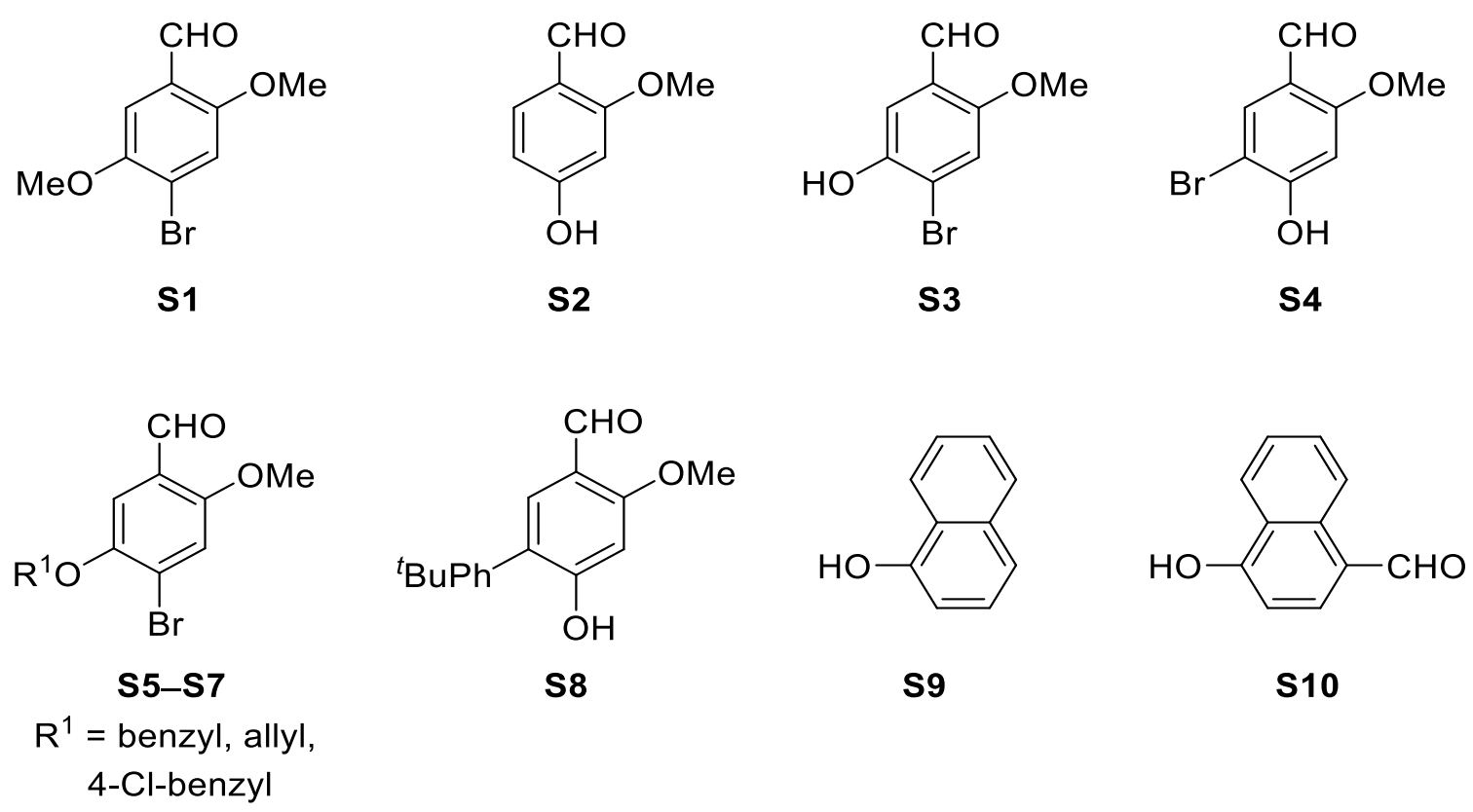

S1, **S2** and **S9** were obtained from commercial sources and used without further purification. Known aromatic aldehydes **S3**,[1] **S4**,[2] **S10**,[3] were prepared according to literature procedure from commercially available materials. The spectra are in accordance with literatures. **S5**–**S7** were prepared from **S3** (2.30 g, 10.0 mmol, 1.0 equiv.) and the corresponding RBr (20.0 mmol, 2.0 equiv.) following **GP1**. **S8** was obtained from **S4** (1.16 g, 5.0 mmol, 1.0 equiv.) and 4-tert-butylphenylboronic (2.67 g, 15.0 mmol, 3.0 equiv.) acid using **GP4** after chromatography on silica gel (PE/EA = 1:1, *v/v*).

b) Procedure for the synthesis of ansa chain S11–S19

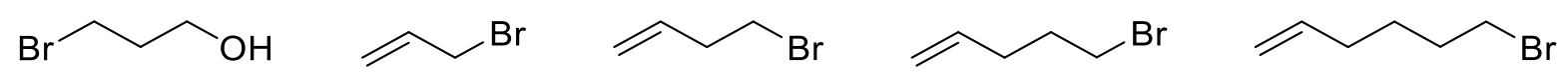

S11 **S12** **S13** **S14** **S15**

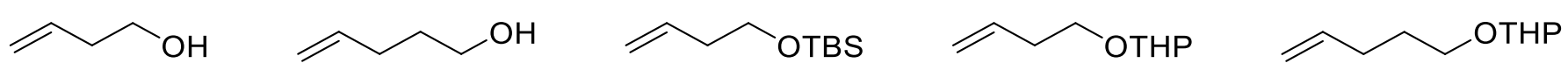

S16 **S17** **S18** **S19** **S20**

S11–**S17** were obtained from commercial sources and used without further purification. **S18** was prepared from **S16** (1.44 g, 20.0 mmol, 1.0 equiv.) by **GP2**. **S19** and **S20** were respectively synthesized from **S16** (721 mg, 10.0 mmol, 1.0 equiv.) and **S17** (861 mg, 10.0 mmol, 1.0 equiv.) following **GP7**.

c) General procedure for the synthesis of substrates 1, S-4–S-32

1, **S-4**–**S-32** represent the substrates of the planar-chiral cyclophanes **2**, **4**–**32**.

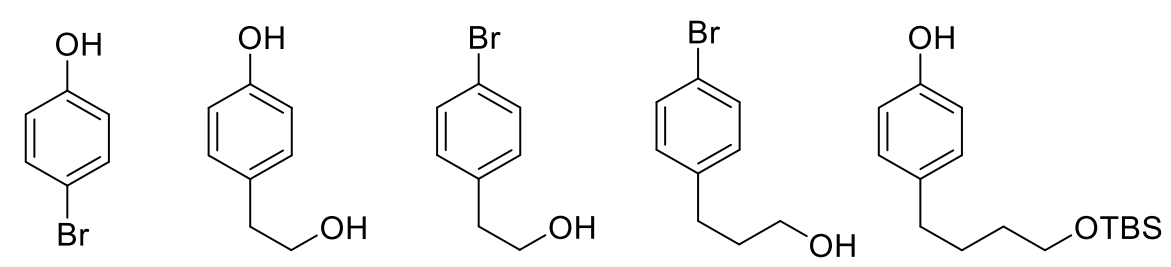

S21 **S22** **S23** **S24** **S25**

S21–**S24** were obtained from commercial sources and used without further purification. **S25** was synthesized from **S18** (1.86 g, 10.0 mmol, 1.0 equiv.) and **S21** (1.38 g, 8.0 mmol, 0.8 equiv.) following **GP3**.

1, **S-4**–**S-6** were prepared as the following procedures.

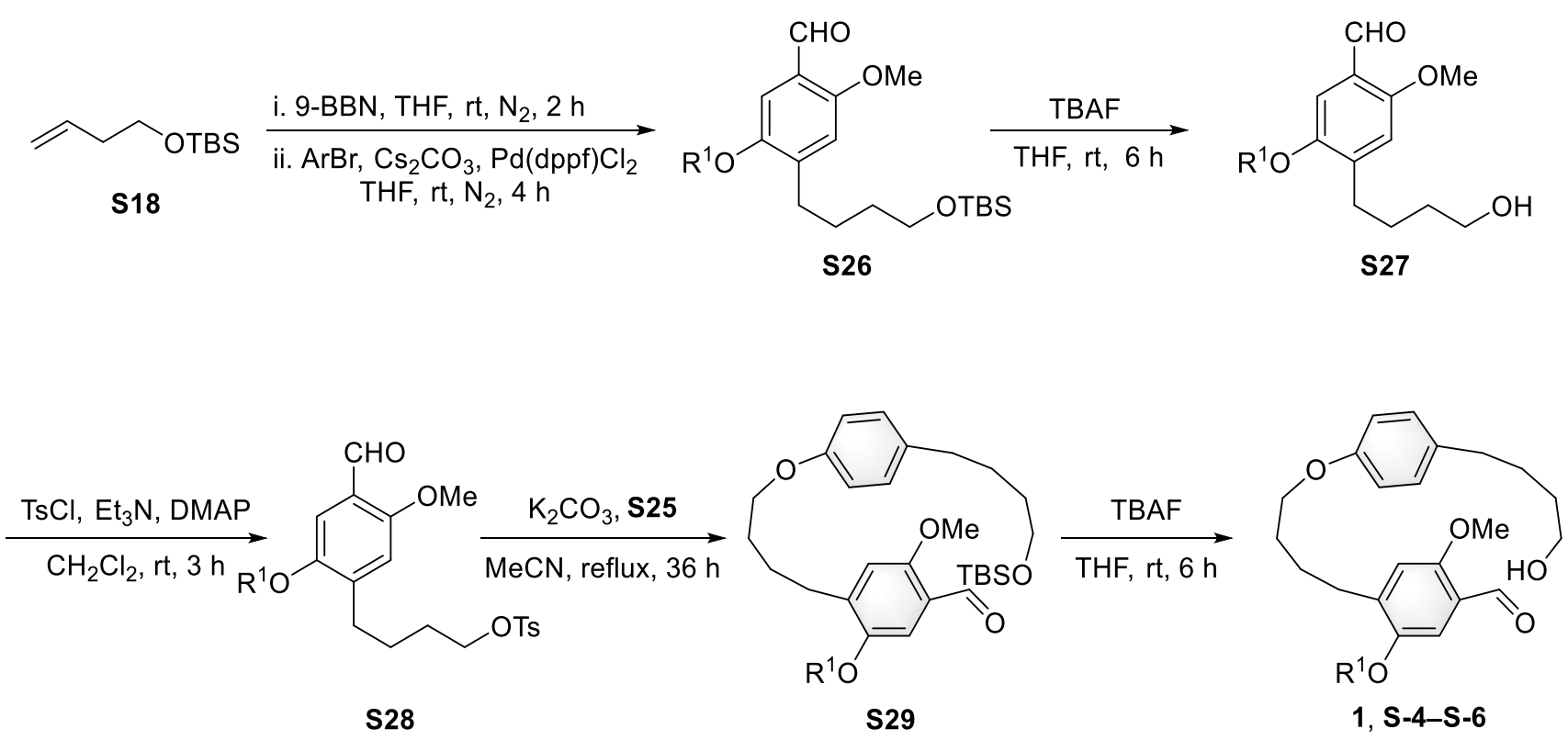

S1 (1.96 g, 8.0 mmol, 0.8 equiv.) or **S5**–**S7** (8.0 mmol, 0.8 equiv.) and **S18** (1.86 g, 10.0 mmol, 1.0 equiv.) underwent the reaction steps **GP3**, **GP5**, and **GP6** to give **S28**. Then to a solution of **S25**

(785 mg, 2.8 mmol, 0.8 equiv.) in dry MeCN (20 mL) was added K_2CO_3 (470 mg, 3.4 mmol, 1.2 equiv.) and **S28** (3.5 mmol, 1.0 equiv.). The reaction mixture was heated to reflux for 36 h. After cooling to room temperature, the reaction mixture was filtered and washed with EtOAc (3 × 15 mL). The combined filtrate was concentrated *in vacuo*, and the residue was purified by flash column chromatography on silica gel (PE/EA = 5:1, *v/v*) to give **S29**. Finally, the linear precursors **1**, **S-4**–**S-6** were obtained from **S29** (1.3 mmol, 1.0 equiv.) following **GP5**.

S-7–**S-21** were prepared as the following procedures.

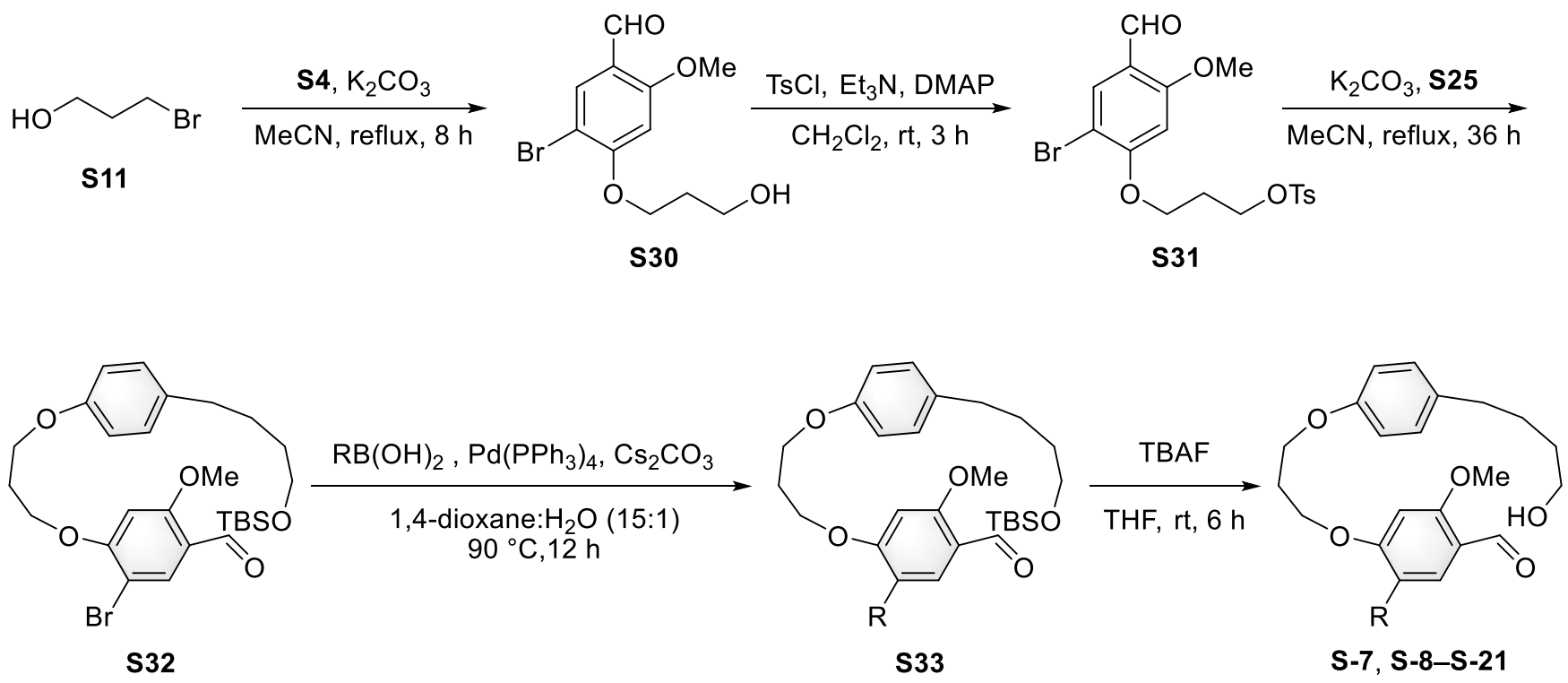

S4 (2.31 g, 10.0 mmol, 1.0 equiv.) and **S11** (2.78 g, 20.0 mmol, 2.0 equiv.) underwent the reaction steps **GP1** and **GP6** to give **S31**. To a solution of **S25** (1.35 g, 4.8 mmol, 0.8 equiv.) in dry MeCN (30 mL) was added K_2CO_3 (995 mg, 7.2 mmol, 1.2 equiv.) and **S31** (2.66 g, 6.0 mmol, 1.0 equiv.). The reaction mixture was heated to reflux for 36 h. After cooling to room temperature, the reaction mixture was filtered and washed with EtOAc (3 × 15 mL). The combined filtrate was concentrated *in vacuo*, and the residue was purified by flash column chromatography on silica gel (PE/EA = 5:1, *v/v*) to give **S32**. Then **S-7** could be directly obtained from **S32** following **GP5**. And **S-8**–**S-21** could be further obtained from **S32** (1.65 g, 3.0 mmol, 1.0 equiv.) following **GP4** and **GP5**.

S-22–**S-26** and **S-32** were prepared as the following procedures.

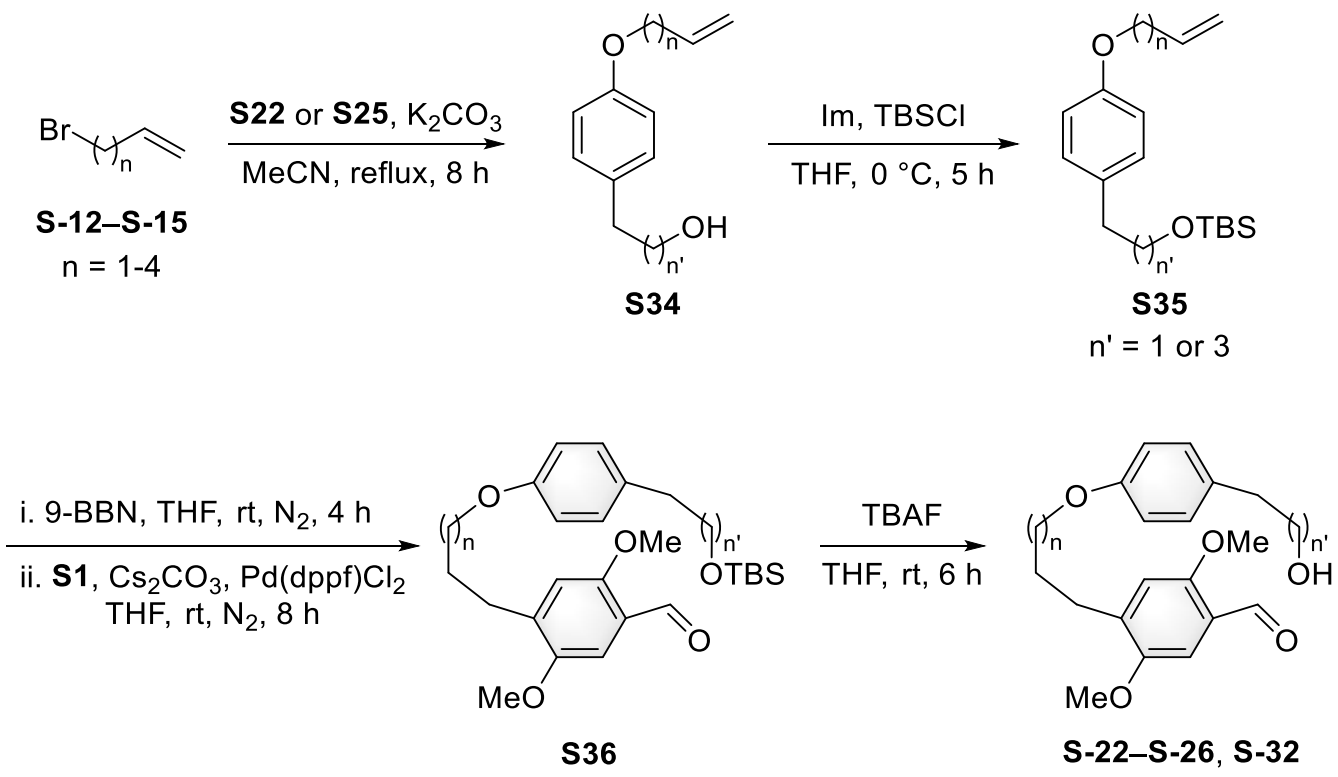

S22 (829 mg, 6.0 mmol, 1.0 equiv.) or **S25** (1.68 mg, 6.0 mmol, 1.0 equiv.) and **S-12**–**S-15** (12.0 mmol, 2.0 equiv.) underwent the reaction steps **GP1** and **GP2** to give **S35**. Then **S35** (4.5 mmol, 1.0 equiv.) reacted with **S1** (882 mg, 3.6 mmol, 0.8 equiv.) *via* **GP3** reaction process to give

S36. Finally, the linear precursors **S-22–S-26**, **S-32** were obtained from **S36** (2.0 mmol, 1.0 equiv.) following **GP5**.

S-27 was prepared as the following procedures.

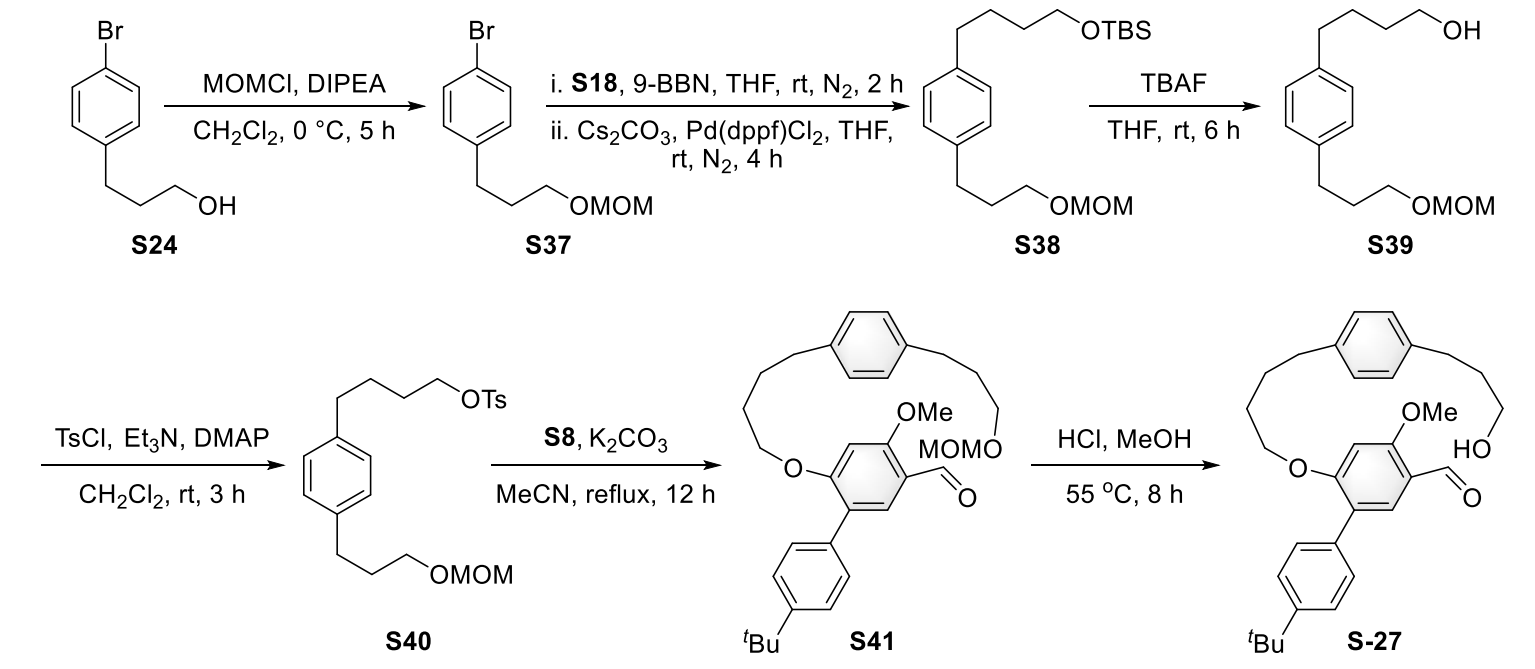

To a solution of **S24** (1.29 g, 6.0 mmol, 1.0 equiv.) in CH_2Cl_2 (50 mL) at 0 °C was added MOMCl (580 mg, 7.2 mmol, 1.2 equiv.) and DIPEA (1.16 g, 9.0 mmol, 1.5 equiv.). The reaction mixture was stirred at room temperature for 5 h. Then saturated aqueous NH_4Cl solution (20 mL) was slowly added and the organic phase was separated. The aqueous phase was extracted with CH_2Cl_2 (3 × 30 mL), the combined organic phases were washed by brine and dried over anhydrous Na_2SO_4. After filtration and concentrated, the residue was purified by flash column chromatography on silica gel (PE/EA = 5:1, *v*/*v*) to afford the MOM-protected product **S37**.

S37 (1.45 g, 5.6 mmol, 0.8 equiv.) and **S18** (1.30 g, 7.0 mmol, 1.0 equiv.) underwent the reaction steps **GP3**, **GP5**, and **GP6** to give **S40**. Then to a solution of **S8** (571 mg, 2.0 mmol, 0.8 equiv.) in dry MeCN (30 mL) was added K_2CO_3 (415 mg, 3.0 mmol, 1.2 equiv.) and **S40** (1.02 g, 2.5 mmol, 1.0 equiv.). The reaction mixture was heated to reflux for 12 h. After cooling to room temperature, the reaction mixture was filtered and washed with EtOAc (3 × 15 mL). The combined filtrate was concentrated *in vacuo*, and the residue was purified by flash column chromatography on silica gel (PE/EA = 5:1, *v*/*v*) to give **S41**.

To a stirred solution of **S41** (519 mg, 1.0 mmol, 1.0 equiv.) in dry MeOH (15 mL) was added two drops (~0.06 mL) of concentrated aqueous hydrochloric acid. The reaction mixture was heated and stirred at 55 °C for 8 h. After cooling to room temperature, a saturated aqueous $NaHCO_3$ (20 mL) and EtOAc (50 mL) were added to the mixture. The phase was separated and the aqueous layer was extracted with EtOAc (3 × 30 mL). The combined organic layers were washed by brine and dried over anhydrous Na_2SO_4. After filtration and concentration, the residue was purified by flash column chromatography on silica gel (PE/EA = 5:1, *v*/*v*) to afford the product **S-27**.

S-28 and **S-29** were prepared as the following procedures.

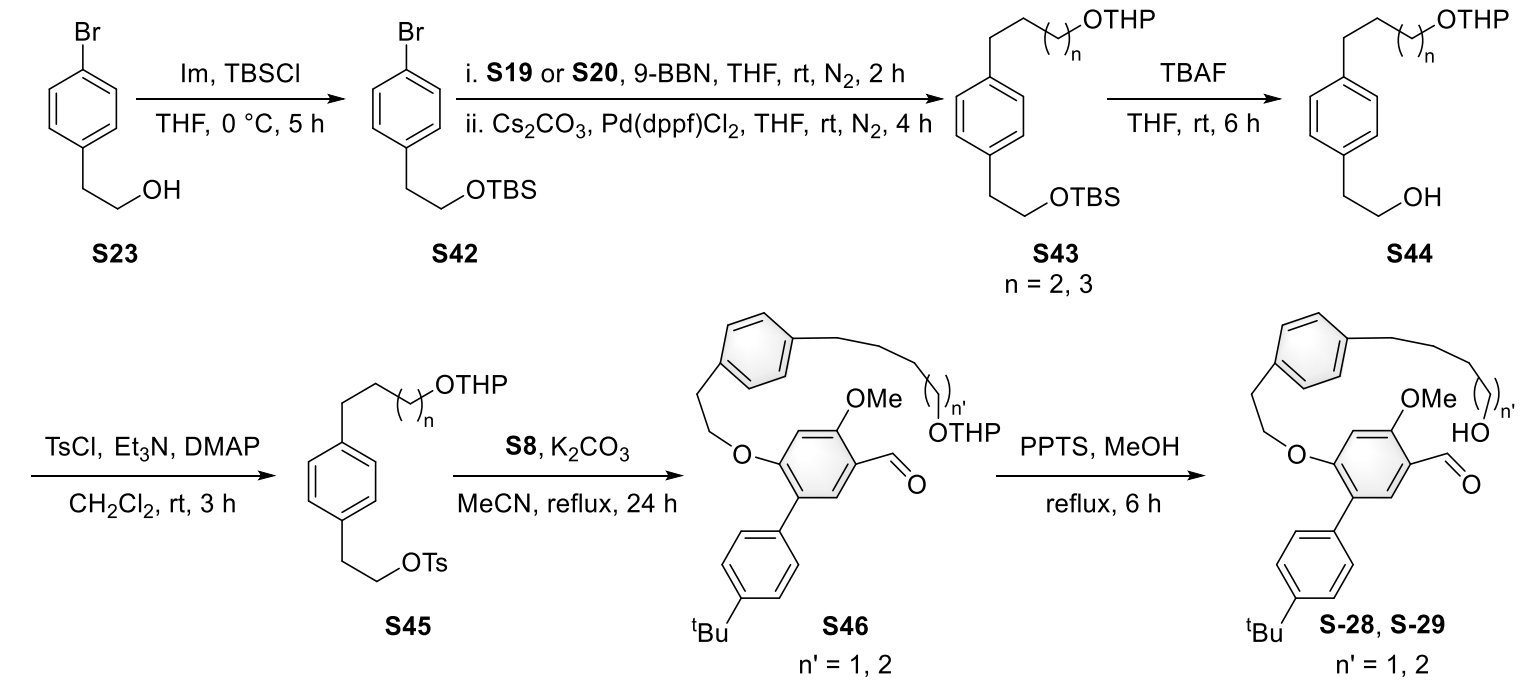

S42 was synthesized from **S23** (1.61 g, 8.0 mmol, 1.0 equiv.) following **GP2**. **S42** (1.20 g, 3.8 mmol, 0.8 equiv.) reacted with **S19** (750 mg, 4.8 mmol, 1.0 equiv.) or **S20** (817 mg, 4.8 mmol, 1.0 equiv.) *via* **GP3** and then underwent the reaction steps **GP5** and **GP6** to give **S45**. Then to a solution of **S8** (428 mg, 1.5 mmol, 0.8 equiv.) in dry MeCN (15 mL) was added K_2CO_3 (318 mg, 2.3 mmol, 1.2 equiv.) and **S45** (1.9 mmol, 1.0 equiv.). The reaction mixture was heated to reflux for 24 h. After cooling to room temperature, the reaction mixture was filtered and washed with EtOAc (3 × 15 mL). The combined filtrate was concentrated *in vacuo*, and the residue was purified by flash column chromatography on silica gel (PE/EA = 5:1, *v*/*v*) to give **S46**. Finally, the linear precursors **S-28** and **S-29** were obtained from **S46** (0.9 mmol, 1.0 equiv.) following **GP8**.

S-30 and **S-31** were prepared as the following procedures.

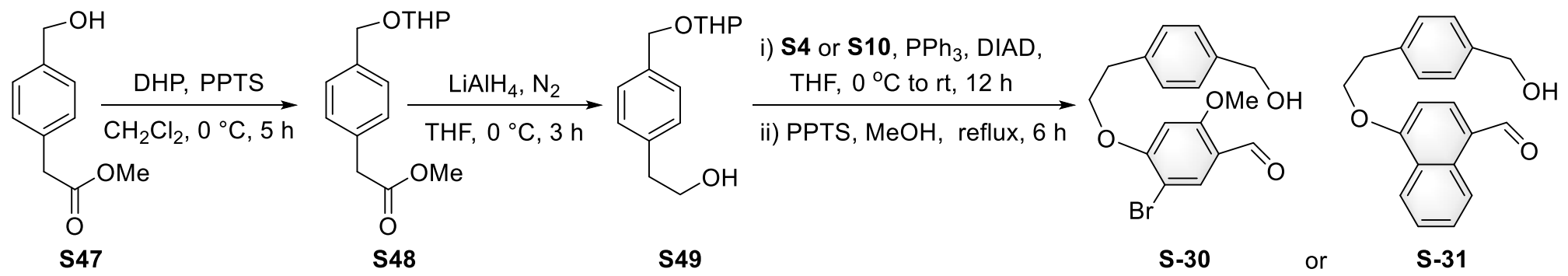

S47 was obtained from commercial sources and used without further purification. **S48** was synthesized from **S47** (1.08 g, 6.0 mmol, 1.0 equiv.) following **GP7**.

To a solution of **S48** (1.32 g, 5.0 mmol, 1.0 equiv.) in anhydrous THF (20 mL) was added $LiAlH_4$ (285.0 mg, 7.5 mmol, 1.5 equiv.) at 0 ºC under N_2 atmosphere. The reaction mixture was stirred at the same temperature for 3 h. Then saturated $NaHCO_3$ (5 mL) was added and the organic phase was separated. The aqueous phase was extracted with EtOAc (3 × 20 mL), the combined organic phases were washed by brine and dried over anhydrous Na_2SO_4. After filtration and removing the solvent *in vacuum*, the residue was purified by flash column chromatography on silica gel (PE/EA = 4:1, *v*/*v*) to afford the products **S49**.

To a solution of **S4** (231 mg, 1.0 mmol, 1.0 equiv.) or **S10** (172 mg, 1.0 mmol, 1.0 equiv.), **S49** (354 mg, 1.5 mmol, 1.5 equiv.), triphenylphosphine (390.0 mg, 1.5 mmol, 1.5 equiv.) in anhydrous THF (5 mL) was added diisopropylazodicarboxylate (DIAD, 300.0 mg, 1.5 mmol, 1.5 equiv.) at 0 °C. The reaction mixture was stirred at room temperature for 12 h. Then saturated aqueous NH_4Cl solution (5 mL) was slowly added and the organic phase was separated. The aqueous phase was extracted with EtOAc (3 × 10 mL), the combined organic phases were washed by brine and dried over anhydrous Na_2SO_4. After filtration and concentrated *in vacuum*, the crude product was dissolved in MeOH (10 mL) and PPTS (13 mg, 0.05 mmol, 0.05 equiv.) was added for reaction following the **GP8** to afford the substrates **S-28** and **S-29**.

3) General procedure for the NHC-catalyzed enantioselective macrocyclization reaction

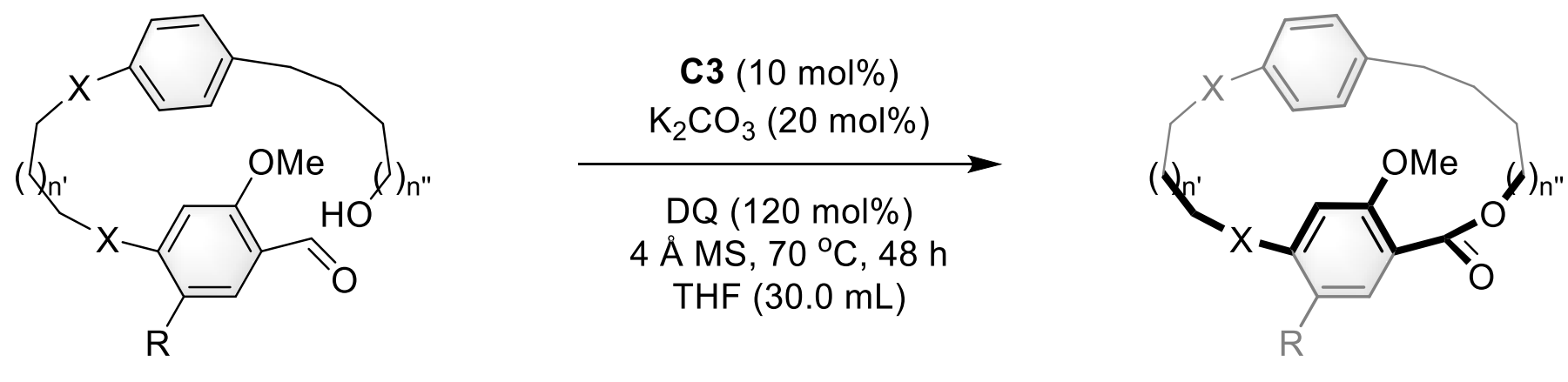

To a 50 mL pressure tube equipped with a magnetic stirring bar was added substrates **1** and **S-4–S-32** (0.10 mmol, 1.0 equiv.), NHC precursor **C3** (4.2 mg, 0.01 mmol, 0.1 equiv.), K_2CO_3 (2.76 mg, 0.02 mmol, 0.2 equiv.), DQ (49.0 mg, 0.12 mmol, 1.2 equiv.) and 4 Å MS (50 mg). The tube was closed with a septum, evacuated, and refilled with nitrogen (3 cycles). After that, the tube was transferred into a glovebox. Under nitrogen atmosphere in the glovebox, the septum was removed, dry THF (30.0 mL) was added, and the tube was sealed with the matching pressure tube cap. The tube was taken out of the glovebox and placed in a preheated oil bath at 70 °C. The reaction mixture was stirred at this temperature for 48 h. Upon the reaction completion (monitored by TLC), the solvent was evaporated and the residue was purified by silica gel column chromatography to afford the [*m.n*]paracyclophanes.

Note: Racemic samples for the standard of chiral HPLC spectra were prepared using racemic NHC precursor **S50** as catalyst.

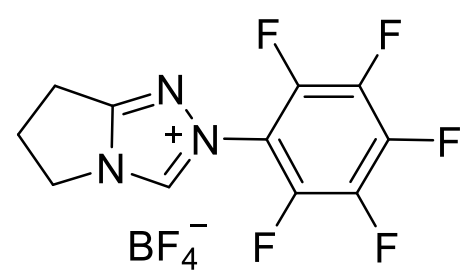

S50

4) Scale-up experiment of 7

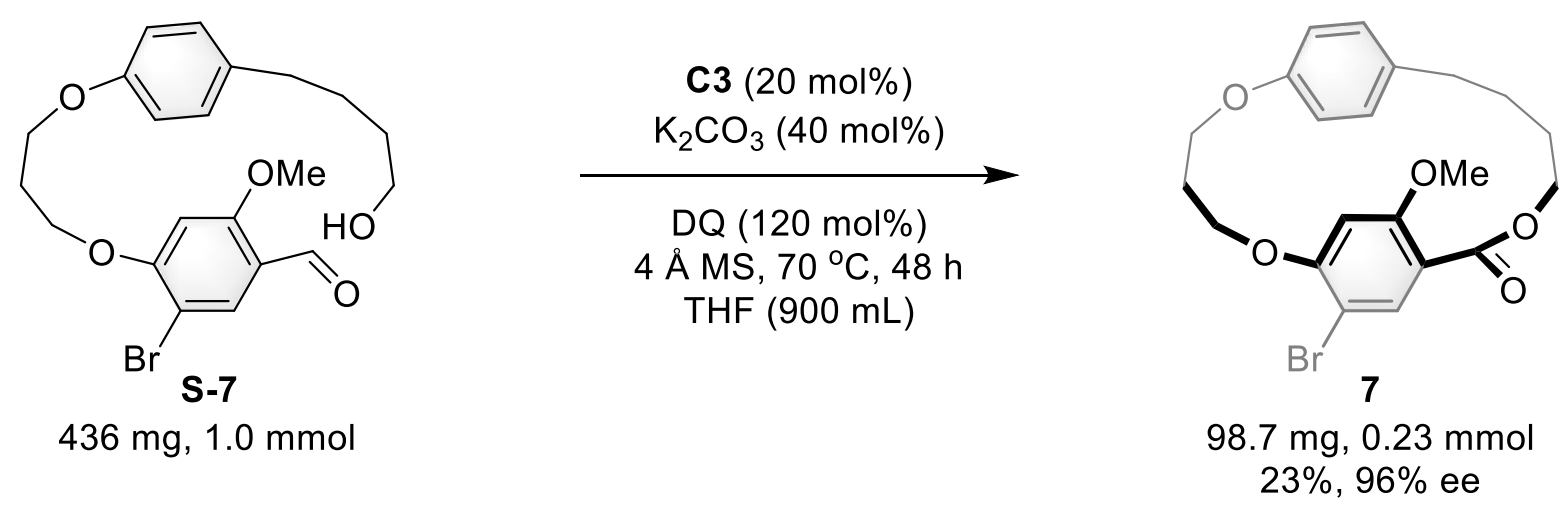

To a 2 L round-bottomed flask equipped with a magnetic stirring bar was added substrates **S-7** (436 mg, 1.0 mmol, 1.0 equiv.), NHC precursor **C3** (83.8 mg, 0.20 mmol, 0.2 equiv.), K_2CO_3 (55.2 mg, 0.40 mmol, 0.4 equiv.), DQ (490 mg, 1.2 mmol, 1.2 equiv.), and 4 Å MS (500 mg). The flask was transferred into a glovebox. Under a nitrogen atmosphere in the glovebox, dry THF (900 mL) was added, and a reflux condenser capped with septum was attached to the flask. After removal, a nitrogen balloon was connected to the top of the condenser. The assembled apparatus was then placed in an oil bath preheated to 70 °C, and the mixture was stirred at reflux for 48 h. The solvent was evaporated and the residue was purified by silica gel column chromatography to afford the **7** (23% yield, 98.7 mg, 96% *ee*).

4. Derivatization and conformational stability studies

1) Derivatization of the planar-chiral cyclophanes

34 and **35** were prepared as the following procedures.[4]

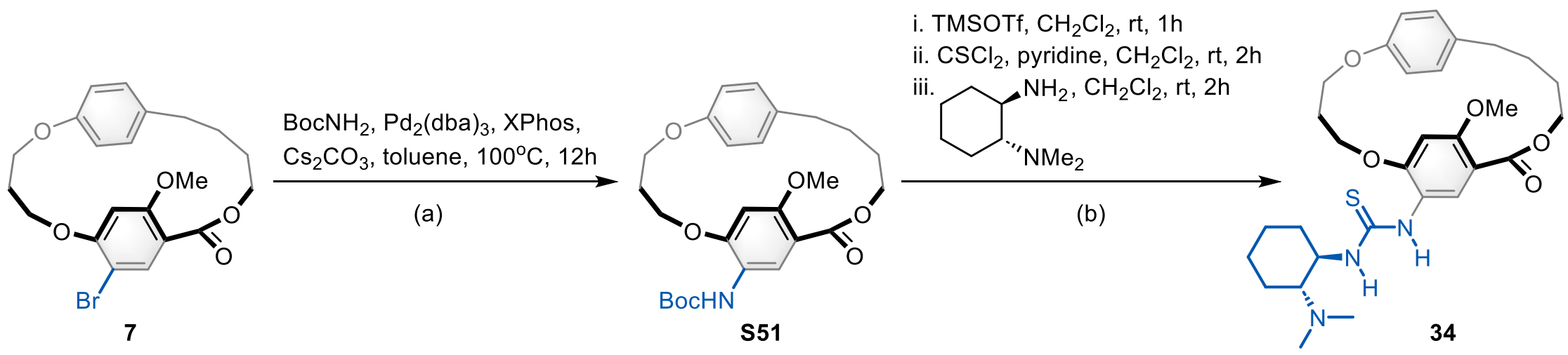

(a) To a solution of **7** (217.7 mg, 0.50 mmol, 1.0 equiv.) in toluene (5 mL) were added $BocNH_2$ (76.1 mg, 0.60 mmol, 1.2 equiv.), Cs_2CO_3 (244.3 mg, 0.75 mmol, 1.5 equiv.), XPhos (71.5 mg, 0.15 mmol, 0.3 equiv.) and $Pd_2(dba)_3$ (45.8 mg, 0.05 mmol, 0.1 equiv.) sequentially under N_2 atmosphere, and the reaction was stirred at 100 °C for 12 h. The progress of the reaction was monitored by TLC. Upon completion, the reaction was cooled to room temperature and diluted with EtOAc. The mixture was filtered through a pad of celite, washed with EtOAc and concentrated. The residue was purified by flash column chromatography on silica gel (PE/EA = 10:1, *v*/*v*) to afford the Boc-protected product **S51**.

(b) To a solution of **S51** (212.2 mg, 0.45 mmol, 1.0 equiv.) in CH_2Cl_2 (4 mL) was added TMSOTf (0.24 mL, 1.35 mmol, 3.0 equiv.) dropwise at room temperature and the reaction was stirred for 1 h. The progress of the reaction was monitored by TLC. Upon completion, the reaction mixture was quenched with saturated aqueous $NaHCO_3$ solution (2 mL) and extracted with CH_2Cl_2 (3 × 5 mL). The combined organic phases were washed by brine and dried over anhydrous Na_2SO_4. After filtration and removing the solvent *in vacuum*, the crude free amine was obtained and used directly for the next step. To a solution of above free amine and pyridine (48.5 µL, 0.60 mmol, 1.5 equiv.) in dry CH_2Cl_2 (4 mL) was added $CSCl_2$ (45.7 µL, 0.60 mmol, 1.5 equiv.). The reaction was stirred at room temperature for 2 h, and the progress of the reaction was monitored by TLC. Upon completion, the mixture was directly purified by quick column chromatography on silica gel (PE/EA = 10:1, *v*/*v*) to afford the desired product. The above product was dissolved in CH_2Cl_2 (4 mL), and (1*R*,2*R*)-*N*,*N*-dimethylcyclohexane-1,2-diamine (0.124 mL, 0.80 mmol, 2.0 equiv.) was added. The mixture was stirred at room temperature for 2 h, and the progress of the reaction was monitored by TLC. Upon completion, the mixture was concentrated and directly purified by column chromatography on silica gel (CH_2Cl_2/MeOH = 20:1, *v*/*v*) to afford the desired product **34**.

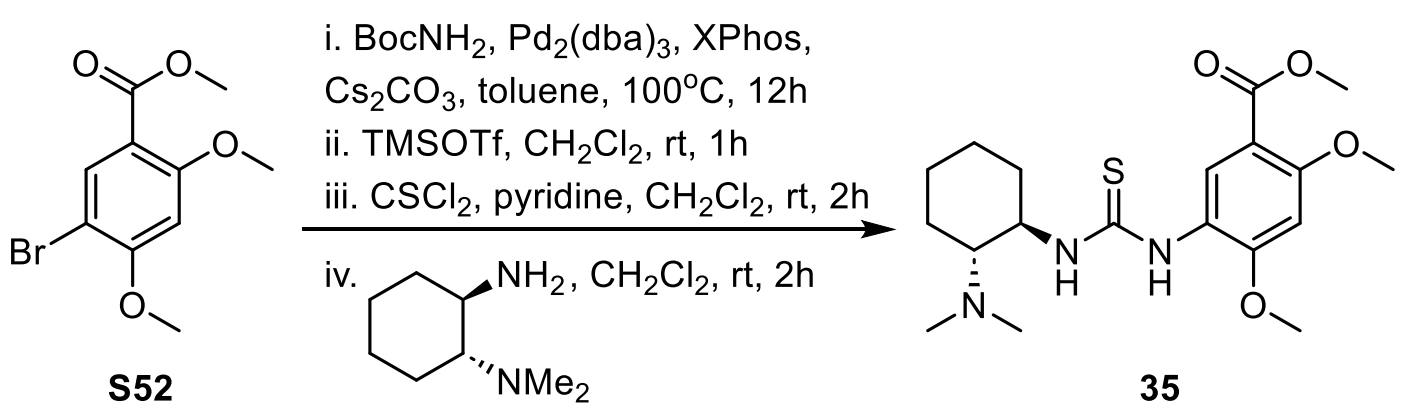

Following the same steps as described above, **35** was obtained from commercially available **S52**.

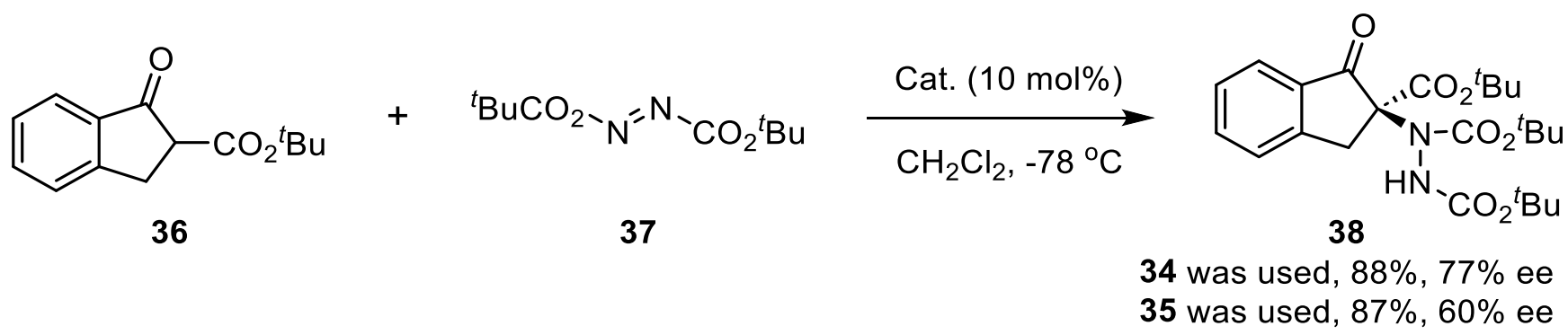

According to the known procedure,[5] using compound **34** (>20:1 dr) as catalyst gave compound **38** in 88% yield and 77% *ee* (40.7 mg, 0.10 mmol scale), while using compound **35** as catalyst gave it in 87% yield and 60% *ee* (40.2 mg, 0.10 mmol scale). This compound is known and the spectroscopic data match those reported. ^{1}**H NMR** (400 MHz, $CDCl_3$) δ 7.81 – 7.67 (m, 1H), 7.63 – 7.56 (m, 1H), 7.50 – 7.41 (m, 1H), 7.38 – 7.30 (m, 1H), 6.86 – 6.16 (m, 1H), 4.26 – 3.44 (m, 2H), 1.51 – 1.22 (m, 27H). $[\alpha]_D^{25}$ (cat. **34**) = +58.4 (c = 1.16, CH_2Cl_2); $[\alpha]_D^{25}$ (cat. **35**) = +44.9 (c = 1.58, CH_2Cl_2). **HPLC**: Chiralpak IE column, 88:12 hexanes/isopropanol, flow rate = 0.5 mL/min, λ = 254 nm, t_R (cat. **34**) = 37.25 min (minor), 40.00 min (major); t_R (cat. **35**) = 37.64 min (minor), 40.17 min (major).

2) Racemization experiment of 2, 13, 25, 26, 27 and 28

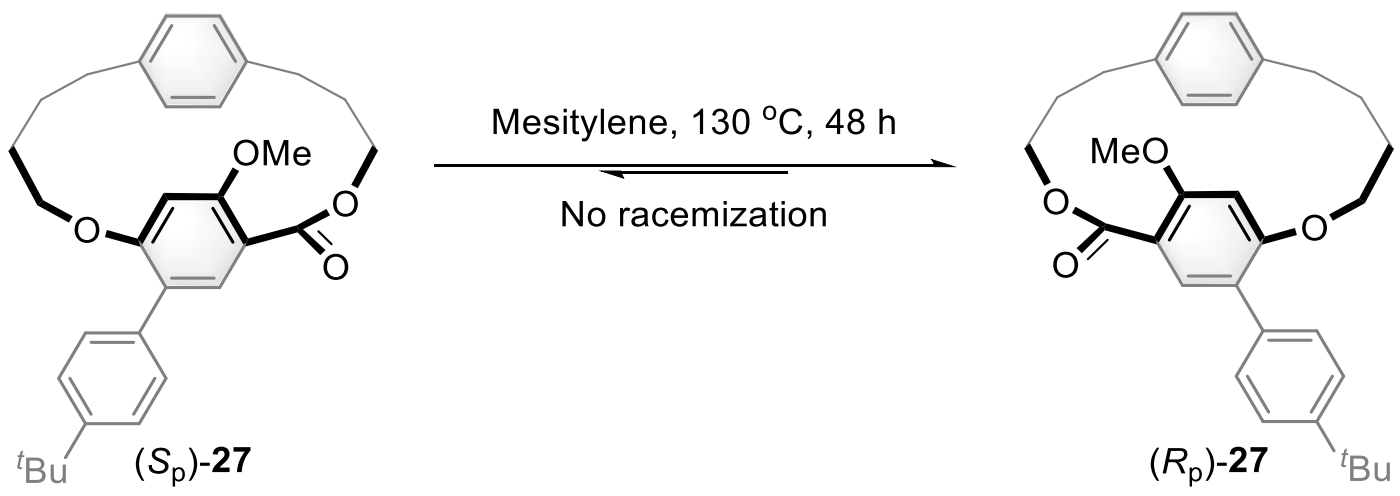

About 3 mg of enantio-enriched (**27**) was heated at 130 °C in mesitylene (1.0 mL). Samples of 20 μL of this solution were injected on Chiralpak IC (80:20 hexanes/isopropanol, 1.0 mL/min, UV detection at 254 nm) to monitor the percentage decrease of the second eluted enantiomer over time. After 48 h, no epimerization was detected.

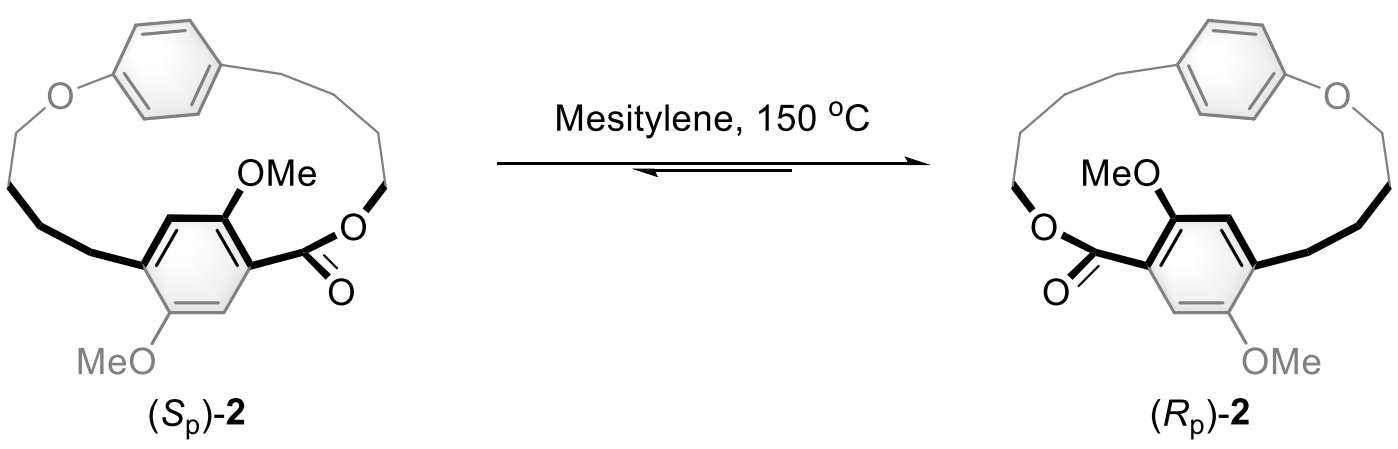

Compound (S_p)-**2** (4 mg, 0.01 mmol, 88.0% *ee*) was dissolved in mesitylene (1.0 mL) in a sealed tube. The reaction mixture was allowed to stir at 150 °C (pre-heated metal module) under air. Within the same time interval, small sample (30 μL) was removed *via* a syringe and diluted by isopropanol, then sample was injected into the HPLC to measure the enantiomeric excess.

Table S3. The racemization experiment of (S_p)-**2** at 150 °C.

time (s)	ee_t (%)	ee_t/ee_0	$\ln(ee_0/ee_t)$
0	88.03	1.0000	0.0000
43200	80.18	0.9108	0.0933
86400	73.18	0.8313	0.1847
129600	66.99	0.7610	0.2731
172800	61.62	0.7000	0.3566
216000	55.93	0.6354	0.4536

259200	51.70	0.5873	0.5322
302400	47.21	0.5363	0.6231

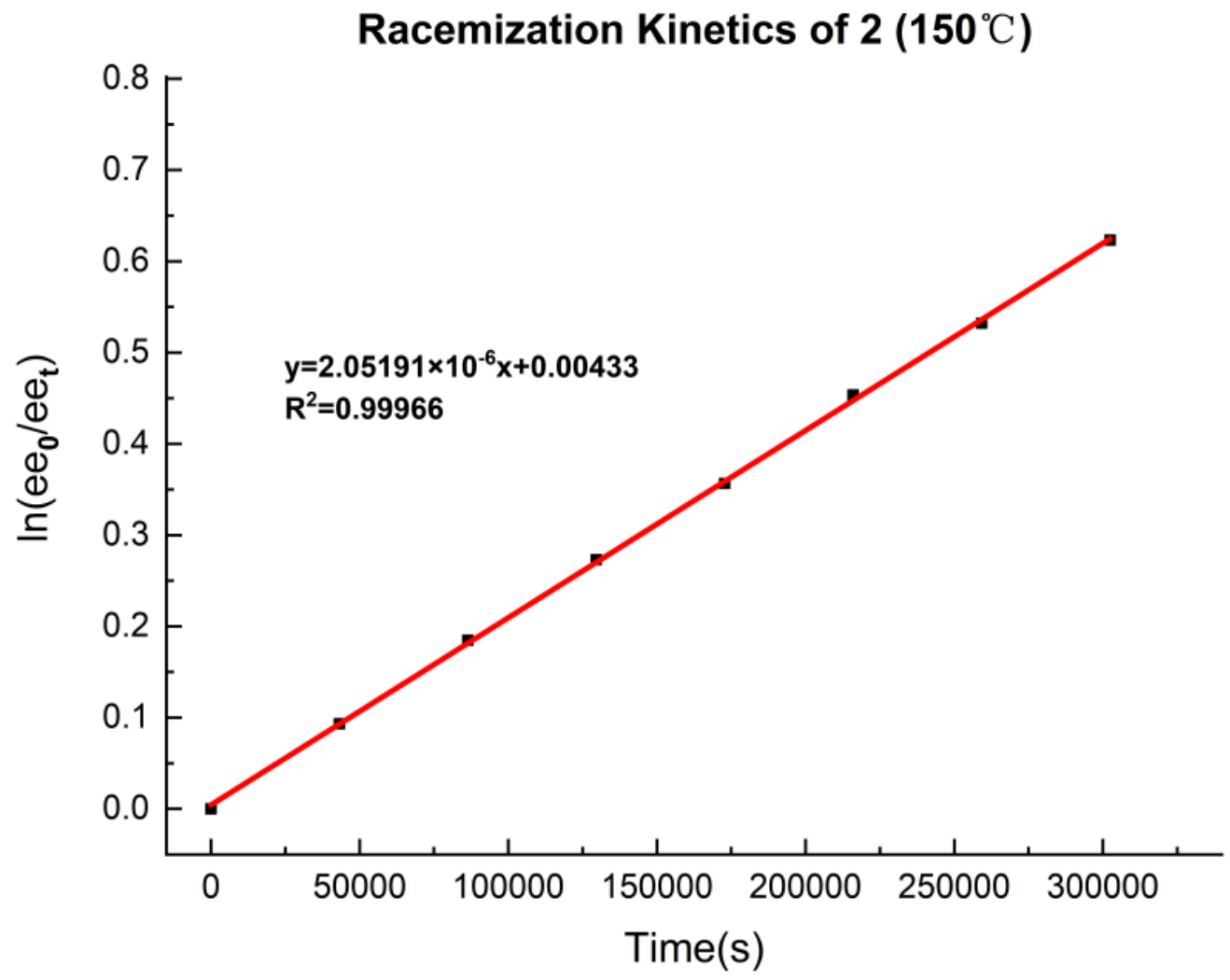

Scheme S1. The racemization curves of (S_p)-**2** at 150 °C.

$T_1 = 150\ °C = 423.15\ K$, $T_2 = 25\ °C = 298.15\ K$

$k_{rac}(150\ °C) = 2.05 \times 10^{-6}\ s^{-1}$

$\tau_{1/2}(150\ °C) = \ln 2 / k_{rac}(150\ °C) = 337805\ s = 5630.08\ min = 93.83\ h$

$k_{ent}(150\ °C) = k_{rac}(150\ °C) / 2 = 1.03 \times 10^{-6}\ s^{-1}$

$\Delta G^{\ddagger}_{ent} = RT_1 \cdot \ln((k_B T_1)/(h k_{ent})) = 153.389\ kJ \cdot mol^{-1} = 36.67\ kcal \cdot mol^{-1}$

$k_{rac}(T_2) = 2 \cdot k_B T_2 \cdot h^{-1} \cdot e\text{^}(-\Delta G^{\ddagger}_{ent} /(RT_2))$

$k_{rac}(25\ °C) = 1.67 \times 10^{-14}\ s^{-1}$

$\tau_{1/2}(25\ °C) = \ln 2 / k_{rac}(25\ °C) = 4.16 \times 10^{13}\ s = 1.16 \times 10^{10}\ h = 4.83 \times 10^{8}\ days$

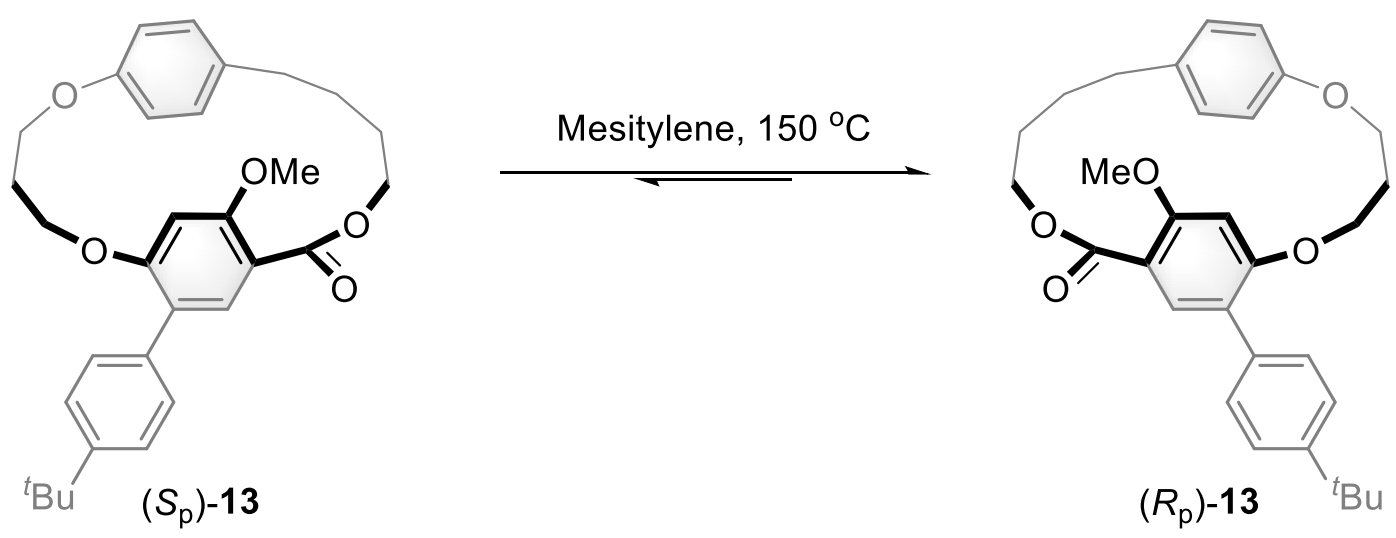

Compound (S_p)-**13** (4 mg, 0.01 mmol, 64.8% *ee*) was dissolved in mesitylene (1.0 mL) in a sealed tube. The reaction mixture was allowed to stir at 150 °C (pre-heated metal module) under air. Within the same time interval, small sample (30 μL) was removed *via* a syringe and diluted by isopropanol, then sample was injected into the HPLC to measure the enantiomeric excess.

Table S4. The racemization experiment of (S_p)-**13** at 150 °C.

time (s)	ee_t (%)	ee_t/ee_0	$\ln(ee_0/ee_t)$
0	64.82	1.0000	0.0000
43200	64.02	0.9877	0.0124
86400	63.30	0.9766	0.0238
129600	62.67	0.9668	0.0339
172800	61.94	0.9556	0.0455
216000	61.19	0.9440	0.0577
259200	60.66	0.9358	0.0663

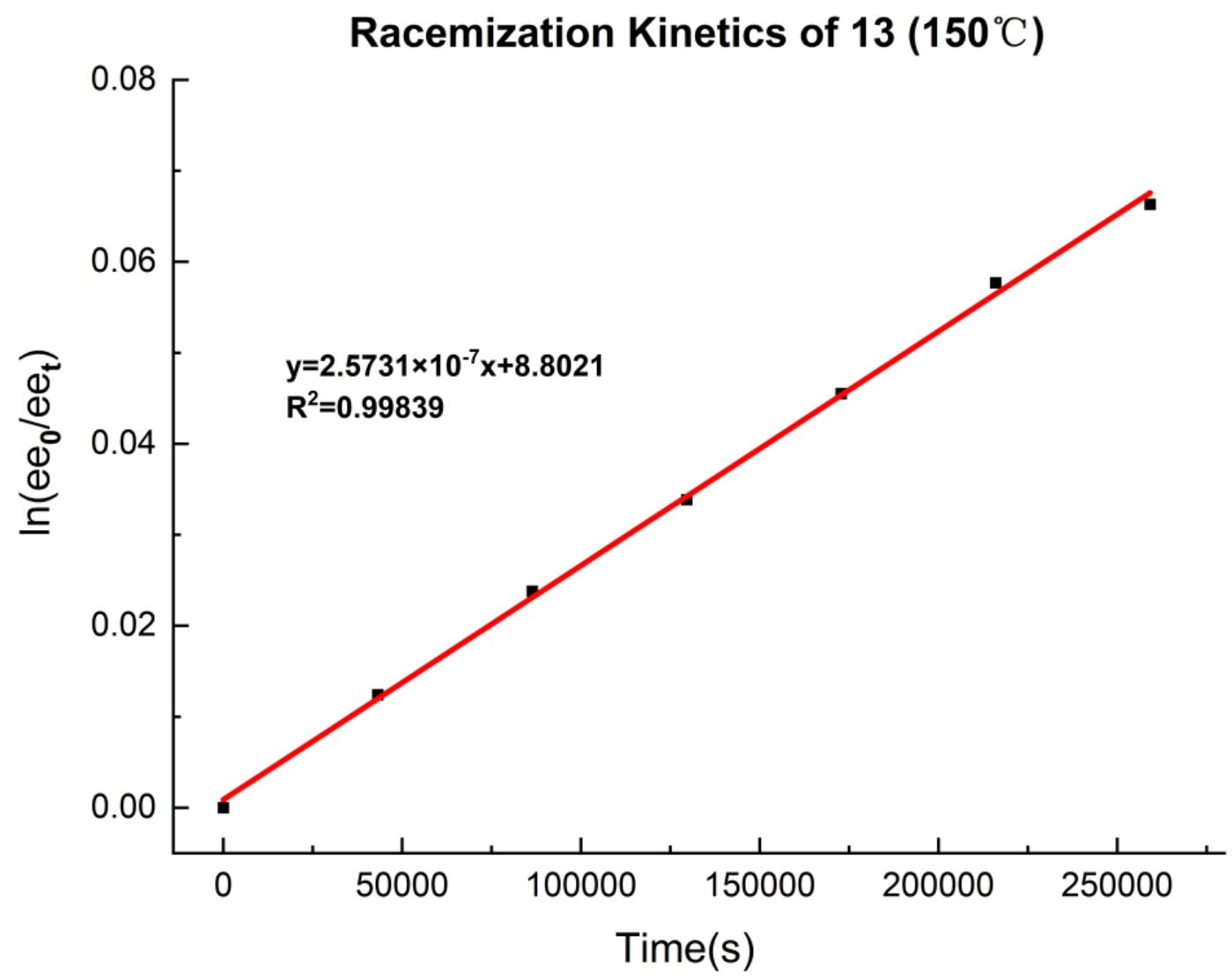

Scheme S2. The racemization curves of (S_p)-**13** at 150 °C.

T_1 = 150 °C = 423.15 K, T_2 = 25 °C = 298.15 K

k_{rac}(150 °C) = 2.57×10^{-7} s^{-1}

$\tau_{1/2}$(150 °C) = ln2 / k_{rac}(150 °C) = 2696300 s = 44938.33 min = 748.28 h = 31 days

k_{ent}(150 °C) = k_{rac}(150 °C) / 2 = 1.29×10^{-7} s^{-1}

$\Delta G^{\ddagger}_{ent} = RT_1\cdot\ln((k_BT_1)/(hk_{ent}))$ = 160.694 kJ·mol^{-1} = 38.41 kcal·mol^{-1}

$k_{rac}(T_2) = 2\cdot k_BT_2\cdot h^{-1}\cdot e$^$(-\Delta G^{\ddagger}_{ent} /(RT_2))$

k_{rac}(25 °C) = 8.75×10^{-16} s^{-1}

$\tau_{1/2}$(25 °C) = ln2 / k_{rac}(25 °C) = 7.92×10^{14} s = 2.20×10^{11} h = 9.17×10^{9} days

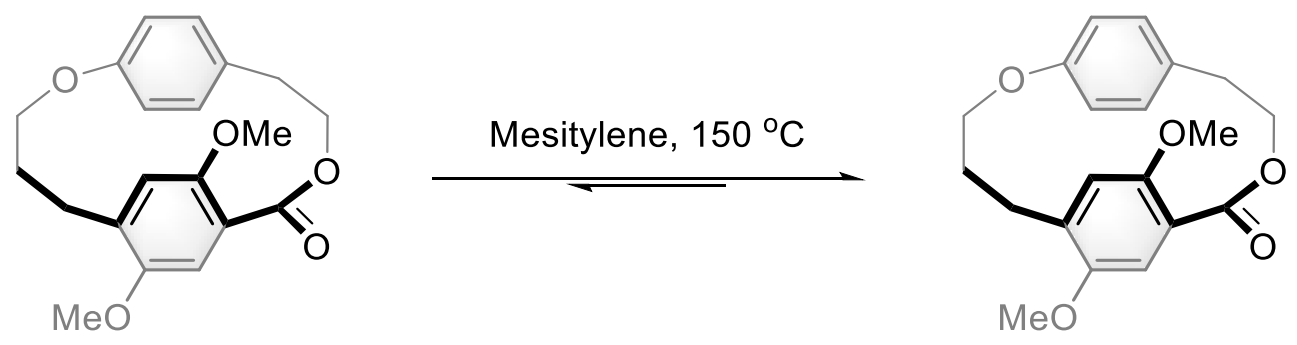

(S_p)-**25** (R_p)-**25**

Compound (S_p)-**25** (3 mg, 0.01 mmol, 94.3% *ee*) was dissolved in mesitylene (1.0 mL) in a sealed tube. The reaction mixture was allowed to stir at 150 °C (pre-heated metal module) under air. Within the same time interval, small sample (30 μL) was removed *via* a syringe and diluted by isopropanol, then sample was injected into the HPLC to measure the enantiomeric excess.

Table S5. The racemization experiment of (S_p)-**25** at 150 °C.

time (s)	ee_t (%)	ee_t/ee_0	$\ln(ee_0/ee_t)$
0	94.30	1.0000	0.0000
43200	93.39	0.9903	0.0097
86400	91.88	0.9743	0.0260
129600	90.44	0.9591	0.0418
172800	89.70	0.9512	0.0500

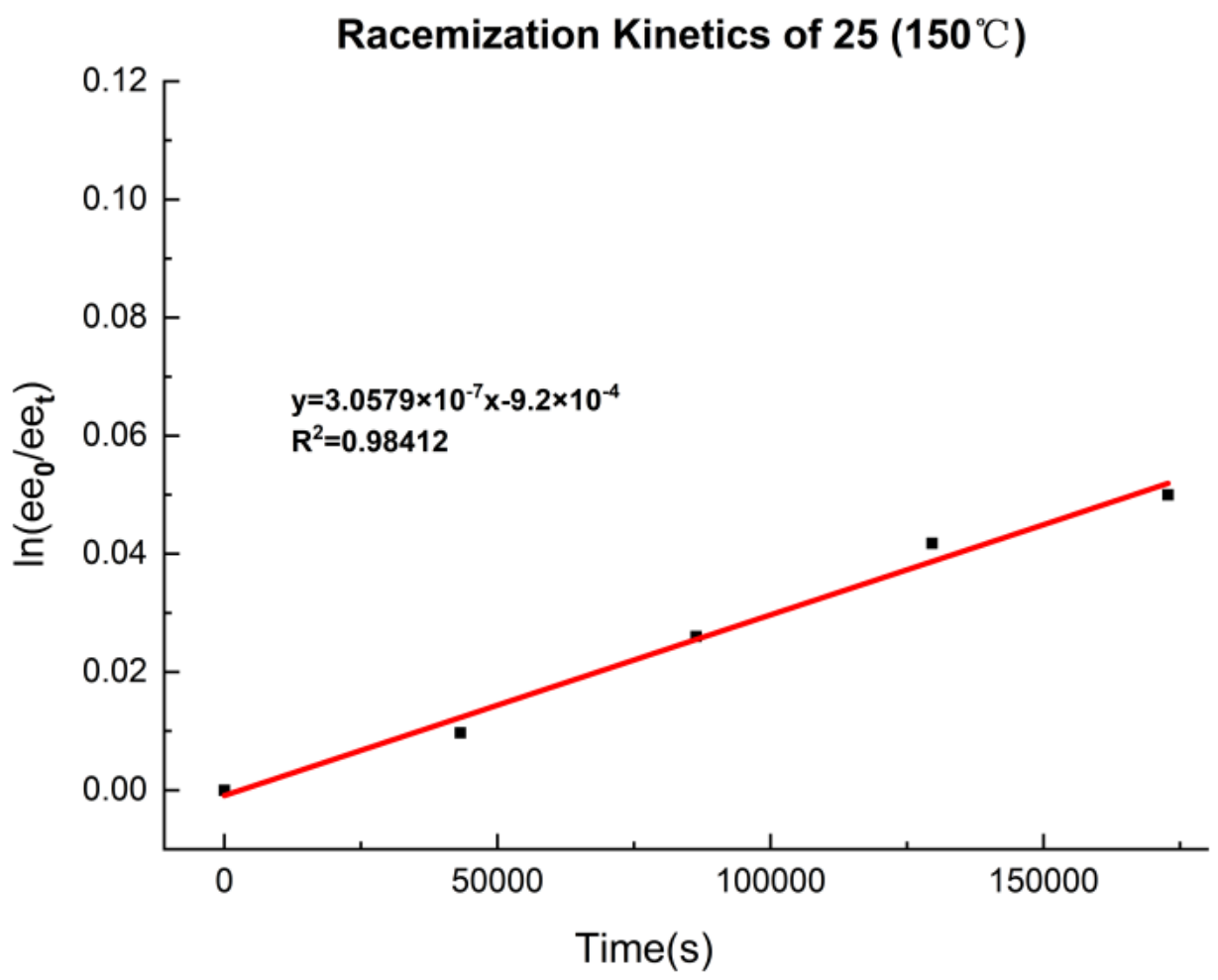

Scheme S3. The racemization curves of (S_p)-**25** at 150 °C.

$T_1 = 150$ °C = 423.15 K, $T_2 = 25$ °C = 298.15 K

$k_{rac}(150\ °C) = 3.06\times10^{-7}\ s^{-1}$

$\tau_{1/2}(150\ °C) = \ln2 / k_{rac}(150\ °C) = 2.267\times10^{-7}$ s = 37783.33 min = 629.79 h = 26 days

$k_{ent}(150\ °C) = k_{rac}(150\ °C) / 2 = 1.528 \times 10^{-7}\ s^{-1}$

$\Delta G^{\ddagger}_{ent} = RT_1\cdot\ln((k_BT_1)/(hk_{ent})) = 160.087\ kJ\cdot mol^{-1} = 38.26\ kcal\cdot mol^{-1}$

$k_{rac}(T_2) = 2\cdot k_BT_2\cdot h^{-1}\cdot e\hat{}(-\Delta G^{\ddagger}_{ent} /(RT_2))$

$k_{rac}(25\ °C) = 1.118 \times 10^{-15}\ s^{-1}$

$\tau_{1/2}$(25 °C) = ln2 / k_{rac}(25 °C) = 6.2 × 10^{14} s = 1.722× 10^{11} h = 7.18× 10^{9} days

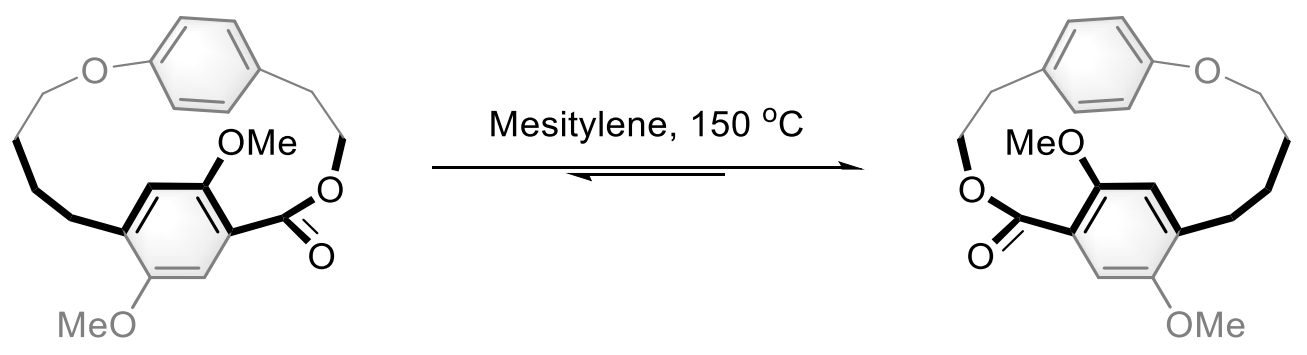

Compound (S_p)-**26** (3 mg, 0.01 mmol, 93.6% *ee*) was dissolved in mesitylene (1.0 mL) in a sealed tube. The reaction mixture was allowed to stir at 150 °C (pre-heated metal module) under air. Within the same time interval, small sample (30 μL) was removed *via* a syringe and diluted by isopropanol, then sample was injected into the HPLC to measure the enantiomeric excess.

Table S6. The racemization experiment of (S_p)-**26** at 150 °C.

time (s)	ee_t (%)	ee_t/ee_0	$\ln(ee_0/ee_t)$
0	93.62	1.0000	0
43200	92.38	0.9868	0.0133
86400	91.78	0.9803	0.0198
129600	90.94	0.9714	0.0290
172800	90.01	0.9614	0.0393
216000	89.30	0.9539	0.0472
259200	88.56	0.9460	0.0556
302400	87.68	0.9366	0.0655

T_1 = 150 °C = 423.15 K, T_2 = 25 °C = 298.15 K

k_{rac}(150 °C) = 2.10×10^{-7} s^{-1}

$\tau_{1/2}$(150 °C) = ln2 / k_{rac}(150 °C) = 3298571 s = 54976.18 min = 916.27 h = 38 days

k_{ent}(150 °C) = k_{rac}(150 °C) / 2 = 1.05 × 10^{-7} s^{-1}

$\Delta G^{\ddagger}_{ent} = RT_1\cdot\ln((k_BT_1)/(hk_{ent}))$ = 161.406 kJ·mol^{-1} = 38.58 kcal·mol^{-1}

$k_{rac}(T_2) = 2\cdot k_BT_2\cdot h^{-1}\cdot e\text{\^{}}(-\Delta G^{\ddagger}_{ent}/(RT_2))$

k_{rac}(25 °C) = 6.57 × 10^{-16} s^{-1}

$\tau_{1/2}$(25 °C) = ln2 / k_{rac}(25 °C) = 1.06 × 10^{15} s = 2.93× 10^{11} h = 1.22× 10^{10} days

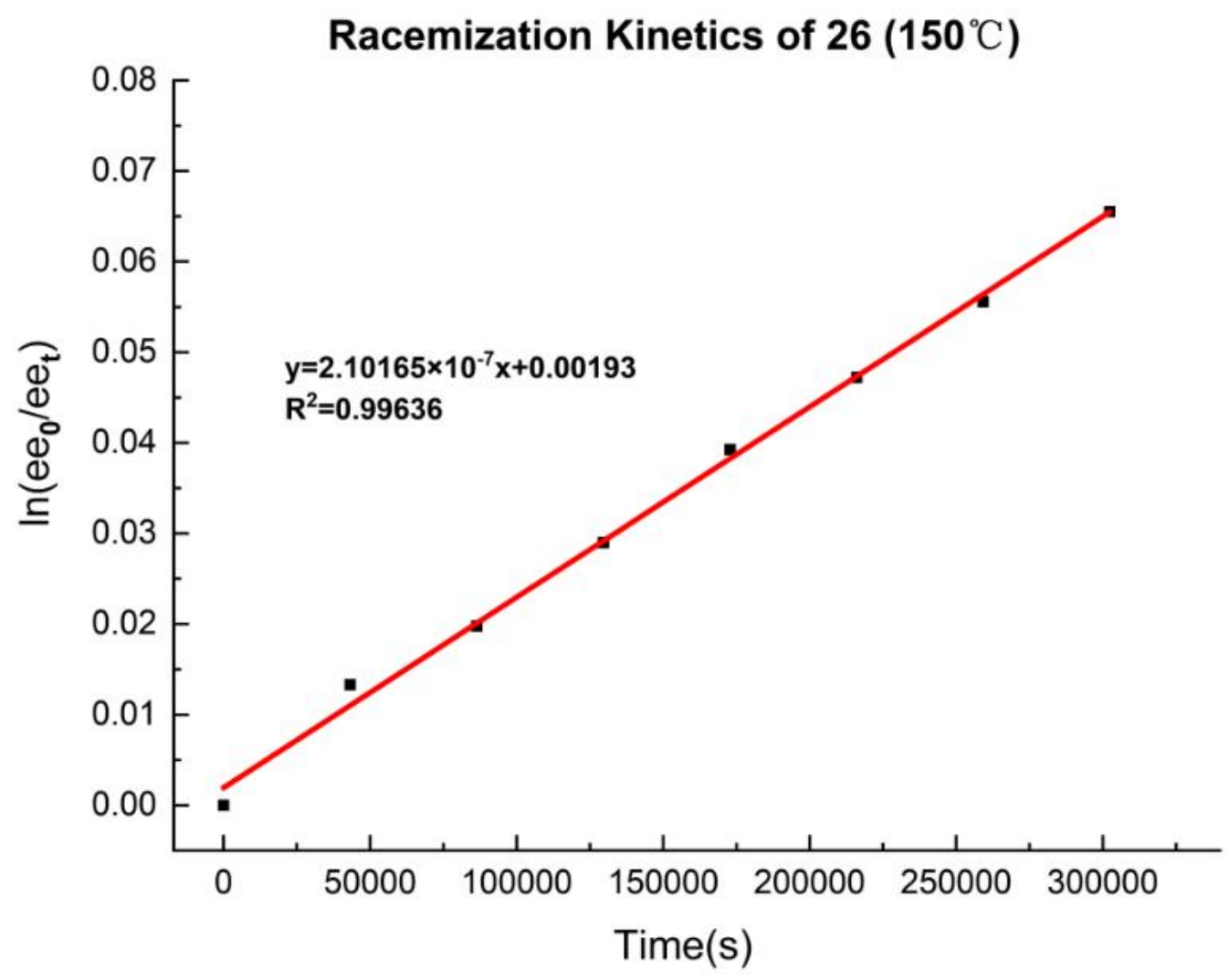

Scheme S4. The racemization curves of (S_p)-**26** at 150 °C.

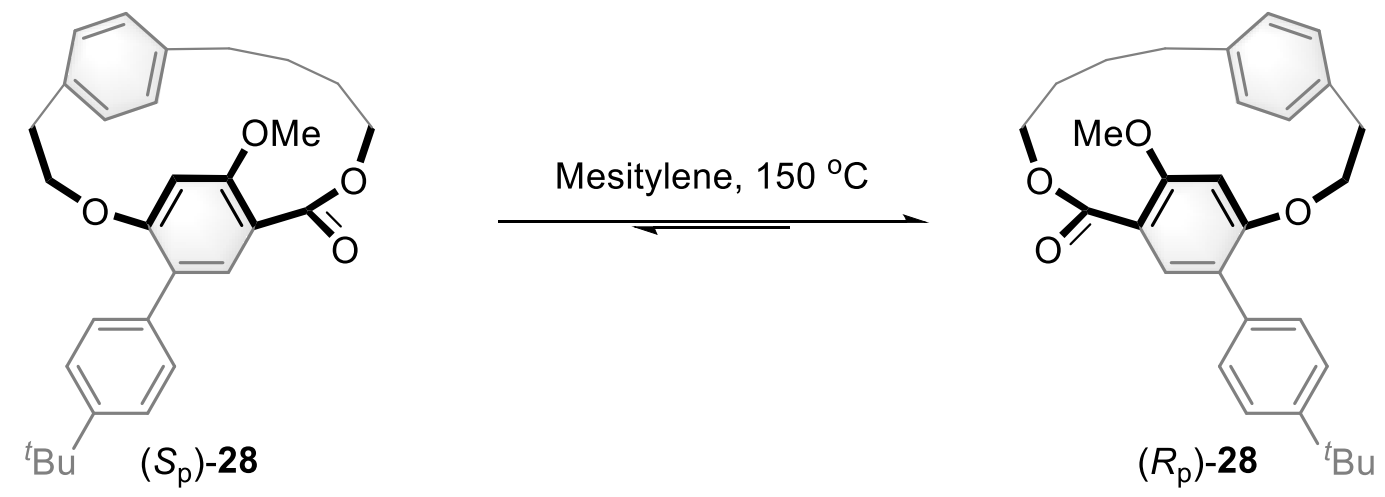

Compound (S_p)-**28** (4 mg, 0.01 mmol, 99.0% *ee*) was dissolved in mesitylene (1.0 mL) in a sealed tube. The reaction mixture was allowed to stir at 150 °C (pre-heated metal module) under air. Within the same time interval, small sample (30 μL) was removed *via* a syringe and diluted by isopropanol, then sample was injected into the HPLC to measure the enantiomeric excess.

Table S7. The racemization experiment of (S_p)-**28** at 150 °C.

time (s)	ee_t (%)	ee_t/ee_0	$\ln(ee_0/ee_t)$
0	98.96	1.0000	0.0000
21600	98.64	0.9968	0.0032
43200	98.35	0.9938	0.0062
86400	97.65	0.9868	0.0134
129600	97.10	0.9812	0.0190
172800	96.42	0.9743	0.0260

T_1 = 150 °C = 423.15 K, T_2 = 25 °C = 298.15 K

k_{rac}(150 °C) = 1.50×10^{-7} s^{-1}

$\tau_{1/2}$(150 °C) = ln2 / k_{rac}(150 °C) = 4622614 s = 77043.57 min = 1284.06 h = 53 days

k_{ent}(150 °C) = k_{rac}(150 °C) / 2 = 7.50 × 10^{-8} s^{-1}

$\Delta G^{\ddagger}_{ent} = RT_1 \cdot \ln((k_B T_1)/(hk_{ent})) = 162.594\ kJ \cdot mol^{-1} = 38.86\ kcal \cdot mol^{-1}$

$k_{rac}(T_2) = 2 \cdot k_B T_2 \cdot h^{-1} \cdot e\hat{}(-\Delta G^{\ddagger}_{ent} /(RT_2))$

$k_{rac}(25\ °C) = 4.07 \times 10^{-16}\ s^{-1}$

$\tau_{1/2}(25\ °C) = \ln 2 / k_{rac}(25\ °C) = 1.70 \times 10^{15}\ s = 4.73\times 10^{11}\ h = 1.97\times 10^{10}$ days

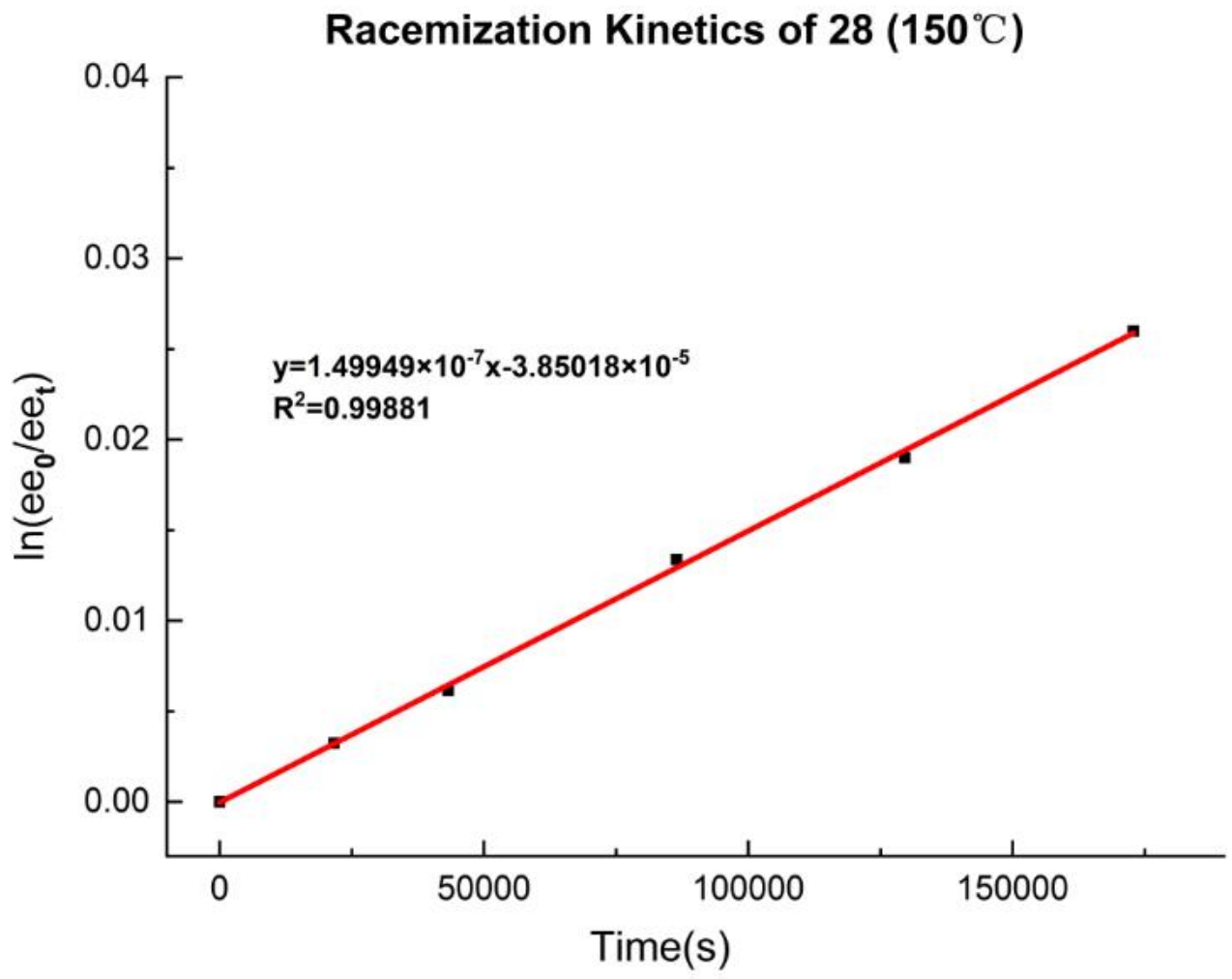

Scheme S5. The racemization curves of (S_p)-**28** at 150 °C.

5. Characterization data

1) Characterization data of substrates 1, S-4–S-32

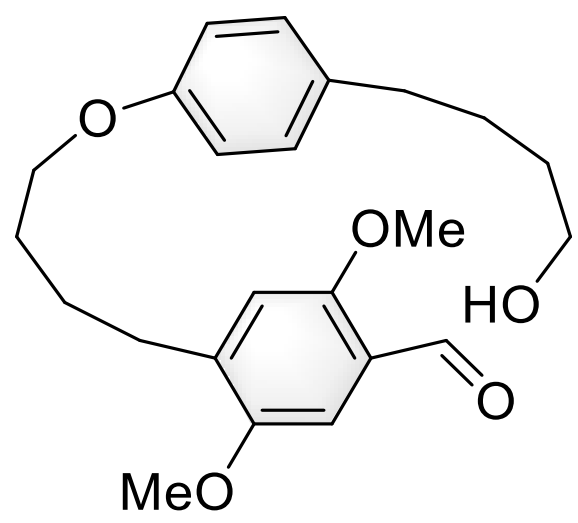

1, yellow solid. **^{1}H NMR** (600 MHz, $CDCl_3$) δ 10.40 (s, 1H), 7.27 (s, 1H), 7.08 (d, J = 8.4 Hz, 2H), 6.81 (d, J = 7.6 Hz, 3H), 3.97 (t, J = 6.0 Hz, 2H), 3.89 (s, 3H), 3.81 (s, 3H), 3.65 (t, J = 6.4 Hz, 2H), 2.72 (t, J = 7.5 Hz, 2H), 2.58 (t, J = 7.5 Hz, 2H), 1.88 – 1.74 (m, 4H), 1.69 – 1.57 (m, 4H). **^{13}C NMR** (101 MHz, Acetone-d_6) δ 188.5, 158.0, 157.3, 152.3, 140.9, 135.1, 129.8, 123.6, 114.9, 108.2, 68.0, 62.2, 56.4, 55.9, 35.2, 33.1, 31.0, 28.7, 26.6. **HRMS** (ESI): m/z calculated for $C_{23}H_{30}NaO_5^+$ $[M + Na]^+$ 409.1985, found 409.1987.

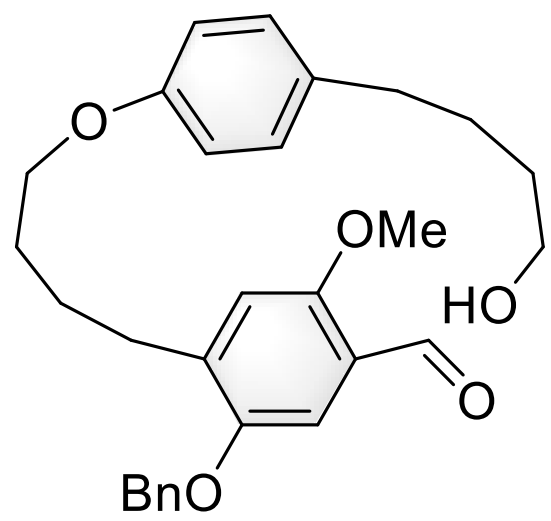

S-4, yellow oil. **^{1}H NMR** (600 MHz, $CDCl_3$) δ 10.41 (s, 1H), 7.44 (d, J = 7.3 Hz, 2H), 7.40 – 7.36 (m, 3H), 7.34 (d, J = 7.1 Hz, 1H), 7.08 (d, J = 8.2 Hz, 2H), 6.86 (s, 1H), 6.79 (d, J = 8.4 Hz, 2H), 5.07 (s, 2H), 3.96 – 3.93 (m, 2H), 3.90 (s, 3H), 3.65 (t, J = 6.4 Hz, 2H), 2.82 – 2.78 (m, 2H), 2.59 (t, J = 7.6 Hz, 2H), 1.86 – 1.82 (m, 4H), 1.69 – 1.64 (m, 2H), 1.63 – 1.58 (m, 2H). **^{13}C NMR** (151 MHz, $CDCl_3$) δ 189.3, 157.2, 156.9, 150.8, 141.0, 137.0, 134.4, 129.3, 128.6, 127.9, 127.3, 123.0, 114.4, 114.0, 109.6, 70.5, 67.7, 62.8, 56.2, 34.8, 32.3, 30.9, 29.1, 27.8, 26.2. **HRMS** (ESI): m/z calculated for $C_{29}H_{34}NaO_5^+$ $[M + Na]^+$ 485.2298, found 485.2294.

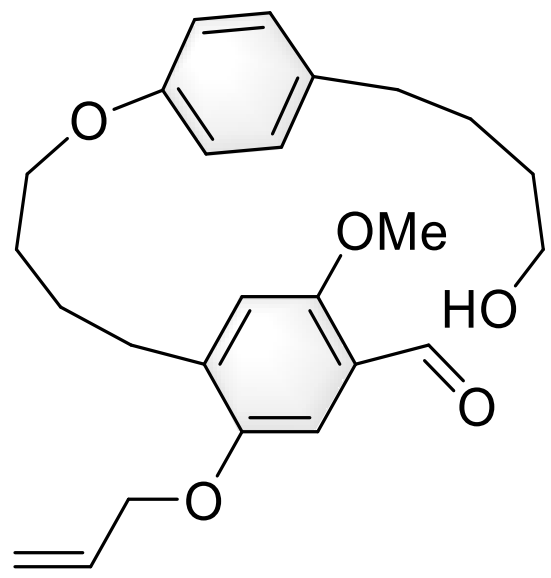

S-5, yellow oil. **^{1}H NMR** (600 MHz, $CDCl_3$) δ 10.39 (s, 1H), 7.27 (s, 1H), 7.08 (d, J = 8.6 Hz, 2H), 6.83 – 6.79 (m, 3H), 6.03 (ddt, J = 17.0, 10.3, 5.0 Hz, 1H), 5.41 (dd, J = 17.2, 1.7 Hz, 1H), 5.25 (dd, J = 10.6, 1.5 Hz, 1H), 4.56 – 4.52 (m, 2H), 3.97 (t, J = 5.8 Hz, 2H), 3.89 (s, 3H), 3.65 (t, J = 6.4 Hz, 2H), 2.76 (t, J = 7.1 Hz, 2H), 2.58 (t, J = 7.4 Hz, 2H), 1.88 – 1.78 (m, 4H), 1.69 – 1.57 (m, 4H). **^{13}C NMR** (151 MHz, $CDCl_3$) δ 189.4, 157.3, 156.9, 150.7, 140.9, 134.5, 133.3, 129.4, 123.1, 117.3, 114.5, 114.0, 109.6, 69.3, 67.8, 63.0, 56.3, 34.8, 32.4, 31.0, 29.2, 27.9, 26.2. **HRMS** (ESI): m/z

calculated for $C_{25}H_{32}NaO_5^+$ [M + Na]$^+$ 435.2142, found 435.2145.

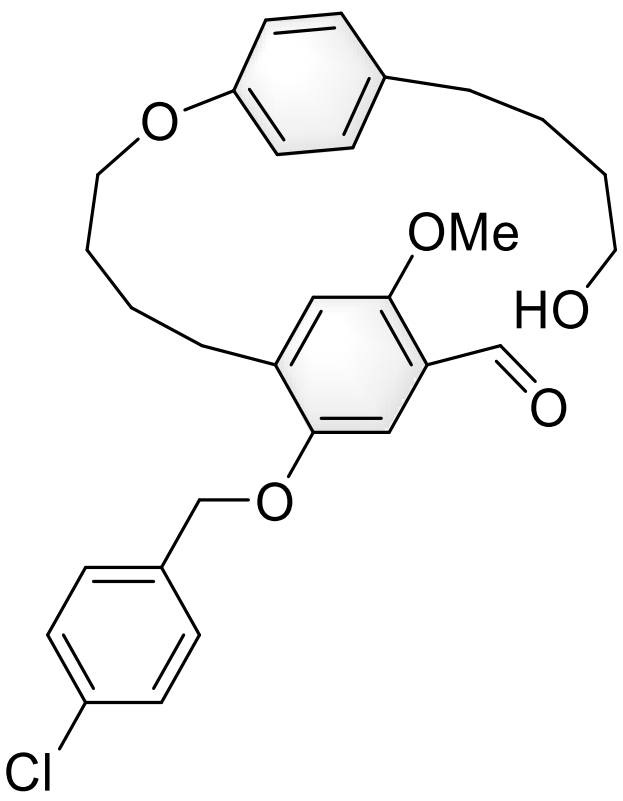

S-6, yellow solid. **^{1}H NMR** (600 MHz, $CDCl_3$) δ 10.39 (s, 1H), 7.34 (d, J = 8.4 Hz, 3H), 7.31 (d, J = 8.5 Hz, 2H), 7.08 (d, J = 8.5 Hz, 2H), 6.85 (s, 1H), 6.77 (d, J = 8.5 Hz, 2H), 5.00 (s, 2H), 3.95 – 3.91 (m, 2H), 3.89 (s, 3H), 3.64 (t, J = 6.4 Hz, 2H), 2.78 (d, J = 7.1 Hz, 2H), 2.58 (t, J = 7.5 Hz, 2H), 1.85 – 1.80 (m, 4H), 1.69 – 1.56 (m, 4H). **^{13}C NMR** (151 MHz, $CDCl_3$) δ 189.2, 157.1, 157.0, 150.5, 140.9, 135.5, 134.5, 133.6, 129.3, 128.7, 128.6, 122.9, 114.3, 114.0, 109.5, 69.6, 67.6, 62.7, 56.1, 34.7, 32.3, 30.8, 29.1, 27.8, 26.2. **HRMS** (ESI): m/z calculated for $C_{29}H_{33}ClNaO_5^+$ [M + Na]$^+$ 519.1909, found 519.1911.

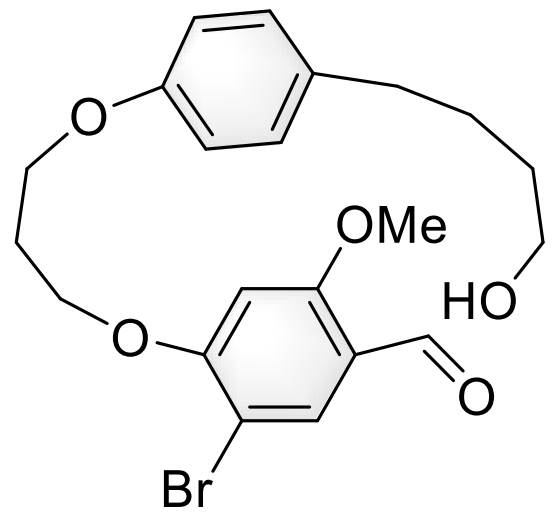

S-7, white solid. **^{1}H NMR** (600 MHz, $CDCl_3$) δ 10.20 (s, 1H), 7.97 (s, 1H), 7.08 (d, J = 8.2 Hz, 2H), 6.83 (d, J = 8.3 Hz, 2H), 6.44 (s, 1H), 4.28 (t, J = 6.0 Hz, 2H), 4.19 (t, J = 5.8 Hz, 2H), 3.89 (s, 3H), 3.63 (t, J = 6.4 Hz, 2H), 2.57 (t, J = 7.5 Hz, 2H), 2.33 (p, J = 5.9 Hz, 2H), 1.68 – 1.55 (m, 6H). **^{13}C NMR** (151 MHz, $CDCl_3$) δ 187.3, 163.0, 161.2, 156.9, 134.9, 132.9, 129.4, 119.4, 114.4, 103.9, 96.5, 66.1, 63.9, 62.8, 56.0, 34.8, 32.3, 29.1, 27.8. **HRMS** (ESI): m/z calculated for $C_{21}H_{25}BrNaO_5^+$ [M + Na]$^+$ 459.0788, found 459.0781.

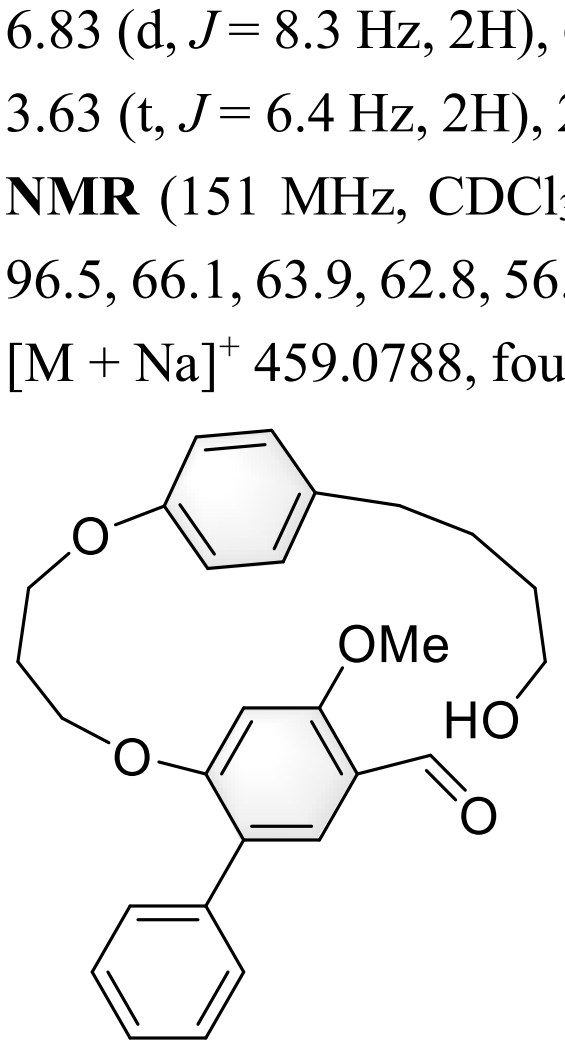

S-8, yellow oil. **^{1}H NMR** (600 MHz, $CDCl_3$) δ 10.34 (s, 1H), 7.83 (s, 1H), 7.46 – 7.44 (m, 2H), 7.37 – 7.33 (m, 2H), 7.32 – 7.28 (m, 1H), 7.08 (d, J = 8.5 Hz, 2H), 6.78 (d, J = 8.6 Hz, 2H), 6.55 (s, 1H), 4.28 (t, J = 6.1 Hz, 2H), 4.05 (t, J = 5.9 Hz, 2H), 3.96 (s, 3H), 3.65 (t, J = 6.4 Hz, 2H), 2.59 (t, J = 7.5 Hz, 2H), 2.22 (p, J = 6.0 Hz, 2H), 1.69 – 1.57 (m, 4H). **^{13}C NMR** (151 MHz, $CDCl_3$) δ 188.4, 163.4, 162.3, 156.9, 137.2, 134.9, 131.3, 129.54, 129.47, 128.1, 127.1, 124.1, 118.6, 114.4, 95.6,

65.4, 64.0, 63.0, 56.0, 34.8, 32.4, 29.1, 27.9. **HRMS** (ESI): m/z calculated for $C_{27}H_{30}NaO_5^+$ [M + Na]$^+$ 457.1985, found 457.1988.

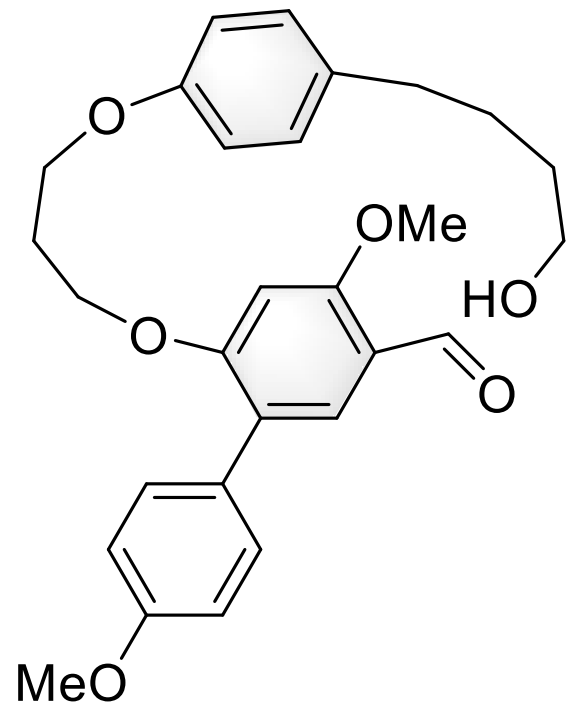

S-9, yellow oil. **^{1}H NMR** (600 MHz, CDCl$_3$) δ 10.33 (s, 1H), 7.79 (s, 1H), 7.38 (d, J = 8.6 Hz, 2H), 7.08 (d, J = 8.3 Hz, 2H), 6.88 (d, J = 8.6 Hz, 2H), 6.78 (d, J = 8.5 Hz, 2H), 6.54 (s, 1H), 4.27 (t, J = 6.1 Hz, 2H), 4.05 (t, J = 5.9 Hz, 2H), 3.95 (s, 3H), 3.83 (s, 3H), 3.65 (t, J = 6.4 Hz, 2H), 2.58 (t, J = 7.5 Hz, 2H), 2.23 (p, J = 6.0 Hz, 2H), 1.69 – 1.56 (m, 4H). **^{13}C NMR** (151 MHz, CDCl$_3$) δ 188.5, 163.1, 162.3, 158.8, 157.0, 134.9, 130.9, 130.6, 129.55, 129.46, 123.9, 118.6, 114.5, 113.6, 95.7, 65.4, 64.1, 62.9, 56.0, 55.4, 34.8, 32.4, 29.1, 27.9. **HRMS** (ESI): m/z calculated for $C_{28}H_{33}O_6^+$ [M + H]$^+$ 465.2272, found 465.2275.

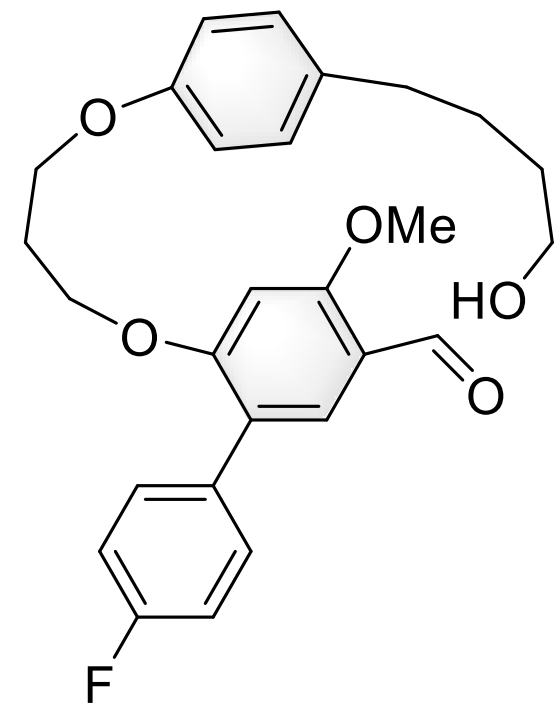

S-10, yellow oil. **^{1}H NMR** (600 MHz, CDCl$_3$) δ 10.33 (s, 1H), 7.78 (s, 1H), 7.41 – 7.38 (m, 2H), 7.08 (d, J = 8.5 Hz, 2H), 7.02 (t, J = 8.7 Hz, 2H), 6.77 (d, J = 8.6 Hz, 2H), 6.55 (s, 1H), 4.28 (t, J = 6.1 Hz, 2H), 4.03 (t, J = 5.9 Hz, 2H), 3.96 (s, 3H), 3.65 (t, J = 6.4 Hz, 2H), 2.58 (t, J = 7.5 Hz, 2H), 2.22 (p, J = 6.0 Hz, 2H), 1.68 – 1.57 (m, 4H). **^{13}C NMR** (151 MHz, CDCl$_3$) δ 188.3, 163.4, 162.2, 162.0 (d, J = 245.8 Hz), 156.8, 134.8, 133.0 (d, J = 3.2 Hz), 131.0 (d, J = 7.9 Hz), 130.8, 129.4, 123.0, 118.4, 114.8 (d, J = 21.3 Hz), 114.3, 95.6, 65.3, 63.9, 62.7, 55.8, 34.7, 32.2, 28.9, 27.8. **^{19}F NMR** (376 MHz, CDCl$_3$) δ -115.52. **HRMS** (ESI): m/z calculated for $C_{27}H_{30}FO_5^+$ [M + H]$^+$ 453.2072, found 453.2070.

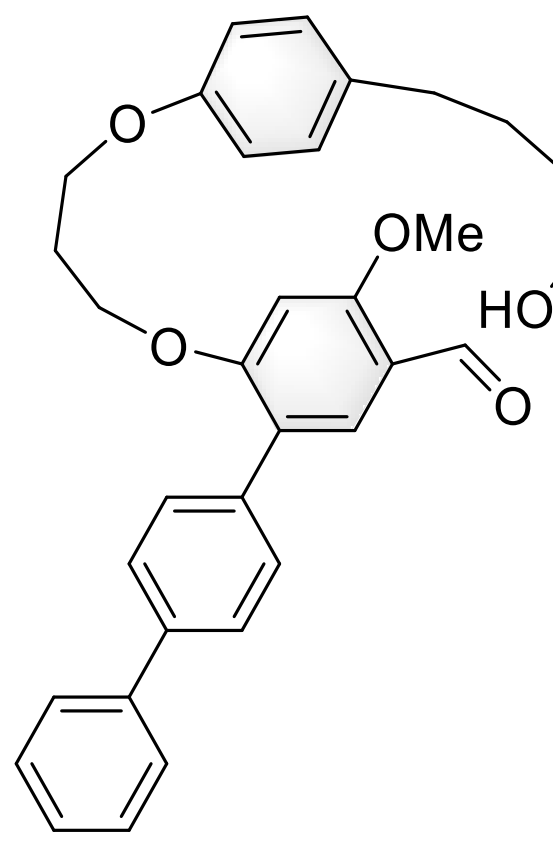

S-11, yellow solid. **^{1}H NMR** (400 MHz, $CDCl_3$) δ 10.37 (s, 1H), 7.91 (s, 1H), 7.65 (d, J = 7.3 Hz, 2H), 7.63 – 7.54 (m, 4H), 7.47 (t, J = 7.6 Hz, 2H), 7.37 (t, J = 7.3 Hz, 1H), 7.07 (d, J = 8.4 Hz, 2H), 6.80 (d, J = 8.5 Hz, 2H), 6.58 (s, 1H), 4.31 (t, J = 6.1 Hz, 2H), 4.08 (t, J = 5.9 Hz, 2H), 3.96 (s, 3H), 3.64 (t, J = 6.2 Hz, 2H), 2.57 (t, J = 7.3 Hz, 2H), 2.25 (p, J = 6.0 Hz, 2H), 1.68 – 1.56 (m, 4H). **^{13}C NMR** (101 MHz, $CDCl_3$) δ 188.4, 163.3, 162.4, 156.8, 140.8, 139.7, 136.1, 134.8, 131.0, 129.8, 129.4, 128.8, 127.3, 127.1, 126.7, 123.6, 118.6, 114.3, 95.7, 65.4, 64.0, 62.7, 55.9, 34.7, 32.3, 29.0, 27.8. **HRMS** (ESI): m/z calculated for $C_{33}H_{35}O_5^+$ $[M + H]^+$ 511.2479, found 511.2475.

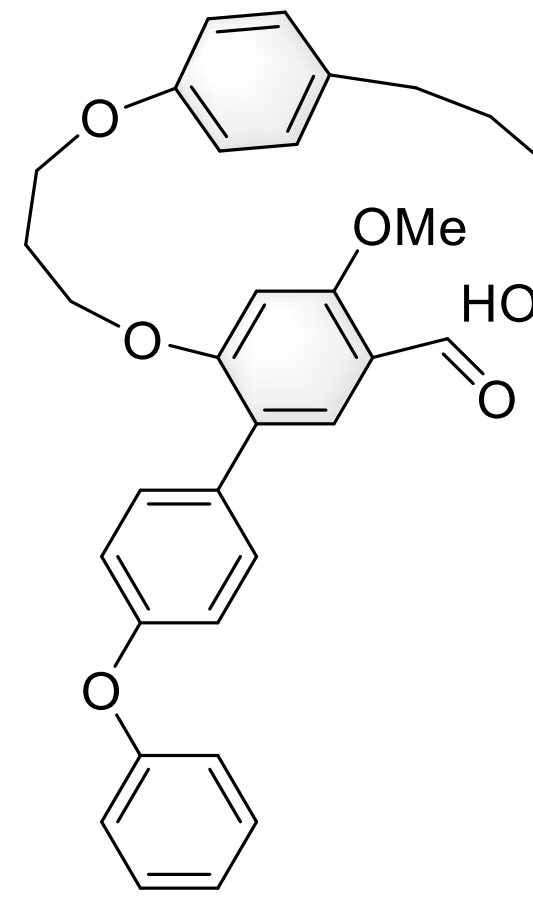

S-12, yellow oil. **^{1}H NMR** (600 MHz, $CDCl_3$) δ 10.34 (s, 1H), 7.82 (s, 1H), 7.41 (d, J = 7.2 Hz, 2H), 7.37 – 7.33 (m, 2H), 7.12 (t, J = 7.5 Hz, 1H), 7.07 – 7.03 (m, 4H), 6.98 (d, J = 7.1 Hz, 2H), 6.77 (d, J = 8.1 Hz, 2H), 6.55 (s, 1H), 4.28 (t, J = 6.0 Hz, 2H), 4.05 (t, J = 5.8 Hz, 2H), 3.95 (s, 3H), 3.63 (d, J = 5.6 Hz, 2H), 2.56 (t, J = 7.6 Hz, 2H), 2.24 – 2.22 (m, 2H), 1.67 – 1.54 (m, 4H). **^{13}C NMR** (151 MHz, $CDCl_3$) δ 188.4, 163.3, 162.3, 157.2, 156.9, 156.4, 134.9, 132.1, 130.9, 130.8, 129.9, 129.4, 123.5, 123.4, 119.1, 118.6, 118.3, 114.4, 95.7, 65.4, 64.0, 62.8, 55.9, 34.8, 32.3, 29.1, 27.8. **HRMS** (ESI): m/z calculated for $C_{33}H_{35}O_6^+$ $[M + H]^+$ 527.2428, found 527.2432.

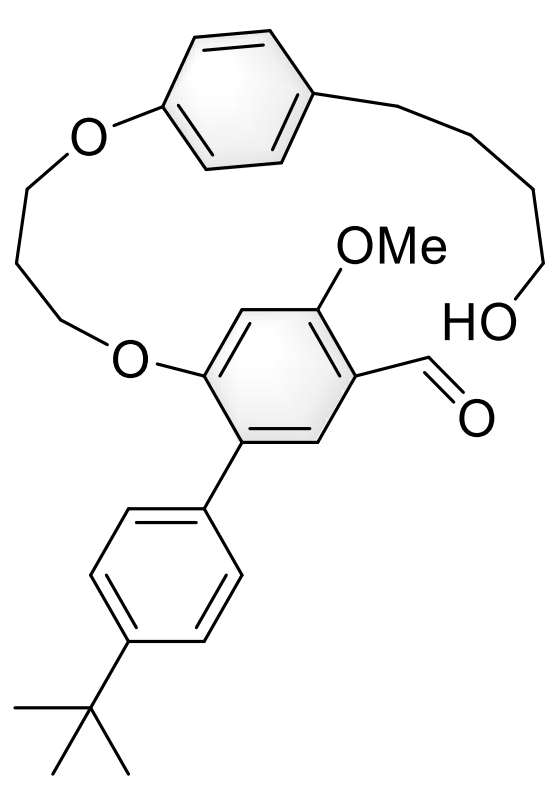

S-13, yellow oil. **^{1}H NMR** (600 MHz, $CDCl_3$) δ 10.34 (s, 1H), 7.86 (s, 1H), 7.43 (d, J = 8.4 Hz, 2H), 7.38 (d, J = 8.4 Hz, 2H), 7.10 (d, J = 8.5 Hz, 2H), 6.81 (d, J = 8.5 Hz, 2H), 6.56 (s, 1H), 4.29 (t, J = 6.2 Hz, 2H), 4.08 (t, J = 6.0 Hz, 2H), 3.95 (s, 3H), 3.65 (t, J = 6.4 Hz, 2H), 2.60 (t, J = 7.5 Hz, 2H), 2.26 (p, J = 6.0 Hz, 2H), 1.70 – 1.57 (m, 4H), 1.36 (s, 9H). **^{13}C NMR** (151 MHz, $CDCl_3$) δ 188.4, 163.1, 162.4, 156.9, 149.9, 134.8, 134.0, 131.1, 129.4, 129.0, 125.0, 123.8, 118.5, 114.4, 95.6, 65.3, 64.1, 62.8, 55.8, 34.8, 34.6, 32.3, 31.4, 29.0, 27.9. **HRMS** (ESI): m/z calculated for $C_{31}H_{39}O_5^+$ $[M+H]^+$ 491.2792, found 491.2790.

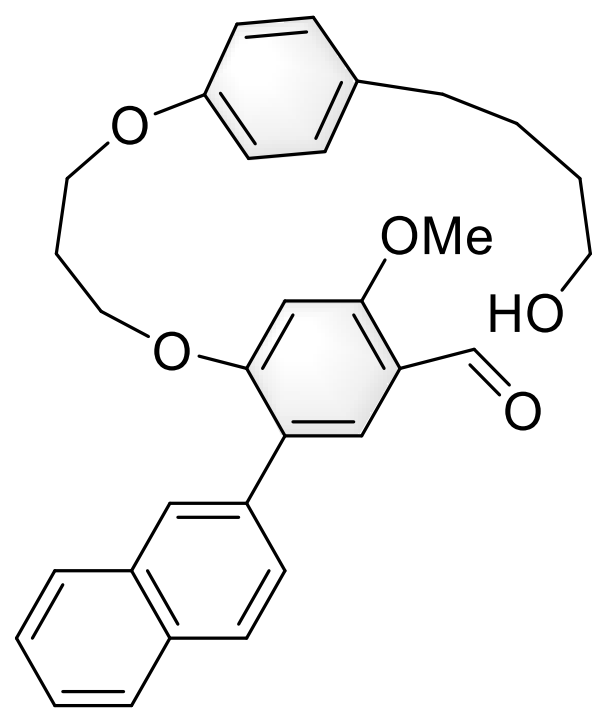

S-14, yellow oil. **^{1}H NMR** (600 MHz, $CDCl_3$) δ 10.38 (s, 1H), 7.96 (s, 1H), 7.92 (s, 1H), 7.88 – 7.84 (m, 1H), 7.81 (dd, J = 8.9, 3.3 Hz, 2H), 7.62 (dd, J = 8.5, 1.7 Hz, 1H), 7.52 – 7.46 (m, 2H), 7.05 (d, J = 8.3 Hz, 2H), 6.76 (d, J = 8.5 Hz, 2H), 6.59 (s, 1H), 4.30 (t, J = 6.0 Hz, 2H), 4.03 (t, J = 5.9 Hz, 2H), 3.97 (s, 3H), 3.65 (t, J = 6.4 Hz, 2H), 2.58 (t, J = 7.5 Hz, 2H), 2.20 (p, J = 6.0 Hz, 2H), 1.70 – 1.57 (m, 4H). **^{13}C NMR** (151 MHz, $CDCl_3$) δ 188.4, 163.5, 162.5, 156.8, 134.80, 134.78, 133.5, 132.5, 131.3, 129.4, 128.2, 127.9, 127.6, 127.3, 126.1, 125.9, 124.1, 118.6, 114.4, 95.7, 65.4, 64.0, 62.8, 55.9, 34.8, 32.3, 29.0, 27.8. **HRMS** (ESI): m/z calculated for $C_{31}H_{32}NaO_5^+$ $[M+Na]^+$ 507.2142, found 507.2140.

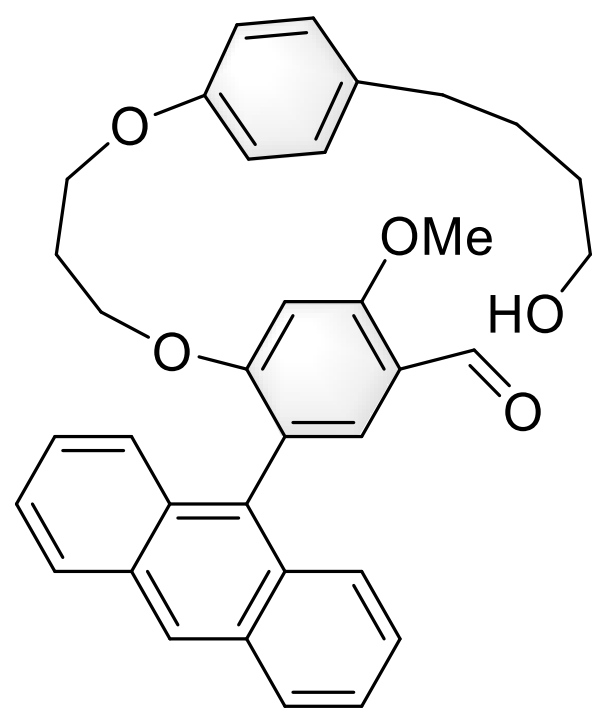

S-15, yellow solid. **^{1}H NMR** (600 MHz, $CDCl_3$) δ 10.43 (s, 1H), 8.48 (s, 1H), 8.03 (d, J = 8.5 Hz,

2H), 7.83 (s, 1H), 7.57 (d, *J* = 8.8 Hz, 2H), 7.43 – 7.40 (m, 2H), 7.26 – 7.23 (m, 2H), 6.99 (d, *J* = 8.6 Hz, 2H), 6.71 (s, 1H), 6.40 (d, *J* = 8.5 Hz, 2H), 4.13 (t, *J* = 5.6 Hz, 2H), 4.06 (s, 3H), 3.67 (t, *J* = 6.4 Hz, 2H), 3.27 (t, *J* = 6.1 Hz, 2H), 2.58 (t, *J* = 7.5 Hz, 2H), 1.76 (p, *J* = 6.0 Hz, 2H), 1.70 – 1.59 (m, 4H). **^{13}C NMR** (151 MHz, $CDCl_3$) δ 188.4, 163.9, 163.7, 156.7, 134.5, 133.5, 132.0, 131.5, 130.7, 129.2, 129.1, 128.6, 127.0, 126.4, 125.6, 125.2, 120.4, 118.7, 114.3, 95.7, 65.2, 63.5, 62.9, 56.0, 34.8, 32.4, 28.7, 27.9. **HRMS** (ESI): m/z calculated for $C_{35}H_{34}NaO_5^+$ [M + Na]$^+$ 557.2298, found 557.2294.

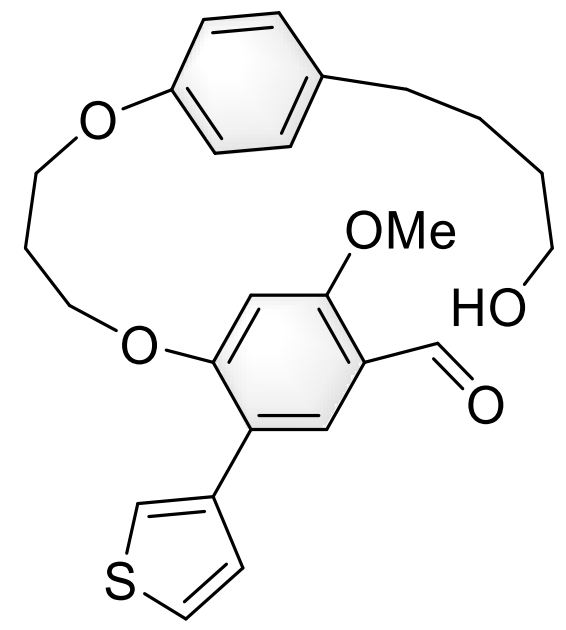

S-16, yellow oil. **^{1}H NMR** (600 MHz, $CDCl_3$) δ 10.32 (s, 1H), 7.98 (s, 1H), 7.53 (dd, *J* = 3.0, 1.3 Hz, 1H), 7.39 (dd, *J* = 5.1, 1.3 Hz, 1H), 7.30 (dd, *J* = 5.1, 3.0 Hz, 1H), 7.09 (d, *J* = 8.3 Hz, 2H), 6.81 (d, *J* = 8.4 Hz, 2H), 6.52 (s, 1H), 4.31 (t, *J* = 6.1 Hz, 2H), 4.11 (t, *J* = 5.8 Hz, 2H), 3.93 (s, 3H), 3.64 (t, *J* = 6.4 Hz, 2H), 2.58 (t, *J* = 7.5 Hz, 2H), 2.31 (p, *J* = 6.0 Hz, 2H), 1.69 – 1.55 (m, 4H). **^{13}C NMR** (151 MHz, $CDCl_3$) δ 188.3, 163.0, 162.2, 156.8, 136.9, 134.9, 129.9, 129.4, 128.3, 124.6, 122.8, 118.7, 118.4, 114.4, 95.7, 65.7, 64.2, 62.8, 55.9, 34.8, 32.3, 29.2, 27.8. **HRMS** (ESI): m/z calculated for $C_{25}H_{28}NaO_5S^+$ [M + Na]$^+$ 463.1550, found 463.1555.

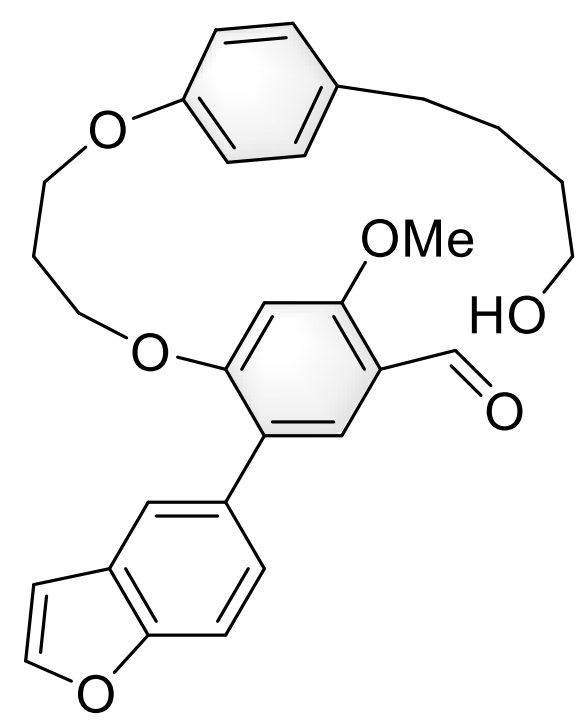

S-17, yellow oil, 70% yield, 319 mg. **^{1}H NMR** (600 MHz, $CDCl_3$) δ 10.36 (s, 1H), 7.86 (s, 1H), 7.64 (dd, *J* = 15.0, 2.0 Hz, 2H), 7.46 (d, *J* = 8.5 Hz, 1H), 7.38 (dd, *J* = 8.5, 1.8 Hz, 1H), 7.06 (d, *J* = 8.3 Hz, 2H), 6.76 (d, *J* = 8.5 Hz, 2H), 6.71 (d, *J* = 1.9 Hz, 1H), 6.56 (s, 1H), 4.27 (t, *J* = 6.0 Hz, 2H), 4.01 (t, *J* = 5.9 Hz, 2H), 3.94 (s, 3H), 3.64 (t, *J* = 6.5 Hz, 2H), 2.58 (t, *J* = 7.5 Hz, 2H), 2.18 (p, *J* = 5.8 Hz, 2H), 1.69 – 1.56 (m, 4H). **^{13}C NMR** (151 MHz, $CDCl_3$) δ 188.3, 163.1, 162.3, 156.8, 154.1, 145.2, 134.7, 131.8, 131.1, 129.3, 127.3, 126.1, 124.3, 121.9, 118.4, 114.2, 110.6, 106.7, 95.6, 65.2, 63.9, 62.6, 55.8, 34.7, 32.2, 28.8, 27.7. **HRMS** (ESI): m/z calculated for $C_{29}H_{30}NaO_6^+$ [M + Na]$^+$ 497.1935, found 497.1933.

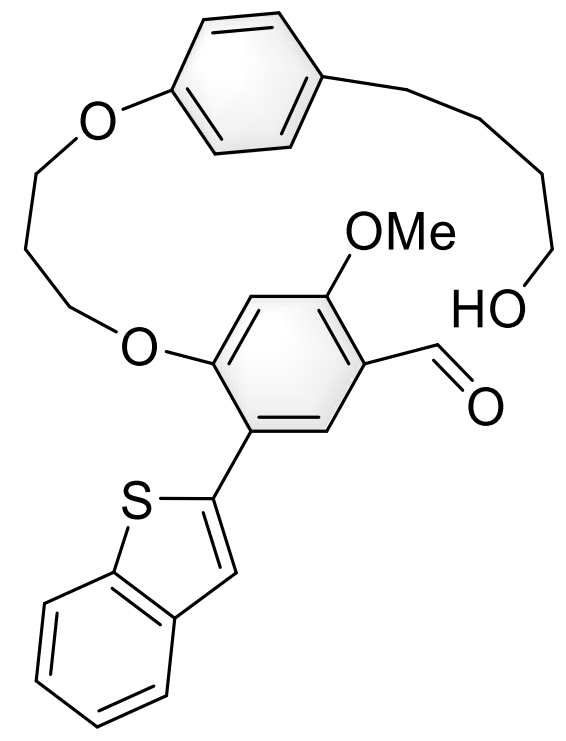

S-18, yellow oil. **^{1}H NMR** (600 MHz, $CDCl_3$) δ 10.33 (s, 1H), 8.19 (s, 1H), 7.80 (d, J = 7.9 Hz, 1H), 7.73 (d, J = 7.7 Hz, 1H), 7.64 (s, 1H), 7.36 – 7.28 (m, 2H), 7.07 (d, J = 8.5 Hz, 2H), 6.83 (d, J = 8.5 Hz, 2H), 6.51 (s, 1H), 4.36 (t, J = 6.1 Hz, 2H), 4.21 (t, J = 5.8 Hz, 2H), 3.92 (s, 3H), 3.64 (t, J = 6.4 Hz, 2H), 2.57 (t, J = 7.5 Hz, 2H), 2.40 (p, J = 6.0 Hz, 2H), 1.68 – 1.55 (m, 4H). **^{13}C NMR** (151 MHz, $CDCl_3$) δ 188.1, 163.4, 161.7, 156.9, 139.9, 139.8, 138.7, 134.9, 129.9, 129.4, 124.3, 124.1, 123.4, 121.9, 121.8, 118.6, 116.8, 114.4, 95.6, 66.2, 64.3, 62.8, 55.9, 34.8, 32.3, 29.2, 27.8. **HRMS** (ESI): m/z calculated for $C_{29}H_{30}NaO_5S^+$ $[M + Na]^+$ 513.1706, found 513.1709.

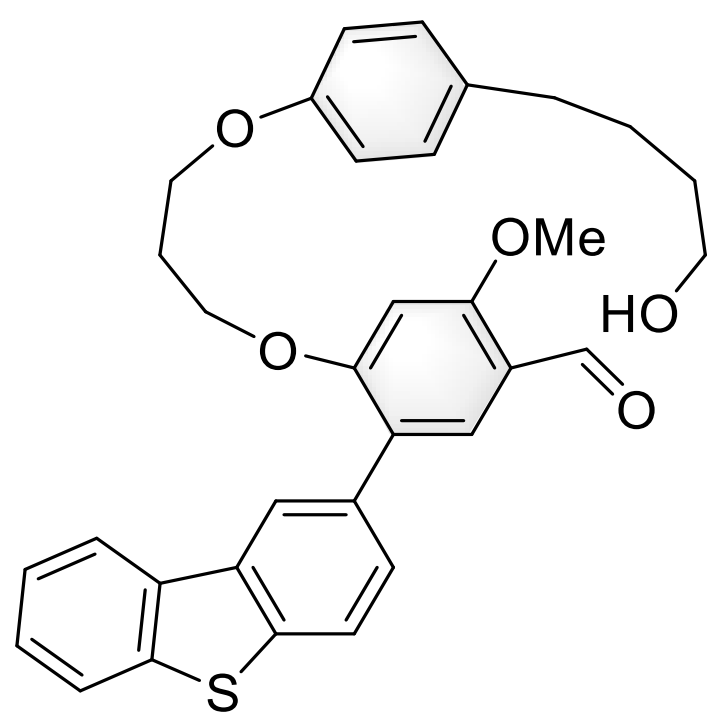

S-19, yellow solid. **^{1}H NMR** (600 MHz, $CDCl_3$) δ 10.37 (s, 1H), 8.23 (d, J = 1.7 Hz, 1H), 8.11 (d, J = 7.7 Hz, 1H), 7.94 (s, 1H), 7.86 (d, J = 7.8 Hz, 1H), 7.80 (d, J = 8.2 Hz, 1H), 7.55 (dd, J = 8.3, 1.7 Hz, 1H), 7.48 – 7.40 (m, 2H), 7.01 (d, J = 8.3 Hz, 2H), 6.72 (d, J = 8.6 Hz, 2H), 6.58 (s, 1H), 4.30 (t, J = 6.0 Hz, 2H), 4.02 (t, J = 5.9 Hz, 2H), 3.97 (s, 3H), 3.64 (t, J = 6.3 Hz, 2H), 2.55 (t, J = 7.4 Hz, 2H), 2.21 – 2.17 (m, 2H), 1.67 – 1.55 (m, 4H). **^{13}C NMR** (151 MHz, $CDCl_3$) δ 188.5, 163.5, 162.5, 156.8, 139.8, 138.2, 135.7, 135.6, 134.9, 133.6, 131.3, 129.4, 128.5, 126.8, 124.5, 124.0, 122.9, 122.4, 122.2, 121.7, 118.7, 114.4, 95.7, 65.4, 64.0, 62.8, 56.0, 34.8, 32.3, 29.3, 27.8. **HRMS** (ESI): m/z calculated for $C_{33}H_{32}NaO_5S^+$ $[M + Na]^+$ 563.1863, found 563.1866.

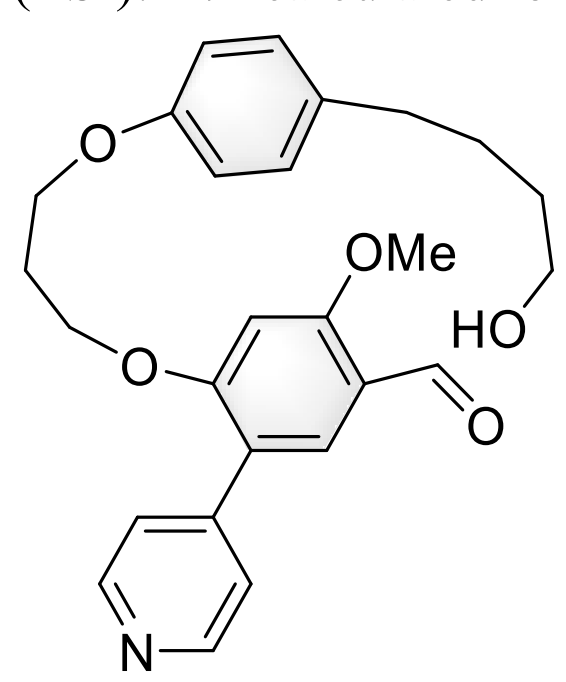

S-20, yellow oil. **^{1}H NMR** (600 MHz, $CDCl_3$) δ 10.33 (s, 1H), 8.49 (d, J = 6.2 Hz, 2H), 7.85 (s, 1H), 7.41 – 7.36 (m, 2H), 7.08 (d, J = 8.4 Hz, 2H), 6.77 (d, J = 8.5 Hz, 2H), 6.57 (s, 1H), 4.32 (t, J = 6.1 Hz, 2H), 4.04 (t, J = 5.8 Hz, 2H), 3.97 (s, 3H), 3.64 (t, J = 6.5 Hz, 2H), 2.58 (t, J = 7.4 Hz,

2H), 2.24 (p, J = 6.0 Hz, 2H), 1.69 – 1.54 (m, 4H). **^{13}C NMR** (151 MHz, $CDCl_3$) δ 188.0, 164.2, 162.3, 156.9, 149.4, 145.2, 135.0, 131.0, 129.6, 124.2, 121.0, 118.8, 114.4, 95.8, 65.5, 63.9, 62.7, 56.0, 34.7, 32.3, 29.0, 27.8. **HRMS** (ESI): m/z calculated for $C_{26}H_{29}NaNO_5^+$ $[M + Na]^+$ 458.1938, found 458.1936.

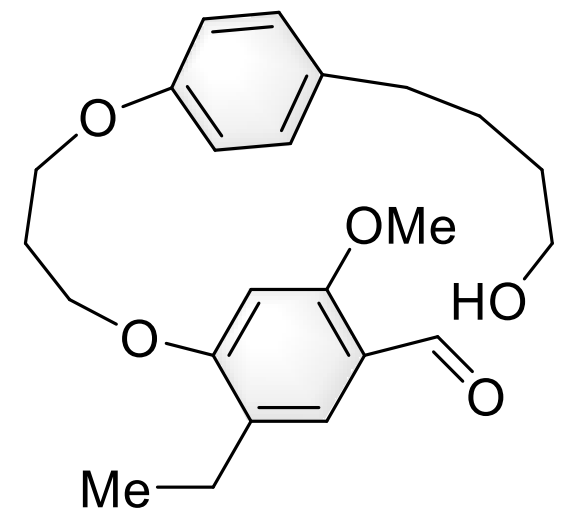

S-21, yellow oil, 70% yield, 319 mg. **^{1}H NMR** (600 MHz, $CDCl_3$) δ 10.27 (s, 1H), 7.62 (s, 1H), 7.07 (d, J = 2.3 Hz, 2H), 6.83 (d, J = 2.5 Hz, 2H), 6.40 (s, 1H), 4.23 (t, J = 6.1 Hz, 2H), 4.15 (t, J = 6.0 Hz, 2H), 3.87 (s, 3H), 3.63 (t, J = 6.5 Hz, 2H), 2.59 – 2.53 (m, 4H), 2.30 (p, J = 6.1 Hz, 2H), 1.68 – 1.55 (m, 4H), 1.15 (t, J = 7.5 Hz, 3H). **^{13}C NMR** (101 MHz, $CDCl_3$) δ 188.6, 163.3, 162.7, 156.9, 134.9, 130.8, 129.4, 125.5, 114.4, 106.5, 98.4, 64.9, 64.2, 62.9, 55.8, 34.8, 32.4, 29.3, 27.9, 22.5, 14.0. **HRMS** (ESI): m/z calculated for $C_{23}H_{31}O_5^+$ $[M + H]^+$ 387.2166, found 387.2169.

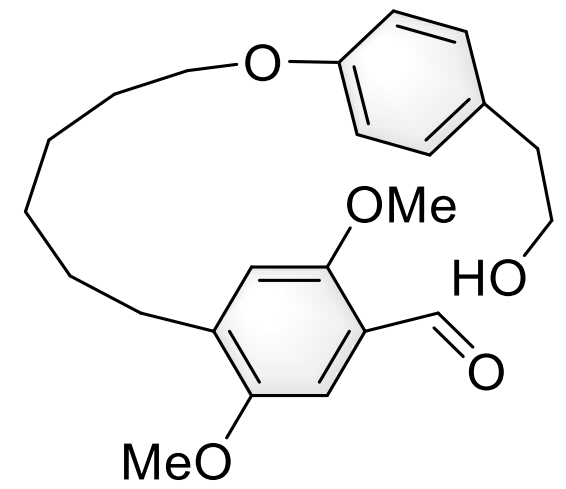

S-22, yellow solid. **^{1}H NMR** (400 MHz, $CDCl_3$) δ 10.39 (s, 1H), 7.27 (s, 1H), 7.13 (d, J = 8.5 Hz, 2H), 6.84 (d, J = 8.6 Hz, 2H), 6.79 (s, 1H), 3.93 (t, J = 6.5 Hz, 2H), 3.88 (s, 3H), 3.85 – 3.80 (m, 5H), 2.81 (t, J = 6.5 Hz, 2H), 2.70 – 2.62 (m, 2H), 1.78 (p, J = 6.7 Hz, 2H), 1.62 (p, J = 7.5 Hz, 2H), 1.55 – 1.40 (m, 4H). **^{13}C NMR** (101 MHz, $CDCl_3$) δ 189.4, 158.0, 156.9, 151.9, 141.1, 130.4, 130.1, 123.0, 114.8, 114.0, 108.3, 68.1, 64.0, 56.3, 55.9, 38.4, 31.2, 29.6, 29.40, 29.36, 26.0. **HRMS** (ESI): m/z calculated for $C_{23}H_{31}O_5^+$ $[M + H]^+$ 387.2166, found 387.2163.

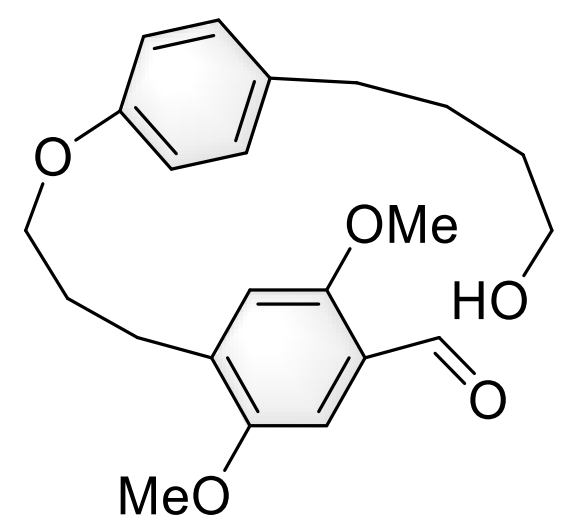

S-23, yellow oil. **^{1}H NMR** (400 MHz, $CDCl_3$) δ 10.38 (s, 1H), 7.27 (s, 1H), 7.08 (d, J = 8.8 Hz, 2H), 6.87 – 6.78 (m, 3H), 3.93 (t, J = 6.1 Hz, 2H), 3.80 (s, 3H), 3.77 (s, 3H), 3.65 (t, J = 6.3 Hz, 2H), 2.85 (t, J = 7.5 Hz, 2H), 2.63 – 2.55 (m, 2H), 2.11 – 2.05 (m, 2H), 1.70 – 1.58 (m, 4H). **^{13}C NMR** (101 MHz, $CDCl_3$) δ 189.4, 157.3, 156.8, 151.9, 139.7, 134.6, 129.4, 123.2, 114.5, 114.4, 108.2, 67.0, 62.9, 56.2, 55.8, 34.8, 32.4, 28.9, 27.9, 27.8. **HRMS** (ESI): m/z calculated for $C_{22}H_{29}O_5^+$ $[M + H]^+$ 373.2010, found 373.2013.

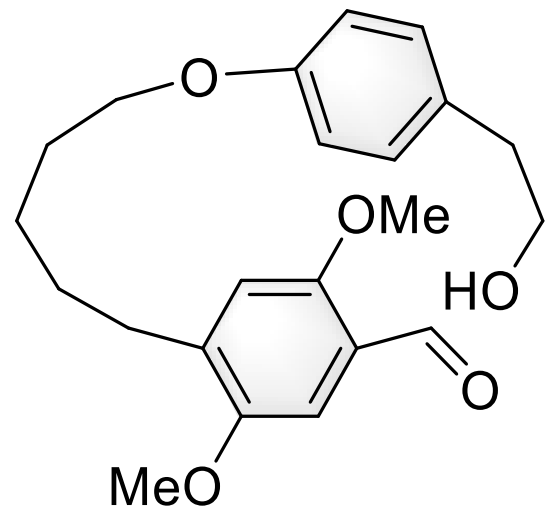

S-24, yellow solid. **^{1}H NMR** (600 MHz, $CDCl_3$) δ 10.40 (s, 1H), 7.27 (s, 1H), 7.13 (d, J = 8.6 Hz, 2H), 6.84 (d, J = 8.6 Hz, 2H), 6.79 (s, 1H), 3.95 (t, J = 6.4 Hz, 2H), 3.89 (s, 3H), 3.84 – 3.80 (m, 5H), 2.81 (t, J = 6.6 Hz, 2H), 2.71 – 2.64 (m, 2H), 1.85 – 1.79 (m, 2H), 1.70 – 1.62 (m, 2H), 1.57 – 1.50 (m, 2H). **^{13}C NMR** (151 MHz, $CDCl_3$) δ 189.4, 157.9, 156.9, 151.8, 140.9, 130.5, 130.1, 123.0, 114.8, 114.0, 108.3, 67.9, 63.9, 56.3, 55.9, 38.4, 31.1, 29.4, 29.2, 26.1. **HRMS** (ESI): m/z calculated for $C_{22}H_{29}O_5^+$ $[M + H]^+$ 373.2010, found 373.2014.

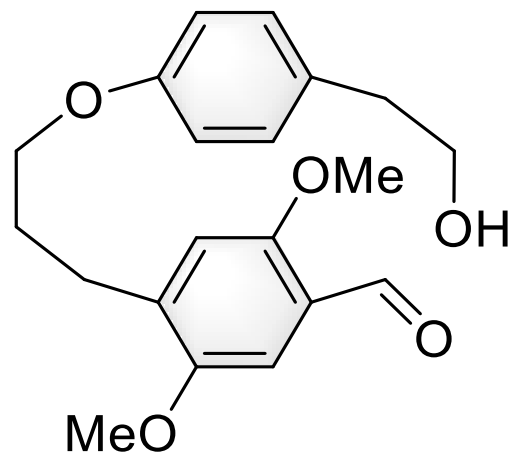

S-25, yellow oil. **^{1}H NMR** (400 MHz, $CDCl_3$) δ 10.38 (s, 1H), 7.27 (s, 1H), 7.13 (d, J = 8.1 Hz, 2H), 6.84 (d, J = 8.5 Hz, 2H), 6.79 (s, 1H), 3.94 (t, J = 6.2 Hz, 2H), 3.84 – 3.79 (m, 5H), 3.78 (s, 3H), 2.83 (dt, J = 16.7, 7.0 Hz, 4H), 2.08 (p, J = 6.6 Hz, 2H). **^{13}C NMR** (101 MHz, $CDCl_3$) δ 189.4, 157.8, 156.7, 151.8, 139.7, 130.6, 130.1, 123.1, 114.7, 114.3, 108.2, 67.1, 63.9, 56.1, 55.8, 38.4, 28.9, 27.8. **HRMS** (ESI): m/z calculated for $C_{20}H_{24}NaNO_5^+$ $[M + Na]^+$ 367.1516, found 367.1519.

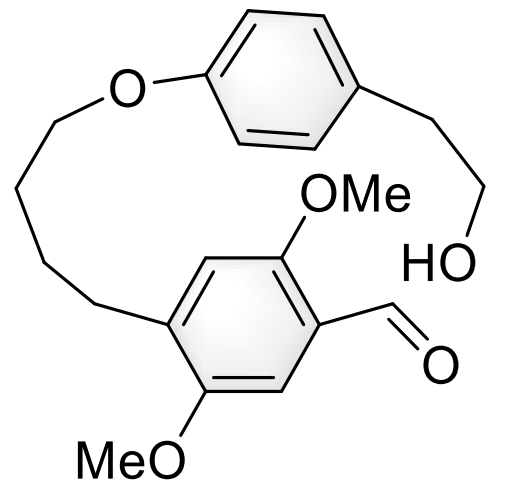

S-26, yellow solid. **^{1}H NMR** (600 MHz, $CDCl_3$) δ 10.39 (s, 1H), 7.27 (s, 1H), 7.12 (d, J = 8.5 Hz, 2H), 6.84 (d, J = 8.5 Hz, 2H), 6.81 (s, 1H), 3.97 (t, J = 6.1 Hz, 2H), 3.88 (s, 3H), 3.83 – 3.80 (m, 5H), 2.80 (t, J = 6.6 Hz, 2H), 2.72 (t, J = 7.5 Hz, 2H), 1.86 – 1.75 (m, 4H). **^{13}C NMR** (151 MHz, $CDCl_3$) δ 189.4, 157.9, 156.8, 151.8, 140.6, 130.5, 130.1, 123.1, 114.8, 114.0, 108.3, 67.8, 63.9, 56.3, 55.9, 38.4, 30.8, 29.2, 26.1. **HRMS** (ESI): m/z calculated for $C_{21}H_{26}NaNO_5^+$ $[M + Na]^+$ 381.1672, found 381.1674.

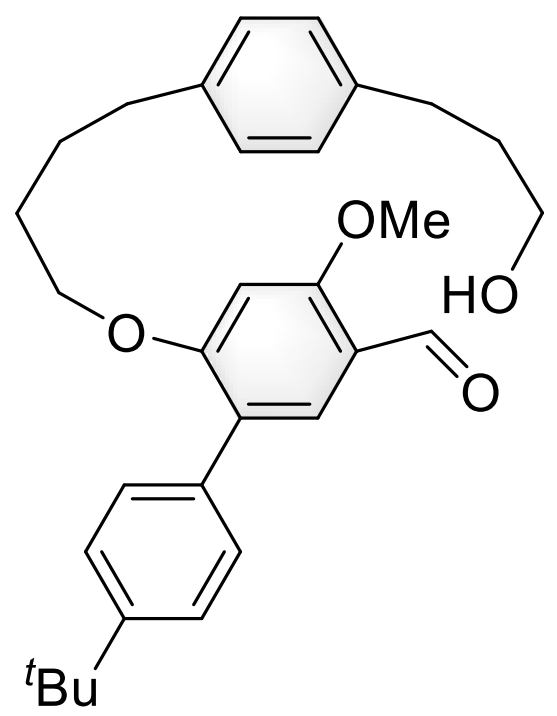

S-27, yellow oil. **^{1}H NMR** (600 MHz, $CDCl_3$) δ 10.33 (s, 1H), 7.86 (s, 1H), 7.45 (d, J = 8.4 Hz, 2H), 7.40 (d, J = 8.4 Hz, 2H), 7.12 (d, J = 8.0 Hz, 2H), 7.07 (d, J = 7.8 Hz, 2H), 6.47 (s, 1H), 4.08 (t, J = 6.1 Hz, 2H), 3.95 (s, 3H), 3.68 (t, J = 6.4 Hz, 2H), 2.71 – 2.66 (m, 2H), 2.62 (t, J = 7.5 Hz, 2H), 1.92 – 1.86 (m, 2H), 1.85 – 1.75 (m, 4H), 1.36 (s, 9H). **^{13}C NMR** (151 MHz, $CDCl_3$) δ 188.4, 163.2, 162.6, 149.9, 139.6, 139.4, 134.1, 131.2, 129.2, 128.52, 128.49, 125.0, 123.9, 118.5, 95.4, 68.6, 62.4, 55.9, 35.1, 34.6, 34.4, 31.8, 31.5, 28.6, 28.0. **HRMS** (ESI): m/z calculated for $C_{31}H_{38}NaNO_4^+$ $[M + Na]^+$ 497.2662, found 497.2659.

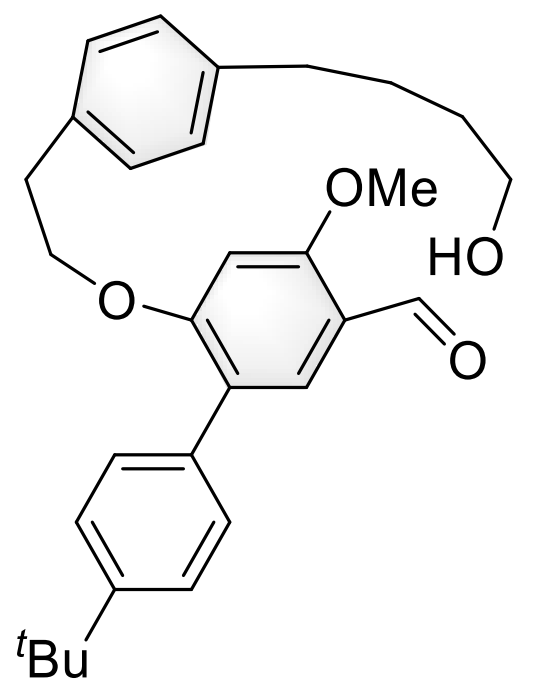

S-28, yellow oil. **^{1}H NMR** (400 MHz, $CDCl_3$) δ 10.31 (s, 1H), 7.83 (s, 1H), 7.41 – 7.33 (m, 4H), 7.11 (s, 4H), 6.46 (s, 1H), 4.25 (t, J = 6.8 Hz, 2H), 3.93 (s, 3H), 3.66 (t, J = 6.4 Hz, 2H), 3.06 (t, J = 6.8 Hz, 2H), 2.63 (t, J = 7.5 Hz, 2H), 1.74 – 1.58 (m, 4H), 1.38 (s, 9H). **^{13}C NMR** (101 MHz, $CDCl_3$) δ 188.4, 163.1, 162.4, 149.9, 140.9, 135.2, 134.0, 131.1, 129.3, 128.6, 125.0, 123.9, 118.5, 95.4, 69.8, 62.8, 55.9, 35.3, 35.2, 34.6, 32.4, 31.5, 27.7. **HRMS** (ESI): m/z calculated for $C_{30}H_{36}NaO_4^+$ $[M + Na]^+$ 483.2506, found 483.2503.

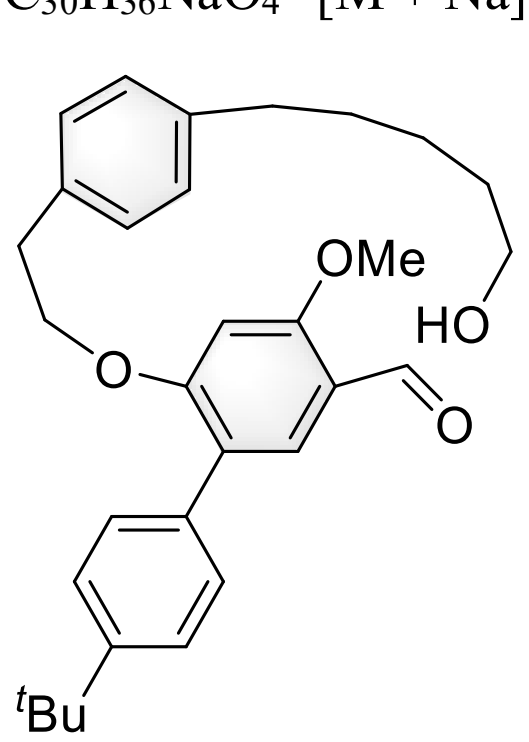

S-29, yellow oil. **^{1}H NMR** (600 MHz, $CDCl_3$) δ 10.32 (s, 1H), 7.83 (s, 1H), 7.40 – 7.34 (m, 4H), 7.10 (s, 4H), 6.46 (s, 1H), 4.26 (t, J = 6.8 Hz, 2H), 3.93 (s, 3H), 3.64 (t, J = 6.6 Hz, 2H), 3.06 (t, J = 6.8 Hz, 2H), 2.61 (t, J = 7.8 Hz, 2H), 1.68 – 1.57 (m, 4H), 1.45 – 1.39 (m, 2H), 1.38 (s, 9H). **^{13}C NMR** (151 MHz, $CDCl_3$) δ 188.4, 163.1, 162.4, 149.9, 141.1, 135.2, 134.0, 131.2, 129.30, 129.26,

128.7, 125.0, 123.9, 118.6, 95.5, 69.9, 63.0, 56.0, 35.6, 35.3, 34.7, 32.8, 31.5, 31.4, 25.6. **HRMS** (ESI): m/z calculated for $C_{31}H_{38}NaO_4^+$ $[M + Na]^+$ 497.2662, found 497.2668.

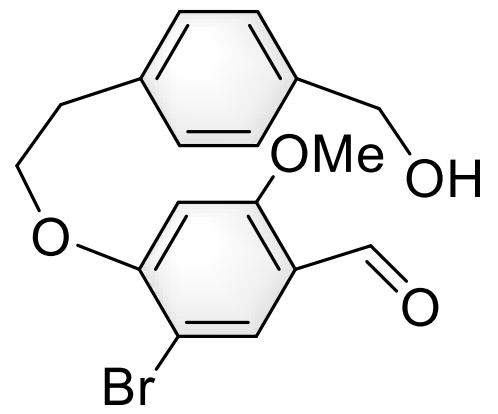

S-30, white solid. **^{1}H NMR** (400 MHz, $CDCl_3$) δ 10.21 (s, 1H), 7.99 (s, 1H), 7.35 (s, 4H), 6.37 (s, 1H), 4.68 (s, 2H), 4.26 (t, J = 6.7 Hz, 2H), 3.88 (s, 3H), 3.20 (t, J = 6.7 Hz, 2H). **^{13}C NMR** (101 MHz, $CDCl_3$) δ 187.3, 163.0, 161.2, 139.7, 137.1, 133.1, 129.7, 127.5, 119.5, 104.0, 96.4, 70.4, 65.2, 56.1, 35.4. **HRMS** (ESI): m/z calculated for $C_{17}H_{17}BrNaO_4^+$ $[M + Na]^+$ 387.0202, found 387.0204.

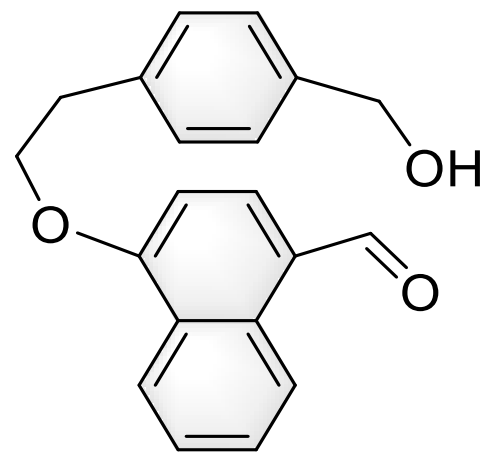

S-31, yellow solid. **^{1}H NMR** (600 MHz, $CDCl_3$) δ 10.16 (s, 1H), 9.28 (d, J = 8.6 Hz, 1H), 8.32 (d, J = 8.4 Hz, 1H), 7.87 (d, J = 8.0 Hz, 1H), 7.71 – 7.66 (m, 1H), 7.58 – 7.55 (m, 1H), 7.36 (s, 4H), 6.87 (d, J = 8.0 Hz, 1H), 4.68 (s, 2H), 4.43 (t, J = 6.7 Hz, 2H), 3.28 (t, J = 6.7 Hz, 2H). **^{13}C NMR** (151 MHz, $CDCl_3$) δ 192.4, 160.1, 139.7, 139.6, 137.4, 132.0, 129.7, 129.3, 127.5, 126.5, 125.6, 125.1, 125.0, 122.5, 103.7, 69.4, 65.2, 35.4. **HRMS** (ESI): m/z calculated for $C_{20}H_{18}NaO_3^+$ $[M + Na]^+$ 329.1148, found 329.1144.

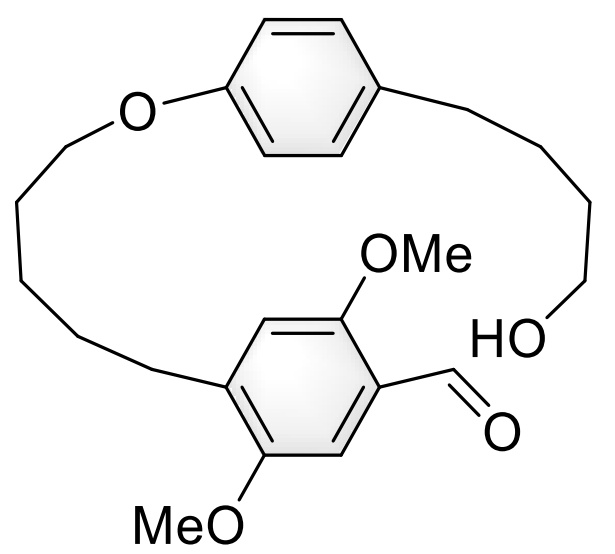

S-32, yellow solid. **^{1}H NMR** (600 MHz, $CDCl_3$) δ 10.39 (s, 1H), 7.26 (s, 1H), 7.07 (d, J = 8.5 Hz, 2H), 6.81 – 6.78 (m, 3H), 3.93 (t, J = 6.5 Hz, 2H), 3.87 (s, 3H), 3.80 (s, 3H), 3.63 (t, J = 6.4 Hz, 2H), 2.67 (t, J = 7.8 Hz, 2H), 2.57 (t, J = 7.5 Hz, 2H), 1.81 (p, J = 6.6 Hz, 2H), 1.69 – 1.62 (m, 4H), 1.60 – 1.50 (m, 4H). **^{13}C NMR** (101 MHz, $CDCl_3$) δ 189.3, 157.2, 156.8, 151.7, 140.9, 134.4, 129.3, 122.8, 114.3, 113.9, 108.1, 67.8, 62.7, 56.2, 55.8, 34.7, 32.3, 31.0, 29.3, 29.2, 27.8, 26.0. **HRMS** (ESI): m/z calculated for $C_{24}H_{32}NaO_5^+$ $[M + Na]^+$ 423.2142, found 423.2145.

2) Characterization data of cyclophanes 2–29, 32–35

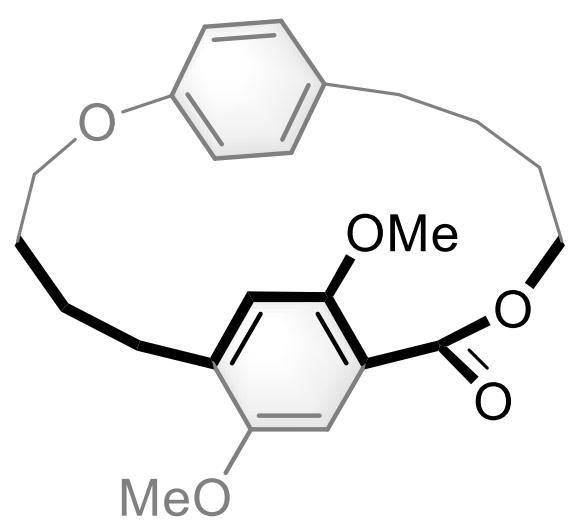

2, yellow oil, 61% yield, 23.4 mg, 94% *ee*. **^{1}H NMR** (600 MHz, $CDCl_3$) δ 6.87 (s, 1H), 6.86 (d, J = 8.3 Hz, 2H), 6.69 (s, 1H), 6.34 (d, J = 8.0 Hz, 2H), 4.47 – 4.41 (m, 1H), 4.06 (dd, J = 10.8, 6.1 Hz, 1H), 3.80 (s, 3H), 3.75 (s, 3H), 3.67 – 3.62 (m, 1H), 3.56 – 3.52 (m, 1H), 3.09 (ddd, J = 13.6, 8.3, 2.7 Hz, 1H), 2.49 – 2.38 (m, 2H), 2.36 – 2.30 (m, 1H), 2.21 – 2.13 (m, 1H), 1.87 – 1.65 (m, 7H). **^{13}C NMR** (151 MHz, $CDCl_3$) δ 165.6, 156.5, 153.8, 150.6, 137.5, 134.8, 129.2, 117.2, 115.3, 114.1, 113.4, 65.8, 64.3, 56.9, 55.7, 34.3, 30.2, 28.5, 28.2, 27.7, 23.4. **HRMS** (ESI): m/z calculated for $C_{23}H_{28}NaO_5^+$ [M + Na]$^+$ 407.1829, found 407.1833. $[\alpha]_D^{25}$ = -32.9 (c = 1.17, CH_2Cl_2). **HPLC**: Chiralpak IF column, 80:20 hexanes/isopropanol, flow rate = 1.0 mL/min, λ = 254 nm, t_R = 9.24 min (minor), 11.16 min (major).

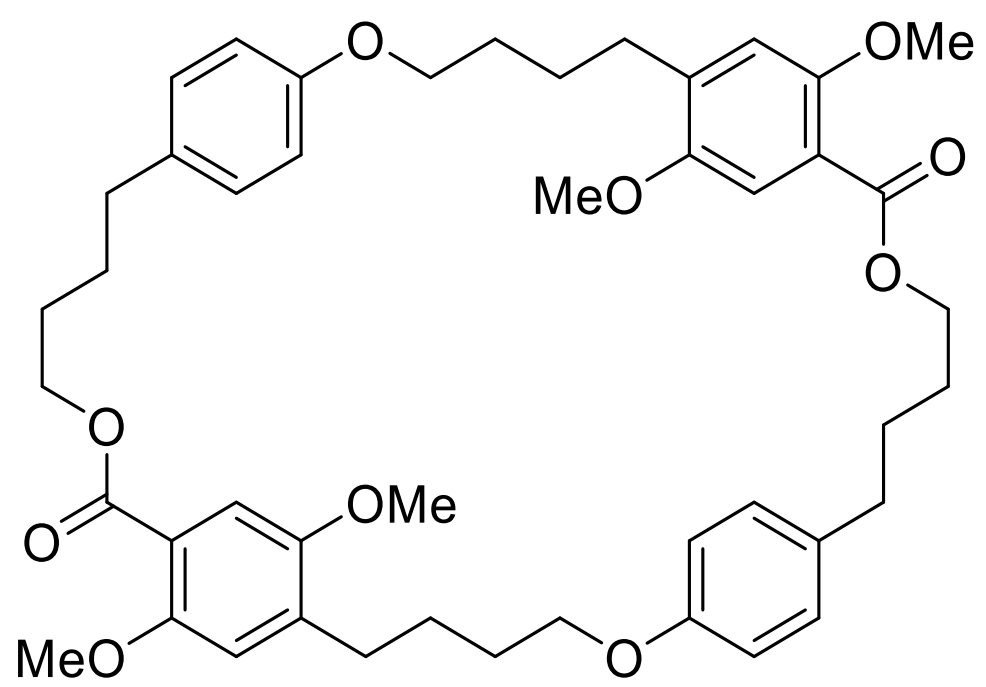

3, white solid. **^{1}H NMR** (600 MHz, $CDCl_3$) δ 7.26 (s, 2H), 7.08 (d, J = 8.6 Hz, 4H), 6.80 – 6.77 (m, 6H), 4.37 – 4.31 (m, 4H), 3.93 (t, J = 5.7 Hz, 4H), 3.80 (s, 6H), 3.77 (s, 6H), 2.68 (t, J = 7.2 Hz, 4H), 2.64 – 2.60 (m, 4H), 1.81 – 1.74 (m, 16H). **^{13}C NMR** (151 MHz, $CDCl_3$) δ 166.5, 157.4, 153.7, 151.1, 137.2, 134.5, 129.4, 117.9, 115.1, 114.5, 113.3, 68.0, 64.7, 56.9, 56.0, 34.7, 30.3, 29.8, 29.1, 28.5, 28.4, 26.5. **HRMS** (ESI): m/z calculated for $C_{46}H_{56}NaO_{10}^+$ [M + Na]$^+$ 791.3766, found 791.3778.

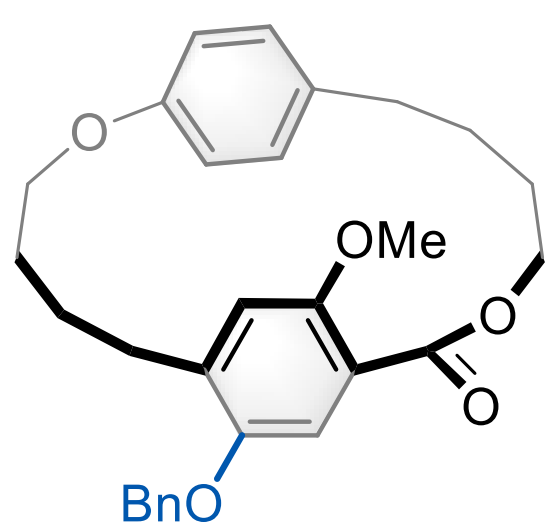

4, yellow oil, 36% yield, 16.6 mg, 94% *ee*. **^{1}H NMR** (600 MHz, $CDCl_3$) δ 7.50 (d, J = 7.5 Hz, 2H), 7.43 (t, J = 7.6 Hz, 2H), 7.36 (t, J = 7.4 Hz, 1H), 7.00 (s, 1H), 6.86 (d, J = 8.5 Hz, 2H), 6.72 (s, 1H), 6.34 (d, J = 8.5 Hz, 2H), 5.01 (d, J = 11.4 Hz, 1H), 4.89 (d, J = 11.4 Hz, 1H), 4.48 – 4.42 (m, 1H), 4.08 (ddd, J = 10.5, 7.0, 2.2 Hz, 1H), 3.81 (s, 3H), 3.66 (ddd, J = 9.9, 6.9, 4.6 Hz, 1H), 3.54 (ddd, J = 9.9, 7.1, 4.6 Hz, 1H), 3.18 (ddd, J = 13.6, 8.3, 2.7 Hz, 1H), 2.49 – 2.33 (m, 3H), 2.27 – 2.20 (m, 1H), 1.89 – 1.66 (m, 7H). **^{13}C NMR** (151 MHz, $CDCl_3$) δ 165.6, 156.5, 154.0, 149.8, 138.0, 137.5,

134.9, 129.2, 128.7, 128.0, 127.6, 117.2, 115.3, 114.9, 114.2, 70.5, 65.9, 64.4, 56.8, 34.3, 30.4, 28.6, 28.3, 27.7, 23.5. **HRMS** (ESI): m/z calculated for $C_{29}H_{32}NaO_5^+$ $[M + Na]^+$ 483.2142, found 483.2140. $[\alpha]_D^{25}$ = -33.4 (c = 0.81, CH_2Cl_2). **HPLC**: Chiralpak IF column, 80:20 hexanes/isopropanol, flow rate = 1.0 mL/min, λ = 254 nm, t_R = 11.31 min (minor), 12.08 min (major).

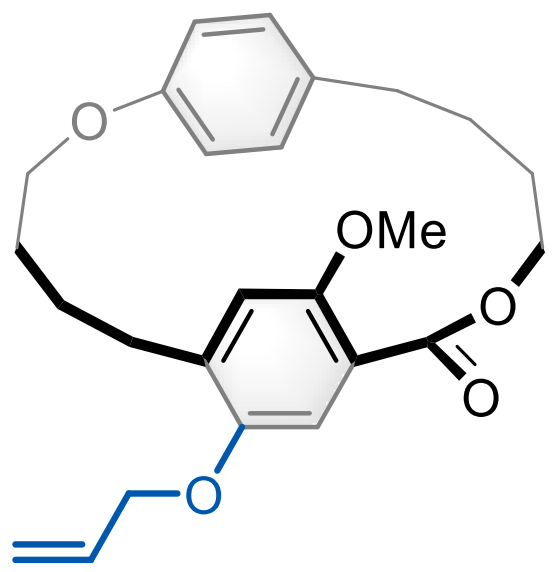

5, colorless oil, 51% yield, 20.9 mg, 94% *ee*. **^{1}H NMR** (600 MHz, $CDCl_3$) δ 6.90 – 6.83 (m, 3H), 6.70 (s, 1H), 6.35 (d, J = 8.2 Hz, 2H), 6.12 – 6.03 (m, 1H), 5.46 (dt, J = 17.3, 1.7 Hz, 1H), 5.30 (dt, J = 10.6, 1.7 Hz, 1H), 4.46 (dddd, J = 14.1, 11.5, 6.2, 2.8 Hz, 2H), 4.40 – 4.35 (m, 1H), 4.06 (ddd, J = 10.5, 7.1, 2.2 Hz, 1H), 3.80 (s, 3H), 3.66 (ddd, J = 11.0, 6.8, 4.6 Hz, 1H), 3.54 (ddd, J = 9.7, 7.2, 4.6 Hz, 1H), 3.15 (ddd, J = 13.5, 8.4, 2.8 Hz, 1H), 2.46 (dd, J = 13.8, 8.9 Hz, 1H), 2.43 – 2.38 (m, 1H), 2.35 (ddd, J = 13.3, 9.8, 2.7 Hz, 1H), 2.25 – 2.16 (m, 1H), 1.87 – 1.78 (m, 2H), 1.78 – 1.71 (m, 4H), 1.70 – 1.65 (m, 1H). **^{13}C NMR** (151 MHz, $CDCl_3$) δ 165.6, 156.5, 153.9, 149.6, 137.9, 134.8, 133.7, 129.2, 117.2, 117.0, 115.2, 114.9, 114.2, 69.3, 65.9, 64.3, 56.8, 34.3, 30.3, 28.6, 28.2, 27.7, 23.5. **HRMS** (ESI): m/z calculated for $C_{25}H_{30}NaO_5^+$ $[M + Na]^+$ 433.1985, found 433.1989. $[\alpha]_D^{25}$ = -38.8 (c = 1.05, CH_2Cl_2). **HPLC**: Chiralpak IF column, 80:20 hexanes/isopropanol, flow rate = 1.0 mL/min, λ = 254 nm, t_R = 8.72 min (minor), 10.48 min (major).

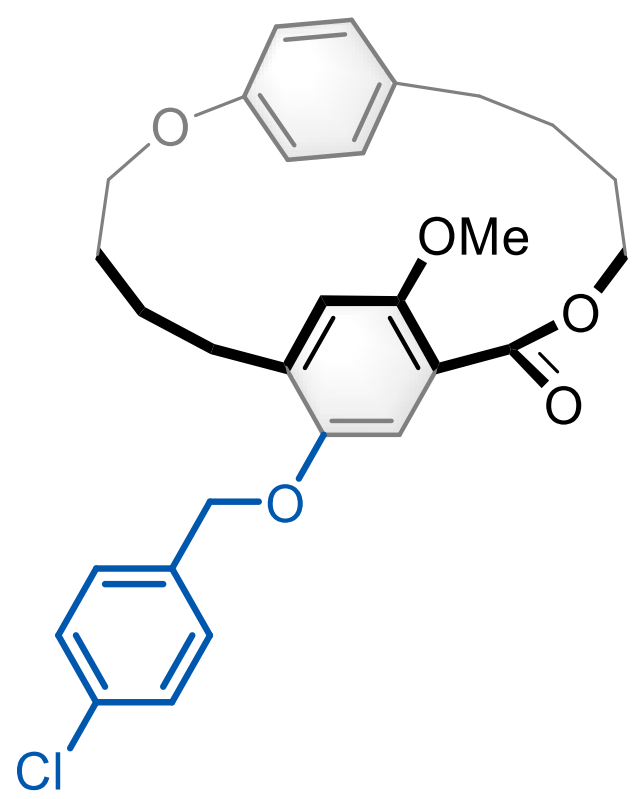

6, yellow solid, 48% yield, 23.7 mg, 94% *ee*. **^{1}H NMR** (600 MHz, $CDCl_3$) δ 7.41 (q, J = 8.5 Hz, 4H), 6.96 (s, 1H), 6.86 (d, J = 8.1 Hz, 2H), 6.72 (s, 1H), 6.33 (d, J = 8.1 Hz, 2H), 4.96 (d, J = 11.6 Hz, 1H), 4.85 (d, J = 11.5 Hz, 1H), 4.45 (ddd, J = 10.1, 6.4, 2.6 Hz, 1H), 4.11 – 4.05 (m, 1H), 3.81 (s, 3H), 3.65 (ddd, J = 10.8, 6.8, 4.7 Hz, 1H), 3.53 (ddd, J = 9.6, 7.2, 4.5 Hz, 1H), 3.14 (ddd, J = 13.5, 8.2, 2.7 Hz, 1H), 2.49 – 2.39 (m, 2H), 2.36 (ddd, J = 13.3, 9.8, 2.6 Hz, 1H), 2.26 – 2.19 (m, 1H), 1.88 – 1.66 (m, 7H). **^{13}C NMR** (151 MHz, $CDCl_3$) δ 165.5, 156.5, 154.1, 149.6, 138.0, 135.9, 134.9, 133.8, 129.2, 128.88, 128.86, 117.2, 115.2, 114.9, 114.1, 69.8, 65.8, 64.4, 56.8, 34.3, 30.5, 28.6, 28.2, 27.7, 23.4. **HRMS** (ESI): m/z calculated for $C_{29}H_{31}ClNaO_5^+$ $[M + Na]^+$ 517.1752, found 517.1756. $[\alpha]_D^{25}$ = -40.9 (c = 1.18, CH_2Cl_2). **HPLC**: Chiralpak IF column, 90:10 hexanes/isopropanol, flow rate = 1.0 mL/min, λ = 254 nm, t_R = 19.90 min (minor), 20.95 min (major).

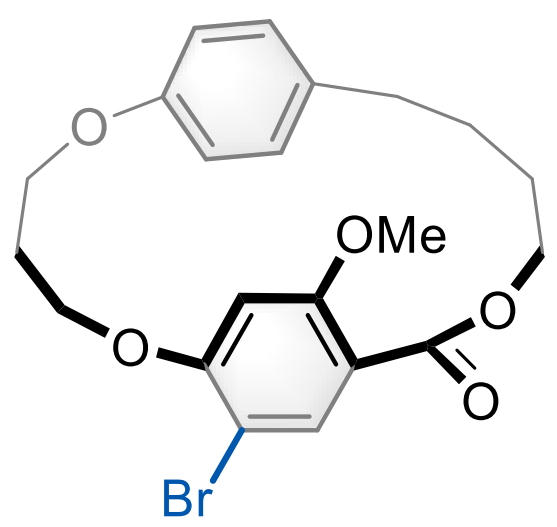

7, yellow solid, 69% yield, 29.9 mg, 96% *ee*. **^{1}H NMR** (600 MHz, $CDCl_3$) δ 7.62 (s, 1H), 6.85 (d, J = 8.4 Hz, 2H), 6.54 (s, 1H), 6.36 (d, J = 8.3 Hz, 2H), 4.75 (td, J = 11.9, 2.6 Hz, 1H), 4.51 (ddd, J = 11.9, 5.2, 2.2 Hz, 1H), 4.32 (ddd, J = 11.0, 6.0, 3.0 Hz, 1H), 4.07 (ddd, J = 10.6, 7.0, 2.4 Hz, 1H), 3.87 – 3.81 (m, 2H), 3.81 (s, 3H), 2.43 (dd, J = 13.7, 8.6 Hz, 1H), 2.39 – 2.33 (m, 1H), 2.23 (dddd, J = 14.6, 11.9, 5.2, 2.5 Hz, 1H), 2.08 – 2.01 (m, 1H), 1.83 – 1.77 (m, 1H), 1.74 – 1.67 (m, 3H). **^{13}C NMR** (151 MHz, $CDCl_3$) δ 164.5, 160.8, 160.2, 156.0, 136.0, 135.1, 129.3, 114.3, 113.1, 103.3, 101.8, 68.2, 64.7, 61.7, 56.1, 34.8, 30.9, 28.6, 27.9. **HRMS** (ESI): m/z calculated for $C_{21}H_{23}BrNaO_5^+$ $[M + Na]^+$ 457.0621, found 457.0624. $[\alpha]_D^{25}$ = -57.7 (c = 0.65, CH_2Cl_2). **HPLC**: Chiralpak IF column, 80:20 hexanes/isopropanol, flow rate = 1.0 mL/min, λ = 254 nm, t_R = 9.66 min (minor), 12.16 min (major).

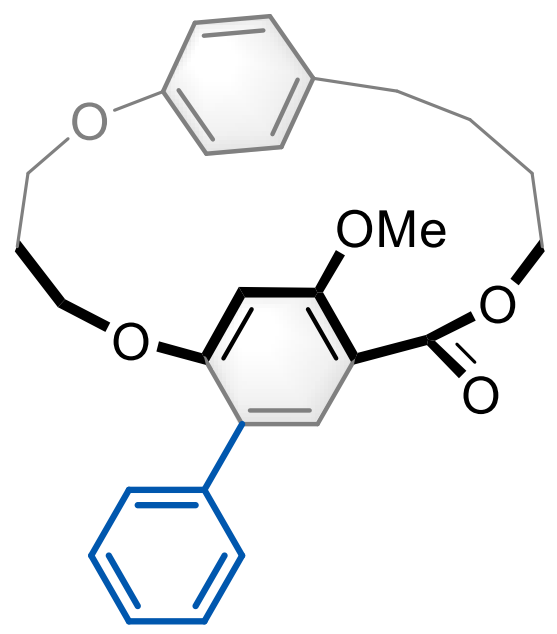

8, colorless oil, 46% yield, 19.9 mg, 94% *ee*. **^{1}H NMR** (600 MHz, $CDCl_3$) δ 7.57 (s, 1H), 7.51 (d, J = 7.6 Hz, 2H), 7.42 (t, J = 7.6 Hz, 2H), 7.31 (t, J = 7.3 Hz, 1H), 6.90 (d, J = 8.3 Hz, 2H), 6.62 (s, 1H), 6.33 (d, J = 8.4 Hz, 2H), 4.61 (ddd, J = 12.7, 10.4, 3.0 Hz, 1H), 4.37 – 4.28 (m, 2H), 4.14 – 4.08 (m, 1H), 3.87 (s, 3H), 3.71 (dt, J = 9.0, 4.1 Hz, 1H), 3.47 (td, J = 9.8, 3.1 Hz, 1H), 2.49 – 2.39 (m, 2H), 1.95 – 1.89 (m, 1H), 1.89 – 1.71 (m, 5H). **^{13}C NMR** (151 MHz, $CDCl_3$) δ 165.6, 161.9, 160.3, 156.2, 137.6, 135.3, 134.4, 129.5, 129.3, 128.2, 126.9, 123.7, 113.4, 113.1, 101.6, 67.6, 64.4, 62.0, 56.1, 34.8, 31.0, 28.8, 28.0. **HRMS** (ESI): m/z calculated for $C_{27}H_{28}NaO_5^+$ $[M + Na]^+$ 455.1829, found 455.1831. $[\alpha]_D^{25}$ = -134.2 (c = 0.69, CH_2Cl_2). **HPLC**: Chiralpak IF column, 80:20 hexanes/isopropanol, flow rate = 1.0 mL/min, λ = 254 nm, t_R = 11.55 min (minor), 12.45 min (major).

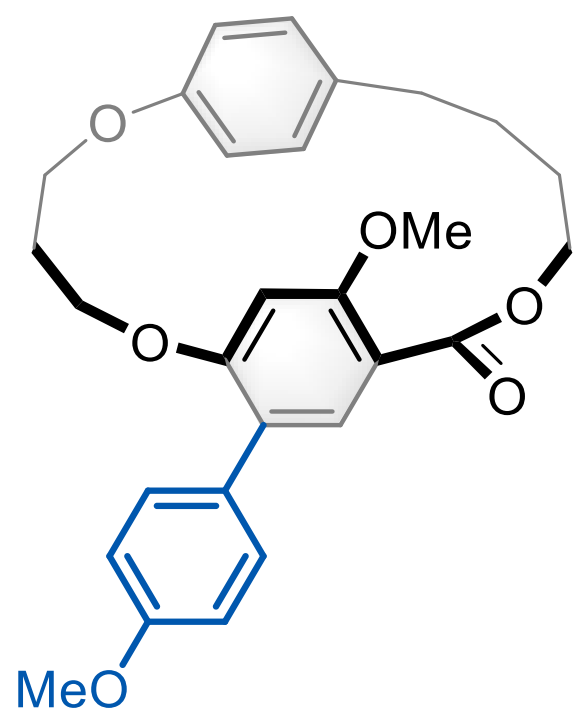

9, yellow oil, 53% yield, 24.5 mg, 96% *ee*. **^{1}H NMR** (600 MHz, $CDCl_3$) δ 7.53 (s, 1H), 7.44 (d, J =

8.7 Hz, 2H), 6.96 (d, J = 8.7 Hz, 2H), 6.89 (d, J = 8.4 Hz, 2H), 6.61 (s, 1H), 6.32 (d, J = 8.4 Hz, 2H), 4.59 (ddd, J = 12.8, 10.3, 3.1 Hz, 1H), 4.36 (ddd, J = 10.9, 6.4, 2.6 Hz, 1H), 4.31 (dt, J = 12.0, 4.1 Hz, 1H), 4.09 (ddd, J = 10.5, 7.4, 2.1 Hz, 1H), 3.86 (s, 6H), 3.70 (dt, J = 8.8, 4.1 Hz, 1H), 3.47 (td, J = 9.9, 3.3 Hz, 1H), 2.50 – 2.38 (m, 2H), 1.95 – 1.71 (m, 6H). **^{13}C NMR** (151 MHz, $CDCl_3$) δ 165.6, 161.9, 160.0, 158.6, 156.2, 135.3, 134.0, 130.6, 130.0, 129.2, 123.4, 113.6, 113.4, 113.1, 101.7, 67.6, 64.4, 62.1, 56.1, 55.4, 34.8, 31.0, 28.8, 28.0. **HRMS** (ESI): m/z calculated for $C_{28}H_{31}O_6^+$ [M + H]$^+$ 463.2115, found 463.2117. $[\alpha]_D^{25}$ = -102.9 (c = 0.38, CH_2Cl_2). **HPLC**: Chiralpak IF column, 80:20 hexanes/isopropanol, flow rate = 1.0 mL/min, λ = 254 nm, t_R = 16.91 min (minor), 20.51 min (major).

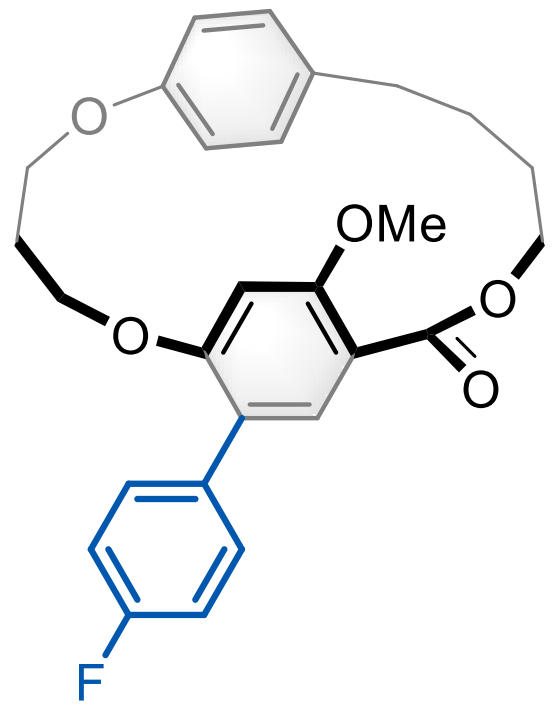

10, yellow solid, 51% yield, 22.9 mg, 95% *ee*. **^{1}H NMR** (600 MHz, $CDCl_3$) δ 7.52 (s, 1H), 7.47 (dd, J = 8.5, 5.5 Hz, 2H), 7.11 (t, J = 8.7 Hz, 2H), 6.89 (d, J = 8.2 Hz, 2H), 6.61 (s, 1H), 6.31 (d, J = 8.1 Hz, 2H), 4.62 (ddd, J = 13.1, 10.4, 2.9 Hz, 1H), 4.35 (ddd, J = 11.0, 6.2, 2.5 Hz, 1H), 4.31 (dt, J = 11.9, 4.0 Hz, 1H), 4.10 (ddd, J = 10.5, 7.4, 1.9 Hz, 1H), 3.87 (s, 3H), 3.71 (dt, J = 8.8, 4.0 Hz, 1H), 3.42 (td, J = 10.0, 3.1 Hz, 1H), 2.50 – 2.38 (m, 2H), 1.97 – 1.89 (m, 1H), 1.88 – 1.72 (m, 5H). **^{13}C NMR** (151 MHz, $CDCl_3$) δ 165.5, 162.0 (d, $J_{C\text{-}F}$ = 245.8 Hz), 161.8, 160.4, 156.2, 135.3, 134.1, 133.5 (d, $J_{C\text{-}F}$ = 3.5 Hz), 131.1 (d, $J_{C\text{-}F}$ = 7.9 Hz), 129.3, 122.6, 115.1 (d, $J_{C\text{-}F}$ = 21.1 Hz), 113.3, 113.1, 101.4, 67.6, 64.4, 62.0, 56.1, 34.8, 30.9, 28.8, 28.0. **^{19}F NMR** (376 MHz, $CDCl_3$) δ -115.85. **HRMS** (ESI): m/z calculated for $C_{28}H_{31}O_6^+$ [M + H]$^+$ 451.1915, found 451.1916. $[\alpha]_D^{25}$ = -99.5 (c = 1.49, CH_2Cl_2). **HPLC**: Chiralpak IF column, 80:20 hexanes/isopropanol, flow rate = 1.0 mL/min, λ = 254 nm, t_R = 9.87 min (minor), 11.08 min (major).

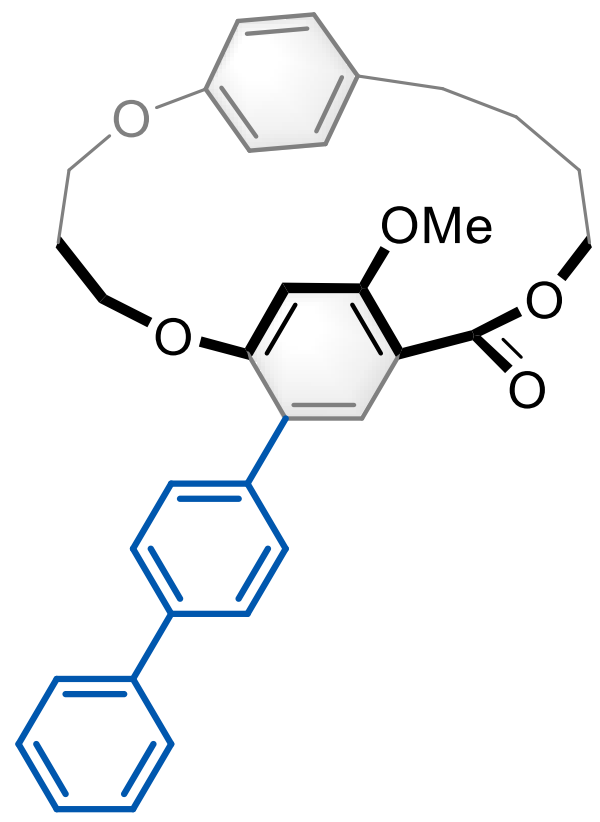

11, yellow solid, 60% yield, 30.5 mg, 95% *ee*. **^{1}H NMR** (600 MHz, $CDCl_3$) δ 7.68 (t, J = 7.4 Hz, 4H), 7.64 – 7.59 (m, 3H), 7.48 (t, J = 7.6 Hz, 2H), 7.37 (t, J = 7.4 Hz, 1H), 6.92 (d, J = 8.1 Hz, 2H), 6.65 (s, 1H), 6.36 (d, J = 8.1 Hz, 2H), 4.65 (ddd, J = 12.7, 9.9, 3.4 Hz, 1H), 4.36 (ddt, J = 12.0, 9.6, 3.2 Hz, 2H), 4.12 (dd, J = 11.0, 7.3 Hz, 1H), 3.89 (s, 3H), 3.73 (dt, J = 8.9, 4.1 Hz, 1H), 3.52 (td, J = 9.6, 3.5 Hz, 1H), 2.46 (qd, J = 13.8, 7.7 Hz, 2H), 1.93 (td, J = 10.4, 4.5 Hz, 2H), 1.89 – 1.73 (m,

4H). **^{13}C NMR** (151 MHz, $CDCl_3$) δ 165.5, 162.0, 160.4, 156.2, 141.0, 139.5, 136.6, 135.3, 134.3, 129.8, 129.3, 128.9, 127.4, 127.1, 126.9, 123.2, 113.4, 113.2, 101.6, 67.6, 64.4, 62.1, 56.1, 34.8, 31.0, 28.8, 28.0. **HRMS** (ESI): m/z calculated for $C_{33}H_{33}O_5^+$ $[M + H]^+$ 509.2323, found 509.2326. $[\alpha]_D^{25}$ = -94.2 (c = 0.95, CH_2Cl_2). **HPLC**: Chiralpak IF column, 80:20 hexanes/isopropanol, flow rate = 1.0 mL/min, λ = 254 nm, t_R = 19.12 min (minor), 22.92 min (major).

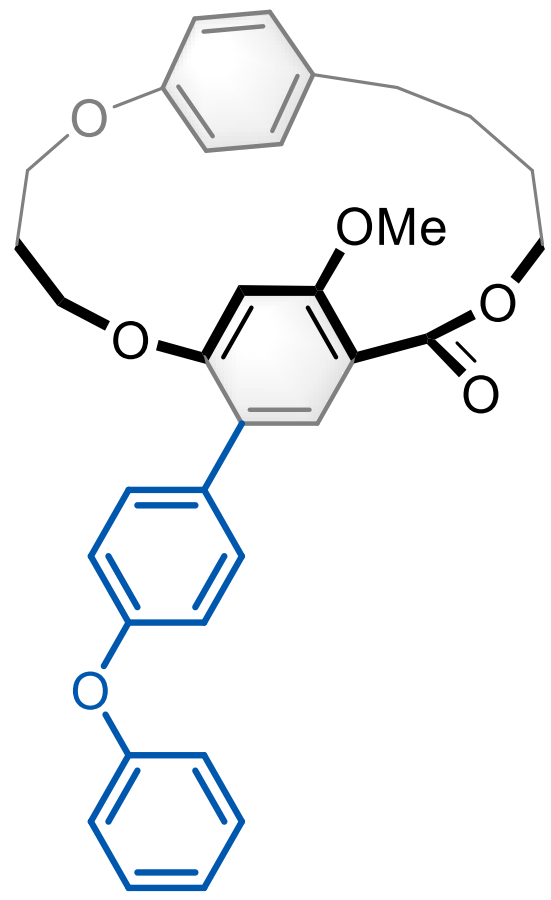

12, yellow solid, 57% yield, 29.9 mg, 95% *ee*. **^{1}H NMR** (600 MHz, $CDCl_3$) δ 7.55 (s, 1H), 7.48 (d, J = 8.6 Hz, 2H), 7.40 – 7.35 (m, 2H), 7.13 (t, J = 7.4 Hz, 1H), 7.10 – 7.04 (m, 4H), 6.89 (d, J = 8.4 Hz, 2H), 6.62 (s, 1H), 6.32 (d, J = 8.5 Hz, 2H), 4.63 (ddd, J = 13.0, 10.3, 3.1 Hz, 1H), 4.35 (ddt, J = 20.1, 12.0, 4.9 Hz, 2H), 4.09 (ddd, J = 10.5, 7.5, 2.0 Hz, 1H), 3.87 (s, 3H), 3.73 (dt, J = 8.8, 4.1 Hz, 1H), 3.48 (td, J = 9.9, 3.3 Hz, 1H), 2.48 – 2.38 (m, 2H), 1.93 (dddd, J = 19.8, 14.6, 10.5, 3.9 Hz, 2H), 1.86 – 1.70 (m, 4H). **^{13}C NMR** (151 MHz, $CDCl_3$) δ 165.5, 161.9, 160.2, 157.3, 156.3, 156.2, 135.3, 134.2, 132.6, 130.8, 129.9, 129.2, 123.5, 123.0, 119.2, 118.4, 113.3, 113.1, 101.5, 67.6, 64.4, 62.1, 56.1, 34.8, 31.0, 28.8, 28.0. **HRMS** (ESI): m/z calculated for $C_{33}H_{33}O_6^+$ $[M + H]^+$ 525.2272, found 525.2276. $[\alpha]_D^{25}$ = -113.2 (c = 1.00, CH_2Cl_2). **HPLC**: Chiralpak IF column, 80:20 hexanes/isopropanol, flow rate = 1.0 mL/min, λ = 254 nm, t_R = 13.77 min (minor), 17.75 min (major).

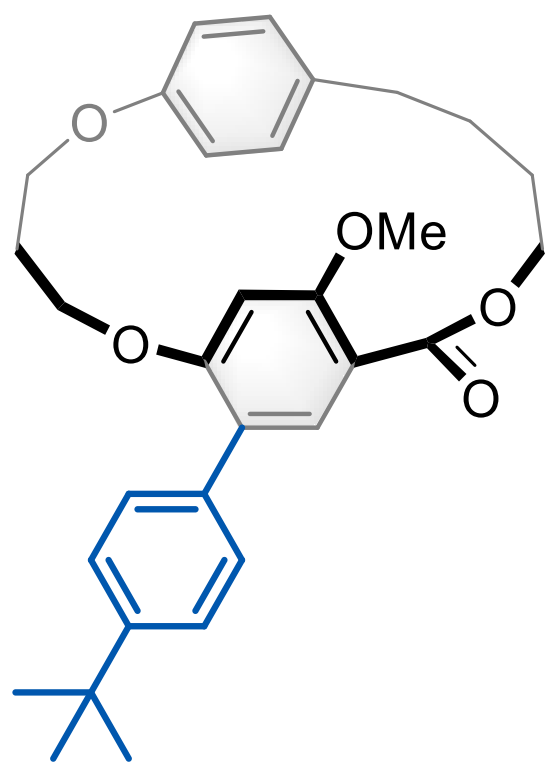

13, colorless solid, 49% yield, 23.9 mg, 97% *ee*. **^{1}H NMR** (600 MHz, $CDCl_3$) δ 7.57 (s, 1H), 7.47 – 7.41 (m, 4H), 6.90 (d, J = 8.5 Hz, 2H), 6.61 (s, 1H), 6.34 (d, J = 8.6 Hz, 2H), 4.61 (ddd, J = 12.7, 9.5, 3.8 Hz, 1H), 4.36 – 4.28 (m, 2H), 4.10 (ddd, J = 10.9, 7.2, 2.1 Hz, 1H), 3.86 (s, 3H), 3.73 (dt, J = 8.9, 4.2 Hz, 1H), 3.52 (td, J = 9.4, 3.9 Hz, 1H), 2.43 (td, J = 7.4, 2.1 Hz, 2H), 1.98 – 1.88 (m, 2H), 1.86 – 1.69 (m, 4H), 1.37 (s, 9H). **^{13}C NMR** (151 MHz, $CDCl_3$) δ 165.6, 161.9, 160.1, 156.2, 149.7, 135.2, 134.6, 134.5, 129.3, 129.0, 125.1, 123.5, 113.4, 113.0, 101.5, 67.4, 64.4, 62.1, 56.1, 34.8, 34.7, 31.5, 31.0, 28.8, 28.0. **HRMS** (ESI): m/z calculated for $C_{31}H_{37}O_5^+$ $[M + H]^+$ 489.2636, found 489.2634. $[\alpha]_D^{25}$ = -119.1 (c = 0.95, CH_2Cl_2). **HPLC**: Chiralpak IB column, 80:20

hexanes/isopropanol, flow rate = 1.0 mL/min, λ = 254 nm, t_R = 5.92 min (major), 10.22 min (minor).

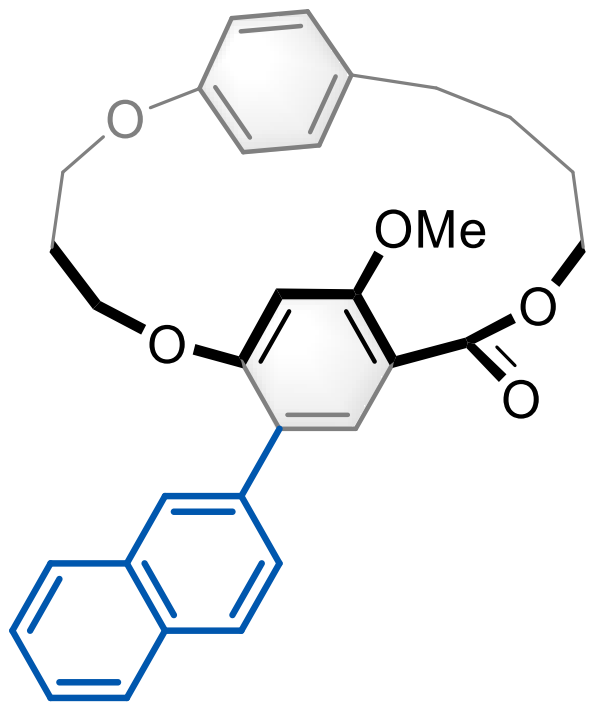

14, yellow oil, 45% yield, 21.7 mg, 96% *ee*. **^{1}H NMR** (600 MHz, $CDCl_3$) δ 7.96 (s, 1H), 7.93 (d, *J* = 7.8 Hz, 1H), 7.88 (d, *J* = 8.2 Hz, 2H), 7.70 (s, 1H), 7.68 (dd, *J* = 8.5, 1.7 Hz, 1H), 7.55 – 7.48 (m, 2H), 6.95 (d, *J* = 8.2 Hz, 2H), 6.67 (s, 1H), 6.36 (d, *J* = 8.2 Hz, 2H), 4.62 (ddd, *J* = 12.7, 10.0, 2.9 Hz, 1H), 4.38 (ddd, *J* = 8.7, 6.1, 2.3 Hz, 1H), 4.33 (dt, *J* = 12.1, 4.1 Hz, 1H), 4.14 (ddd, *J* = 10.5, 7.3, 2.2 Hz, 1H), 3.90 (s, 3H), 3.66 (dt, *J* = 8.9, 4.2 Hz, 1H), 3.47 (td, *J* = 9.9, 3.1 Hz, 1H), 2.53 – 2.42 (m, 2H), 1.93 – 1.86 (m, 2H), 1.83 – 1.76 (m, 4H). **^{13}C NMR** (151 MHz, $CDCl_3$) δ 165.6, 162.2, 160.5, 156.3, 135.4, 135.3, 134.7, 133.6, 132.5, 129.3, 128.3, 128.10, 128.07, 127.8, 127.5, 126.1, 125.9, 123.6, 113.4, 113.3, 101.7, 67.8, 64.4, 62.1, 56.1, 34.8, 30.9, 28.8, 28.0. **HRMS** (ESI): m/z calculated for $C_{31}H_{30}NaO_5^+$ $[M + Na]^+$ 505.1985, found 505.1985. $[\alpha]_D^{25}$ = -167.7 (c = 0.38, CH_2Cl_2). **HPLC**: Chiralpak IF column, 80:20 hexanes/isopropanol, flow rate = 1.0 mL/min, λ = 254 nm, t_R = 14.63 min (minor), 16.05 min (major).

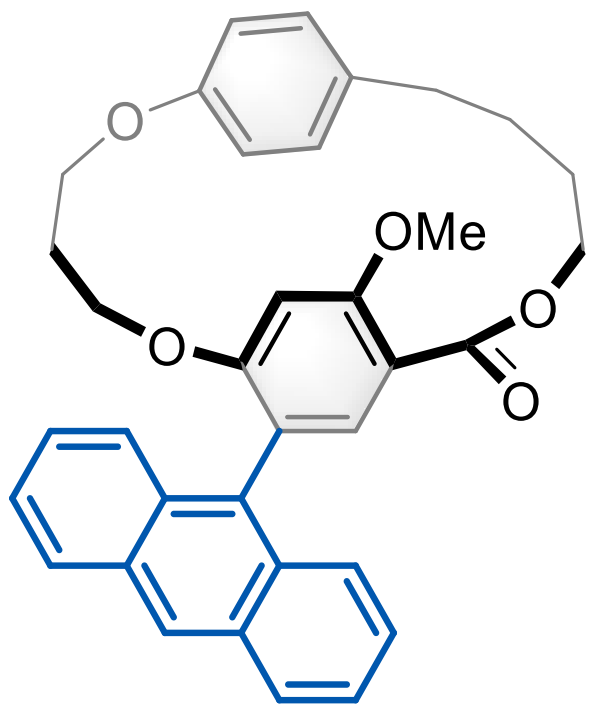

15, yellow solid, 43% yield, 22.9 mg, 94% *ee*. **^{1}H NMR** (600 MHz, $CDCl_3$) δ 8.49 (s, 1H), 8.08 (d, *J* = 7.5 Hz, 1H), 8.02 (d, *J* = 8.4 Hz, 1H), 7.94 (d, *J* = 8.4 Hz, 1H), 7.62 (s, 1H), 7.57 – 7.50 (m, 3H), 7.46 – 7.41 (m, 1H), 7.36 – 7.31 (m, 1H), 7.07 (d, *J* = 8.2 Hz, 2H), 6.74 (s, 1H), 6.64 (d, *J* = 8.3 Hz, 2H), 4.45 (ddd, *J* = 12.5, 9.8, 2.9 Hz, 1H), 4.41 – 4.36 (m, 1H), 4.05 (ddd, *J* = 10.3, 8.3, 1.8 Hz, 1H), 3.93 (s, 3H), 3.88 (dt, *J* = 12.2, 4.4 Hz, 1H), 3.78 (dt, *J* = 9.1, 4.3 Hz, 1H), 3.59 (td, *J* = 9.8, 2.7 Hz, 1H), 2.61 (dd, *J* = 13.9, 7.7 Hz, 1H), 2.44 (dd, *J* = 14.2, 8.5 Hz, 1H), 1.89 – 1.70 (m, 5H), 1.52 – 1.41 (m, 1H). **^{13}C NMR** (151 MHz, $CDCl_3$) δ 166.9, 162.4, 160.0, 156.7, 136.4, 135.6, 132.6, 131.9, 131.4, 130.8, 130.2, 129.5, 128.9, 128.5, 127.2, 127.0, 125.5, 125.3, 125.2, 125.0, 119.3, 113.9, 113.4, 99.7, 65.1, 64.7, 62.3, 55.7, 34.6, 30.6, 28.7, 28.2. **HRMS** (ESI): m/z calculated for $C_{35}H_{32}NaO_5^+$ $[M + Na]^+$ 555.2142, found 555.2144. $[\alpha]_D^{25}$ = -228.4 (c = 0.80, CH_2Cl_2). **HPLC**: Chiralpak IF column, 80:20 hexanes/isopropanol, flow rate = 1.0 mL/min, λ = 254 nm, t_R = 10.98 min (major), 12.14 min (minor).

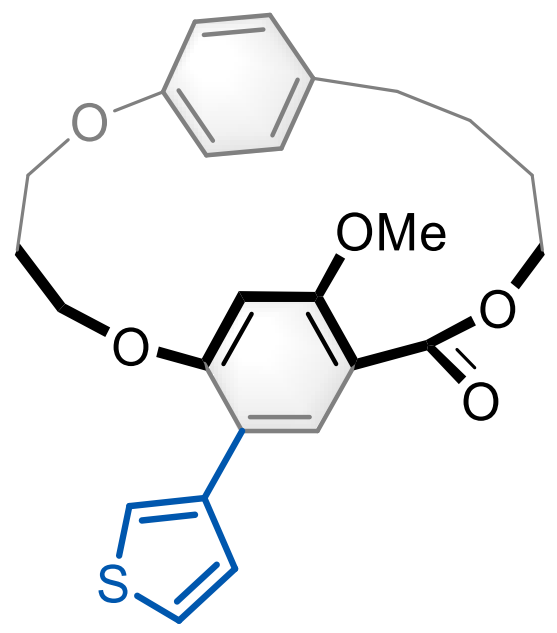

16, yellow oil, 47% yield, 20.6 mg, 94% *ee*. **^{1}H NMR** (600 MHz, $CDCl_3$) δ 7.67 (s, 1H), 7.57 (dd, J = 3.1, 1.4 Hz, 1H), 7.40 (d, J = 5.0 Hz, 1H), 7.35 (dd, J = 5.0, 3.0 Hz, 1H), 6.86 (d, J = 8.1 Hz, 2H), 6.61 (s, 1H), 6.27 (d, J = 8.1 Hz, 2H), 4.71 (ddd, J = 11.4, 9.4, 4.2 Hz, 1H), 4.43 (dt, J = 11.9, 3.9 Hz, 1H), 4.39 (ddd, J = 10.5, 5.5, 2.3 Hz, 1H), 4.07 (ddd, J = 10.6, 7.6, 2.4 Hz, 1H), 3.86 (s, 3H), 3.68 (dt, J = 8.7, 3.9 Hz, 1H), 3.48 (td, J = 9.5, 4.4 Hz, 1H), 2.47 (dd, J = 13.7, 8.7 Hz, 1H), 2.42 – 2.34 (m, 1H), 2.02 – 1.94 (m, 2H), 1.87 (q, J = 6.9 Hz, 1H), 1.80 – 1.69 (m, 3H). **^{13}C NMR** (151 MHz, $CDCl_3$) δ 165.4, 161.9, 160.1, 156.2, 137.5, 135.2, 133.2, 129.2, 128.4, 124.6, 122.5, 118.6, 113.3, 113.0, 101.5, 67.9, 64.4, 61.8, 56.1, 34.8, 31.0, 28.8, 28.0. **HRMS** (ESI): m/z calculated for $C_{25}H_{26}NaO_5S^+$ $[M + Na]^+$ 461.1393, found 461.1395. $[\alpha]_D^{25}$ = -135.7 (c = 1.17, CH_2Cl_2). **HPLC**: Chiralpak IF column, 80:20 hexanes/isopropanol, flow rate = 1.0 mL/min, λ = 254 nm, t_R = 14.62 min (major), 15.56 min (minor).

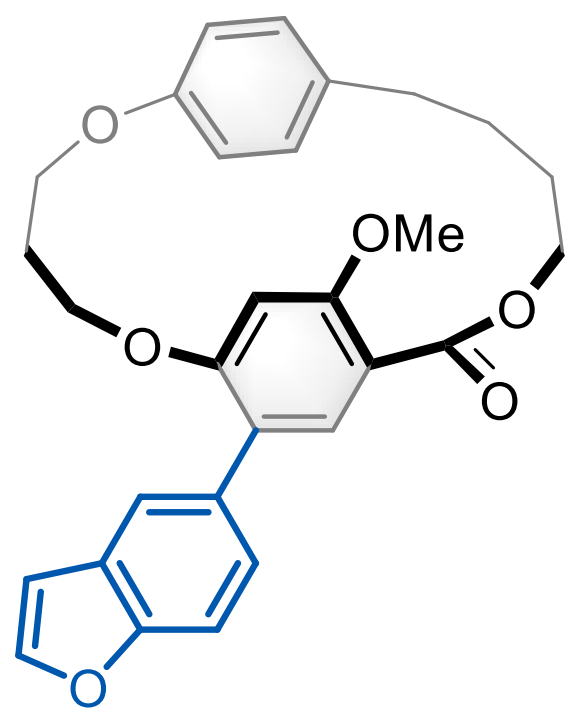

17, yellow solid, 65% yield, 30.7 mg, 95% *ee*. **^{1}H NMR** (600 MHz, $CDCl_3$) δ 7.72 (s, 1H), 7.66 (s, 1H), 7.60 (s, 1H), 7.54 (d, J = 8.5 Hz, 1H), 7.44 (d, J = 8.6 Hz, 1H), 6.92 (d, J = 8.0 Hz, 2H), 6.84 (s, 1H), 6.64 (s, 1H), 6.35 (d, J = 7.9 Hz, 2H), 4.60 (t, J = 10.7 Hz, 1H), 4.38 – 4.26 (m, 2H), 4.14 (t, J = 8.8 Hz, 1H), 3.88 (s, 3H), 3.72 – 3.67 (m, 1H), 3.53 – 3.42 (m, 1H), 2.49 – 2.41 (m, 2H), 1.94 – 1.75 (m, 6H). **^{13}C NMR** (151 MHz, $CDCl_3$) δ 165.7, 161.9, 160.1, 156.3, 154.2, 145.3, 135.3, 134.7, 132.5, 129.3, 127.4, 126.3, 124.0, 122.1, 113.4, 111.0, 107.0, 101.6, 67.5, 64.4, 62.1, 56.1, 34.8, 31.0, 28.8, 28.0. **HRMS** (ESI): m/z calculated for $C_{29}H_{28}NaO_6^+$ $[M + Na]^+$ 495.1778, found 495.1775. $[\alpha]_D^{25}$ = -177.1 (c = 0.99, CH_2Cl_2). **HPLC**: Chiralpak IF column, 80:20 hexanes/isopropanol, flow rate = 1.0 mL/min, λ = 254 nm, t_R = 13.81 min (minor), 17.71 min (major).

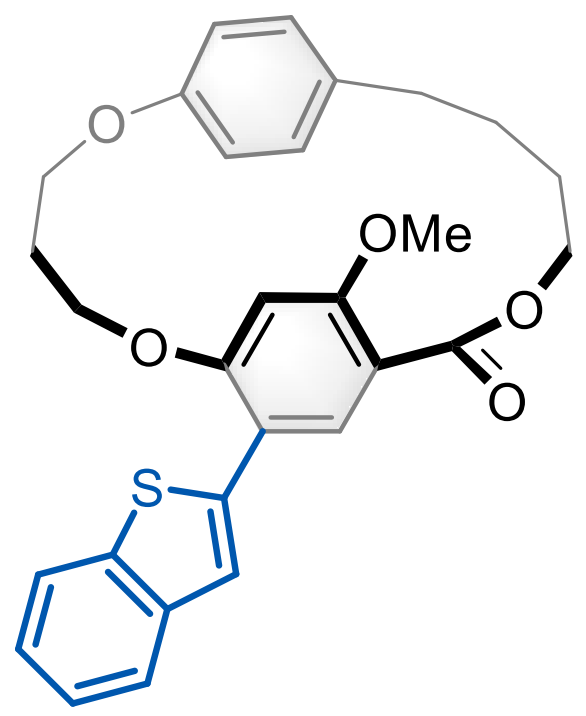

18, yellow oil, 54% yield, 26.4 mg, 96% *ee*. **^{1}H NMR** (600 MHz, $CDCl_3$) δ 7.88 (s, 1H), 7.85 (d, J = 7.9 Hz, 1H), 7.80 (d, J = 7.8 Hz, 1H), 7.67 (s, 1H), 7.34 (dt, J = 27.3, 7.4 Hz, 2H), 6.87 (d, J = 7.9 Hz, 2H), 6.63 (s, 1H), 6.30 (d, J = 8.1 Hz, 2H), 4.85 (t, J = 11.5 Hz, 1H), 4.61 – 4.54 (m, 1H), 4.39 – 4.35 (m, 1H), 4.14 (dd, J = 10.5, 6.3 Hz, 1H), 3.87 (s, 3H), 3.69 (ddd, J = 9.4, 4.7, 2.5 Hz, 1H), 3.62 (t, J = 10.6 Hz, 1H), 2.48 – 2.44 (m, 1H), 2.42 – 2.36 (m, 1H), 2.28 – 2.19 (m, 2H), 2.05 – 1.98 (m, 1H), 1.80 – 1.73 (m, 3H). **^{13}C NMR** (151 MHz, $CDCl_3$) δ 165.3, 161.4, 160.6, 156.1, 140.1, 139.8, 139.4, 135.1, 132.9, 129.3, 124.4, 124.1, 123.4, 122.0, 121.4, 116.7, 113.5, 113.2, 101.1, 68.0, 64.6, 61.8, 56.1, 34.8, 30.9, 28.7, 27.9. **HRMS** (ESI): m/z calculated for $C_{25}H_{26}NaO_5S^+$ [M + Na]$^+$ 511.1550, found 511.1553. $[\alpha]_D^{25}$ = -225.7 (c = 1.57, CH_2Cl_2). **HPLC**: Chiralpak IF column, 80:20 hexanes/isopropanol, flow rate = 1.0 mL/min, λ = 254 nm, t_R = 16.23 min (major), 21.43 min (minor).

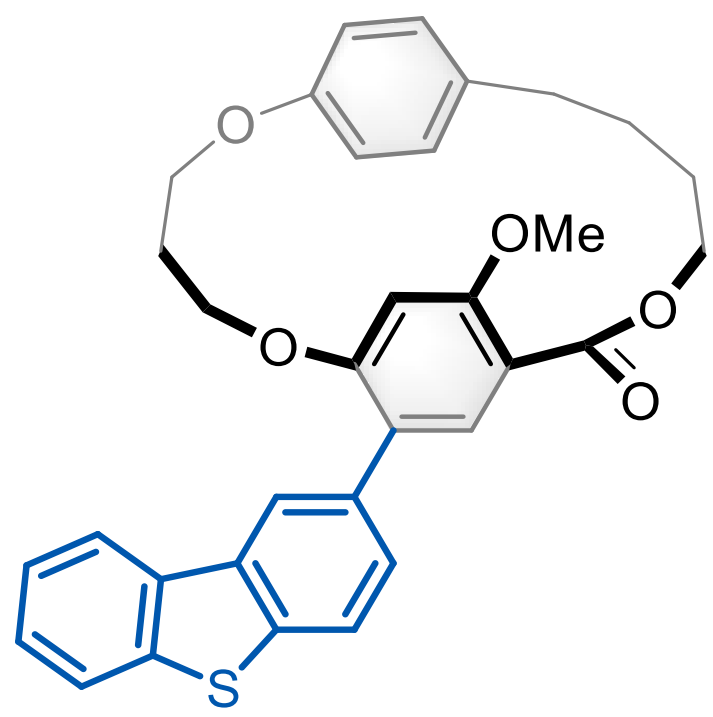

19, yellow oil, 47% yield, 25.3 mg, 96% *ee*. **^{1}H NMR** (600 MHz, $CDCl_3$) δ 8.27 (d, J = 1.7 Hz, 1H), 8.24 (d, J = 7.2 Hz, 1H), 7.89 (dd, J = 8.0, 2.9 Hz, 2H), 7.68 (s, 1H), 7.63 (dd, J = 8.3, 1.7 Hz, 1H), 7.55 – 7.47 (m, 2H), 6.96 (d, J = 8.5 Hz, 2H), 6.68 (s, 1H), 6.37 (d, J = 8.5 Hz, 2H), 4.63 (ddd, J = 13.0, 10.5, 2.9 Hz, 1H), 4.40 (ddd, J = 11.0, 6.1, 2.6 Hz, 1H), 4.34 (dt, J = 12.0, 4.1 Hz, 1H), 4.13 (ddd, J = 10.7, 7.4, 2.2 Hz, 1H), 3.91 (s, 3H), 3.69 (dt, J = 8.8, 4.1 Hz, 1H), 3.48 (td, J = 9.9, 3.1 Hz, 1H), 2.52 (dd, J = 13.8, 8.7 Hz, 1H), 2.45 (dd, J = 14.0, 7.8 Hz, 1H), 1.96 – 1.88 (m, 2H), 1.85 – 1.75 (m, 4H). **^{13}C NMR** (151 MHz, $CDCl_3$) δ 165.5, 162.1, 160.5, 156.3, 140.0, 138.0, 135.9, 135.7, 135.4, 134.6, 134.1, 129.3, 128.5, 126.9, 124.6, 123.5, 123.1, 122.5, 122.4, 121.7, 113.5, 113.3, 101.7, 67.8, 64.4, 62.2, 56.2, 34.8, 30.9, 28.9, 28.0. **HRMS** (ESI): m/z calculated for $C_{33}H_{30}NaO_5S^+$ [M + Na]$^+$ 561.1706, found 561.1706. $[\alpha]_D^{25}$ = -137.6 (c = 0.50, CH_2Cl_2). **HPLC**: Chiralpak IF column, 80:20 hexanes/isopropanol, flow rate = 1.0 mL/min, λ = 254 nm, t_R = 22.05 min (minor), 28.45 min (major).

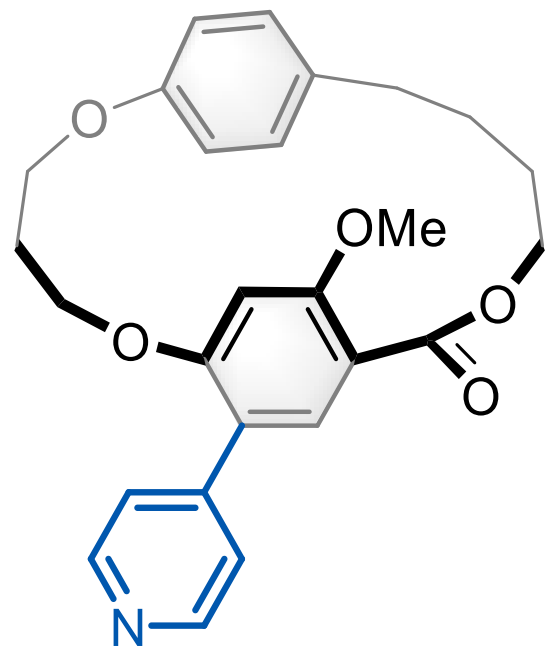

20, yellow oil, 53% yield, 22.9 mg, 96% *ee*. **^{1}H NMR** (400 MHz, $CDCl_3$) δ 8.64 (s, 2H), 7.59 (s, 1H), 7.47 (d, J = 5.8 Hz, 2H), 6.89 (d, J = 8.5 Hz, 2H), 6.63 (s, 1H), 6.28 (d, J = 8.5 Hz, 2H), 4.69 (td, J = 10.9, 10.4, 3.5 Hz, 1H), 4.42 – 4.33 (m, 2H), 4.08 (ddd, J = 10.5, 6.9, 2.7 Hz, 1H), 3.89 (s, 3H), 3.68 (dt, J = 9.5, 3.9 Hz, 1H), 3.36 (td, J = 9.9, 3.6 Hz, 1H), 2.53 – 2.35 (m, 2H), 2.05 – 1.89 (m, 2H), 1.86 – 1.66 (m, 4H). **^{13}C NMR** (101 MHz, $CDCl_3$) δ 165.1, 162.3, 161.5, 156.1, 149.7, 145.3, 135.4, 134.0, 129.3, 124.1, 120.4, 113.4, 113.2, 101.2, 67.8, 64.6, 61.9, 56.1, 34.8, 30.8, 28.8, 28.0. **HRMS** (ESI): m/z calculated for $C_{26}H_{27}NaNO_5^+$ $[M + Na]^+$ 456.1781, found 456.1783. $[\alpha]_D^{25}$ = -98.9 (c = 1.33, CH_2Cl_2). **HPLC**: Chiralpak IF column, 65:35 hexanes/isopropanol, flow rate = 1.0 mL/min, λ = 254 nm, t_R = 13.35 min (minor), 26.20 min (major)

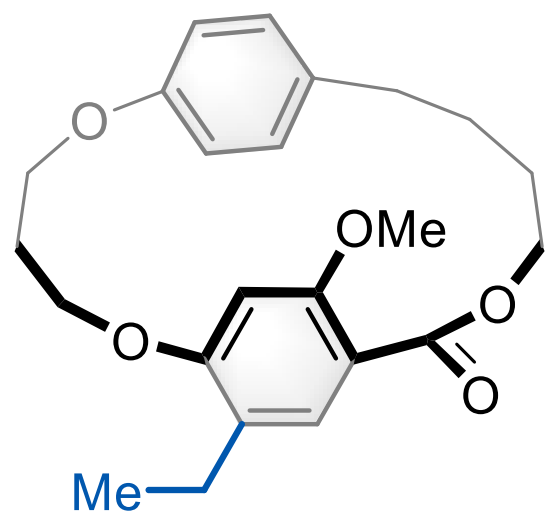

21, yellow oil, 28% yield, 10.8 mg, 94% *ee*. **^{1}H NMR** (400 MHz, $CDCl_3$) δ 7.39 (s, 1H), 6.83 (d, J = 8.2 Hz, 2H), 6.44 (s, 1H), 6.31 (d, J = 8.3 Hz, 2H), 4.76 (td, J = 11.6, 2.8 Hz, 1H), 4.39 – 4.28 (m, 2H), 4.15 – 4.06 (m, 1H), 3.80 (dt, J = 9.6, 3.8 Hz, 1H), 3.73 (s, 3H), 3.57 (td, J = 10.5, 9.6, 3.1 Hz, 1H), 2.76 – 2.63 (m, 1H), 2.55 – 2.42 (m, 2H), 2.39 – 2.31 (m, 1H), 2.19 – 1.99 (m, 2H), 1.80 – 1.63 (m, 4H), 1.16 (t, J = 7.5 Hz, 3H). **^{13}C NMR** (101 MHz, $CDCl_3$) δ 166.6, 162.1, 159.1, 156.3, 135.2, 132.8, 129.3, 124.9, 113.2, 112.4, 99.6, 66.0, 64.4, 62.0, 55.9, 34.6, 31.4, 28.6, 28.0, 22.6, 14.5. **HRMS** (ESI): m/z calculated for $C_{23}H_{29}O_5^+$ $[M + H]^+$ 385.2010, found 385.2013. $[\alpha]_D^{25}$ = -4.9 (c = 0.43, CH_2Cl_2). **HPLC**: Chiralpak IF column, 80:20 hexanes/isopropanol, flow rate = 1.0 mL/min, λ = 254 nm, t_R = 8.26 min (minor), 10.61 min (major).

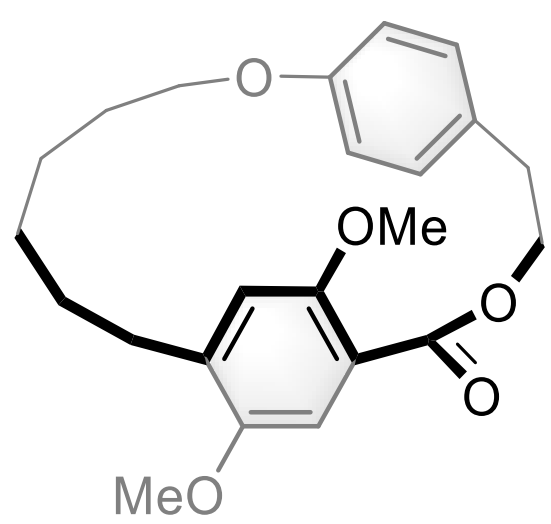

22, yellow oil, 30% yield, 11.5 mg, 90% *ee*. **^{1}H NMR** (400 MHz, $CDCl_3$) δ 7.00 (d, J = 8.5 Hz, 2H), 6.85 (s, 1H), 6.60 – 6.52 (m, 3H), 4.99 – 4.91 (m, 1H), 4.46 – 4.38 (m, 1H), 3.79 (s, 3H), 3.78 – 3.73 (m, 1H), 3.71 (s, 3H), 3.67 (dt, J = 9.9, 6.0 Hz, 1H), 3.10 (dt, J = 14.0, 6.0 Hz, 1H), 3.07 – 2.98 (m, 2H), 2.27 – 2.17 (m, 1H), 1.77 – 1.65 (m, 1H), 1.54 (p, J = 6.5 Hz, 2H), 1.50 – 1.39 (m, 1H),

1.25 – 1.18 (m, 2H), 1.03 – 0.77 (m, 2H). **^{13}C NMR** (101 MHz, $CDCl_3$) δ 165.4, 158.0, 154.3, 150.8, 137.5, 130.3, 129.4, 116.5, 115.5, 115.0, 113.7, 67.1, 62.8, 57.0, 55.8, 34.0, 30.2, 28.4, 27.9, 25.2, 24.9. **HRMS** (ESI): m/z calculated for $C_{23}H_{29}O_5^+$ $[M + H]^+$ 385.2010, found 385.2012. $[\alpha]_D^{25}$ = -53.6 (c = 0.53, CH_2Cl_2). **HPLC**: Chiralpak IF column, 80:20 hexanes/isopropanol, flow rate = 1.0 mL/min, λ = 254 nm, t_R = 9.07 min (minor), 10.23 min (major).

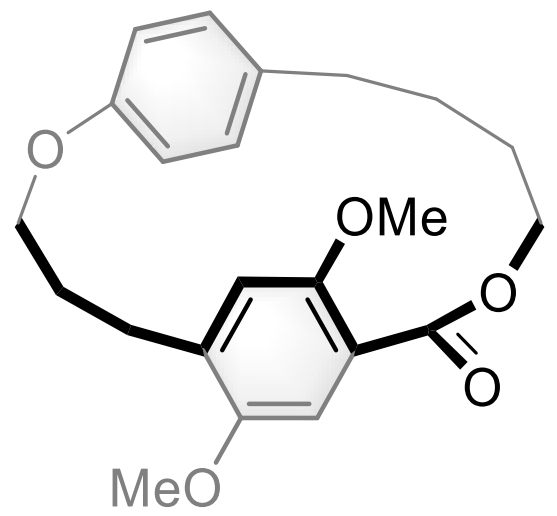

23, yellow oil, 20% yield, 7.4 mg, 95% *ee*. **^{1}H NMR** (600 MHz, $CDCl_3$) δ 6.94 (s, 1H), 6.77 (d, J = 8.4 Hz, 2H), 6.67 (s, 1H), 6.28 (d, J = 8.5 Hz, 2H), 4.25 (dd, J = 10.8, 6.4 Hz, 1H), 4.17 (dd, J = 11.1, 5.9 Hz, 1H), 3.99 – 3.90 (m, 2H), 3.82 (s, 3H), 3.78 (s, 3H), 3.22 (dt, J = 12.9, 6.3 Hz, 1H), 2.47 – 2.30 (m, 4H), 2.07 – 1.99 (m, 1H), 1.79 – 1.66 (m, 4H). **^{13}C NMR** (151 MHz, $CDCl_3$) δ 165.8, 156.3, 153.8, 151.0, 135.5, 134.4, 128.9, 117.1, 116.0, 113.5, 113.4, 65.6, 64.3, 56.7, 55.7, 34.5, 28.6, 28.5, 28.0, 27.3. **HRMS** (ESI): m/z calculated for $C_{22}H_{27}O_5^+$ $[M + H]^+$ 371.1853, found 371.1858. $[\alpha]_D^{25}$ = -44.9 (c = 0.54, CH_2Cl_2). **HPLC**: Chiralpak IF column, 80:20 hexanes/isopropanol, flow rate = 1.0 mL/min, λ = 254 nm, t_R = 10.28 min (minor), 12.16 min (major).

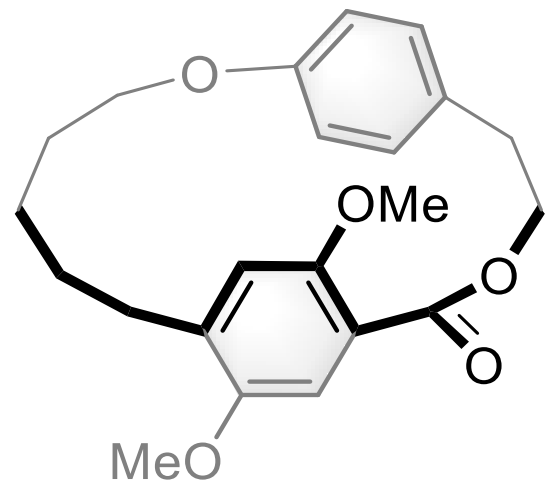

24, yellow oil, 19% yield, 7.0 mg, 91% *ee*. **^{1}H NMR** (600 MHz, $CDCl_3$) δ 6.98 – 6.94 (m, 3H), 6.59 (s, 1H), 6.49 (d, J = 8.1 Hz, 2H), 4.91 – 4.85 (m, 1H), 4.49 (dt, J = 11.0, 6.3 Hz, 1H), 3.81 (s, 3H), 3.79 – 3.76 (m, 2H), 3.75 (s, 3H), 3.09 – 3.03 (m, 2H), 3.02 – 2.97 (m, 1H), 2.24 (ddd, J = 12.9, 8.6, 4.0 Hz, 1H), 1.84 – 1.77 (m, 1H), 1.57 – 1.49 (m, 1H), 1.24 – 1.20 (m, 1H), 1.17 – 1.02 (m, 3H). **^{13}C NMR** (151 MHz, $CDCl_3$) δ 165.7, 156.9, 154.4, 150.7, 137.5, 130.5, 129.3, 116.6, 115.34, 115.27, 114.2, 67.4, 63.2, 56.9, 55.9, 34.3, 30.5, 29.0, 28.0, 23.2. **HRMS** (ESI): m/z calculated for $C_{22}H_{27}O_5^+$ $[M + H]^+$ 371.1853, found 371.1855. $[\alpha]_D^{25}$ = -50.6 (c = 0.54, CH_2Cl_2). **HPLC**: Chiralpak IF column, 80:20 hexanes/isopropanol, flow rate = 1.0 mL/min, λ = 254 nm, t_R = 9.54 min (minor), 11.15 min (major).

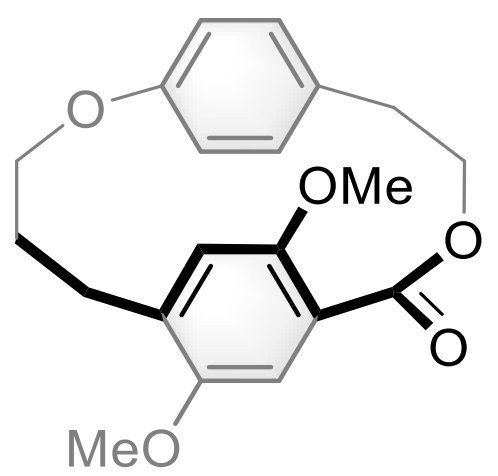

25, white solid, 7% yield, 2.4 mg, 93% *ee*. **^{1}H NMR** (600 MHz, $CDCl_3$) δ 6.86 (s, 1H), 6.74 (t, J = 8.6 Hz, 2H), 6.52 (s, 1H), 6.31 (d, J = 8.3 Hz, 1H), 6.22 (d, J = 8.2 Hz, 1H), 4.83 (dt, J = 10.6, 7.8 Hz, 1H), 4.60 – 4.54 (m, 1H), 4.00 (dd, J = 11.5, 6.5 Hz, 1H), 3.96 – 3.91 (m, 1H), 3.82 (s, 3H),

3.80 (s, 3H), 3.14 (dd, J = 12.6, 6.4 Hz, 1H), 2.92 (t, J = 7.1 Hz, 2H), 2.36 – 2.26 (m, 2H), 2.07 – 2.00 (m, 1H). **^{13}C NMR** (151 MHz, $CDCl_3$) δ 168.4, 157.0, 153.8, 150.8, 135.1, 130.4, 129.3, 129.1, 117.7, 116.3, 115.7, 115.4, 113.5, 66.4, 65.0, 56.5, 55.8, 34.7, 29.6, 29.3. **HRMS** (ESI): m/z calculated for $C_{20}H_{22}NaO_5^+$ $[M + Na]^+$ 365.1359 found 365.1363. $[\alpha]_D^{25}$ = -86.2 (c = 0.28, CH_2Cl_2). **HPLC**: Chiralpak IF column, 80:20 hexanes/isopropanol, flow rate = 1.0 mL/min, λ = 254 nm, t_R = 10.20 min (minor), 11.78 min (major).

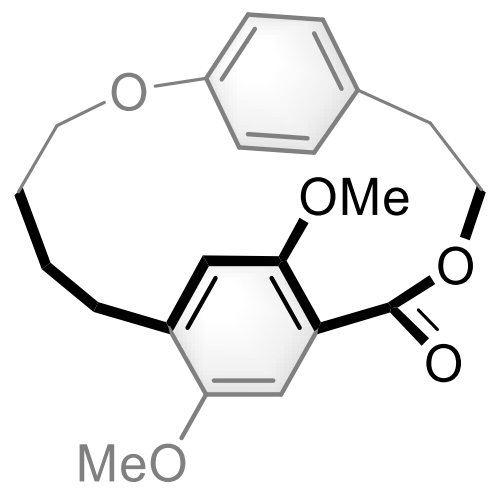

26, colorless solid, 21% yield, 7.5 mg, 94% *ee*. **^{1}H NMR** (600 MHz, $CDCl_3$) δ 6.88 (s, 1H), 6.87 (dd, J = 8.4, 2.3 Hz, 1H), 6.81 (dd, J = 8.4, 2.3 Hz, 1H), 6.50 (s, 1H), 6.43 (dd, J = 8.4, 2.8 Hz, 1H), 6.29 (dd, J = 8.4, 2.8 Hz, 1H), 4.70 (ddd, J = 11.2, 7.1, 5.6 Hz, 1H), 4.64 (dt, J = 11.2, 7.1 Hz, 1H), 3.91 – 3.86 (m, 1H), 3.83 – 3.79 (m, 1H), 3.79 (s, 3H), 3.73 (s, 3H), 3.05 (ddd, J = 13.0, 7.6, 3.2 Hz, 1H), 3.03 – 2.93 (m, 2H), 2.16 (ddd, J = 13.1, 10.0, 3.2 Hz, 1H), 1.88 – 1.79 (m, 1H), 1.65 – 1.57 (m, 1H), 1.49 – 1.41 (m, 1H), 1.41 – 1.34 (m, 1H). **^{13}C NMR** (151 MHz, $CDCl_3$) δ 166.8, 155.9, 153.8, 150.5, 137.2, 130.5, 130.0, 129.0, 117.3, 115.9, 115.7, 114.5, 113.9, 66.5, 63.7, 56.9, 55.7, 33.9, 30.0, 25.1, 24.1. **HRMS** (ESI): m/z calculated for $C_{21}H_{24}NaO_5^+$ $[M + Na]^+$ 379.1516 found 379,1518. $[\alpha]_D^{25}$ = -191.4 (c = 0.34, CH_2Cl_2). **HPLC**: Chiralpak IF column, 80:20 hexanes/isopropanol, flow rate = 1.0 mL/min, λ = 254 nm, t_R = 9.24 min (minor), 11.24 min (major).

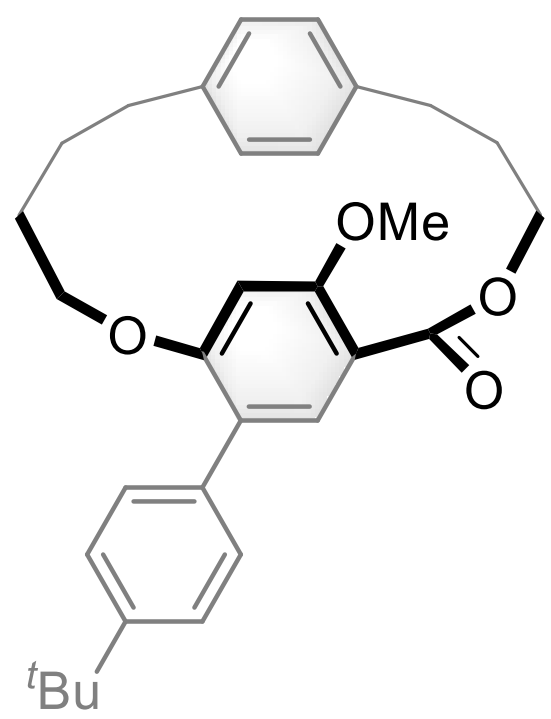

27, yellow oil, 57% yield, 26.9 mg, 97% *ee*. **^{1}H NMR** (600 MHz, $CDCl_3$) δ 7.44 – 7.39 (m, 5H), 6.95 (d, J = 7.7 Hz, 2H), 6.71 (d, J = 7.7 Hz, 2H), 6.37 (s, 1H), 4.52 – 4.47 (m, 1H), 4.46 – 4.41 (m, 1H), 4.29 (dt, J = 12.2, 4.2 Hz, 1H), 4.02 (ddd, J = 12.7, 10.1, 3.1 Hz, 1H), 3.78 (s, 3H), 2.78 – 2.70 (m, 2H), 2.46 (ddd, J = 13.7, 7.4, 2.7 Hz, 1H), 2.19 – 2.13 (m, 3H), 1.75 – 1.66 (m, 1H), 1.52 – 1.45 (m, 1H), 1.37 (s, 9H), 1.34 – 1.27 (m, 2H). **^{13}C NMR** (151 MHz, $CDCl_3$) δ 166.3, 158.6, 157.4, 149.7, 139.3, 138.6, 134.6, 133.6, 128.7, 128.4, 127.6, 125.2, 124.9, 113.8, 103.0, 68.3, 65.9, 56.4, 34.7, 34.2, 31.5, 28.3, 25.6, 25.4. **HRMS** (ESI): m/z calculated for $C_{31}H_{36}NaO_4^+$ $[M + Na]^+$ 495.2506 found 495.2508. $[\alpha]_D^{25}$ = -39.9 (c = 0.58, CH_2Cl_2). **HPLC**: Chiralpak IC column, 80:20 hexanes/isopropanol, flow rate = 1.0 mL/min, λ = 254 nm, t_R = 16.83 min (major), 27.49 min (minor).

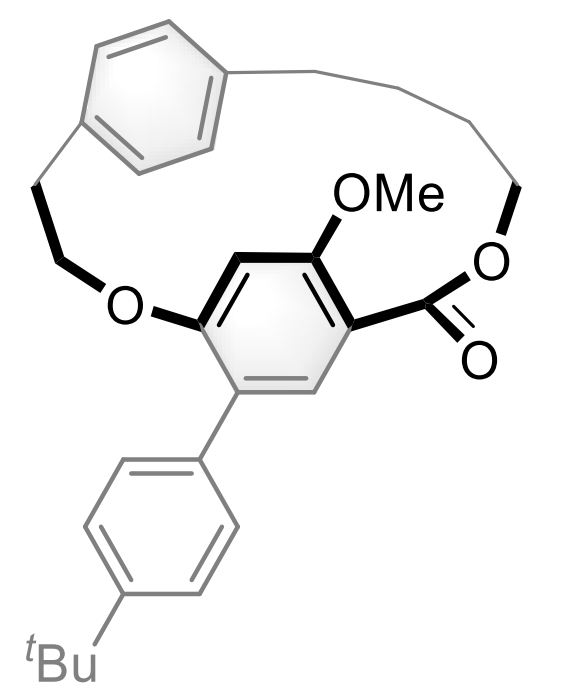

28, white solid, 59% yield, 27.0 mg, 97% *ee*. **^{1}H NMR** (400 MHz, $CDCl_3$) δ 7.47 (s, 4H), 7.43 (s, 1H), 6.94 – 6.87 (m, 2H), 6.73 (d, *J* = 7.8 Hz, 1H), 6.63 (d, *J* = 7.8 Hz, 1H), 6.37 (s, 1H), 4.59 (ddd, *J* = 12.0, 8.9, 2.6 Hz, 1H), 4.35 (ddd, *J* = 12.4, 5.6, 3.2 Hz, 1H), 4.19 (dt, *J* = 11.0, 5.6 Hz, 1H), 3.93 (ddd, *J* = 10.5, 7.2, 5.2 Hz, 1H), 3.76 (s, 3H), 2.87 – 2.72 (m, 2H), 2.53 (ddd, *J* = 14.2, 8.2, 2.7 Hz, 1H), 2.40 (ddd, *J* = 13.8, 8.1, 2.6 Hz, 1H), 1.82 – 1.60 (m, 4H), 1.39 (s, 9H). **^{13}C NMR** (101 MHz, $CDCl_3$) δ 168.1, 159.7, 157.8, 149.9, 140.7, 136.2, 134.6, 133.0, 129.1, 128.9, 128.5, 128.3, 128.1, 125.3, 124.8, 114.7, 103.1, 70.1, 66.4, 55.7, 37.9, 34.7, 34.5, 31.5, 28.7, 26.6. **HRMS** (ESI): m/z calculated for $C_{30}H_{34}NaO_4^+$ $[M + Na]^+$ 481.2349 found 481.2347. $[\alpha]_D^{25}$ = -183.1 (c = 0.38, CH_2Cl_2). **HPLC**: Chiralpak IC column, 80:20 hexanes/isopropanol, flow rate = 1.0 mL/min, λ = 254 nm, t_R = 17.89 min (major), 40.43 min (minor).

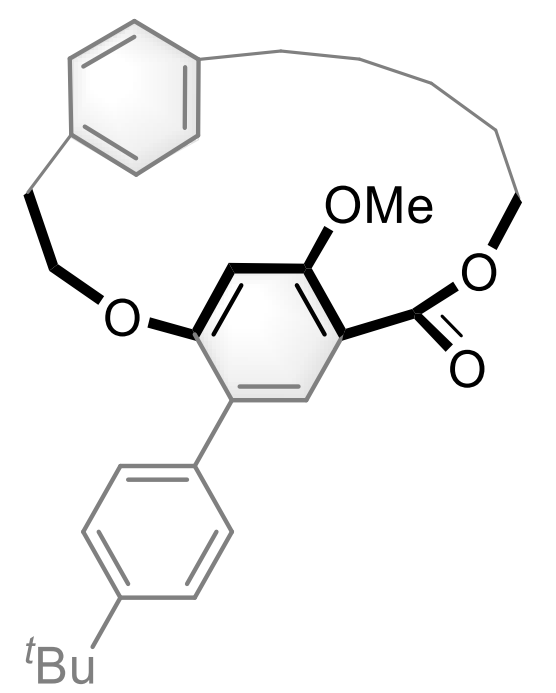

29, yellow solid, 51% yield, 24.2 mg, 94% *ee*. **^{1}H NMR** (600 MHz, $CDCl_3$) δ 7.63 (s, 1H), 7.47 – 7.42 (m, 4H), 6.76 (br, 4H), 6.32 (s, 1H), 4.58 (ddd, *J* = 12.3, 7.6, 4.4 Hz, 1H), 4.41 – 4.33 (m, 3H), 3.74 (s, 3H), 2.84 – 2.80 (m, 2H), 2.49 – 2.40 (m, 2H), 1.73 (p, *J* = 6.3 Hz, 2H), 1.59 – 1.44 (m, 2H), 1.39 (s, 9H), 1.35 – 1.27 (m, 2H). **^{13}C NMR** (151 MHz, $CDCl_3$) δ 166.5, 160.5, 159.4, 149.7, 140.4, 135.6, 134.8, 134.7, 129.0, 128.3, 125.2, 124.2, 113.1, 102.2, 69.8, 64.3, 55.9, 37.9, 35.2, 34.7, 31.6, 30.8, 28.4, 25.2. **HRMS** (ESI): m/z calculated for $C_{31}H_{36}NaO_4^+$ $[M + Na]^+$ 495.2506 found 495.2509. $[\alpha]_D^{25}$ = -156.3 (c = 1.12, CH_2Cl_2). **HPLC**: Chiralpak IC column, 80:20 hexanes/isopropanol, flow rate = 1.0 mL/min, λ = 254 nm, t_R = 12.81 min (major), 21.37 min (minor).

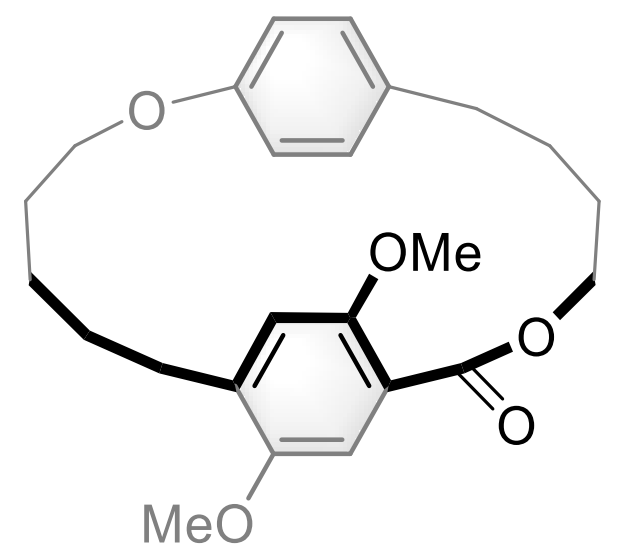

32, yellow oil, 45% yield, 17.9 mg, 0% *ee*. **^{1}H NMR** (600 MHz, $CDCl_3$) δ 6.97 (s, 1H), 6.94 (d, *J* =

8.6 Hz, 2H), 6.67 (s, 1H), 6.44 (d, J = 8.6 Hz, 2H), 4.56 – 4.49 (m, 1H), 4.04 (ddd, J = 10.7, 6.3, 3.8 Hz, 1H), 3.82 (s, 3H), 3.77 (s, 3H), 3.76 – 3.72 (m, 1H), 3.72 – 3.67 (m, 1H), 3.09 (ddd, J = 13.0, 7.8, 3.2 Hz, 1H), 2.59 (ddd, J = 14.1, 8.5, 2.2 Hz, 1H), 2.46 – 2.38 (m, 1H), 2.25 (ddd, J = 13.0, 9.5, 3.3 Hz, 1H), 2.01 – 1.92 (m, 1H), 1.88 – 1.80 (m, 1H), 1.78 – 1.71 (m, 2H), 1.67 – 1.59 (m, 1H), 1.49 – 1.42 (m, 1H), 1.41 – 1.26 (m, 4H). **^{13}C NMR** (151 MHz, $CDCl_3$) δ 165.4, 156.8, 153.9, 150.8, 137.1, 134.3, 129.4, 117.3, 115.5, 114.0, 113.6, 66.3, 63.9, 56.8, 55.9, 33.7, 30.6, 28.6, 28.1, 27.4, 27.3, 23.0. **HRMS** (ESI): m/z calculated for $C_{24}H_{30}NaO_4^+$ $[M + Na]^+$ 421.1985 found 421.1987. **HPLC**: Chiralpak IF column, 80:20 hexanes/isopropanol, flow rate = 1.0 mL/min, λ = 254 nm, t_R = 8.89 min, 10.20 min.

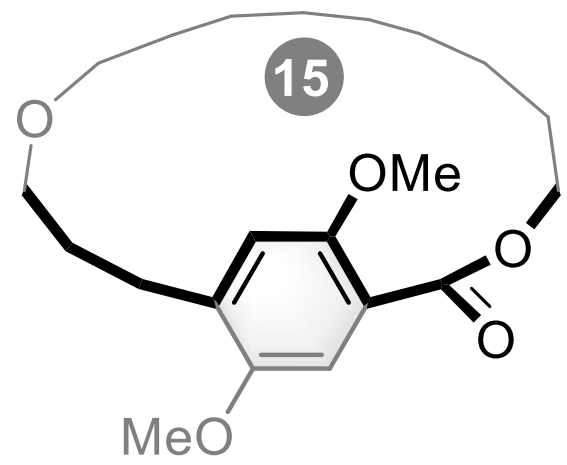

33, colorless oil. **^{1}H NMR** (400 MHz, $CDCl_3$). δ 7.32 (s, 1H), 6.81 (s, 1H), 4.55 – 4.47 (m, 1H), 4.30 – 4.22 (m, 1H), 3.86 (s, 3H), 3.82 (s, 3H), 3.47 – 3.33 (m, 2H), 3.13 (ddd, J = 13.6, 8.0, 3.7 Hz, 1H), 2.98 (t, J = 6.3 Hz, 2H), 2.38 (ddd, J = 13.2, 9.2, 3.7 Hz, 1H), 2.13 – 2.03 (m, 1H), 1.87 – 1.67 (m, 3H), 1.53 – 1.42 (m, 2H), 1.34 – 1.27 (m, 2H), 1.20 – 1.03 (m, 6H), 0.86 – 0.72 (m, 2H). **^{13}C NMR** (101 MHz, $CDCl_3$) δ 166.3, 153.7, 151.4, 137.5, 117.4, 115.7, 113.0, 71.1, 70.6, 65.6, 56.8, 55.9, 30.6, 29.9, 28.7, 28.5, 28.4, 27.2, 27.0, 25.7.

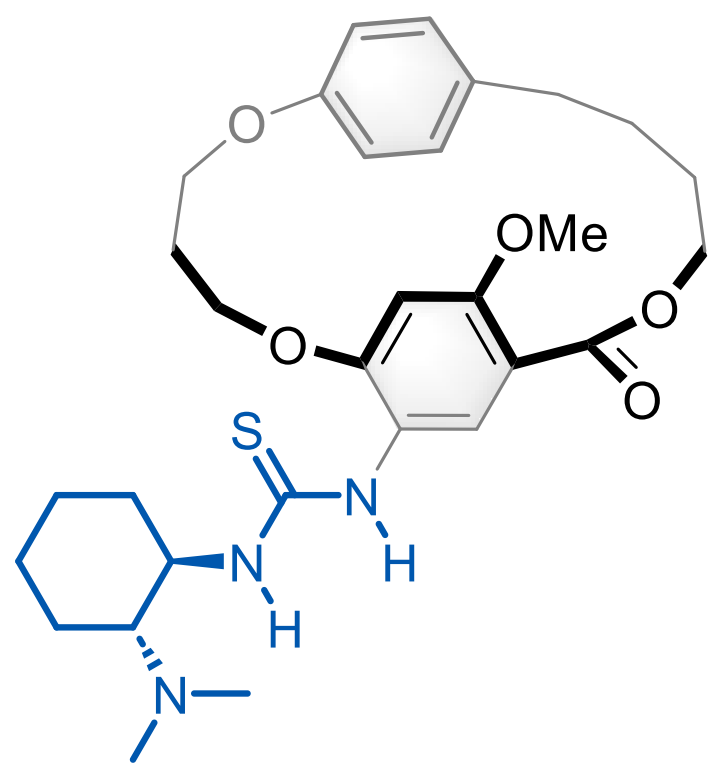

34, yellow oil, 49% yield (4 steps), 136 mg, >20:1 *dr*. **^{1}H NMR** (400 MHz, $CDCl_3$) δ 7.48 (s, 1H), 6.85 (d, J = 8.0 Hz, 2H), 6.53 (s, 1H), 6.39 (d, J = 8.2 Hz, 2H), 4.71 (t, J = 11.5 Hz, 1H), 4.46 (dt, J = 12.0, 3.8 Hz, 1H), 4.34 – 4.25 (m, 1H), 4.11 – 4.02 (m, 1H), 3.96 (s, 1H), 3.79 (s, 3H), 3.64 (t, J = 10.1 Hz, 1H), 2.71 (d, J = 12.4 Hz, 1H), 2.42 – 2.37 (m, 2H), 2.26 (s, 6H), 2.10 – 2.01 (m, 1H), 1.89 – 1.79 (m, 2H), 1.75 – 1.69 (m, 4H), 1.42 – 1.31 (m, 2H), 1.29 – 1.02 (m, 8H). **^{13}C NMR** (101 MHz, $CDCl_3$) δ 179.8, 165.0, 158.6, 156.7, 156.0, 135.3, 129.5, 129.0, 113.2, 112.6, 100.4, 67.2, 67.0, 64.7, 62.4, 56.3, 56.2, 39.8, 34.7, 32.7, 30.8, 29.8, 28.7, 27.9, 25.2, 24.7, 21.9. **HRMS** (ESI): m/z calculated for $C_{30}H_{42}N_3O_5S^+$ $[M + H]^+$ 556.2840 found 556.2847. $[\alpha]_D^{25}$ = -7.0 (c = 0.76, CH_2Cl_2).

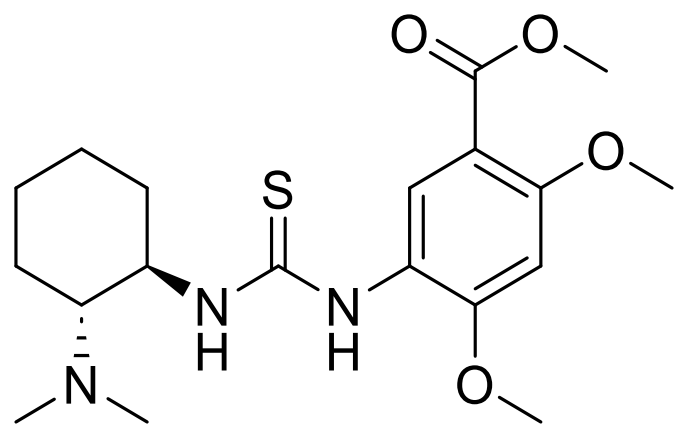

35, yellow oil, 51% yield (4 steps), 101 mg. **^{1}H NMR** (600 MHz, $CDCl_3$) δ 7.99 (s, 1H), 6.48 (s, 1H), 4.00 (s, 1H), 3.92 (s, 3H), 3.88 (s, 3H), 3.82 (s, 3H), 2.72 – 2.52 (m, 2H), 2.33 (s, 6H), 1.86 (dd, J = 27.6, 12.4 Hz, 2H), 1.69 (d, J = 13.8 Hz, 1H), 1.36 – 1.31 (m, 1H), 1.27 – 1.20 (m, 4H), 1.09 (q, J = 12.6 Hz, 1H). **^{13}C NMR** (151 MHz, $CDCl_3$) δ 181.0, 165.5, 159.7, 157.5, 130.1, 111.4, 96.4, 66.9, 56.6, 56.1, 55.9, 51.9, 39.8, 32.7, 29.8, 25.1, 24.6, 22.0. **HRMS** (ESI): m/z calculated for $C_{19}H_{30}N_3O_4S^+$ $[M + H]^+$ 396.1952 found 396.1956. $[\alpha]_D^{25}$ = -18.1 (c = 1.37, CH_2Cl_2).

6. References

[1] H. Ulrich, D. V. Rao, B. Tucker and A. A. R. Sayigh, *J. Org. Chem.*, 1974, **39**, 2437–2438.

[2] C. Q. Meng, L. Ni, K. J. Worsencroft, Z. Ye, M. D. Weingarten, J. E. Simpson, J. W. Skudlarek, E. M. Marino, K.-L. Suen, C. Kunsch, A. Souder, R. B. Howard, C. L. Sundell, M. A. Wasserman and J. A. Sikorski, *J. Med. Chem.*, 2007, **50**, 1304–1315.

[3] X. Lv, F. Su, H. Long, F. Lu, Y. Zeng, M. Liao, F. Che, X. Wu and Y. R. Chi, *Nat. Commun.*, 2024, **15**, 958.

[4] H.-H. Chen, J.-T. Jiang, Y.-N. Yang, L.-W. Ye and B. Zhou, *Angew. Chem. Int. Ed.*, 2025, **64**, e202505167.

[5] J. Y. Mang, D. Kwon and D. Y. Kim, *Bull. Korean Chem. Soc.*, 2009, **30**, 249–252.

7. X-Ray report

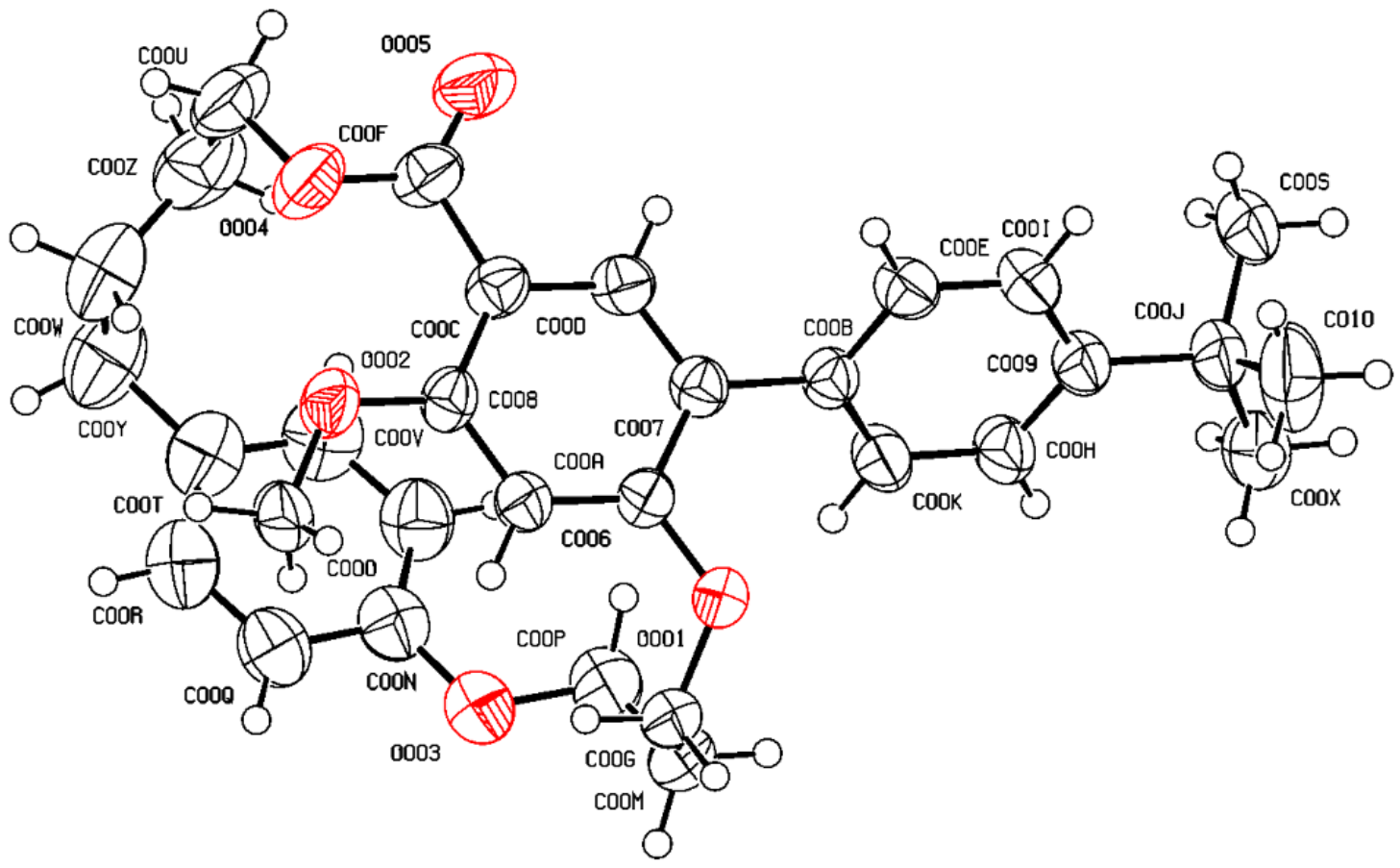

Scheme S6. Thermal ellipsoid plot for the crystal structure of 13 (at 50% probability level, CCDC No. 2540611)

Experimental

Single crystals of $C_{31}H_{36}O_5$ (**13**) were recrystallised from ethyl acetate (EA), petroleum ether (PE), diethyl ether mounted in inert oil and transferred to the cold gas stream of the diffractometer. The crystal was kept at 100.0 K during data collection.

Table S8. Crystal data and structure refinement for 13.

Identification code	**13**
Empirical formula	$C_{31}H_{36}O_5$
Formula weight	488.60
Temperature/K	100.00(10)
Crystal system	Monoclinic
Space group	$P2_1$
a/Å	11.86500(10)
b/Å	9.16130(10)
c/Å	12.48520(10)
α/°	90
β/°	95.6340(10)
γ/°	90
Volume/Å^3	1350.57(2)
Z	2
ρ_{calc}g/cm^3	1.201
μ/mm^{-1}	0.641
F(000)	524.0
Crystal size/mm^3	0.2 × 0.1 × 0.05
Radiation	Cu Kα (λ = 1.54184)
2Θ range for data collection/°	7.114 to 152.158
Index ranges	$-14 \leq h \leq 14$, $-10 \leq k \leq 10$, $-15 \leq l \leq 15$
Reflections collected	20206
Independent reflections	5311 [R_{int} = 0.0246, R_{sigma} = 0.0214]
Data/restraints/parameters	5311/1/330
Goodness-of-fit on F^2	1.058
Final R indexes [I>=2σ (I)]	R_1 = 0.0336, wR_2 = 0.0940
Final R indexes [all data]	R_1 = 0.0360, wR_2 = 0.0959
Largest diff. peak/hole / e Å^{-3}	0.25/-0.12
Flack parameter	-0.11(6)

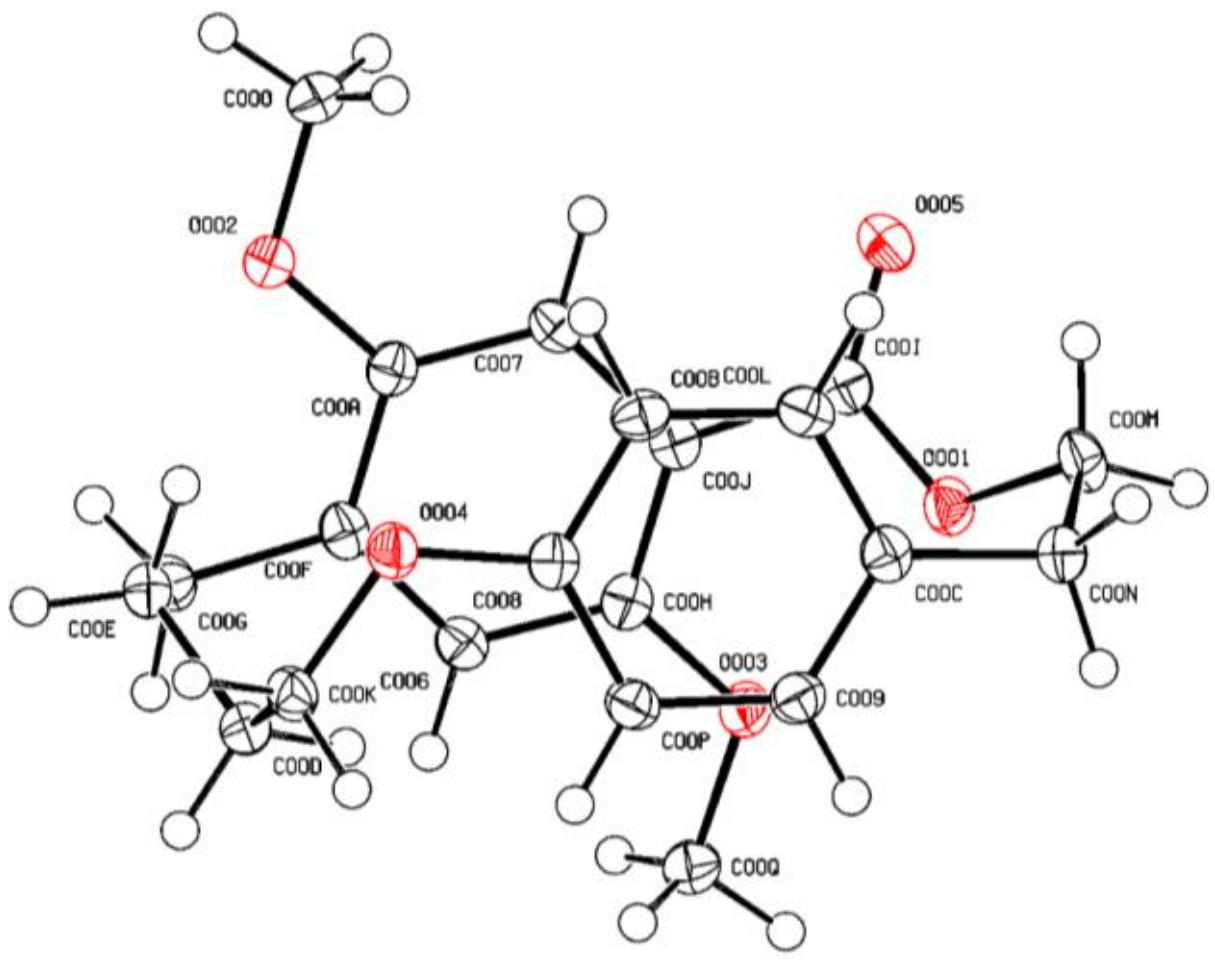

Scheme S7. Thermal ellipsoid plot for the crystal structure of 26 (at 50% probability level, CCDC No. 2540659

Experimental

Single crystals of $C_{21}H_{24}O_5$ (**26**) were recrystallised from *n*-hexane and ethyl acetate (EA) mounted in inert oil and transferred to the cold gas stream of the diffractometer. The crystal was kept at 100.0 K during data collection.

Table S9. Crystal data and structure refinement for 26.

Identification code	**26**
Empirical formula	$C_{21}H_{24}O_5$
Formula weight	356.40
Temperature/K	100.00(10)
Crystal system	Monoclinic
Space group	$P2_1$
a/Å	8.28910(10)
b/Å	10.60680(10)
c/Å	10.3518(2)
α/°	90
β/°	101.5840(10)
γ/°	90
Volume/Å^3	891.60(2)
Z	2
ρ_{calc}g/cm^3	1.328
μ/mm^{-1}	0.768
F(000)	380.0
Crystal size/mm^3	0.25 × 0.1 × 0.1
Radiation	Cu Kα (λ = 1.54184)
2Θ range for data collection/°	8.72 to 151.724
Index ranges	$-10 \le h \le 10$, $-13 \le k \le 13$, $-12 \le l \le 12$
Reflections collected	13848
Independent reflections	3515 [R_{int} = 0.0554 R_{sigma} = 0.0333]
Data/restraints/parameters	3515/1/238
Goodness-of-fit on F^2	1.057
Final R indexes [I>=2σ (I)]	R_1 = 0.0281, wR_2 = 0.0737
Final R indexes [all data]	R_1 = 0.0412, wR_2 = 0.0808
Largest diff. peak/hole / e Å^{-3}	0.32/-0.29
Flack parameter	0.04(8)

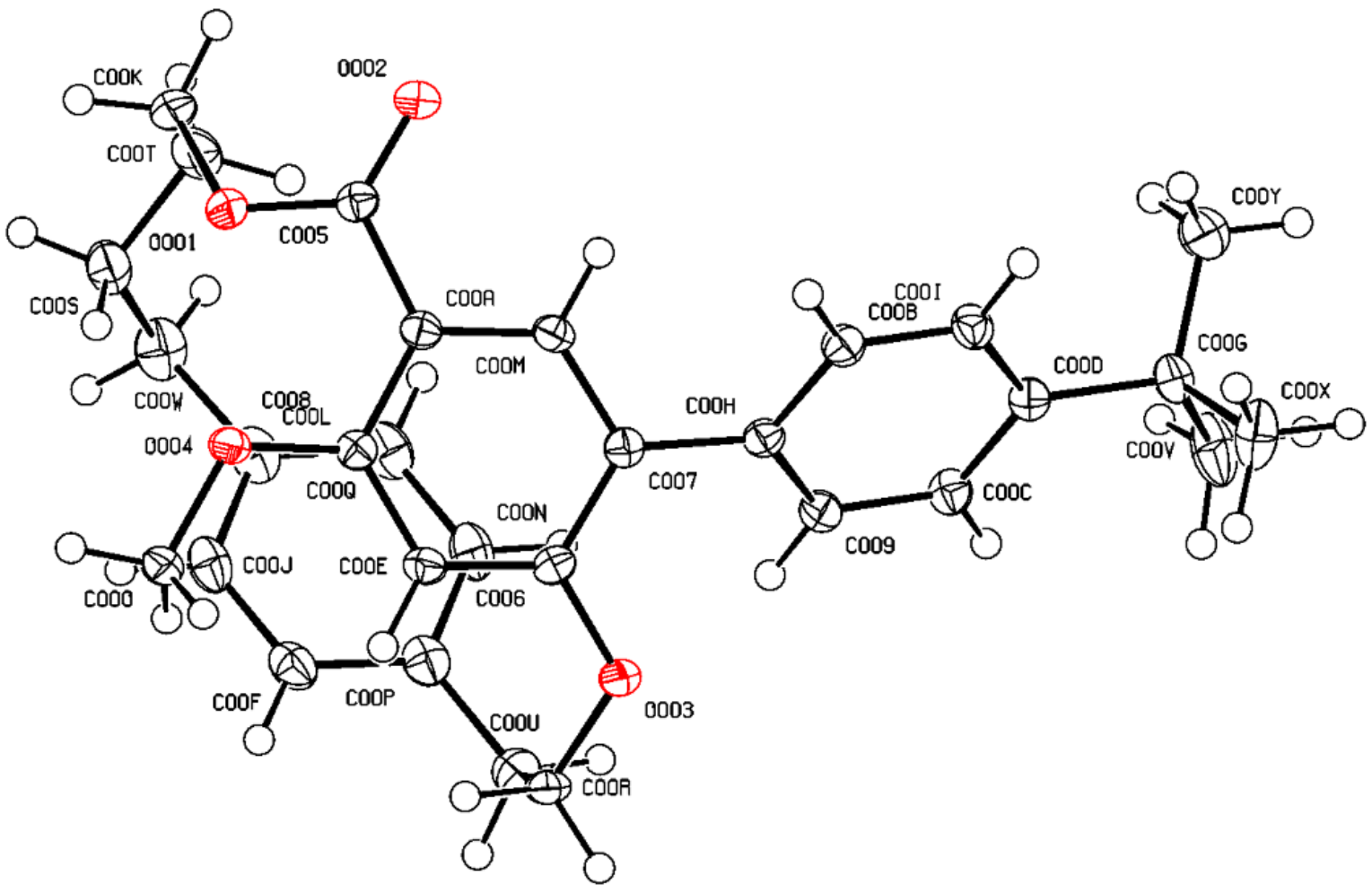

Scheme S8. Thermal ellipsoid plot for the crystal structure of 28 (at 50% probability level, CCDC No. 2540648)

Experimental

Single crystals of $C_{30}H_{34}O_4$ (**28**) were recrystallised from ethyl acetate (EA), petroleum ether (PE), diethyl ether mounted in inert oil and transferred to the cold gas stream of the diffractometer. The crystal was kept at 100.0 K during data collection.

Table S10. Crystal data and structure refinement for 28.

Identification code	**28**
Empirical formula	$C_{30}H_{34}O_4$
Formula weight	458.57
Temperature/K	100.00(10)
Crystal system	Monoclinic
Space group	$P2_1$
a/Å	8.14900(10)
b/Å	15.3664(2)
c/Å	10.24360(10)
α/°	90
β/°	100.5640(10)
γ/°	90
Volume/Å^3	1260.97(3)
Z	2
ρ_{calc}g/cm^3	1.208
μ/mm^{-1}	0.624
F(000)	492.0
Crystal size/mm^3	0.15 × 0.15 × 0.05
Radiation	Cu Kα (λ = 1.54184)
2Θ range for data collection/°	8.782 to 151.342
Index ranges	$-10 \leq h \leq 10$, $-18 \leq k \leq 19$, $-12 \leq l \leq 12$
Reflections collected	16148
Independent reflections	4219[R_{int} = 0.0548, R_{sigma} = 0.0323]
Data/restraints/parameters	4219/1/312
Goodness-of-fit on F^2	1.086
Final R indexes [I>=2σ (I)]	R_1 = 0.0311, wR_2 = 0.0748
Final R indexes [all data]	R_1 = 0.0447, wR_2 = 0.0821
Largest diff. peak/hole / e Å^{-3}	0.30/-0.31
Flack parameter	0.01(8)

8. DFT simulations computational methods

1) Computational methods

We calculated the ring strain energy as the negative enthalpic change based on a homodesmotic reaction.[1,2] In a homodesmotic reaction, both the number of carbon-carbon bond types and the number of each type of carbon atom (sp, sp^2, sp^3) with zero, one, two and three hydrogens are conversed between reactants and products such that the cancellation of bond-type and hybridization effects is guaranteed. As shown in Scheme S9, we cleaved each C-C bond of the -CH_2-CH2- backbone and capped the dangling bonds with methyl groups.

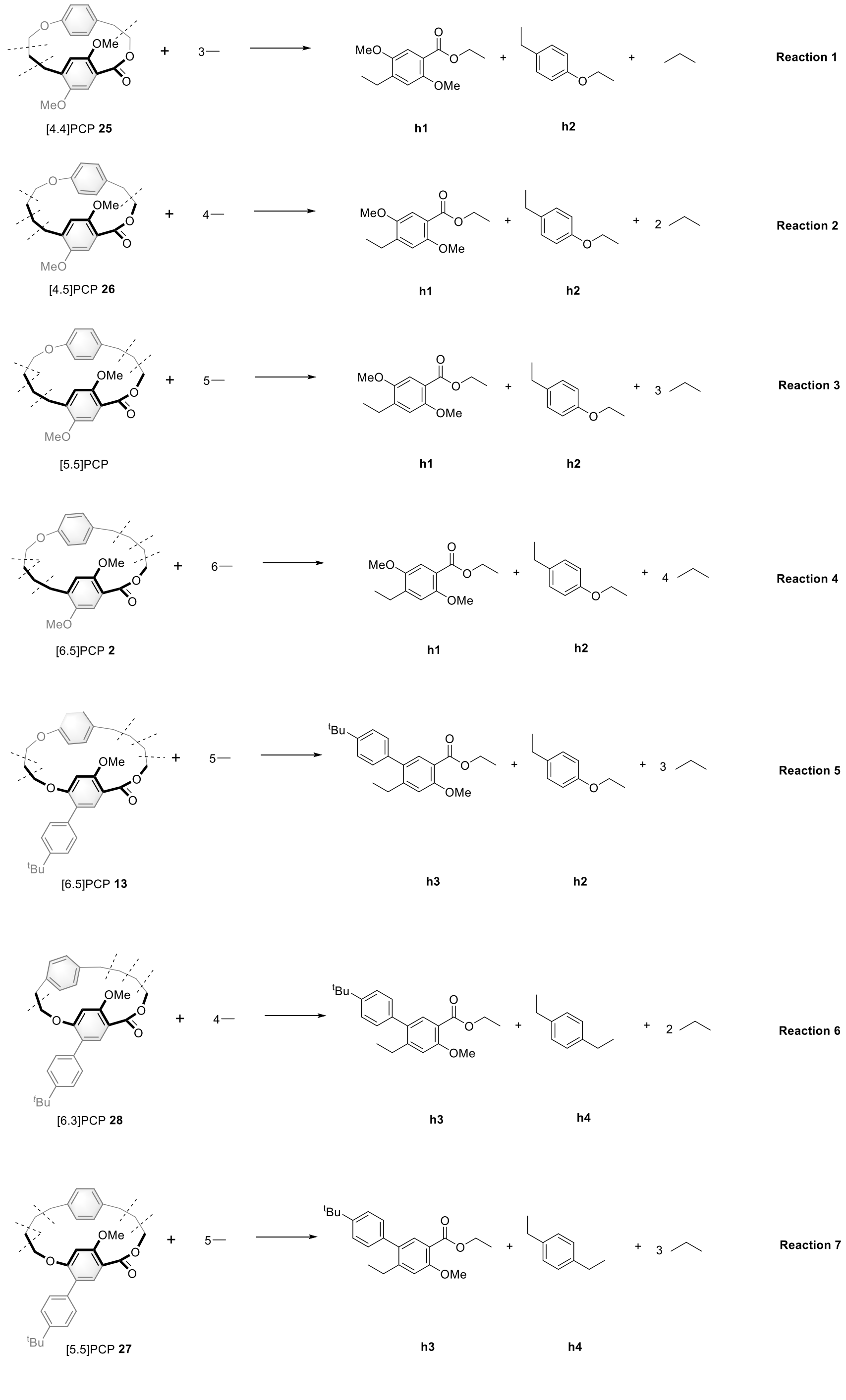

Scheme S9. Homodesmotic reactions used to compute the strain energy of paracyclophanes.

All molecular geometries were optimized using M06-2X/6-31G(d)[3] with the polarizable continuum model (PCM)[4] to account for tetrahydrofuran (THF) solvation. For the macrocyclic molecules, the initial geometries were obtained via a systematic conformational search in THF using the iterative metadynamics and genetic crossing workflow[5] with the GFN2 extended tight binding (xTB) method[6] and the analytical linearized Poisson-Boltzmann solvation model.[7] All structures in the low-energy conformational space were subsequently optimized using M06-2X/6-31G(d)/PCM(THF) and the conformer with the lowest electronic energy was taken as the optimized geometry. For the simple acyclic molecules, the lowest-energy GFN2-xTB conformer was used as the initial structure for DFT geometry optimization. All DFT optimized geometries were characterized as minima by the harmonic vibrational frequency analysis. The single-point energy was computed at the B2PLYP-D3(BJ)/def2-TZVP[8-10] level of theory with the conductor-like PCM[11] for THF solvation. The final enthalpy at 298.15 K was evaluated as

$$H_{298\mathrm{K}} = E_{\mathrm{elec}}(\text{B2PLYP-D3}) + E_{\mathrm{ZPVE}}(\text{M06-2X}) + \Delta H_{298\mathrm{K}}(\text{M06-2X}),$$

where E_{elec}, E_{ZPVE}, and $\Delta H_{298\mathrm{K}}$ correspond to the electronic single-point energy, the zero-point vibrational energy (ZPVE) and the thermal correction, respectively. The ring strain energy (RSE) was calculated as the negative enthalpic change of the reaction $\mathrm{RSE}_{298\mathrm{K}} = H_{298\mathrm{K}}(\mathrm{reactants}) - H_{298\mathrm{K}}(\mathrm{products})$.

We employed CREST 3.0[12] for the conformer sampling and PCA clustering. Geometry optimizations and vibrational frequencies at the M06-2X/6-31G(d) level of theory were performed using Gaussian 16,[13] while the B2PLYP-D3(BJ)/def2-TZVP single-point energies were obtained using ORCA 6.0.[14] For the latter, the RIJCOSX approximation[15] was adopted together with the def2/J and the def2-TZVP/C auxiliary basis sets.[10,16]

To visualize the local strain of paracyclophanes, we employed the StrainVis analysis[17] at the M06-2X/6-31G(d)/PCM(THF) level of theory. We characterized the aromaticity of benzene rings in paracyclophanes using the nucleus-independent chemical shifts (NICS) index at the B3LYP/6-311++G(d,p)[18,19] level of theory. Specifically, we employed the easily accessible $\mathrm{NICS}(1)_{zz}$ approach[20-22], defined as the negative zz component of the magnetic shielding tensor for a ghost atom placed 1 Å over the benzene ring center (the centroid of the six carbons) outside the macrocycle. The magnetic shielding tensor and the $\mathrm{NICS}(1)_{zz}$ values were obtained using Gaussian 16 and Multiwfn 3.8,[23] respectively.

The noncovalent interaction was visualized using the method of independent gradient model based on Hirshfeld partition[24] (IGMH) using Multiwfn 3.8.[23] The magnetically induced current density (MICD) analysis was performed using the gauge-including magnetically induced current (GIMIC) method.[25,26] All data below is presented with the corresponding structures visualized using CYLview.[27]

2) Strain energy of paracyclophanes

Table S11. Ring strain energy (in kcal mol^{-1}) of paracyclophanes.[a]

Molecule	Eelec(M06-2X)	ΔEelec(B2PLYP-D3)	ΔEZPVE	ΔΔH298K	RSE298K
[4.4]PCP **25**	7.0	0.1	-1.2	-0.4	5.6
[5.5]PCP	-1.7	1.0	-0.9	-0.2	-1.9
[4.5]PCP **26**	3.9	0.7	-0.8	-0.4	3.4
[6.5]PCP **2**	-1.0	0.4	-0.7	-0.1	-1.4

Molecule	Eelec(M06-2X)	ΔEelec(B2PLYP-D3)	ΔEZPVE	ΔΔH298K	RSE298K
[6.5]PCP **13**	0.5	1.1	-0.9	-0.2	0.6
[6.3]PCP **28**	4.9	-0.3	-1.3	-0.3	3.0
[5.5]PCP **27**	1.3	0.4	-1.0	-0.2	0.4
[12]PCP	3.5	0.8	-1.6	0.5	3.2
[14]PCP	-0.3	1.2	-1.4	0.7	0.1

[a] E_{elec}(M06-2X): the RSE based on M06-2X/6-31G(d) electronic energy, ΔE_{elec}(B2PLYP-D3): the energy correction due to B2PLYP-D3(BJ)/def2-TZVP, ΔE_{ZPVE}: the energy correction due to ZPVE, $\Delta\Delta H_{298K}$: the energy correction due to thermal energy at 298.15K.

Table S11 presents the strain energies of the cyclophanes with incremental contributions from electronic energies, zero-point vibrational energies (ZPVE), and thermal corrections. Focusing on the regular series (**26**, **25**, and **2**), the strain energy generally decreases with increasing macrocycle size. However, molecule **2** displays a negative RSE value, suggesting that the conventional ring strain is offset by other factors. We attribute the observed RSE to two competing factors: the structural deformation of the C/O macrocyclic backbone and the non-covalent aromatic interactions between the two benzene moieties.

To isolate the aromatic interaction, we considered a model system obtained by removing the carbon backbone connecting the two substituted benzenes. The dangling bonds were then capped by hydrogen atoms, which were optimized using M06-2X/6-31G(d)/PCM(THF) while freezing all other atoms. The aromatic interaction energy (AIE) was calculated as the energy difference between the hydrogen-optimized structure and the non-interacting system. The latter was obtained by elongating the distance between the two benzene centers to 20 Å with all other coordinates frozen. Table S12 lists the AIEs for the model dimers derived from the cyclophanes. All systems exhibited negative AIE values, indicating that non-covalent interactions between the two benzene moieties serve as a significant stabilizing factor for the macrocyclic configurations.

Table S12. The aromatic interaction energy (in kcal mol^{-1}) for the model dimers.[a]

	Reactant[b]		
System	d	φ	AIE
[4.4]PCP **25**	3.489	2.1	-6.0
[4.5]PCP **26**	3.588	4.4	-6.1
[5.5]PCP	3.591	2.1	-7.6
[6.5]PCP **2**	3.784	7.6	-6.8
[6.5]PCP **13**	3.779	8.6	-7.0
[6.3]PCP **28**	3.590	15.8	-5.5
[5.5]PCP **27**	3.646	5.0	-5.8

[a] d: the distance (in Å) between two benzene centers (centroid of six carbons), φ: the angle (in degrees) between the average planes of two benzene rings defined using all six carbon atoms.

3) StrainViz decomposition analysis

To complement the StrainViz maps discussed in the main text, the bond-, angle-, and dihedral-force contributions were compared at representative local connections in six paracyclophanes. The total force is the sum of the three components. Table S13 summarizes the position with the largest total force in each molecule, and Tables S14-S19 provide the corresponding position-wise decompositions.

Table S13. Summary of the StrainViz decomposition at the position with the largest total force.

Molecule	Position	Total force	Bond share (%)	Angle share (%)	Dihedral share (%)	Dominant component
[4.4]PCP **25**	O9C10	0.005184	1.3	23.2	75.4	Dihedral
[4.5]PCP **26**	O9C10	0.005404	0.9	21.3	77.8	Dihedral
[6.5]PCP **2**	O9C10	0.003536	2.2	52.3	45.5	Angle
[6.5]PCP **13**	O9C10	0.003564	2.0	48.7	49.2	Dihedral
[6.3]PCP **28**	C21O22	0.004871	1.1	73.6	25.3	Angle
[5.5]PCP **27**	C4O23	0.003698	1.1	41.8	57.0	Dihedral

Position labels [i, j] denote the bond or local connection to which StrainViz maps the contribution. For torsional strain, the four-atom dihedral contribution is distributed over the three connecting bonds; therefore, [i, j] is a projected location label rather than a complete dihedral definition.

Table S14. Position-wise bond, angle, and dihedral contributions for [4.4]PCP **25**.

Position	Bond total	Angle total	Dihedral total	Total force	Bond share (%)	Angle share (%)	Dihedral share (%)	Dominant
O9C10	0.000070	0.001204	0.003910	0.005184	1.3	23.2	75.4	Dihedral
C10C11	0.000072	0.001720	0.002526	0.004319	1.7	39.8	58.5	Dihedral
C7O9	0.000032	0.000339	0.003130	0.003500	0.9	9.7	89.4	Dihedral
C7O8	0.000028	0.000086	0.001251	0.001364	2.0	6.3	91.7	Dihedral

Table S15. Position-wise bond, angle, and dihedral contributions for [4.5]PCP **26**.

Position	Bond total	Angle total	Dihedral total	Total force	Bond share (%)	Angle share (%)	Dihedral share (%)	Dominant
O9C10	0.000048	0.001153	0.004203	0.005404	0.9	21.3	77.8	Dihedral
C10C11	0.000070	0.001776	0.003181	0.005027	1.4	35.3	63.3	Dihedral
C7O9	0.000026	0.000451	0.003446	0.003923	0.7	11.5	87.8	Dihedral
C7O8	0.000025	0.000127	0.000915	0.001067	2.3	11.9	85.8	Dihedral

Table S16. Position-wise bond, angle, and dihedral contributions for [6.5]PCP **2**.

Position	Bond total	Angle total	Dihedral total	Total force	Bond share (%)	Angle share (%)	Dihedral share (%)	Dominant
O9C10	0.000077	0.001848	0.001611	0.003536	2.2	52.3	45.5	Angle
C22C23	0.000023	0.002046	0.001165	0.003234	0.7	63.3	36.0	Angle
C10C11	0.000034	0.001470	0.001672	0.003176	1.1	46.3	52.7	Dihedral
C21C22	0.000061	0.002192	0.000904	0.003158	1.9	69.4	28.6	Angle
C11C12	0.000037	0.000414	0.002592	0.003043	1.2	13.6	85.2	Dihedral
C7O9	0.000023	0.000600	0.002003	0.002626	0.9	22.8	76.3	Dihedral
C7O8	0.000021	0.000099	0.000396	0.000516	4.1	19.1	76.8	Dihedral

Table S17. Position-wise bond, angle, and dihedral contributions for [6.5]PCP **13**.

Position	Bond total	Angle total	Dihedral total	Total force	Bond share (%)	Angle share (%)	Dihedral share (%)	Dominant
O9C10	0.000072	0.001736	0.001755	0.003564	2.0	48.7	49.2	Dihedral
C4O24	0.000081	0.001734	0.001661	0.003476	2.3	49.9	47.8	Angle
C23O24	0.000051	0.001853	0.001546	0.003450	1.5	53.7	44.8	Angle

Position	Bond total	Angle total	Dihedral total	Total force	Bond share (%)	Angle share (%)	Dihedral share (%)	Dominant
C12C13	0.000048	0.001815	0.001537	0.003400	1.4	53.4	45.2	Angle
C10C11	0.000020	0.001354	0.001703	0.003077	0.7	44.0	55.3	Dihedral
C22C23	0.000024	0.001844	0.001179	0.003047	0.8	60.5	38.7	Angle
C7O9	0.000025	0.000601	0.002265	0.002891	0.9	20.8	78.4	Dihedral
C13C14	0.000071	0.001757	0.000937	0.002765	2.6	63.5	33.9	Angle
C7O8	0.000024	0.000103	0.000445	0.000573	4.3	18.0	77.7	Dihedral

Table S18. Position-wise bond, angle, and dihedral contributions for [6.3]PCP **28**.

Position	Bond total	Angle total	Dihedral total	Total force	Bond share (%)	Angle share (%)	Dihedral share (%)	Dominant
C21O22	0.000052	0.003585	0.001234	0.004871	1.1	73.6	25.3	Angle
C4O22	0.000035	0.003325	0.001491	0.004851	0.7	68.5	30.7	Angle
O9C10	0.000042	0.001488	0.002429	0.003958	1.1	37.6	61.4	Dihedral
C7O9	0.000035	0.000298	0.002984	0.003317	1.1	9.0	90.0	Dihedral

Table S19. Position-wise bond, angle, and dihedral contributions for [5.5]PCP **27**.

Position	Bond total	Angle total	Dihedral total	Total force	Bond share (%)	Angle share (%)	Dihedral share (%)	Dominant
C4O23	0.000042	0.001546	0.002109	0.003698	1.1	41.8	57.0	Dihedral
O9C10	0.000066	0.001654	0.001940	0.003659	1.8	45.2	53.0	Dihedral
C22O23	0.000042	0.001833	0.001670	0.003545	1.2	51.7	47.1	Angle
C20C21	0.000043	0.001549	0.001605	0.003197	1.3	48.5	50.2	Dihedral
C21C22	0.000026	0.001768	0.001123	0.002917	0.9	60.6	38.5	Angle
C10C11	0.000022	0.001807	0.000798	0.002628	0.8	68.8	30.4	Angle
C7O9	0.000043	0.000476	0.001929	0.002448	1.7	19.4	78.8	Dihedral
C7O8	0.000010	0.000106	0.000543	0.000659	1.4	16.1	82.5	Dihedral

Scheme S10. Structural data of representative molecules and their StrainViz fragments with greater contributions.

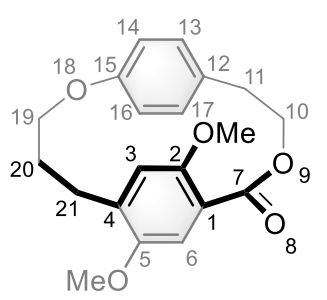

[4.4]PCP **25**

bond angles (°)		dihedral angles (°)	
C11–C10–O9	108.4	C1–C7–O9–C10	144.2
C20–C19–O18	108.2	C7–O9–C10–C11	104.1
C7–O9–C10	116.1	O9–C10–C11–C12	41.6
C19–O18–C15	121.7		

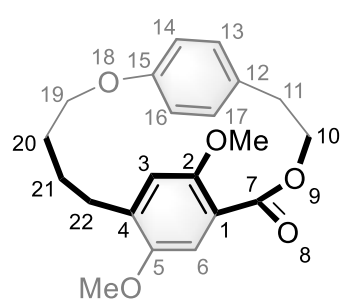

[4.5]PCP **26**

bond angles (°)		dihedral angles (°)	
C11–C10–O9	108.6	C1–C7–O9–C10	146.1
C20–C19–O18	108.5	C7–O9–C10–C11	104.3
C7–O9–C10	115.8	O9–C10–C11–C12	38.2
C19–O18–C15	117.5		

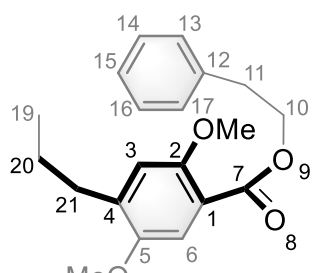

[4.4]PCP **25-r7**

bond angles (°)		dihedral angles (°)	
C11–C10–O9	108.0	C1–C7–O9–C10	153.5
C20–C19–O18	-	C7–O9–C10–C11	102.3
C7–O9–C10	116.5	O9–C10–C11–C12	46.8
C19–O18–C15	-		

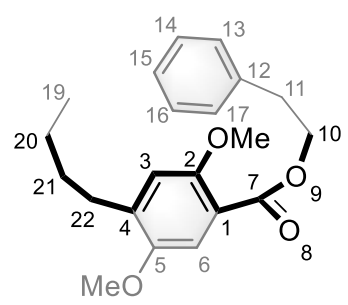

[4.5]PCP **26-r7**

bond angles (°)		dihedral angles (°)	
C11–C10–O9	108.8	C1–C7–O9–C10	168.3
C20–C19–O18	-	C7–O9–C10–C11	99.5
C7–O9–C10	117.5	O9–C10–C11–C12	60.2
C19–O18–C15	-		

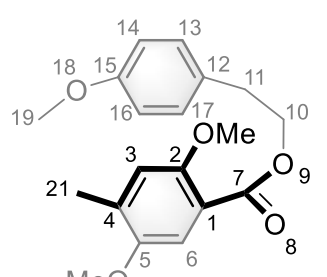

[4.4]PCP **25-r9**

bond angles (°)		dihedral angles (°)	
C11–C10–O9	108.2	C1–C7–O9–C10	155.7
C20–C19–O18	-	C7–O9–C10–C11	98.4
C7–O9–C10	116.4	O9–C10–C11–C12	50.4
C19–O18–C15	117.3		

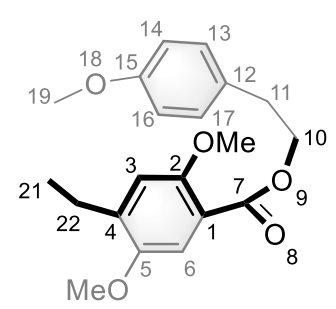

[4.5]PCP **26-r9**

bond angles (°)		dihedral angles (°)	
C11–C10–O9	108.9	C1–C7–O9–C10	169.4
C20–C19–O18	-	C7–O9–C10–C11	98.1
C7–O9–C10	117.5	O9–C10–C11–C12	60.1
C19–O18–C15	117.3		

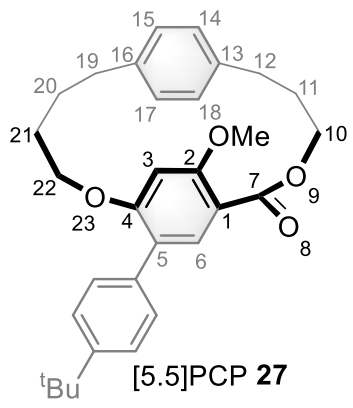

[5.5]PCP **27**

bond angles (°)		dihedral angles (°)	
C11–C10–O9	112.3	C1–C7–O9–C10	171.6
C21–C22–O23	111.2	C7–O9–C10–C11	72.1
C7–O9–C10	116.2	O9–C10–C11–C12	54.3
C4–O23–C22	118.9	C3–C4–O23–C22	46.1
		C4–O23–C22–C21	88.4
		O23–C22–C21–C20	54.5

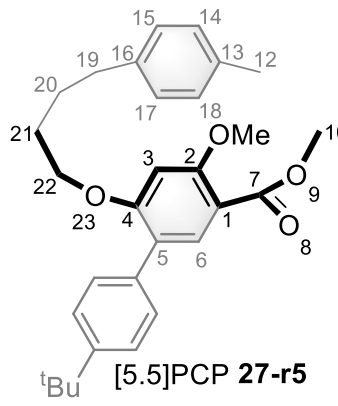

[5.5]PCP **27-r5**

bond angles (°)		dihedral angles (°)	
C11–C10–O9	-	C1–C7–O9–C10	172.0
C21–C22–O23	111.0	C7–O9–C10–C11	-
C7–O9–C10	115.2	O9–C10–C11–C12	-
C4–O23–C22	119.5	C3–C4–O23–C22	44.9
		C4–O23–C22–C21	88.1
		O23–C22–C21–C20	55.5

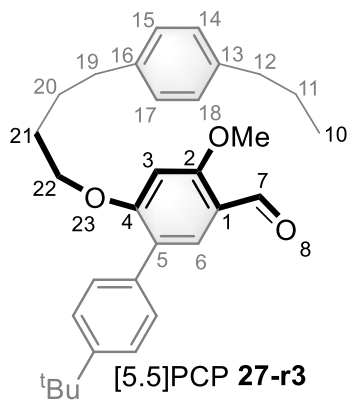

[5.5]PCP **27-r3**

bond angles (°)		dihedral angles (°)	
C11–C10–O9	-	C1–C7–O9–C10	-
C21–C22–O23	110.7	C7–O9–C10–C11	-
C7–O9–C10	-	O9–C10–C11–C12	-
C4–O23–C22	119.5	C3–C4–O23–C22	44.5
		C4–O23–C22–C21	85.3
		O23–C22–C21–C20	53.3

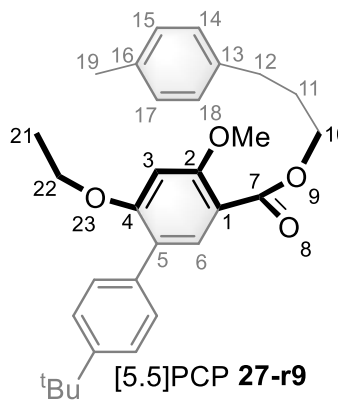

[5.5]PCP **27-r9**

bond angles (°)		dihedral angles (°)	
C11–C10–O9	112.0	C1–C7–O9–C10	172.9
C21–C22–O23	111.8	C7–O9–C10–C11	72.2
C7–O9–C10	116.4	O9–C10–C11–C12	48.4
C4–O23–C22	115.4	C3–C4–O23–C22	75.8
		C4–O23–C22–C21	74.2
		O23–C22–C21–C20	-

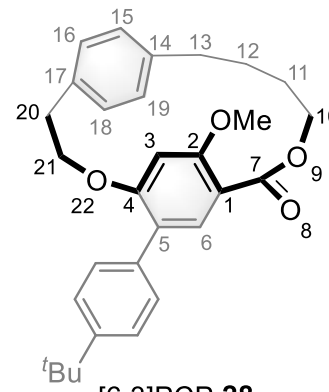

[6.3]PCP **28**

bond angles (°)		dihedral angles (°)	
C11–C10–O9	109.0	C1–C7–O9–C10	158.5
C20–C21–O22	113.9	C7–O9–C10–C11	73.1
C7–O9–C10	115.3	O9–C10–C11–C12	64.0
C4–O22–C21	114.9	C3–C4–O22–C21	122.7
		C4–O22–C21–C20	53.1

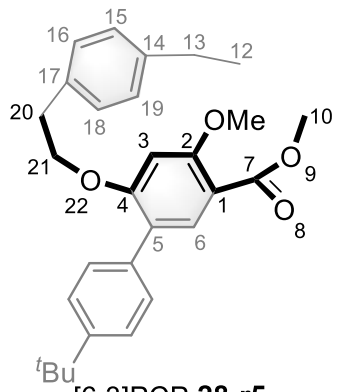

[6.3]PCP **28-r5**

bond angles (°)		dihedral angles (°)	
C11–C10–O9	-	C1–C7–O9–C10	175.9
C20–C21–O22	113.8	C7–O9–C10–C11	-
C7–O9–C10	115.1	O9–C10–C11–C12	-
C4–O22–C21	116.7	C3–C4–O22–C21	125.3
		C4–O22–C21–C20	55.6

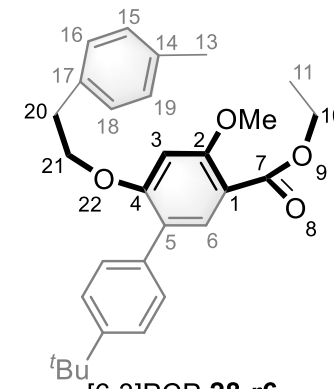

[6.3]PCP **28-r6**

bond angles (°)		dihedral angles (°)	
C11–C10–O9	111.0	C1–C7–O9–C10	179.2
C20–C21–O22	114.0	C7–O9–C10–C11	80.1
C7–O9–C10	116.1	O9–C10–C11–C12	-
C4–O22–C21	116.3	C3–C4–O22–C21	124.7
		C4–O22–C21–C20	54.0

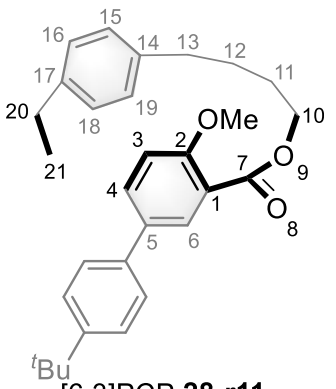

[6.3]PCP **28-r11**

bond angles (°)		dihedral angles (°)	
C11–C10–O9	109.8	C1–C7–O9–C10	164.3
C20–C21–O22	-	C7–O9–C10–C11	77.4
C7–O9–C10	116.1	O9–C10–C11–C12	61.0
C4–O22–C21	-	C3–C4–O22–C21	-
		C4–O22–C21–C20	-

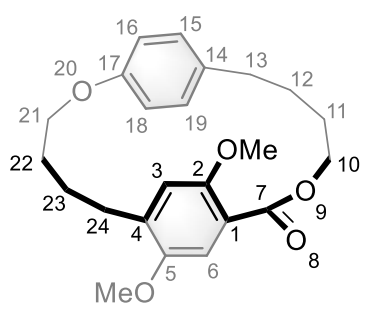

[6.5]PCP **2**

bond angles (°)		dihedral angles (°)	
C11–C10–O9	109.5	C1–C7–O9–C10	172.0
C22–C21–O20	108.5	C7–O9–C10–C11	76.4
C7–O9–C10	115.5	O9–C10–C11–C12	63.1
C21–O20–C17	117.4	C10–C11–C12–C13	139.4
		C11–C12–C13–C14	78.2
		C12–C13–C14–C15	84.2

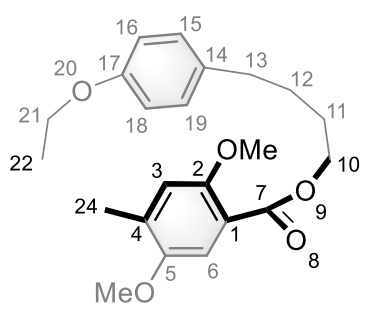

[6.5]PCP **2-r12**

bond angles (°)		dihedral angles (°)	
C11–C10–O9	109.9	C1–C7–O9–C10	170.4
C22–C21–O20	107.6	C7–O9–C10–C11	72.3
C7–O9–C10	116.5	O9–C10–C11–C12	55.2
C21–O20–C17	117.5	C10–C11–C12–C13	141.0
		C11–C12–C13–C14	72.3
		C12–C13–C14–C15	85.6

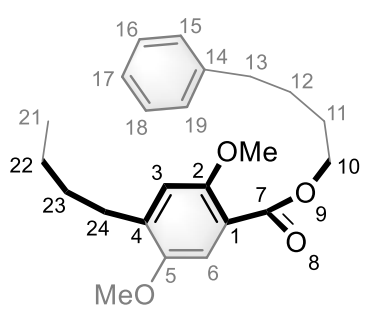

[6.5]PCP **2-r9**

bond angles (°)		dihedral angles (°)	
C11–C10–O9	110.5	C1–C7–O9–C10	177.7
C22–C21–O20	-	C7–O9–C10–C11	76.4
C7–O9–C10	116.5	O9–C10–C11–C12	58.5
C21–O20–C17	-	C10–C11–C12–C13	149.4
		C11–C12–C13–C14	67.2
		C12–C13–C14–C15	69.9

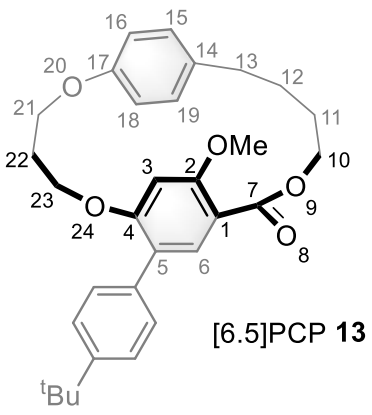

[6.5]PCP **13**

bond angles (°)		dihedral angles (°)	
C11–C10–O9	109.2	C1–C7–O9–C10	168.5
C22–C21–O20	107.9	C7–O9–C10–C11	75.5
C22–C23–O24	110.8	O9–C10–C11–C12	63.1
C7–O9–C10	115.6	C3–C4–O24–C23	125.7
C21–O20–C17	117.9	C4–O24–C23–C22	100.7
C4–O24–C23	117.3	O24–C23–C22–C21	55.9

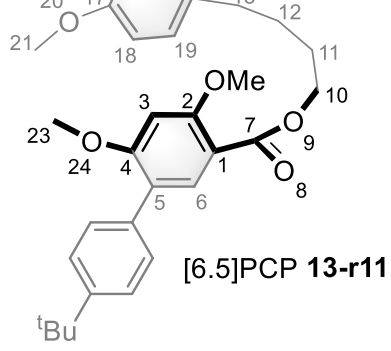

[6.5]PCP **13-r11**

bond angles (°)		dihedral angles (°)	
C11–C10–O9	109.7	C1–C7–O9–C10	172.3
C22–C21–O20	-	C7–O9–C10–C11	74.3
C22–C23–O24	-	O9–C10–C11–C12	60.7
C7–O9–C10	115.7	C3–C4–O24–C23	137.6
C21–O20–C17	116.8	C4–O24–C23–C22	-
C4–O24–C23	118.9	O24–C23–C22–C21	-

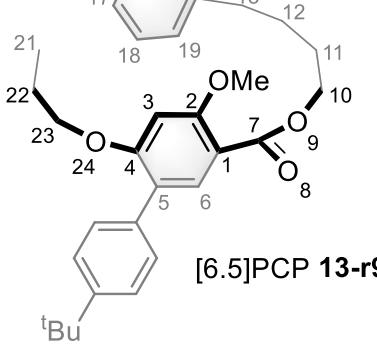

[6.5]PCP **13-r9**

bond angles (°)		dihedral angles (°)	
C11–C10–O9	109.6	C1–C7–O9–C10	170.4
C22–C21–O20	-	C7–O9–C10–C11	73.9
C22–C23–O24	108.9	O9–C10–C11–C12	58.3
C7–O9–C10	116.1	C3–C4–O24–C23	126.4
C21–O20–C17	-	C4–O24–C23–C22	120.4
C4–O24–C23	118.6	O24–C23–C22–C21	66.0

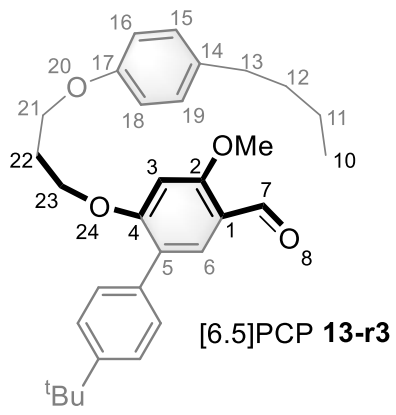

[6.5]PCP **13-r3**

bond angles (°)		dihedral angles (°)	
C11–C10–O9	-	C1–C7–O9–C10	-
C22–C21–O20	107.7	C7–O9–C10–C11	-
C22–C23–O24	109.6	O9–C10–C11–C12	-
C7–O9–C10	-	C3–C4–O24–C23	125.3
C21–O20–C17	117.7	C4–O24–C23–C22	116.5
C4–O24–C23	117.5	O24–C23–C22–C21	53.6

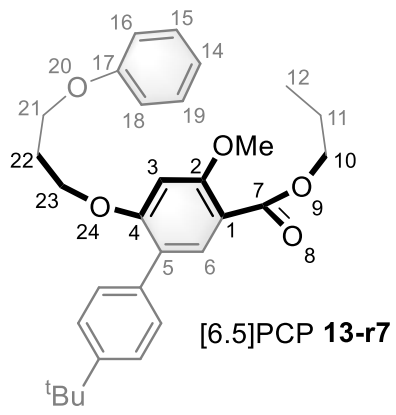

[6.5]PCP **13-r7**

bond angles (°)		dihedral angles (°)	
C11–C10–O9	110.4	C1–C7–O9–C10	173.9
C22–C21–O20	107.6	C7–O9–C10–C11	79.8
C22–C23–O24	110.8	O9–C10–C11–C12	56.1
C7–O9–C10	116.5	C3–C4–O24–C23	125.0
C21–O20–C17	118.6	C4–O24–C23–C22	100.9
C4–O24–C23	117.7	O24–C23–C22–C21	58.3

4) StrainViz decomposition analysis references

[1] P. George, M. Trachtman, C. W. Bock and A. M. Brett, *Tetrahedron*, **1976**, *32*, 1357–1362.

[2] S. E. Wheeler, K. N. Houk, P. v. R. Schleyer and W. D. Allen, *J. Am. Chem. Soc.*, **2009**, *131*, 2547–2560.

[3] Y. Zhao and D. G. Truhlar, *Theor. Chem. Acc.*, **2008**, *120*, 215–241.

[4] E. Cancès, B. Mennucci and J. Tomasi, *J. Chem. Phys.*, **1997**, *107*, 3032–3041.

[5] S. Grimme, *J. Chem. Theory Comput.*, **2019**, *15*, 2847–2862.

[6] C. Bannwarth, S. Ehlert and S. Grimme, *J. Chem. Theory Comput.*, **2019**, *15*, 1652–1671.

[7] S. Ehlert, M. Stahn, S. Spicher and S. Grimme, *J. Chem. Theory Comput.*, **2021**, *17*, 4250–4261.

[8] S. Grimme, *J. Chem. Phys.*, **2006**, *124*, 034108.

[9] A. D. Becke and E. R. Johnson, *J. Chem. Phys.*, **2005**, *123*, 154101.

[10] F. Weigend and R. Ahlrichs, *Phys. Chem. Chem. Phys.*, **2005**, *7*, 3297–3305.

[11] A. Klamt and G. Schüürmann, *J. Chem. Soc., Perkin Trans. 2*, **1993**, 799–805.

[12] P. Pracht, S. Grimme, C. Bannwarth, F. Bohle, S. Ehlert, G. Feldmann, J. Gorges, M. Müller, T. Neudecker, C. Plett, S. Spicher, P. Steinbach, P. A. Wesołowski and F. Zeller, *J. Chem. Phys.*, **2024**, *160*, 114110.

[13] M. J. Frisch, G. W. Trucks, H. B. Schlegel, G. E. Scuseria, M. A. Robb, J. R. Cheeseman, G. Scalmani, V. Barone, G. A. Petersson, H. Nakatsuji, X. Li, M. Caricato, A. V. Marenich, J. Bloino, B. G. Janesko, R. Gomperts, B. Mennucci, H. P. Hratchian, J. V. Ortiz, A. F. Izmaylov, J. L. Sonnenberg, D. Williams-Young, F. Ding, F. Lipparini, F. Egidi, J. Goings, B. Peng, A. Petrone, T. Henderson, D. Ranasinghe, V. G. Zakrzewski, J. Gao, N. Rega, G. Zheng, W. Liang, M. Hada, M. Ehara, K. Toyota, R. Fukuda, J. Hasegawa, M. Ishida, T. Nakajima, Y. Honda, O. Kitao, H. Nakai, T. Vreven, K. Throssell, J. A. Montgomery, Jr., J. E. Peralta, F. Ogliaro, M. J. Bearpark, J. J. Heyd, E. N. Brothers, K. N. Kudin, V. N. Staroverov, T. A. Keith, R. Kobayashi, J. Normand, K. Raghavachari, A. P. Rendell, J. C. Burant, S. S. Iyengar, J. Tomasi, M. Cossi, J. M. Millam, M. Klene, C. Adamo, R. Cammi, J. W. Ochterski, R. L. Martin, K. Morokuma, O. Farkas, J. B. Foresman and D. J. Fox, Gaussian 16, Revision A.03, Gaussian, Inc., Wallingford CT, 2016.

[14] F. Neese, *WIREs Comput. Mol. Sci.*, **2025**, *15*, e70019.

[15] F. Neese, F. Wennmohs, A. Hansen and U. Becker, *Chem. Phys.*, **2009**, *356*, 98–109.

[16] F. Weigend, *Phys. Chem. Chem. Phys.*, **2006**, *8*, 1057–1065.

[17] C. E. Colwell, T. W. Price, T. Stauch and R. Jasti, *Chem. Sci.*, **2020**, *11*, 3923–3930.

[18] P. C. Hariharan and J. A. Pople, *Theor. Chim. Acta*, **1973**, *28*, 213–222.

[19] T. Clark, J. Chandrasekhar, G. W. Spitznagel and P. v. R. Schleyer, *J. Comput. Chem.*, **1983**, *4*, 294–301.

[20] P. von R. Schleyer, C. Maerker, A. Dransfeld, H. Jiao and N. J. R. van Eikema Hommes, *J. Am. Chem. Soc.*, **1996**, *118*, 6317–6318.

[21] E. Steiner, P. W. Fowler and L. W. Jenneskens, *Angew. Chem. Int. Ed.*, **2001**, *40*, 362–366.

[22] H. Fallah-Bagher-Shaidaei, C. S. Wannere, C. Corminboeuf, R. Puchta and P. v. R. Schleyer, *Org. Lett.*, **2006**, *8*, 863–866.

[23] T. Lu and F. Chen, *J. Comput. Chem.*, **2012**, *33*, 580–592.

[24] T. Lu and Q. Chen, *J. Comput. Chem.*, **2022**, *43*, 539–555.

[25] H. Fliegl, S. Taubert, O. Lehtonen and D. Sundholm, *Phys. Chem. Chem. Phys.*, **2011**, *13*, 20500–20518.

[26] D. Sundholm, H. Fliegl and R. J. F. Berger, *WIREs Comput. Mol. Sci.*, **2016**, *6*, 639–678.

[27] C. Y. Legault, CYLview, 1.0b, Université de Sherbrooke, 2009, http://www.cylview.org.

5) Optimized molecular geometries

The following Cartesian coordinates and energies are in Ångström and Hartree, respectively.

C_2H_6

M06-2X/6-31G(d) single-point energy: -79.7719801

M06-2X/6-31G(d) zero-point correction: 0.075727

M06-2X/6-31G(d) thermal correction to enthalpy: 0.004416

B2PLYP-D3BJ/def2-TZVP single-point energy: -79.769508

Final enthalpy: -79.689365

Cartesian coordinates

C	-0.76372	0.00000	-0.00001
H	-1.15876	0.99788	-0.21204
H	-1.15873	-0.31530	0.97021
H	-1.15874	-0.68261	-0.75813
C	0.76372	-0.00001	0.00001
H	1.15875	-0.99787	0.21204
H	1.15875	0.31532	-0.97019
H	1.15872	0.68260	0.75814

$C_{13}H_{18}O_4$ h1

M06-2X/6-31G(d) single-point energy: -806.775791

M06-2X/6-31G(d) zero-point correction: 0.298293

M06-2X/6-31G(d) thermal correction to enthalpy: 0.018884

B2PLYP-D3BJ/def2-TZVP single-point energy: -806.796210

Final enthalpy: -806.479033

Cartesian coordinates

C	1.15989	1.52101	-0.20427
C	2.17603	0.57449	-0.19849
C	1.83063	-0.78844	-0.19138
C	0.49593	-1.15822	-0.19582
C	-0.53130	-0.20206	-0.20244
C	-0.19705	1.16434	-0.20552
H	1.44494	2.56679	-0.20960
H	0.19495	-2.19836	-0.19417
C	-1.91069	-0.77270	-0.20576
O	-2.12273	-1.97030	-0.20428
O	-2.89039	0.13410	-0.21647
C	-0.82836	3.45435	-0.21323
O	-1.18908	2.08561	-0.21138
H	-1.76705	4.00710	-0.21609
H	-0.25361	3.71663	0.68183
H	-0.25019	3.71337	-1.10701
O	2.87342	-1.66788	-0.18361
C	2.55676	-3.04637	-0.19360
H	1.98065	-3.32693	0.69552
H	1.98873	-3.31563	-1.09140
H	3.50875	-3.57616	-0.19245
C	-4.23230	-0.38292	-0.21053
C	3.62483	0.98616	-0.15667
C	-4.65891	-0.77821	1.19094
H	-4.29031	-1.22846	-0.89891

H	-4.83889	0.43846	-0.59469
H	-5.70437	-1.09892	1.18355
H	-4.56167	0.07084	1.87247
H	-4.04494	-1.60283	1.55936
C	4.21780	0.83044	1.24927
H	3.71213	2.02721	-0.48346
H	4.19546	0.37100	-0.85964
H	5.27012	1.12808	1.26092
H	4.14964	-0.20989	1.57788
H	3.67636	1.45417	1.96689

$C_{10}H_{14}O$ h2

M06-2X/6-31G(d) single-point energy: -464.5048256

M06-2X/6-31G(d) zero-point correction: 0.220628

M06-2X/6-31G(d) thermal correction to enthalpy: 0.012105

B2PLYP-D3BJ/def2-TZVP single-point energy: -464.491991

Final enthalpy: -464.259258

Cartesian coordinates

C	-0.34402	-0.99994	-0.18157
C	-0.90027	0.27511	-0.07272
C	-0.06342	1.39729	-0.10164
C	1.30694	1.23954	-0.23722
C	1.88717	-0.03082	-0.34981
C	1.03926	-1.13509	-0.31759
H	-0.96713	-1.88633	-0.16598
H	-0.51415	2.38128	-0.01969
H	1.94445	2.12079	-0.26011
H	1.46200	-2.13363	-0.40334
O	-2.23082	0.52814	0.05996
C	-3.11601	-0.58282	0.09736

C	3.38551	-0.19193	-0.44349
C	-4.51981	-0.03756	0.25446
H	-2.85173	-1.23848	0.93745
H	-3.02035	-1.16366	-0.82945
H	-4.77117	0.61402	-0.58613
H	-4.60321	0.53827	1.17957
H	-5.23987	-0.85891	0.28771
C	4.04869	-0.18841	0.93840
H	3.80526	0.61897	-1.04972
H	3.62226	-1.12895	-0.95949
H	3.66187	-1.00812	1.55192
H	3.84033	0.74931	1.46332
H	5.13378	-0.30183	0.85712

C_3H_8

M06-2X/6-31G(d) single-point energy: -119.064766

M06-2X/6-31G(d) zero-point correction: 0.104847

M06-2X/6-31G(d) thermal correction to enthalpy: 0.005436

B2PLYP-D3BJ/def2-TZVP single-point energy: -119.058231

Final enthalpy: -118.947948

Cartesian coordinates

C	1.26670	-0.26129	0.00000
C	0.00000	0.59222	-0.00000
H	1.29952	-0.90720	-0.88430
H	1.29951	-0.90722	0.88428
C	-1.26670	-0.26129	-0.00000
H	0.00001	1.24875	-0.87796
H	-0.00001	1.24873	0.87797
H	-2.16953	0.35676	-0.00021
H	-1.29961	-0.90706	0.88439

H	-1.29941	-0.90736	-0.88418
H	2.16952	0.35677	0.00001

$C_{22}H_{28}O_4$ h3

M06-2X/6-31G(d) single-point energy: -1155.624842

M06-2X/6-31G(d) zero-point correction: 0.465505

M06-2X/6-31G(d) thermal correction to enthalpy: 0.027487

B2PLYP-D3BJ/def2-TZVP single-point energy: -1155.617723

Final enthalpy: -1155.124731

Cartesian coordinates

C	2.01037	1.93020	0.01674
C	0.64175	1.65918	0.02486
C	0.16660	0.33225	-0.06820
C	1.12134	-0.66973	-0.15929
C	2.50158	-0.43839	-0.17072
C	2.94924	0.89605	-0.08150
H	2.35004	2.95204	0.09495
H	0.79639	-1.70184	-0.24393
C	3.35182	-1.65391	-0.28574
O	2.88632	-2.77585	-0.35352
O	4.66724	-1.41616	-0.31511
C	4.72059	2.48092	-0.00636
O	4.27463	1.13866	-0.09290
O	-0.29050	2.62795	0.13896
C	-1.27869	-0.00682	-0.04679
C	-1.72934	-1.09848	0.70707
C	-2.22473	0.70050	-0.79360
C	-3.06892	-1.46408	0.70879
H	-1.02037	-1.65800	1.31164
C	-3.56748	0.32933	-0.78746

H	-1.90855	1.54833	-1.39182
C	-4.02264	-0.75898	-0.03776
H	-3.37532	-2.31404	1.31314
H	-4.26194	0.90681	-1.38813
C	-5.49022	-1.19202	-0.00042
C	-6.37545	-0.30926	-0.88582
H	-6.35480	0.73647	-0.56125
H	-6.06528	-0.35186	-1.93528
H	-7.41164	-0.65772	-0.82788
C	-5.60695	-2.64401	-0.49408
H	-5.02822	-3.33043	0.13147
H	-6.65379	-2.96670	-0.46891
H	-5.24372	-2.73378	-1.52319
C	-6.00925	-1.10327	1.44487
H	-5.43938	-1.75147	2.11765
H	-5.93732	-0.07696	1.81988
H	-7.05954	-1.41273	1.48756
C	5.51672	-2.57154	-0.41660
C	0.12573	3.98528	0.26128
H	4.40365	2.94437	0.93392
H	4.35601	3.07502	-0.85107
H	5.80804	2.43194	-0.03873
C	-1.12837	4.82587	0.37078
H	0.71651	4.26859	-0.61859
H	0.75653	4.09543	1.15198
H	-1.71113	4.52767	1.24551
H	-1.74794	4.70337	-0.52112
H	-0.86227	5.88105	0.46951
C	5.69675	-3.23773	0.93480
H	6.46135	-2.17900	-0.79608

H	5.09233	-3.26222	-1.14811
H	6.08984	-2.52303	1.66255
H	6.40286	-4.06828	0.84823
H	4.74348	-3.62791	1.29764

$C_{10}H_{14}$ h4

M06-2X/6-31G(d) single-point energy: -389.318023

M06-2X/6-31G(d) zero-point correction: 0.215628

M06-2X/6-31G(d) thermal correction to enthalpy: 0.01124

B2PLYP-D3BJ/def2-TZVP single-point energy: -389.291414

Final enthalpy: -389.064546

Cartesian coordinates

C	0.67483	-1.19815	0.17017
C	1.37162	-0.00004	0.34747
C	0.67499	1.19821	0.17048
C	-0.67484	1.19834	-0.17017
C	-1.37162	0.00024	-0.34747
C	-0.67499	-1.19802	-0.17048
H	1.19852	-2.14286	0.30059
H	1.19880	2.14282	0.30115
H	-1.19852	2.14305	-0.30059
H	-1.19880	-2.14262	-0.30115
C	-2.84902	0.00035	-0.65895
C	2.84902	-0.00019	0.65895
C	-3.69777	-0.00048	0.61756
H	-3.09753	-0.87981	-1.26236
H	-3.09762	0.88122	-1.26127
H	-3.47967	0.88357	1.22489
H	-3.47953	-0.88523	1.22383
H	-4.76598	-0.00043	0.38177

C	3.69777	-0.00009	-0.61756
H	3.09751	-0.88079	1.26170
H	3.09765	0.88023	1.26192
H	3.47965	0.88439	-1.22426
H	3.47955	-0.88441	-1.22446
H	4.76598	-0.00018	-0.38177

[4.4]PCP 25

M06-2X/6-31G(d) single-point energy: -1151.018212

M06-2X/6-31G(d) zero-point correction: 0.394678

M06-2X/6-31G(d) thermal correction to enthalpy: 0.022615

B2PLYP-D3BJ/def2-TZVP single-point energy: -1151.026535

Final enthalpy: -1150.609242

Cartesian coordinates

C	-1.02893	-0.85219	-1.63496
C	-2.09047	-0.11481	-1.12870
C	-1.85099	1.19465	-0.67896
C	-0.56142	1.69665	-0.69048
C	0.52515	0.92197	-1.13139
C	0.28696	-0.36300	-1.64034
H	-1.23333	-1.85481	-1.99368
H	-0.33244	2.67711	-0.29202
C	0.31532	-1.91847	1.25258
C	-0.44199	-0.85938	1.75546
C	0.21425	0.28797	2.22035
C	1.58812	0.41508	2.08214
C	2.34900	-0.58989	1.47107
C	1.69257	-1.75917	1.09443
H	-0.14919	-2.84979	0.94887
H	-0.38564	1.08887	2.64148

H	2.06954	1.33619	2.40380
H	2.26271	-2.56525	0.63802
C	1.86000	1.49056	-0.79167
O	1.99719	2.61942	-0.36550
O	2.88715	0.62809	-0.90404
C	3.93519	0.76638	0.06495
C	3.79657	-0.36688	1.09757
C	-3.45103	-0.74126	-0.96681
C	-3.76639	-1.10722	0.49970
C	-2.61381	-1.81386	1.21165
O	-1.79863	-0.82107	1.83236
H	4.88488	0.70011	-0.46929
H	3.86151	1.74928	0.53250
H	4.20348	-1.29197	0.67797
H	4.40931	-0.10630	1.96888
H	-2.97948	-2.48905	1.99275
H	-2.03559	-2.40314	0.49324
H	-4.00818	-0.20395	1.06714
H	-4.65216	-1.75080	0.51389
H	-4.22795	-0.06492	-1.33675
H	-3.49026	-1.65149	-1.57502
C	1.09680	-2.44419	-2.43877
O	1.32967	-1.09065	-2.10208
H	0.67644	-2.99433	-1.58850
H	0.42541	-2.53521	-3.30002
H	2.07117	-2.85904	-2.69544
O	-2.94051	1.87041	-0.21274
C	-2.71372	3.15157	0.34013
H	-2.03297	3.09566	1.19774
H	-2.29748	3.83828	-0.40595

H	-3.68582	3.51791	0.66879

[4.5]PCP 26

M06-2X/6-31G(d) single-point energy: -1190.316081

M06-2X/6-31G(d) zero-point correction: 0.424501

M06-2X/6-31G(d) thermal correction to enthalpy: 0.023571

B2PLYP-D3BJ/def2-TZVP single-point energy: -1190.319321

Final enthalpy: -1189.871249

Cartesian coordinates

C	0.95516	-0.23161	1.74916
C	1.96543	0.46409	1.09679
C	1.61893	1.61885	0.37085
C	0.28913	1.98029	0.24582
C	-0.73685	1.23034	0.84123
C	-0.39888	0.12298	1.63447
H	1.23623	-1.09525	2.34177
H	-0.01175	2.82815	-0.35619
C	-0.49690	-2.25589	-0.66713
C	0.26253	-1.37298	-1.43719
C	-0.38342	-0.37325	-2.17341
C	-1.75825	-0.21122	-2.07105
C	-2.52458	-1.02966	-1.23031
C	-1.87247	-2.05962	-0.55729
H	-0.02937	-3.07342	-0.13033
H	0.21865	0.29131	-2.78528
H	-2.23737	0.59915	-2.61643
H	-2.44505	-2.71644	0.09350
C	-2.10600	1.60414	0.38814
O	-2.31331	2.57560	-0.31133
O	-3.07921	0.74432	0.73829

C	-4.15261	0.59964	-0.20191
C	-3.97993	-0.74001	-0.94247
C	2.31146	-2.27661	-0.63366
O	1.62007	-1.40595	-1.51946
H	-5.08588	0.62060	0.36377
H	-4.13880	1.44528	-0.89024
H	-4.38590	-1.54966	-0.32867
H	-4.57692	-0.69773	-1.86108
H	2.20517	-3.31530	-0.97487
H	1.87305	-2.20427	0.36806
C	-1.04352	-1.75699	2.92940
O	-1.38614	-0.56224	2.25600
H	-0.55750	-2.46677	2.24943
H	-0.38666	-1.56247	3.78476
H	-1.98348	-2.17872	3.28456
O	2.65599	2.31244	-0.18279
C	2.33113	3.43863	-0.97340
H	1.80628	4.19938	-0.38427
H	3.27821	3.84337	-1.32896
H	1.70913	3.15398	-1.83012
C	3.41025	0.02552	1.15686
C	4.00664	-0.40321	-0.20333
C	3.77163	-1.86471	-0.59572
H	4.00241	0.86617	1.53577
H	3.51297	-0.78973	1.88371
H	5.08898	-0.23817	-0.17033
H	3.61492	0.25131	-0.98538
H	4.22194	-2.04599	-1.57843
H	4.27834	-2.53044	0.11428

[5.5]PCP

M06-2X/6-31G(d) single-point energy: -1229.617794

M06-2X/6-31G(d) zero-point correction: 0.453355

M06-2X/6-31G(d) thermal correction to enthalpy: 0.024879

B2PLYP-D3BJ/def2-TZVP single-point energy: -1229.616624

Final enthalpy: -1229.138390

Cartesian coordinates

C	1.27080	-0.21352	1.77166
C	2.18385	0.54553	1.05033
C	1.70366	1.63842	0.30621
C	0.34365	1.89480	0.25771
C	-0.58052	1.08671	0.93495
C	-0.11100	0.02360	1.72093
H	1.65491	-1.03107	2.37190
H	-0.05943	2.70802	-0.33294
C	-0.23666	-2.20388	-0.67642
C	0.50220	-1.29609	-1.43643
C	-0.16824	-0.32640	-2.18952
C	-1.55236	-0.24621	-2.14925
C	-2.31116	-1.11938	-1.35785
C	-1.62742	-2.09419	-0.63508
H	0.25431	-2.98772	-0.11041
H	0.42084	0.36533	-2.78405
H	-2.05272	0.51895	-2.73886
H	-2.18892	-2.78929	-0.01518
C	-2.00832	1.41138	0.66049
O	-2.35274	2.42497	0.08246
O	-2.87681	0.47657	1.05567
C	-3.82290	-1.02297	-1.31747
C	2.58810	-2.14788	-0.65657

O	1.86153	-1.27534	-1.50827
H	-4.21810	-1.24523	-2.31663
H	2.49995	-3.18166	-1.01820
H	2.17010	-2.10898	0.35524
C	-0.52908	-1.89356	3.05332
O	-1.00309	-0.73042	2.40534
H	0.15519	-1.64893	3.87358
H	-1.41097	-2.39091	3.45623
H	-0.02435	-2.56075	2.34435
O	2.64754	2.38931	-0.33320
C	2.18794	3.46356	-1.12884
H	1.64935	4.20316	-0.52523
H	3.07631	3.92381	-1.56038
H	1.53199	3.10811	-1.93217
C	4.03873	-1.69713	-0.64575
C	4.23916	-0.21911	-0.29698
C	3.66207	0.23202	1.06200
H	4.57394	-2.33024	0.07301
H	4.48120	-1.88954	-1.62994
H	5.31703	-0.02309	-0.29009
H	3.81189	0.40309	-1.08688
H	4.19682	1.13778	1.37012
H	3.86336	-0.53199	1.82305
C	-4.37591	0.34418	-0.88628
C	-4.22755	0.63662	0.60094
H	-4.21573	-1.80033	-0.65046
H	-3.89916	1.14368	-1.46128
H	-5.44759	0.38586	-1.11314
H	-4.57273	1.64699	0.83490
H	-4.79341	-0.08483	1.19493

[6.5]PCP 2

M06-2X/6-31G(d) single-point energy: -1268.909310

M06-2X/6-31G(d) zero-point correction: 0.482771

M06-2X/6-31G(d) thermal correction to enthalpy: 0.026011

B2PLYP-D3BJ/def2-TZVP single-point energy: -1268.904882

Final enthalpy: -1,268.396100

Cartesian coordinates

C	1.36413	0.10862	1.72282
C	2.36983	0.67273	0.94865
C	2.01766	1.66110	0.01091
C	0.68886	2.00676	-0.15832
C	-0.32882	1.41036	0.60072
C	0.01054	0.44595	1.56275
H	1.64945	-0.63050	2.46310
H	0.38359	2.74137	-0.89272
C	-0.10245	-2.47288	-0.22523
C	0.61099	-1.64545	-1.09688
C	-0.08632	-0.84111	-2.00266
C	-1.47577	-0.86672	-2.03465
C	-2.21093	-1.68297	-1.16570
C	-1.49483	-2.47145	-0.26358
H	0.41039	-3.12726	0.47020
H	0.48093	-0.20180	-2.67210
H	-1.99827	-0.24848	-2.76112
H	-2.03770	-3.12091	0.42055
C	-1.70577	1.83607	0.22896
O	-1.93030	2.56143	-0.72197
O	-2.67484	1.36734	1.02227
C	2.69476	-2.32103	-0.17519

O	1.96966	-1.57328	-1.13931
H	2.56771	-3.39585	-0.36396
H	2.30159	-2.10772	0.82604
C	-0.62010	-1.17957	3.17322
O	-0.97269	-0.13050	2.29349
H	-0.12783	-1.99784	2.63532
H	0.03337	-0.82557	3.97864
H	-1.55734	-1.53682	3.59933
O	3.04981	2.22825	-0.67887
C	2.72013	3.19295	-1.65832
H	2.21481	4.05654	-1.21088
H	3.66380	3.51217	-2.09965
H	2.07829	2.76325	-2.43629
C	4.15799	-1.92785	-0.26101
C	4.41404	-0.42205	-0.15510
C	3.81744	0.26478	1.09293
H	4.68316	-2.45667	0.54422
H	4.57493	-2.29312	-1.20644
H	5.49842	-0.26730	-0.14835
H	4.03232	0.07597	-1.04924
H	4.40334	1.16880	1.29407
H	3.92665	-0.38757	1.96811
C	-4.36432	0.66601	-0.55635
C	-4.35171	-0.80676	-0.11774
C	-3.72256	-1.73695	-1.17015
C	-4.01778	1.61988	0.57570
H	-4.63816	1.44291	1.45717
H	-4.10689	2.66387	0.26889
H	-3.63839	0.83396	-1.35904
H	-5.34490	0.93787	-0.96234

H	-5.37376	-1.13530	0.09996
H	-3.77966	-0.90850	0.81273
H	-4.10694	-1.47595	-2.16387
H	-4.03632	-2.76794	-0.97179

[6.5]PCP 13

M06-2X/6-31G(d) single-point energy: -1578.463188

M06-2X/6-31G(d) zero-point correction: 0.620659

M06-2X/6-31G(d) thermal correction to enthalpy: 0.033458

B2PLYP-D3BJ/def2-TZVP single-point energy: -1578.434169

Final enthalpy: -1,577.780052

Cartesian coordinates

C	0.91103	1.31457	1.75825
C	-0.40223	1.18054	1.31563
C	-0.88511	-0.05389	0.84244
C	0.04353	-1.08531	0.74334
C	1.38205	-0.96747	1.12019
C	1.82260	0.25963	1.66063
H	1.20911	2.28292	2.13915
H	-0.27734	-2.05039	0.36393
C	1.55455	0.38933	-2.07410
C	1.58674	1.66693	-1.50967
C	2.80660	2.20306	-1.09083
C	3.96928	1.44791	-1.22982
C	3.96278	0.17327	-1.79909
C	2.72922	-0.33979	-2.21983
H	0.59776	-0.01321	-2.39250
H	2.86503	3.20268	-0.67492
H	4.91352	1.87579	-0.89873
H	2.68491	-1.32333	-2.68179

C	2.23103	-2.15133	0.82551
O	1.82849	-3.10012	0.17933
O	3.47294	-2.08749	1.31889
C	0.36201	3.55252	-0.71519
O	0.39339	2.31852	-1.41718
H	0.85055	4.33758	-1.30655
H	0.90427	3.45116	0.23131
C	3.55597	1.64783	2.48934
O	3.10896	0.38088	2.04122
H	4.61849	1.52914	2.69847
H	3.41650	2.40817	1.71309
H	3.03472	1.95274	3.40317
C	-1.09225	3.89626	-0.43822
C	-1.84240	2.73258	0.19614
H	-1.11956	4.77269	0.21744
H	-1.60486	4.16204	-1.36960
H	-2.85561	3.02817	0.48029
H	-1.90933	1.90636	-0.51275
C	4.81161	-2.73285	-0.58043
C	5.58288	-1.40682	-0.65245
C	5.25861	-0.59717	-1.92167
C	4.38473	-3.08823	0.83532
H	5.22501	-3.05544	1.53237
H	3.90731	-4.06878	0.88319
H	3.90410	-2.68214	-1.19169
H	5.41581	-3.55045	-0.98906
H	6.65908	-1.60603	-0.60472
H	5.33240	-0.78857	0.21834
H	5.21880	-1.27203	-2.78562
H	6.07025	0.11314	-2.11380

C	-2.30794	-0.29834	0.49728
C	-3.34583	0.18673	1.30576
C	-2.65375	-1.05071	-0.62659
C	-4.67143	-0.07785	0.99242
H	-3.10525	0.77510	2.18650
C	-3.98770	-1.31571	-0.93387
H	-1.87048	-1.42485	-1.28101
C	-5.02695	-0.83573	-0.13373
H	-5.44747	0.31152	1.64648
H	-4.20492	-1.90236	-1.81982
C	-6.50380	-1.09895	-0.43683
C	-7.22726	0.24242	-0.64502
H	-8.28780	0.06860	-0.85856
H	-7.16237	0.87717	0.24407
H	-6.79204	0.79077	-1.48707
C	-7.14266	-1.84260	0.74849
H	-7.07990	-1.25921	1.67220
H	-8.20150	-2.03619	0.54377
H	-6.64383	-2.80252	0.91789
C	-6.69142	-1.94955	-1.69705
H	-7.75985	-2.11303	-1.87051
H	-6.28008	-1.45419	-2.58290
H	-6.21395	-2.92991	-1.59589
O	-1.18609	2.29813	1.39871

[6.3]PCP 28

M06-2X/6-31G(d) single-point energy: -1463.976741

M06-2X/6-31G(d) zero-point correction: 0.585904

M06-2X/6-31G(d) thermal correction to enthalpy: 0.031495

B2PLYP-D3BJ/def2-TZVP single-point energy: -1463.940263

Final enthalpy: -1463.322864

Cartesian coordinates

C	1.64510	-2.63818	-0.55986
C	0.32879	-2.24310	-0.34781
C	-0.13608	-0.97911	-0.74901
C	0.82704	-0.08853	-1.21645
C	2.17724	-0.41940	-1.35393
C	2.58444	-1.74220	-1.07246
H	1.91953	-3.63750	-0.24710
H	0.52234	0.91654	-1.48518
C	2.10910	-0.65596	2.13651
C	0.76045	-0.92631	2.37607
C	-0.11042	0.16080	2.49778
C	0.35085	1.46488	2.35817
C	1.70029	1.73928	2.10745
C	2.57402	0.65304	2.00909
H	2.80975	-1.48291	2.04314
H	-1.16592	-0.01053	2.69946
H	-0.35000	2.29211	2.45274
H	3.63440	0.82437	1.83815
C	3.16285	0.65458	-1.63511
O	4.36630	0.55401	-1.54963
O	2.55497	1.82540	-1.93151
C	0.24834	-2.34460	2.53893
C	4.27502	-3.39781	-0.96806
O	3.87231	-2.07480	-1.27881
H	5.33136	-3.45392	-1.22647
H	4.14711	-3.60834	0.09945
H	3.71246	-4.13085	-1.55579
C	3.51444	3.26499	-0.25515

C	2.18255	3.59076	0.43335
C	2.14982	3.17163	1.91856
C	3.36099	3.00003	-1.74627
H	4.32653	2.85970	-2.23557
H	2.81015	3.79893	-2.24760
H	4.22751	4.08337	-0.10436
H	3.96922	2.37374	0.19079
H	1.36383	3.07901	-0.08825
H	1.98804	4.66550	0.34689
H	1.46132	3.82514	2.46441
H	3.14311	3.32148	2.35960
C	-1.56034	-0.57585	-0.66270
C	-1.91909	0.72729	-0.29299
C	-2.58852	-1.46777	-0.98282
C	-3.25192	1.11403	-0.24453
H	-1.14333	1.43711	-0.01914
C	-3.92366	-1.07411	-0.92827
H	-2.34127	-2.48323	-1.27611
C	-4.28823	0.22381	-0.55841
H	-3.48679	2.13298	0.05262
H	-4.68443	-1.80199	-1.18861
C	-5.74303	0.69345	-0.48511
C	-6.05874	1.15076	0.94924
H	-5.92213	0.32688	1.65757
H	-5.41258	1.97733	1.26037
H	-7.09782	1.49216	1.01495
C	-6.72801	-0.41674	-0.86488
H	-6.64628	-1.27606	-0.19089
H	-7.75153	-0.03431	-0.79772
H	-6.56685	-0.76507	-1.89052

C	-5.94679	1.87288	-1.45129
H	-6.98459	2.22135	-1.40531
H	-5.29688	2.71687	-1.20048
H	-5.73027	1.57155	-2.48147
O	-0.47543	-3.14519	0.29700
C	-0.90189	-2.74674	1.60888
H	-1.63851	-1.93882	1.53104
H	-1.40530	-3.63252	2.00088
H	-0.12397	-2.46956	3.56369
H	1.06906	-3.06059	2.41490

[5.5]PCP 27

M06-2X/6-31G(d) single-point energy: -1503.275202

M06-2X/6-31G(d) zero-point correction: 0.615457

M06-2X/6-31G(d) thermal correction to enthalpy: 0.032595

B2PLYP-D3BJ/def2-TZVP single-point energy: -1503.233663

Final enthalpy: -1502.585611

Cartesian coordinates

C	-1.34387	2.27138	-1.16669
C	-0.01091	1.90865	-0.96610
C	0.38844	0.56174	-1.03582
C	-0.61304	-0.38438	-1.24688
C	-1.95902	-0.05937	-1.40502
C	-2.32755	1.30267	-1.36755
H	-1.60751	3.32015	-1.15186
H	-0.33782	-1.43279	-1.27253
C	-2.95233	0.64068	1.78787
C	-1.59227	0.72941	2.10478
C	-0.87902	-0.46469	2.22264
C	-1.49827	-1.69611	2.01065

C	-2.85282	-1.78279	1.68917
C	-3.57435	-0.58779	1.59037
H	-3.53325	1.55540	1.68128
H	0.17905	-0.44292	2.47025
H	-0.91262	-2.60929	2.09518
H	-4.63253	-0.61323	1.33979
C	-2.97244	-1.13805	-1.52225
O	-4.12490	-1.00633	-1.87126
O	-2.45700	-2.33357	-1.16832
C	-3.52239	-3.12721	1.48608
C	0.57715	2.11283	2.11725
C	-0.94823	2.08910	2.29473
H	-2.76830	-3.91960	1.56938
H	0.88141	1.34883	1.39528
H	1.06182	1.84089	3.06180
C	-4.01371	2.96923	-1.35783
O	-3.63169	1.61367	-1.50363
H	-5.09901	2.98280	-1.44758
H	-3.72079	3.35834	-0.37560
H	-3.57509	3.59366	-2.14362
C	1.13824	3.45711	1.64557
C	0.66629	3.87233	0.25406
O	0.94151	2.85067	-0.71289
H	0.86525	4.26042	2.34259
H	2.23208	3.39801	1.63524
H	-0.39749	4.12324	0.25416
H	1.21377	4.75451	-0.08511
C	-4.28460	-3.28837	0.16282
C	-3.38835	-3.41662	-1.06184
H	-4.23313	-3.29126	2.30579

H	-4.89334	-4.19872	0.21033
H	-4.97616	-2.45342	0.01738
H	-2.75799	-4.30610	-0.98931
H	-3.98658	-3.47208	-1.97515
C	1.78572	0.12668	-0.79478
C	2.88808	0.80795	-1.32953
C	2.03836	-1.00994	-0.02094
C	4.18089	0.35986	-1.09606
H	2.72625	1.69161	-1.93718
C	3.33920	-1.45452	0.20970
H	1.20340	-1.54657	0.42332
C	4.44098	-0.78015	-0.32103
H	5.00805	0.91304	-1.53374
H	3.48048	-2.33883	0.82165
C	5.88563	-1.22975	-0.09029
C	6.66453	-0.10354	0.61039
H	6.21480	0.13343	1.58031
H	7.70241	-0.41218	0.77830
H	6.67820	0.81102	0.00964
C	5.96654	-2.48662	0.78183
H	7.01542	-2.76882	0.91827
H	5.53316	-2.31760	1.77333
H	5.44919	-3.33304	0.31788
C	6.54602	-1.53718	-1.44511
H	6.00972	-2.33804	-1.96480
H	6.55787	-0.65793	-2.09655
H	7.58261	-1.85862	-1.29393
H	-1.42389	2.77519	1.58347
H	-1.19993	2.47978	3.28997

[12]PCP

M06-2X/6-31G(d) single-point energy: -1041.340585

M06-2X/6-31G(d) zero-point correction: 0.453015

M06-2X/6-31G(d) thermal correction to enthalpy: 0.023483

B2PLYP-D3BJ/def2-TZVP single-point energy: -1041.329752

Final enthalpy: -1040.853254

Cartesian coordinates

C	-1.33704	-0.01393	1.68744
C	-2.28036	-0.49074	0.78435
C	-1.90097	-1.48255	-0.13620
C	-0.57876	-1.89728	-0.19139
C	0.39062	-1.34369	0.65696
C	0.00316	-0.41868	1.64037
H	-1.65526	0.73186	2.40712
H	-0.23718	-2.61554	-0.92693
C	1.79792	-1.68687	0.30191
O	2.09540	-2.63468	-0.39708
O	2.68737	-0.78285	0.72526
C	3.94877	-0.75501	0.03851
C	3.80476	-0.16678	-1.36030
C	-3.63375	0.15521	0.67169
C	-3.66994	1.16483	-0.49708
C	-2.61131	2.27725	-0.39367
H	4.58338	-0.12618	0.66714
H	4.36415	-1.76475	-0.00051
H	4.80256	-0.18430	-1.81514
H	3.17631	-0.83659	-1.95939
H	-3.01383	3.20581	-0.81727
H	-2.41867	2.49200	0.66811
H	-4.67255	1.60590	-0.52002

H	-3.54476	0.61853	-1.44007
H	-4.40646	-0.60345	0.51553
H	-3.86354	0.68163	1.60485
C	0.55615	1.03647	3.43132
O	0.94376	0.04789	2.49564
H	0.18092	1.93398	2.92599
H	-0.20918	0.65819	4.11787
H	1.45650	1.28437	3.99207
O	-2.88251	-1.93094	-0.97007
C	-2.51746	-2.89635	-1.93713
H	-3.42735	-3.13379	-2.48726
H	-1.76475	-2.49940	-2.62798
H	-2.12882	-3.80477	-1.46291
C	-1.27844	1.97103	-1.08862
C	-0.20052	3.00060	-0.74813
C	1.15778	2.73749	-1.40644
C	3.24221	1.25929	-1.42159
C	1.77645	1.40052	-0.99321
H	-0.92767	0.97364	-0.80395
H	-1.43560	1.94556	-2.17666
H	-0.06391	3.01071	0.34390
H	-0.55598	4.00258	-1.02374
H	1.05500	2.77325	-2.50005
H	1.84699	3.54869	-1.13504
H	1.69246	1.28822	0.09444
H	1.19589	0.57491	-1.42922
H	3.33669	1.60895	-2.45839
H	3.86686	1.92978	-0.81530

[14]PCP

M06-2X/6-31G(d) single-point energy: -1119.932189

M06-2X/6-31G(d) zero-point correction: 0.511462

M06-2X/6-31G(d) thermal correction to enthalpy: 0.025717

B2PLYP-D3BJ/def2-TZVP single-point energy: -1119.912557

Final enthalpy: -1119.375378

Cartesian coordinates

C	-1.28511	-0.18080	1.66981
C	-2.25199	-0.84404	0.92008
C	-1.83430	-1.77375	-0.04686
C	-0.47951	-2.01171	-0.22849
C	0.49312	-1.33505	0.51922
C	0.08679	-0.39900	1.48846
H	-1.62578	0.53250	2.41271
H	-0.12250	-2.71885	-0.96662
C	1.90080	-1.66756	0.15004
O	2.17909	-2.54718	-0.64217
O	2.82179	-0.89319	0.72653
C	0.59761	1.19328	3.17439
O	1.03100	0.25132	2.21092
H	1.50506	1.60016	3.61908
H	0.02506	2.00432	2.70861
H	-0.01056	0.71745	3.95152
O	-2.81642	-2.38067	-0.77356
C	-2.41269	-3.29689	-1.77234
H	-1.86221	-4.13981	-1.33925
H	-3.32870	-3.66114	-2.23629
C	-3.71170	-0.50015	1.10044
H	-1.78815	-2.80814	-2.52888
H	-4.32989	-1.23539	0.58033
H	-3.96544	-0.54983	2.16546

C	4.41034	1.18941	-0.99266
C	3.15243	1.85258	-0.42203
C	1.87478	1.58442	-1.21674
C	0.67693	2.33297	-0.63621
C	-0.66161	1.96899	-1.27580
C	-1.80630	2.80775	-0.70560
C	-3.19194	2.48457	-1.27716
C	-4.01184	0.91283	0.57226
C	4.37544	-0.34010	-1.08555
C	4.16915	-1.04968	0.24707
C	-3.70426	1.08357	-0.92414
H	-3.42082	1.62571	1.15937
H	-5.06224	1.15592	0.76883
H	-2.95987	0.34260	-1.23831
H	-4.60650	0.86516	-1.50688
H	-3.15584	2.59318	-2.36897
H	-3.91268	3.23018	-0.91549
H	-1.81126	2.68832	0.38738
H	-1.58727	3.86788	-0.88946
H	-0.60731	2.11387	-2.36453
H	-0.85052	0.89901	-1.10911
H	0.84666	3.41552	-0.72794
H	0.62141	2.12015	0.44106
H	2.02001	1.87421	-2.26760
H	1.65047	0.50980	-1.22339
H	3.32160	2.93750	-0.38770
H	2.99166	1.52686	0.61279
H	4.59922	1.58953	-1.99732
H	5.27309	1.47995	-0.37876
H	5.33017	-0.68937	-1.49652

H	3.60065	-0.67630	-1.78351
H	4.39219	-2.11538	0.16569
H	4.78993	-0.60779	1.03070

[4.4]PCP 25 benzene dimer

Cartesian coordinates

C	0.19911	2.15965	0.01021
C	1.54324	1.89189	0.23049
C	2.12303	0.77751	-0.39903
C	1.33491	-0.06009	-1.16871
C	-0.04254	0.16836	-1.32872
C	-0.61705	1.31231	-0.75581
H	-0.22921	3.03285	0.48956
H	1.73554	-0.96172	-1.61490
C	-1.14874	0.01242	2.21352
C	0.12160	-0.55803	2.11916
C	0.26993	-1.78833	1.46529
C	-0.81004	-2.37778	0.82575
C	-2.06655	-1.75870	0.81015
C	-2.21614	-0.58083	1.53831
H	-1.31645	0.92681	2.77132
H	1.25796	-2.23695	1.43083
H	-0.65974	-3.30753	0.28111
H	-3.18676	-0.09047	1.56221
C	-0.78871	-0.97872	-1.91861
O	-0.22708	-1.93582	-2.41187
O	-2.12581	-0.91561	-1.77827
O	1.27125	-0.01792	2.60336
C	-2.53077	2.60373	-0.21305
O	-1.93488	1.55133	-0.94586

H	-2.38470	2.46451	0.86472
H	-2.13146	3.57934	-0.51228
H	-3.59470	2.56283	-0.44491
O	3.45138	0.58344	-0.15632
C	4.03851	-0.58515	-0.69308
H	3.54993	-1.48607	-0.30363
H	3.98385	-0.59044	-1.78782
H	5.08251	-0.57222	-0.38147
H	-2.90013	-2.20046	0.27994
H	2.15295	2.53602	0.85435
H	1.08827	0.86157	2.96798
H	-2.38546	-0.07091	-1.36034

[4.5]PCP 26 benzene dimer

Cartesian coordinates

C	0.36772	-0.78221	1.97113
C	1.67562	-1.00336	1.55793
C	2.27153	-0.06257	0.69751
C	1.52531	0.99744	0.21360
C	0.17983	1.17466	0.57117
C	-0.40274	0.28950	1.49140
H	-0.06939	-1.48870	2.66818
H	1.93891	1.70194	-0.49663
C	-1.63816	-1.95481	-0.60686
C	-0.37974	-1.90485	-1.20948
C	-0.10640	-0.90235	-2.14703
C	-1.05039	0.08058	-2.41084
C	-2.28380	0.09906	-1.74581
C	-2.56191	-0.94366	-0.86575
H	-1.89691	-2.75141	0.08131

H	0.86877	-0.89009	-2.62390
H	-0.80357	0.87696	-3.10981
H	-3.51616	-0.95977	-0.34426
C	-0.53603	2.19889	-0.24003
O	0.04254	2.92034	-1.02791
O	-1.87356	2.20823	-0.09871
O	0.63090	-2.77436	-0.93870
C	-2.29792	-0.48195	2.69132
O	-1.68175	0.49813	1.88010
H	-2.28092	-1.46403	2.20390
H	-1.81565	-0.55297	3.67281
H	-3.33018	-0.15687	2.81894
O	3.58254	-0.28082	0.38654
C	4.18661	0.61130	-0.52895
H	4.18757	1.63685	-0.14226
H	5.21338	0.26882	-0.65407
H	3.67289	0.59191	-1.49724
H	-3.00376	0.88486	-1.93269
H	2.24672	-1.85203	1.91583
H	-2.14919	1.55119	0.57105
H	1.43495	-2.46284	-1.37756

[5.5]PCP benzene dimer

Cartesian coordinates

C	0.32102	-1.78194	-1.25700
C	1.64652	-1.36840	-1.30366
C	2.19271	-0.74697	-0.16603
C	1.39307	-0.50686	0.93879
C	0.03830	-0.86743	0.95700
C	-0.50629	-1.53264	-0.15175

H	-0.08019	-2.29344	-2.12512
H	1.77550	-0.00064	1.81638
C	-1.46771	1.19034	-1.69635
C	-0.16130	1.64663	-1.51548
C	0.16997	2.33055	-0.34099
C	-0.78321	2.51838	0.64897
C	-2.08900	2.02927	0.50688
C	-2.40671	1.37332	-0.68011
H	-1.76182	0.68782	-2.61112
H	1.18792	2.68866	-0.22058
H	-0.50231	3.04857	1.55642
H	-3.41315	0.98637	-0.82177
C	-0.71100	-0.40779	2.15976
O	-0.15894	0.04226	3.14606
O	-2.04006	-0.48302	2.04900
O	0.84741	1.47674	-2.41382
C	-2.37764	-2.43010	-1.29986
O	-1.80451	-1.91485	-0.11518
H	-1.92441	-3.38603	-1.58563
H	-3.43332	-2.58415	-1.07796
H	-2.27766	-1.71753	-2.12737
O	3.51526	-0.41689	-0.24012
C	4.07658	0.25410	0.87028
H	4.02487	-0.36256	1.77499
H	5.11968	0.44104	0.61680
H	3.56745	1.20734	1.05486
H	2.26454	-1.54544	-2.17615
H	-2.82130	2.15352	1.29525
H	-2.29541	-0.87044	1.18895
H	1.68441	1.71026	-1.98805

[6.5]PCP 2 benzene dimer

Cartesian coordinates

C	-0.43773	-0.04979	2.07919
C	-1.70660	0.42899	1.77965
C	-2.32404	-0.00856	0.59324
C	-1.64268	-0.85115	-0.26677
C	-0.34737	-1.30569	0.02145
C	0.26894	-0.90343	1.21690
H	0.01728	0.26991	3.01004
H	-2.07911	-1.18059	-1.20121
C	1.86998	1.98317	0.30395
C	0.61326	2.17147	-0.27795
C	0.37132	1.67464	-1.56179
C	1.37304	1.00112	-2.25112
C	2.63726	0.79505	-1.68446
C	2.85445	1.29185	-0.39825
H	2.09618	2.37950	1.28711
H	-0.61019	1.82635	-2.00028
H	1.17052	0.64058	-3.25709
H	3.82863	1.15278	0.06669
C	0.26895	-2.13312	-1.05136
O	-0.26870	-2.31122	-2.12841
O	1.45297	-2.67067	-0.73944
O	-0.41436	2.83163	0.32303
C	2.16395	-0.83221	2.64432
O	1.51997	-1.34116	1.49322
H	2.23025	0.26123	2.60921
H	1.64800	-1.13840	3.56143
H	3.16725	-1.25756	2.63525

O	-3.59444	0.44113	0.37725
C	-4.22186	0.05192	-0.82837
H	-4.32958	-1.03728	-0.88639
H	-5.20809	0.51504	-0.82055
H	-3.65785	0.40506	-1.69959
H	-2.23050	1.10042	2.44989
H	3.41466	0.26670	-2.22372
H	1.75731	-2.36081	0.13647
H	-1.22350	2.67393	-0.18432

[6.5]PCP 13 benzene dimer

Cartesian coordinates

C	1.80963	-1.41236	-1.32387
C	0.47111	-1.39889	-0.94082
C	-0.20365	-0.18605	-0.70712
C	0.56378	0.97262	-0.77458
C	1.92185	0.99010	-1.09531
C	2.55691	-0.23357	-1.39738
H	2.25960	-2.37740	-1.51848
H	0.09181	1.93085	-0.58134
C	2.13301	0.25123	2.29345
C	2.37259	-1.08616	1.96804
C	3.67458	-1.50076	1.67846
C	4.71065	-0.56950	1.70480
C	4.49667	0.76965	2.03605
C	3.18431	1.16009	2.33134
H	1.11483	0.55716	2.51385
H	3.89368	-2.53776	1.44987
H	5.72062	-0.90417	1.47619
H	2.97927	2.19155	2.60841

C	2.57964	2.31949	-0.99945
O	2.01656	3.29626	-0.54266
O	3.84007	2.35496	-1.44655
O	1.28931	-1.91303	1.96821
C	4.50679	-1.47321	-1.92627
O	3.86408	-0.22777	-1.72229
H	5.55050	-1.23703	-2.13038
H	4.43974	-2.09880	-1.02947
H	4.07794	-2.00631	-2.78163
C	-1.66152	-0.09826	-0.44208
C	-2.58069	-0.86478	-1.17264
C	-2.16335	0.77890	0.52098
C	-3.94376	-0.74855	-0.94041
H	-2.21768	-1.55547	-1.92824
C	-3.53445	0.89324	0.74699
H	-1.47292	1.37295	1.11442
C	-4.45608	0.13293	0.02369
H	-4.62491	-1.35630	-1.53062
H	-3.87437	1.58662	1.50852
C	-5.96803	0.22368	0.24284
C	-6.50326	-1.15287	0.67253
H	-7.58665	-1.10322	0.82857
H	-6.30700	-1.91614	-0.08675
H	-6.03521	-1.47567	1.60841
C	-6.64880	0.64671	-1.07008
H	-6.46028	-0.07290	-1.87266
H	-7.73290	0.71433	-0.92622
H	-6.28350	1.62546	-1.39806
C	-6.33383	1.24361	1.32580
H	-7.42166	1.27506	1.44411

H	-5.89898	0.97650	2.29475
H	-5.99577	2.25100	1.06081
O	-0.14171	-2.61726	-0.83903
H	5.31339	1.48131	2.06245
H	0.49140	-1.37766	2.08632
H	-1.01510	-2.49916	-0.43071
H	4.14305	1.46613	-1.71339

[6.3]PCP 28 benzene dimer

Cartesian coordinates

C	-2.39672	-1.89729	-0.60575
C	-1.03303	-1.64449	-0.70594
C	-0.36696	-0.81438	0.21173
C	-1.17103	-0.13176	1.12098
C	-2.55750	-0.28819	1.18914
C	-3.17651	-1.23527	0.34375
H	-2.83146	-2.56919	-1.33467
H	-0.70541	0.56200	1.81172
C	-2.52366	1.18444	-1.98481
C	-1.23757	0.87132	-2.42894
C	-0.19937	1.74253	-2.08518
C	-0.43983	2.86787	-1.30499
C	-1.72606	3.17920	-0.84910
C	-2.76689	2.32043	-1.21267
H	-3.35108	0.52686	-2.24264
H	0.81382	1.53910	-2.42598
H	0.38782	3.52624	-1.04810
H	-3.78486	2.53943	-0.89840
C	-3.35325	0.64605	2.02454
O	-4.55669	0.77494	1.99585

O	-2.56134	1.42562	2.79484
C	-5.11648	-2.36927	-0.40498
O	-4.50190	-1.43551	0.46650
H	-6.16805	-2.38950	-0.12290
H	-5.02328	-2.05371	-1.44997
H	-4.68325	-3.36773	-0.28400
C	1.10440	-0.63091	0.21710
C	1.67343	0.62089	0.48682
C	1.97116	-1.70471	-0.00838
C	3.05182	0.78506	0.52459
H	1.02558	1.47852	0.64623
C	3.35298	-1.53196	0.02719
H	1.55960	-2.68736	-0.21653
C	3.92689	-0.28543	0.29382
H	3.45175	1.77439	0.73199
H	3.98309	-2.39648	-0.15149
C	5.43928	-0.05536	0.33985
C	5.82827	0.98688	-0.72250
H	5.55899	0.63763	-1.72484
H	5.32754	1.94454	-0.55019
H	6.90949	1.16281	-0.69848
C	6.22739	-1.34017	0.06588
H	6.00635	-1.74457	-0.92759
H	7.30002	-1.12586	0.10942
H	6.00943	-2.11320	0.81030
C	5.83293	0.46654	1.73224
H	6.91402	0.63922	1.77701
H	5.33128	1.41064	1.96596
H	5.56811	-0.26008	2.50738
O	-0.38724	-2.22827	-1.76352

H	-1.05306	-0.01751	-3.02335
H	-1.90892	4.06067	-0.24408
H	0.50994	-1.85940	-1.82235
H	-3.15220	2.03608	3.26980

[5.5]PCP 27 benzene dimer

Cartesian coordinates

C	2.05712	2.20057	-0.31161
C	0.68063	1.97996	-0.38375
C	0.07940	0.93173	0.33614
C	0.92648	0.09634	1.06225
C	2.30921	0.26140	1.11796
C	2.88293	1.34335	0.41610
H	2.47926	3.04371	-0.84128
H	0.49315	-0.73931	1.60029
C	3.33915	-0.92299	-1.91647
C	2.00346	-0.81701	-2.31991
C	1.11226	-1.78735	-1.85871
C	1.53772	-2.80958	-1.01087
C	2.86837	-2.91070	-0.60489
C	3.76753	-1.95054	-1.08239
H	4.05626	-0.17623	-2.25357
H	0.06583	-1.74547	-2.14944
H	0.81658	-3.54306	-0.65591
H	4.81356	-1.99402	-0.78723
C	3.14586	-0.73604	1.83172
O	4.31113	-0.60937	2.13739
O	2.44546	-1.85665	2.10351
C	4.80579	2.51021	-0.33336
O	4.22176	1.49118	0.45736

H	5.88164	2.41350	-0.19441
H	4.55827	2.37804	-1.39319
H	4.48358	3.50401	-0.00427
O	-0.11941	2.78331	-1.14085
C	-1.37255	0.63828	0.26200
C	-2.34631	1.64588	0.30456
C	-1.81207	-0.68496	0.15997
C	-3.69694	1.33043	0.25017
H	-2.03866	2.68264	0.38687
C	-3.17003	-0.99485	0.10572
H	-1.07843	-1.48571	0.10455
C	-4.14436	0.00477	0.14838
H	-4.42044	2.14071	0.29202
H	-3.45757	-2.03728	0.02135
C	-5.64516	-0.28899	0.08747
C	-6.25366	0.41640	-1.13651
H	-5.79085	0.05618	-2.06128
H	-7.32970	0.21643	-1.18941
H	-6.11473	1.50072	-1.08698
C	-5.93544	-1.78883	-0.02686
H	-7.01768	-1.94873	-0.06689
H	-5.49968	-2.21534	-0.93662
H	-5.54649	-2.34142	0.83494
C	-6.31979	0.23872	1.36519
H	-5.90405	-0.24941	2.25286
H	-6.18357	1.31885	1.47633
H	-7.39622	0.03630	1.33325
H	3.19453	-3.70549	0.05670
H	1.67274	-0.00890	-2.96362
H	3.04732	-2.44828	2.58878

H 0.42621 3.44250 -1.59687

Fully Optimized StrainViz Fragments of [4.4]PCP 25

Parent

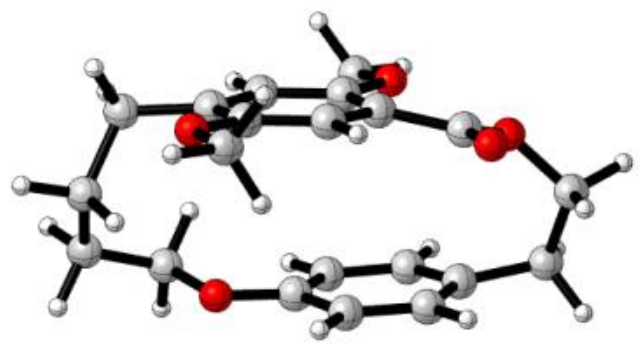

Fragment 1

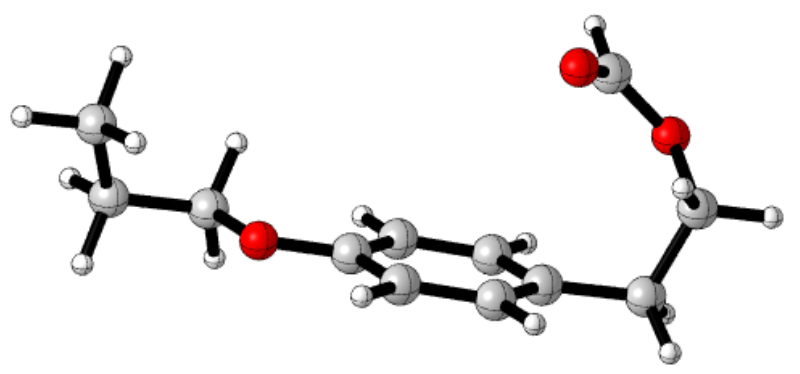

Fragment 2

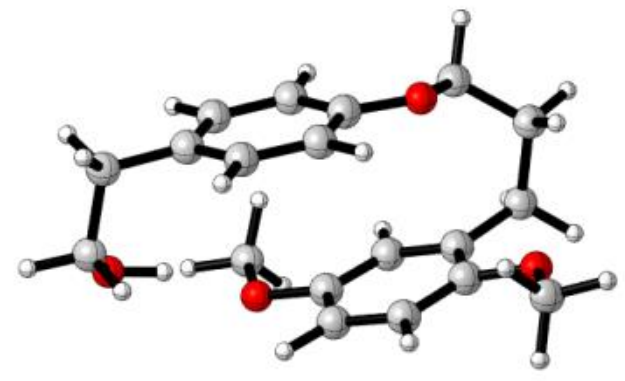

Fragment 3

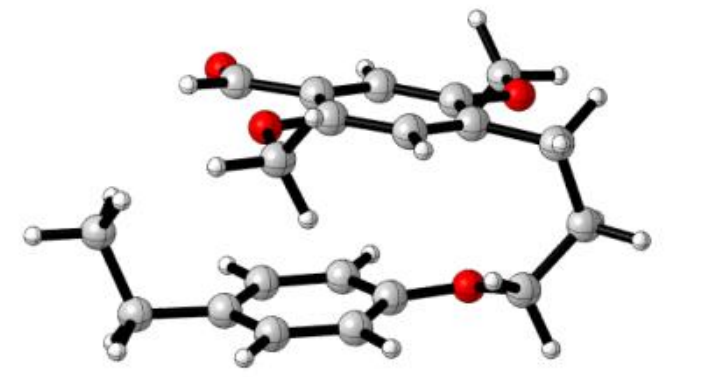

Fragment 4

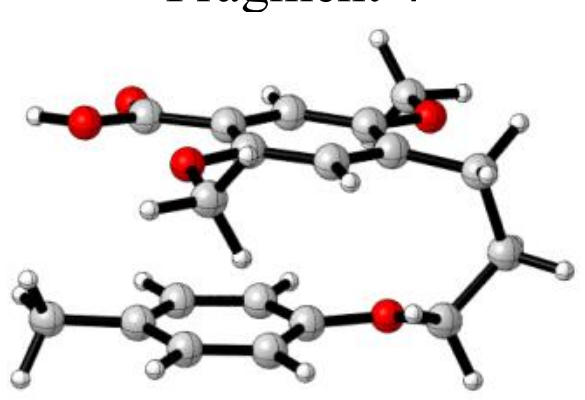

Fragment 5

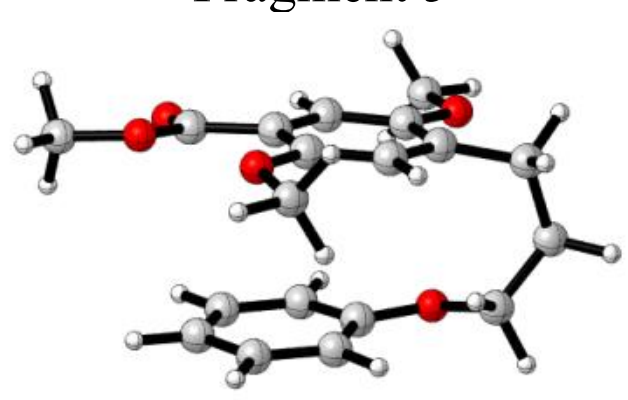

Fragment 6

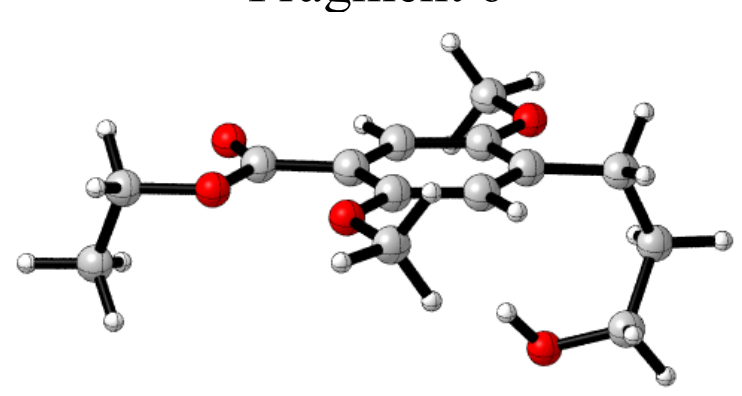

Fragment 7

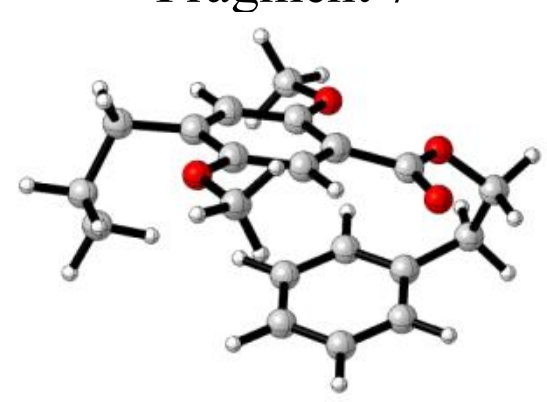

Fragment 8

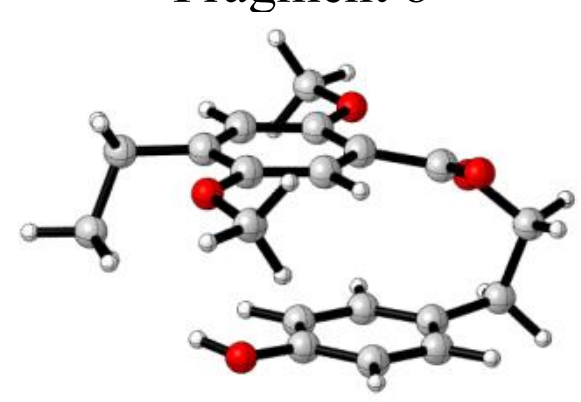

Fragment 9

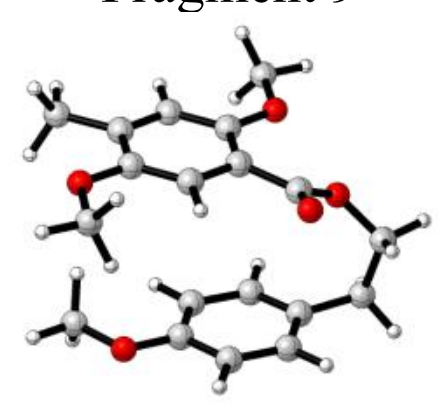

Fragment 10

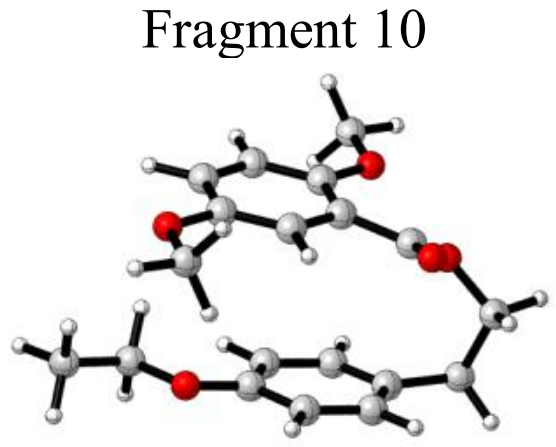

[4.4]PCP 25 StrainViz fragment r1

Cartesian coordinates

C	-0.49650	-0.46040	-1.11840
C	-1.17110	-0.66590	0.08560
C	-0.46060	-1.10330	1.21070
C	0.90420	-1.32410	1.12790
C	1.60020	-1.12390	-0.07080
C	0.87910	-0.69230	-1.18110
H	-1.02240	-0.12820	-2.00560
H	-1.00380	-1.26020	2.13700
H	1.44430	-1.65780	2.01110
H	1.39770	-0.52920	-2.12280
C	2.59610	1.95020	-0.19700
O	2.10910	2.01050	0.90160
O	3.48150	1.04650	-0.62470
C	3.83360	-0.01860	0.27480
C	3.09720	-1.29200	-0.13680
C	-4.83900	1.19840	0.72160
C	-4.70190	0.14950	-0.37880
C	-3.26740	-0.01750	-0.84470
O	-2.50540	-0.47750	0.26300
H	4.91410	-0.14170	0.18100
H	3.58300	0.27470	1.29570
H	3.40490	-1.55930	-1.15320
H	3.42720	-2.09770	0.52860
H	-3.20030	-0.74270	-1.66650
H	-2.86250	0.94160	-1.19810
H	-5.07030	-0.82120	-0.02850
H	-5.30720	0.42750	-1.24860
H	-5.88020	1.30320	1.03790
H	-4.49170	2.17590	0.37040
H	-4.24180	0.92230	1.59430

H	2.38390	2.65740	-1.00980

[4.4]PCP 25 StrainViz fragment r2

Cartesian coordinates

C	0.88750	-0.78290	-1.54370
C	2.09880	-0.65330	-0.86910
C	2.21750	-1.18640	0.42300
C	1.14110	-1.85140	1.00890
C	-0.06900	-1.96040	0.32650
C	-0.20460	-1.41290	-0.94200
H	0.80450	-0.36270	-2.54090
H	1.22170	-2.27370	2.00370
C	-0.83250	1.96430	-0.48990
C	0.11130	1.70670	0.50590
C	-0.31020	1.16820	1.72730
C	-1.64600	0.86350	1.93290
C	-2.60520	1.08460	0.93620
C	-2.17120	1.63350	-0.26960
H	-0.54630	2.42020	-1.43120
H	0.43750	0.98070	2.49160
H	-1.95540	0.44230	2.88720
H	-2.89730	1.82790	-1.05600
O	-4.02450	-1.08700	-0.51410
C	-4.30040	-0.77620	0.83450
C	-4.04770	0.70120	1.15150
C	3.23760	0.12050	-1.48680
C	3.38030	1.54740	-0.92120
C	2.04610	2.28060	-0.83700
O	1.44640	1.93820	0.40850
H	-5.35550	-1.01350	1.00460

H	-3.70470	-1.40290	1.51600
H	-4.69630	1.30540	0.50760
H	-4.34070	0.89420	2.19060
H	2.17480	3.36840	-0.87550
H	1.40430	1.98170	-1.67260
H	3.81130	1.51390	0.08380
H	4.06940	2.11320	-1.55710
H	4.18080	-0.41570	-1.34270
H	3.06260	0.18850	-2.56670
C	-1.62790	-0.90620	-2.78970
O	-1.43720	-1.52990	-1.53140
H	-1.40970	0.16600	-2.73110
H	-0.99840	-1.37020	-3.55660
H	-2.67890	-1.05320	-3.03920
O	3.42730	-0.99970	1.02880
C	3.56740	-1.47050	2.35320
H	2.84150	-0.99260	3.02190
H	3.44310	-2.55850	2.40540
H	4.57760	-1.20670	2.66550
H	-0.92130	-2.45360	0.78380
H	-3.05770	-1.09410	-0.63190

[4.4]PCP 25 StrainViz fragment r3

Cartesian coordinates

C	0.87340	-0.22580	1.73130
C	2.04470	0.06180	1.04180
C	2.07590	1.16300	0.16140
C	0.94750	1.94690	-0.00260
C	-0.23490	1.64730	0.69010
C	-0.28240	0.54750	1.55350

H	0.86570	-1.07920	2.40080
H	0.92520	2.79670	-0.67580
C	-0.74890	-2.31790	-0.49820
C	0.09550	-1.47390	-1.22060
C	-0.45160	-0.39810	-1.92910
C	-1.81270	-0.14330	-1.87060
C	-2.67340	-0.95400	-1.11860
C	-2.11690	-2.04150	-0.44950
H	-0.36360	-3.18810	0.02160
H	0.21950	0.24240	-2.49290
H	-2.21720	0.70930	-2.41260
H	-2.76400	-2.70130	0.12450
C	-1.43450	2.46240	0.44480
O	-1.49020	3.34920	-0.38880
C	-4.41560	0.58750	-0.14100
C	-4.14680	-0.63770	-1.02090
C	3.25130	-0.83240	1.17790
C	3.44420	-1.77770	-0.02490
C	2.15190	-2.47600	-0.43090
O	1.44720	-1.59980	-1.30470
H	-5.48860	0.77560	-0.03970
H	-3.95390	1.48170	-0.57330
H	-4.67700	-1.50550	-0.61330
H	-4.55610	-0.46000	-2.02260
H	2.34480	-3.41830	-0.95640
H	1.55450	-2.69680	0.46090
H	3.81650	-1.21940	-0.88860
H	4.19910	-2.52690	0.23550
H	4.15330	-0.22520	1.30140
H	3.13430	-1.43660	2.08430

C	-1.56000	-0.88880	2.94710
O	-1.46390	0.29030	2.16860
H	-1.31830	-1.77180	2.34540
H	-0.89880	-0.84210	3.81940
H	-2.59630	-0.94430	3.27940
O	3.25990	1.36450	-0.48290
C	3.30800	2.42330	-1.41880
H	2.57260	2.27550	-2.21840
H	3.12470	3.38930	-0.93460
H	4.31270	2.41220	-1.84010
H	-2.31220	2.22390	1.06710
H	-3.98900	0.44030	0.85710

[4.4]PCP 25 StrainViz fragment r4

Cartesian coordinates

C	0.76270	-0.41540	1.73830
C	1.89210	0.11570	1.13280
C	1.75900	1.27700	0.35140
C	0.51220	1.85390	0.18890
C	-0.63610	1.29700	0.77630
C	-0.51430	0.14330	1.56380
H	0.88200	-1.31150	2.33690
H	0.36790	2.73040	-0.43050
C	-0.49240	-2.24150	-0.77840
C	0.32110	-1.31830	-1.44270
C	-0.27230	-0.26180	-2.13720
C	-1.64890	-0.08370	-2.09900
C	-2.47340	-0.94960	-1.37550
C	-1.87030	-2.03780	-0.74180
H	-0.06860	-3.10700	-0.28080

H	0.37160	0.43620	-2.66320
H	-2.08790	0.76810	-2.61260
H	-2.49010	-2.74650	-0.19620
C	-1.91230	1.96100	0.40820
O	-1.98200	2.87260	-0.39400
O	-3.00960	1.49770	1.02460
C	-3.96120	-0.72520	-1.27800
C	3.21990	-0.58980	1.23670
C	3.59020	-1.36700	-0.04400
C	2.42980	-2.18610	-0.60250
O	1.68130	-1.34430	-1.47500
H	-4.27040	-0.63550	-0.23160
H	-4.52450	-1.55340	-1.72130
H	2.78560	-3.04870	-1.17670
H	1.80370	-2.55490	0.21670
H	3.91770	-0.67300	-0.82360
H	4.43170	-2.03060	0.18110
H	4.01350	0.13140	1.45590
H	3.17400	-1.29340	2.07510
C	-1.49600	-1.64160	2.77870
O	-1.62560	-0.40320	2.10780
H	-1.06880	-2.40420	2.11740
H	-0.87720	-1.54750	3.67810
H	-2.50690	-1.93130	3.06450
O	2.90800	1.74210	-0.21760
C	2.79480	2.86530	-1.06840
H	2.12930	2.65440	-1.91390
H	2.41940	3.73970	-0.52440
H	3.79920	3.07040	-1.43740
H	-3.75150	2.02040	0.66960

H	-4.25270	0.19430	-1.79380

[4.4]PCP 25 StrainViz fragment r5

Cartesian coordinates

C	-0.72560	-0.42620	-1.71870
C	-1.77090	0.34920	-1.23860
C	-1.47130	1.49350	-0.47940
C	-0.15190	1.80780	-0.20720
C	0.90490	1.00270	-0.66350
C	0.61880	-0.13270	-1.43560
H	-0.97400	-1.30380	-2.30480
H	0.11390	2.66090	0.40470
C	-0.10730	-2.42000	0.96550
C	-0.73000	-1.29150	1.50640
C	0.02860	-0.35950	2.22370
C	1.40230	-0.51780	2.34160
C	2.04080	-1.61020	1.75170
C	1.27650	-2.55970	1.08130
H	-0.67970	-3.18500	0.45280
H	-0.48020	0.50220	2.64440
H	1.98080	0.23080	2.87470
H	1.75480	-3.42920	0.63900
C	2.24960	1.40050	-0.16390
O	2.41570	2.29080	0.64800
O	3.27020	0.70330	-0.67100
C	4.55060	1.04390	-0.14090
C	-3.20100	-0.08050	-1.44240
C	-3.82200	-0.70320	-0.17440
C	-2.90040	-1.71290	0.50330
O	-2.05200	-0.99530	1.39560

H	5.25980	0.38450	-0.63810
H	4.78740	2.08930	-0.34890
H	-3.46550	-2.45290	1.08100
H	-2.31070	-2.24410	-0.25120
H	-4.05790	0.08010	0.55190
H	-4.76210	-1.19310	-0.44830
H	-3.81150	0.77230	-1.75530
H	-3.23030	-0.82050	-2.24980
C	1.30880	-2.13160	-2.51030
O	1.63510	-0.91690	-1.86400
H	0.67730	-2.76020	-1.87200
H	0.80270	-1.95470	-3.46610
H	2.25800	-2.63490	-2.69270
O	-2.54520	2.21040	-0.03920
C	-2.27750	3.33060	0.78050
H	-1.75260	3.03330	1.69600
H	-1.67750	4.07770	0.24820
H	-3.24600	3.75830	1.03790
H	3.11720	-1.72560	1.82740
H	4.56890	0.88200	0.93950

[4.4]PCP 25 StrainViz fragment r6

Cartesian coordinates

C	-0.88600	-1.22560	-0.77370
C	-1.81570	-0.19040	-0.73290
C	-1.35950	1.12060	-0.50970
C	-0.00530	1.35290	-0.32940
C	0.93190	0.30980	-0.36220
C	0.48940	-1.00610	-0.59090
H	-1.25490	-2.22860	-0.95700

H	0.38030	2.34920	-0.15270
C	2.34680	0.73880	-0.14510
O	2.65600	1.90260	0.02140
O	3.23750	-0.25420	-0.15170
C	4.60850	0.12280	0.06860
C	4.88710	0.32790	1.54590
C	-3.29100	-0.46300	-0.88030
C	-4.06640	-0.36630	0.44530
C	-3.56210	-1.31480	1.53140
O	-2.38260	-0.86040	2.17590
H	5.18440	-0.71170	-0.33380
H	4.82570	1.02410	-0.50810
H	4.63020	-0.57200	2.11060
H	5.94920	0.54020	1.69620
H	-4.31410	-1.39790	2.32140
H	-3.41760	-2.32350	1.11250
H	-4.01730	0.65760	0.82970
H	-5.11960	-0.59110	0.24020
H	-3.71750	0.25220	-1.59120
H	-3.42560	-1.46610	-1.30090
C	0.92420	-3.32630	-0.85190
O	1.39440	-2.00900	-0.62950
H	0.23680	-3.64210	-0.05950
H	0.42720	-3.41310	-1.82420
H	1.80920	-3.96110	-0.83880
O	-2.31740	2.08800	-0.48760
C	-1.89050	3.41740	-0.25560
H	-1.40160	3.51000	0.72060
H	-1.20240	3.75390	-1.03920
H	-2.79030	4.03110	-0.27250

H	-1.66080	-0.90060	1.53140
H	4.30650	1.16870	1.93150

[4.4]PCP 25 StrainViz fragment r7

Cartesian coordinates

C	-1.43290	-0.98750	-1.32100
C	-2.45950	-0.19070	-0.83030
C	-2.16510	1.12980	-0.44880
C	-0.86520	1.59820	-0.54530
C	0.17480	0.78780	-1.02580
C	-0.11360	-0.52580	-1.43690
H	-1.67930	-2.00180	-1.61340
H	-0.59800	2.59840	-0.22880
C	0.92150	-1.73350	1.68880
C	0.56380	-0.75800	2.61690
C	1.38210	0.35710	2.79410
C	2.54420	0.49880	2.03980
C	2.90640	-0.46810	1.09680
C	2.08490	-1.58560	0.93630
H	0.29270	-2.60860	1.54760
H	1.11260	1.12030	3.51790
H	3.16840	1.38020	2.16980
H	2.35400	-2.33820	0.19950
C	1.53080	1.39830	-0.92870
O	1.71680	2.49550	-0.44210
O	2.52660	0.62720	-1.39310
C	3.82500	0.85060	-0.82940
C	4.09770	-0.26410	0.18860
C	-3.85140	-0.73650	-0.64860
C	-4.16220	-1.05200	0.82530

C	-3.22100	-2.09710	1.41920
H	4.54090	0.82090	-1.65280
H	3.85190	1.83710	-0.36510
H	4.31040	-1.19520	-0.34550
H	4.99490	0.00330	0.75870
H	-3.49340	-2.33230	2.45220
H	-3.25460	-3.02800	0.84110
H	-4.10390	-0.12370	1.40350
H	-5.19770	-1.40470	0.89150
H	-4.58000	-0.00890	-1.02060
H	-3.95980	-1.65010	-1.24430
C	0.60740	-2.67380	-2.13820
O	0.88180	-1.30300	-1.92440
H	0.23920	-3.15020	-1.22140
H	-0.12290	-2.81880	-2.94200
H	1.55620	-3.12490	-2.42820
O	-3.21640	1.86760	0.01280
C	-2.94820	3.20070	0.40100
H	-2.23520	3.23580	1.23270
H	-2.55270	3.78530	-0.43750
H	-3.90120	3.62120	0.72070
H	-2.18580	-1.73990	1.41450
H	-0.34520	-0.86690	3.20130

[4.4]PCP 25 StrainViz fragment r8

Cartesian coordinates

C	-1.25950	-2.08960	0.12240
C	-2.26550	-1.17920	-0.19090
C	-1.94830	-0.06330	-0.98040
C	-0.64560	0.11640	-1.42490

C	0.37140	-0.77970	-1.07710
C	0.06200	-1.90970	-0.30240
H	-1.52650	-2.95310	0.72120
H	-0.36170	0.98210	-2.01020
C	0.07070	0.84490	1.90590
C	-0.34270	1.98730	1.22410
C	0.58730	2.75040	0.51520
C	1.91410	2.34020	0.45840
C	2.34340	1.17570	1.10360
C	1.40150	0.44620	1.83140
H	-0.65280	0.24790	2.45640
H	0.25160	3.64450	-0.00120
H	2.62470	2.92290	-0.12360
H	1.70700	-0.47290	2.32590
C	1.74210	-0.32900	-1.45320
O	1.94350	0.50530	-2.31020
O	2.72210	-0.85620	-0.69640
C	3.87090	-0.02470	-0.48520
C	3.73710	0.62890	0.89970
C	-3.66730	-1.35670	0.33440
C	-3.99430	-0.35090	1.44480
O	-1.64330	2.39720	1.20330
H	4.74770	-0.67260	-0.53730
H	3.92700	0.72210	-1.27830
H	3.95140	-0.11810	1.67030
H	4.49580	1.41540	0.98230
H	-3.95730	0.66800	1.04790
H	-4.99790	-0.51890	1.84510
H	-4.38310	-1.22820	-0.48430
H	-3.78260	-2.37690	0.71330

C	0.73650	-3.90790	0.78500
O	1.05610	-2.78430	-0.01260
H	0.36220	-3.60310	1.76900
H	-0.00690	-4.54710	0.29570
H	1.66690	-4.46170	0.90620
O	-2.97640	0.79120	-1.25330
C	-2.65330	1.98980	-1.93370
H	-1.91530	2.56890	-1.36800
H	-2.27120	1.78340	-2.94000
H	-3.58260	2.55410	-2.00840
H	-3.28230	-0.44480	2.27250
H	-2.19430	1.72770	1.63790

[4.4]PCP 25 StrainViz fragment r9

Cartesian coordinates

C	-1.30520	-2.48340	0.50580
C	-2.37620	-1.66540	0.16320
C	-2.15430	-0.58750	-0.70890
C	-0.88090	-0.34810	-1.20220
C	0.20170	-1.15440	-0.82890
C	-0.00980	-2.24630	0.02630
H	-1.49620	-3.32020	1.16760
H	-0.67140	0.49050	-1.85510
C	0.09620	1.45250	1.55040
C	-0.18550	2.54850	0.73600
C	0.82320	3.09520	-0.06700
C	2.09010	2.53320	-0.06980
C	2.39010	1.41970	0.72460
C	1.37760	0.90090	1.52900
H	-0.66890	1.00200	2.17210

H	0.58390	3.95190	-0.68900
H	2.85740	2.95270	-0.71650
H	1.58090	0.02240	2.13690
C	1.52680	-0.66860	-1.31080
O	1.64210	0.06970	-2.26600
O	2.56090	-1.05480	-0.54430
C	3.73570	-0.23490	-0.59670
C	3.71720	0.70880	0.61360
C	-3.75820	-1.90790	0.70220
C	-2.46870	2.57060	1.38320
O	-1.40580	3.14810	0.64660
H	4.59060	-0.91250	-0.55850
H	3.75110	0.31920	-1.53590
H	3.89990	0.12430	1.52090
H	4.54370	1.42010	0.50550
H	-2.26910	2.60690	2.46000
H	-2.64430	1.53200	1.07660
H	-4.46960	-2.07440	-0.11280
H	-3.77380	-2.77560	1.36500
C	0.82040	-4.14600	1.18340
O	1.04690	-3.03800	0.33300
H	0.45780	-3.82590	2.16670
H	0.10360	-4.84750	0.74220
H	1.78630	-4.63700	1.29580
O	-3.24870	0.17720	-0.99300
C	-3.04440	1.31230	-1.81540
H	-2.33010	2.00770	-1.35740
H	-2.68510	1.02020	-2.80870
H	-4.01660	1.79620	-1.90860
H	-4.11550	-1.03460	1.25790

H	-3.35160	3.16870	1.15720

[4.4]PCP 25 StrainViz fragment r10

Cartesian coordinates

C	-0.00290	2.73760	0.58990
C	1.27280	2.47060	0.11110
C	1.48190	1.48680	-0.85610
C	0.38800	0.77530	-1.33670
C	-0.89670	1.01090	-0.83670
C	-1.10680	2.01010	0.12870
H	-0.12850	3.51810	1.33000
H	0.49860	-0.01480	-2.06890
C	0.50190	-1.01120	1.64680
C	1.30150	-1.62380	0.68220
C	0.72410	-2.51730	-0.22910
C	-0.64010	-2.76280	-0.19610
C	-1.46640	-2.13430	0.74410
C	-0.86920	-1.26820	1.65750
H	0.92070	-0.31390	2.36280
H	1.36460	-2.99400	-0.96510
H	-1.07690	-3.43730	-0.92920
H	-1.49230	-0.75400	2.38550
C	-1.92730	0.04200	-1.31620
O	-1.80880	-0.57520	-2.35270
O	-2.93890	-0.15050	-0.45220
C	-3.55040	-1.44790	-0.47590
C	-2.97030	-2.26780	0.68680
C	4.71420	-0.38010	1.02170
C	3.26230	-0.49430	1.43200
O	2.63550	-1.40320	0.53450

H	-4.62480	-1.29500	-0.35950
H	-3.35540	-1.92120	-1.43900
H	-3.40290	-1.91160	1.62690
H	-3.27820	-3.31160	0.55670
H	3.16680	-0.87220	2.45870
H	2.76280	0.48080	1.37570
H	5.19750	-1.36030	1.04110
H	5.24490	0.28460	1.70820
C	-2.57830	3.25750	1.50790
O	-2.37510	2.23750	0.54880
H	-2.04050	3.04160	2.43810
H	-2.26270	4.23400	1.12380
H	-3.64970	3.27360	1.70390
O	2.77080	1.28430	-1.24930
C	2.99040	0.28880	-2.23320
H	2.68330	-0.69560	-1.86500
H	2.45440	0.52610	-3.15940
H	4.06330	0.28690	-2.42640
H	2.12740	3.02820	0.48200
H	4.78580	0.03310	0.01260

Fully Optimized StrainViz Fragments of [4.5]PCP 26

Parent

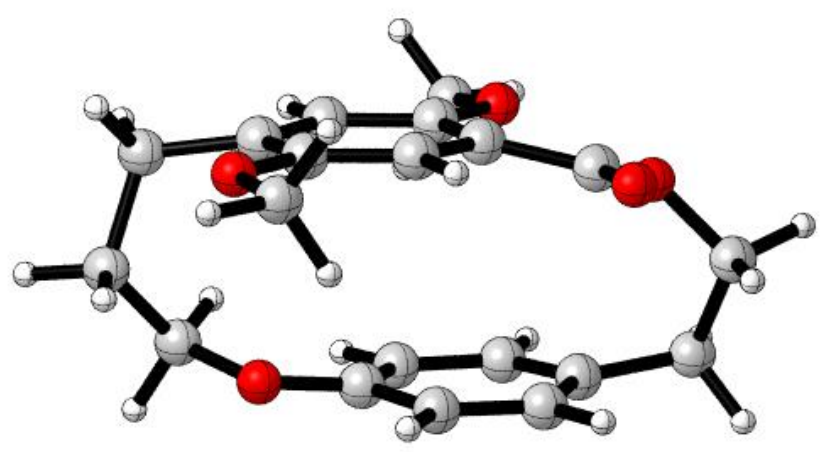

Fragment 1<br>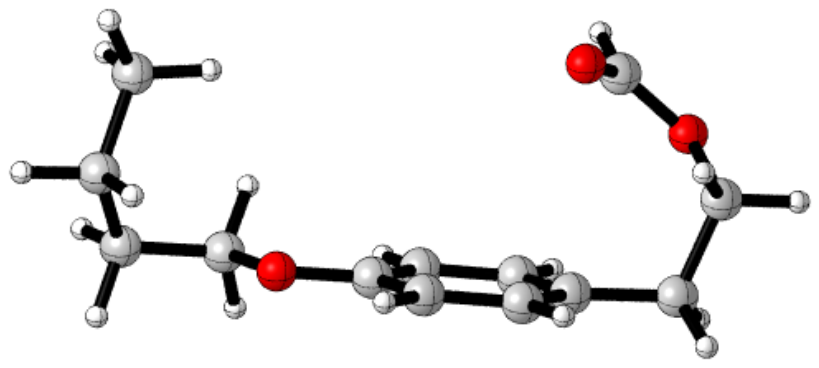	Fragment 2<br>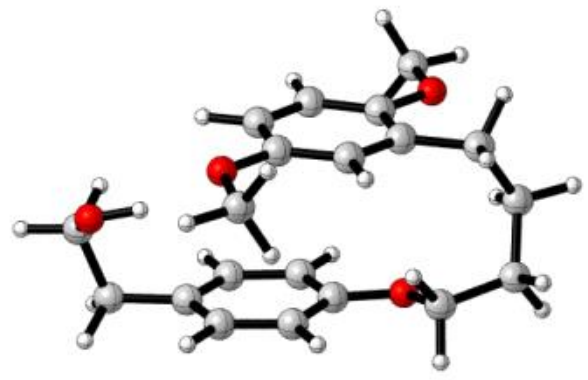	Fragment 3<br>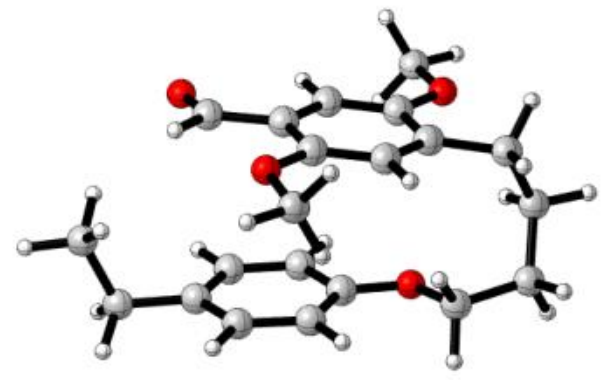
Fragment 4<br>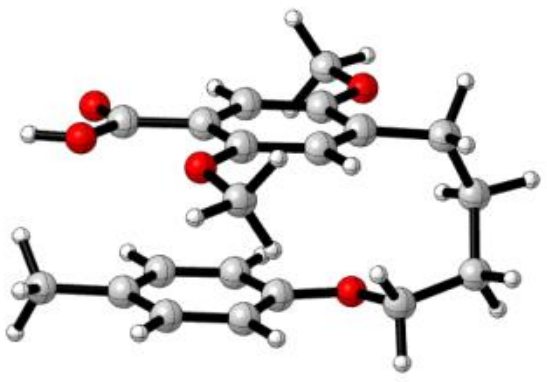	Fragment 5<br>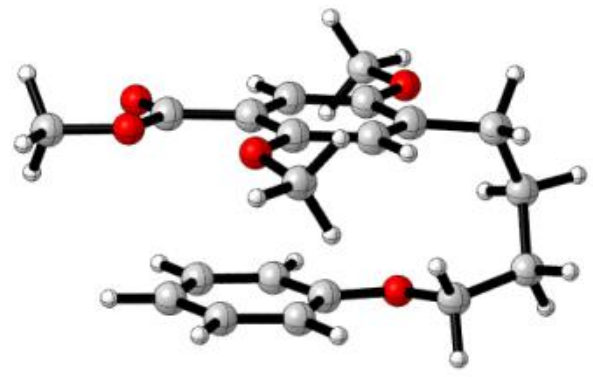	Fragment 6<br>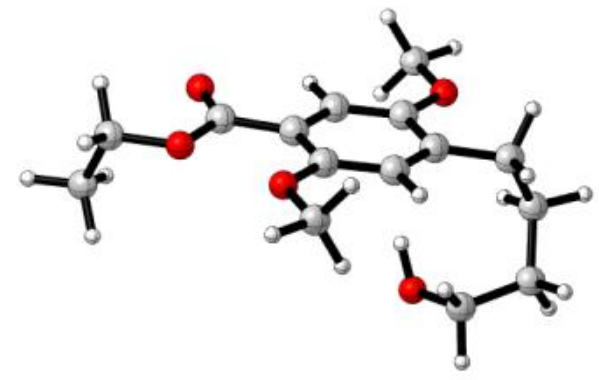
Fragment 7<br>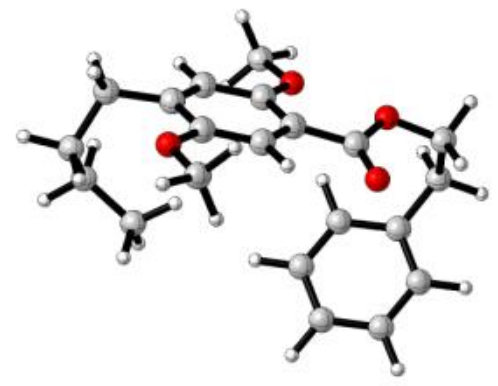	Fragment 8<br>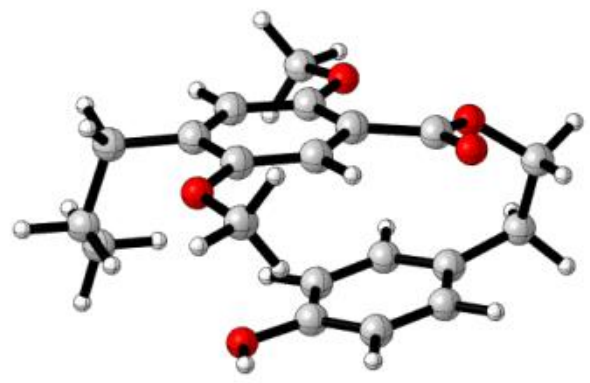	Fragment 9<br>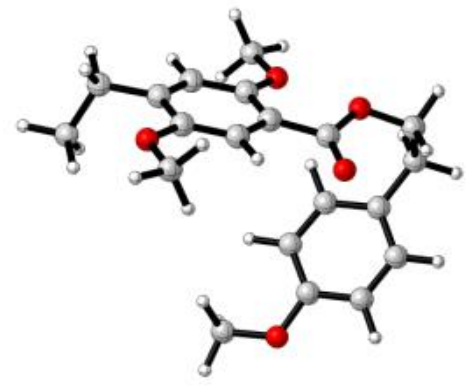
Fragment 10<br>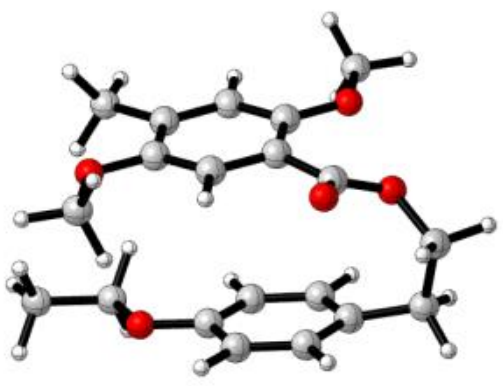	Fragment 11<br>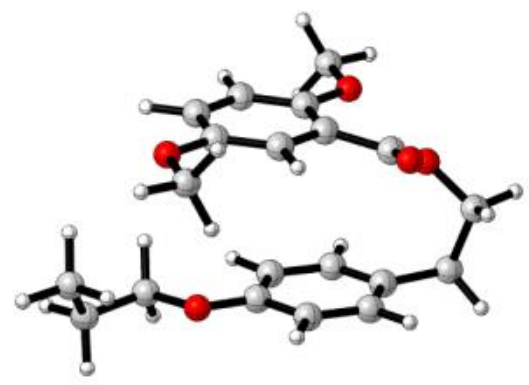	

[4.5]PCP 26 StrainViz fragment r1

Cartesian coordinates

C	-0.13350	-0.77710	-1.09780
C	-0.81180	-0.89580	0.11570
C	-0.08880	-1.12790	1.29270
C	1.29120	-1.23600	1.25070
C	1.99120	-1.12150	0.04300
C	1.25820	-0.89130	-1.11810
H	-0.66820	-0.60250	-2.02390
H	-0.63520	-1.21900	2.22600
H	1.84000	-1.41110	2.17330
H	1.78000	-0.79640	-2.06750

C	2.70760	1.99190	-0.38330
O	2.19180	2.11060	0.69740
O	3.68340	1.13980	-0.70770
C	4.11250	0.20600	0.29800
C	3.49830	-1.16300	0.01250
C	-2.93580	-0.54210	-0.90500
O	-2.16120	-0.80820	0.25590
H	5.20110	0.17150	0.22590
H	3.81960	0.57690	1.28190
H	3.84850	-1.50490	-0.96710
H	3.88310	-1.86400	0.76170
H	-2.79100	-1.34330	-1.64170
H	-2.60350	0.40020	-1.36110
C	-4.18790	1.98250	0.24960
C	-4.65220	0.57460	0.61780
C	-4.39170	-0.45950	-0.48090
H	-4.49450	2.70740	1.00900
H	-4.61230	2.30000	-0.70980
H	-5.72630	0.58570	0.83390
H	-4.14980	0.25350	1.53610
H	-4.71730	-1.44930	-0.14200
H	-4.98120	-0.21440	-1.37320
H	2.44900	2.59430	-1.26420
H	-3.09740	2.03270	0.16870

[4.5]PCP 26 StrainViz fragment r2

Cartesian coordinates

C	0.80900	-0.04190	1.73050
C	1.93650	0.44010	1.06820
C	1.80880	1.59550	0.27890

C	0.57440	2.23290	0.15310
C	-0.54580	1.72030	0.79950
C	-0.43330	0.57810	1.58110
H	0.91430	-0.92040	2.35940
H	0.46800	3.12490	-0.45270
C	-0.92700	-1.93010	-0.60240
C	0.02440	-1.22030	-1.33790
C	-0.37890	-0.13660	-2.12430
C	-1.71050	0.24720	-2.14540
C	-2.67770	-0.43170	-1.39310
C	-2.26050	-1.51980	-0.62650
H	-0.64230	-2.79580	-0.01460
H	0.37520	0.39990	-2.69200
H	-2.01010	1.09710	-2.75500
H	-2.99420	-2.07060	-0.04160
O	-4.16350	0.49920	1.02860
C	-4.36520	1.04920	-0.25450
C	-4.11180	0.03350	-1.37350
C	1.84410	-2.44680	-0.40960
O	1.35240	-1.50990	-1.35610
H	-5.40700	1.38340	-0.29020
H	-3.72830	1.93290	-0.41730
H	-4.78390	-0.81810	-1.21970
H	-4.37370	0.49360	-2.33350
H	1.55380	-3.46460	-0.70400
H	1.40460	-2.24120	0.57320
C	-1.54160	-1.09210	2.87700
O	-1.58870	0.13130	2.16690
H	-1.17220	-1.90180	2.23760
H	-0.90640	-1.00680	3.76530

H	-2.56720	-1.30280	3.18050
O	2.95750	2.04070	-0.31270
C	2.86170	3.17770	-1.14550
H	2.53050	4.05810	-0.58250
H	3.86360	3.35390	-1.53610
H	2.17070	3.00200	-1.97870
C	3.28710	-0.22810	1.20820
C	3.83660	-0.87660	-0.08040
C	3.35460	-2.30470	-0.35240
H	4.00560	0.53190	1.53570
H	3.23530	-0.97850	2.00730
H	4.93000	-0.90160	-0.01490
H	3.58780	-0.23880	-0.93200
H	3.78450	-2.65230	-1.29890
H	3.71920	-2.98110	0.43090
H	-1.51530	2.19920	0.69940
H	-3.21190	0.33240	1.15380

[4.5]PCP 26 StrainViz fragment r3

Cartesian coordinates

C	1.03890	-0.13490	1.76170
C	2.04670	0.41550	0.97770
C	1.74980	1.53350	0.16820
C	0.46140	2.03790	0.12990
C	-0.55570	1.45610	0.89740
C	-0.27030	0.36170	1.72160
H	1.28600	-0.97700	2.39920
H	0.18850	2.88110	-0.49490
C	-0.85780	-2.13050	-0.46110
C	-0.03470	-1.32300	-1.24900

C	-0.60280	-0.29650	-2.01110
C	-1.97290	-0.08310	-1.97950
C	-2.81840	-0.88190	-1.19810
C	-2.23300	-1.89450	-0.43940
H	-0.44650	-2.94850	0.11970
H	0.05310	0.32370	-2.61440
H	-2.40080	0.71830	-2.57790
H	-2.86540	-2.53570	0.17150
C	-1.92470	1.97790	0.78350
O	-2.24370	2.88280	0.03210
C	-4.77590	0.32950	-0.12260
C	-4.31480	-0.66710	-1.19050
C	1.94380	-2.41890	-0.50390
O	1.31540	-1.46360	-1.34410
H	-5.86550	0.43080	-0.12620
H	-4.33850	1.31730	-0.29780
H	-4.81410	-1.62880	-1.02630
H	-4.63430	-0.31210	-2.17690
H	1.66620	-3.43420	-0.81860
H	1.60130	-2.28230	0.52860
C	-1.05920	-1.35240	3.16150
O	-1.29880	-0.16180	2.43480
H	-0.69580	-2.14670	2.49970
H	-0.33610	-1.18720	3.96770
H	-2.01860	-1.64230	3.58840
O	2.79840	2.05440	-0.53110
C	2.52420	3.15100	-1.38090
H	2.15220	4.01030	-0.81130
H	3.46940	3.41060	-1.85640
H	1.78900	2.88140	-2.14800

C	3.44890	-0.14770	0.98550
C	3.90180	-0.77260	-0.35160
C	3.44590	-2.21420	-0.59510
H	4.13800	0.67000	1.22550
H	3.53700	-0.88690	1.79120
H	4.99680	-0.76000	-0.38020
H	3.55890	-0.13830	-1.17200
H	3.79270	-2.53040	-1.58540
H	3.91270	-2.88660	0.13540
H	-2.67670	1.49280	1.42600
H	-4.46740	-0.00800	0.87310

[4.5]PCP 26 StrainViz fragment r4

Cartesian coordinates

C	0.85900	-0.45710	1.75330
C	1.79350	0.38100	1.15760
C	1.33430	1.54830	0.52090
C	-0.02390	1.81390	0.46410
C	-0.96690	0.94360	1.02890
C	-0.52010	-0.21210	1.68880
H	1.22530	-1.34200	2.26180
H	-0.41040	2.68890	-0.04360
C	-0.58640	-1.97400	-1.03160
C	0.24140	-1.02580	-1.63240
C	-0.32870	0.08240	-2.27020
C	-1.70190	0.26010	-2.25760
C	-2.54950	-0.65350	-1.61600
C	-1.96770	-1.76820	-1.01720
H	-0.17120	-2.85840	-0.56090
H	0.33260	0.80550	-2.73840

H	-2.12940	1.14250	-2.72790
H	-2.60080	-2.49570	-0.51500
C	-2.38540	1.32770	0.81660
O	-2.72560	2.30050	0.16940
O	-3.29590	0.53280	1.39740
C	-4.03890	-0.42120	-1.58590
C	2.24250	-2.07440	-0.85830
O	1.60080	-1.08180	-1.64470
H	-4.52130	-1.05220	-0.83440
H	-4.49950	-0.64130	-2.55550
H	2.15440	-3.05390	-1.34770
H	1.75630	-2.14160	0.12070
C	-0.97620	-2.29130	2.73540
O	-1.43380	-1.05700	2.21930
H	-0.43040	-2.85970	1.97280
H	-0.33390	-2.14840	3.61150
H	-1.87000	-2.84070	3.02920
O	2.29590	2.36070	-0.00690
C	1.85930	3.52540	-0.67880
H	1.31120	4.19180	-0.00270
H	2.75920	4.02690	-1.03340
H	1.21930	3.27380	-1.53260
C	3.27130	0.06750	1.19330
C	3.90690	-0.24620	-0.17770
C	3.69930	-1.67200	-0.69880
H	3.79000	0.93810	1.61100
H	3.44510	-0.76840	1.88210
H	4.98620	-0.07740	-0.09530
H	3.53140	0.46880	-0.91370
H	4.20490	-1.76830	-1.66650

H	4.17000	-2.39470	-0.02050
H	-4.16250	0.91370	1.16660
H	-4.25870	0.62620	-1.35100

[4.5]PCP 26 StrainViz fragment r5

Cartesian coordinates

C	0.67930	-0.31770	1.73960
C	1.62580	0.56340	1.23230
C	1.17630	1.66460	0.48120
C	-0.17590	1.82050	0.22790
C	-1.12510	0.90440	0.70360
C	-0.69320	-0.18330	1.47860
H	1.03320	-1.14800	2.34060
H	-0.54930	2.64210	-0.37060
C	-0.18610	-2.25250	-1.01810
C	0.57390	-1.22160	-1.57760
C	-0.05060	-0.23170	-2.34440
C	-1.42810	-0.24800	-2.51030
C	-2.20050	-1.24950	-1.91750
C	-1.57150	-2.24830	-1.18210
H	0.28670	-3.04870	-0.45420
H	0.56460	0.55320	-2.77350
H	-1.90630	0.53820	-3.08660
H	-2.15820	-3.03940	-0.72420
C	-2.52250	1.17270	0.26440
O	-2.81790	2.09600	-0.47010
O	-3.43850	0.32320	0.73850
C	-4.76740	0.55760	0.27520
C	2.57760	-1.96530	-0.51880
O	1.91980	-1.09730	-1.43020

H	-5.37840	-0.22220	0.72650
H	-5.11460	1.54510	0.58620
H	2.65200	-2.97280	-0.95010
H	1.99530	-2.03720	0.40630
C	-1.13640	-2.23800	2.57850
O	-1.60770	-1.07020	1.93660
H	-0.45530	-2.79850	1.92710
H	-0.62850	-2.00350	3.52080
H	-2.02010	-2.84070	2.78610
O	2.14040	2.52980	0.05000
C	1.72130	3.61760	-0.74950
H	1.03330	4.27170	-0.20160
H	2.62370	4.17200	-1.00560
H	1.23230	3.26910	-1.66680
C	3.10400	0.37270	1.48260
C	3.94470	0.07100	0.22340
C	3.95450	-1.39090	-0.23380
H	3.49030	1.29540	1.93060
H	3.24480	-0.42420	2.22330
H	4.98080	0.36140	0.42890
H	3.59670	0.70580	-0.59490
H	4.57160	-1.47370	-1.13580
H	4.42250	-2.02300	0.53120
H	-4.80640	0.49370	-0.81480
H	-3.27950	-1.25390	-2.03690

[4.5]PCP 26 StrainViz fragment r6

Cartesian coordinates

C	-0.74690	-1.01980	-1.01790
C	-1.63050	0.04690	-0.88910

C	-1.11060	1.31100	-0.54800
C	0.24800	1.45240	-0.30480
C	1.13060	0.36830	-0.40710
C	0.62950	-0.89230	-0.78030
H	-1.15660	-1.97960	-1.31020
H	0.67760	2.40810	-0.03160
C	2.55670	0.69610	-0.10260
O	2.92770	1.82920	0.13420
O	3.38170	-0.35170	-0.10750
C	4.76040	-0.07400	0.19690
C	4.97380	0.05210	1.69370
C	-2.78040	-1.73910	1.58080
O	-1.97720	-0.75350	2.20830
H	5.30300	-0.92690	-0.21320
H	5.06320	0.83430	-0.32810
H	4.63340	-0.85220	2.20470
H	6.03770	0.19040	1.90540
H	-2.98560	-2.49620	2.34390
H	-2.22320	-2.23860	0.77720
C	0.95340	-3.19830	-1.24630
O	1.48490	-1.93210	-0.90040
H	0.22750	-3.54150	-0.50050
H	0.48090	-3.17640	-2.23440
H	1.80320	-3.87940	-1.26590
O	-2.01060	2.33140	-0.48860
C	-1.51930	3.61260	-0.13920
H	-0.77940	3.96360	-0.86700
H	-2.38170	4.27790	-0.14560
H	-1.07010	3.60400	0.86020
C	-3.11510	-0.11700	-1.11080

C	-3.96840	0.06000	0.16380
C	-4.10040	-1.18260	1.04900
H	-3.43000	0.63510	-1.84240
H	-3.30880	-1.10090	-1.55430
H	-4.97500	0.36720	-0.13870
H	-3.56220	0.88740	0.75440
H	-4.74040	-0.92580	1.90130
H	-4.61160	-1.98130	0.49540
H	-1.56760	-0.22290	1.50710
H	4.42640	0.91130	2.08690

[4.5]PCP 26 StrainViz fragment r7

Cartesian coordinates

C	1.86120	-1.53570	-0.48370
C	2.77180	-0.74620	0.20740
C	2.31670	0.01910	1.29500
C	0.97550	-0.01020	1.64070
C	0.05430	-0.79960	0.93650
C	0.50070	-1.57840	-0.14610
H	2.23110	-2.11670	-1.32070
H	0.58580	0.58400	2.45800
C	-1.78190	1.64480	-1.57400
C	-2.37400	2.83680	-1.16200
C	-3.57350	2.80170	-0.45300
C	-4.17220	1.58040	-0.15380
C	-3.58670	0.37960	-0.56160
C	-2.38740	0.42500	-1.27990
H	-0.84620	1.66310	-2.12550
H	-4.04250	3.72650	-0.13060
H	-5.10390	1.55650	0.40690

H	-1.91770	-0.50670	-1.58820
C	-1.35660	-0.68410	1.40900
O	-1.72180	0.19040	2.16680
O	-2.16430	-1.64700	0.94550
C	-3.57390	-1.47570	1.13410
C	-4.18500	-0.94810	-0.16580
C	2.34730	1.68360	-1.95080
H	-3.96710	-2.46490	1.37600
H	-3.75360	-0.79370	1.96630
H	-4.01400	-1.69000	-0.95290
H	-5.26710	-0.85840	-0.02110
H	1.80070	1.86840	-2.88170
H	1.68330	1.13960	-1.27280
C	0.06370	-3.02560	-1.98020
O	-0.39620	-2.32140	-0.84100
H	0.49090	-2.34320	-2.72370
H	0.80810	-3.78170	-1.70810
H	-0.81460	-3.51610	-2.39830
O	3.26120	0.76490	1.93640
C	2.82750	1.56310	3.02070
H	2.39720	0.94720	3.81860
H	3.71400	2.07480	3.39410
H	2.08680	2.30260	2.69600
C	4.20480	-0.64270	-0.24040
C	4.51160	0.67340	-0.98000
C	3.64430	0.91840	-2.22550
H	4.86610	-0.72130	0.62900
H	4.43070	-1.48430	-0.90490
H	5.56850	0.64430	-1.26780
H	4.39850	1.51060	-0.28130

H	4.22890	1.48630	-2.95820
H	3.41240	-0.04370	-2.70310
H	2.56620	2.65510	-1.49430
H	-1.90410	3.78810	-1.39190

[4.5]PCP 26 StrainViz fragment r8

Cartesian coordinates

C	1.25130	-1.59580	0.90660
C	2.21190	-0.59190	0.93900
C	1.79620	0.72900	1.17780
C	0.45710	0.99870	1.40460
C	-0.51340	-0.01320	1.36110
C	-0.11340	-1.33290	1.09560
H	1.58800	-2.60920	0.71870
H	0.10400	2.00490	1.59180
C	-0.40190	-0.45540	-2.14870
C	0.07630	0.85280	-2.07360
C	-0.79180	1.88840	-1.72260
C	-2.11820	1.60350	-1.41040
C	-2.60400	0.29400	-1.43640
C	-1.72500	-0.72230	-1.82020
H	0.27480	-1.25030	-2.44520
H	-0.42500	2.91140	-1.67860
H	-2.77550	2.41520	-1.10730
H	-2.07860	-1.75040	-1.83910
C	-1.91440	0.47790	1.48340
O	-2.17590	1.62350	1.79130
O	-2.86250	-0.42440	1.17180
C	-4.06480	0.10830	0.60120
C	-3.98520	-0.04940	-0.92750

O	1.39200	1.06120	-2.36110
H	-4.89590	-0.46370	1.01730
H	-4.16850	1.15400	0.89290
H	-4.21880	-1.08420	-1.19600
H	-4.75450	0.58830	-1.37800
C	-0.65400	-3.58590	0.59160
O	-1.05480	-2.30440	1.03630
H	-0.17520	-3.52860	-0.39330
H	0.03050	-4.06360	1.30130
H	-1.56760	-4.17560	0.52020
O	2.77890	1.67800	1.16950
C	2.37770	3.01960	1.35780
H	1.91470	3.16380	2.34080
H	3.28380	3.62120	1.29140
H	1.67030	3.33330	0.58010
C	3.66810	-0.88400	0.68620
C	4.08680	-0.54980	-0.75630
C	3.45580	-1.48900	-1.77840
H	4.27530	-0.29870	1.38370
H	3.86520	-1.94430	0.88440
H	5.17940	-0.60380	-0.82510
H	3.79850	0.48370	-0.97590
H	3.73570	-1.21190	-2.79890
H	3.77310	-2.52410	-1.60610
H	2.36510	-1.44970	-1.71000
H	1.60860	1.99450	-2.21640

[4.5]PCP 26 StrainViz fragment r9

Cartesian coordinates

C	2.42190	1.15810	1.05700

C	3.26610	0.32260	0.33580
C	2.81990	-0.19240	-0.89360
C	1.56190	0.14790	-1.36490
C	0.71740	1.00430	-0.64440
C	1.14720	1.51380	0.59350
H	2.77930	1.53770	2.00740
H	1.18330	-0.23730	-2.30360
C	-1.95170	-1.27690	1.19050
C	-2.68200	-2.23240	0.48320
C	-3.71480	-1.82380	-0.36920
C	-4.00170	-0.47620	-0.51600
C	-3.27930	0.49860	0.18240
C	-2.26080	0.07550	1.03450
H	-1.14590	-1.56520	1.85540
H	-4.27290	-2.58300	-0.90780
H	-4.79870	-0.17170	-1.19050
H	-1.67400	0.81770	1.57160
C	-0.60830	1.25810	-1.28230
O	-1.03660	0.57740	-2.19050
O	-1.25000	2.32480	-0.78790
C	-2.61870	2.50980	-1.16900
C	-3.51940	1.96810	-0.05670
C	-1.41660	-4.01920	1.39050
O	-2.46910	-3.57440	0.55700
H	-2.75130	3.58650	-1.29120
H	-2.80740	2.00470	-2.11750
H	-3.32010	2.53900	0.85650
H	-4.56100	2.15180	-0.34270
H	-1.59140	-3.73800	2.43530
H	-0.45290	-3.61210	1.06350

C	0.74490	2.76950	2.57170
O	0.31030	2.31030	1.30490
H	0.95010	1.93370	3.24970
H	1.63940	3.39560	2.48300
H	-0.07670	3.36560	2.96740
O	3.69120	-1.01230	-1.54870
C	3.27130	-1.54020	-2.79220
H	3.06910	-0.74040	-3.51380
H	4.09380	-2.15680	-3.15300
H	2.37340	-2.15800	-2.67730
C	4.61780	-0.07110	0.87300
C	4.60490	-1.48820	1.45930
H	5.35560	-0.02070	0.06590
H	4.92170	0.64630	1.64200
H	5.59380	-1.75960	1.83940
H	4.31830	-2.21390	0.69370
H	3.88910	-1.55720	2.28400
H	-1.40010	-5.10530	1.30520

[4.5]PCP 26 StrainViz fragment r10

Cartesian coordinates

C	0.47340	2.56670	0.43350
C	1.67060	2.04960	-0.05270
C	1.62790	0.99200	-0.97430
C	0.40410	0.49370	-1.39670
C	-0.79810	1.00110	-0.88960
C	-0.76860	2.05560	0.03620
H	0.52380	3.38890	1.13790
H	0.33780	-0.33110	-2.09510
C	0.11120	-1.15510	1.68360

C	0.76170	-1.95190	0.74180
C	0.00790	-2.74400	-0.13350
C	-1.37740	-2.70260	-0.09300
C	-2.05080	-1.87880	0.81790
C	-1.28320	-1.12240	1.70040
H	0.66750	-0.53440	2.37580
H	0.53210	-3.36850	-0.85060
H	-1.94810	-3.29880	-0.80160
H	-1.78280	-0.46190	2.40530
C	-2.01360	0.25360	-1.32160
O	-2.03680	-0.42670	-2.32550
O	-3.04440	0.33140	-0.46050
C	-3.93230	-0.79450	-0.43930
C	-3.54760	-1.68680	0.75210
C	2.90870	-1.19260	1.43330
O	2.11210	-2.00980	0.58480
H	-4.94360	-0.39870	-0.33040
H	-3.85500	-1.33230	-1.38490
H	-3.89500	-1.21800	1.67800
H	-4.07410	-2.64230	0.64810
H	2.77050	-1.50630	2.47670
H	2.58200	-0.14890	1.34410
C	-1.90930	3.61820	1.41090
O	-1.94860	2.54980	0.48470
H	-1.38820	3.32870	2.33050
H	-1.42390	4.50160	0.98100
H	-2.94830	3.85140	1.64110
O	2.83830	0.51750	-1.38800
C	2.82530	-0.57000	-2.29570
H	2.31710	-0.29850	-3.22810

H	3.87000	-0.79940	-2.50690
H	2.34110	-1.44360	-1.84610
C	3.00100	2.60310	0.37700
C	4.35010	-1.34610	0.99950
H	3.55130	2.99650	-0.48380
H	2.87500	3.40420	1.10830
H	4.65690	-2.39420	1.04720
H	5.00040	-0.76240	1.65670
H	4.47340	-0.98210	-0.02340
H	3.62930	1.82070	0.81690

[4.5]PCP 26 StrainViz fragment r11

Cartesian coordinates

C	-0.39020	2.72550	0.66920
C	0.88350	2.55970	0.14090
C	1.11750	1.63950	-0.88130
C	0.05290	0.88450	-1.36060
C	-1.22680	1.01850	-0.81430
C	-1.46450	1.95690	0.20440
H	-0.53720	3.45950	1.45170
H	0.18480	0.13660	-2.13210
C	0.34840	-0.94390	1.55870
C	1.19090	-1.56410	0.63530
C	0.65990	-2.49660	-0.26500
C	-0.69400	-2.79880	-0.24050
C	-1.55610	-2.18910	0.67940
C	-1.00910	-1.26230	1.56480
H	0.72270	-0.19520	2.24670
H	1.33000	-2.97040	-0.97590
H	-1.09220	-3.51390	-0.95690

H	-1.66350	-0.75150	2.26760
C	-2.20490	0.00310	-1.30620
O	-2.06490	-0.57730	-2.36130
O	-3.18990	-0.27580	-0.43560
C	-3.71040	-1.61150	-0.48490
C	-3.04940	-2.41830	0.64270
C	3.11040	-0.42580	1.46650
O	2.52690	-1.32670	0.53230
H	-4.78990	-1.53710	-0.34220
H	-3.50440	-2.04410	-1.46460
H	-3.48580	-2.11530	1.59950
H	-3.28850	-3.47710	0.49230
H	2.82590	-0.71890	2.48520
H	2.73180	0.58880	1.27650
C	-2.95910	3.04320	1.69070
O	-2.72930	2.08660	0.67400
H	-2.37890	2.81330	2.59150
H	-2.71380	4.05490	1.34860
H	-4.02250	2.98540	1.91980
O	2.39780	1.54010	-1.33590
C	2.63880	0.57900	-2.34770
H	2.03770	0.78750	-3.24020
H	3.69650	0.65850	-2.59660
H	2.42280	-0.43170	-1.98130
C	5.08820	-0.08170	-0.08710
C	4.61820	-0.48390	1.30690
H	6.18010	-0.07300	-0.14580
H	4.71200	-0.78410	-0.83570
H	4.95640	-1.49900	1.54390
H	5.05340	0.18150	2.06110

H	4.71760	0.91580	-0.34550
H	1.71490	3.15030	0.51380

Fully Optimized StrainViz Fragments of [6.5]PCP 2

Parent

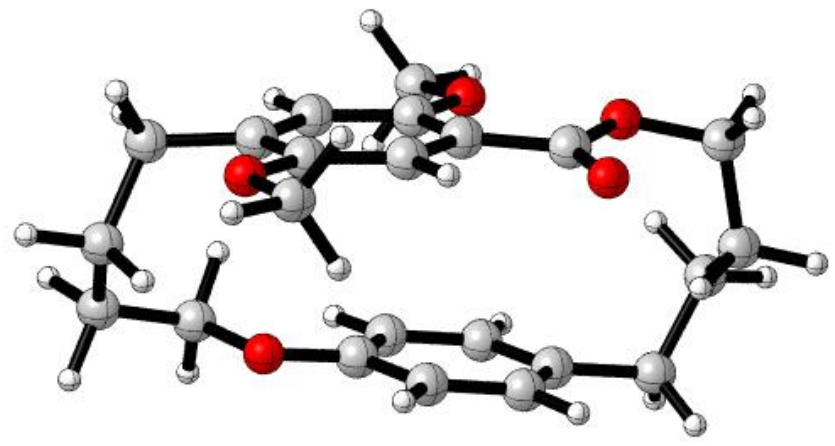

Fragment 1 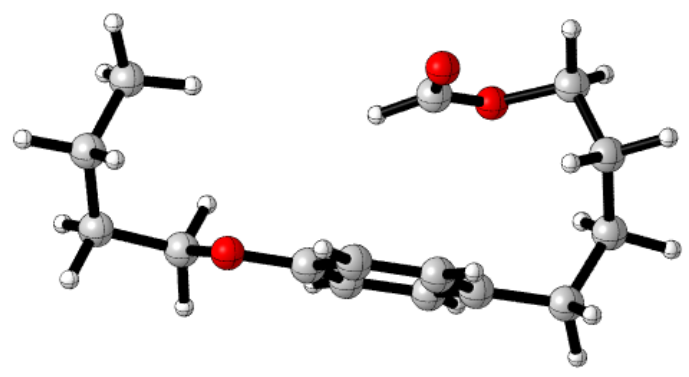	Fragment 2 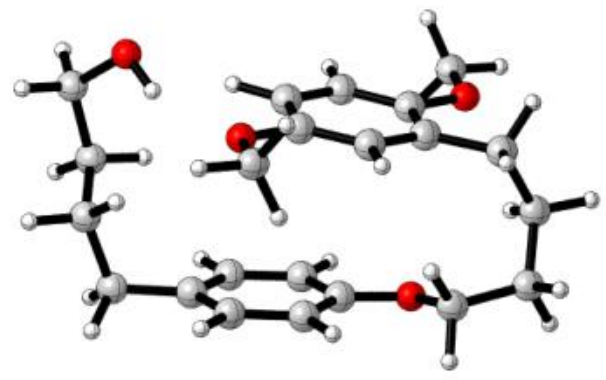	Fragment 3 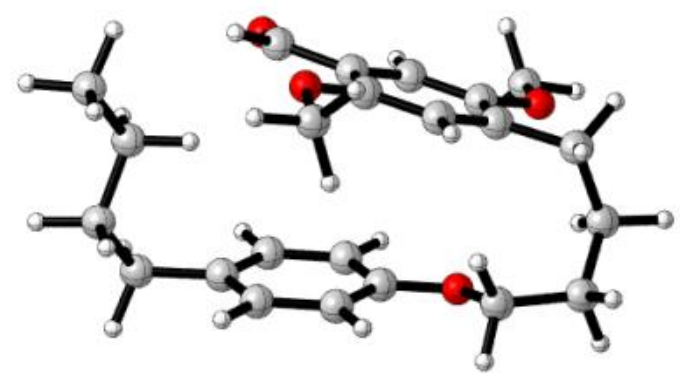
Fragment 4 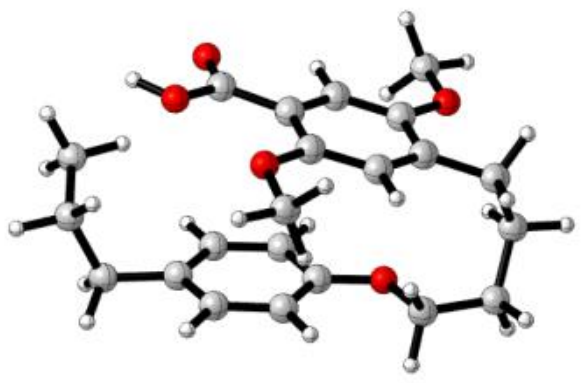	Fragment 5 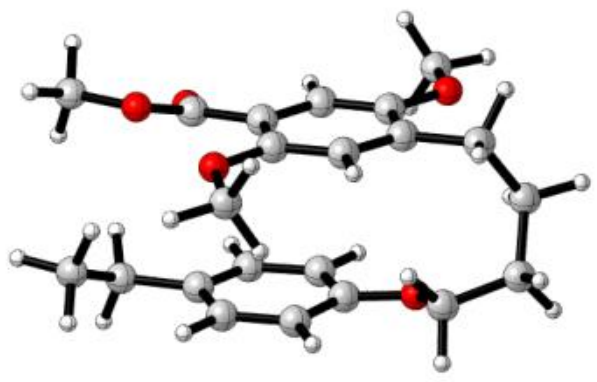	Fragment 6 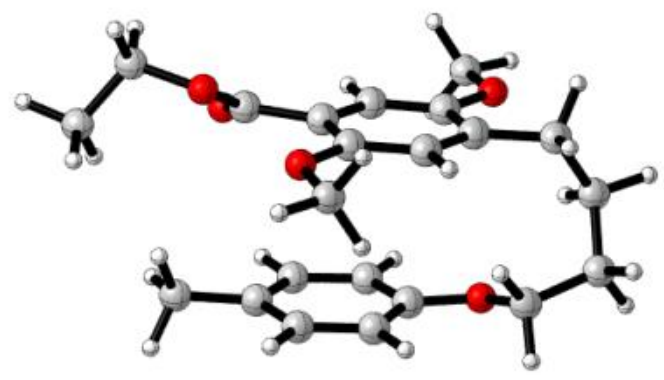
Fragment 7 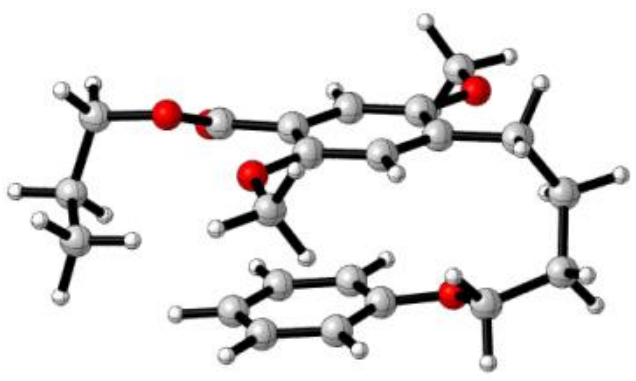	Fragment 8 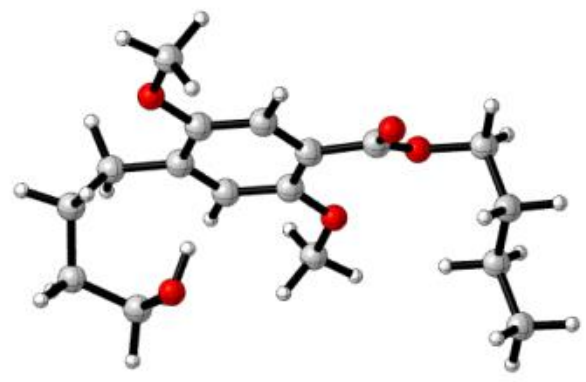	Fragment 9 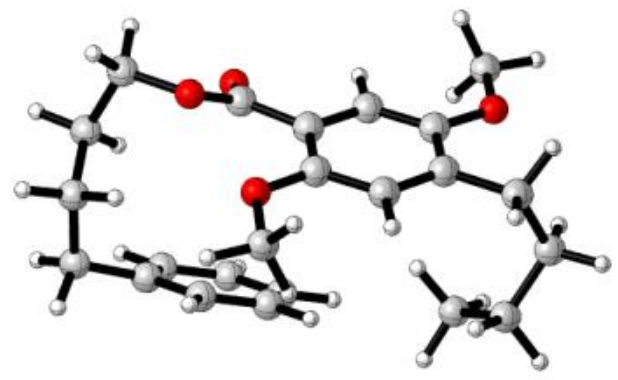
Fragment 10 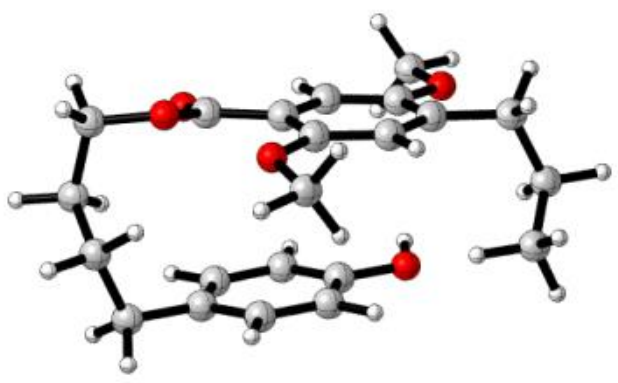	Fragment 11 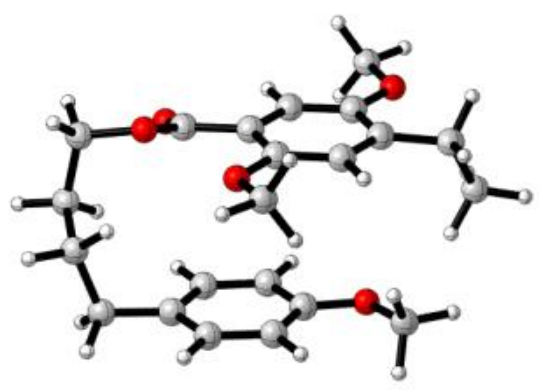	Fragment 12 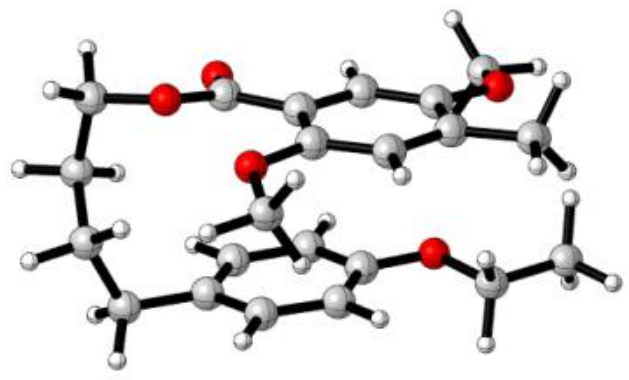
Fragment 13 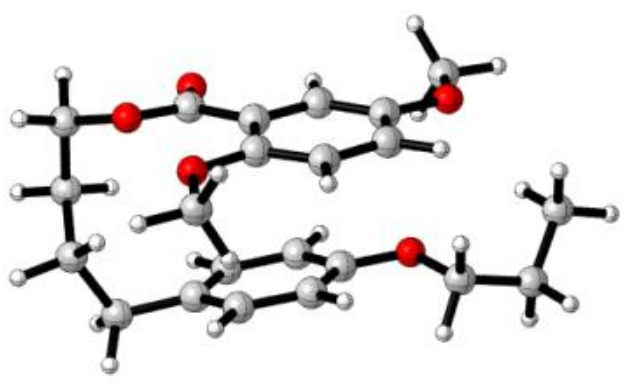		

[6.5]PCP 2 StrainViz fragment r1

Cartesian coordinates

C	-0.46980	-1.36830	-0.94960
C	-1.10030	-1.09590	0.26890
C	-0.33760	-1.02950	1.43820
C	1.03470	-1.23680	1.38760
C	1.68770	-1.51350	0.18110

C	0.90950	-1.56960	-0.97610
H	-1.03660	-1.43120	-1.87100
H	-0.84060	-0.81000	2.37440
H	1.61250	-1.18370	2.30740
H	1.38810	-1.78200	-1.92980
C	0.99120	1.74610	-0.04500
O	1.11870	2.16410	1.07820
O	1.96950	1.62090	-0.94220
C	-3.23310	-0.84020	-0.76300
O	-2.43620	-0.88450	0.41220
H	-3.20380	-1.81200	-1.27310
H	-2.82920	-0.08530	-1.45180
C	-4.65210	-0.49310	-0.34780
C	-4.75200	0.80140	0.46410
C	-4.13150	2.00850	-0.23690
H	-5.25080	-0.40880	-1.26320
H	-5.06590	-1.32580	0.23170
H	-5.80970	0.99880	0.67070
H	-4.26440	0.65370	1.43330
H	-4.32850	2.92890	0.31970
H	-4.53800	2.13180	-1.24720
C	3.85810	0.74380	0.30840
C	3.93050	-0.54870	-0.52800
C	3.18220	-1.72890	0.11240
C	3.29820	1.92180	-0.47250
H	3.87490	2.11050	-1.38030
H	3.26420	2.82980	0.13350
H	3.22670	0.59610	1.19210
H	4.85030	1.02500	0.67680
H	4.97510	-0.83640	-0.68540

H	3.50370	-0.36940	-1.52260
H	3.57400	-1.90030	1.12210
H	3.39170	-2.63600	-0.46670
H	-3.04490	1.90220	-0.32140
H	0.04020	1.41700	-0.48540

[6.5]PCP 2 StrainViz fragment r2

Cartesian coordinates

C	0.99780	0.42460	1.51960
C	2.13180	0.88110	0.84790
C	1.96740	1.79800	-0.20350
C	0.69390	2.23460	-0.56670
C	-0.42690	1.76120	0.10750
C	-0.28080	0.84310	1.14090
H	1.13030	-0.26610	2.34630
H	0.55950	2.95620	-1.36400
C	-0.11000	-2.52920	0.08480
C	0.67110	-1.84100	-0.84690
C	0.04900	-1.13940	-1.88220
C	-1.33690	-1.11340	-1.97060
C	-2.13940	-1.77640	-1.03490
C	-1.49820	-2.48040	-0.01650
H	0.34750	-3.10380	0.88230
H	0.67080	-0.60870	-2.59630
H	-1.80710	-0.56550	-2.78430
H	-2.09750	-3.01340	0.71960
O	-3.54490	2.24760	1.03980
C	2.67480	-2.32640	0.33170
O	2.03100	-1.80730	-0.82170
H	2.60100	-3.42250	0.34210

H	2.17350	-1.94770	1.23090
C	-1.31930	-0.45970	2.84740
O	-1.43870	0.41070	1.73830
H	-0.81380	-1.38840	2.56220
H	-0.77320	0.02090	3.66710
H	-2.33680	-0.68400	3.16850
O	3.11930	2.23960	-0.79340
C	2.99370	3.16020	-1.85730
H	2.51770	4.09050	-1.52590
H	4.00720	3.37290	-2.19670
H	2.41380	2.73450	-2.68470
C	4.12320	-1.87770	0.31100
C	4.29430	-0.36130	0.19000
C	3.53020	0.46400	1.24970
H	4.58920	-2.23680	1.23710
H	4.64330	-2.36860	-0.51950
H	5.36310	-0.13590	0.27050
H	3.98130	-0.03840	-0.80560
H	4.10410	1.37570	1.45030
H	3.48460	-0.09170	2.19510
C	-4.08610	0.78500	-0.86240
C	-4.21980	-0.58840	-0.20560
C	-3.64770	-1.72130	-1.07380
C	-4.50930	1.93040	0.05100
H	-5.47520	1.68210	0.52010
H	-4.65930	2.84380	-0.53380
H	-3.04970	0.95240	-1.18360
H	-4.70560	0.80150	-1.76830
H	-5.28170	-0.77880	-0.00630
H	-3.70440	-0.60160	0.76480

H	-3.99630	-1.59350	-2.10650
H	-4.04710	-2.67880	-0.72130
H	-1.42010	2.12100	-0.13800
H	-3.07090	1.44160	1.30880

[6.5]PCP 2 StrainViz fragment r3

Cartesian coordinates

C	1.15380	0.29570	1.69620
C	2.31770	0.56460	0.98450
C	2.27110	1.50260	-0.07020
C	1.07820	2.12740	-0.39260
C	-0.09000	1.85020	0.32910
C	-0.05690	0.92360	1.37720
H	1.20260	-0.40900	2.51970
H	1.00340	2.85190	-1.19580
C	-0.52220	-2.45440	-0.24790
C	0.41480	-1.71000	-0.97010
C	-0.02680	-0.77520	-1.90930
C	-1.38690	-0.59510	-2.12390
C	-2.34340	-1.34610	-1.43090
C	-1.88270	-2.26780	-0.48980
H	-0.20700	-3.18880	0.48460
H	0.71290	-0.19040	-2.44750
H	-1.71530	0.14510	-2.85000
H	-2.59780	-2.86810	0.06860
C	-1.33460	2.54960	-0.03330
O	-1.44390	3.26150	-1.01510
C	2.23830	-2.60470	0.26140
O	1.76250	-1.84260	-0.83570
H	1.96040	-3.66010	0.13900

H	1.77070	-2.24020	1.18590
C	-1.27340	-0.40370	2.92910
O	-1.22140	0.69520	2.03660
H	-0.95530	-1.32430	2.42690
H	-0.64980	-0.22780	3.81230
H	-2.31640	-0.49180	3.23330
O	3.45790	1.75080	-0.69420
C	3.44200	2.66970	-1.76900
H	3.12390	3.66410	-1.43560
H	4.46480	2.71990	-2.14070
H	2.77670	2.32800	-2.57050
C	3.74790	-2.45560	0.32090
C	4.23540	-1.00590	0.27120
C	3.63700	-0.07680	1.34840
H	4.08980	-2.93920	1.24430
H	4.19600	-3.00810	-0.51270
H	5.32430	-1.01410	0.38890
H	4.02910	-0.58660	-0.71570
H	4.35340	0.73150	1.53480
H	3.52410	-0.62390	2.29250
C	-4.11790	0.48400	0.21770
C	-4.62800	-0.79360	-0.44040
C	-3.82380	-1.17140	-1.69170
C	-4.92830	0.89000	1.44500
H	-5.97060	1.10060	1.18190
H	-4.51320	1.78520	1.91940
H	-3.07190	0.33460	0.50910
H	-4.12290	1.29780	-0.52170
H	-5.68410	-0.67870	-0.71620
H	-4.58710	-1.61470	0.28840

H	-3.95970	-0.39250	-2.45170
H	-4.23340	-2.09650	-2.11660
H	-2.18630	2.40580	0.65170
H	-4.93050	0.08710	2.19090

[6.5]PCP 2 StrainViz fragment r4

Cartesian coordinates

C	1.43440	0.12230	1.72040
C	2.38370	0.60590	0.82990
C	1.99040	1.58130	-0.10630
C	0.67560	2.00930	-0.14330
C	-0.28700	1.49940	0.74130
C	0.09330	0.53530	1.68780
H	1.75370	-0.60900	2.45460
H	0.33960	2.75070	-0.85730
C	-0.40140	-2.36010	-0.10780
C	0.31230	-1.59540	-1.03300
C	-0.38340	-0.78050	-1.93230
C	-1.77170	-0.75760	-1.92330
C	-2.50960	-1.53200	-1.01670
C	-1.79530	-2.31140	-0.10710
H	0.10900	-3.01260	0.59130
H	0.18640	-0.18700	-2.64070
H	-2.29560	-0.14660	-2.65480
H	-2.33920	-2.92560	0.60750
C	-1.65260	2.04740	0.55540
O	-1.93130	2.85570	-0.31030
O	-2.58190	1.60670	1.41600
C	2.40280	-2.40220	-0.23680
O	1.66840	-1.59790	-1.14590

H	2.17390	-3.46290	-0.40860
H	2.10860	-2.15880	0.79120
C	-0.46840	-1.01700	3.39210
O	-0.84170	0.04060	2.53040
H	-0.08680	-1.87230	2.82260
H	0.28540	-0.69530	4.11930
H	-1.37770	-1.30600	3.91810
O	2.97340	2.05800	-0.92340
C	2.59660	3.00440	-1.90400
H	2.20100	3.91710	-1.44390
H	3.50250	3.24120	-2.46110
H	1.84480	2.58900	-2.58520
C	3.88160	-2.12750	-0.44390
C	4.25610	-0.64330	-0.41220
C	3.81560	0.12440	0.85230
H	4.42680	-2.67160	0.33730
H	4.19580	-2.55040	-1.40480
H	5.34600	-0.57130	-0.49470
H	3.84100	-0.14480	-1.29100
H	4.46290	1.00280	0.95650
H	3.97540	-0.49740	1.74170
C	-4.65980	0.88950	-0.91050
C	-4.70480	-0.46960	-0.21810
C	-4.02110	-1.57790	-1.03470
H	-3.63360	1.21390	-1.10600
H	-5.18290	0.84700	-1.87240
H	-5.75040	-0.75230	-0.04940
H	-4.22610	-0.40980	0.76670
H	-4.37860	-1.52410	-2.07140
H	-4.33960	-2.55140	-0.64530

H	-3.41110	2.04940	1.15790
H	-5.15110	1.66350	-0.30920

[6.5]PCP 2 StrainViz fragment r5

Cartesian coordinates

C	0.99940	0.02070	1.73110
C	2.01270	0.70990	1.07570
C	1.65130	1.70750	0.15120
C	0.31340	1.96420	-0.10250
C	-0.70370	1.25460	0.54990
C	-0.35970	0.25800	1.47470
H	1.28520	-0.73570	2.45390
H	0.00650	2.71200	-0.82360
C	0.01780	-2.39770	-0.52230
C	0.83100	-1.54880	-1.27330
C	0.24480	-0.63260	-2.15190
C	-1.13460	-0.52530	-2.21890
C	-1.97180	-1.32040	-1.42440
C	-1.36980	-2.26560	-0.59620
H	0.45090	-3.14560	0.13300
H	0.89270	0.01700	-2.73260
H	-1.57810	0.22620	-2.86790
H	-1.98240	-2.91000	0.02810
C	-2.09010	1.62650	0.15450
O	-2.35050	2.24930	-0.85690
O	-3.03280	1.27010	1.03420
C	2.80990	-2.21560	-0.12760
O	2.19020	-1.53090	-1.20610
H	2.82610	-3.29570	-0.32720
H	2.23280	-2.05210	0.78970

C	-0.98640	-1.55220	2.87390
O	-1.35070	-0.45170	2.06510
H	-0.36870	-2.26470	2.31520
H	-0.44870	-1.22890	3.77250
H	-1.92180	-2.03000	3.16480
O	2.68350	2.38360	-0.43310
C	2.35180	3.35840	-1.40160
H	1.75240	4.16540	-0.96440
H	3.29730	3.76250	-1.76220
H	1.80040	2.91320	-2.23810
C	4.21730	-1.66760	0.02800
C	4.27940	-0.14480	0.17770
C	3.47310	0.43860	1.35890
H	4.66610	-2.15210	0.90430
H	4.81600	-1.96350	-0.84110
H	5.33040	0.13380	0.31130
H	3.94530	0.32630	-0.74980
H	3.93680	1.38950	1.64550
H	3.55340	-0.22710	2.22740
C	-4.21900	-1.58040	-0.23210
C	-3.47210	-1.12430	-1.48470
C	-4.35680	1.70020	0.71750
H	-4.98590	1.32980	1.52520
H	-4.39920	2.79020	0.66520
H	-5.26990	-1.27890	-0.27840
H	-4.19600	-2.66930	-0.12510
H	-3.65920	-0.05680	-1.65820
H	-3.87980	-1.64370	-2.36230
H	-4.68190	1.28330	-0.23810
H	-3.76520	-1.13850	0.66150

[6.5]PCP 2 StrainViz fragment r6

Cartesian coordinates

C	0.94510	0.14270	1.68230
C	1.86060	0.95640	1.02360
C	1.38160	1.83430	0.03440
C	0.02770	1.87090	-0.26430
C	-0.88850	1.05440	0.40960
C	-0.42510	0.15950	1.38290
H	1.31960	-0.52590	2.44980
H	-0.36680	2.53380	-1.02480
C	0.56820	-2.60540	-0.38150
C	1.22260	-1.63830	-1.15010
C	0.49690	-0.87940	-2.06880
C	-0.87820	-1.04390	-2.17190
C	-1.56070	-1.96620	-1.37470
C	-0.81140	-2.75130	-0.49720
H	1.12070	-3.23330	0.30930
H	1.02370	-0.13640	-2.65980
H	-1.43900	-0.41570	-2.85930
H	-1.31690	-3.48850	0.12340
C	-2.31510	1.24570	0.01930
O	-2.65160	1.68410	-1.06280
O	-3.18600	0.99220	1.00410
C	3.25230	-1.85370	0.07550
O	2.55400	-1.36790	-1.06120
H	3.47830	-2.92150	-0.04810
H	2.62210	-1.74430	0.96620
C	-0.82740	-1.63750	2.87590
O	-1.32220	-0.66620	1.97560

H	-0.08880	-2.28510	2.38940
H	-0.37880	-1.17090	3.76050
H	-1.68980	-2.23090	3.17880
O	2.31510	2.63540	-0.55810
C	1.86880	3.50140	-1.58230
H	1.14010	4.22610	-1.20100
H	2.75230	4.02900	-1.94040
H	1.41840	2.93850	-2.40830
C	4.52470	-1.03950	0.22410
C	4.28720	0.47230	0.26880
C	3.33050	0.95510	1.38090
H	5.02550	-1.37130	1.14230
H	5.19910	-1.27030	-0.60850
H	5.25740	0.95930	0.41450
H	3.90790	0.81020	-0.69820
H	3.60570	1.98430	1.63890
H	3.47920	0.35310	2.28620
C	-5.33350	0.38170	-0.01860
C	-3.06080	-2.09420	-1.42530
C	-4.54500	1.40490	0.77470
H	-4.95820	1.53050	1.77690
H	-4.53490	2.37180	0.26640
H	-4.91100	0.26270	-1.01830
H	-6.37060	0.71620	-0.11520
H	-3.48620	-1.45430	-2.20330
H	-3.37320	-3.12500	-1.62310
H	-3.50110	-1.79600	-0.46770
H	-5.33060	-0.58650	0.48910

[6.5]PCP 2 StrainViz fragment r7

Cartesian coordinates

C	0.98900	-0.14000	1.70650
C	2.00460	0.64560	1.17410
C	1.65240	1.70280	0.31520
C	0.32020	1.92560	0.00280
C	-0.69710	1.12590	0.53760
C	-0.36220	0.06510	1.39110
H	1.26810	-0.94420	2.37880
H	0.02030	2.71820	-0.67200
C	0.21150	-2.41010	-0.81540
C	1.00450	-1.43240	-1.42250
C	0.42000	-0.48570	-2.27070
C	-0.95100	-0.49560	-2.48090
C	-1.75610	-1.44830	-1.85260
C	-1.16750	-2.40050	-1.02710
H	0.65490	-3.17100	-0.18300
H	1.06020	0.26210	-2.72810
H	-1.39930	0.25900	-3.11970
H	-1.78260	-3.15190	-0.53910
C	-2.07820	1.46300	0.09510
O	-2.31740	2.07270	-0.92880
O	-3.02520	1.08340	0.95870
C	2.95490	-2.10900	-0.23220
O	2.34810	-1.32290	-1.24690
H	3.01550	-3.15710	-0.55540
H	2.34220	-2.07190	0.67590
C	-0.98710	-1.89010	2.58310
O	-1.35280	-0.73620	1.85360
H	-0.30860	-2.52590	2.00260
H	-0.51600	-1.62910	3.53750

H	-1.91620	-2.42730	2.77390
O	2.68400	2.46790	-0.14740
C	2.36260	3.51350	-1.04280
H	1.70240	4.25110	-0.57190
H	3.30710	3.98780	-1.30770
H	1.88100	3.12530	-1.94790
C	4.33690	-1.54070	0.03730
C	4.34720	-0.03920	0.33840
C	3.45750	0.40710	1.51840
H	4.76320	-2.09830	0.88060
H	4.98010	-1.73260	-0.82910
H	5.38050	0.24880	0.56050
H	4.05210	0.51390	-0.55610
H	3.86480	1.34590	1.91100
H	3.51900	-0.32890	2.32980
C	-4.81400	0.16610	-0.40530
C	-4.61340	-1.21880	0.20600
C	-4.38650	1.27470	0.54420
H	-4.96020	1.23990	1.47350
H	-4.49070	2.26130	0.08730
H	-4.23770	0.26460	-1.33190
H	-5.86690	0.32740	-0.66350
H	-5.22820	-1.33940	1.10490
H	-3.56530	-1.36020	0.48940
H	-2.82890	-1.45510	-2.01610
H	-4.89130	-2.00670	-0.50090

[6.5]PCP 2 StrainViz fragment r8

Cartesian coordinates

C	-1.12770	-0.74410	-1.21110

C	-2.18650	0.08760	-0.85630
C	-1.89590	1.33450	-0.26900
C	-0.57580	1.68940	-0.02290
C	0.48260	0.84740	-0.38260
C	0.20950	-0.39300	-0.98270
H	-1.36550	-1.69440	-1.67550
H	-0.32050	2.63080	0.44820
C	1.84590	1.36150	-0.05650
O	2.04350	2.19330	0.80590
O	2.80940	0.86010	-0.83060
C	-2.88320	-2.34800	1.20320
O	-2.24000	-1.38270	2.01830
H	-2.91140	-3.27200	1.78880
H	-2.28590	-2.55660	0.30560
C	0.95150	-2.50160	-1.78540
O	1.24570	-1.21110	-1.28230
H	0.33490	-3.07090	-1.08060
H	0.44330	-2.44540	-2.75410
H	1.91400	-2.99640	-1.90930
O	-2.96690	2.12680	0.01270
C	-2.70860	3.37690	0.62540
H	-2.08580	4.00990	-0.01650
H	-3.68000	3.84800	0.76990
H	-2.21600	3.24690	1.59540
C	-4.30520	-1.94720	0.81490
C	-4.43580	-0.54970	0.20300
C	-3.62470	-0.31120	-1.08920
H	-4.69490	-2.69820	0.11500
H	-4.93680	-1.98750	1.71030
H	-5.49380	-0.37590	-0.01970

H	-4.15390	0.20230	0.94690
H	-4.10770	0.49360	-1.65440
H	-3.65360	-1.20940	-1.71740
C	4.69220	0.43700	0.65580
C	4.64290	-1.06860	0.39920
C	5.18080	-1.87050	1.58120
C	4.15650	1.25130	-0.51100
H	4.71930	1.04700	-1.42450
H	4.17820	2.32270	-0.30070
H	4.11830	0.68480	1.55660
H	5.72680	0.75330	0.84060
H	5.22530	-1.29860	-0.50280
H	3.60820	-1.36040	0.18440
H	6.22250	-1.60640	1.79260
H	5.13920	-2.94620	1.38720
H	-1.96460	-0.65290	1.44160
H	4.59680	-1.66920	2.48550

[6.5]PCP 2 StrainViz fragment r9

Cartesian coordinates

C	1.71820	0.25240	1.67630
C	2.78820	0.57310	0.84750
C	2.53170	1.25680	-0.35360
C	1.22940	1.59790	-0.68780
C	0.15870	1.29960	0.16510
C	0.39900	0.60810	1.36190
H	1.93230	-0.28190	2.59520
H	0.99530	2.11180	-1.61220
C	-0.65960	-2.16900	-0.29030
C	-0.53210	-2.15410	-1.67930

C	-1.65420	-1.91970	-2.46960
C	-2.89470	-1.69320	-1.87420
C	-3.03310	-1.69420	-0.48450
C	-1.89990	-1.94230	0.29720
H	0.21110	-2.34890	0.33560
H	-1.56520	-1.90820	-3.55180
H	-3.76650	-1.50160	-2.49570
H	-1.99560	-1.94830	1.38070
C	-1.18060	1.75210	-0.31050
O	-1.42670	1.96230	-1.48130
O	-2.05580	1.97430	0.67470
C	2.89600	-2.32440	-0.72850
H	2.32030	-3.25550	-0.68020
H	2.19080	-1.48900	-0.67670
C	-0.42080	-0.55360	3.26130
O	-0.65590	0.29210	2.15280
H	0.03060	-1.50330	2.94810
H	0.22530	-0.07290	4.00420
H	-1.39900	-0.74320	3.70350
O	3.62190	1.54040	-1.12260
C	3.39260	2.20890	-2.34770
H	2.93360	3.19060	-2.18380
H	4.37040	2.33740	-2.81080
H	2.74960	1.61530	-3.00760
C	3.93310	-2.25650	0.39320
C	4.77670	-0.97260	0.37100
C	4.19670	0.17930	1.21180
H	3.43160	-2.34790	1.36690
H	4.59520	-3.12560	0.30920
H	5.77960	-1.18070	0.76040

H	4.90460	-0.63430	-0.66340
H	4.85000	1.05260	1.10500
H	4.21350	-0.11310	2.26790
C	-4.16190	1.15860	-0.22490
C	-4.27230	0.04660	0.82500
C	-4.34210	-1.35150	0.18770
C	-3.38300	2.36320	0.27920
H	-3.82360	2.77690	1.18910
H	-3.31540	3.14460	-0.48130
H	-3.65260	0.78610	-1.11990
H	-5.15720	1.49130	-0.54130
H	-5.15030	0.21350	1.45930
H	-3.39030	0.07930	1.47730
H	-5.16060	-1.38960	-0.54120
H	-4.56590	-2.09250	0.96380
H	3.38740	-2.28480	-1.70710
H	0.43600	-2.32470	-2.14120

[6.5]PCP 2 StrainViz fragment r10

Cartesian coordinates

C	1.53310	-0.43010	1.66700
C	2.55370	0.26480	1.03230
C	2.21500	1.36560	0.22440
C	0.89580	1.77470	0.13530
C	-0.12980	1.09070	0.80560
C	0.18490	-0.05480	1.55280
H	1.80670	-1.28770	2.27190
H	0.60300	2.63170	-0.45900
C	0.00140	-2.05520	-1.06840
C	0.48550	-1.00170	-1.84700

C	-0.40920	-0.10820	-2.43140
C	-1.77910	-0.26680	-2.22920
C	-2.28540	-1.30120	-1.43920
C	-1.36530	-2.18670	-0.86330
H	0.70560	-2.75780	-0.63360
H	-0.03660	0.71170	-3.04130
H	-2.46340	0.42890	-2.70850
H	-1.73250	-3.00480	-0.24650
C	-1.49420	1.64800	0.59880
O	-1.74150	2.49820	-0.23530
O	-2.41670	1.16170	1.43510
O	1.83600	-0.91050	-2.01690
C	-0.49440	-2.02890	2.67620
O	-0.81470	-0.76460	2.12940
H	0.01220	-2.66050	1.93700
H	0.13530	-1.93480	3.56800
H	-1.44590	-2.48230	2.95390
O	3.25320	1.97190	-0.42510
C	2.94620	3.10390	-1.21380
H	2.50430	3.90310	-0.60790
H	3.89020	3.44590	-1.63690
H	2.25420	2.84650	-2.02520
C	3.88200	-2.40070	0.01080
C	4.49750	-1.00590	0.00800
C	3.99730	-0.14310	1.17880
H	4.16240	-2.94800	0.91820
H	4.21210	-2.98370	-0.85400
H	5.58930	-1.08130	0.07130
H	4.26350	-0.49570	-0.93220
H	4.61140	0.75990	1.25050

H	4.12000	-0.70110	2.11490
C	-4.28990	0.77100	-0.04740
C	-4.20360	-0.75420	0.12310
C	-3.76370	-1.46800	-1.16830
C	-3.77800	1.53190	1.16570
H	-4.31840	1.25650	2.07410
H	-3.83830	2.61270	1.01830
H	-3.69440	1.09350	-0.90680
H	-5.32180	1.08000	-0.24760
H	-5.17290	-1.14700	0.44930
H	-3.47670	-0.99510	0.90900
H	-4.34840	-1.08450	-2.01370
H	-3.99120	-2.53660	-1.08490
H	2.79140	-2.33370	-0.02780
H	2.04990	-0.06110	-2.43200

[6.5]PCP 2 StrainViz fragment r11

Cartesian coordinates

C	1.60390	-0.23860	1.39500
C	2.72240	0.31000	0.77980
C	2.56780	1.48020	0.01550
C	1.30840	2.03640	-0.14190
C	0.18220	1.47880	0.47890
C	0.32620	0.32400	1.26430
H	1.73980	-1.14140	1.98040
H	1.14900	2.91680	-0.75220
C	-0.82460	-2.98010	-0.23980
C	0.14010	-2.25190	-0.93630
C	-0.24650	-1.13410	-1.68170
C	-1.57940	-0.74830	-1.72070

C	-2.56470	-1.45520	-1.01600
C	-2.15630	-2.56780	-0.28260
H	-0.55860	-3.86150	0.33280
H	0.51870	-0.57300	-2.21000
H	-1.85520	0.11780	-2.31740
H	-2.89790	-3.13950	0.27110
C	-1.10830	2.14670	0.15520
O	-1.24020	2.87830	-0.80770
O	-2.09840	1.90010	1.01930
C	1.89080	-3.63220	-0.13600
O	1.46740	-2.56200	-0.95490
H	1.43930	-4.58010	-0.45030
H	1.64290	-3.44740	0.91700
C	-0.59690	-1.29860	2.72850
O	-0.77490	-0.22170	1.83050
H	-0.17450	-2.17470	2.22550
H	0.04570	-1.01010	3.56840
H	-1.59310	-1.54590	3.09560
O	3.70860	1.99600	-0.52900
C	3.57900	3.16650	-1.31120
H	3.17080	3.99480	-0.72060
H	4.58410	3.42000	-1.64710
H	2.93540	2.99300	-2.18120
C	4.52190	-0.96710	-0.44380
C	4.07230	-0.35210	0.88840
H	5.44740	-1.53580	-0.31350
H	4.69900	-0.18400	-1.18420
H	4.81560	0.38340	1.21650
H	4.02190	-1.12990	1.65780
C	-3.99010	1.48470	-0.42700

C	-4.26950	0.06400	0.08570
C	-4.01340	-1.02350	-0.97800
C	-3.39490	2.39180	0.63820
H	-3.97960	2.37070	1.56060
H	-3.30120	3.42160	0.28780
H	-3.28720	1.46250	-1.26580
H	-4.91060	1.93990	-0.80950
H	-5.30600	0.00360	0.43520
H	-3.62720	-0.14630	0.94940
H	-4.33170	-0.65470	-1.96100
H	-4.63080	-1.90060	-0.75670
H	3.74840	-1.63340	-0.83490
H	2.97370	-3.69400	-0.24580

[6.5]PCP 2 StrainViz fragment r12

Cartesian coordinates

C	1.14520	2.06570	1.02780
C	2.30280	1.71590	0.34260
C	2.18340	1.03910	-0.88170
C	0.92830	0.80480	-1.41900
C	-0.23780	1.19460	-0.74820
C	-0.13290	1.79700	0.51750
H	1.25320	2.55900	1.98700
H	0.80110	0.29830	-2.36790
C	0.28910	-1.34480	1.59670
C	0.81850	-1.89740	0.42840
C	-0.04570	-2.42890	-0.53270
C	-1.41710	-2.41680	-0.32180
C	-1.97360	-1.86250	0.83810
C	-1.09380	-1.32480	1.77850

H	0.93370	-0.92390	2.35980
H	0.38060	-2.85790	-1.43440
H	-2.06900	-2.85910	-1.07130
H	-1.49590	-0.88850	2.69120
C	-1.50790	0.87500	-1.45780
O	-1.55480	0.18310	-2.45610
O	-2.58830	1.45080	-0.92080
C	3.06130	-1.60230	1.16700
O	2.14870	-1.96810	0.14140
H	2.89400	-2.23890	2.04660
H	2.88500	-0.56090	1.46330
C	-1.12440	2.46230	2.56660
O	-1.26240	2.08010	1.21200
H	-0.54660	1.71880	3.12860
H	-0.64610	3.44330	2.66320
H	-2.13740	2.51560	2.96510
O	3.35570	0.66460	-1.47030
C	3.25930	-0.21950	-2.57250
H	2.78260	0.26620	-3.43150
H	4.28250	-0.49000	-2.83460
H	2.69710	-1.11760	-2.29310
C	4.46100	-1.77500	0.61710
C	3.66860	2.03820	0.88320
H	5.19820	-1.53060	1.38700
H	4.62230	-2.80720	0.29510
H	4.18940	2.73720	0.22020
H	3.60120	2.48630	1.87690
C	-4.16580	-0.38590	-0.92010
C	-4.10250	-0.50080	0.61160
C	-3.46700	-1.82320	1.07600

C	-3.86190	1.01780	-1.42200
H	-4.56930	1.75110	-1.02860
H	-3.85930	1.06270	-2.51380
H	-3.43790	-1.06020	-1.38080
H	-5.15190	-0.68620	-1.29140
H	-5.10880	-0.40810	1.03490
H	-3.50350	0.32300	1.01880
H	-3.94960	-2.66120	0.55760
H	-3.66160	-1.95850	2.14590
H	4.29190	1.13980	0.94080
H	4.60980	-1.10680	-0.23530

[6.5]PCP 2 StrainViz fragment r13

Cartesian coordinates

C	0.43050	2.39010	1.31150
C	1.66480	2.25560	0.69430
C	1.77040	1.66370	-0.56550
C	0.61150	1.24740	-1.20710
C	-0.64340	1.38840	-0.60460
C	-0.74220	1.94430	0.68570
H	0.38970	2.84060	2.29510
H	0.63480	0.77940	-2.18310
C	0.53280	-1.09330	1.47170
C	1.21950	-1.58940	0.36130
C	0.52470	-2.33000	-0.59940
C	-0.82650	-2.60170	-0.42890
C	-1.53000	-2.13950	0.69010
C	-0.82590	-1.36950	1.61770
H	1.03800	-0.48180	2.21060
H	1.06930	-2.70390	-1.46100

H	-1.34210	-3.20520	-1.17260
H	-1.34860	-0.98040	2.48970
C	-1.77860	0.85280	-1.41310
O	-1.61880	0.26630	-2.46510
O	-2.98430	1.08360	-0.88720
C	3.34900	-0.93870	1.21810
O	2.55260	-1.41270	0.13780
H	3.01690	-1.41000	2.15140
H	3.22430	0.14870	1.31330
C	-2.00600	2.46340	2.62500
O	-1.95600	2.01520	1.28420
H	-1.38510	1.83660	3.27580
H	-1.68510	3.50740	2.71190
H	-3.04960	2.38020	2.92670
O	3.02450	1.54390	-1.08110
C	3.13260	0.89560	-2.33460
H	2.58320	1.44220	-3.10980
H	4.19370	0.88470	-2.58170
H	2.75850	-0.13290	-2.27040
C	4.79840	-1.29900	0.94530
C	5.33800	-0.69750	-0.34770
H	5.38840	-0.95050	1.80080
H	4.88720	-2.39110	0.92010
H	6.39940	-0.92870	-0.47410
H	4.79700	-1.09720	-1.20940
C	-3.97690	-1.11330	-1.04100
C	-3.91460	-1.28810	0.48370
C	-2.99620	-2.44760	0.90380
C	-4.07730	0.34670	-1.45870
H	-4.96910	0.82610	-1.04950

H	-4.06690	0.45960	-2.54520
H	-3.07520	-1.52620	-1.50470
H	-4.82750	-1.66180	-1.46060
H	-4.92210	-1.45220	0.88240
H	-3.53240	-0.36810	0.94290
H	-3.26930	-3.35120	0.34470
H	-3.16300	-2.66990	1.96400
H	5.21490	0.39030	-0.34910
H	2.56750	2.60390	1.18730

Fully Optimized StrainViz Fragments of [6.5]PCP 13

Parent

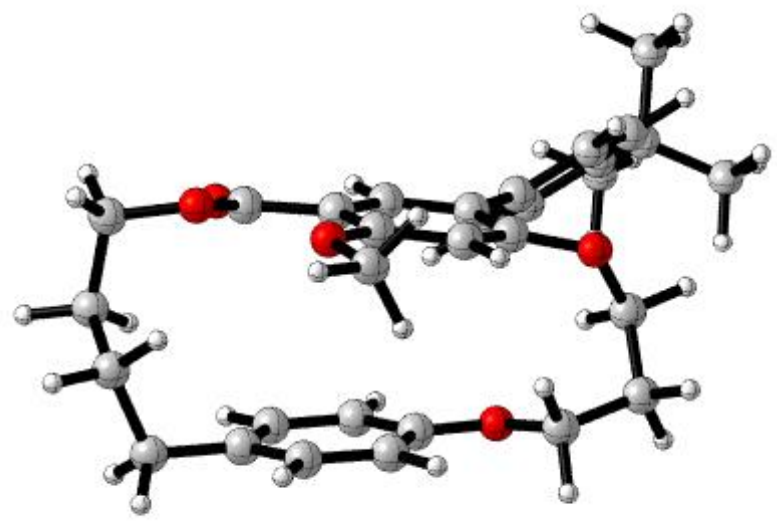

Fragment 1 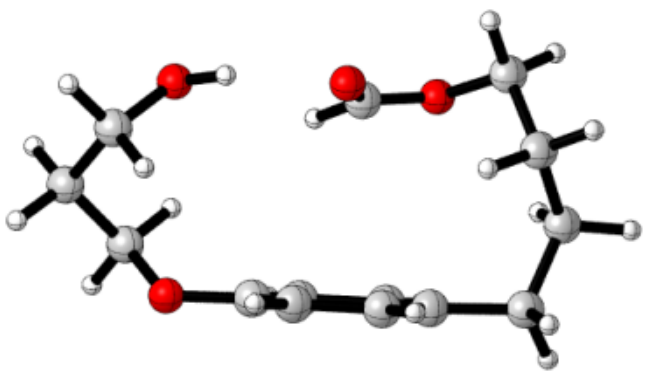	Fragment 2 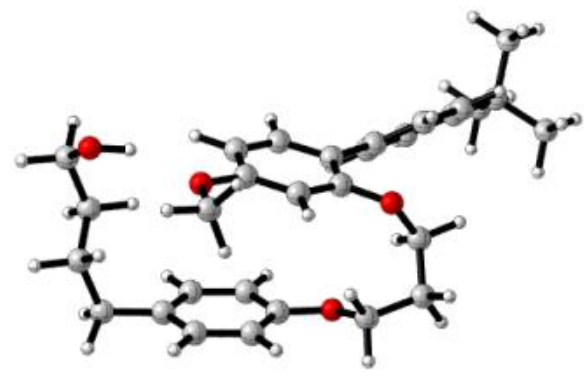	Fragment 3 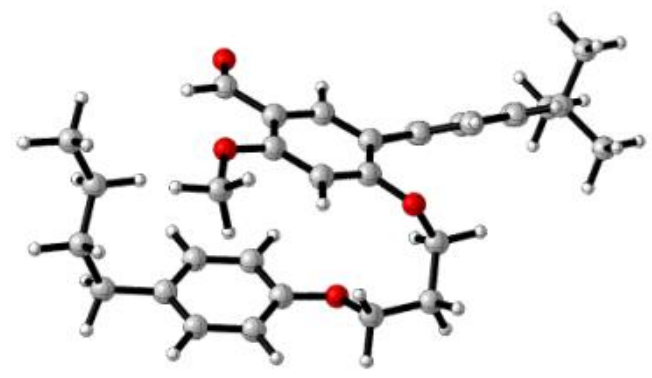
Fragment 4 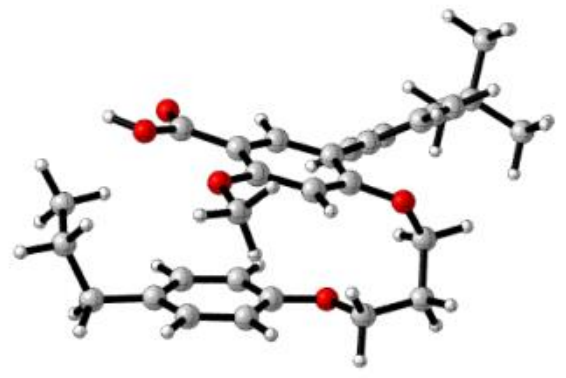	Fragment 5 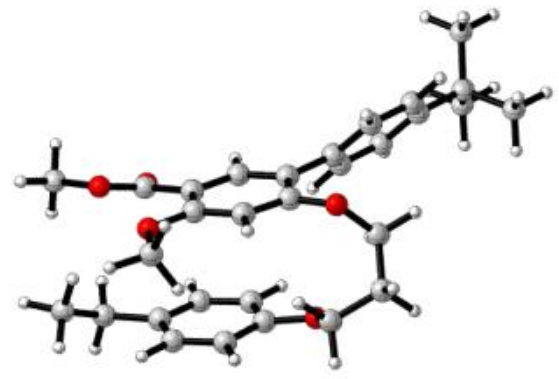	Fragment 6 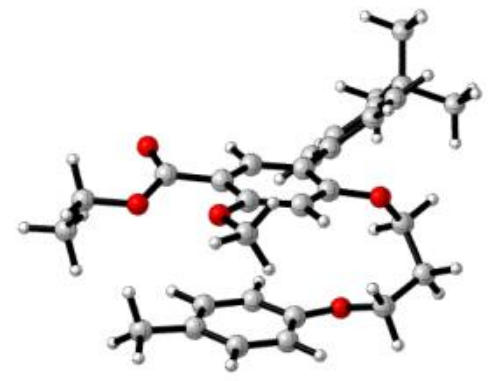
Fragment 7 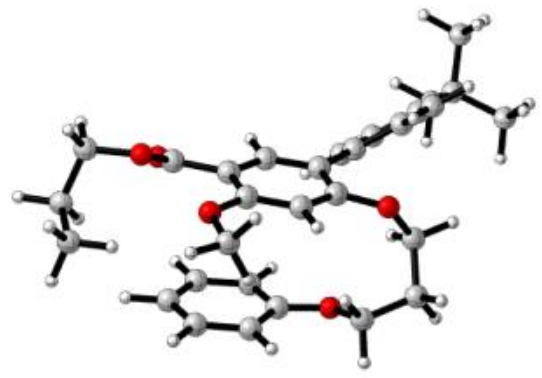	Fragment 8 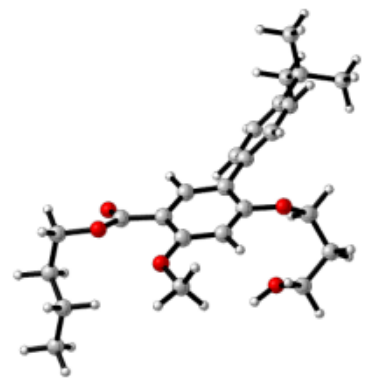	Fragment 9 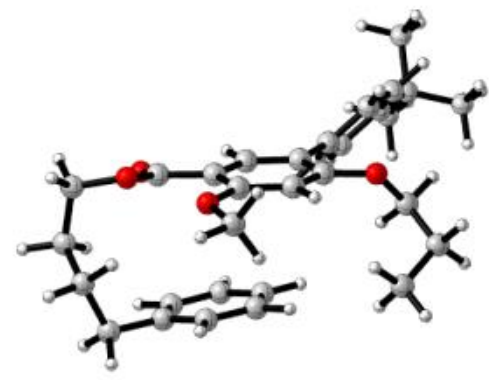
Fragment 10 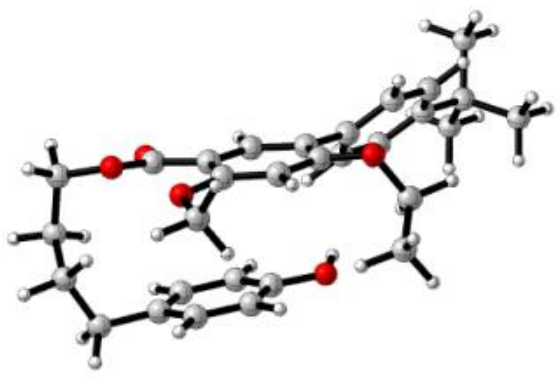	Fragment 11 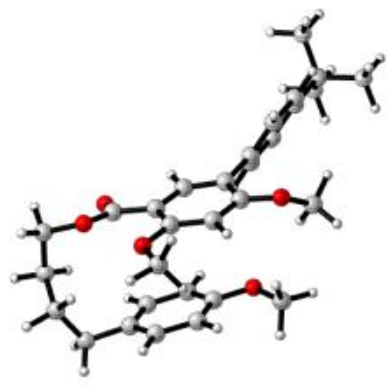	Fragment 12 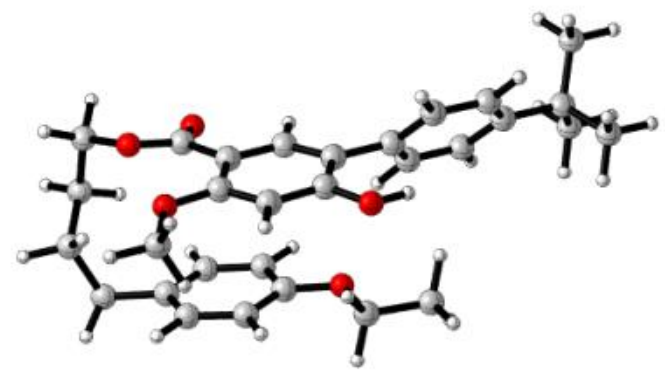
Fragment 13 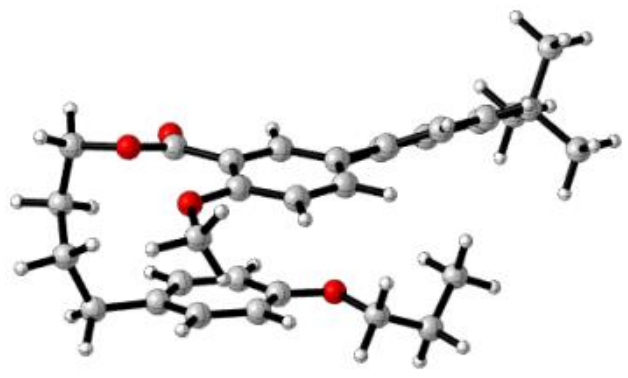		

[6.5]PCP 13 StrainViz fragment r1

Cartesian coordinates

C	-0.57330	-1.54360	1.26530
C	-1.23340	-1.47540	0.03810
C	-0.49250	-1.50260	-1.14690
C	0.89750	-1.57000	-1.08670
C	1.57700	-1.62980	0.13340

C	0.81420	-1.62190	1.30460
H	-1.16360	-1.52680	2.17610
H	-0.99640	-1.48280	-2.10820
H	1.46470	-1.58400	-2.01510
H	1.31690	-1.66780	2.26780
C	0.51750	1.67150	-0.44480
O	0.27750	1.91590	0.71810
O	1.71120	1.73430	-1.01350
C	-3.27630	-0.62770	-0.89490
O	-2.59510	-1.43090	0.07130
H	-3.83310	-1.28370	-1.57190
H	-2.55450	-0.04770	-1.47660
C	-4.19680	0.32370	-0.14810
C	-3.40390	1.21380	0.79880
H	-4.73400	0.94080	-0.87770
H	-4.94140	-0.24320	0.42160
H	-4.08550	1.88700	1.33660
H	-2.89950	0.58410	1.54240
C	3.28710	0.86440	0.62580
C	3.68960	-0.31060	-0.28370
C	3.08770	-1.64900	0.17330
C	2.82550	2.07990	-0.16080
H	3.59390	2.42890	-0.85310
H	2.52450	2.89930	0.49550
H	2.47090	0.57010	1.29450
H	4.12290	1.17030	1.26380
H	4.77990	-0.39860	-0.33040
H	3.34760	-0.11480	-1.30700
H	3.42320	-1.87310	1.19280
H	3.46800	-2.45040	-0.47100

O	-2.46000	1.95850	0.04760
H	-0.24860	1.38790	-1.17630
H	-1.64350	2.04660	0.56860

[6.5]PCP 13 StrainViz fragment r2

Cartesian coordinates

C	1.01370	0.41830	1.66970
C	-0.33570	0.47020	1.31130
C	-0.94100	-0.57860	0.60120
C	-0.12090	-1.65300	0.22410
C	1.22280	-1.71200	0.55110
C	1.79290	-0.66800	1.28420
H	1.42450	1.24850	2.23150
H	-0.57270	-2.48510	-0.30870
C	1.73240	0.92210	-1.92540
C	1.73450	2.04030	-1.08790
C	2.93690	2.49910	-0.54910
C	4.12080	1.82260	-0.84550
C	4.14150	0.70270	-1.67540
C	2.92260	0.26910	-2.21250
H	0.78430	0.57470	-2.32460
H	2.96390	3.37590	0.08860
H	5.05440	2.18710	-0.42120
H	2.90800	-0.60080	-2.86560
O	5.15320	-2.74170	1.49210
C	0.42430	3.53670	0.22540
O	0.52390	2.61900	-0.85370
H	0.87550	4.49900	-0.04770
H	0.96180	3.13940	1.09420
C	3.72900	0.22290	2.36390

O	3.11660	-0.79050	1.58070
H	4.76720	-0.08820	2.48400
H	3.68660	1.18670	1.84750
H	3.24880	0.30000	3.34510
C	-1.04920	3.69310	0.55970
C	-1.72780	2.34230	0.74250
H	-1.13540	4.29150	1.47260
H	-1.56760	4.23240	-0.24100
H	-2.75980	2.46580	1.08320
H	-1.74080	1.80030	-0.20510
C	4.82190	-2.26040	-0.88820
C	5.77630	-1.07170	-0.88090
C	5.43770	-0.02210	-1.95080
C	5.15200	-3.29140	0.18650
H	6.15860	-3.69280	0.02300
H	4.44870	-4.13580	0.11910
H	3.79540	-1.90000	-0.74260
H	4.84770	-2.75440	-1.86930
H	6.80190	-1.43040	-1.03910
H	5.75260	-0.59980	0.10900
H	5.38690	-0.51220	-2.93130
H	6.25270	0.70870	-2.00400
C	-2.39430	-0.62360	0.30090
C	-3.35050	-0.28380	1.26860
C	-2.85440	-1.05120	-0.94600
C	-4.70680	-0.37210	0.98850
H	-3.02060	0.05070	2.24800
C	-4.21850	-1.14000	-1.22090
H	-2.13580	-1.30550	-1.72120
C	-5.17620	-0.80270	-0.26180

H	-5.41640	-0.10450	1.76730
H	-4.52460	-1.47340	-2.20660
C	-6.68240	-0.88380	-0.52110
C	-7.30650	0.51040	-0.34030
H	-8.38640	0.46520	-0.52000
H	-7.14980	0.89410	0.67250
H	-6.86990	1.22480	-1.04610
C	-7.32000	-1.85990	0.48230
H	-7.16430	-1.53520	1.51570
H	-8.39990	-1.92520	0.30880
H	-6.89270	-2.86210	0.37290
C	-6.99850	-1.37380	-1.93770
H	-8.08370	-1.41630	-2.07500
H	-6.59050	-0.69940	-2.69790
H	-6.59850	-2.37770	-2.11530
O	-1.03230	1.57390	1.73420
H	4.30530	-2.27740	1.60090
H	1.83350	-2.56510	0.27370

[6.5]PCP 13 StrainViz fragment r3

Cartesian coordinates

C	0.91220	0.19000	1.61600
C	-0.42560	0.35040	1.26020
C	-1.12730	-0.65220	0.55600
C	-0.40240	-1.78010	0.18010
C	0.94630	-1.95290	0.49300
C	1.60720	-0.95600	1.23560
H	1.39200	0.99810	2.15430
H	-0.89840	-2.58340	-0.35840
C	2.13830	0.83040	-1.51690

C	2.02850	2.06900	-0.87750
C	3.18120	2.75740	-0.49950
C	4.43230	2.20250	-0.78110
C	4.56440	0.97270	-1.42440
C	3.38970	0.29620	-1.78380
H	1.22660	0.29990	-1.77750
H	3.11850	3.71910	-0.00240
H	5.32670	2.75020	-0.49190
H	3.46050	-0.66830	-2.28190
C	1.65270	-3.16250	0.03960
O	1.12560	-4.04530	-0.61160
C	0.56730	3.50280	0.33930
O	0.76130	2.52890	-0.67570
H	1.01370	4.46150	0.04830
H	1.05510	3.16260	1.26200
C	3.65360	-0.08740	2.05850
O	2.90820	-1.17050	1.52240
H	4.68570	-0.43380	2.11130
H	3.58850	0.78620	1.40070
H	3.30250	0.16850	3.06370
C	-0.93040	3.65890	0.55150
C	-1.64470	2.31880	0.65070
H	-1.08970	4.24620	1.46160
H	-1.37540	4.21480	-0.28120
H	-2.69350	2.45470	0.92840
H	-1.60560	1.79980	-0.30920
C	5.61910	-1.79340	-0.37530
C	6.44050	-0.51900	-0.55950
C	5.92820	0.38170	-1.69510
C	6.16910	-2.69260	0.72910

H	7.19190	-3.01300	0.50310
H	5.55550	-3.58940	0.85750
H	4.57960	-1.53090	-0.14760
H	5.60060	-2.34610	-1.32470
H	7.48430	-0.78890	-0.76620
H	6.44570	0.05550	0.37790
H	5.89690	-0.20340	-2.62280
H	6.64520	1.19440	-1.85720
C	-2.58040	-0.56900	0.26230
C	-3.49710	-0.14930	1.23620
C	-3.08030	-0.95370	-0.98280
C	-4.85750	-0.12020	0.96310
H	-3.13490	0.15350	2.21460
C	-4.44820	-0.92370	-1.25020
H	-2.39020	-1.26960	-1.76100
C	-5.36800	-0.50650	-0.28540
H	-5.53740	0.20550	1.74620
H	-4.78690	-1.22850	-2.23440
C	-6.87680	-0.45780	-0.53700
C	-7.37930	0.98360	-0.34690
H	-8.45970	1.03090	-0.52270
H	-7.18730	1.34730	0.66720
H	-6.88630	1.66210	-1.05100
C	-7.58940	-1.38120	0.46590
H	-7.40130	-1.07550	1.49960
H	-8.67180	-1.35380	0.29810
H	-7.24910	-2.41540	0.34970
C	-7.24040	-0.91250	-1.95400
H	-8.32590	-0.86140	-2.08560
H	-6.78010	-0.27180	-2.71360

H	-6.92880	-1.94610	-2.13800
O	-1.01710	1.51230	1.66060
H	6.19540	-2.16660	1.69030
H	2.71790	-3.23380	0.32130

[6.5]PCP 13 StrainViz fragment r4

Cartesian coordinates

C	1.03300	1.41720	1.70510
C	-0.27480	1.26840	1.24970
C	-0.77810	0.00350	0.88980
C	0.11830	-1.05890	0.94250
C	1.44730	-0.93620	1.35230
C	1.91540	0.33510	1.74740
H	1.34960	2.41260	1.98840
H	-0.22660	-2.05480	0.68270
C	1.67140	0.10540	-2.04060
C	1.74150	1.41600	-1.55890
C	2.97260	1.93700	-1.15860
C	4.11380	1.14000	-1.24660
C	4.07270	-0.16080	-1.74870
C	2.82370	-0.66300	-2.14070
H	0.70430	-0.28390	-2.34420
H	3.05950	2.95730	-0.80260
H	5.06930	1.56060	-0.94060
H	2.75510	-1.66680	-2.55310
C	2.23520	-2.19080	1.33920
O	1.80430	-3.24390	0.90970
O	3.47160	-2.10490	1.85230
C	0.57210	3.41160	-0.96770

O	0.56850	2.10840	-1.53020
H	1.09640	4.11020	-1.63230
H	1.09530	3.39830	-0.00540
C	3.67040	1.75340	2.47290
O	3.19850	0.46160	2.13300
H	4.72350	1.62740	2.72080
H	3.57130	2.44070	1.62570
H	3.13490	2.15530	3.33950
C	-0.87520	3.82510	-0.75640
C	-1.66680	2.74530	-0.03060
H	-0.88870	4.75990	-0.18680
H	-1.36300	4.01440	-1.71910
H	-2.67330	3.09340	0.21520
H	-1.75080	1.86090	-0.66300
C	4.74440	-3.21270	-0.84550
C	5.60790	-1.95810	-0.74970
C	5.34780	-0.96070	-1.89090
H	3.67720	-2.97990	-0.79150
H	4.92490	-3.73150	-1.79370
H	6.66460	-2.24860	-0.77540
H	5.43490	-1.45510	0.20930
H	5.33580	-1.50850	-2.84230
H	6.18780	-0.25940	-1.94600
C	-2.19670	-0.24260	0.52870
C	-3.24480	0.34120	1.25440
C	-2.52810	-1.09670	-0.52450
C	-4.56690	0.07280	0.92960
H	-3.01530	1.00940	2.07940
C	-3.85880	-1.36440	-0.84350
H	-1.73540	-1.55020	-1.11410

C	-4.90830	-0.78640	-0.12600
H	-5.35160	0.54070	1.51860
H	-4.06490	-2.03190	-1.67300
C	-6.38180	-1.04990	-0.44490
C	-7.07160	0.27850	-0.79930
H	-8.12910	0.10380	-1.02670
H	-7.01900	0.99350	0.02760
H	-6.60250	0.73710	-1.67610
C	-7.06710	-1.66580	0.78680
H	-7.01660	-0.99860	1.65270
H	-8.12400	-1.85860	0.57130
H	-6.59300	-2.61420	1.06010
C	-6.55370	-2.01250	-1.62410
H	-7.62030	-2.17060	-1.81280
H	-6.10710	-1.61150	-2.54020
H	-6.10150	-2.98810	-1.41700
O	-1.03280	2.40470	1.21370
H	4.97420	-3.91980	-0.04020
H	3.85510	-2.99690	1.76890

[6.5]PCP 13 StrainViz fragment r5

Cartesian coordinates

C	-1.21430	1.08940	-1.70740
C	0.12570	1.01300	-1.33460
C	0.65410	-0.16390	-0.77290
C	-0.24590	-1.20210	-0.53850
C	-1.60000	-1.14040	-0.86290
C	-2.09530	0.03220	-1.46950
H	-1.55480	2.01770	-2.14800
H	0.11620	-2.12380	-0.09280

C	-1.30220	0.54650	2.13330
C	-1.53470	1.79790	1.55840
C	-2.82340	2.14130	1.15030
C	-3.85010	1.20160	1.25450
C	-3.62770	-0.07180	1.77770
C	-2.33810	-0.36920	2.23610
H	-0.29170	0.29740	2.44340
H	-3.03050	3.12290	0.73630
H	-4.83910	1.47660	0.89800
H	-2.13330	-1.35990	2.63510
C	-2.41200	-2.33000	-0.49280
O	-2.05460	-3.15000	0.33030
O	-3.55690	-2.45240	-1.17320
C	-0.51170	3.67250	0.48210
O	-0.44590	2.60710	1.42080
H	-0.96710	4.55760	0.94330
H	-1.13050	3.37440	-0.37100
C	-3.91930	1.32620	-2.25570
O	-3.40940	0.10160	-1.76030
H	-4.99430	1.17960	-2.35660
H	-3.72340	2.14360	-1.55300
H	-3.48860	1.56840	-3.23330
C	0.90650	3.96080	0.01450
C	1.60660	2.70410	-0.48460
H	0.86730	4.71550	-0.77800
H	1.49840	4.37460	0.83890
H	2.60930	2.93560	-0.85360
H	1.69500	1.98200	0.32890
C	-5.80040	-0.98820	0.78600
C	-4.71560	-1.12280	1.85460

C	-4.32480	-3.61770	-0.87190
H	-5.21450	-3.55150	-1.49580
H	-3.75520	-4.51910	-1.10760
H	-6.47220	-1.85170	0.80960
H	-6.41280	-0.09530	0.94530
H	-4.23390	-2.10530	1.76970
H	-5.17980	-1.10450	2.84980
C	2.09530	-0.34990	-0.47070
C	3.08800	0.06830	-1.36820
C	2.50340	-0.98260	0.70500
C	4.43070	-0.14420	-1.08970
H	2.79830	0.56220	-2.29130
C	3.85420	-1.19460	0.97800
H	1.75590	-1.30460	1.42580
C	4.84870	-0.78060	0.08900
H	5.17020	0.18890	-1.81340
H	4.12060	-1.68670	1.90700
C	6.34190	-0.99050	0.35060
C	7.05000	0.37450	0.38520
H	8.12140	0.23750	0.56930
H	6.93740	0.91160	-0.56170
H	6.64150	1.00300	1.18350
C	6.94140	-1.84570	-0.77870
H	6.82610	-1.36370	-1.75430
H	8.01190	-2.00120	-0.60420
H	6.45420	-2.82530	-0.82350
C	6.59990	-1.70150	1.68280
H	7.67780	-1.83000	1.82440
H	6.21730	-1.12200	2.52970
H	6.13800	-2.69420	1.70580

O	0.88290	2.12520	-1.58280
H	-4.60180	-3.63510	0.18450
H	-5.34720	-0.92200	-0.20870

[6.5]PCP 13 StrainViz fragment r6

Cartesian coordinates

C	-1.19250	1.01370	-1.61650
C	0.14430	0.97130	-1.22230
C	0.70990	-0.21010	-0.70880
C	-0.14970	-1.29440	-0.54510
C	-1.50370	-1.26480	-0.87390
C	-2.03430	-0.08750	-1.44400
H	-1.56140	1.94470	-2.02800
H	0.24790	-2.23000	-0.16360
C	-1.85490	0.79180	1.85040
C	-1.83840	2.10600	1.37680
C	-3.01920	2.69560	0.92940
C	-4.19570	1.94480	0.91410
C	-4.22450	0.61740	1.33440
C	-3.03390	0.06150	1.82050
H	-0.92240	0.34820	2.18680
H	-3.03170	3.72460	0.58650
H	-5.11010	2.40860	0.55080
H	-3.02740	-0.97310	2.15790
C	-2.25850	-2.51480	-0.58780
O	-1.71340	-3.58810	-0.41710
O	-3.58270	-2.35290	-0.49240
C	-0.46950	3.82710	0.46810
O	-0.62750	2.73510	1.36490
H	-0.86610	4.74850	0.91230

H	-1.02540	3.62300	-0.45430
C	-3.83500	1.08200	-2.44620
O	-3.33010	-0.08030	-1.81240
H	-4.88270	0.87100	-2.65850
H	-3.76300	1.95110	-1.78460
H	-3.30220	1.28200	-3.38240
C	1.01050	3.96040	0.15310
C	1.60870	2.63420	-0.29370
H	1.13180	4.71880	-0.62740
H	1.56130	4.30230	1.03650
H	2.64700	2.75840	-0.61340
H	1.58390	1.91960	0.53080
C	-4.29350	-3.86540	1.28630
C	-5.48090	-0.20590	1.22850
C	-4.34210	-3.53720	-0.19430
H	-5.35700	-3.29620	-0.51590
H	-3.95990	-4.36410	-0.79670
H	-3.26930	-4.08550	1.59500
H	-4.91540	-4.74120	1.49230
H	-5.64270	-0.81030	2.12670
H	-6.35990	0.42880	1.08500
C	2.15540	-0.36290	-0.40700
C	3.13580	0.10260	-1.29410
C	2.58010	-1.01460	0.75190
C	4.48400	-0.08110	-1.02100
H	2.83190	0.61110	-2.20490
C	3.93610	-1.19770	1.01980
H	1.84080	-1.37250	1.46430
C	4.91890	-0.73500	0.14190
H	5.21410	0.28920	-1.73610

H	4.21580	-1.70580	1.93620
C	6.41730	-0.91130	0.39900
C	7.08940	0.47080	0.46380
H	8.16390	0.35780	0.64600
H	6.96330	1.02510	-0.47130
H	6.66420	1.07090	1.27510
C	7.03840	-1.72550	-0.74870
H	6.90970	-1.22570	-1.71370
H	8.11280	-1.85660	-0.57780
H	6.57700	-2.71640	-0.81480
C	6.69440	-1.64430	1.71530
H	7.77540	-1.74670	1.85470
H	6.29620	-1.09420	2.57450
H	6.25960	-2.64940	1.71620
O	0.86630	2.11650	-1.40970
H	-4.67300	-3.02720	1.87710
H	-5.40790	-0.88780	0.37390

[6.5]PCP 13 StrainViz fragment r7

Cartesian coordinates

C	-1.16210	1.20490	-1.64460
C	0.18220	1.07370	-1.30260
C	0.67270	-0.11660	-0.73520
C	-0.26640	-1.11060	-0.46360
C	-1.62200	-0.99520	-0.76280
C	-2.08190	0.19080	-1.37030
H	-1.47170	2.14130	-2.09070
H	0.06510	-2.04080	-0.01150
C	-1.22680	0.62790	2.25120
C	-1.40920	1.86780	1.63140

C	-2.69120	2.28880	1.26950
C	-3.77600	1.44010	1.49290
C	-3.59820	0.19690	2.09180
C	-2.31680	-0.20060	2.47810
H	-0.21800	0.32820	2.51790
H	-2.84890	3.26230	0.81770
H	-4.77020	1.76280	1.19530
H	-2.16360	-1.17380	2.93400
C	-2.48440	-2.13750	-0.35810
O	-2.18180	-2.91510	0.52530
O	-3.59830	-2.25460	-1.08790
C	-0.33850	3.69950	0.52550
O	-0.27840	2.59580	1.41880
H	-0.76050	4.57600	1.03290
H	-0.98360	3.45150	-0.32380
C	-3.86860	1.55580	-2.12340
O	-3.40060	0.31750	-1.62180
H	-4.95090	1.45400	-2.19870
H	-3.62260	2.37410	-1.43770
H	-3.45060	1.76650	-3.11380
C	1.07530	3.97100	0.03560
C	1.74420	2.71260	-0.50210
H	1.03230	4.74200	-0.74060
H	1.69170	4.35820	0.85500
H	2.73480	2.93960	-0.90450
H	1.85590	1.98140	0.29990
C	-5.37170	-2.73760	0.49970
C	-6.08540	-1.43110	0.15990
C	-4.54030	-3.25290	-0.66510
H	-5.16290	-3.42910	-1.54550

H	-4.00520	-4.16910	-0.40690
H	-4.71030	-2.60150	1.36290
H	-6.09540	-3.51500	0.77030
H	-6.78060	-1.57420	-0.67480
H	-5.35890	-0.66500	-0.13020
C	2.11130	-0.36170	-0.46410
C	3.10010	0.00440	-1.38850
C	2.51920	-0.99940	0.70890
C	4.43860	-0.26240	-1.13790
H	2.81080	0.50130	-2.31010
C	3.86560	-1.26640	0.95360
H	1.77570	-1.28210	1.45000
C	4.85620	-0.90420	0.03810
H	5.17480	0.03180	-1.88160
H	4.13210	-1.76020	1.88170
C	6.34460	-1.17630	0.26770
C	7.11310	0.15610	0.26470
H	8.18140	-0.02590	0.42610
H	7.00190	0.68400	-0.68750
H	6.75180	0.81360	1.06240
C	6.87770	-2.07350	-0.86230
H	6.75850	-1.60130	-1.84230
H	7.94450	-2.27330	-0.71190
H	6.34720	-3.03120	-0.87960
C	6.60320	-1.87900	1.60420
H	7.67760	-2.05200	1.72280
H	6.26580	-1.27160	2.45070
H	6.09980	-2.85040	1.65290
O	0.98100	2.14970	-1.58140
H	-6.65790	-1.05970	1.01540

H	-4.45050	-0.45170	2.26710

[6.5]PCP 13 StrainViz fragment r8

Cartesian coordinates

C	-1.32070	1.68260	-1.01660
C	0.02380	1.40640	-0.76800
C	0.44060	0.11940	-0.38310
C	-0.56020	-0.83610	-0.21160
C	-1.91220	-0.58870	-0.44400
C	-2.30110	0.70270	-0.86090
H	-1.57110	2.69050	-1.32110
H	-0.28320	-1.84380	0.08310
C	-2.83280	-1.73880	-0.22620
O	-2.55100	-2.68210	0.48550
O	-3.97190	-1.65840	-0.92030
C	-0.07900	4.17580	1.13140
O	-0.26880	3.08030	2.01190
H	-0.30350	5.12870	1.62970
H	-0.73860	4.09130	0.25850
C	-4.00140	2.26420	-1.41010
O	-3.61460	0.95040	-1.04640
H	-5.08730	2.24120	-1.48930
H	-3.70360	2.98990	-0.64490
H	-3.56880	2.55100	-2.37420
C	1.36910	4.15830	0.66760
C	1.76720	2.79610	0.12170
H	1.51000	4.93070	-0.09590
H	2.03470	4.39480	1.50610
H	2.78810	2.80730	-0.27010
H	1.70470	2.04420	0.91030

C	-5.72580	-2.41850	0.58870
C	-6.43200	-1.06360	0.56660
C	-7.20820	-0.79890	1.85390
C	-4.94010	-2.69470	-0.68350
H	-5.58980	-2.66220	-1.56120
H	-4.43070	-3.65960	-0.64240
H	-5.04510	-2.47470	1.44660
H	-6.46150	-3.22330	0.71320
H	-7.11390	-1.02780	-0.29320
H	-5.68720	-0.27540	0.40580
H	-7.97240	-1.56670	2.01540
H	-7.70860	0.17330	1.82750
C	1.86610	-0.26170	-0.21990
C	2.83630	0.13850	-1.14930
C	2.27390	-1.06960	0.84280
C	4.15850	-0.25900	-1.00950
H	2.54530	0.76520	-1.98750
C	3.60370	-1.46700	0.97640
H	1.54570	-1.38230	1.58660
C	4.57650	-1.07090	0.05580
H	4.88050	0.06760	-1.75370
H	3.87160	-2.09080	1.82210
C	6.04660	-1.48140	0.16670
C	6.92050	-0.22070	0.27900
H	7.97690	-0.50120	0.35640
H	6.80760	0.42720	-0.59560
H	6.65230	0.35870	1.16870
C	6.45110	-2.27020	-1.09050
H	6.32720	-1.67260	-1.99890
H	7.50330	-2.56850	-1.02440

H	5.84270	-3.17480	-1.19290
C	6.30910	-2.36120	1.39310
H	7.37010	-2.62810	1.42950
H	6.06240	-1.83940	2.32380
H	5.73080	-3.29040	1.35370
O	0.89290	2.43870	-0.96260
H	-6.53890	-0.80940	2.72080
H	-1.21950	2.94040	2.12090

[6.5]PCP 13 StrainViz fragment r9

Cartesian coordinates

C	-0.92250	1.76940	-1.40880
C	0.40160	1.49560	-1.07220
C	0.81960	0.18400	-0.78630
C	-0.16100	-0.80470	-0.82300
C	-1.49560	-0.56370	-1.14520
C	-1.88740	0.76040	-1.44070
H	-1.17580	2.80200	-1.61160
H	0.11720	-1.83240	-0.61010
C	-1.60490	-0.62560	2.43970
C	-1.30360	0.68610	2.08500
C	-2.32340	1.52910	1.64460
C	-3.62850	1.05590	1.55170
C	-3.94830	-0.25860	1.91190
C	-2.91790	-1.09030	2.35900
H	-0.81890	-1.29190	2.78370
H	-2.09810	2.55610	1.36990
H	-4.42080	1.71630	1.20320
H	-3.14590	-2.11150	2.65560
C	-2.38960	-1.75150	-1.09940

O	-2.04940	-2.81540	-0.61970
O	-3.59020	-1.55660	-1.65560
C	0.24790	4.30120	1.13130
H	0.01730	5.30630	1.49450
H	-0.45450	4.06240	0.32620
C	-3.58590	2.36630	-1.82760
O	-3.18490	1.01400	-1.70540
H	-4.66790	2.34410	-1.95340
H	-3.33160	2.93360	-0.92450
H	-3.12600	2.84220	-2.70020
C	1.68730	4.20420	0.63550
C	2.02770	2.81950	0.11230
H	1.86590	4.93320	-0.16280
H	2.38600	4.44010	1.44660
H	3.08540	2.74360	-0.15690
H	1.81440	2.05510	0.86860
C	-5.06620	-2.54030	-0.01420
C	-5.69610	-1.18440	0.33970
C	-5.37430	-0.74370	1.77890
C	-4.56010	-2.59580	-1.44730
H	-5.35240	-2.37630	-2.16640
H	-4.11180	-3.56400	-1.68090
H	-4.21290	-2.74640	0.63990
H	-5.78460	-3.35290	0.14080
H	-6.78190	-1.23060	0.20150
H	-5.31520	-0.41450	-0.34280
H	-5.55530	-1.57780	2.46780
H	-6.05430	0.06470	2.06990
C	2.22890	-0.18050	-0.49880
C	3.28350	0.30680	-1.28250

C	2.53830	-1.05590	0.54340
C	4.59290	-0.07400	-1.02370
H	3.06820	0.98880	-2.10060
C	3.85540	-1.43600	0.79740
H	1.73810	-1.43660	1.17380
C	4.91240	-0.95390	0.02160
H	5.38380	0.32030	-1.65660
H	4.04630	-2.11510	1.62120
C	6.37230	-1.34270	0.26670
C	7.19420	-0.07650	0.56230
H	8.24270	-0.34160	0.73780
H	7.16120	0.63020	-0.27270
H	6.81430	0.43260	1.45430
C	6.93300	-2.03140	-0.98920
H	6.89310	-1.37270	-1.86220
H	7.97930	-2.31350	-0.82750
H	6.36390	-2.93780	-1.22070
C	6.52280	-2.30250	1.45110
H	7.58030	-2.55060	1.58680
H	6.16160	-1.85330	2.38230
H	5.97790	-3.23760	1.28390
O	1.25040	2.56340	-1.06860
H	-0.28010	1.04760	2.14290
H	0.07160	3.59710	1.95320

[6.5]PCP 13 StrainViz fragment r10

Cartesian coordinates

C	-0.95900	2.12820	-1.16140
C	0.38080	1.79310	-1.00040
C	0.79290	0.44930	-0.94410

C	-0.19850	-0.51290	-1.11050
C	-1.54780	-0.21110	-1.30270
C	-1.94360	1.14220	-1.29210
H	-1.20700	3.18210	-1.16690
H	0.07190	-1.56430	-1.10290
C	-1.25810	-1.01060	2.10280
C	-1.02190	0.36190	2.06920
C	-2.09810	1.24580	1.96300
C	-3.39100	0.74840	1.87820
C	-3.65550	-0.62690	1.91710
C	-2.56520	-1.49150	2.02920
H	-0.42560	-1.70400	2.20060
H	-1.90610	2.31440	1.95230
H	-4.22280	1.44560	1.79540
H	-2.73290	-2.56470	2.07850
C	-2.44830	-1.38680	-1.44400
O	-2.07650	-2.52830	-1.25150
O	-3.69250	-1.08370	-1.82820
O	0.22950	0.89970	2.15980
C	-3.64230	2.78900	-1.18010
O	-3.25430	1.44210	-1.38320
H	-4.73170	2.78840	-1.19390
H	-3.28540	3.15630	-0.21100
H	-3.26760	3.43790	-1.97890
C	0.87240	3.96530	1.17120
C	1.82230	3.09900	0.36840
H	0.67610	4.90650	0.65010
H	1.30270	4.18810	2.15200
H	2.76990	3.60990	0.18000
H	2.03220	2.16110	0.89070

C	-5.01310	-2.45660	-0.33830
C	-5.59290	-1.23570	0.39350
C	-5.08330	-1.12110	1.84070
C	-4.64570	-2.15770	-1.78340
H	-5.50150	-1.78470	-2.35030
H	-4.22920	-3.03440	-2.28430
H	-4.10490	-2.80500	0.16380
H	-5.72170	-3.29190	-0.31950
H	-6.68720	-1.28510	0.39140
H	-5.31110	-0.31920	-0.13940
H	-5.16920	-2.09630	2.33570
H	-5.72630	-0.42810	2.39490
C	2.19440	0.04060	-0.68090
C	3.29290	0.68660	-1.26670
C	2.45450	-1.03010	0.17920
C	4.58760	0.26880	-0.99270
H	3.12470	1.51970	-1.94080
C	3.75800	-1.44420	0.45090
H	1.62240	-1.55850	0.64140
C	4.85530	-0.80270	-0.12660
H	5.41190	0.79110	-1.47140
H	3.90350	-2.27850	1.12830
C	6.30280	-1.21880	0.14390
C	7.06970	-0.03090	0.74960
H	8.10910	-0.31620	0.94640
H	7.07950	0.82990	0.07390
H	6.61440	0.28330	1.69460
C	6.96840	-1.63250	-1.17970
H	6.97310	-0.81100	-1.90260
H	8.00760	-1.92970	-1.00040

H	6.44100	-2.47950	-1.63060
C	6.39120	-2.39760	1.11830
H	7.44200	-2.65740	1.28120
H	5.95240	-2.15030	2.09080
H	5.88390	-3.28480	0.72500
O	1.28370	2.81900	-0.93440
H	0.89260	0.19690	2.06880
H	-0.07280	3.43900	1.32700

[6.5]PCP 13 StrainViz fragment r11

Cartesian coordinates

C	-0.84800	1.53070	-1.68260
C	0.47600	1.40410	-1.26080
C	0.95490	0.18850	-0.74120
C	0.02680	-0.84060	-0.60930
C	-1.30360	-0.74650	-1.01500
C	-1.74870	0.46840	-1.58000
H	-1.15110	2.49510	-2.07030
H	0.35390	-1.79180	-0.20100
C	-1.73660	0.32420	2.23480
C	-1.71080	1.59650	1.65820
C	-2.87010	2.10810	1.07040
C	-4.03530	1.34570	1.07640
C	-4.09210	0.08400	1.67100
C	-2.91450	-0.41430	2.24220
H	-0.82680	-0.06230	2.68360
H	-2.87720	3.09160	0.61450
H	-4.93230	1.75660	0.61680
H	-2.92110	-1.39080	2.72080
C	-2.12860	-1.95680	-0.76600

O	-1.75280	-2.87140	-0.05770
O	-3.30380	-1.97200	-1.40480
C	-0.55160	3.63170	1.32280
O	-0.53110	2.27650	1.72930
H	-1.34960	4.18410	1.83120
H	-0.68030	3.71690	0.23600
C	-3.48330	1.81260	-2.47580
O	-3.03420	0.56870	-1.97180
H	-4.53910	1.67460	-2.70660
H	-3.37220	2.60410	-1.72670
H	-2.94160	2.09090	-3.38640
C	2.13350	2.89910	-0.37030
H	3.16260	2.63560	-0.62260
H	1.85410	2.41620	0.57050
C	-4.79750	-2.77430	0.32030
C	-5.61510	-1.47390	0.36730
C	-5.39520	-0.68430	1.66990
C	-4.19320	-3.04330	-1.04840
H	-4.95240	-3.04030	-1.83370
H	-3.64770	-3.98920	-1.07070
H	-3.97240	-2.73380	1.03870
H	-5.41830	-3.63030	0.60730
H	-6.68000	-1.70180	0.24950
H	-5.33010	-0.82820	-0.47280
H	-5.42850	-1.37200	2.52390
H	-6.21930	0.02530	1.80450
C	2.38070	-0.07460	-0.42240
C	3.38940	0.21090	-1.35160
C	2.74990	-0.67150	0.78300
C	4.71610	-0.08650	-1.07210

H	3.12410	0.67220	-2.29980
C	4.08390	-0.97080	1.05760
H	1.98480	-0.89490	1.52250
C	5.09660	-0.68490	0.13890
H	5.47040	0.14710	-1.81900
H	4.32330	-1.42980	2.01060
C	6.57220	-0.99660	0.40020
C	7.38760	0.30620	0.33940
H	8.44690	0.09540	0.52330
H	7.30580	0.78830	-0.63960
H	7.04150	1.01590	1.09820
C	7.08500	-1.96820	-0.67650
H	6.99470	-1.54200	-1.68040
H	8.14190	-2.19860	-0.50200
H	6.51990	-2.90580	-0.65300
C	6.79170	-1.63900	1.77350
H	7.85820	-1.84120	1.91480
H	6.46520	-0.97880	2.58400
H	6.25550	-2.58950	1.86430
O	1.24140	2.51930	-1.41870
H	0.41270	4.05180	1.60710
H	2.04730	3.98330	-0.27060

[6.5]PCP 13 StrainViz fragment r12

Cartesian coordinates

C	0.95680	1.46420	2.00480
C	-0.38180	1.20630	1.71960
C	-0.78160	-0.00620	1.13300
C	0.21670	-0.93440	0.86420
C	1.56320	-0.72510	1.16300

C	1.94180	0.51570	1.72370
H	1.20080	2.43240	2.42280
H	-0.04990	-1.88100	0.40380
C	1.37090	-0.03960	-2.28650
C	1.05490	1.19270	-1.70690
C	2.08120	1.99530	-1.20180
C	3.39930	1.54720	-1.27530
C	3.73770	0.32690	-1.86280
C	2.69270	-0.45740	-2.36710
H	0.56490	-0.64990	-2.68300
H	1.86760	2.95860	-0.75250
H	4.19100	2.18070	-0.87910
H	2.91620	-1.40800	-2.84540
C	2.48010	-1.83320	0.79520
O	2.14130	-2.76950	0.09680
O	3.70340	-1.73090	1.32810
C	-0.61130	2.82530	-1.22440
O	-0.26400	1.52810	-1.69020
H	-0.12890	3.58260	-1.85740
H	-0.25510	2.96330	-0.19670
C	3.63050	2.06810	2.31760
O	3.25020	0.75740	1.94070
H	4.71960	2.06420	2.34440
H	3.28230	2.80270	1.58240
H	3.24170	2.32760	3.30810
C	-2.11980	2.94140	-1.28460
H	-2.43480	3.92420	-0.92430
H	-2.47540	2.81270	-2.31040
C	5.10260	-2.26910	-0.56940
C	5.64710	-0.83440	-0.65700

C	5.18580	-0.10560	-1.93150
C	4.68660	-2.65700	0.84100
H	5.51530	-2.56890	1.54710
H	4.28190	-3.67110	0.87740
H	4.22210	-2.38260	-1.20910
H	5.84660	-2.98840	-0.92920
H	6.74200	-0.84930	-0.61790
H	5.30090	-0.25570	0.20830
H	5.34160	-0.75740	-2.80030
H	5.81190	0.78050	-2.08540
C	-2.19460	-0.25740	0.76090
C	-3.24140	-0.08300	1.67800
C	-2.52000	-0.66700	-0.53480
C	-4.56150	-0.30200	1.30010
H	-3.01810	0.19670	2.70560
C	-3.84350	-0.89650	-0.90190
H	-1.72440	-0.77580	-1.26650
C	-4.89460	-0.71340	0.00300
H	-5.34410	-0.15960	2.04050
H	-4.04840	-1.21110	-1.91940
C	-6.36190	-0.94270	-0.36720
C	-7.14640	0.36520	-0.16670
H	-8.19970	0.21480	-0.42780
H	-7.10290	0.70680	0.87210
H	-6.74420	1.16010	-0.80360
C	-6.95120	-2.03500	0.54150
H	-6.90320	-1.75150	1.59740
H	-8.00280	-2.20740	0.28680
H	-6.40800	-2.97750	0.41730
C	-6.52700	-1.38610	-1.82430

H	-7.58950	-1.53690	-2.04010
H	-6.14730	-0.63160	-2.52130
H	-6.00810	-2.33050	-2.01970
O	-1.25610	2.20730	1.99540
H	-2.13990	1.95820	1.67520
H	-2.58690	2.16990	-0.66480

[6.5]PCP 13 StrainViz fragment r13

Cartesian coordinates

C	1.12870	-0.35170	2.62770
C	-0.20830	-0.36300	2.25180
C	-0.61450	-0.84240	1.00280
C	0.37920	-1.33780	0.15900
C	1.72700	-1.35460	0.51070
C	2.11910	-0.82690	1.76030
H	1.39650	0.05440	3.59500
H	0.12010	-1.74560	-0.81260
C	1.35900	1.54760	-1.84640
C	0.91350	1.98390	-0.59420
C	1.83970	2.14460	0.43990
C	3.19270	1.91410	0.19200
C	3.66290	1.52600	-1.06340
C	2.71160	1.32970	-2.07250
H	0.62910	1.41760	-2.63950
H	1.52270	2.44340	1.43240
H	3.90640	2.06140	1.00100
H	3.03810	1.02680	-3.06480
C	2.64730	-1.90700	-0.52530
O	2.30130	-2.11670	-1.67050
O	3.87530	-2.17630	-0.07380

C	-0.88560	2.88600	0.69060
O	-0.41760	2.25250	-0.49300
H	-0.16880	3.65350	1.00730
H	-0.97080	2.13870	1.49270
C	3.81780	-0.09380	3.24260
O	3.43400	-0.77130	2.06030
H	4.90730	-0.09050	3.24110
H	3.44660	0.93770	3.24350
H	3.45570	-0.61260	4.13660
C	-2.22730	3.53550	0.39850
C	-3.29190	2.55470	-0.07970
H	-2.55360	4.03480	1.31840
H	-2.07430	4.32020	-0.35140
H	-4.24900	3.06380	-0.23020
H	-2.99580	2.09420	-1.02480
C	5.21690	-1.15930	-1.80640
C	5.66130	-0.03560	-0.85720
C	5.14650	1.34350	-1.30170
C	4.87340	-2.44640	-1.07140
H	5.72420	-2.83070	-0.50480
H	4.50950	-3.21850	-1.75320
H	4.32560	-0.85300	-2.36310
H	5.99430	-1.37180	-2.54850
H	6.75470	-0.01620	-0.78880
H	5.27710	-0.23210	0.15140
H	5.37150	1.49130	-2.36540
H	5.69000	2.12200	-0.75400
C	-2.03650	-0.83800	0.58880
C	-3.06840	-0.99160	1.52500
C	-2.39530	-0.68790	-0.75250

C	-4.39890	-0.97800	1.12920
H	-2.82760	-1.14510	2.57350
C	-3.73130	-0.69060	-1.14620
H	-1.62210	-0.53110	-1.49970
C	-4.76490	-0.82400	-0.21610
H	-5.16820	-1.10010	1.88750
H	-3.95660	-0.56090	-2.19930
C	-6.24500	-0.79850	-0.60280
C	-6.93640	0.36600	0.12650
H	-7.99810	0.40230	-0.14250
H	-6.86790	0.25870	1.21350
H	-6.47760	1.32130	-0.15020
C	-6.90630	-2.12400	-0.18890
H	-6.83290	-2.29160	0.89000
H	-7.96870	-2.11410	-0.45680
H	-6.43120	-2.96950	-0.69740
C	-6.44350	-0.60820	-2.10970
H	-7.51410	-0.59360	-2.33770
H	-6.01350	0.33810	-2.45500
H	-5.98970	-1.42390	-2.68250
H	-0.94560	0.03770	2.94260
H	-3.44310	1.74560	0.64430

Fully Optimized StrainViz Fragments of [6.3]PCP 28

Parent

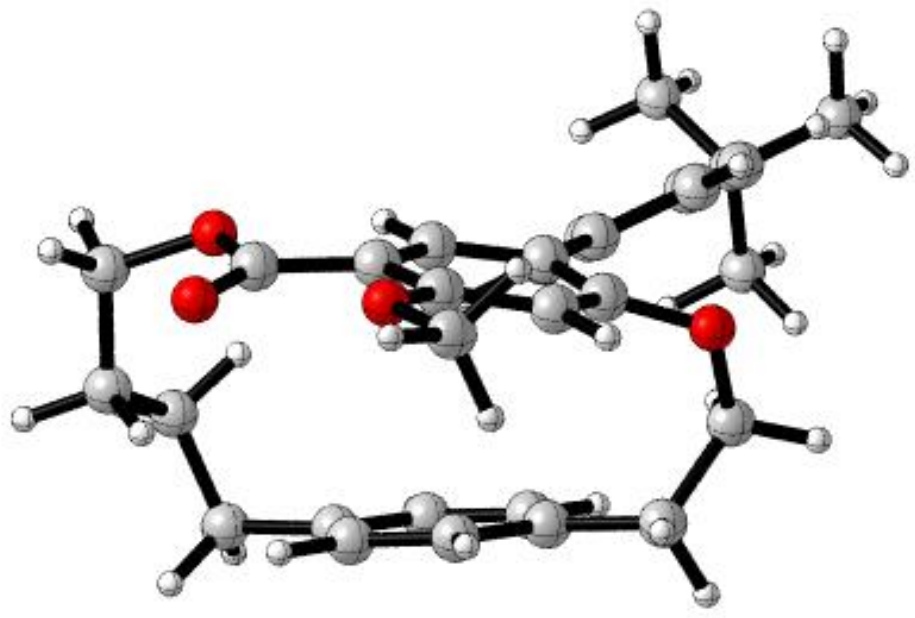

Fragment 1 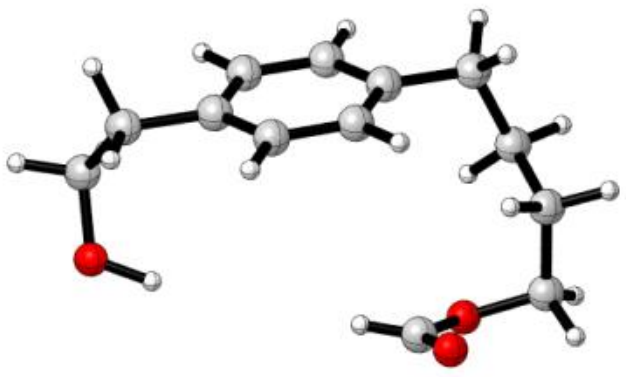	Fragment 2 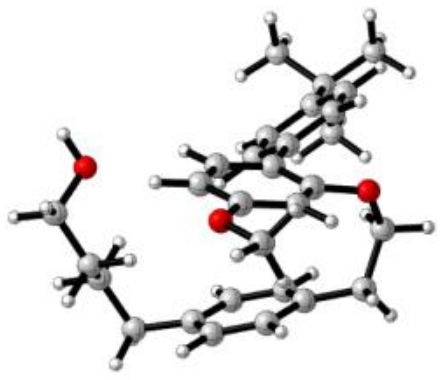	Fragment 3 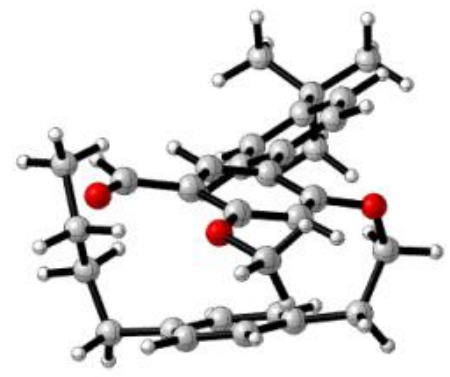
Fragment 4 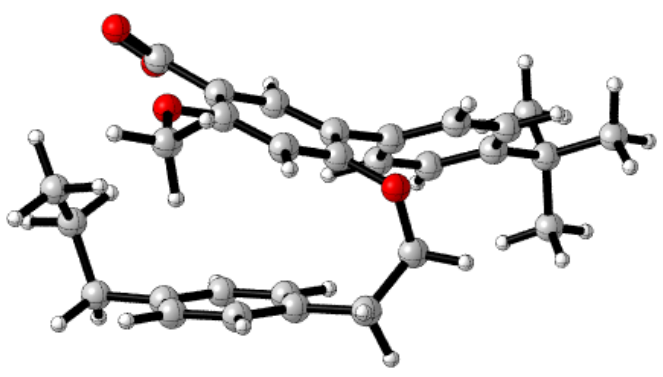	Fragment 5 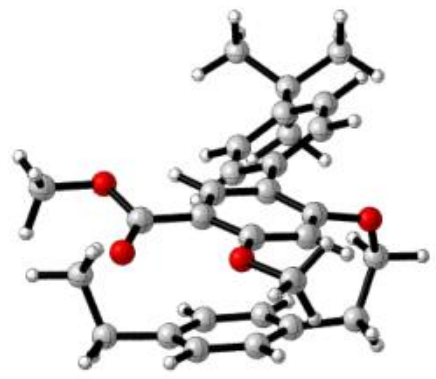	Fragment 6 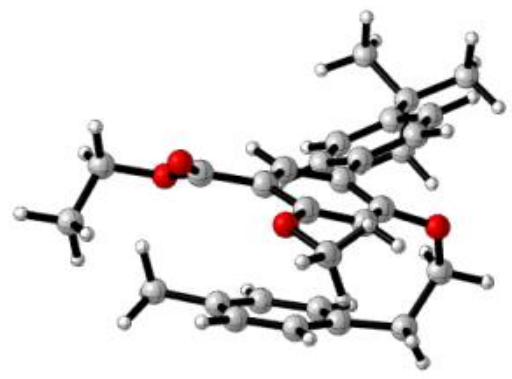
Fragment 7 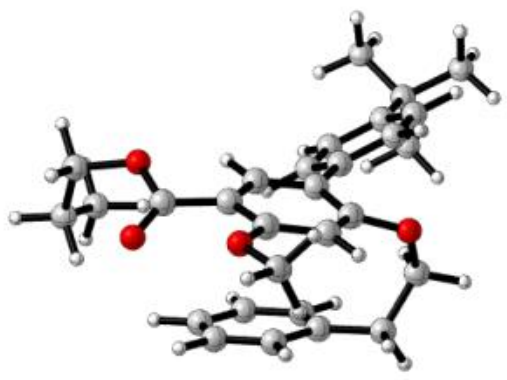	Fragment 8 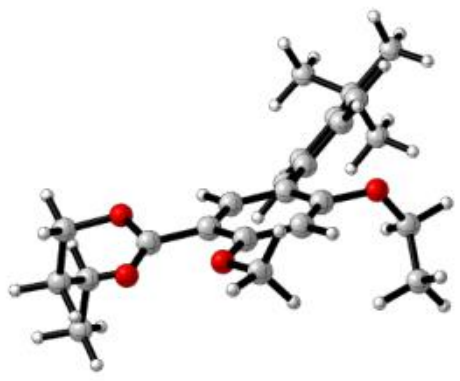	Fragment 9 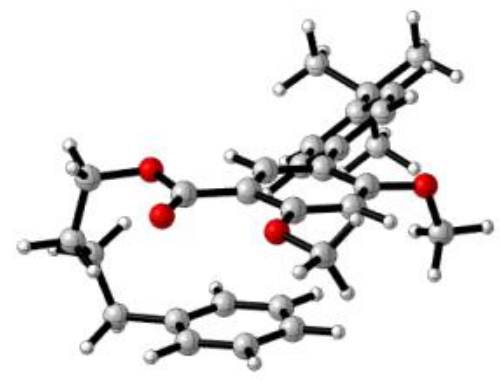
Fragment 10 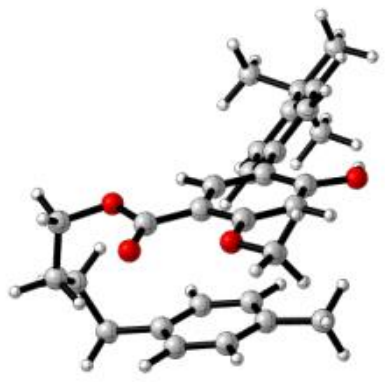	Fragment 11 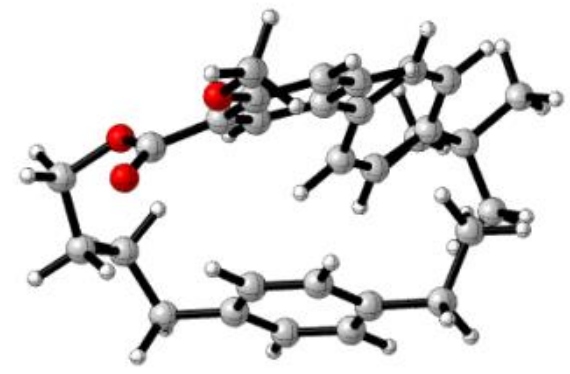	

[6.3]PCP 28 StrainViz fragment r1

Cartesian coordinates

C	2.10910	-0.65596	2.13651
C	0.76045	-0.92631	2.37607
C	-0.11042	0.16080	2.49778

C	0.35085	1.46488	2.35817
C	1.70029	1.73928	2.10745
C	2.57402	0.65304	2.00909
H	2.80975	-1.48291	2.04314
H	-1.16592	-0.01053	2.69946
H	-0.35000	2.29211	2.45274
H	3.63440	0.82437	1.83815
C	3.16285	0.65458	-1.63511
O	4.36630	0.55401	-1.54963
O	2.55497	1.82540	-1.93151
C	0.24834	-2.34460	2.53893
C	3.51444	3.26499	-0.25515
C	2.18255	3.59076	0.43335
C	2.14982	3.17163	1.91856
C	3.36099	3.00003	-1.74627
H	4.32653	2.85970	-2.23557
H	2.81015	3.79893	-2.24760
H	4.22751	4.08337	-0.10436
H	3.96922	2.37374	0.19079
H	1.36383	3.07901	-0.08825
H	1.98804	4.66550	0.34689
H	1.46132	3.82514	2.46441
H	3.14311	3.32148	2.35960
O	-0.47543	-3.14519	0.29700
C	-0.90189	-2.74674	1.60888
H	-1.63851	-1.93882	1.53104
H	-1.40530	-3.63252	2.00088
H	-0.12397	-2.46956	3.56369
H	1.06906	-3.06059	2.41490
H	2.57959	-0.06127	-1.32383

H	0.12337	-2.43414	-0.23006

[6.3]PCP 28 StrainViz fragment r2

Cartesian coordinates

C	2.04370	-2.48340	-0.78160
C	0.70450	-2.16980	-0.54410
C	0.15340	-0.94430	-0.94360
C	1.02410	-0.02280	-1.54530
C	2.35850	-0.30970	-1.77800
C	2.87560	-1.55370	-1.40310
H	2.39980	-3.44900	-0.44320
H	0.64800	0.94780	-1.85580
C	2.38680	-0.69670	2.10350
C	1.04550	-1.02210	2.31150
C	0.13190	0.02480	2.46510
C	0.54430	1.35090	2.38370
C	1.88470	1.67870	2.15990
C	2.80130	0.63090	2.03680
H	3.11550	-1.49490	1.98130
H	-0.91900	-0.19580	2.64100
H	-0.18790	2.14870	2.49370
H	3.85260	0.85810	1.87350
O	1.90060	2.98980	-2.32450
C	0.58490	-2.46220	2.39070
C	4.74270	-3.00630	-1.27210
O	4.19080	-1.76610	-1.67050
H	5.79120	-2.98120	-1.56710
H	4.67140	-3.14030	-0.18640
H	4.24170	-3.84250	-1.77250
C	3.25290	2.94530	-0.36360

C	2.21350	3.60180	0.54130
C	2.30300	3.12070	2.00010
C	3.16070	3.39830	-1.80980
H	3.97890	2.95120	-2.39200
H	3.26550	4.49210	-1.86950
H	4.26100	3.17280	0.00800
H	3.13540	1.85610	-0.33530
H	1.20910	3.39410	0.15210
H	2.34840	4.69110	0.51460
H	1.66330	3.75370	2.62530
H	3.33210	3.25300	2.35760
C	-1.27890	-0.60250	-0.76780
C	-1.65750	0.68180	-0.35430
C	-2.29500	-1.52440	-1.03650
C	-2.99660	1.02050	-0.20920
H	-0.88720	1.41270	-0.12210
C	-3.63660	-1.17950	-0.88620
H	-2.03130	-2.52660	-1.36030
C	-4.02020	0.09840	-0.46840
H	-3.24620	2.02590	0.12090
H	-4.38790	-1.92970	-1.10820
C	-5.48230	0.51340	-0.28630
C	-5.71430	0.93130	1.17580
H	-5.50140	0.09900	1.85500
H	-5.07610	1.77350	1.46010
H	-6.75730	1.23480	1.31990
C	-6.45180	-0.62440	-0.62150
H	-6.29410	-1.49270	0.02700
H	-7.48120	-0.28070	-0.47700
H	-6.34910	-0.94770	-1.66280

C	-5.79460	1.70270	-1.21020
H	-6.83870	2.01120	-1.08680
H	-5.15990	2.56540	-0.98560
H	-5.63800	1.42980	-2.25910
O	-0.03580	-3.14470	0.08580
C	-0.51620	-2.84510	1.39890
H	-1.28100	-2.06140	1.35260
H	-0.99830	-3.77140	1.71960
H	0.18490	-2.65420	3.39470
H	1.43460	-3.14130	2.25490
H	1.84470	3.27490	-3.24730
H	3.00480	0.41640	-2.26070

[6.3]PCP 28 StrainViz fragment r3

Cartesian coordinates

C	2.60640	-2.18720	-0.37420
C	1.21940	-2.09120	-0.25830
C	0.50520	-1.01770	-0.81820
C	1.26290	-0.04170	-1.46080
C	2.65450	-0.08260	-1.56220
C	3.34170	-1.18830	-1.01210
H	3.08160	-3.04720	0.08040
H	0.74190	0.79360	-1.92680
C	2.11580	0.07520	2.12690
C	0.95230	-0.60300	2.49200
C	-0.20840	0.14760	2.70670
C	-0.20820	1.52740	2.54260
C	0.95010	2.20610	2.15010
C	2.11210	1.45750	1.94970
H	3.03150	-0.48640	1.95770

H	-1.12630	-0.35590	3.00450
H	-1.12520	2.08910	2.71060
H	3.02590	1.96230	1.64350
C	3.30490	1.04340	-2.25520
O	4.49170	1.20450	-2.44230
C	0.91220	-2.10700	2.65460
C	5.37550	-2.30770	-0.55140
O	4.68100	-1.21170	-1.12490
H	6.43050	-2.12740	-0.75140
H	5.20830	-2.35480	0.53020
H	5.06910	-3.25260	-1.01190
C	0.94580	3.54880	-0.64810
C	0.18440	4.03260	0.58440
C	0.90400	3.69540	1.90040
C	0.13180	3.68750	-1.93200
H	0.70250	3.37340	-2.81220
H	-0.17710	4.72630	-2.09060
H	1.88520	4.11060	-0.73490
H	1.23240	2.49960	-0.49940
H	-0.81570	3.57450	0.60010
H	0.02880	5.11640	0.51690
H	0.38630	4.18960	2.73060
H	1.92300	4.09980	1.87080
C	-0.96790	-0.87870	-0.74260
C	-1.53230	0.37020	-0.45270
C	-1.83200	-1.95070	-0.97690
C	-2.90940	0.53670	-0.40350
H	-0.87690	1.20980	-0.23030
C	-3.21400	-1.77800	-0.92150
H	-1.41980	-2.93010	-1.20060

C	-3.78490	-0.53390	-0.63570
H	-3.30640	1.52100	-0.16730
H	-3.84710	-2.63770	-1.11230
C	-5.29690	-0.30740	-0.56190
C	-5.66800	0.19050	0.84550
H	-5.38680	-0.54750	1.60420
H	-5.16640	1.13300	1.08550
H	-6.74880	0.35760	0.91210
C	-6.08740	-1.58900	-0.84400
H	-5.85710	-2.37320	-0.11510
H	-7.15950	-1.37690	-0.78040
H	-5.88130	-1.97730	-1.84700
C	-5.70460	0.75190	-1.60000
H	-6.78540	0.92590	-1.55450
H	-5.20200	1.70720	-1.42030
H	-5.45190	0.41980	-2.61240
O	0.60650	-3.11780	0.40500
C	-0.01380	-2.82170	1.66460
H	-0.92850	-2.23940	1.50610
H	-0.29240	-3.80460	2.04930
H	0.54660	-2.34780	3.66030
H	1.91830	-2.53420	2.57340
H	-0.77700	3.07530	-1.88560
H	2.57660	1.79900	-2.62160

[6.3]PCP 28 StrainViz fragment r4

Cartesian coordinates

C	1.58570	-0.71770	-1.97780
C	0.24010	-0.74690	-1.62520
C	-0.37410	0.34690	-0.99250

C	0.43870	1.44030	-0.69670
C	1.79240	1.49680	-1.03200
C	2.37640	0.39420	-1.69260
H	1.99670	-1.60580	-2.44040
H	-0.00560	2.30260	-0.21020
C	2.45500	-2.74270	0.63160
C	1.06950	-2.59430	0.70670
C	0.55890	-1.63800	1.59010
C	1.40890	-0.84680	2.35390
C	2.79780	-0.99230	2.27860
C	3.30690	-1.95720	1.40660
H	2.87510	-3.48080	-0.04870
H	-0.51650	-1.49830	1.67950
H	0.98850	-0.10420	3.02930
H	4.38370	-2.09470	1.33240
C	2.56970	2.72510	-0.73890
O	3.57280	3.09590	-1.30420
O	2.01570	3.44560	0.26200
C	0.14980	-3.44910	-0.14090
C	4.32070	-0.73610	-2.43190
O	3.69060	0.45290	-1.98330
H	5.37990	-0.49660	-2.51410
H	4.17760	-1.54620	-1.70750
H	3.93790	-1.04330	-3.41060
C	4.80940	1.13770	1.17100
C	3.96100	1.25730	2.43310
C	3.69700	-0.10180	3.10130
H	5.77190	0.66340	1.39670
H	4.30760	0.53310	0.40840
H	3.00090	1.73130	2.19070

H	4.46420	1.91080	3.15540
H	3.23680	0.06290	4.08240
H	4.65630	-0.60430	3.27760
C	-1.81290	0.37050	-0.63410
C	-2.22010	0.81910	0.62880
C	-2.80010	-0.03570	-1.53580
C	-3.56620	0.85990	0.96980
H	-1.46880	1.12320	1.35410
C	-4.14780	0.00660	-1.18640
H	-2.50920	-0.39130	-2.51960
C	-4.56300	0.45460	0.07160
H	-3.84190	1.20950	1.96130
H	-4.87930	-0.31400	-1.92010
C	-6.03360	0.51300	0.49210
C	-6.24700	-0.38380	1.72350
H	-5.99200	-1.42380	1.49460
H	-5.63180	-0.06090	2.56910
H	-7.29620	-0.34770	2.03720
C	-6.97170	0.03520	-0.62090
H	-6.77150	-1.00520	-0.89800
H	-8.00830	0.09610	-0.27440
H	-6.88120	0.65600	-1.51850
C	-6.40410	1.96280	0.84840
H	-7.45500	2.01770	1.15330
H	-5.79410	2.34410	1.67310
H	-6.26090	2.62240	-0.01380
O	-0.44740	-1.88500	-1.95420
C	-0.94830	-2.68460	-0.87610
H	-1.53050	-2.06670	-0.18560
H	-1.63280	-3.38840	-1.35450

H	-0.35330	-4.18950	0.49550
H	0.73770	-4.01210	-0.87420
H	5.00880	2.11760	0.72830
H	2.55710	4.24950	0.35570

[6.3]PCP 28 StrainViz fragment r5

Cartesian coordinates

C	2.02330	-2.20350	-0.88700
C	0.66770	-1.96390	-0.68280
C	0.11860	-0.67890	-0.83440
C	1.01670	0.35480	-1.09630
C	2.39180	0.15890	-1.23810
C	2.90240	-1.15430	-1.15800
H	2.37010	-3.22200	-0.76740
H	0.63180	1.36280	-1.20980
C	2.06230	-0.98700	2.13760
C	0.74110	-1.39810	2.31670
C	-0.20660	-0.43270	2.67130
C	0.15250	0.90420	2.80470
C	1.46760	1.32270	2.57980
C	2.41860	0.35390	2.25470
H	2.81780	-1.72180	1.87030
H	-1.24180	-0.72730	2.83250
H	-0.60350	1.63980	3.07430
H	3.44860	0.65400	2.07320
C	3.28520	1.33000	-1.43320
O	4.47880	1.36260	-1.23690
O	2.59750	2.41660	-1.84230
C	0.32180	-2.83740	2.10510
C	4.72740	-2.65980	-1.28140

O	4.22480	-1.33590	-1.34060
H	5.79530	-2.58030	-1.47900
H	4.57040	-3.09770	-0.28950
H	4.25840	-3.29500	-2.04020
C	1.36990	3.51140	1.35000
C	1.82650	2.78820	2.62230
C	3.37020	3.61040	-1.96490
H	4.15420	3.48080	-2.71360
H	2.67000	4.38460	-2.27350
H	0.27950	3.46920	1.25270
H	1.66870	4.56420	1.36090
H	1.36310	3.26140	3.49590
H	2.91030	2.89690	2.73710
C	-1.33480	-0.39850	-0.74340
C	-1.79970	0.76170	-0.11120
C	-2.28150	-1.25250	-1.31690
C	-3.15710	1.04900	-0.05510
H	-1.08520	1.43140	0.35980
C	-3.64220	-0.96050	-1.25330
H	-1.95030	-2.15820	-1.81580
C	-4.11300	0.19490	-0.62230
H	-3.47580	1.95730	0.44990
H	-4.33800	-1.65270	-1.71520
C	-5.59910	0.54940	-0.53070
C	-6.00900	0.64680	0.94880
H	-5.84610	-0.30810	1.45960
H	-5.43690	1.41630	1.47610
H	-7.07140	0.90280	1.02860
C	-6.48600	-0.50000	-1.20850
H	-6.37370	-1.48450	-0.74220

H	-7.53590	-0.20370	-1.11700
H	-6.25580	-0.59390	-2.27500
C	-5.84270	1.90470	-1.21610
H	-6.90360	2.17180	-1.15450
H	-5.26600	2.70540	-0.74310
H	-5.55980	1.86070	-2.27300
O	-0.08730	-3.05380	-0.33790
C	-0.68370	-3.03900	0.96660
H	-1.47070	-2.27780	1.01250
H	-1.15120	-4.02220	1.04930
H	-0.15760	-3.21610	3.01650
H	1.19810	-3.46940	1.92050
H	3.82770	3.86800	-1.00690
H	1.80030	3.03750	0.46160

[6.3]PCP 28 StrainViz fragment r6

Cartesian coordinates

C	1.73770	-2.37870	-0.79800
C	0.40600	-2.03620	-0.58450
C	-0.05800	-0.72320	-0.77910
C	0.90970	0.23930	-1.06460
C	2.26080	-0.06310	-1.23370
C	2.67930	-1.40460	-1.13170
H	2.01700	-3.41130	-0.63160
H	0.60100	1.27240	-1.18720
C	1.90430	-0.93190	2.13910
C	0.56820	-1.27520	2.36170
C	-0.32900	-0.25030	2.66840
C	0.08790	1.07780	2.70630
C	1.41140	1.42620	2.43000

C	2.31720	0.39570	2.16270
H	2.62410	-1.71430	1.91030
H	-1.37300	-0.48690	2.86460
H	-0.63290	1.85920	2.93770
H	3.35660	0.63990	1.95160
C	3.23260	1.02920	-1.50100
O	4.27130	0.92540	-2.11370
O	2.81090	2.19420	-0.96830
C	0.08370	-2.70540	2.24450
C	4.41780	-3.01410	-1.13050
O	3.99050	-1.67310	-1.29580
H	5.49090	-3.00870	-1.31570
H	4.22290	-3.36870	-0.11220
H	3.92450	-3.67730	-1.84880
C	4.82560	3.32870	-0.20170
C	1.84820	2.86780	2.38330
C	3.66460	3.33100	-1.17900
H	4.01580	3.32450	-2.21320
H	3.01390	4.19360	-1.02470
H	5.42560	4.23320	-0.33540
H	5.46410	2.45900	-0.37070
H	1.11860	3.52240	2.86750
H	2.81510	3.00800	2.87690
C	-1.49140	-0.35010	-0.70190
C	-1.88870	0.87110	-0.14190
C	-2.48740	-1.18020	-1.22560
C	-3.22760	1.23750	-0.10470
H	-1.13880	1.52810	0.28950
C	-3.82920	-0.80840	-1.18120
H	-2.21000	-2.13080	-1.67040

C	-4.23230	0.40690	-0.62030
H	-3.49310	2.19100	0.34480
H	-4.56390	-1.48620	-1.60230
C	-5.69590	0.84900	-0.55000
C	-6.09440	1.05700	0.92110
H	-5.98320	0.12660	1.48790
H	-5.47820	1.82340	1.40130
H	-7.14060	1.37630	0.98590
C	-6.64230	-0.18750	-1.16390
H	-6.58310	-1.14740	-0.64000
H	-7.67370	0.17190	-1.08980
H	-6.42160	-0.35740	-2.22300
C	-5.86590	2.17270	-1.31470
H	-6.91000	2.50180	-1.26900
H	-5.24390	2.96660	-0.89000
H	-5.58940	2.05040	-2.36710
O	-0.41260	-3.05420	-0.16980
C	-0.96470	-2.92980	1.14860
H	-1.71410	-2.13020	1.16760
H	-1.47430	-3.88220	1.30770
H	-0.38280	-3.01130	3.18930
H	0.92750	-3.38360	2.07280
H	4.45850	3.30630	0.82830
H	1.96010	3.19480	1.34280

[6.3]PCP 28 StrainViz fragment r7

Cartesian coordinates

C	1.79460	-2.31650	-1.03990
C	0.45830	-2.01740	-0.78930
C	-0.02370	-0.69900	-0.83620

C	0.93130	0.30430	-0.99340
C	2.29330	0.04400	-1.14070
C	2.72860	-1.29500	-1.21390
H	2.08510	-3.35920	-1.01200
H	0.60830	1.33990	-0.99710
C	2.13240	-1.31510	2.05580
C	0.77150	-1.59140	2.20610
C	-0.07660	-0.55290	2.60520
C	0.41970	0.72980	2.82300
C	1.77750	0.99660	2.64770
C	2.63480	-0.03250	2.26630
H	2.80490	-2.11170	1.74630
H	-1.13950	-0.74520	2.73700
H	-0.25580	1.52410	3.12930
H	3.69200	0.16410	2.11300
C	3.25330	1.17640	-1.07830
O	4.40450	1.10470	-0.70990
O	2.66720	2.33840	-1.42890
C	0.22010	-2.98070	1.94990
C	4.47270	-2.86790	-1.51630
O	4.03900	-1.52140	-1.43100
H	5.54080	-2.82360	-1.72360
H	4.30480	-3.39720	-0.57190
H	3.96110	-3.39680	-2.32750
C	3.26810	3.90550	0.33030
C	1.81140	4.09440	0.74710
C	3.40690	3.53450	-1.13890
H	4.45140	3.38880	-1.42120
H	2.95830	4.29650	-1.78060
H	3.83620	4.82740	0.49880

H	3.74070	3.12350	0.93610
H	1.24980	3.16150	0.63110
H	1.32540	4.86220	0.13550
C	-1.45980	-0.35600	-0.70240
C	-1.86490	0.77050	0.02550
C	-2.45130	-1.12240	-1.32260
C	-3.20800	1.10930	0.12640
H	-1.11810	1.37330	0.53510
C	-3.79680	-0.77900	-1.21380
H	-2.16660	-2.00000	-1.89460
C	-4.20810	0.34310	-0.48800
H	-3.48030	1.98840	0.70480
H	-4.52820	-1.40350	-1.71520
C	-5.67590	0.75150	-0.34270
C	-6.06030	0.74580	1.14680
H	-5.93110	-0.25310	1.57650
H	-5.44840	1.44600	1.72370
H	-7.10960	1.03840	1.26540
C	-6.61670	-0.20120	-1.08720
H	-6.54190	-1.22390	-0.70280
H	-7.65120	0.13150	-0.95540
H	-6.40450	-0.21910	-2.16140
C	-5.87020	2.16700	-0.91220
H	-6.91770	2.47210	-0.81110
H	-5.25340	2.90190	-0.38570
H	-5.60380	2.19760	-1.97390
O	-0.35030	-3.07740	-0.47020
C	-0.86390	-3.07010	0.87010
H	-1.59230	-2.25960	0.98780
H	-1.39320	-4.02120	0.95340

H	-0.23210	-3.36070	2.87470
H	1.03330	-3.66840	1.69090
H	1.73820	4.40050	1.79480
H	2.16420	1.99780	2.81500

[6.3]PCP 28 StrainViz fragment r8

Cartesian coordinates

C	1.68970	-2.73800	-0.19430
C	0.36650	-2.30800	-0.13640
C	0.03110	-0.94960	-0.26900
C	1.08900	-0.05750	-0.44240
C	2.42670	-0.45330	-0.48700
C	2.73370	-1.82680	-0.37090
H	1.87080	-3.80010	-0.08930
H	0.86660	0.99660	-0.56940
C	3.49180	0.57580	-0.63770
O	4.66230	0.43170	-0.36640
O	2.98880	1.73360	-1.10930
C	-0.31700	-3.90410	2.36360
C	4.32430	-3.58330	-0.37360
O	4.02500	-2.19940	-0.44980
H	5.40570	-3.65760	-0.47640
H	4.01810	-4.00030	0.59160
H	3.83820	-4.13740	-1.18340
C	4.07480	3.48420	0.20210
C	2.75640	3.93660	0.82800
C	2.96490	4.61130	2.18120
C	3.89640	2.84580	-1.16660
H	4.84970	2.50630	-1.57730
H	3.42640	3.54150	-1.86560

H	4.74580	4.34480	0.08480
H	4.58170	2.77380	0.86550
H	2.09570	3.06910	0.94430
H	2.24760	4.62610	0.14180
H	2.01480	4.92390	2.62360
H	3.59880	5.49880	2.08160
C	-1.36800	-0.45320	-0.25130
C	-1.69100	0.72580	0.43300
C	-2.39460	-1.11560	-0.93060
C	-2.98880	1.22010	0.43080
H	-0.91740	1.25310	0.98540
C	-3.69450	-0.61460	-0.92710
H	-2.17370	-2.03110	-1.46960
C	-4.02350	0.56270	-0.24880
H	-3.19760	2.13560	0.97810
H	-4.45590	-1.16060	-1.47370
C	-5.43940	1.14310	-0.22180
C	-5.93040	1.22100	1.23390
H	-5.95330	0.22530	1.68910
H	-5.28390	1.85910	1.84420
H	-6.94310	1.63790	1.26690
C	-6.42910	0.28810	-1.01960
H	-6.50340	-0.72650	-0.61420
H	-7.42400	0.74210	-0.97120
H	-6.14290	0.21920	-2.07440
C	-5.42160	2.55640	-0.82850
H	-6.43000	2.98490	-0.81220
H	-4.76180	3.22700	-0.26950
H	-5.07660	2.52790	-1.86730
O	-0.59460	-3.26900	0.02380

C	-1.22630	-3.29140	1.31410
H	-1.52900	-2.27640	1.59410
H	-2.12930	-3.88930	1.17300
H	-0.83740	-3.95770	3.32400
H	-0.02070	-4.91480	2.06980
H	3.45570	3.92960	2.88390
H	0.58610	-3.30110	2.49760

[6.3]PCP 28 StrainViz fragment r9

Cartesian coordinates

C	1.24320	-2.87940	-0.33850
C	-0.07860	-2.45580	-0.25050
C	-0.45640	-1.14850	-0.58910
C	0.55990	-0.29660	-1.02110
C	1.89670	-0.68290	-1.11740
C	2.24740	-2.00430	-0.76000
H	1.45920	-3.89990	-0.04850
H	0.30350	0.71970	-1.29830
C	3.25300	-0.41340	2.10780
C	1.94300	-0.82990	2.33040
C	0.90790	0.10240	2.28490
C	1.18930	1.44120	2.02610
C	2.50180	1.87790	1.81270
C	3.52880	0.93010	1.85590
H	4.06550	-1.13400	2.13180
H	-0.12010	-0.21230	2.44800
H	0.38040	2.16960	2.00350
H	4.55760	1.24810	1.70480
C	2.91800	0.31160	-1.54330
O	4.11680	0.14560	-1.56070

O	2.34230	1.47250	-1.92480
C	3.89390	-3.66680	-0.39710
O	3.54170	-2.36530	-0.83280
H	4.97510	-3.73390	-0.50920
H	3.62280	-3.81490	0.65430
H	3.41350	-4.43580	-1.01110
C	3.72200	3.14420	-0.84880
C	2.57780	3.67370	0.03030
C	2.76790	3.33750	1.52010
C	3.23530	2.56920	-2.16960
H	4.06600	2.22580	-2.78970
H	2.63650	3.29130	-2.72930
H	4.44770	3.93850	-1.05540
H	4.26770	2.34970	-0.32970
H	1.62620	3.23650	-0.29740
H	2.48600	4.75830	-0.09150
H	2.08470	3.95080	2.11800
H	3.78710	3.60290	1.82600
C	-1.85030	-0.65460	-0.47270
C	-2.09740	0.62510	0.04230
C	-2.94910	-1.41980	-0.87350
C	-3.39220	1.11590	0.14660
H	-1.26050	1.23380	0.37640
C	-4.24570	-0.92140	-0.76430
H	-2.78890	-2.41440	-1.27620
C	-4.49940	0.35490	-0.25330
H	-3.54010	2.11190	0.55600
H	-5.06590	-1.54990	-1.09380
C	-5.90950	0.93440	-0.11630
C	-6.18570	1.25740	1.36210

H	-6.11950	0.35270	1.97540
H	-5.47200	1.98780	1.75540
H	-7.19210	1.67600	1.47380
C	-6.98480	-0.04090	-0.60570
H	-6.97600	-0.97260	-0.03000
H	-7.97220	0.41660	-0.48770
H	-6.85360	-0.28730	-1.66460
C	-6.01270	2.22530	-0.94630
H	-7.01800	2.65140	-0.85480
H	-5.29540	2.98000	-0.60940
H	-5.82020	2.02180	-2.00490
O	-1.00030	-3.37330	0.18790
C	-1.38290	-3.18320	1.54720
H	-1.83330	-2.19600	1.69640
H	-2.11700	-3.95640	1.77520
H	1.72980	-1.87600	2.53000
H	-0.51350	-3.29510	2.20600

[6.3]PCP 28 StrainViz fragment r10

Cartesian coordinates

C	1.24560	-2.87390	-0.39630
C	-0.08060	-2.46660	-0.26230
C	-0.48510	-1.16900	-0.61560
C	0.50660	-0.29470	-1.05340
C	1.84740	-0.65670	-1.17400
C	2.21880	-1.98200	-0.84050
H	1.48910	-3.88650	-0.10190
H	0.22420	0.71940	-1.31470
C	3.05630	-0.31370	2.08790
C	1.76080	-0.74210	2.38300

C	0.73090	0.20130	2.31780
C	0.99600	1.52820	1.99610
C	2.29830	1.96670	1.73000
C	3.32350	1.01860	1.77410
H	3.87380	-1.03090	2.11500
H	-0.29150	-0.10860	2.52710
H	0.18050	2.24950	1.97060
H	4.34840	1.32420	1.57750
C	2.84580	0.35490	-1.59780
O	4.05020	0.23250	-1.56740
O	2.24550	1.48440	-2.03670
C	1.48440	-2.15930	2.81770
C	3.88350	-3.65640	-0.64000
O	3.51280	-2.33020	-0.97210
H	4.95500	-3.71930	-0.82420
H	3.68030	-3.87140	0.41500
H	3.35890	-4.38410	-1.26830
C	3.54700	3.21530	-0.95260
C	2.36800	3.71770	-0.10470
C	2.54800	3.42020	1.39450
C	3.11010	2.60450	-2.27460
H	3.96690	2.27850	-2.86850
H	2.50420	3.29920	-2.86090
H	4.24940	4.03130	-1.15550
H	4.10760	2.44840	-0.40950
H	1.43970	3.23690	-0.43800
H	2.23670	4.79510	-0.25200
H	1.85190	4.03960	1.97090
H	3.56050	3.70880	1.70220
C	-1.89340	-0.72370	-0.48080

C	-2.19520	0.51150	0.11010
C	-2.96010	-1.49700	-0.95190
C	-3.50800	0.94420	0.22550
H	-1.38320	1.12850	0.48580
C	-4.27880	-1.05960	-0.82370
H	-2.76210	-2.43870	-1.45960
C	-4.58390	0.16760	-0.23230
H	-3.69880	1.90760	0.69100
H	-5.06950	-1.69360	-1.20960
C	-6.01550	0.68410	-0.07270
C	-6.31460	0.89630	1.42120
H	-6.22080	-0.04470	1.97320
H	-5.63130	1.62370	1.87030
H	-7.33640	1.27020	1.54970
C	-7.04820	-0.29610	-0.63790
H	-7.00820	-1.26350	-0.12610
H	-8.05330	0.11460	-0.49960
H	-6.89970	-0.46460	-1.70970
C	-6.15970	2.02300	-0.81620
H	-7.18150	2.40310	-0.70820
H	-5.47600	2.78060	-0.42100
H	-5.94980	1.89840	-1.88360
O	-0.93330	-3.38420	0.25750
H	1.46720	-2.23220	3.91140
H	2.25670	-2.84220	2.45210
H	0.51770	-2.51230	2.44600
H	-1.79500	-2.96460	0.42080

[6.3]PCP 28 StrainViz fragment r11

Cartesian coordinates

C	2.04950	-3.20390	0.26080
C	0.71690	-2.87990	0.02330
C	0.34770	-1.66680	-0.56510
C	1.37460	-0.78660	-0.91110
C	2.71350	-1.06600	-0.65040
C	3.06490	-2.29750	-0.05920
H	2.28670	-4.15700	0.71660
H	1.13300	0.16080	-1.38000
C	0.59390	0.69310	2.15000
C	-0.71700	0.90160	2.59010
C	-1.34920	2.08630	2.21020
C	-0.69130	3.03410	1.42820
C	0.62650	2.83870	1.00420
C	1.25560	1.64440	1.37880
H	1.10790	-0.22990	2.41380
H	-2.37400	2.26900	2.52710
H	-1.20970	3.94740	1.14520
H	2.27870	1.44370	1.07000
C	3.72020	0.00800	-0.88420
O	4.72060	0.18530	-0.22700
O	3.34190	0.82560	-1.88500
C	-1.43150	-0.13930	3.41950
C	4.74060	-3.79080	0.70010
O	4.37590	-2.53920	0.14390
H	5.82760	-3.78120	0.76510
H	4.31500	-3.91690	1.70120
H	4.41790	-4.61730	0.05780
C	3.50690	3.04020	-0.91740
C	2.01340	3.33890	-1.09920
C	1.32250	3.89130	0.16350

C	4.03770	2.08200	-1.97400
H	5.10940	1.90850	-1.86240
H	3.82810	2.44180	-2.98370
H	4.08240	3.97210	-0.96570
H	3.70880	2.60870	0.06890
H	1.49320	2.42660	-1.41430
H	1.90360	4.05410	-1.92220
H	0.57730	4.64010	-0.12280
H	2.06430	4.42310	0.77520
C	-1.07230	-1.28570	-0.74750
C	-1.46810	0.05300	-0.62410
C	-2.06560	-2.23980	-0.97800
C	-2.80590	0.41000	-0.71220
H	-0.72960	0.82040	-0.40480
C	-3.40850	-1.87610	-1.05880
H	-1.79230	-3.28410	-1.10420
C	-3.81160	-0.54460	-0.91990
H	-3.06850	1.45740	-0.58460
H	-4.14320	-2.65430	-1.23550
C	-5.27690	-0.10660	-0.95970
C	-5.64490	0.52470	0.39440
H	-5.51320	-0.19920	1.20600
H	-5.02100	1.39710	0.61370
H	-6.69150	0.84940	0.38850
C	-6.22480	-1.28170	-1.21860
H	-6.15330	-2.03860	-0.43040
H	-7.25750	-0.91920	-1.24160
H	-6.01600	-1.76280	-2.18010
C	-5.47550	0.93120	-2.07700
H	-6.52190	1.25460	-2.10780

H	-4.85420	1.81810	-1.91960
H	-5.21870	0.50470	-3.05230
C	-1.94210	-1.31330	2.57720
H	-2.64750	-0.97060	1.81240
H	-2.44160	-2.05540	3.20790
H	-2.27190	0.33000	3.94300
H	-0.75040	-0.51820	4.19070
H	-1.11050	-1.80370	2.06010
H	-0.05110	-3.58480	0.33010

Fully Optimized StrainViz Fragments of [5.5]PCP 27

Parent

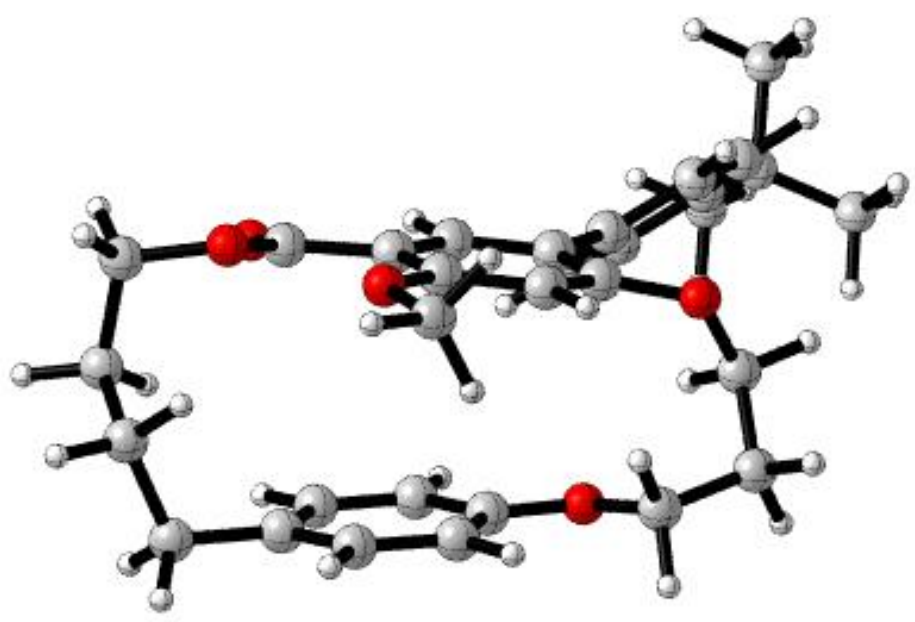

Fragment 1 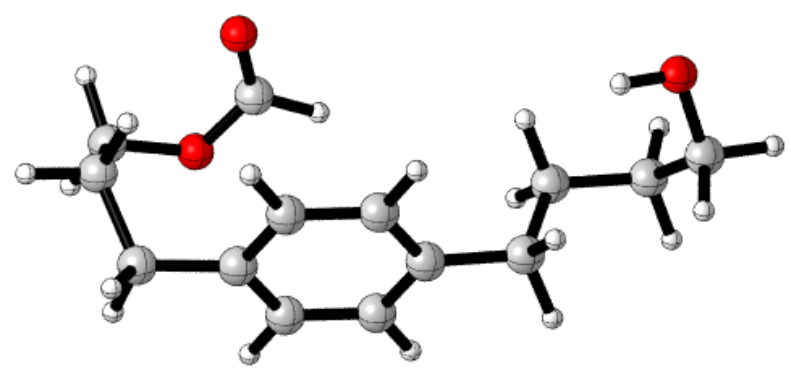	Fragment 2 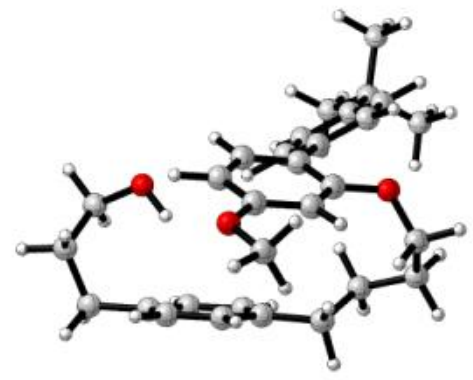	Fragment 3 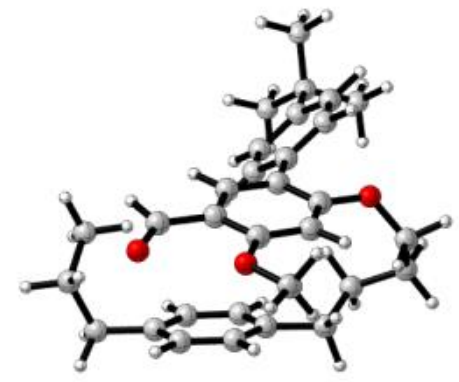
Fragment 4 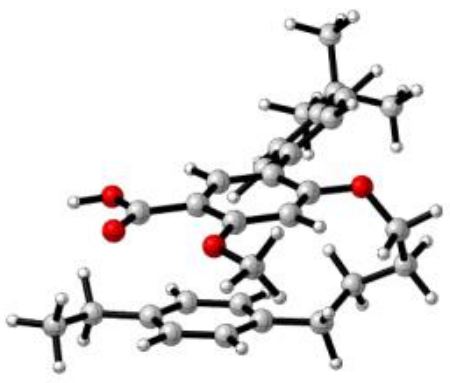	Fragment 5 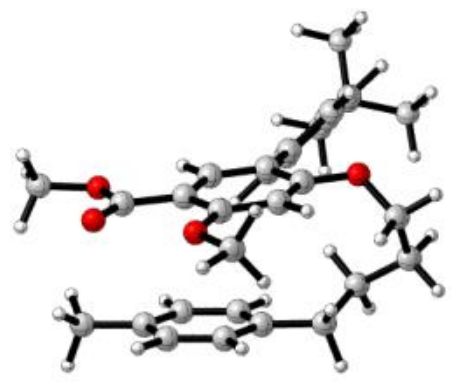	Fragment 6 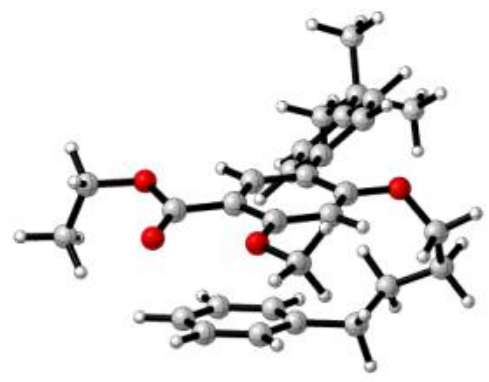
Fragment 7 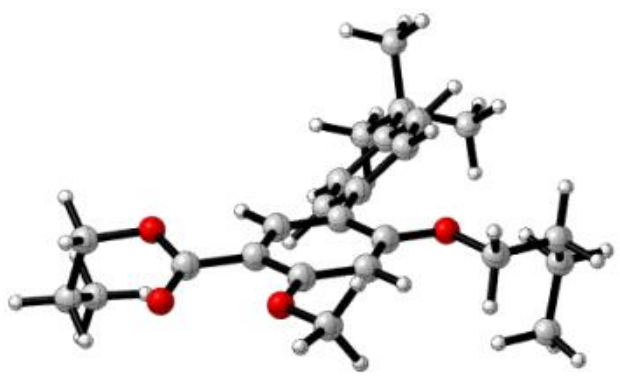	Fragment 8 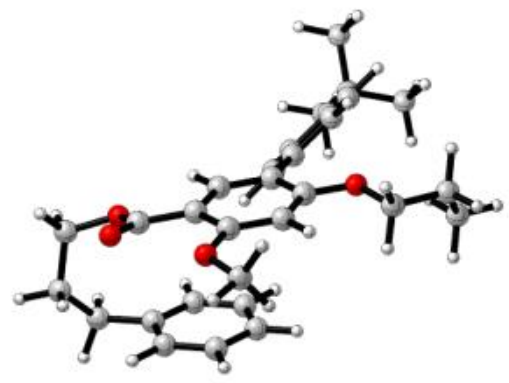	Fragment 9 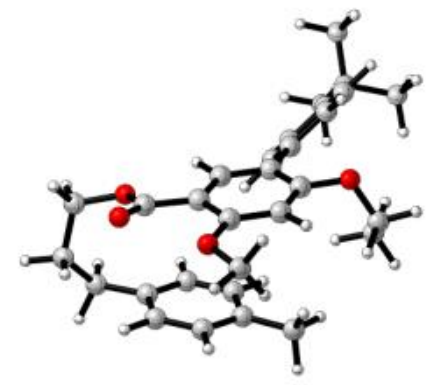
Fragment 10 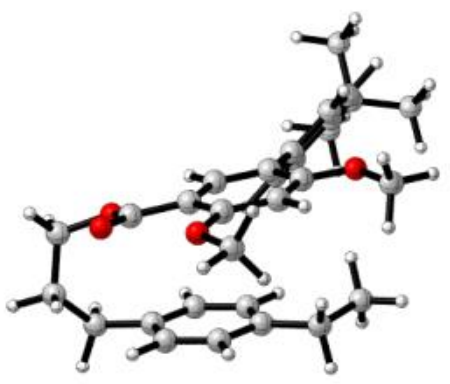	Fragment 11 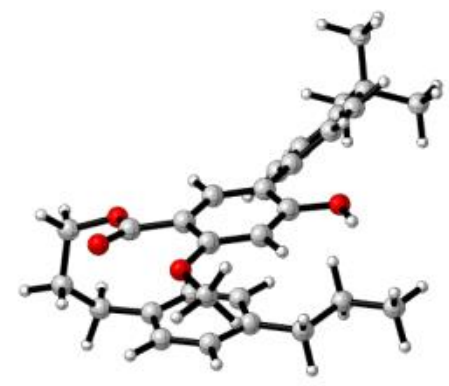	Fragment 12 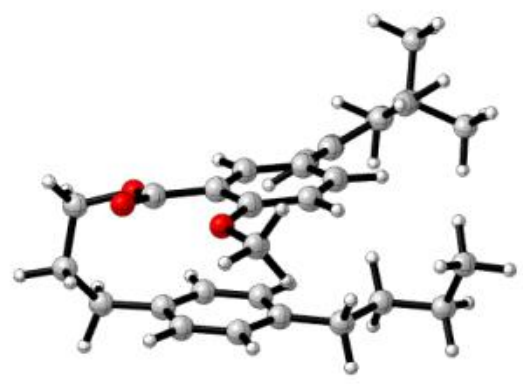

[5.5]PCP 27 StrainViz fragment r1

Cartesian coordinates

C	0.28380	-0.79160	1.24670
C	0.90050	-1.20100	0.06220
C	0.08730	-1.65500	-0.98010
C	-1.29730	-1.68500	-0.84590
C	-1.91590	-1.25950	0.33170
C	-1.10180	-0.82310	1.38060
H	0.89680	-0.44050	2.07420
H	0.54650	-1.98270	-1.91040
H	-1.91180	-2.02460	-1.67620

H	-1.55630	-0.49250	2.31200
C	-1.70720	1.75930	-0.51580
O	-1.85030	2.39870	0.49580
O	-2.67030	1.11190	-1.16770
C	-3.42390	-1.22870	0.46460
C	2.78160	0.24760	-0.76950
C	2.39410	-1.08830	-0.11640
H	-3.88010	-1.66780	-0.43080
H	2.44550	1.07280	-0.12690
H	2.22380	0.35260	-1.70760
C	4.27850	0.38830	-1.05480
C	5.15270	0.28390	0.19210
O	4.80760	1.24260	1.18030
H	4.59330	-0.38620	-1.76600
H	4.46190	1.35870	-1.52990
H	5.11430	-0.73340	0.60680
H	6.19540	0.48520	-0.06910
C	-3.99320	0.18060	0.69510
C	-3.96460	1.07450	-0.53820
H	-3.72340	-1.85810	1.31090
H	-5.04210	0.10290	1.00300
H	-3.45620	0.67050	1.51270
H	-4.62340	0.68420	-1.31640
H	-4.26830	2.09430	-0.28680
H	2.88380	-1.18820	0.86020
H	2.76360	-1.91180	-0.74050
H	3.94450	0.99690	1.54330
H	-0.75130	1.62880	-1.04260

[5.5]PCP 27 StrainViz fragment r2

Cartesian coordinates

C	-1.70950	2.56160	-1.03010
C	-0.37220	2.21580	-0.79800
C	0.12830	0.96260	-1.18160
C	-0.76560	0.07640	-1.80240
C	-2.09910	0.38240	-2.00150
C	-2.57760	1.63510	-1.60460
H	-2.05280	3.55150	-0.75680
H	-0.40050	-0.89410	-2.12240
C	-3.25220	0.03320	1.24400
C	-2.02860	0.14500	1.91270
C	-1.33190	-1.03210	2.19780
C	-1.85740	-2.27770	1.85450
C	-3.09060	-2.38780	1.20300
C	-3.77380	-1.20700	0.89240
H	-3.80310	0.93620	0.98880
H	-0.37130	-0.98380	2.70380
H	-1.30380	-3.18050	2.10400
H	-4.72660	-1.26270	0.37020
O	-1.39870	-2.81350	-1.34050
C	-3.64780	-3.73550	0.80600
C	0.01750	1.63570	2.30040
C	-1.51080	1.50500	2.32620
H	-3.21070	-4.50720	1.45090
H	0.42050	1.07380	1.45120
H	0.44120	1.17300	3.19910
C	-4.42330	3.11100	-1.39440
O	-3.90010	1.86600	-1.81570
H	-5.48870	3.08460	-1.62000
H	-4.28100	3.25570	-0.31640

H	-3.95650	3.94200	-1.93500
C	0.51820	3.07980	2.21640
C	0.08630	3.81600	0.95160
O	0.49100	3.11060	-0.22420
H	0.16520	3.66140	3.07850
H	1.61310	3.07710	2.25700
H	-0.99310	3.99170	0.94310
H	0.57760	4.79060	0.89690
C	-3.37590	-4.11280	-0.65990
C	-1.89310	-4.10690	-1.02850
H	-4.72930	-3.74890	0.98130
H	-3.78780	-5.11210	-0.84170
H	-3.89610	-3.42060	-1.33370
H	-1.30490	-4.56640	-0.21850
H	-1.72830	-4.70470	-1.92910
C	1.51230	0.51590	-0.88890
C	2.62990	1.35190	-1.02320
C	1.73470	-0.80020	-0.46790
C	3.90690	0.88070	-0.74950
H	2.49350	2.37610	-1.35140
C	3.01960	-1.26580	-0.19550
H	0.88740	-1.47190	-0.34550
C	4.13620	-0.43700	-0.32800
H	4.74680	1.55990	-0.87340
H	3.13590	-2.29370	0.13080
C	5.56390	-0.90560	-0.03670
C	6.16670	-0.03970	1.08250
H	5.57740	-0.12860	2.00110
H	7.19140	-0.36290	1.29820
H	6.19910	1.01720	0.80070

C	5.61080	-2.37060	0.40890
H	6.64850	-2.65950	0.60440
H	5.03900	-2.53020	1.32930
H	5.21600	-3.03920	-0.36340
C	6.41800	-0.75940	-1.30760
H	6.00910	-1.36740	-2.12130
H	6.45940	0.27980	-1.64810
H	7.44400	-1.09100	-1.11190
H	-1.97000	2.24520	1.66230
H	-1.87420	1.74250	3.33560
H	-2.78030	-0.33270	-2.45050
H	-1.58460	-2.22430	-0.59140

[5.5]PCP 27 StrainViz fragment r3

Cartesian coordinates

C	-1.68020	2.39190	-0.94760
C	-0.32980	2.06990	-0.77080
C	0.15620	0.78160	-1.06670
C	-0.77290	-0.14980	-1.52260
C	-2.13240	0.12330	-1.66550
C	-2.59260	1.42360	-1.36240
H	-2.00730	3.40460	-0.75370
H	-0.42390	-1.15090	-1.76940
C	-2.98150	0.04760	1.58200
C	-1.70200	0.24600	2.11110
C	-0.90340	-0.88110	2.32230
C	-1.37700	-2.15960	2.03200
C	-2.66050	-2.35710	1.51680
C	-3.45270	-1.22770	1.28710
H	-3.61760	0.90970	1.38890

H	0.10120	-0.76620	2.72120
H	-0.73930	-3.02220	2.21570
H	-4.44740	-1.34870	0.86330
C	-2.99620	-0.99410	-2.07100
O	-4.20960	-1.01310	-2.11020
C	-3.17390	-3.73970	1.18950
C	0.29320	1.83850	2.33100
C	-1.22530	1.64320	2.44840
H	-2.60590	-4.48440	1.76090
H	0.68390	1.21360	1.52080
H	0.78150	1.48350	3.24560
C	-4.38930	2.95580	-1.17350
O	-3.90910	1.66320	-1.50260
H	-5.46760	2.91850	-1.31990
H	-4.16790	3.20260	-0.12880
H	-3.95580	3.71790	-1.82970
C	0.72640	3.28420	2.07700
C	0.21360	3.85650	0.75820
O	0.56290	2.99780	-0.33560
H	0.37950	3.94340	2.88350
H	1.82080	3.32900	2.07270
H	-0.86650	4.01840	0.78910
H	0.68750	4.81750	0.54660
C	-3.07730	-4.07740	-0.30660
C	-1.63580	-4.11170	-0.80720
H	-4.21970	-3.82270	1.50690
H	-3.54860	-5.05180	-0.47910
H	-3.65990	-3.34190	-0.87530
H	-1.05750	-4.87490	-0.27410
H	-1.58780	-4.33800	-1.87700

C	1.56650	0.38270	-0.84230
C	2.64710	1.18500	-1.23240
C	1.84980	-0.84400	-0.23500
C	3.95300	0.76190	-1.02760
H	2.45830	2.14270	-1.70570
C	3.16410	-1.26230	-0.03230
H	1.02820	-1.47030	0.10710
C	4.24570	-0.47060	-0.42460
H	4.76440	1.40880	-1.35120
H	3.33170	-2.22060	0.44740
C	5.70380	-0.88920	-0.22130
C	6.41160	0.14550	0.67000
H	5.92660	0.20920	1.64980
H	7.45790	-0.14200	0.82180
H	6.39840	1.14250	0.21890
C	5.82220	-2.26280	0.44650
H	6.87960	-2.51960	0.56650
H	5.36090	-2.26930	1.43980
H	5.35320	-3.04640	-0.15790
C	6.41020	-0.95220	-1.58630
H	5.92470	-1.68390	-2.24040
H	6.39660	0.01780	-2.09260
H	7.45670	-1.24900	-1.45460
H	-1.75430	2.33960	1.78780
H	-1.54290	1.90490	3.46700
H	-2.41910	-1.90370	-2.34210
H	-1.14060	-3.14830	-0.64030

[5.5]PCP 27 StrainViz fragment r4

Cartesian coordinates

C	-1.50820	2.20040	-1.04710
C	-0.16380	1.87820	-0.85390
C	0.29310	0.55790	-1.02340
C	-0.65890	-0.40470	-1.35130
C	-2.01480	-0.12240	-1.51740
C	-2.44480	1.21320	-1.35450
H	-1.81830	3.23190	-0.95110
H	-0.33330	-1.43170	-1.47430
C	-2.99980	0.30550	1.68840
C	-1.67190	0.43750	2.09600
C	-0.90440	-0.72920	2.18640
C	-1.44720	-1.96800	1.86400
C	-2.77700	-2.09820	1.44790
C	-3.54810	-0.93720	1.37270
H	-3.61770	1.19710	1.59480
H	0.13510	-0.67180	2.49920
H	-0.82500	-2.85880	1.93390
C	-2.96270	-1.21830	-1.81540
O	-4.15240	-1.11770	-2.02370
O	-2.35590	-2.42830	-1.84490
C	-3.31400	-3.47360	1.10590
C	0.41900	1.92350	2.24700
C	-1.10490	1.80790	2.40820
H	-2.63330	-3.93270	0.37860
H	0.76690	1.22270	1.48130
H	0.91250	1.61920	3.17680
C	-4.19720	2.80790	-1.26670
O	-3.75630	1.48160	-1.49680
H	-5.27920	2.78520	-1.38770
H	-3.94730	3.13590	-0.25110

H	-3.76260	3.50330	-1.99280
C	0.90900	3.32220	1.86420
C	0.42100	3.79040	0.49550
O	0.74800	2.83220	-0.51900
H	0.58810	4.06600	2.60540
H	2.00440	3.32370	1.85960
H	-0.65340	3.98950	0.50350
H	0.92650	4.71410	0.20520
C	-4.73760	-3.50110	0.55750
H	-3.25940	-4.10380	2.00290
H	-5.02710	-4.52400	0.30200
H	-5.45470	-3.12630	1.29490
C	1.70270	0.16040	-0.78870
C	2.78560	0.92120	-1.25100
C	1.98640	-1.02310	-0.10100
C	4.09070	0.50200	-1.03390
H	2.59910	1.84340	-1.79040
C	3.29980	-1.43800	0.11380
H	1.16540	-1.62060	0.28850
C	4.38260	-0.68610	-0.34740
H	4.90250	1.11650	-1.41520
H	3.46590	-2.36190	0.65710
C	5.83990	-1.10220	-0.13400
C	6.57050	-0.00940	0.66480
H	6.10220	0.13310	1.64440
H	7.61690	-0.29400	0.82160
H	6.55780	0.95050	0.13940
C	5.95670	-2.42160	0.63560
H	7.01360	-2.67760	0.76190
H	5.50810	-2.34810	1.63200

H	5.47400	-3.24510	0.09890
C	6.52450	-1.27650	-1.50040
H	6.02290	-2.05190	-2.08870
H	6.51020	-0.34810	-2.07960
H	7.57070	-1.57160	-1.36210
H	-1.61290	2.52660	1.75380
H	-1.38080	2.09810	3.43080
H	-4.57920	-0.98730	1.03550
H	-3.05120	-3.07340	-2.06410
H	-4.83190	-2.88210	-0.34110

[5.5]PCP 27 StrainViz fragment r5

Cartesian coordinates

C	-1.60760	1.80110	-1.38590
C	-0.24600	1.56630	-1.19330
C	0.24220	0.25640	-1.03270
C	-0.69070	-0.77700	-1.05860
C	-2.06170	-0.57680	-1.22190
C	-2.52510	0.74800	-1.37730
H	-1.94820	2.81580	-1.54080
H	-0.33650	-1.79430	-0.93670
C	-3.08960	0.93360	1.74950
C	-1.73200	1.07410	2.05350
C	-1.01310	-0.08260	2.36320
C	-1.62710	-1.33410	2.34710
C	-2.97900	-1.47160	2.02850
C	-3.70360	-0.31300	1.73510
H	-3.67000	1.81850	1.49370
H	0.04490	-0.01490	2.60420
H	-1.03980	-2.22130	2.57530

H	-4.75230	-0.39350	1.45910
C	-2.98360	-1.74140	-1.23250
O	-4.18380	-1.71630	-1.39370
O	-2.31620	-2.90470	-1.06530
C	-3.64670	-2.82210	2.00430
C	0.41960	2.45770	1.75570
C	-1.09500	2.44830	2.01710
H	-4.07100	-3.07440	2.98280
H	0.70870	1.56600	1.19080
H	0.96050	2.39710	2.70650
C	-4.32070	2.27600	-1.62910
O	-3.85000	0.94590	-1.51310
H	-5.40590	2.20270	-1.68370
H	-4.03660	2.87380	-0.75530
H	-3.94040	2.75510	-2.53780
C	0.91020	3.68600	0.98460
C	0.36180	3.77760	-0.43730
O	0.65580	2.58730	-1.17760
H	0.63550	4.61060	1.50930
H	2.00390	3.65860	0.93010
H	-0.71310	3.97540	-0.43900
H	0.84800	4.59060	-0.98090
C	-3.09840	-4.08430	-1.24020
H	-4.46410	-2.83260	1.27660
H	-2.41620	-4.91710	-1.07650
H	-3.50740	-4.12000	-2.25270
C	1.66920	-0.03880	-0.75580
C	2.71740	0.57830	-1.45250
C	2.00450	-0.97800	0.22390
C	4.03900	0.25700	-1.17560

H	2.49020	1.30990	-2.22020
C	3.33400	-1.29590	0.49730
H	1.21100	-1.45640	0.79350
C	4.38230	-0.68610	-0.19520
H	4.82250	0.75300	-1.74290
H	3.54040	-2.02770	1.27080
C	5.85570	-1.00090	0.07400
C	6.57980	0.28420	0.51020
H	6.13620	0.68650	1.42700
H	7.63750	0.07310	0.70340
H	6.52640	1.05860	-0.26110
C	6.02980	-2.05020	1.17650
H	7.09630	-2.24220	1.33200
H	5.60850	-1.70860	2.12800
H	5.55370	-2.99930	0.90840
C	6.50520	-1.53850	-1.21250
H	6.00810	-2.45660	-1.54300
H	6.44960	-0.80990	-2.02710
H	7.56240	-1.76450	-1.03390
H	-1.61620	3.02480	1.24300
H	-1.30050	2.97490	2.95860
H	-3.91960	-4.11860	-0.52300
H	-2.93550	-3.60970	1.73790

[5.5]PCP 27 StrainViz fragment r6

Cartesian coordinates

C	-1.66930	1.93670	-1.34540
C	-0.31380	1.65580	-1.16270
C	0.13090	0.33190	-0.99320
C	-0.84040	-0.66810	-0.98860

C	-2.20420	-0.41760	-1.13320
C	-2.62300	0.91890	-1.31110
H	-1.97190	2.95970	-1.52360
H	-0.52360	-1.69640	-0.85550
C	-2.75390	0.78790	1.95240
C	-1.41300	1.06480	2.23610
C	-0.59730	0.01200	2.66230
C	-1.10610	-1.27900	2.79280
C	-2.44400	-1.53930	2.50430
C	-3.27060	-0.49820	2.08620
H	-3.39870	1.59200	1.60220
H	0.45050	0.19620	2.88490
H	-0.45380	-2.08310	3.12270
H	-4.31020	-0.69080	1.83810
C	-3.17430	-1.54040	-1.05880
O	-4.37430	-1.44280	-0.92430
O	-2.55460	-2.73450	-1.16510
C	0.61470	2.55680	1.73930
C	-0.88270	2.47440	2.07360
H	0.91650	1.67080	1.17090
H	1.20150	2.53450	2.66430
C	-4.36510	2.49950	-1.61740
O	-3.94090	1.15750	-1.46200
H	-5.45120	2.46080	-1.68740
H	-4.07520	3.10770	-0.75280
H	-3.95530	2.94380	-2.53100
C	1.01230	3.79620	0.93430
C	0.37020	3.85710	-0.44930
O	0.61880	2.64860	-1.17750
H	0.74100	4.71520	1.47020

H	2.10090	3.80370	0.81240
H	-0.70340	4.04840	-0.38000
H	0.81260	4.66140	-1.04150
C	-3.74750	-4.24210	0.33520
C	-3.39960	-3.89260	-1.09940
H	-4.34530	-5.15760	0.36100
H	-4.32520	-3.43480	0.79240
H	-2.81550	-4.68410	-1.57160
H	-4.29880	-3.70720	-1.69070
C	1.55540	-0.01390	-0.76540
C	2.59630	0.57060	-1.50040
C	1.89610	-0.97020	0.19580
C	3.91580	0.20140	-1.27880
H	2.36510	1.31360	-2.25570
C	3.22370	-1.33550	0.41360
H	1.11070	-1.42170	0.79700
C	4.26460	-0.75940	-0.31800
H	4.69280	0.67240	-1.87560
H	3.43500	-2.07890	1.17460
C	5.73520	-1.13060	-0.11310
C	6.52650	0.12410	0.29340
H	6.13940	0.54010	1.22950
H	7.58270	-0.12830	0.43960
H	6.46960	0.90260	-0.47350
C	5.91650	-2.18980	0.97860
H	6.98070	-2.42230	1.08800
H	5.54910	-1.83640	1.94790
H	5.39420	-3.11940	0.72880
C	6.30690	-1.68790	-1.42790
H	5.76070	-2.58460	-1.73870

H	6.24410	-0.95430	-2.23750
H	7.36140	-1.95530	-1.29630
H	-1.47750	2.95800	1.28990
H	-1.08000	3.04950	2.98820
H	-2.84020	-2.54430	2.60990
H	-2.83750	-4.40480	0.91910

[5.5]PCP 27 StrainViz fragment r7

Cartesian coordinates

C	-1.85620	2.23910	-0.32560
C	-0.56210	1.71730	-0.32270
C	-0.34000	0.32250	-0.32460
C	-1.46290	-0.49460	-0.33190
C	-2.77480	-0.00750	-0.32730
C	-2.96730	1.38910	-0.32570
H	-1.99880	3.30960	-0.32350
H	-1.32100	-1.56960	-0.34860
C	-3.91420	-0.95870	-0.29910
O	-5.06330	-0.69230	-0.02430
O	-3.51420	-2.20970	-0.61180
C	-3.33510	-4.11520	1.59760
C	2.68940	3.89430	0.90680
C	2.08900	3.97770	2.30880
H	-2.90020	-4.94990	1.03730
H	2.88820	2.84650	0.65630
H	3.65420	4.41310	0.88650
C	-4.43400	3.25700	-0.37790
O	-4.23050	1.85480	-0.34550
H	-5.51360	3.39750	-0.39920
H	-4.02050	3.73780	0.51500

H	-3.98860	3.70120	-1.27450
C	1.79300	4.50350	-0.17510
C	0.39360	3.91320	-0.20460
O	0.53590	2.50010	-0.30380
H	1.69020	5.58400	-0.01620
H	2.25530	4.36520	-1.15890
H	-0.16400	4.15860	0.70850
H	-0.17080	4.29260	-1.06540
C	-4.65610	-3.67010	0.97490
C	-4.49880	-3.24540	-0.47760
H	-3.47760	-4.43950	2.63180
H	-5.38550	-4.48780	1.00450
H	-5.08570	-2.83810	1.54230
H	-4.11200	-4.06710	-1.08520
H	-5.44490	-2.89640	-0.89710
C	1.02090	-0.26970	-0.27810
C	2.08070	0.21820	-1.05670
C	1.27310	-1.37530	0.53770
C	3.33080	-0.38320	-1.01140
H	1.92010	1.07270	-1.70490
C	2.53020	-1.97670	0.57670
H	0.47830	-1.76520	1.16850
C	3.58930	-1.49430	-0.19480
H	4.12360	0.02310	-1.63440
H	2.67270	-2.82910	1.23190
C	4.98650	-2.11830	-0.17930
C	6.01090	-1.06330	0.27180
H	5.77790	-0.70310	1.27920
H	7.01690	-1.49750	0.28520
H	6.02530	-0.20130	-0.40230

C	5.07370	-3.31390	0.77450
H	6.08790	-3.72550	0.75170
H	4.85290	-3.02270	1.80680
H	4.38150	-4.11160	0.48470
C	5.34420	-2.60150	-1.59530
H	4.62940	-3.35640	-1.93900
H	5.34070	-1.77740	-2.31540
H	6.34490	-3.04790	-1.59870
H	1.19780	3.34790	2.39600
H	1.80170	5.00620	2.55560
H	2.80530	3.63870	3.06230
H	-2.60920	-3.29630	1.59710

[5.5]PCP 27 StrainViz fragment r8

Cartesian coordinates

C	-1.24380	2.36220	-0.70840
C	0.06220	1.88440	-0.59670
C	0.35100	0.51400	-0.76550
C	-0.71810	-0.32510	-1.05200
C	-2.03760	0.11710	-1.17630
C	-2.29970	1.48780	-0.98610
H	-1.44200	3.41320	-0.55800
H	-0.52410	-1.38410	-1.18100
C	-3.43120	0.65420	2.00920
C	-2.06600	0.74730	2.27590
C	-1.27860	-0.39980	2.21950
C	-1.85460	-1.62750	1.89640
C	-3.22010	-1.73470	1.62380
C	-4.00060	-0.57500	1.68750
H	-4.05720	1.54140	2.04980

H	-0.21090	-0.33800	2.41280
H	-1.23270	-2.51890	1.84410
H	-5.06680	-0.63260	1.48090
C	-3.12310	-0.85900	-1.43350
O	-4.25650	-0.60480	-1.77790
O	-2.69810	-2.12530	-1.23170
C	-3.84930	-3.07210	1.28670
C	2.87660	3.94940	1.57180
H	-3.07370	-3.84780	1.28700
H	3.07020	2.90120	1.32840
H	3.82680	4.42360	1.83100
C	-3.86370	3.24930	-0.71180
O	-3.57870	1.90530	-1.05290
H	-4.94780	3.34390	-0.76000
H	-3.51870	3.47880	0.30300
H	-3.40580	3.94730	-1.42100
C	2.21440	4.66020	0.39440
C	0.87210	4.05170	0.03160
O	1.10650	2.68830	-0.30190
H	2.05000	5.71820	0.62600
H	2.86500	4.62200	-0.48650
H	0.17610	4.10360	0.88070
H	0.42040	4.57170	-0.82250
C	-4.60910	-3.10850	-0.04850
C	-3.70220	-3.14490	-1.27200
H	-4.55350	-3.33710	2.08480
H	-5.23350	-4.00850	-0.08460
H	-5.28310	-2.25020	-0.12260
H	-3.13380	-4.07750	-1.30370
H	-4.28570	-3.04690	-2.19130

C	1.71160	-0.04630	-0.57180
C	2.86470	0.56800	-1.08170
C	1.87280	-1.24820	0.12280
C	4.11530	-0.00570	-0.89840
H	2.77680	1.50000	-1.62930
C	3.13130	-1.82040	0.30210
H	0.99840	-1.73820	0.54480
C	4.28260	-1.21220	-0.20240
H	4.98300	0.49990	-1.31460
H	3.20010	-2.75170	0.85390
C	5.68440	-1.80010	-0.02500
C	6.56820	-0.79290	0.73020
H	6.15160	-0.57760	1.71980
H	7.57620	-1.20190	0.86250
H	6.65740	0.15250	0.18630
C	5.66390	-3.11160	0.76650
H	6.68520	-3.49260	0.86760
H	5.25820	-2.96780	1.77360
H	5.06840	-3.87860	0.26000
C	6.29890	-2.07870	-1.40730
H	5.68830	-2.79610	-1.96530
H	6.37980	-1.16490	-2.00410
H	7.30510	-2.49750	-1.29420
H	2.23110	3.97860	2.45620
H	-1.61860	1.70720	2.51670

[5.5]PCP 27 StrainViz fragment r9

Cartesian coordinates

C	-1.22630	2.33880	-1.05140
C	0.11020	1.96730	-0.91270

C	0.49770	0.61980	-0.92900
C	-0.51080	-0.32380	-1.12570
C	-1.85240	0.01380	-1.29380
C	-2.22120	1.37590	-1.23580
H	-1.46180	3.39620	-1.02680
H	-0.24160	-1.37380	-1.15210
C	-3.54580	0.65360	1.75860
C	-2.23240	0.93890	2.14710
C	-1.32940	-0.12180	2.21780
C	-1.72840	-1.42580	1.92380
C	-3.04240	-1.71370	1.55500
C	-3.94490	-0.64660	1.47230
H	-4.26950	1.46350	1.68900
H	-0.29970	0.06920	2.51440
H	-1.00390	-2.23540	1.98530
H	-4.97640	-0.83320	1.18320
C	-2.85610	-1.06120	-1.51360
O	-3.99240	-0.91510	-1.90490
O	-2.33910	-2.27340	-1.22760
C	-3.49330	-3.13670	1.28900
C	-1.83090	2.34910	2.49770
H	-2.62890	-3.80850	1.35880
C	-3.90510	3.04000	-1.18180
O	-3.52590	1.68760	-1.35760
H	-4.99100	3.05730	-1.26490
H	-3.60710	3.40680	-0.19260
H	-3.46960	3.68080	-1.95600
C	1.75630	2.69220	1.54020
C	1.11210	3.58750	0.49660
O	1.05350	2.95270	-0.78640

H	1.77860	3.20150	2.50870
H	2.77850	2.43540	1.24880
H	0.10080	3.88450	0.80230
H	1.70020	4.49390	0.33760
C	-4.20210	-3.35310	-0.05680
C	-3.25240	-3.37610	-1.24740
H	-4.18290	-3.43650	2.08780
H	-4.72330	-4.31690	-0.04170
H	-4.96290	-2.58290	-0.21240
H	-2.60600	-4.25580	-1.20860
H	-3.80520	-3.37560	-2.19040
C	1.89530	0.18530	-0.69110
C	2.99080	0.80350	-1.30830
C	2.14970	-0.87330	0.18340
C	4.28440	0.37220	-1.04880
H	2.82260	1.62820	-1.99250
C	3.45130	-1.30130	0.44050
H	1.31590	-1.35610	0.68850
C	4.54870	-0.68680	-0.16680
H	5.10890	0.87370	-1.54930
H	3.59720	-2.12200	1.13440
C	5.99460	-1.11610	0.09300
C	6.78180	0.07210	0.67160
H	6.33920	0.40670	1.61570
H	7.82000	-0.22150	0.86290
H	6.79390	0.92170	-0.01800
C	6.08190	-2.28060	1.08450
H	7.13150	-2.55120	1.23750
H	5.65920	-2.01290	2.05870
H	5.55800	-3.16720	0.71200

C	6.64110	-1.55800	-1.23100
H	6.09910	-2.40600	-1.66260
H	6.64660	-0.74820	-1.96690
H	7.67890	-1.86420	-1.05880
H	-1.98930	3.02680	1.65140
H	-2.42430	2.73050	3.33560
H	1.19090	1.76090	1.65050
H	-0.77610	2.40430	2.78070

[5.5]PCP 27 StrainViz fragment r10

Cartesian coordinates

C	-1.31130	2.40740	-0.90940
C	0.03150	2.06490	-0.74670
C	0.45890	0.72730	-0.86930
C	-0.51410	-0.22190	-1.15750
C	-1.86690	0.08230	-1.32630
C	-2.26800	1.42610	-1.18690
H	-1.61690	3.43770	-0.80080
H	-0.21240	-1.25910	-1.25000
C	-3.15350	0.42190	1.87420
C	-1.80750	0.62560	2.20330
C	-0.95820	-0.47930	2.16060
C	-1.43710	-1.74010	1.80130
C	-2.77830	-1.94400	1.48160
C	-3.63270	-0.83520	1.52590
H	-3.83800	1.26860	1.89250
H	0.09600	-0.36370	2.39680
H	-0.74960	-2.58330	1.76870
H	-4.68510	-0.95370	1.27850
C	-2.84430	-1.00440	-1.57620

O	-4.00500	-0.87010	-1.89690
O	-2.28310	-2.21890	-1.39520
C	-3.30020	-3.32290	1.12960
C	0.16850	2.16280	2.78750
C	-1.33900	2.00920	2.60700
H	-2.46300	-4.03180	1.11650
H	0.71200	1.88840	1.87680
H	0.53730	1.53200	3.60260
C	-4.00080	3.04050	-1.04100
O	-3.57760	1.71670	-1.31050
H	-5.08520	3.03090	-1.14180
H	-3.72950	3.34120	-0.02220
H	-3.57510	3.75030	-1.75850
C	0.57430	4.30430	-0.14600
O	0.98040	2.97820	-0.43470
H	-0.14280	4.32230	0.68290
H	0.13260	4.78500	-1.02530
C	-4.06510	-3.41200	-0.19930
C	-3.17270	-3.34010	-1.43180
H	-3.97340	-3.65560	1.92960
H	-4.59660	-4.36960	-0.24470
H	-4.82360	-2.62600	-0.25350
H	-2.50770	-4.20580	-1.47730
H	-3.77330	-3.29890	-2.34440
C	1.86490	0.31090	-0.64160
C	2.95640	1.02140	-1.16100
C	2.13570	-0.85060	0.08690
C	4.25520	0.57770	-0.95550
H	2.78160	1.92440	-1.73580
C	3.44270	-1.29080	0.29030

H	1.30890	-1.41520	0.51100
C	4.53320	-0.58710	-0.22460
H	5.07290	1.15390	-1.38120
H	3.59740	-2.19610	0.86740
C	5.98410	-1.03180	-0.02530
C	6.76250	0.07690	0.70330
H	6.32270	0.27790	1.68570
H	7.80470	-0.22860	0.84850
H	6.76190	1.01110	0.13330
C	6.08510	-2.31610	0.80370
H	7.13780	-2.59310	0.92010
H	5.66040	-2.18390	1.80450
H	5.57060	-3.15130	0.31700
C	6.63050	-1.28880	-1.39720
H	6.09450	-2.07680	-1.93650
H	6.62660	-0.38850	-2.01930
H	7.67160	-1.60580	-1.26930
H	-1.69140	2.72340	1.85140
H	-1.84420	2.29190	3.54020
H	1.47940	4.83660	0.14330
H	0.41890	3.19920	3.03260

[5.5]PCP 27 StrainViz fragment r11

Cartesian coordinates

C	-1.35230	2.27500	-1.38940
C	-0.01390	1.93650	-1.18690
C	0.39510	0.59600	-1.09340
C	-0.60390	-0.36900	-1.20980
C	-1.95100	-0.07020	-1.40880
C	-2.33220	1.28770	-1.48720

H	-1.61740	3.32510	-1.43970
H	-0.32360	-1.41390	-1.13970
C	-2.94630	0.82070	1.75670
C	-1.57760	0.94220	2.02480
C	-0.84010	-0.23420	2.15860
C	-1.44670	-1.48310	2.02250
C	-2.81060	-1.60340	1.76080
C	-3.55410	-0.42380	1.63300
H	-3.54600	1.72230	1.64190
H	0.22640	-0.18550	2.35950
H	-0.84330	-2.38330	2.12190
H	-4.62110	-0.47740	1.42760
C	-2.95310	-1.16220	-1.46250
O	-4.10720	-1.06060	-1.81710
O	-2.42630	-2.33410	-1.04990
C	-3.46890	-2.96410	1.65040
C	0.56470	2.35290	2.27280
C	-0.95820	2.31750	2.16650
H	-2.70660	-3.74290	1.77700
H	1.00720	1.81650	1.42370
H	0.88270	1.82330	3.17950
C	-4.02890	2.93790	-1.59240
O	-3.63920	1.57690	-1.63070
H	-5.11490	2.93810	-1.67230
H	-3.72910	3.40660	-0.64790
H	-3.60140	3.49790	-2.43120
C	1.09440	3.78310	2.30490
O	0.92350	2.90670	-1.04250
H	0.67440	4.34110	3.14880
H	2.18410	3.80300	2.39440

C	-4.24050	-3.21560	0.34670
C	-3.35130	-3.41320	-0.87380
H	-4.17130	-3.08160	2.48510
H	-4.84100	-4.12610	0.45640
H	-4.93990	-2.39650	0.15490
H	-2.71600	-4.29370	-0.75200
H	-3.95430	-3.52620	-1.77860
C	1.79880	0.19600	-0.82790
C	2.89280	0.84140	-1.42230
C	2.06730	-0.88150	0.02200
C	4.19030	0.41640	-1.17200
H	2.72250	1.67730	-2.09170
C	3.37240	-1.30360	0.26810
H	1.24100	-1.39290	0.50930
C	4.46530	-0.66470	-0.32180
H	5.00940	0.94010	-1.65830
H	3.52440	-2.14250	0.93870
C	5.91440	-1.09270	-0.07890
C	6.69730	0.08140	0.53310
H	6.25780	0.38340	1.48950
H	7.73820	-0.21190	0.70970
H	6.70050	0.95240	-0.12940
C	6.01100	-2.28680	0.87590
H	7.06250	-2.55640	1.01740
H	5.58990	-2.05150	1.85910
H	5.49040	-3.16420	0.47770
C	6.55910	-1.49020	-1.41780
H	6.02010	-2.32720	-1.87400
H	6.55840	-0.65790	-2.12830
H	7.59900	-1.79640	-1.25830

H	-1.26820	2.92610	1.30550
H	-1.38740	2.81350	3.04900
H	0.82480	4.31340	1.38530
H	0.50180	3.77800	-1.09980

[5.5]PCP 27 StrainViz fragment r12

Cartesian coordinates

C	-1.30570	2.14510	-1.67820
C	0.00220	1.71790	-1.48170
C	0.30810	0.37650	-1.24440
C	-0.75340	-0.53180	-1.23310
C	-2.07460	-0.13730	-1.43160
C	-2.36290	1.23110	-1.64140
H	-1.49450	3.19990	-1.83530
H	-0.55780	-1.58450	-1.06500
C	-2.74710	1.05080	1.69840
C	-1.36280	1.04120	1.90900
C	-0.75030	-0.19660	2.10710
C	-1.49290	-1.37760	2.09130
C	-2.87230	-1.36600	1.89100
C	-3.48990	-0.12470	1.69410
H	-3.25100	2.00210	1.53490
H	0.32260	-0.25270	2.26640
H	-0.98560	-2.32870	2.24060
H	-4.56470	-0.07530	1.53270
C	-3.16180	-1.15340	-1.36250
O	-4.31400	-0.98970	-1.69490
O	-2.71240	-2.32210	-0.86470
C	-3.67620	-2.65040	1.92060
C	0.91520	2.22330	2.09040

C	-0.59820	2.34990	1.92460
H	-2.99650	-3.49480	2.08870
H	1.32160	1.57650	1.29910
H	1.12740	1.72210	3.04320
C	-3.94130	2.97920	-1.89200
O	-3.65340	1.59760	-1.77300
H	-5.02670	3.05210	-1.94600
H	-3.57830	3.53460	-1.01960
H	-3.50120	3.40080	-2.80200
C	1.63860	3.57280	2.05930
C	1.71440	4.18760	0.66260
H	1.13900	4.26830	2.74640
H	2.65740	3.44060	2.44090
H	0.71940	4.34140	0.23080
H	2.22130	5.15690	0.68120
C	-4.52500	-2.92400	0.67060
C	-3.71270	-3.30220	-0.56040
H	-4.35100	-2.62080	2.78530
H	-5.20510	-3.75880	0.87580
H	-5.15040	-2.05700	0.43750
H	-3.14560	-4.21960	-0.38600
H	-4.36280	-3.43870	-1.42850
C	1.69910	-0.06070	-0.97930
C	2.79360	0.59430	-1.56130
C	1.96800	-1.12740	-0.11670
C	4.09430	0.19600	-1.28470
H	2.62470	1.41090	-2.25810
C	3.27580	-1.52590	0.15350
H	1.14420	-1.64260	0.37030
C	4.36980	-0.87390	-0.42100

H	4.91300	0.72730	-1.76320
H	3.42990	-2.35630	0.83410
C	5.82060	-1.27800	-0.14930
C	6.57560	-0.08570	0.46250
H	6.11490	0.22120	1.40730
H	7.61720	-0.36210	0.66090
H	6.57900	0.77750	-0.21020
C	5.91800	-2.46000	0.82040
H	6.97080	-2.71180	0.98320
H	5.47620	-2.22040	1.79350
H	5.41810	-3.34960	0.42300
C	6.49320	-1.68060	-1.47280
H	5.97440	-2.53040	-1.92860
H	6.49240	-0.85620	-2.19250
H	7.53460	-1.96940	-1.29240
H	-0.82740	2.88780	0.99600
H	-0.98910	2.97710	2.73850
H	2.27510	3.52850	-0.01090
H	0.79470	2.46040	-1.48130

9. Copies of NMR spectra

2, ^{1}H NMR (600 MHz, $CDCl_3$)

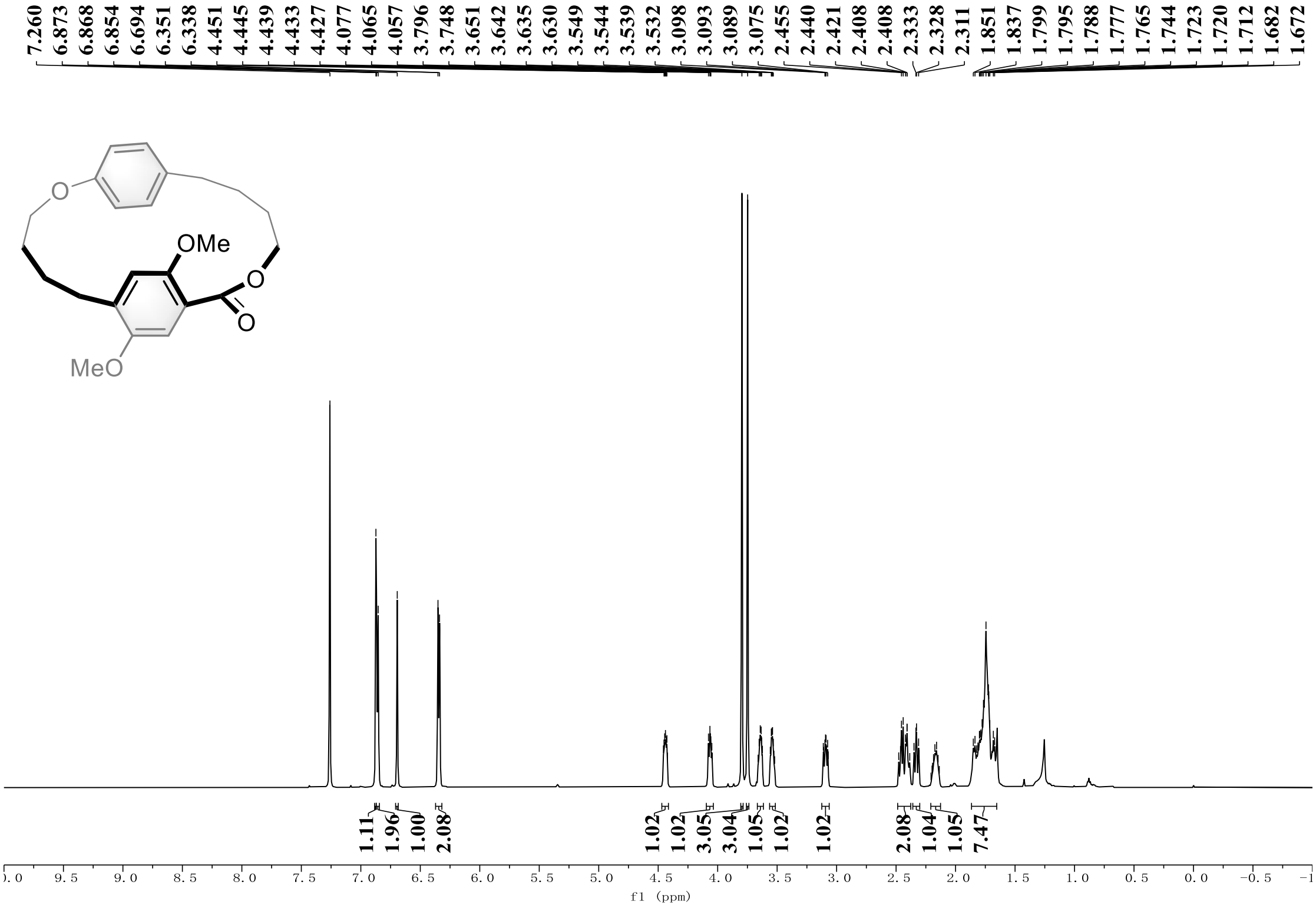

2, ^{13}C NMR (151 MHz, $CDCl_3$)

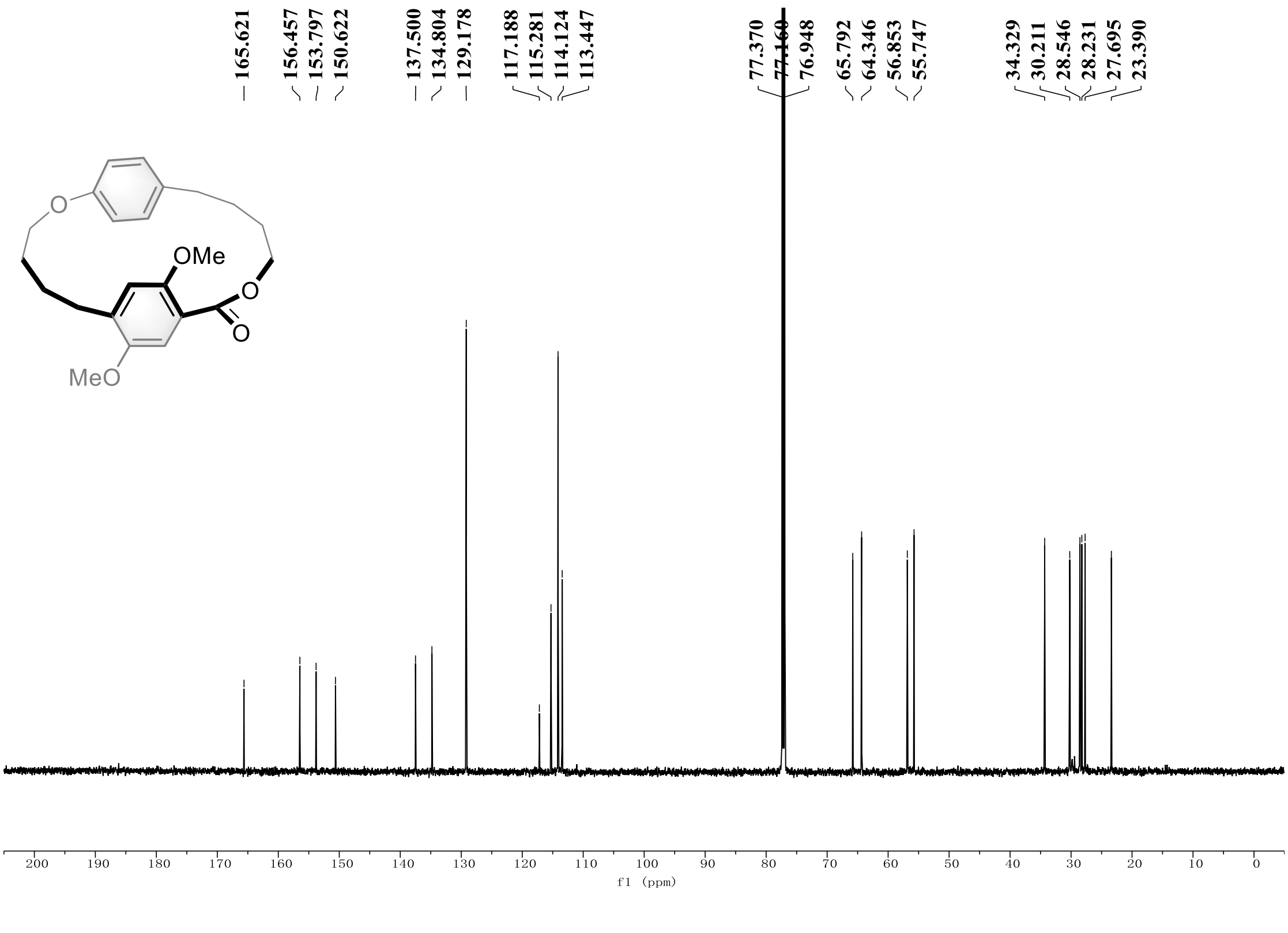

3, ^{1}H NMR (600 MHz, $CDCl_3$)

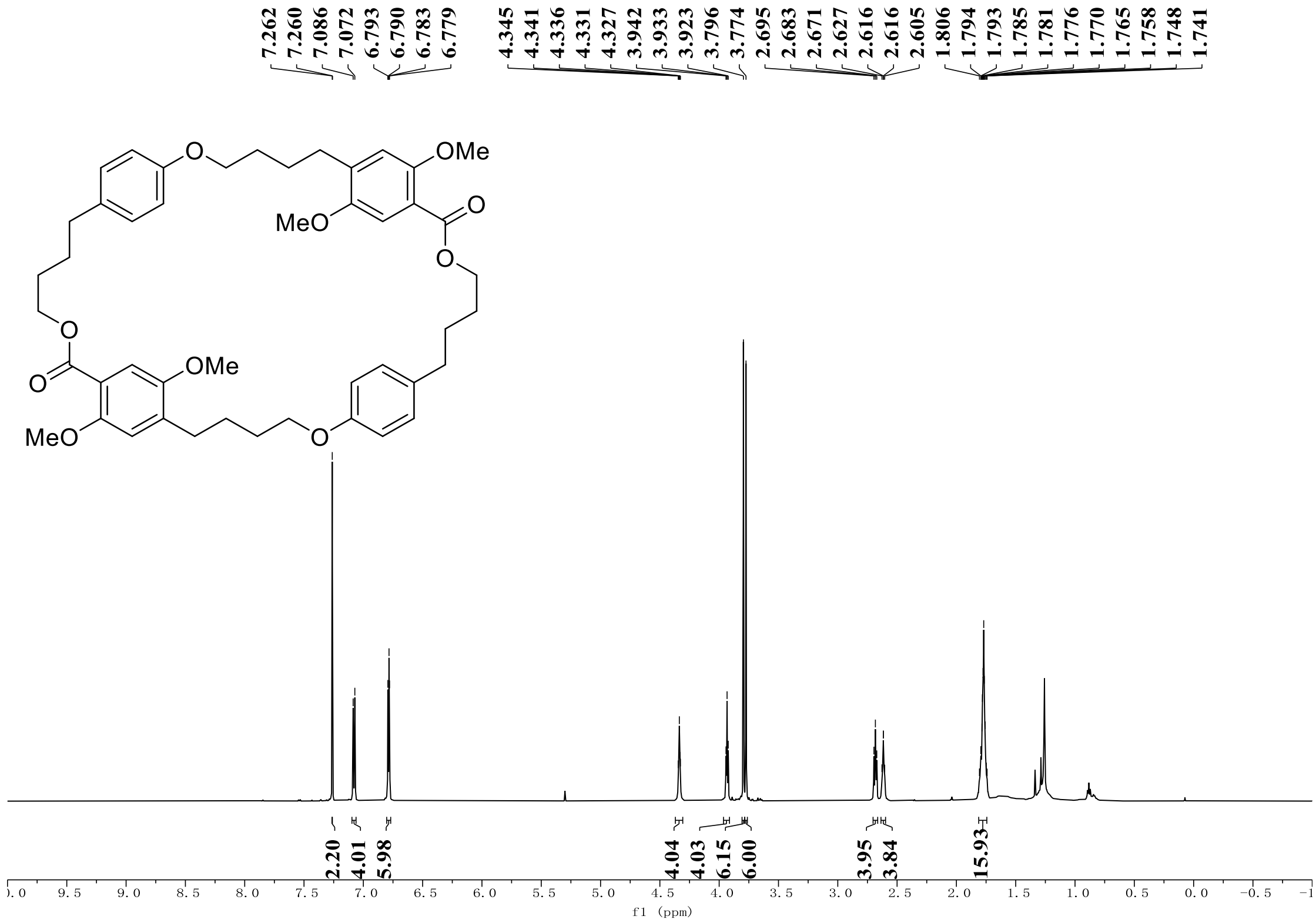

3, ^{13}C NMR (151 MHz, $CDCl_3$)

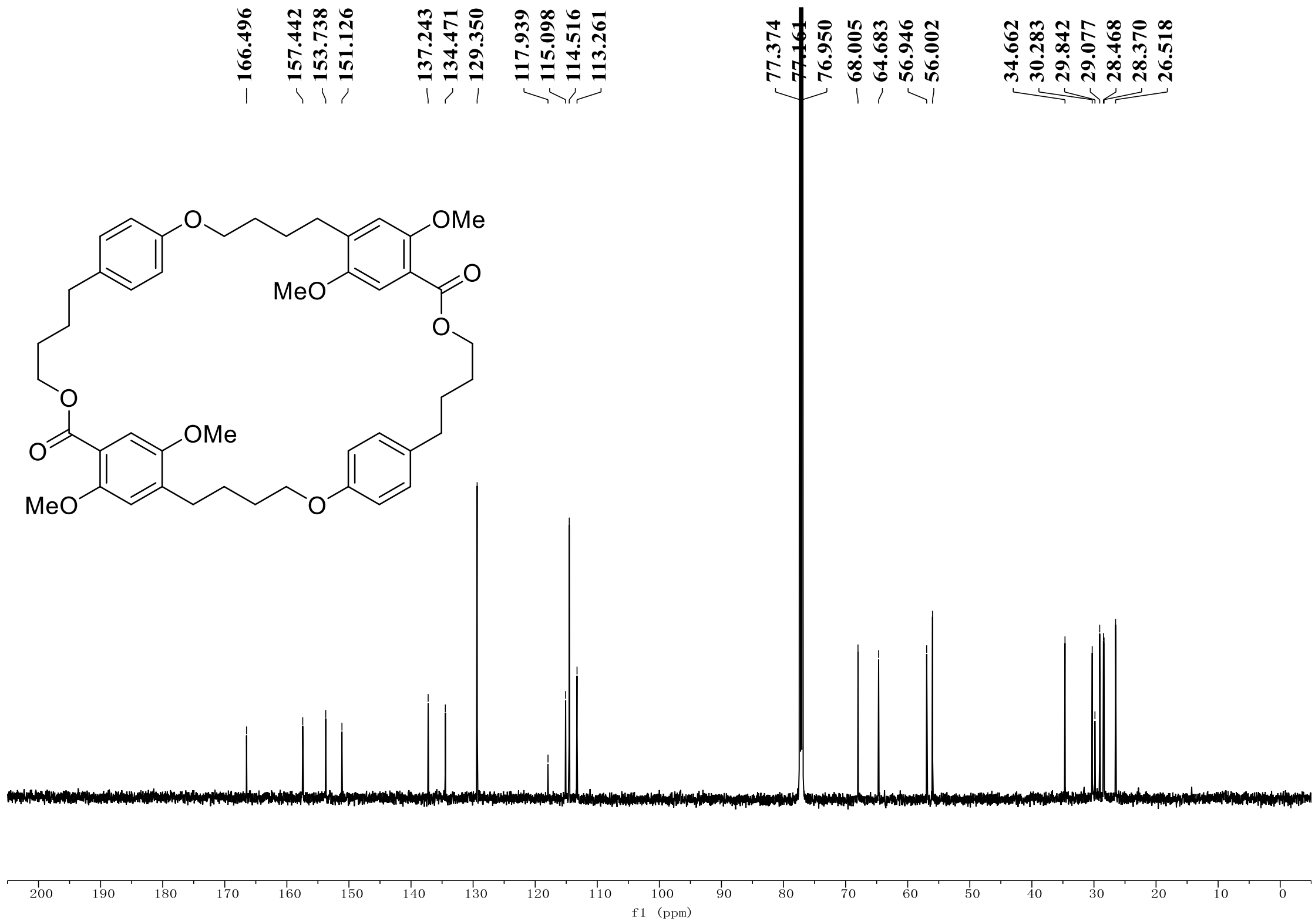

4, ^{1}H NMR (600 MHz, $CDCl_3$)

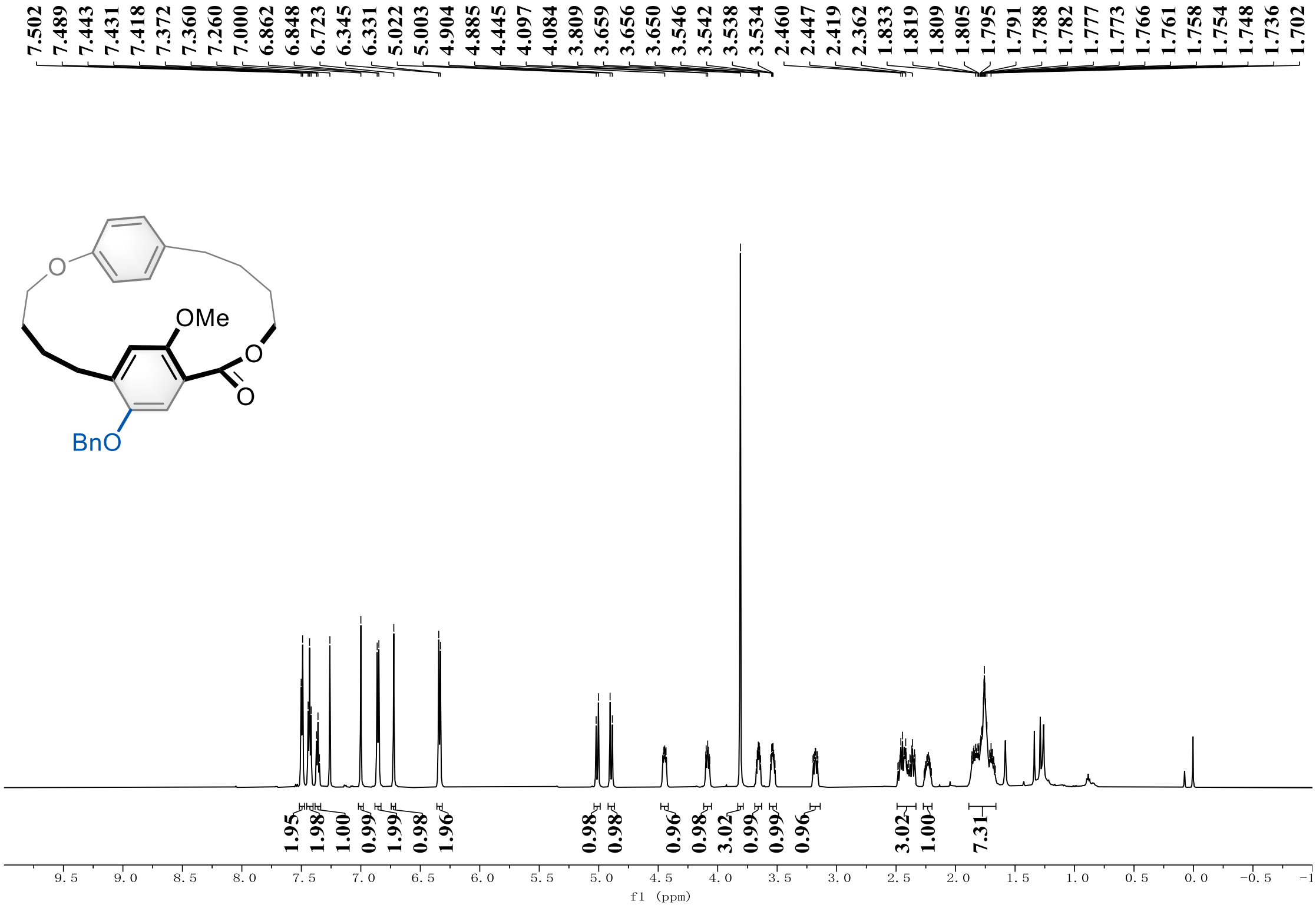

4, ^{13}C NMR (151 MHz, $CDCl_3$)

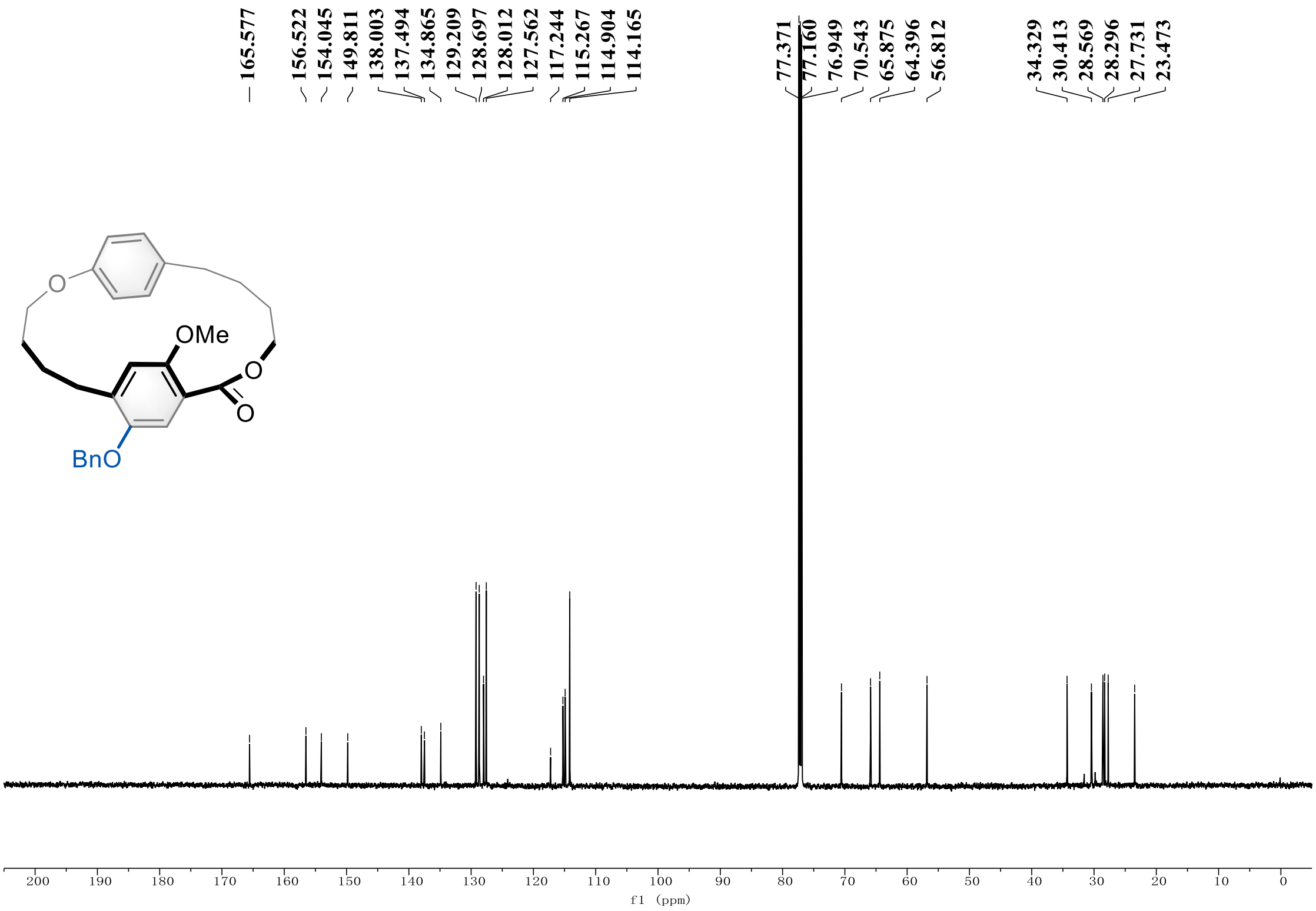

5, ^{1}H NMR (600 MHz, $CDCl_3$)

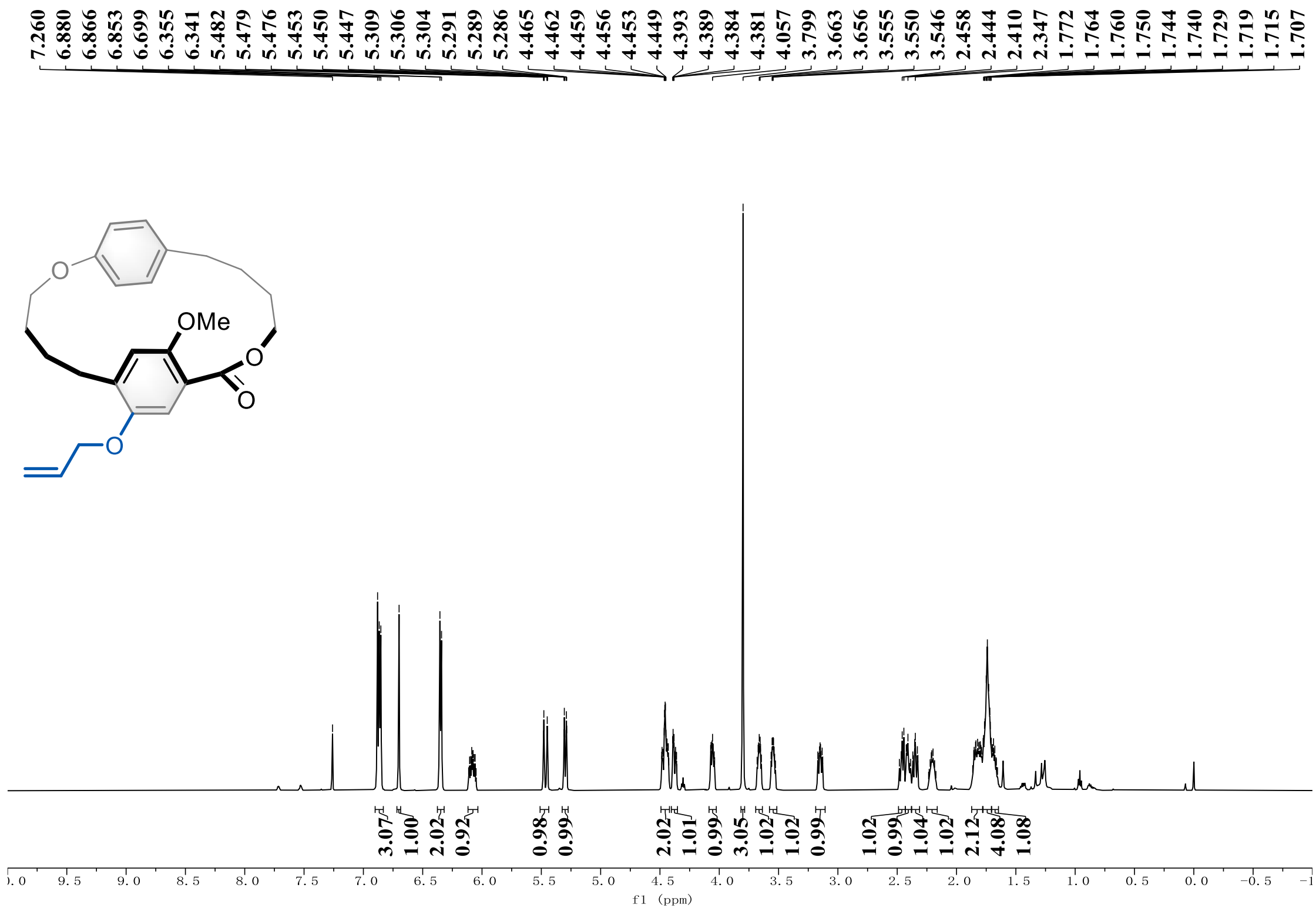

5, ^{13}C NMR (151 MHz, $CDCl_3$)

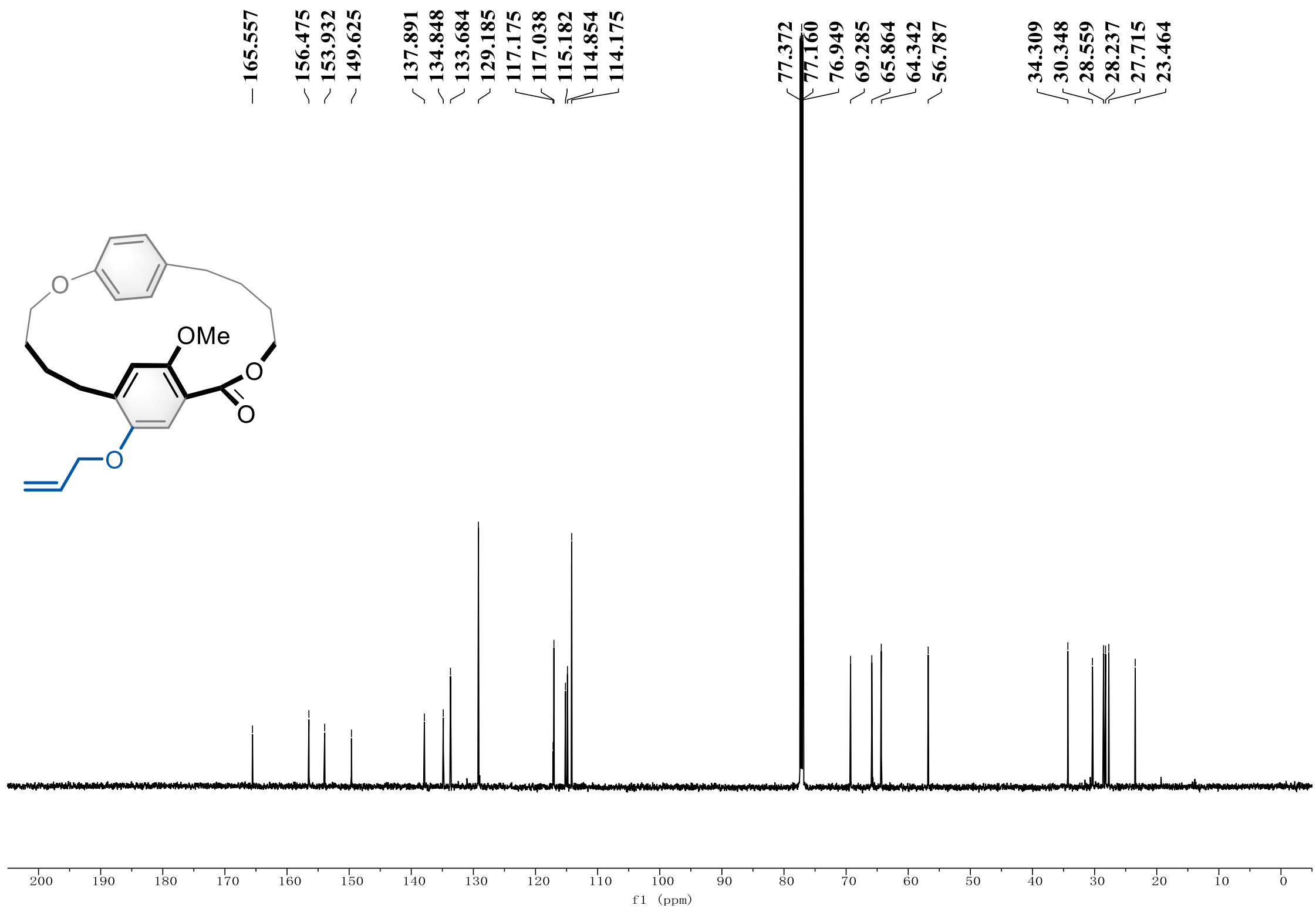

6, ¹H NMR (600 MHz, $CDCl_3$)

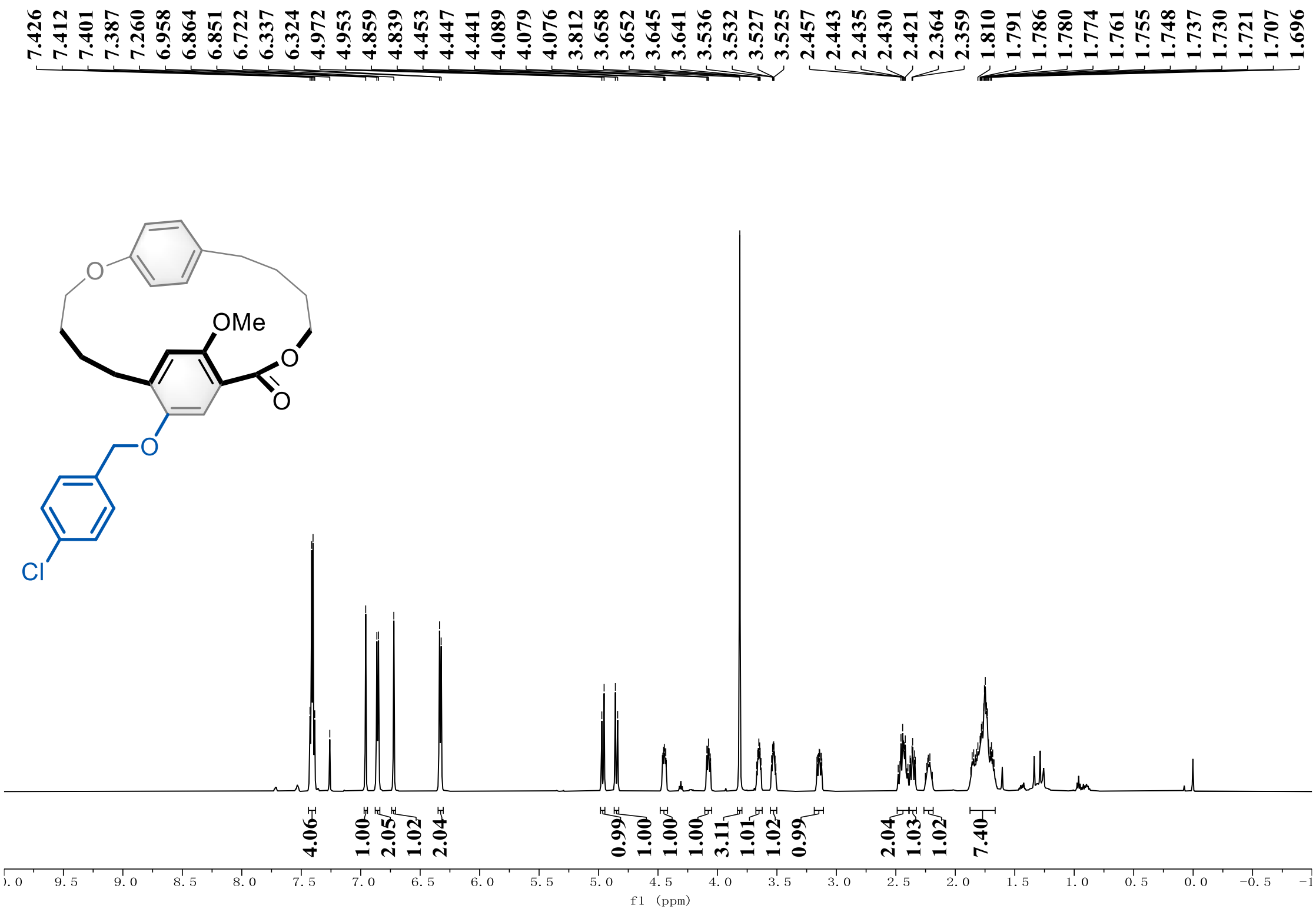

6, ¹³C NMR (151 MHz, $CDCl_3$)

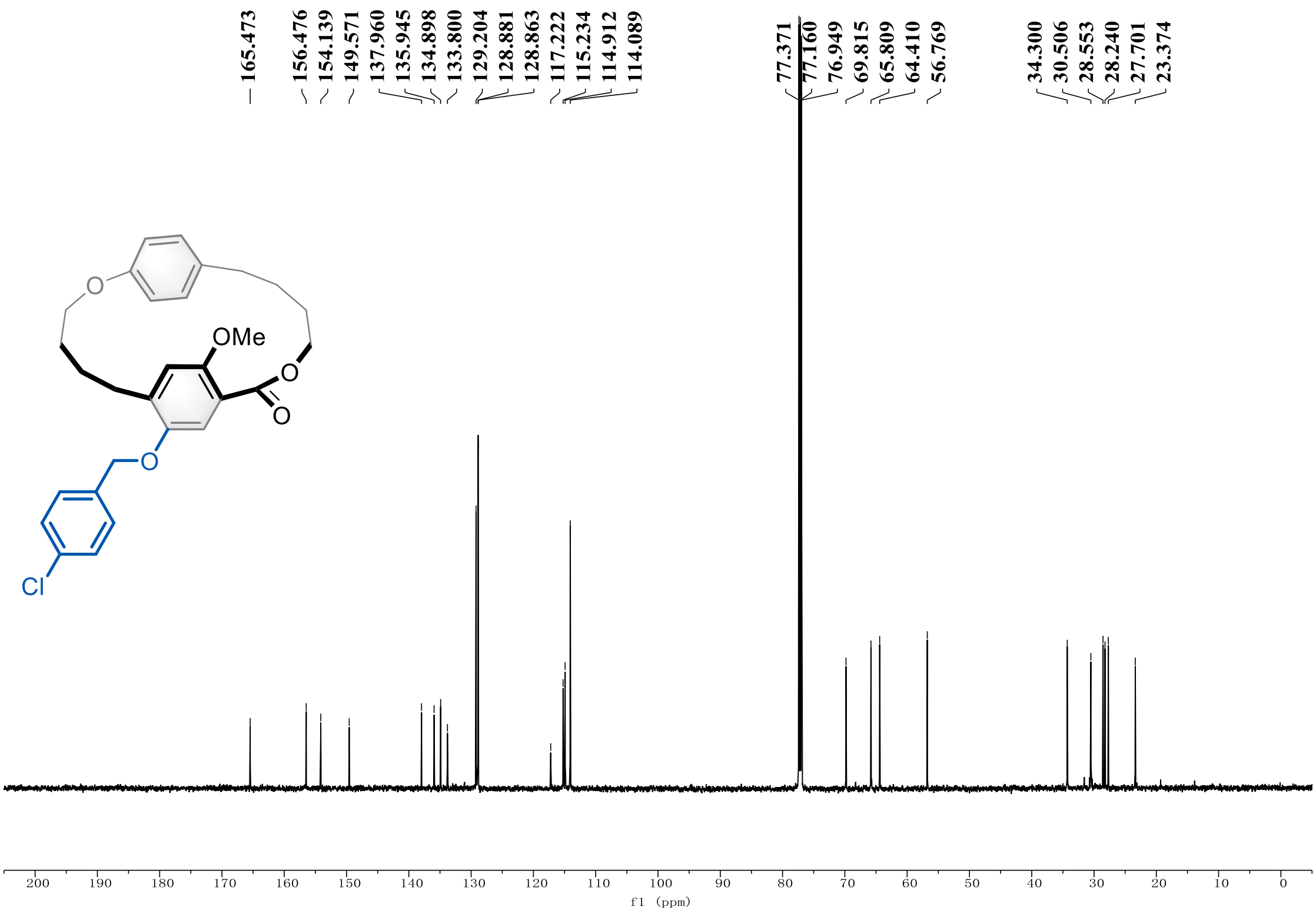

7, **^{1}H NMR** (600 MHz, $CDCl_3$)

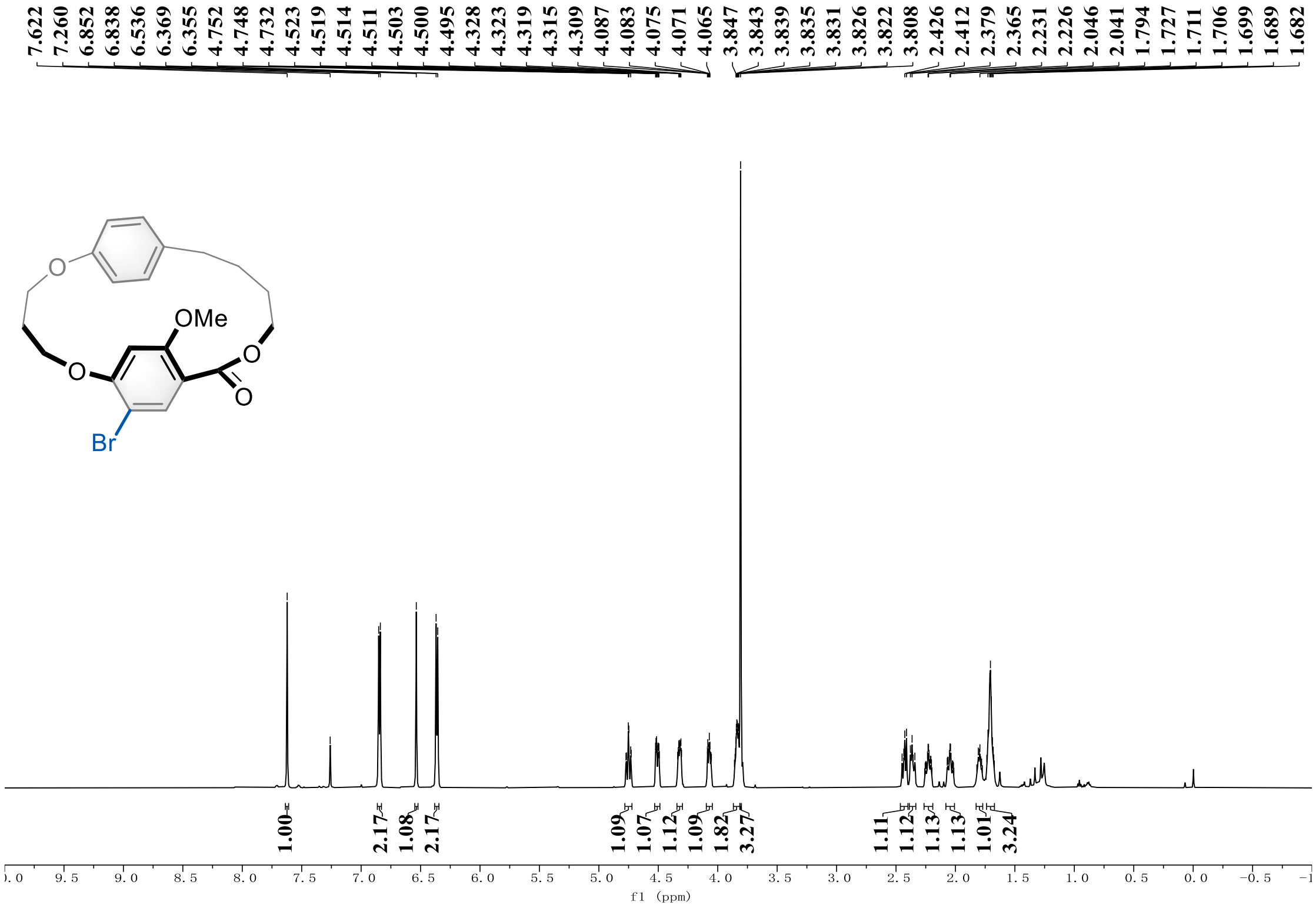

7, **^{13}C NMR** (151 MHz, $CDCl_3$)

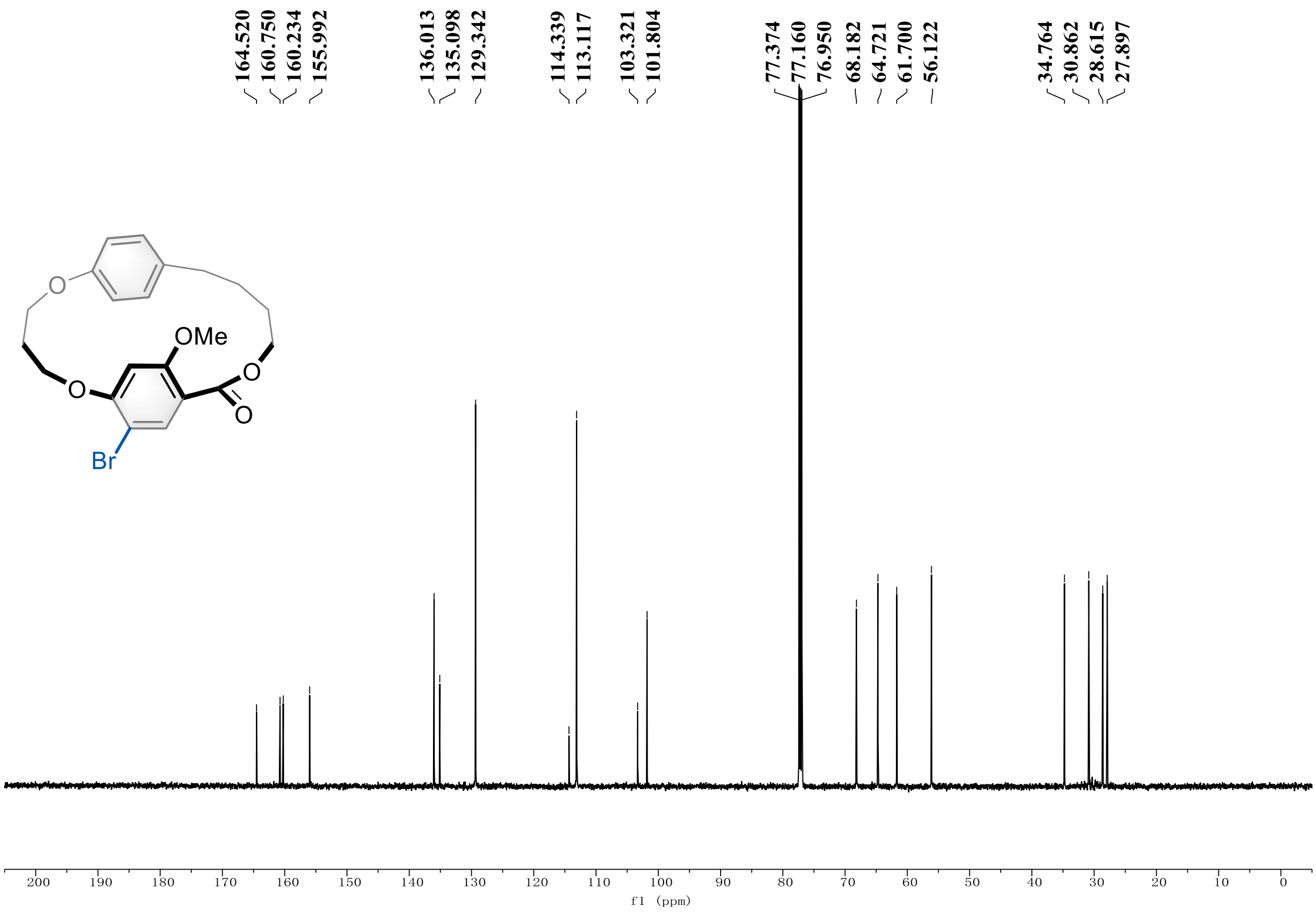

**8, **

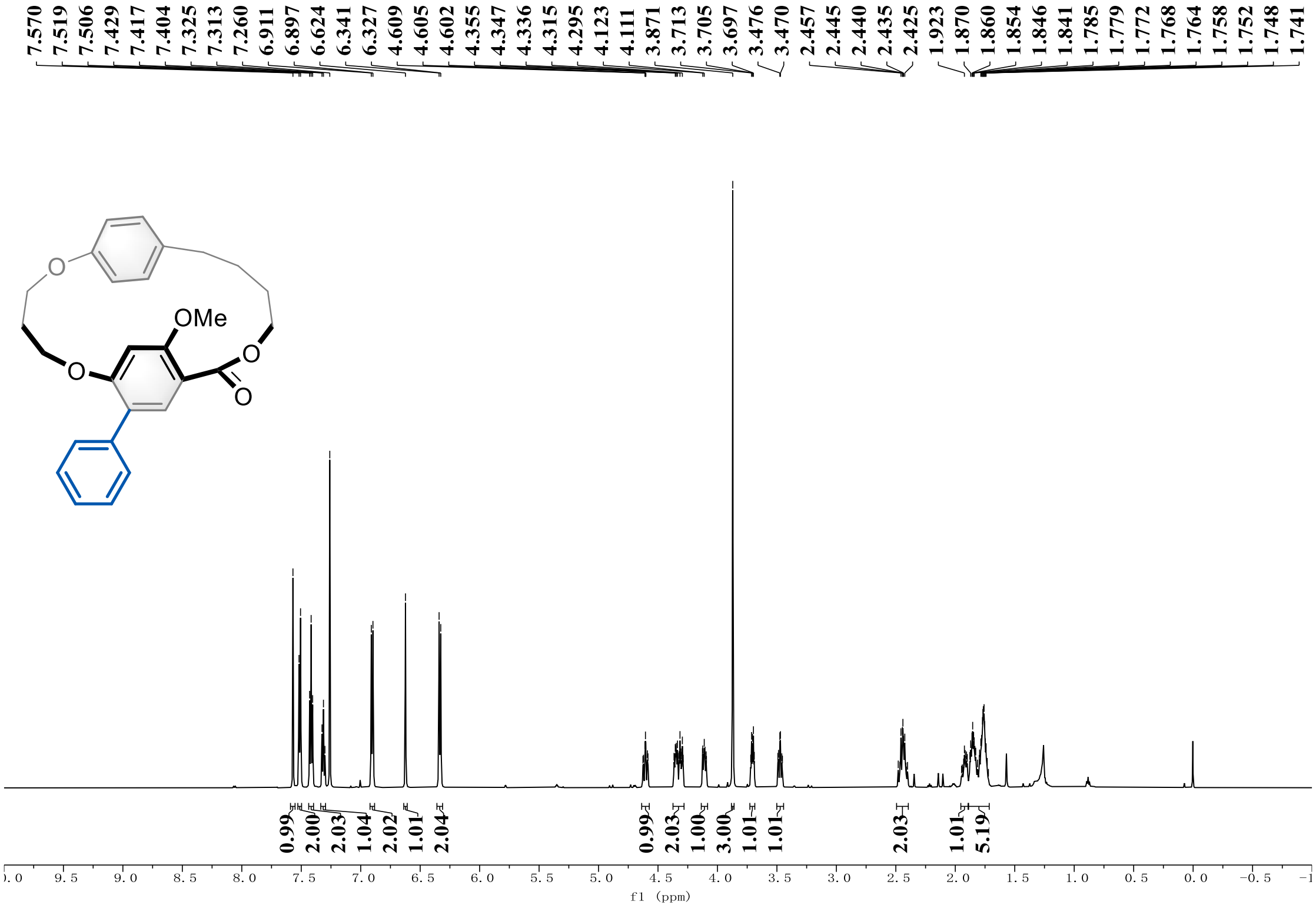

**8, **

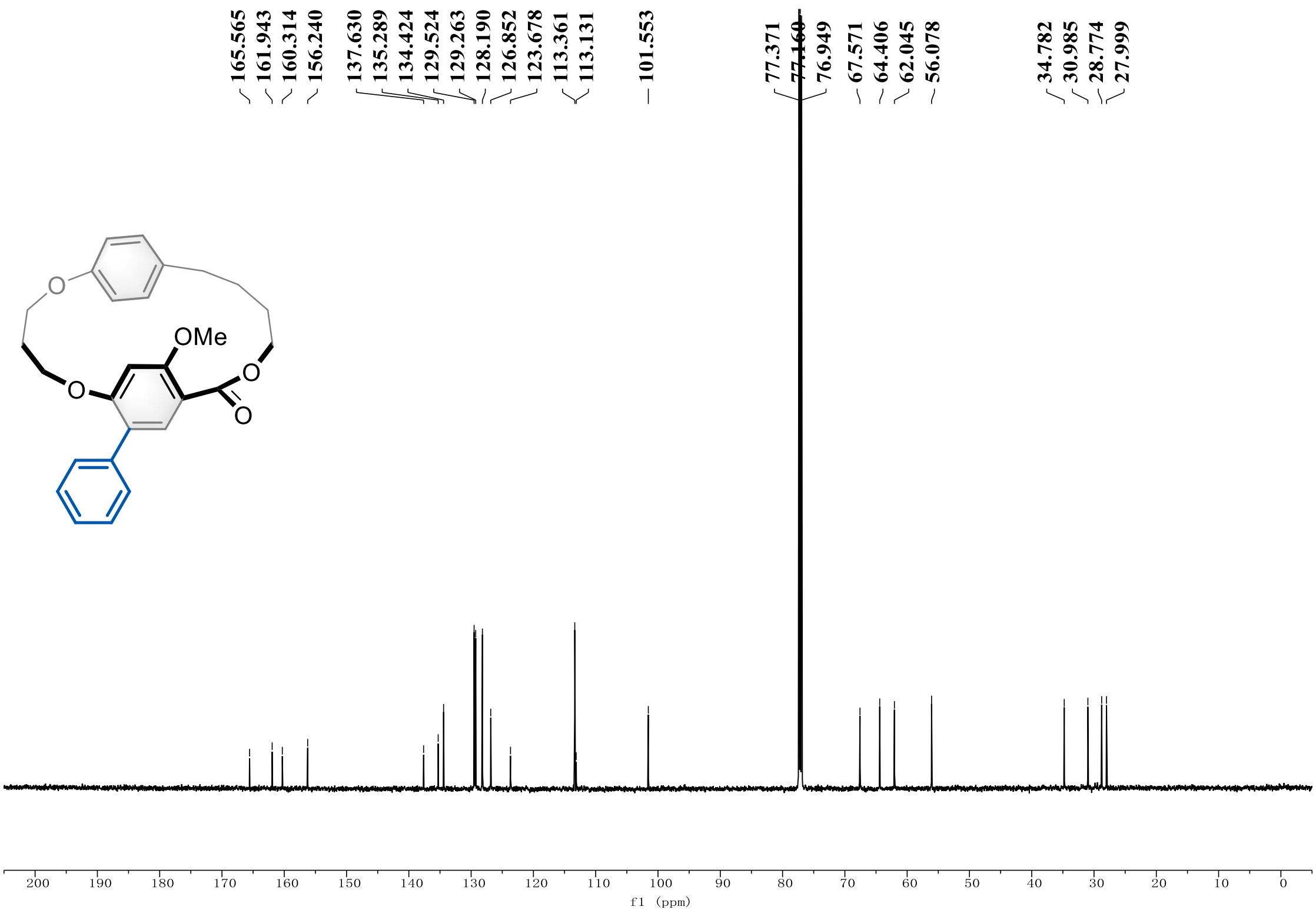

9, ^{1}H NMR (600 MHz, $CDCl_3$)

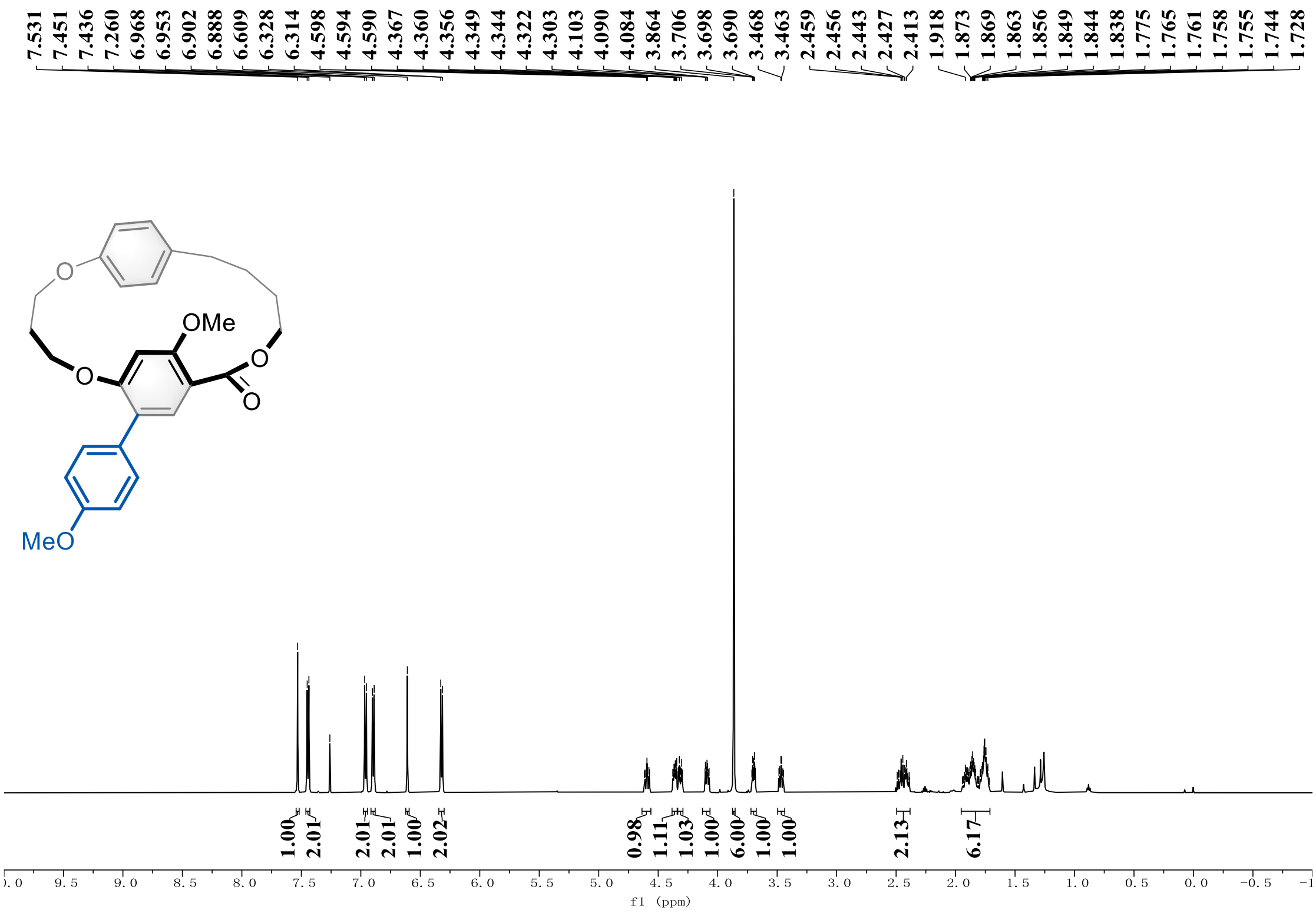

9, ^{13}C NMR (151 MHz, $CDCl_3$)

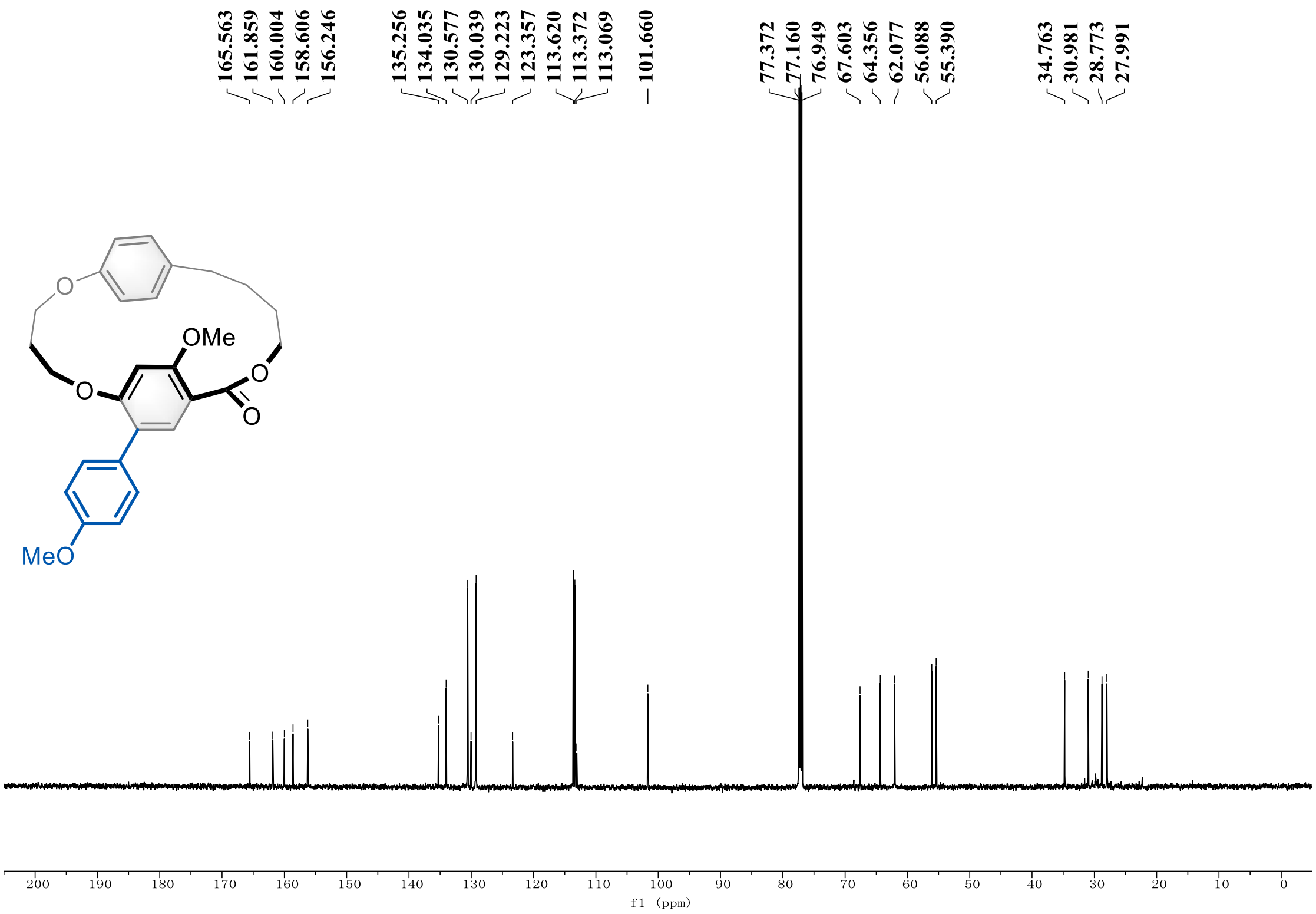

10, ^{1}H NMR (600 MHz, $CDCl_3$)

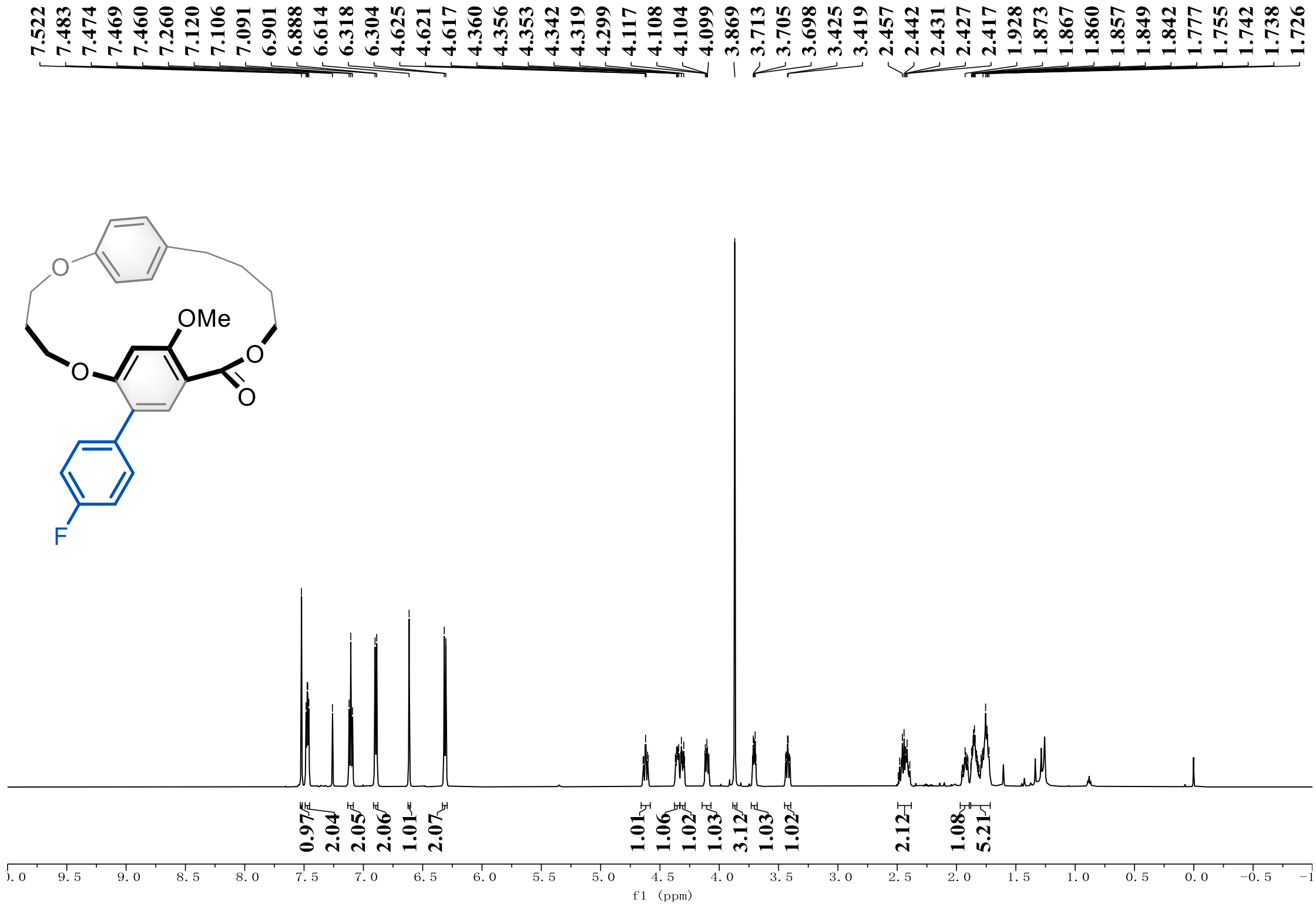

10, ^{13}C NMR (151 MHz, $CDCl_3$)

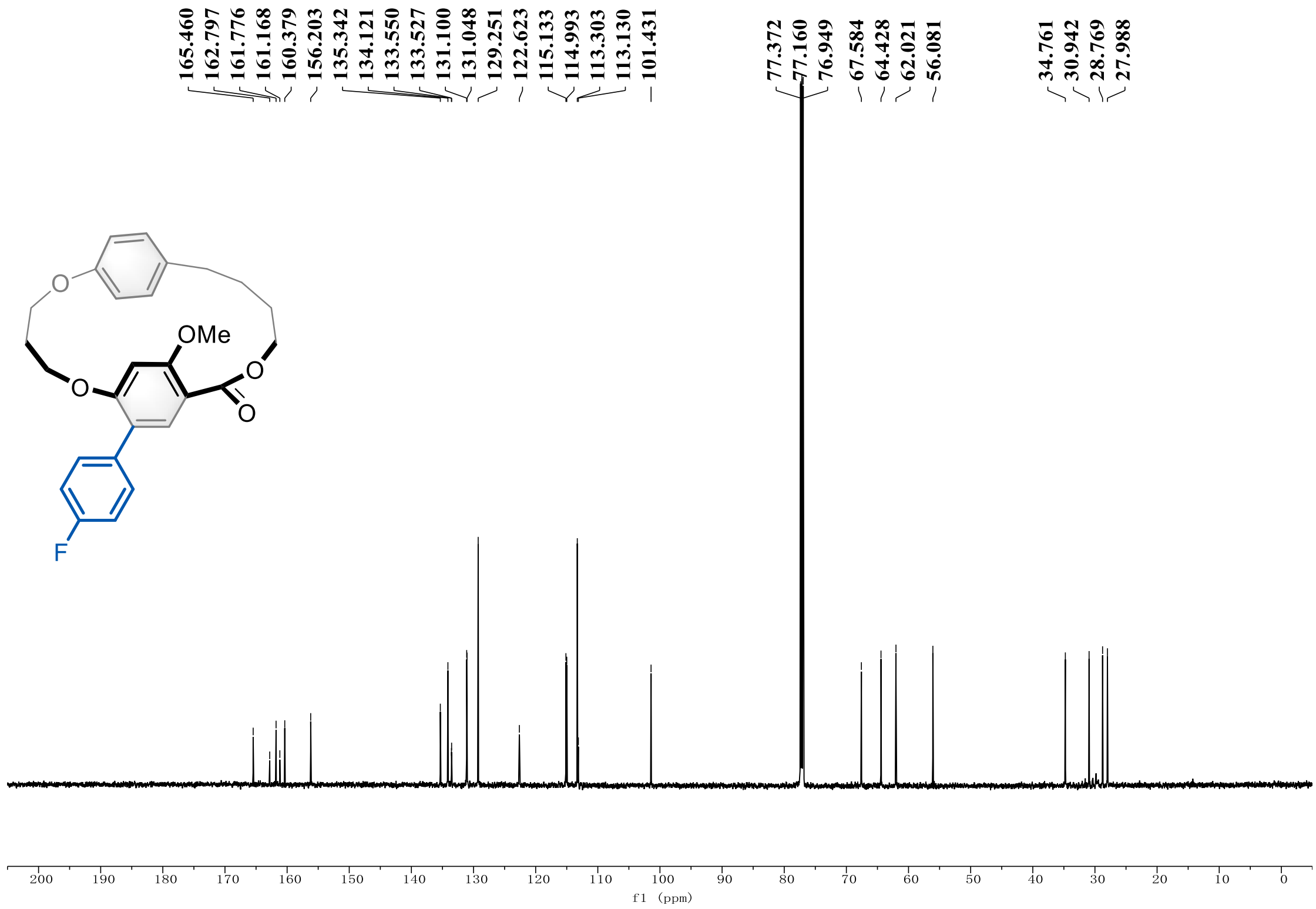

10, ^{19}F NMR (376 MHz, $CDCl_3$)

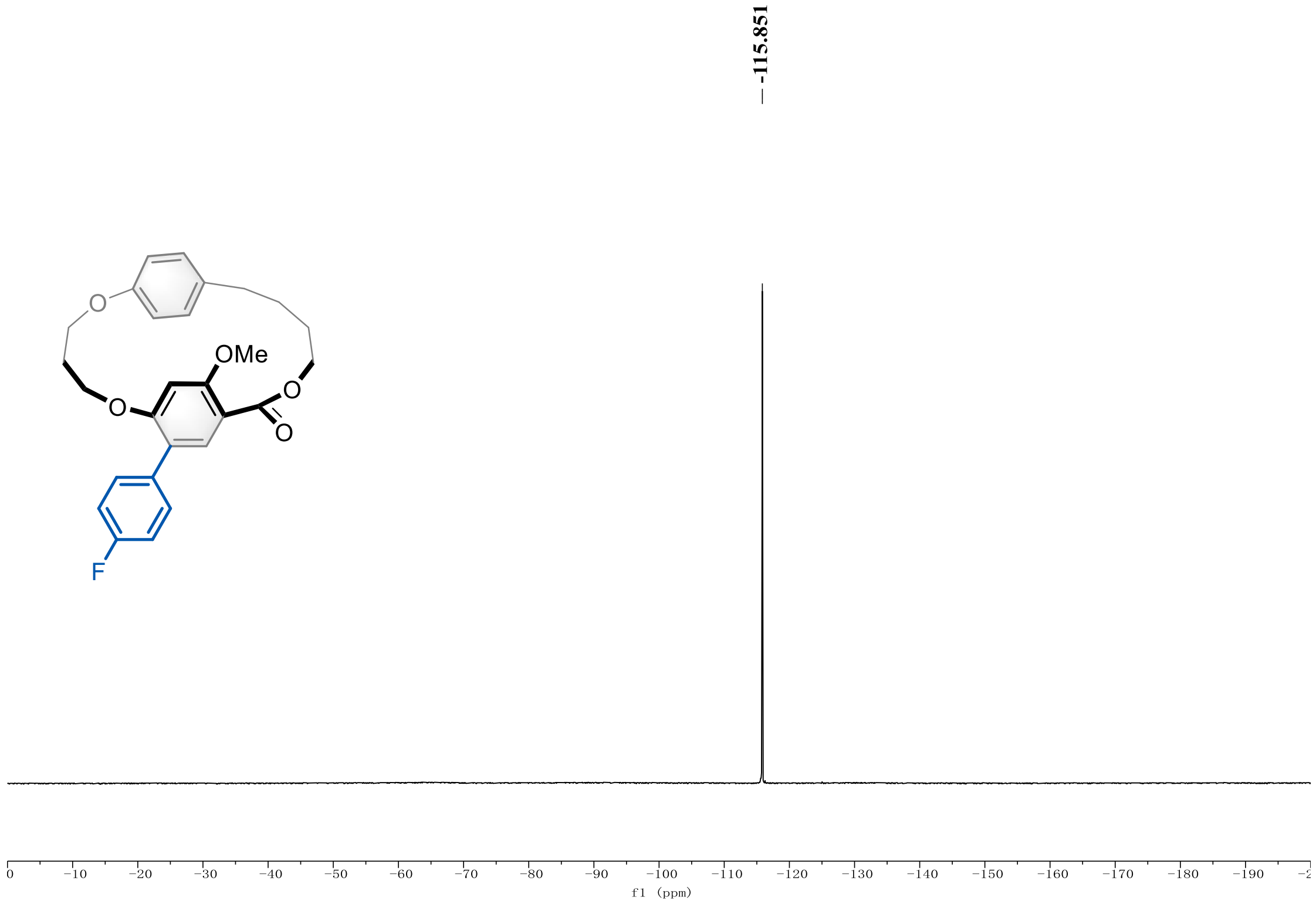

11, ^{1}H NMR (600 MHz, $CDCl_3$)

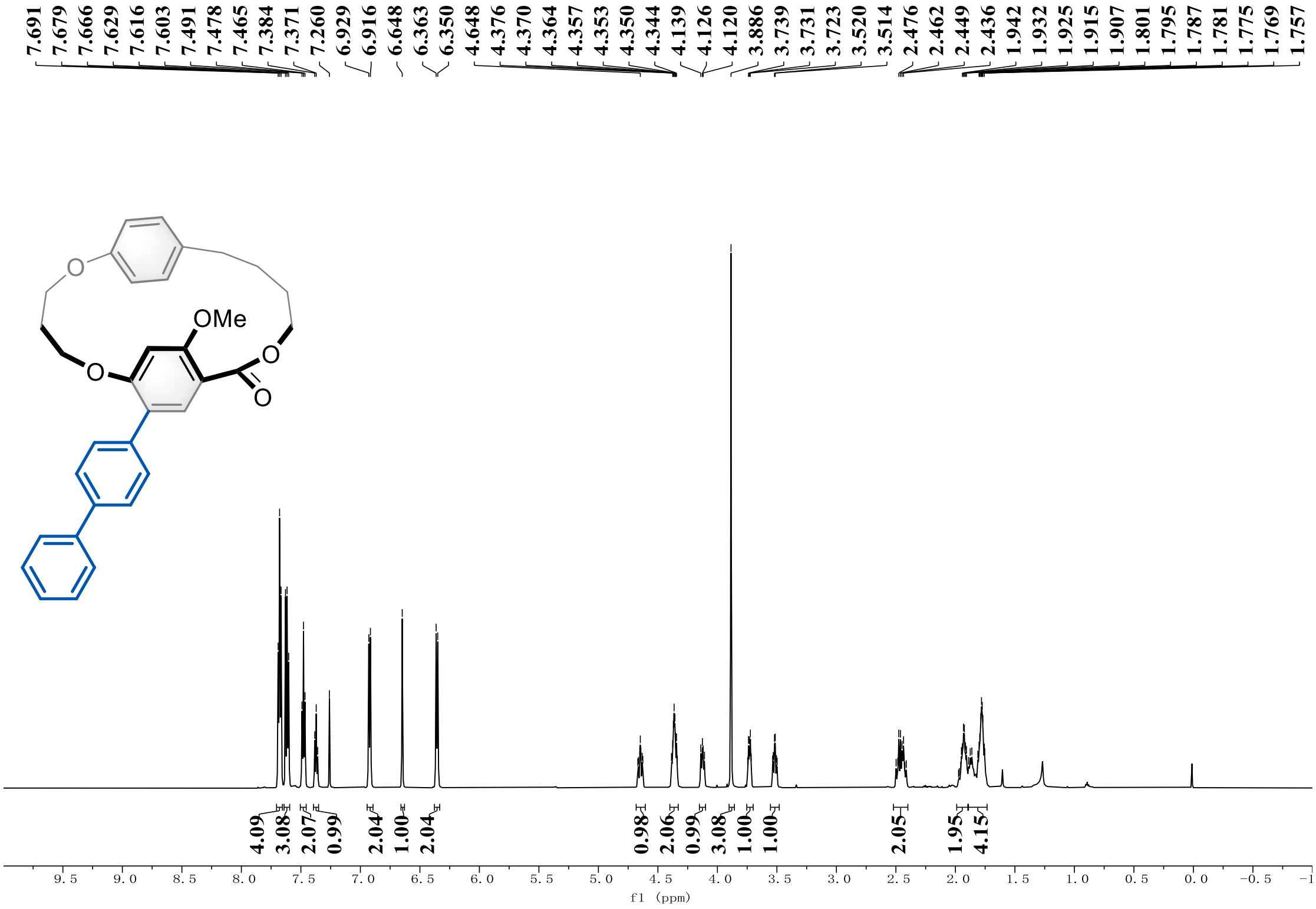

11, ^{13}C NMR (151 MHz, $CDCl_3$)

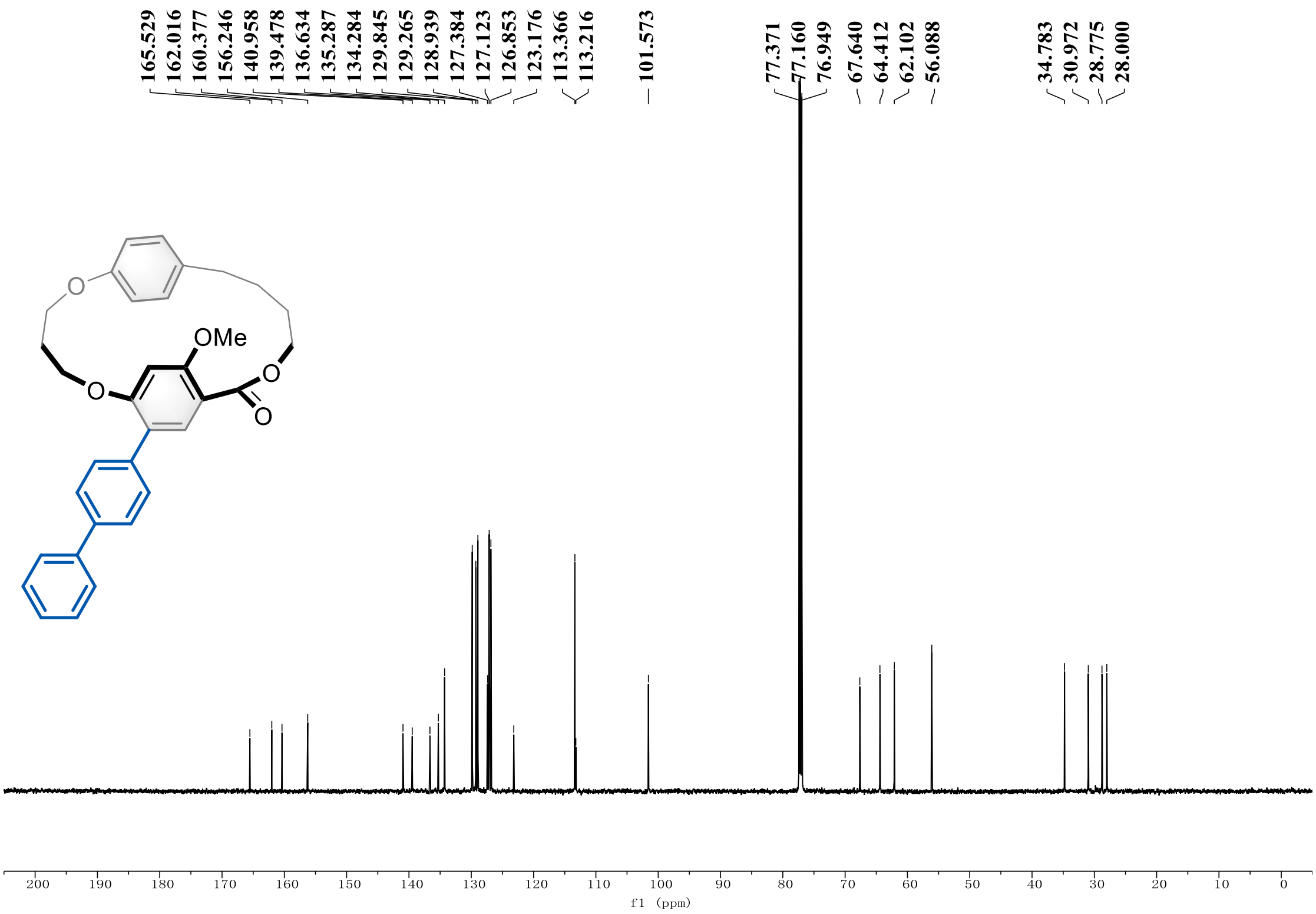

12, ^{1}H NMR (600 MHz, $CDCl_3$)

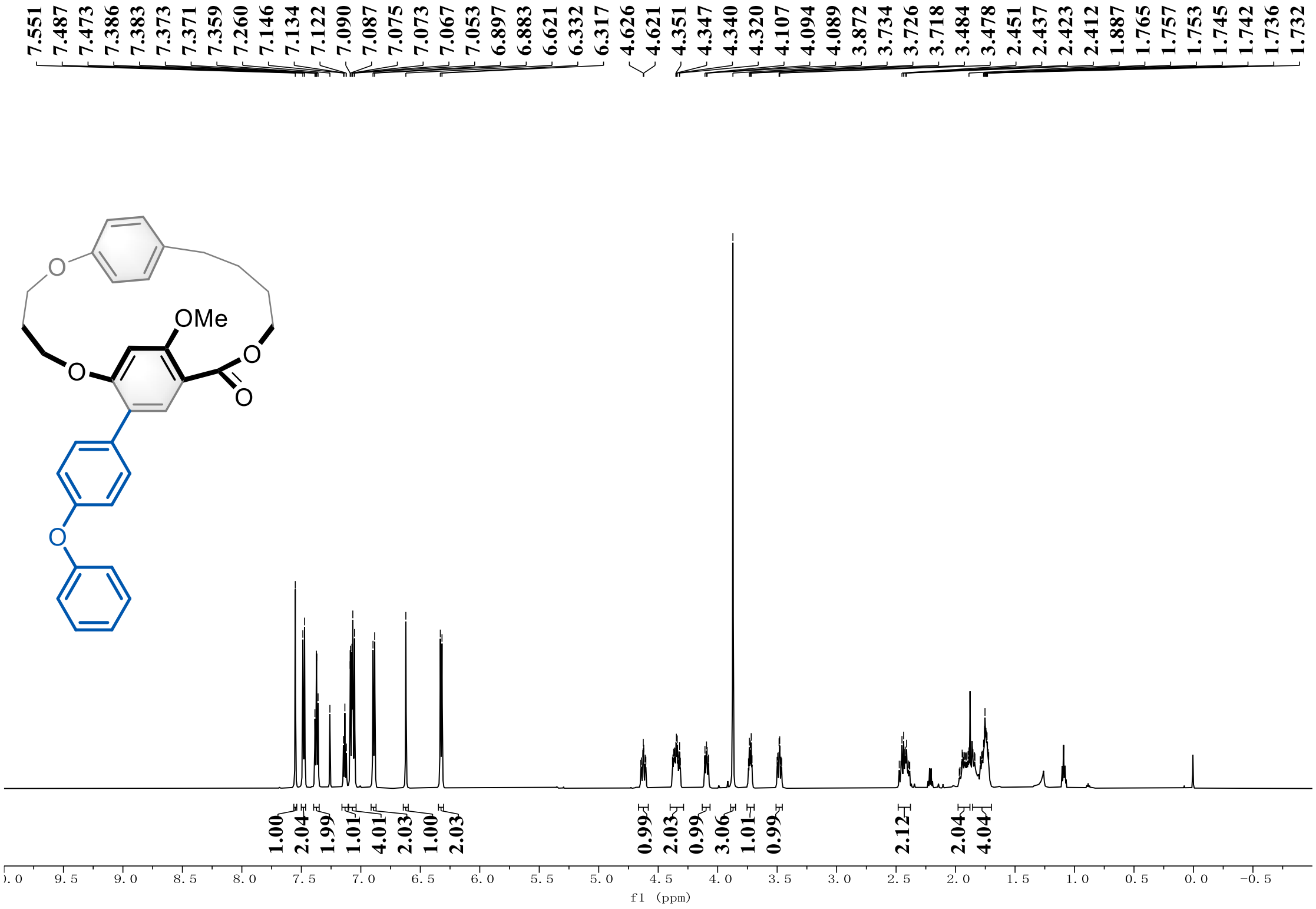

12, ^{13}C NMR (151 MHz, $CDCl_3$)

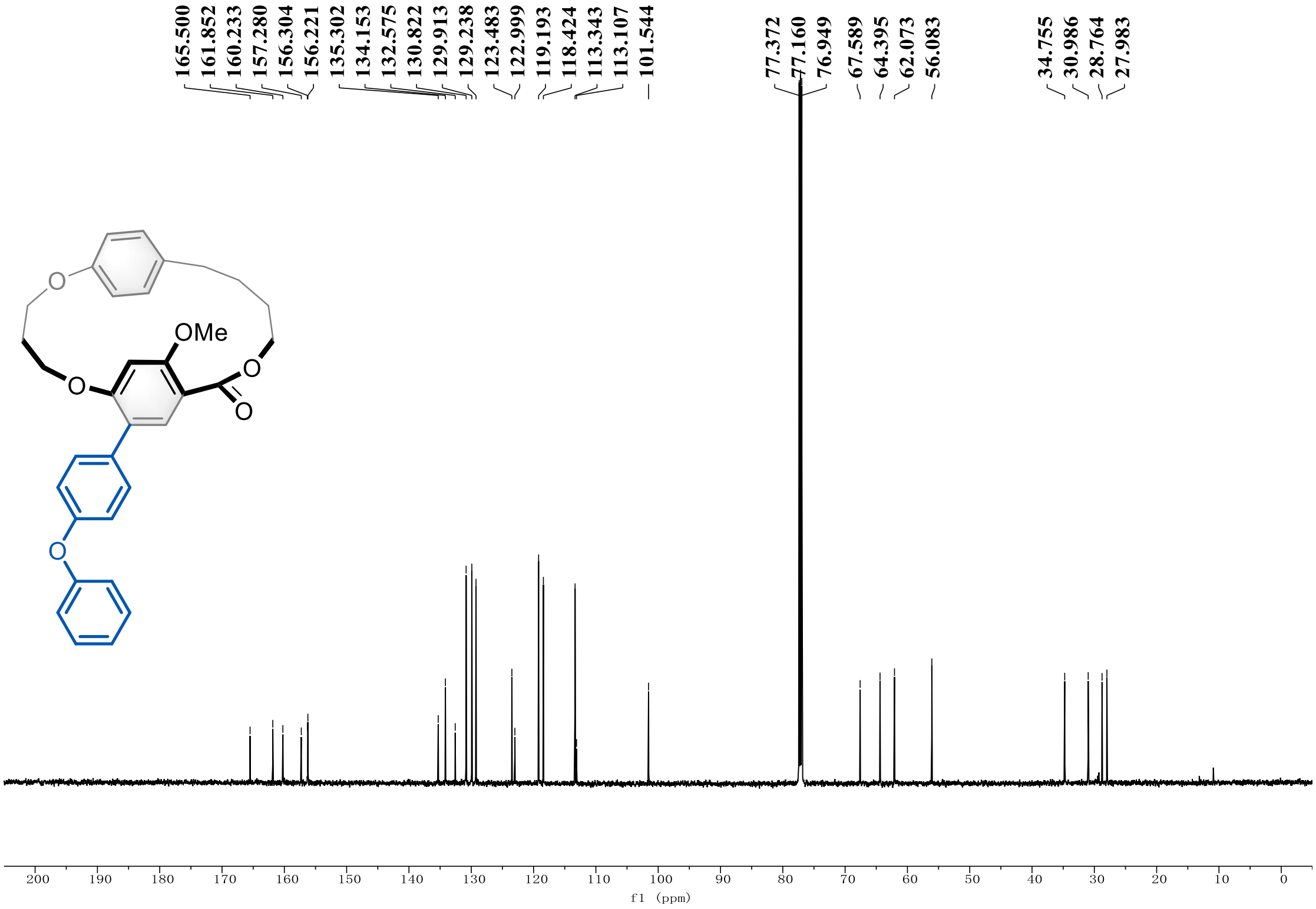

13, ^{1}H NMR (600 MHz, $CDCl_3$)

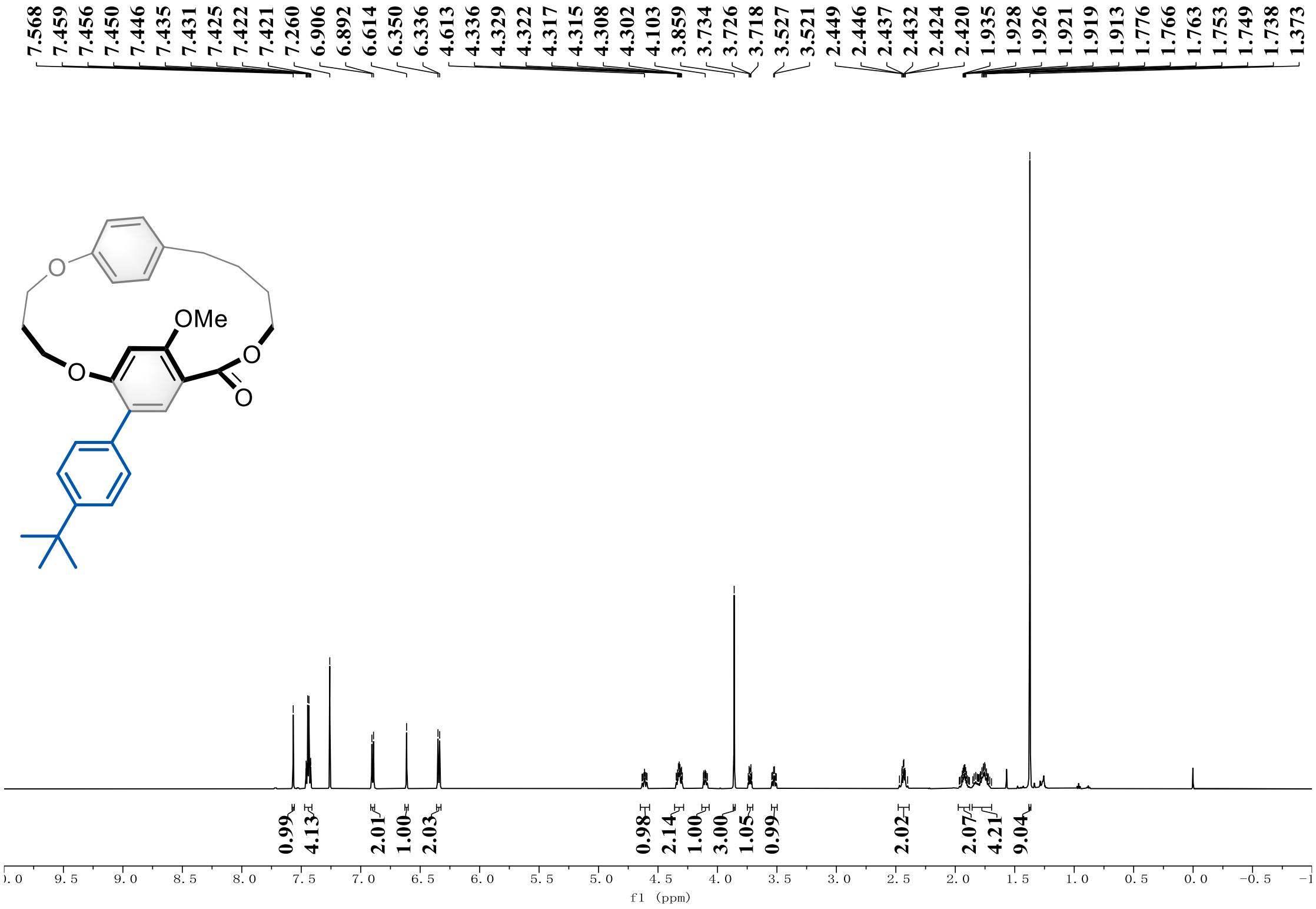

13, ^{13}C NMR (151 MHz, $CDCl_3$)

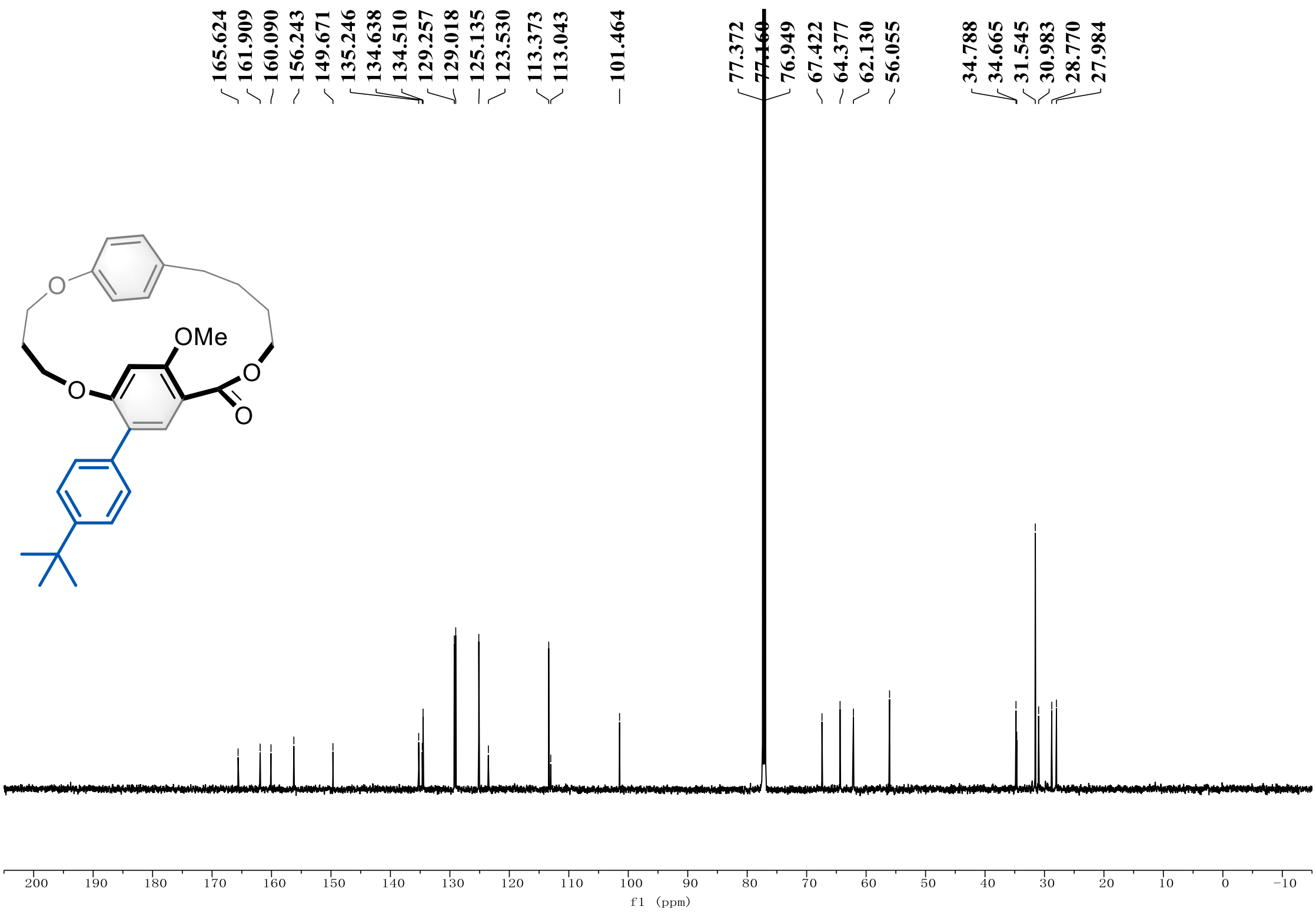

14, ^{1}H NMR (600 MHz, $CDCl_3$)

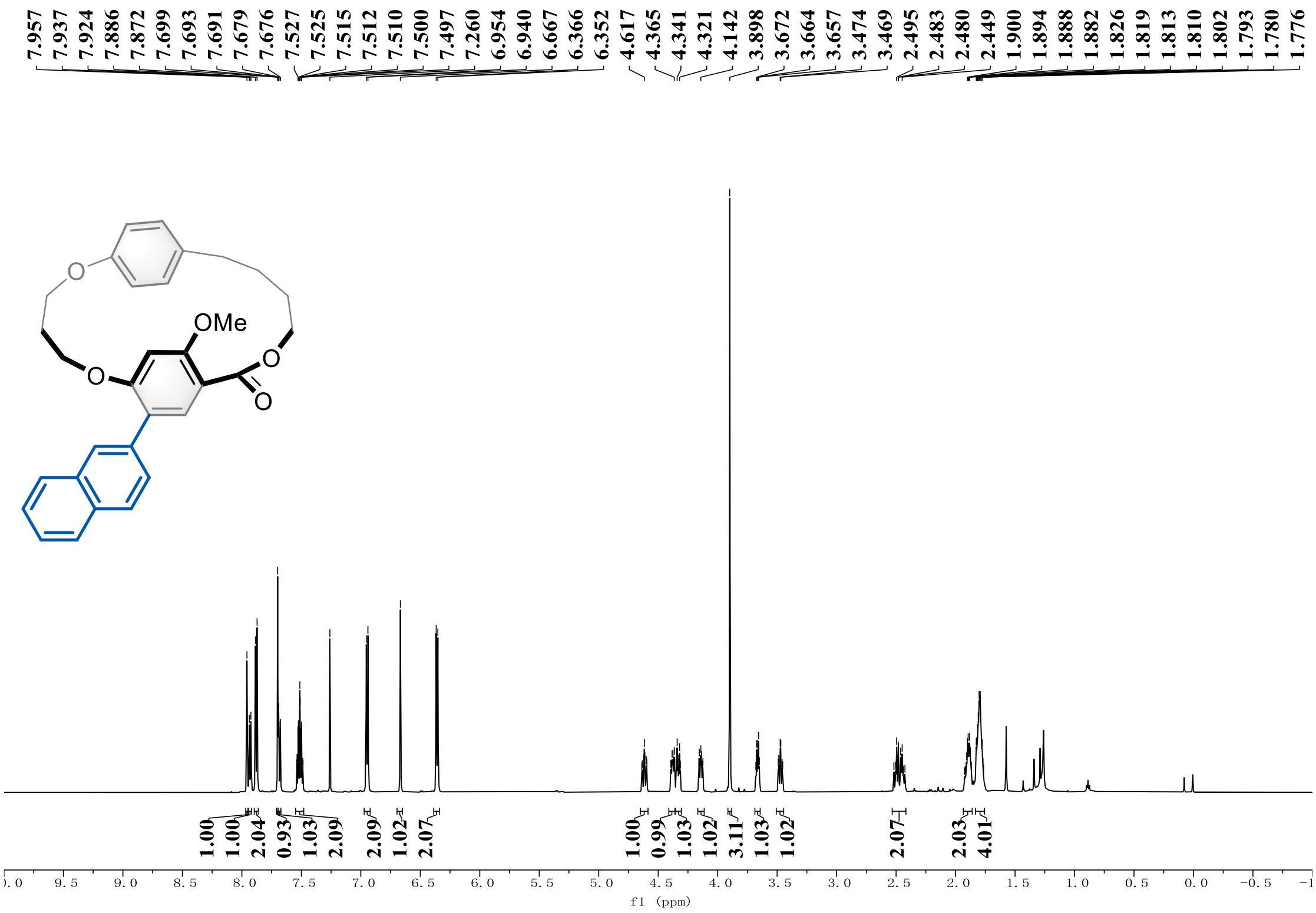

14, ^{13}C NMR (151 MHz, $CDCl_3$)

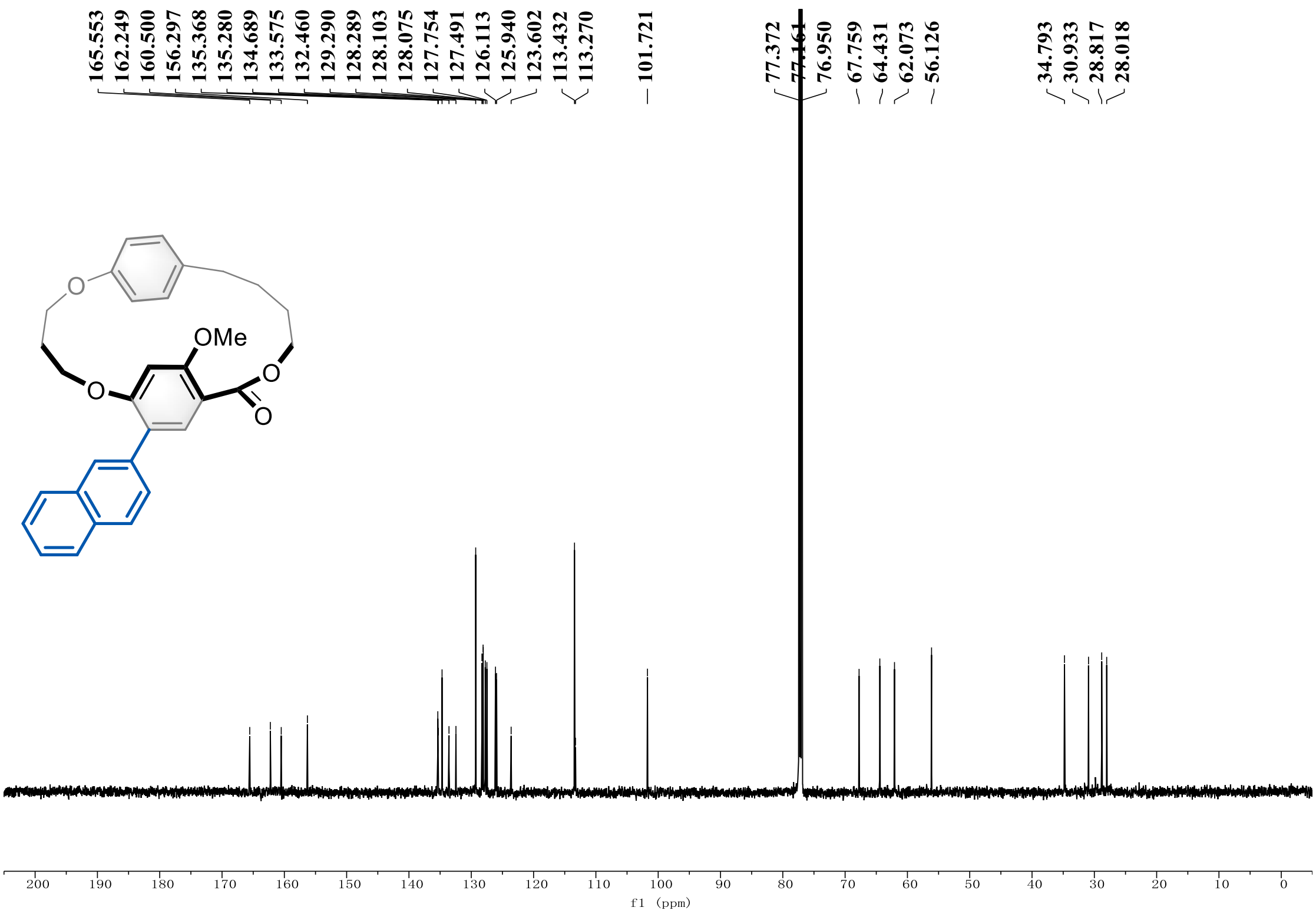

15, ¹H NMR (600 MHz, $CDCl_3$)

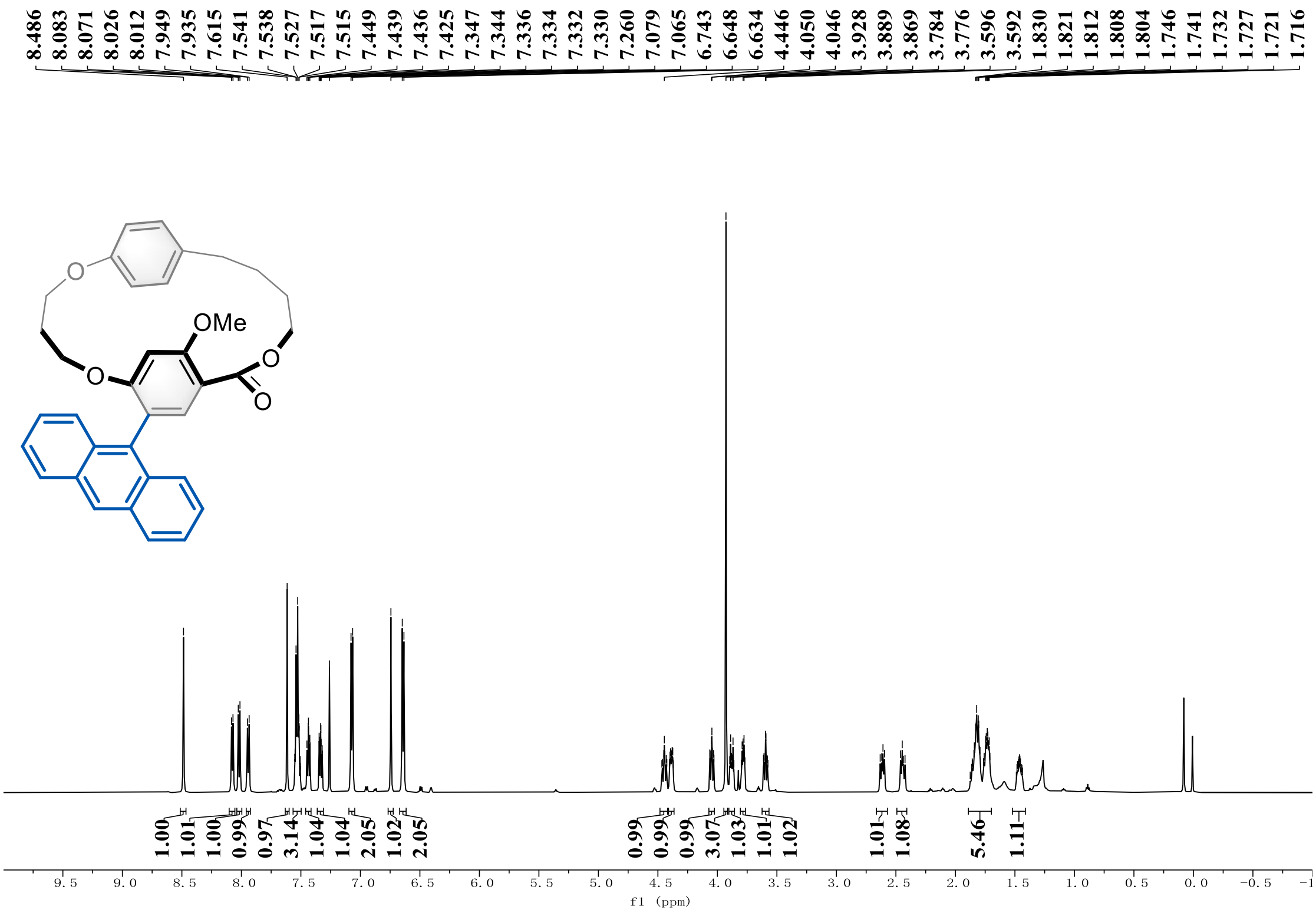

15, ¹³C NMR (151 MHz, $CDCl_3$)

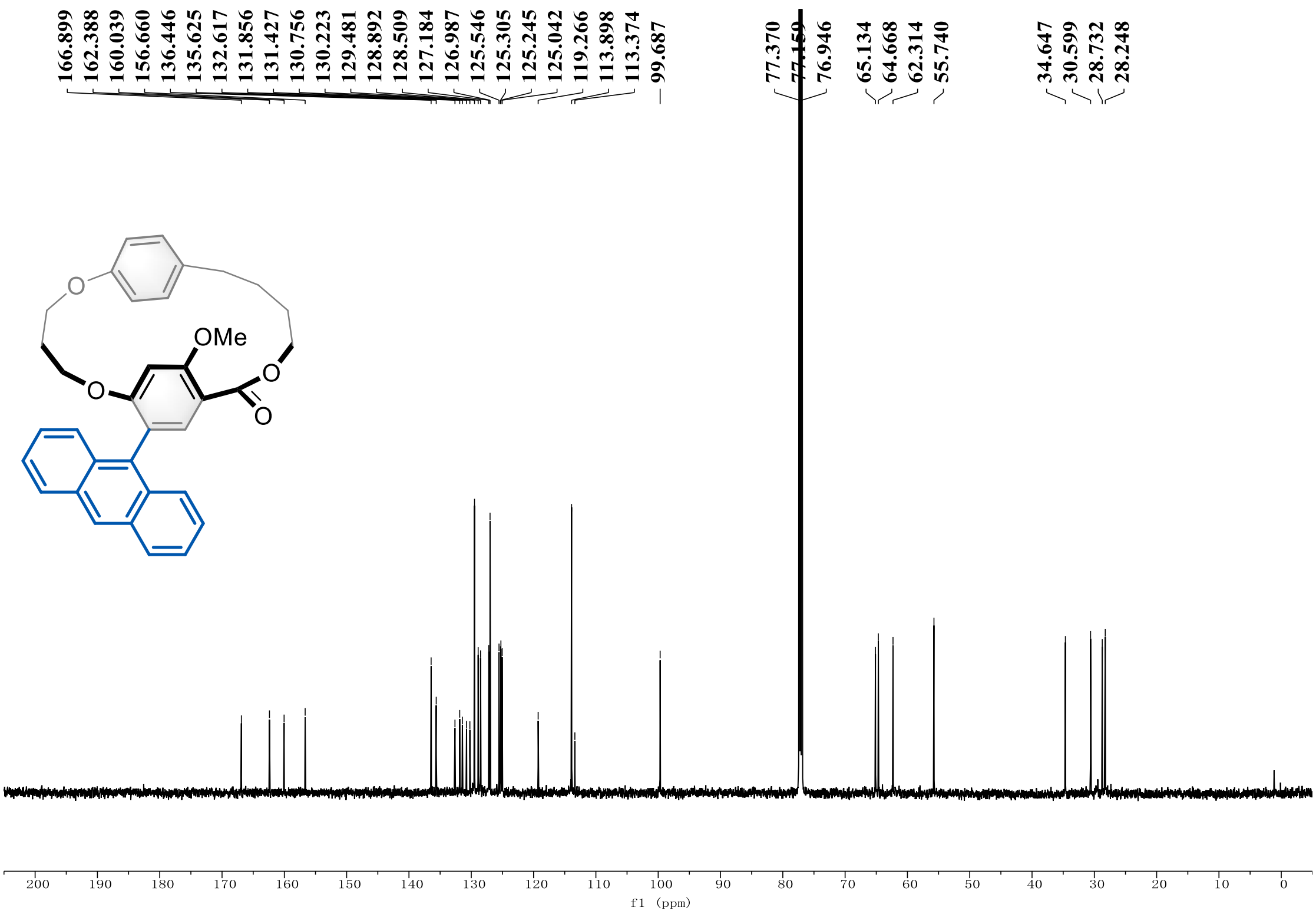

16, ¹H NMR (600 MHz, CDCl$_3$)

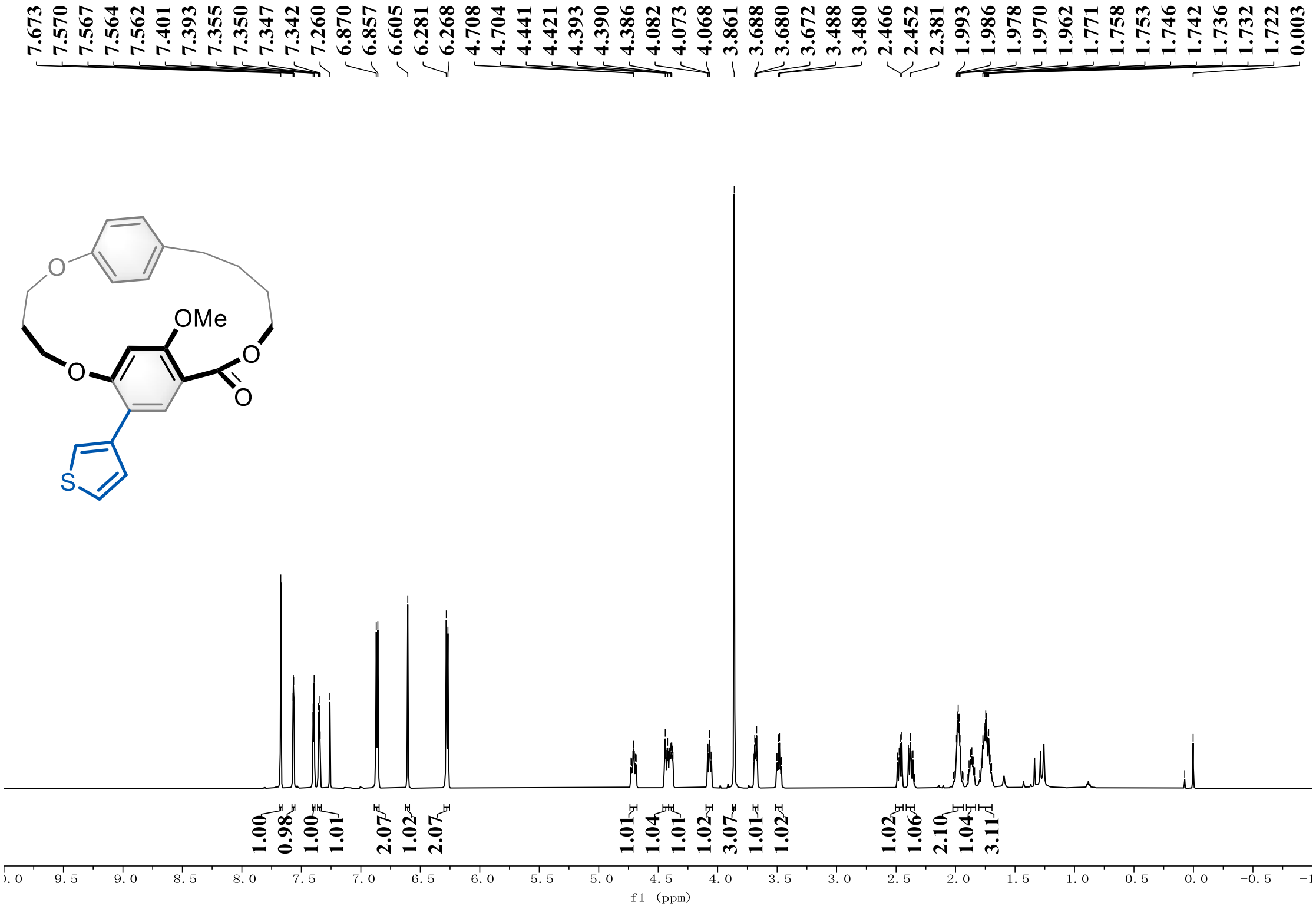

16, ¹³C NMR (151 MHz, CDCl$_3$)

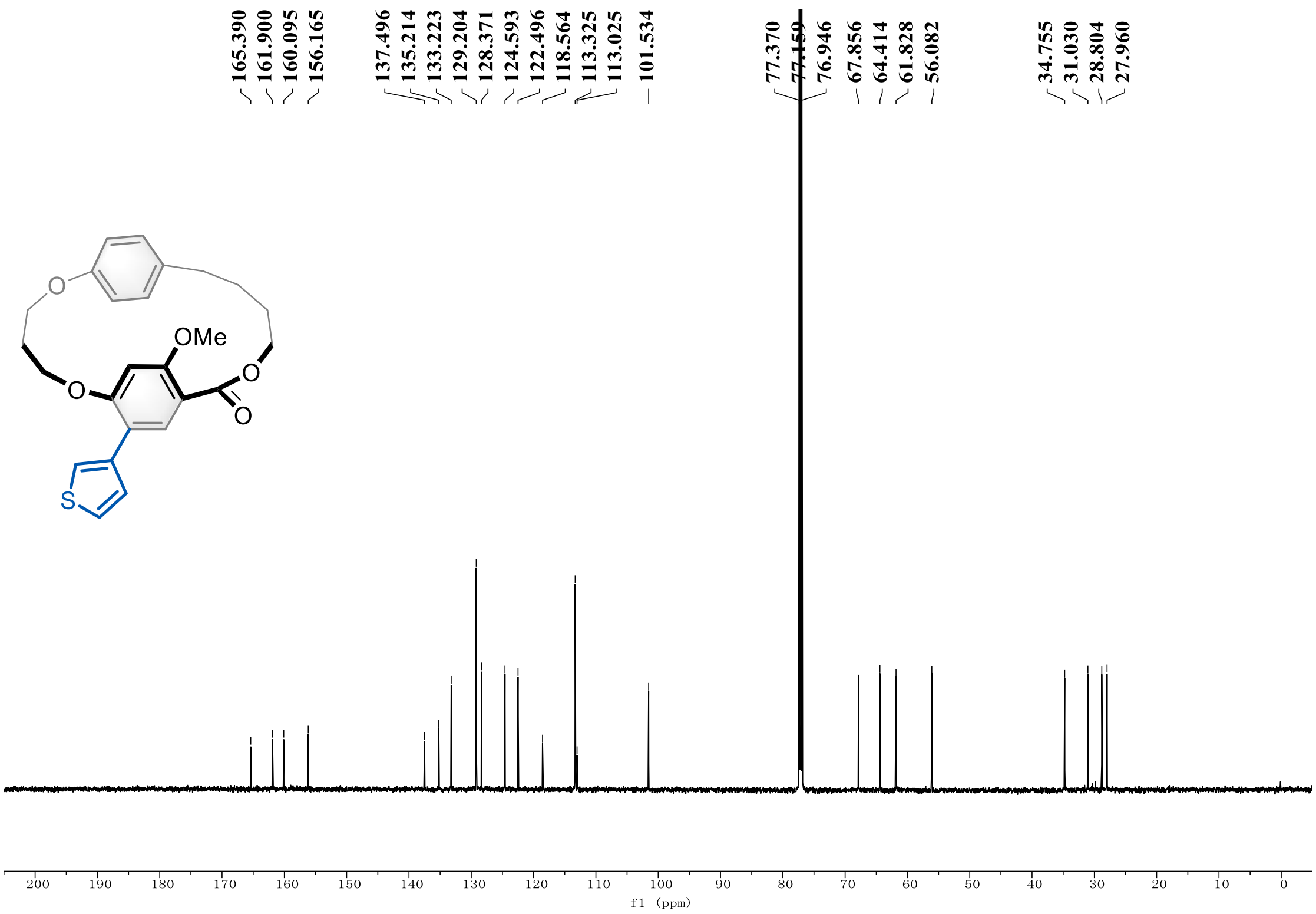

17, ^{1}H NMR (600 MHz, $CDCl_3$)

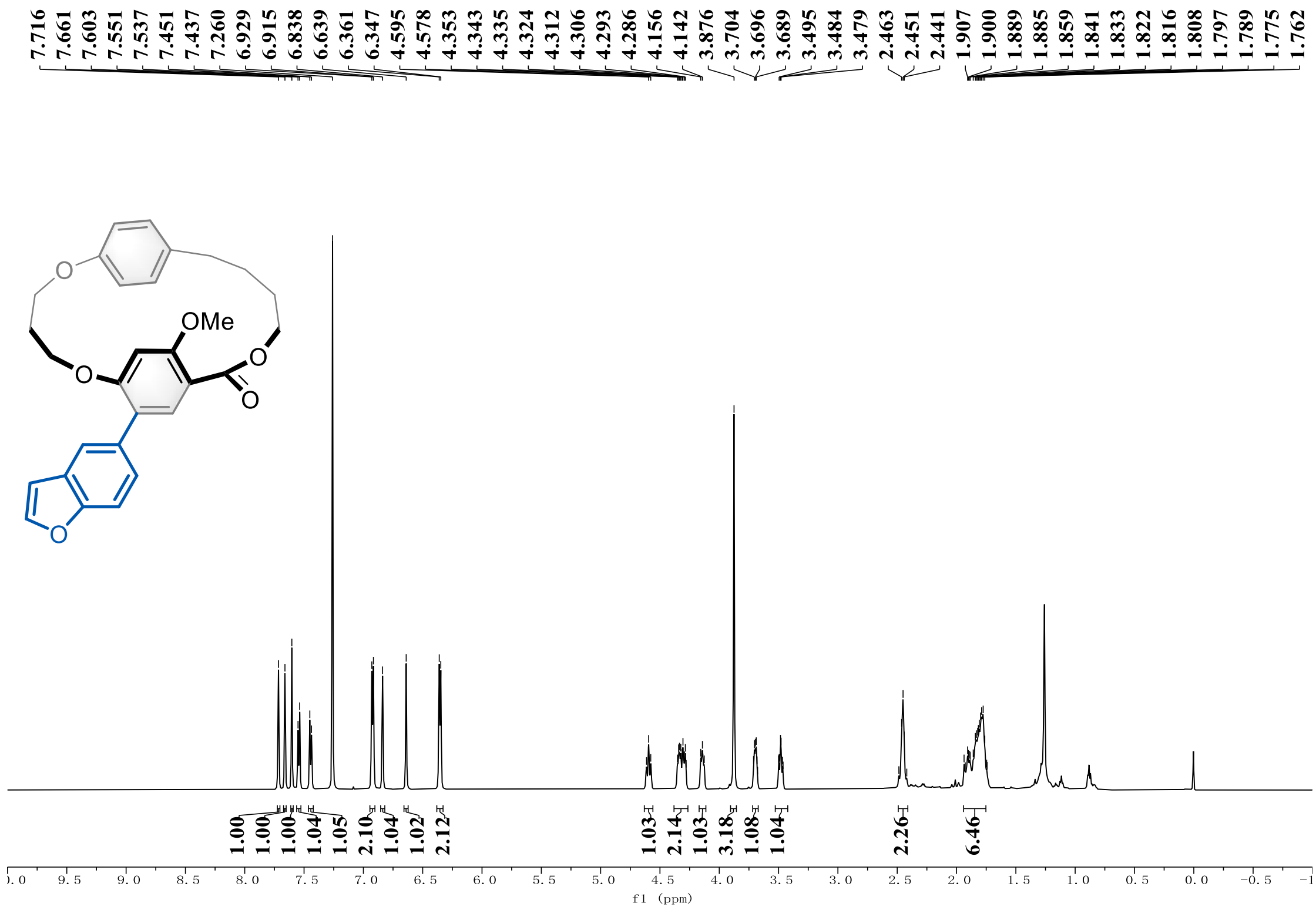

17, ^{13}C NMR (151 MHz, $CDCl_3$)

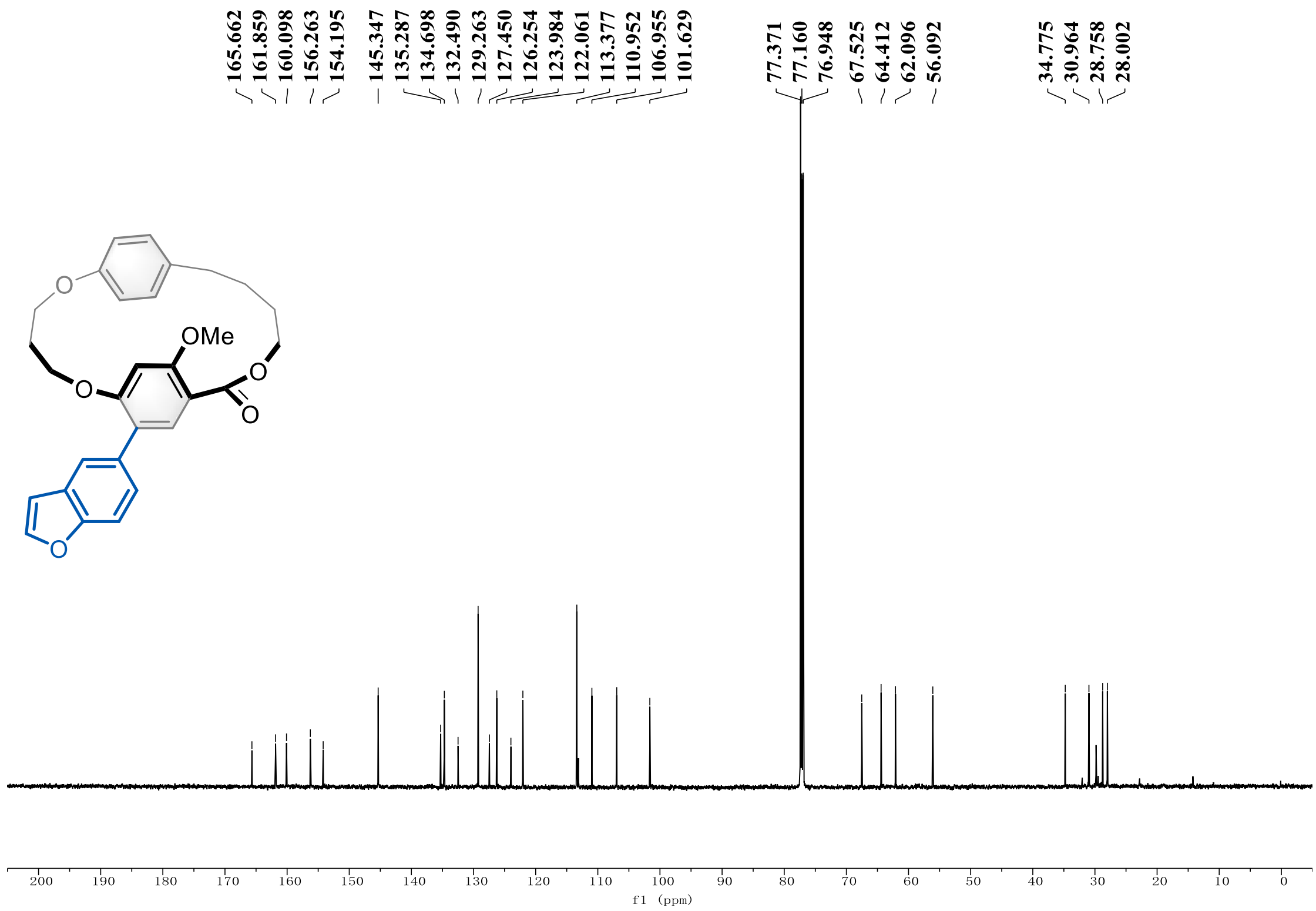

18, ^{1}H NMR (600 MHz, $CDCl_3$)

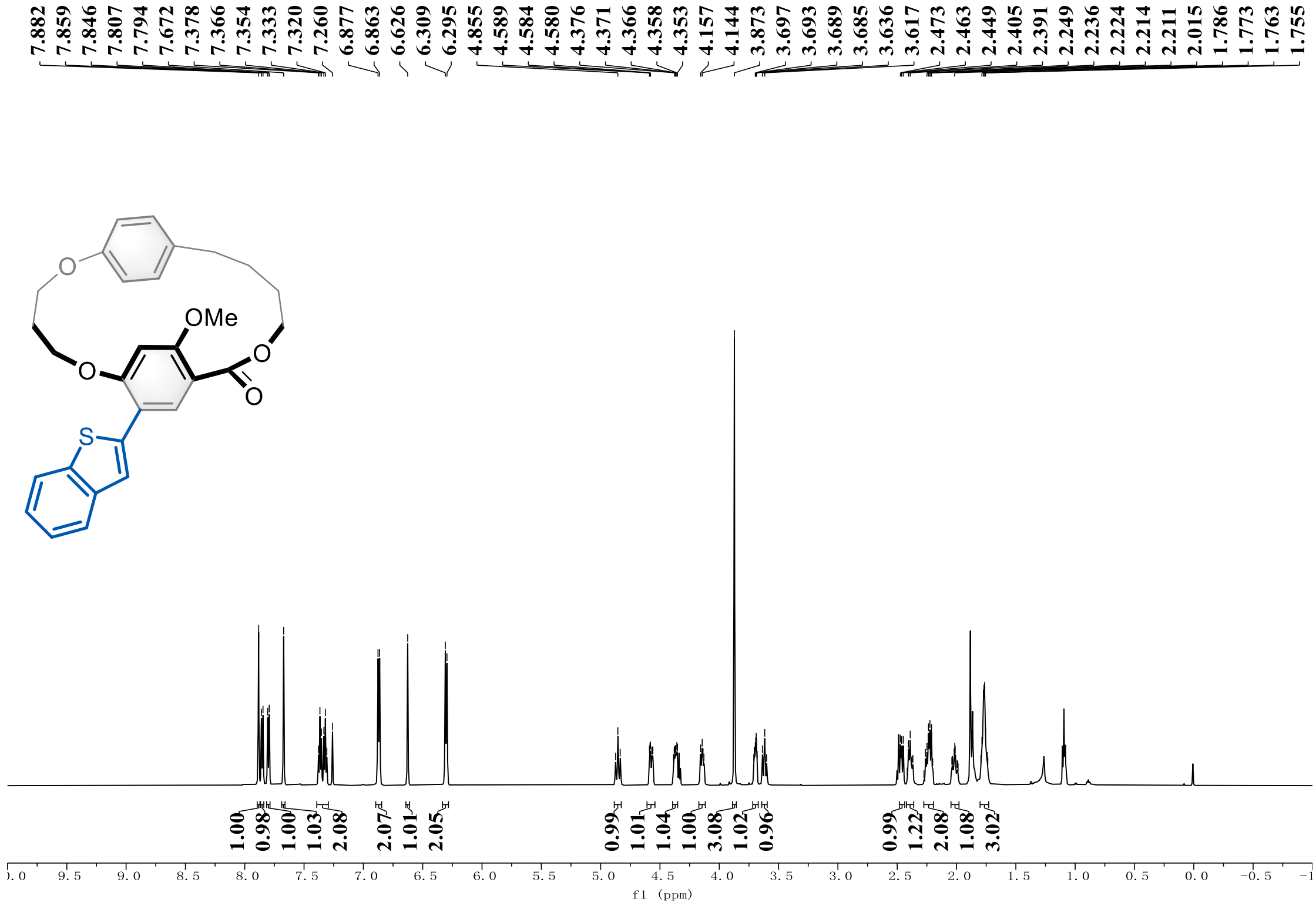

18, ^{13}C NMR (151 MHz, $CDCl_3$)

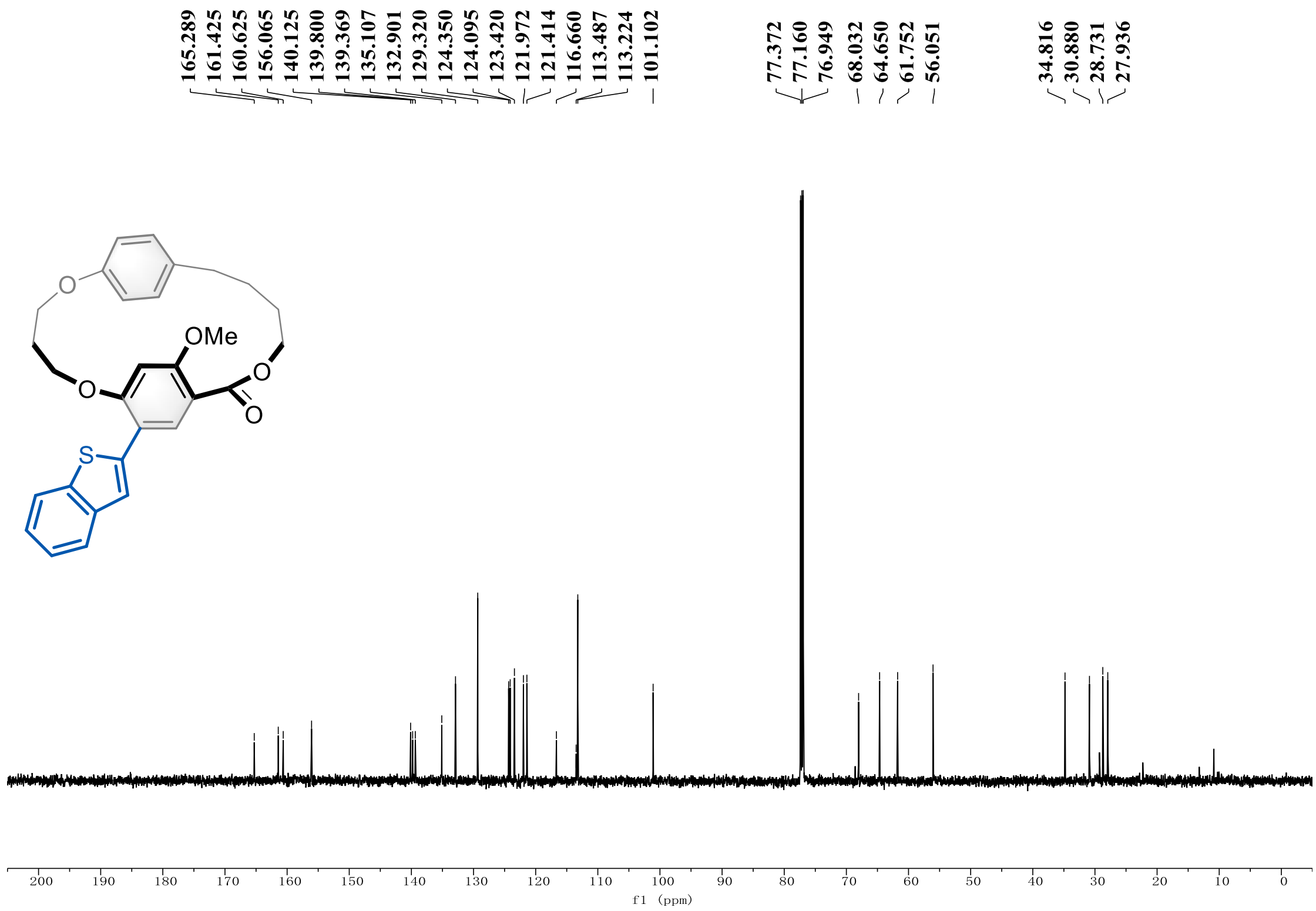

19, ¹H NMR (600 MHz, $CDCl_3$)

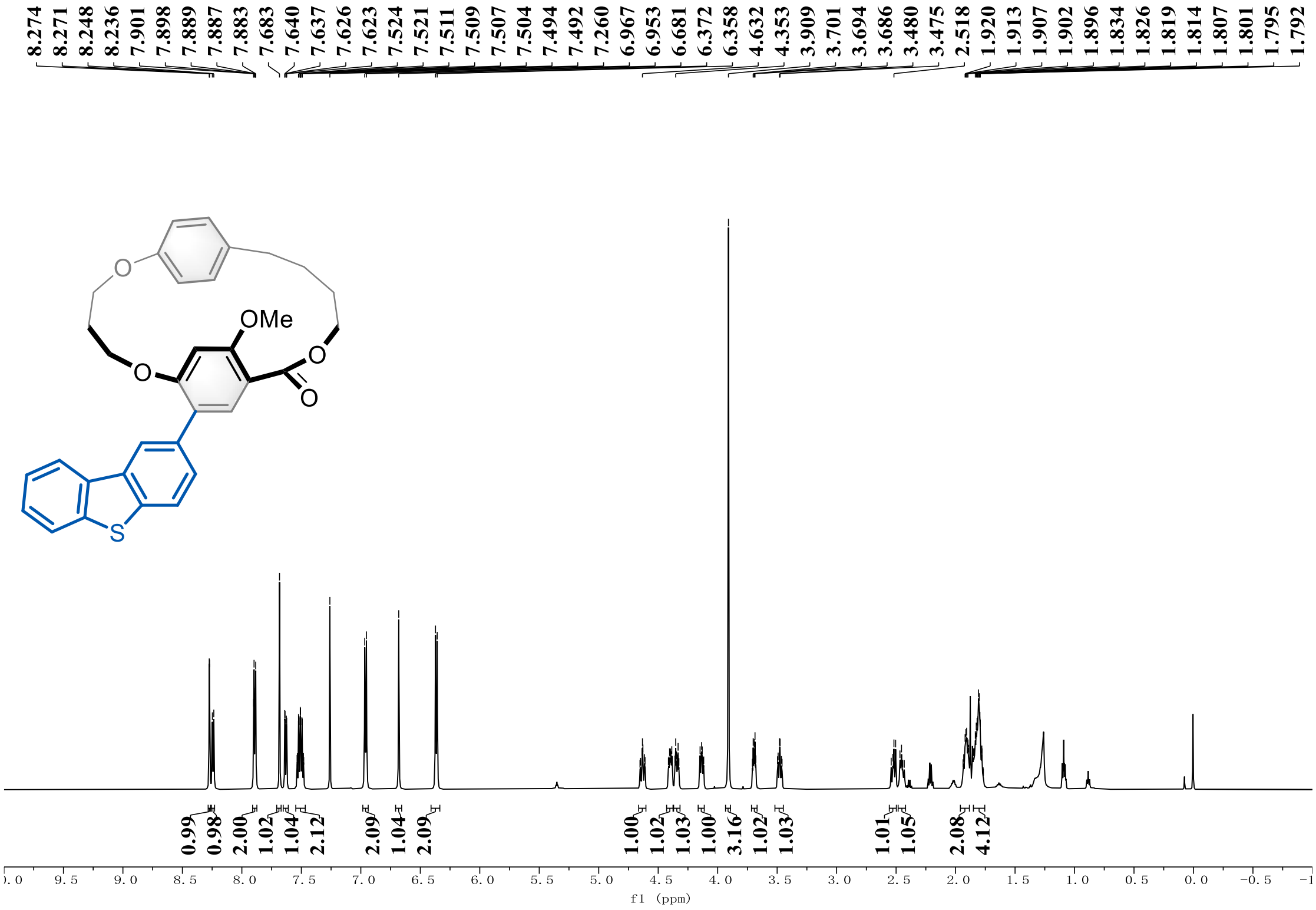

19, ¹³C NMR (151 MHz, $CDCl_3$)

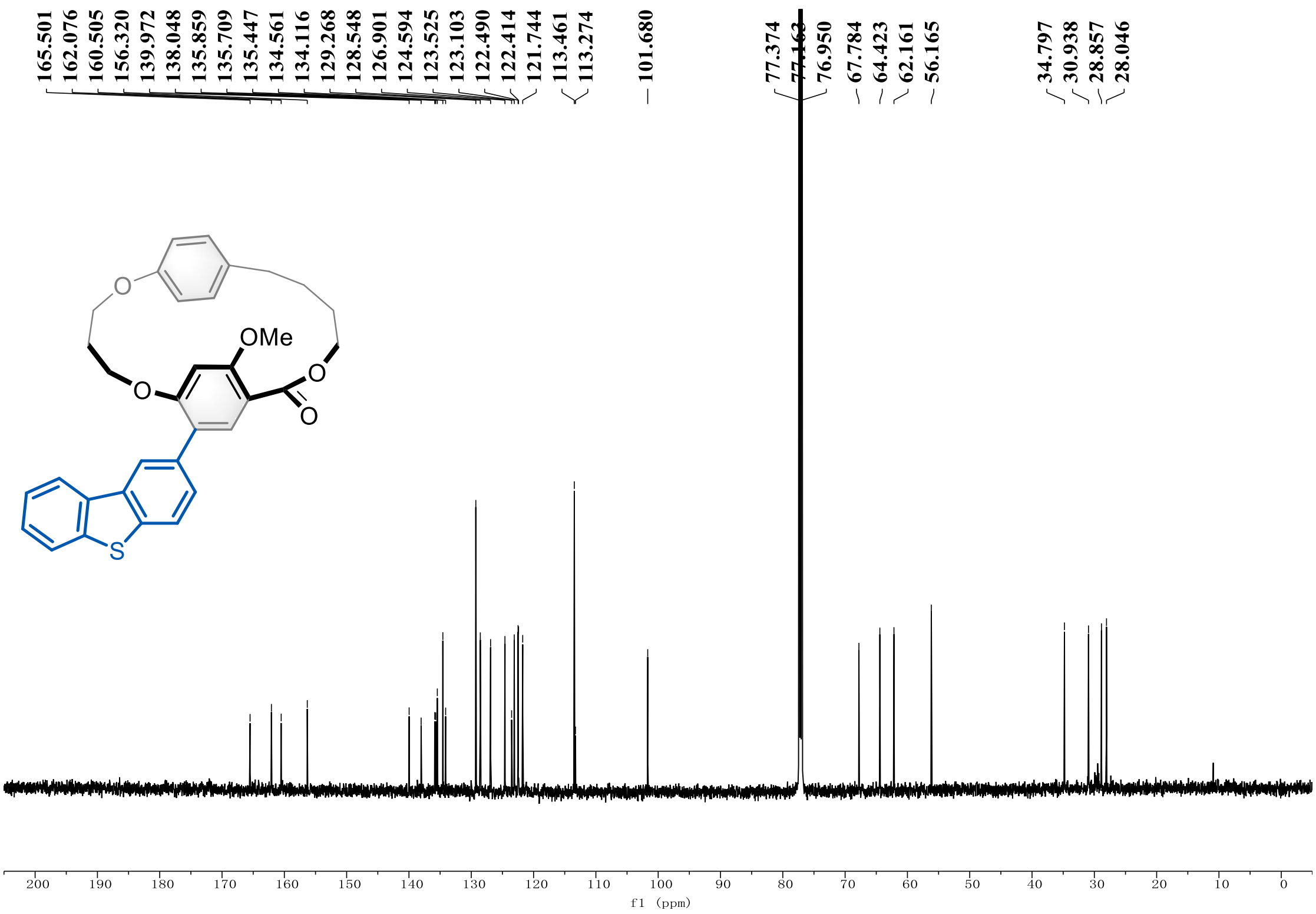

20, ^{1}H NMR (400 MHz, $CDCl_3$)

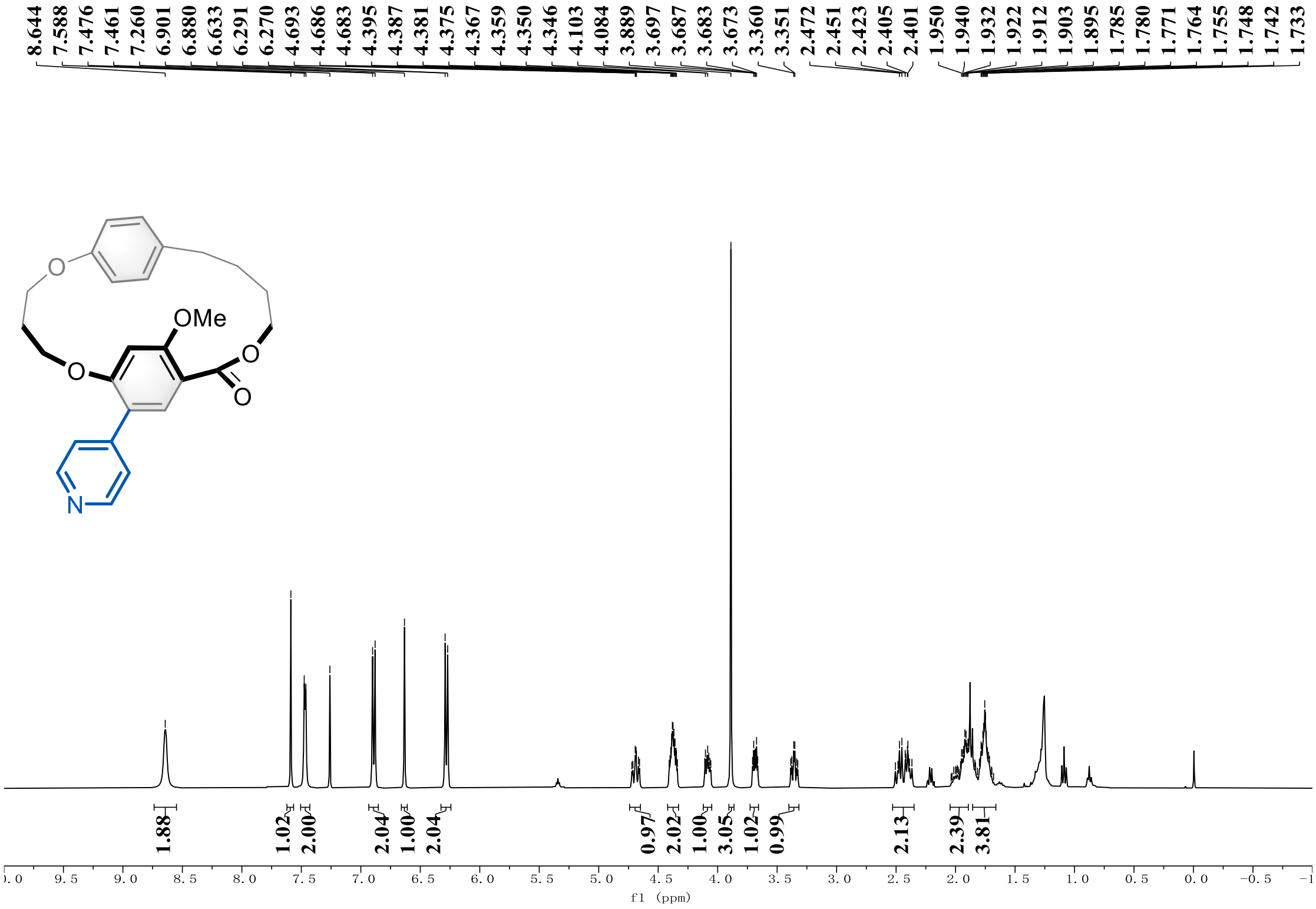

20, ^{13}C NMR (101 MHz, $CDCl_3$)

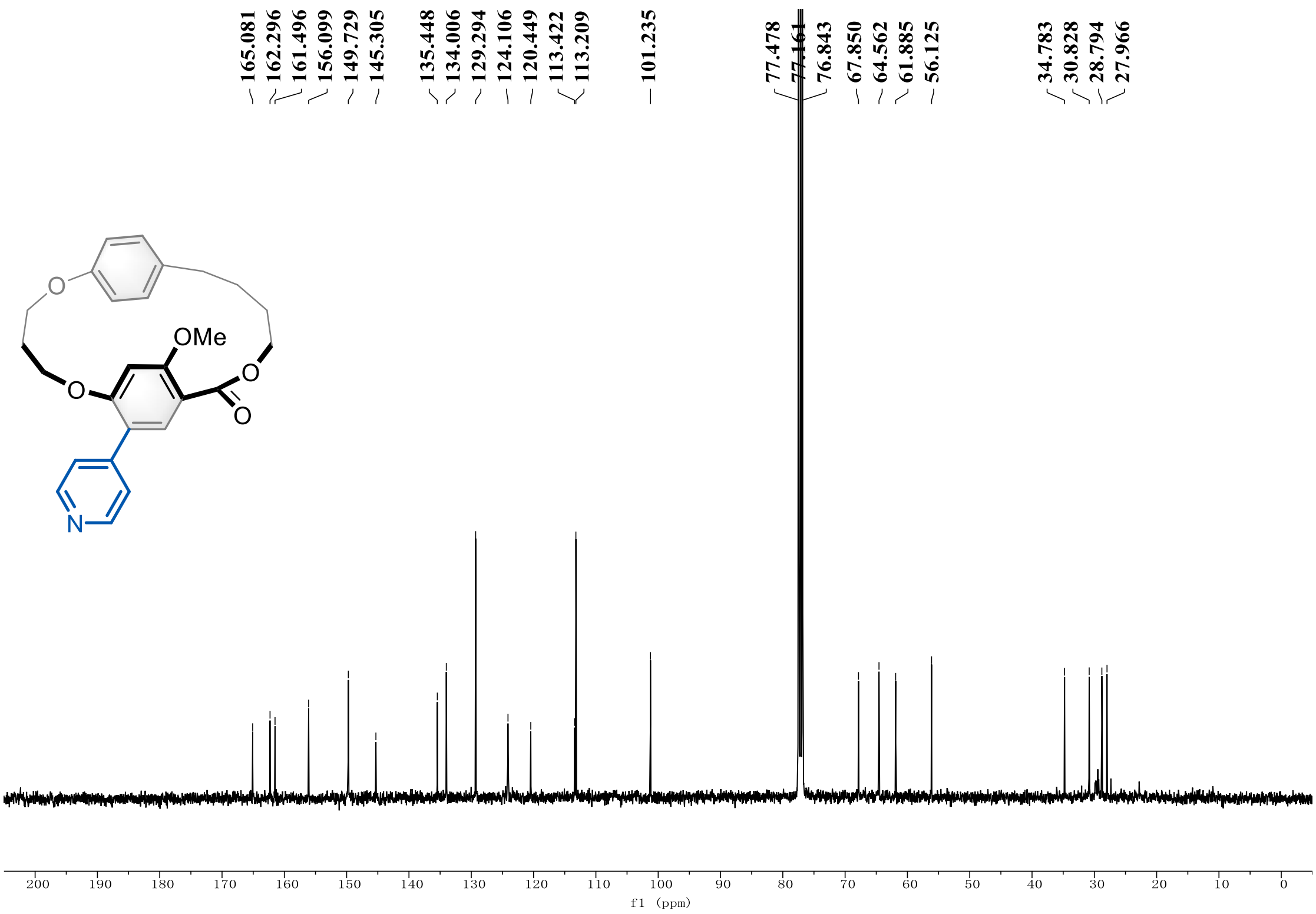

21, ^{1}H NMR (400 MHz, $CDCl_3$)

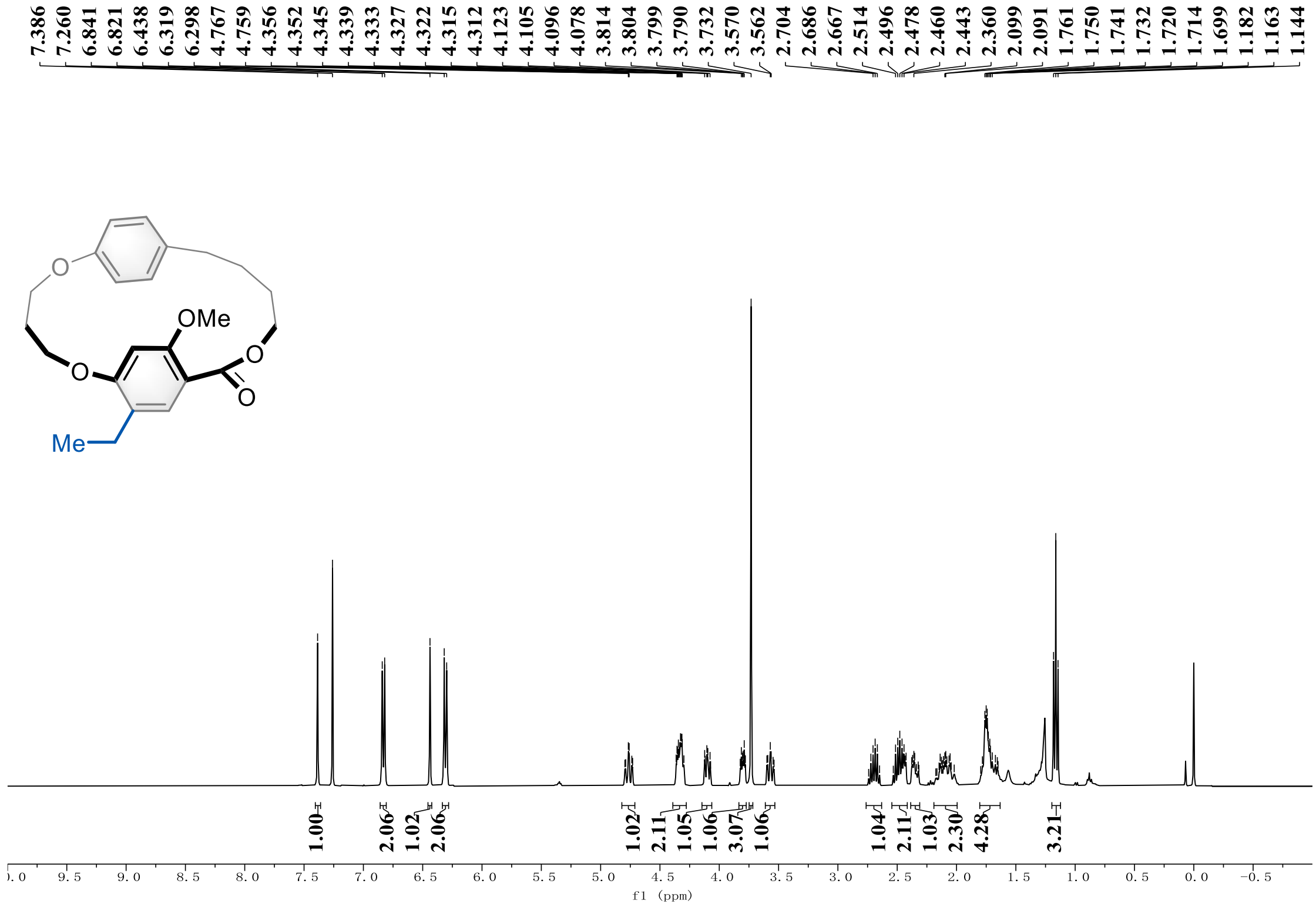

21, ^{13}C NMR (101 MHz, $CDCl_3$)

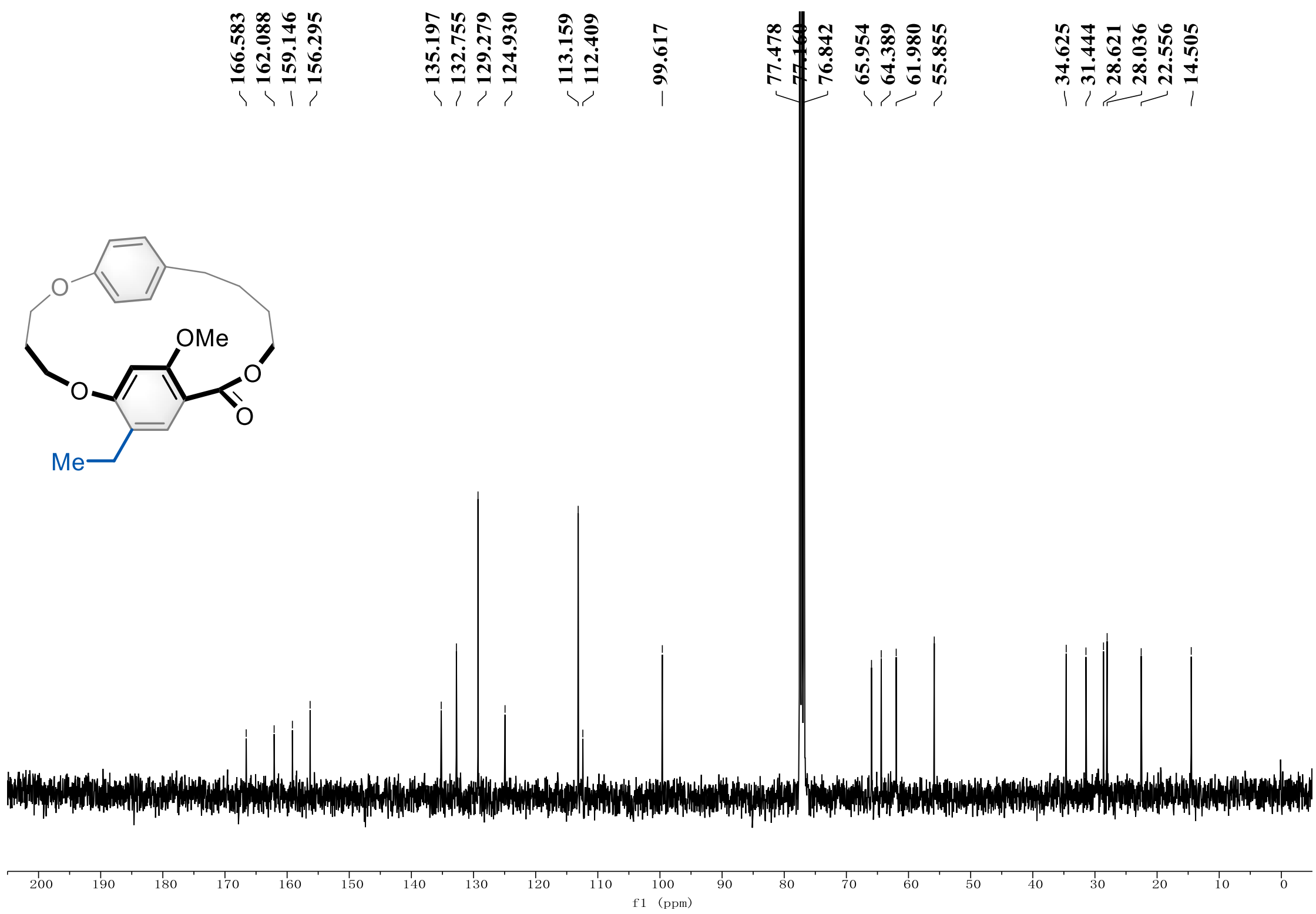

22, ^{1}H NMR (400 MHz, $CDCl_3$)

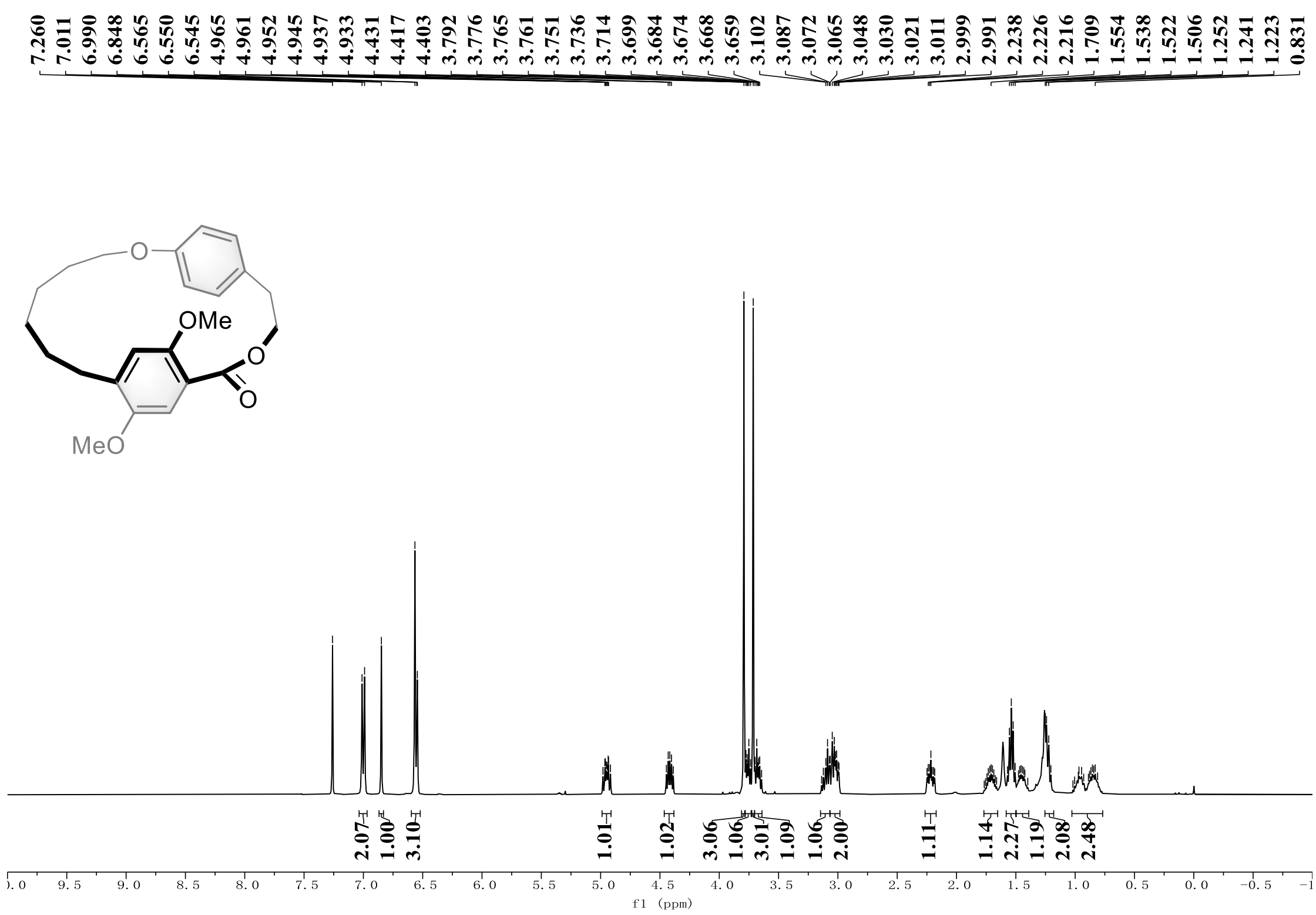

22, ^{13}C NMR (101 MHz, $CDCl_3$)

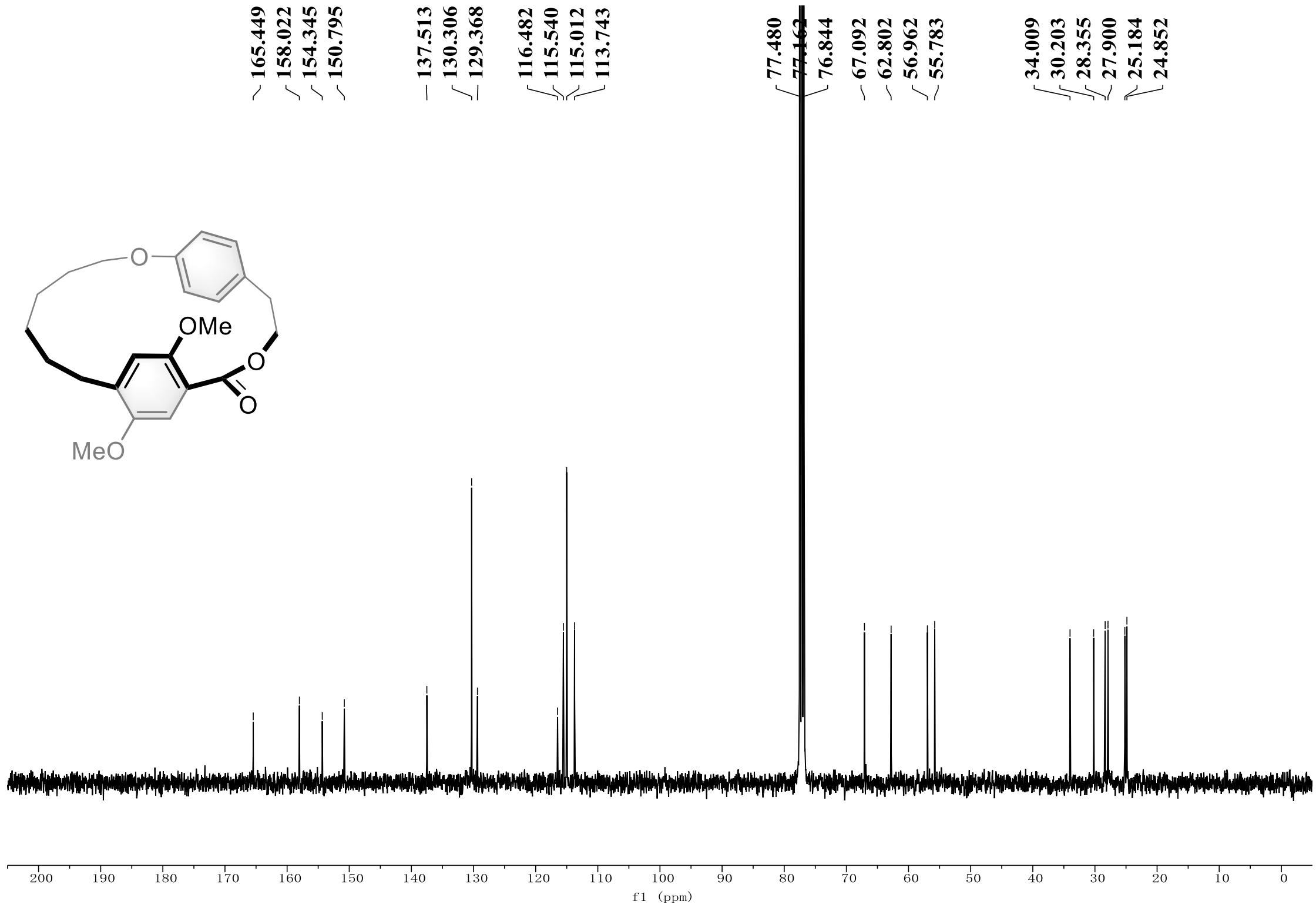

23, ^{1}H NMR (600 MHz, $CDCl_3$)

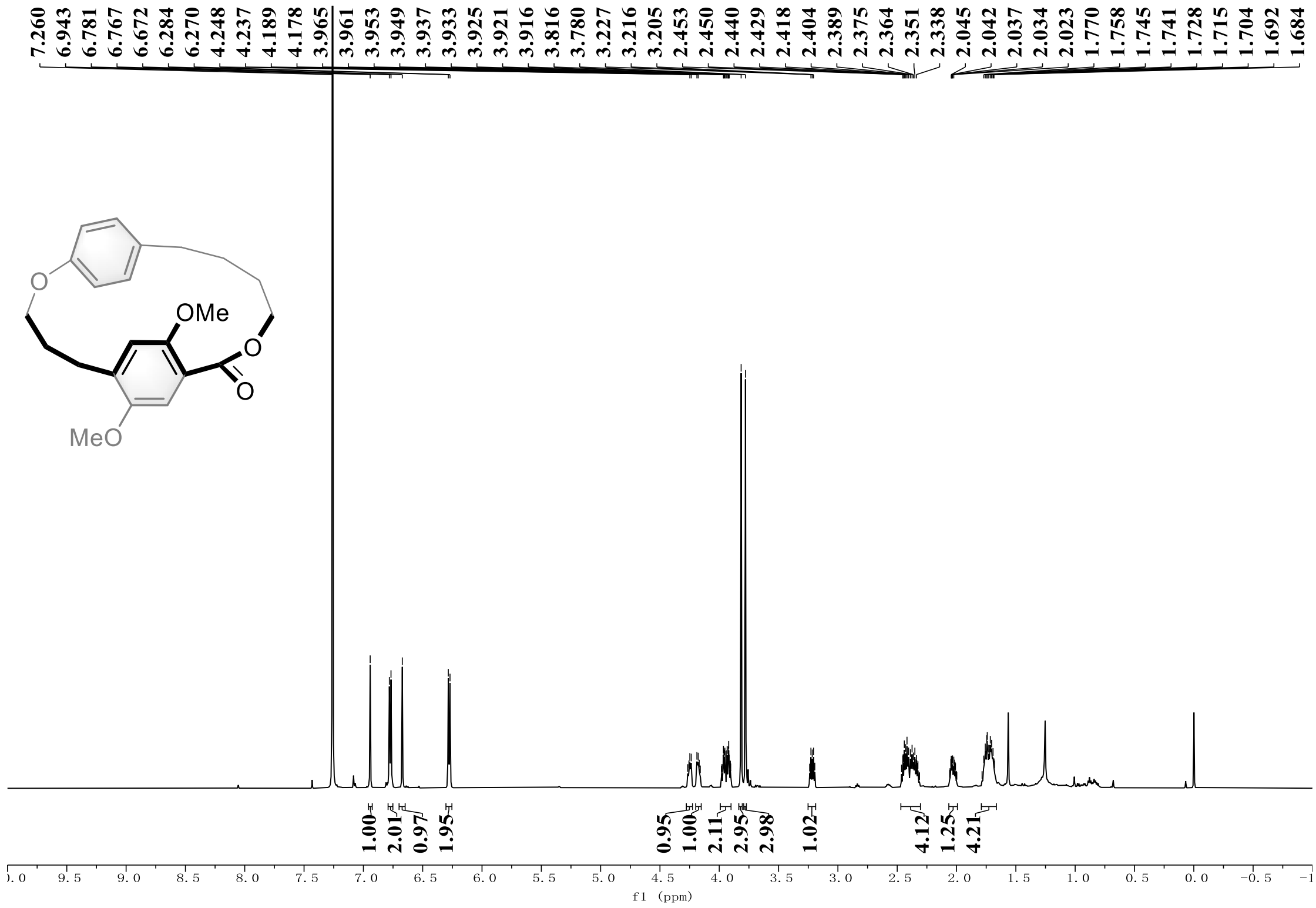

23, ^{13}C NMR (151 MHz, $CDCl_3$)

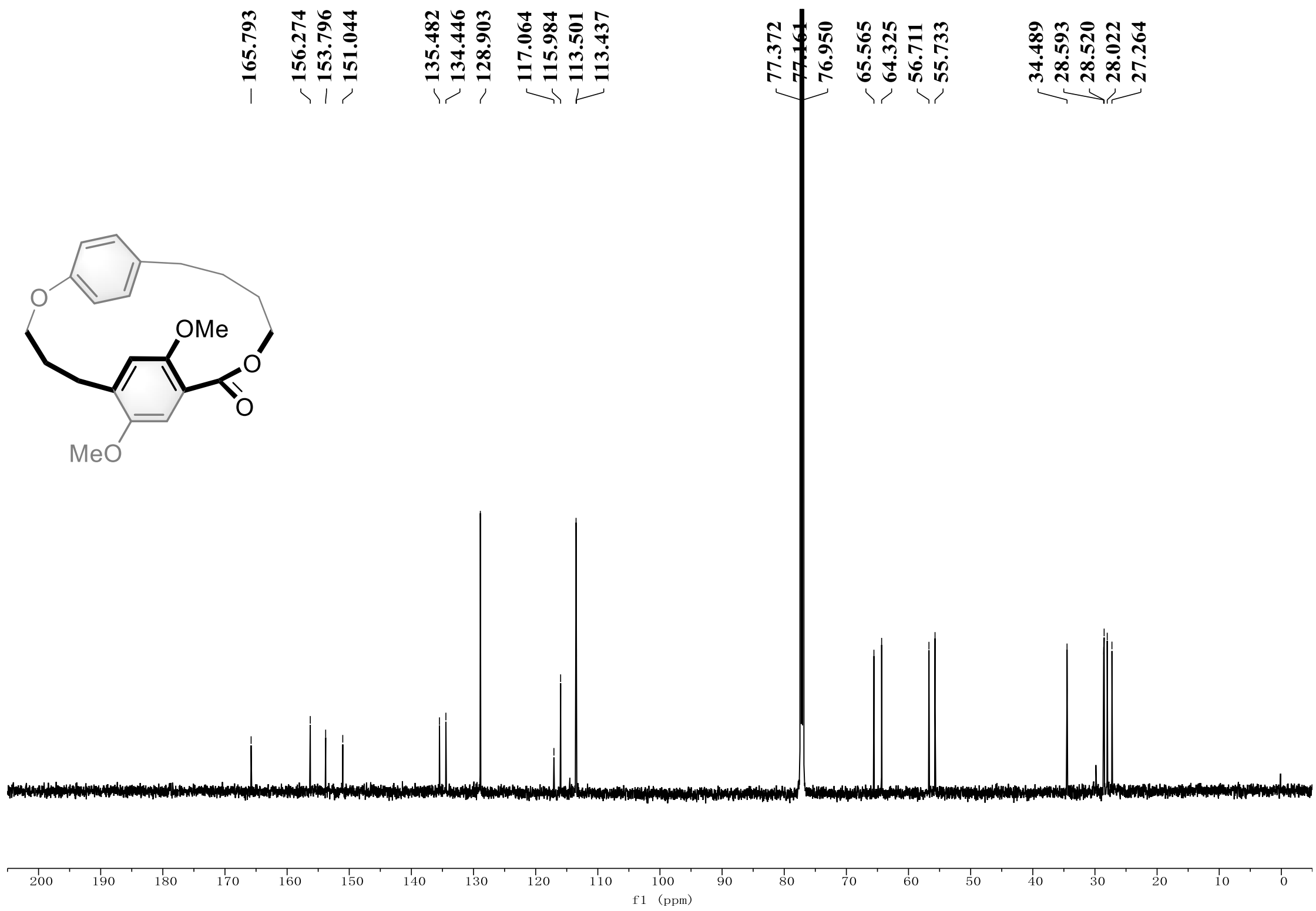

24, ^{1}H NMR (600 MHz, $CDCl_3$)

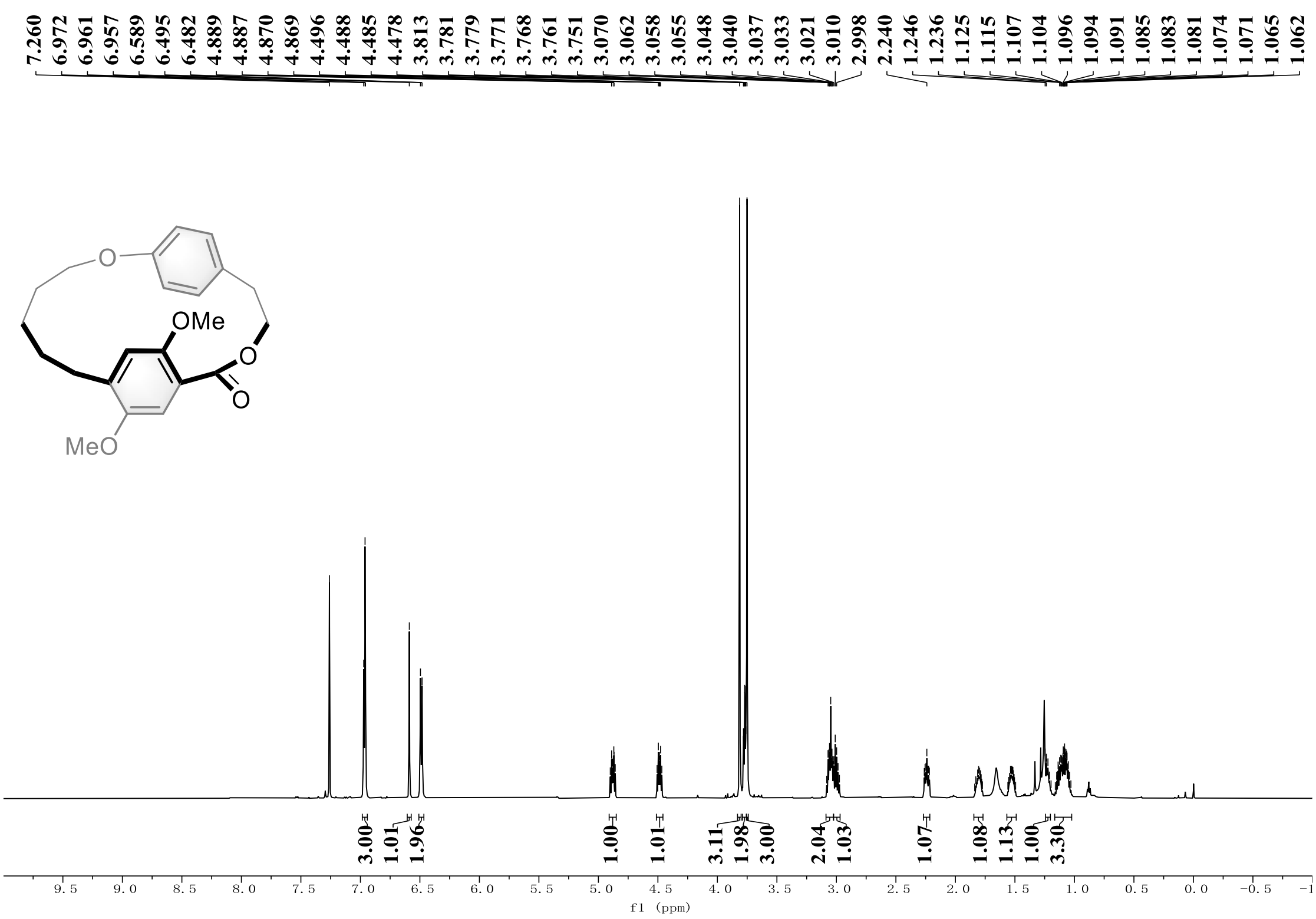

24, ^{13}C NMR (151 MHz, $CDCl_3$)

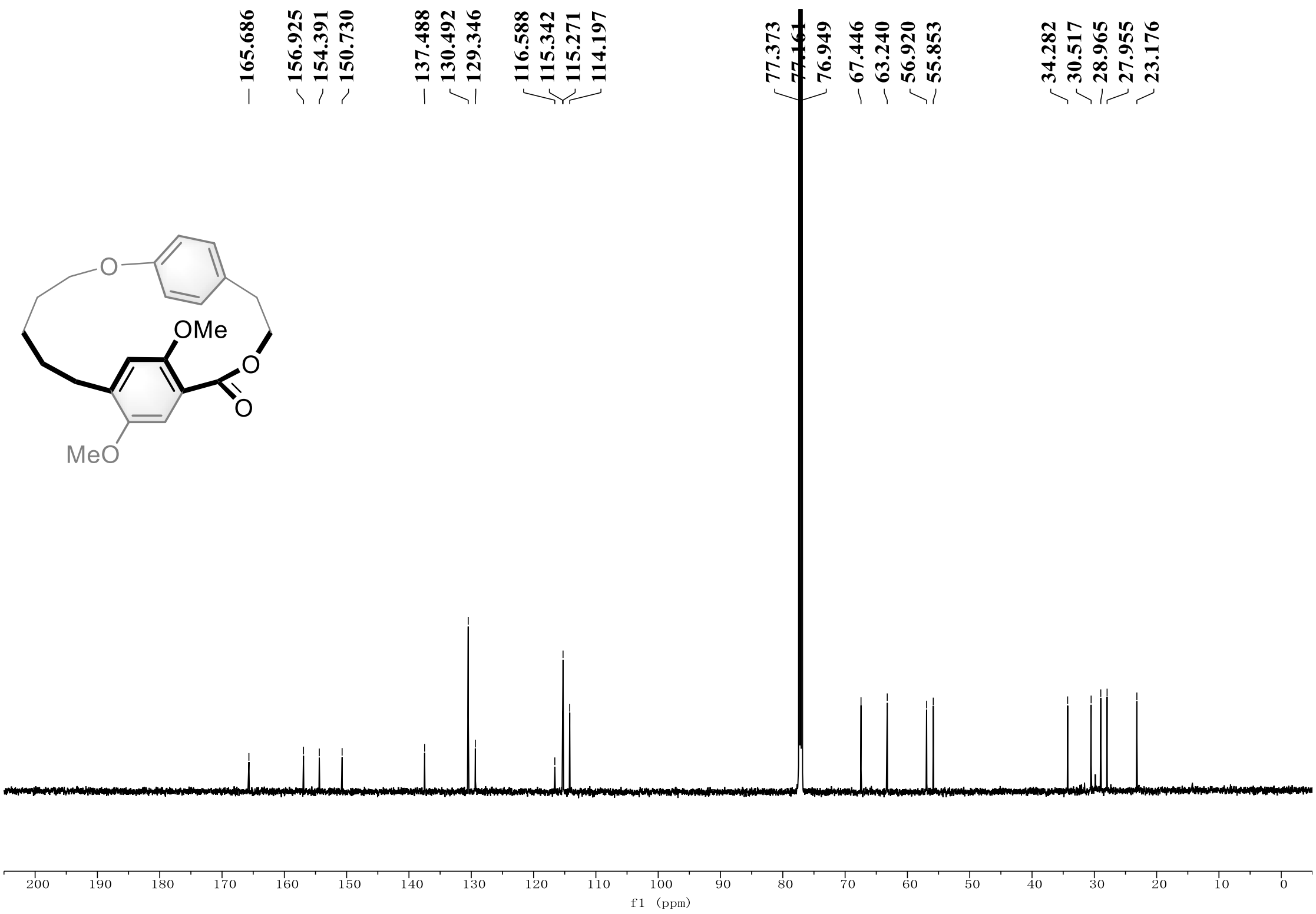

25, ^{1}H NMR (600 MHz, $CDCl_3$)

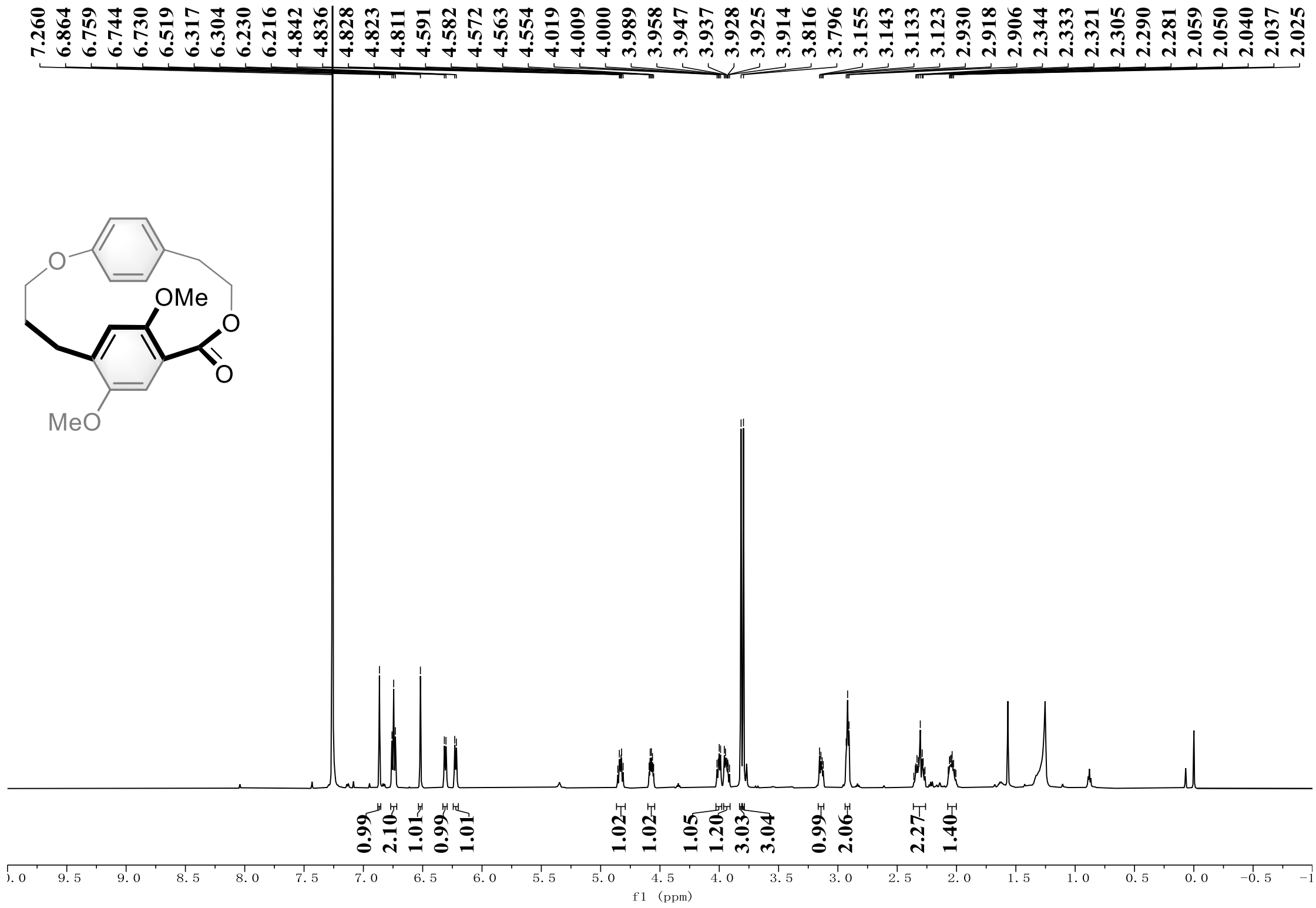

25, ^{13}C NMR (151 MHz, $CDCl_3$)

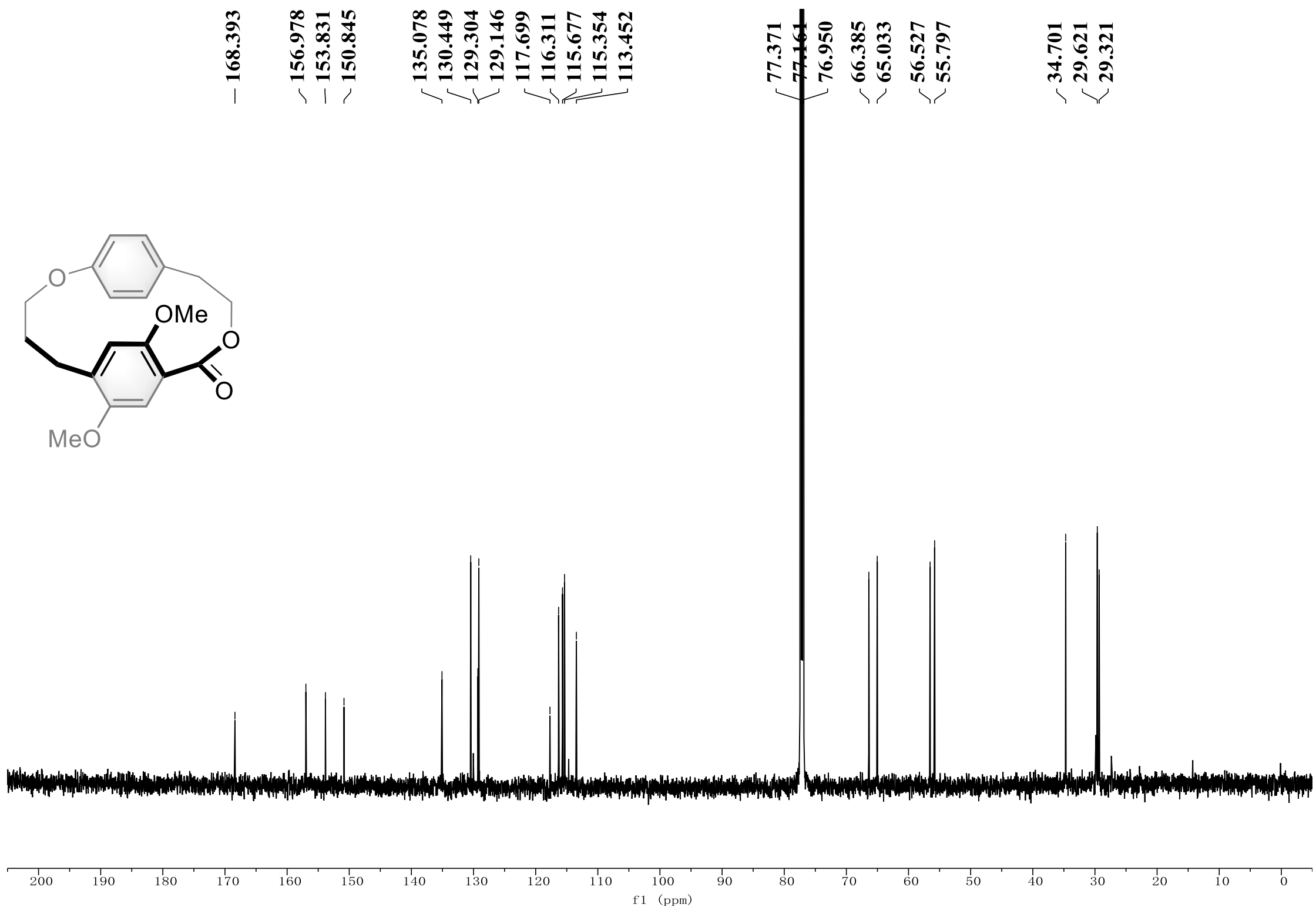

26, ^{1}H NMR (600 MHz, CDCl$_3$)

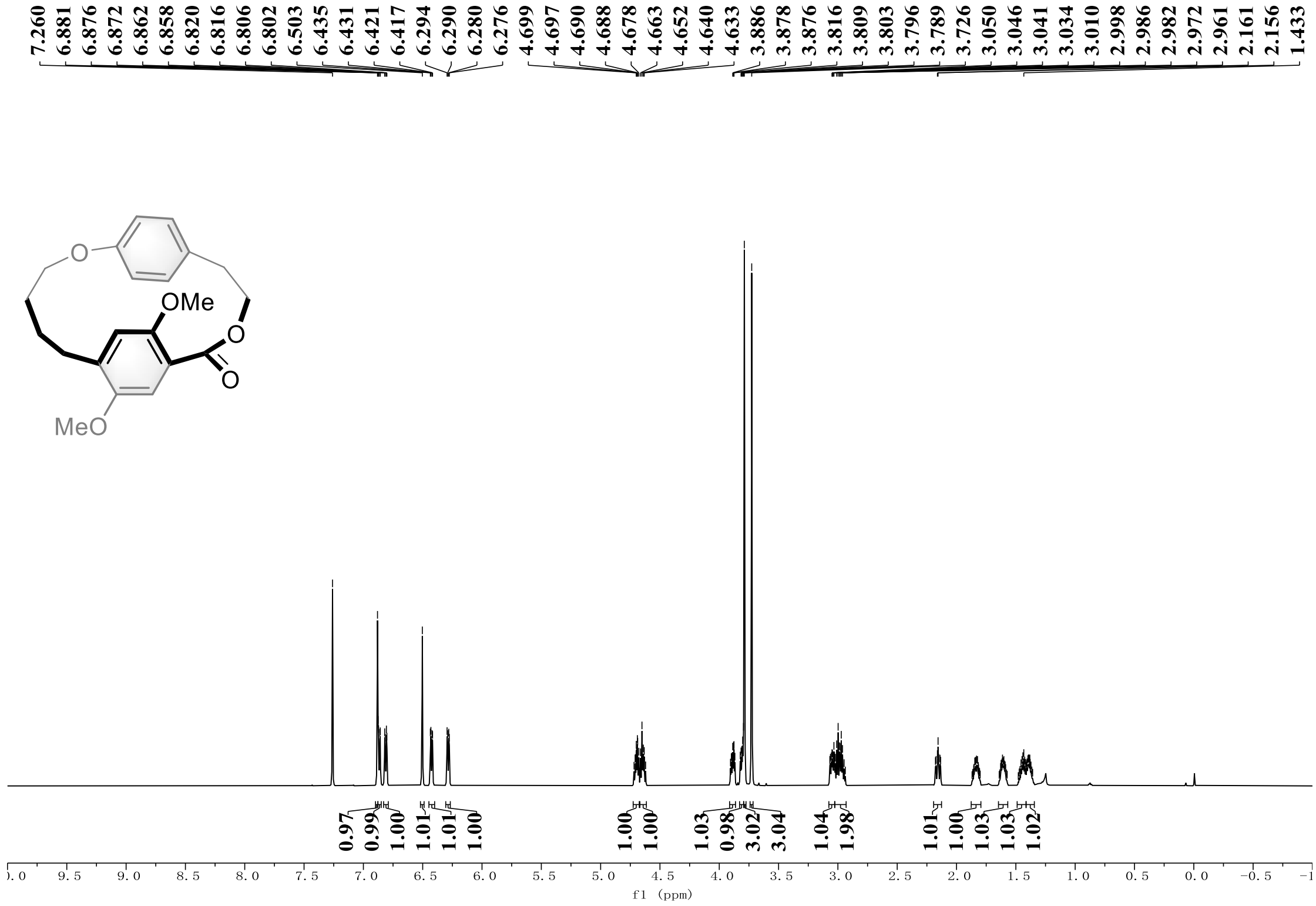

26, ^{13}C NMR (151 MHz, CDCl$_3$)

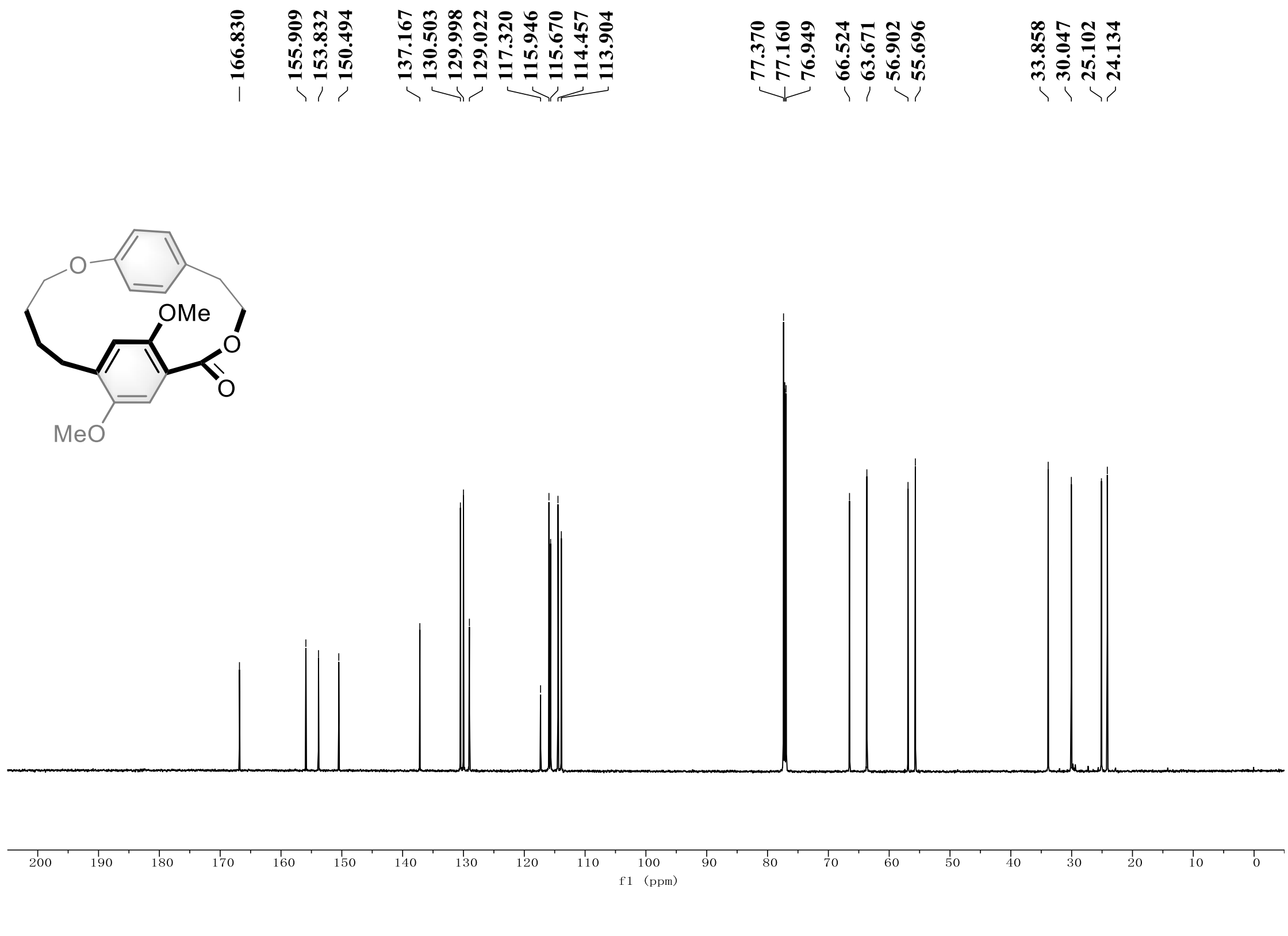

27, ¹H NMR (600 MHz, $CDCl_3$)

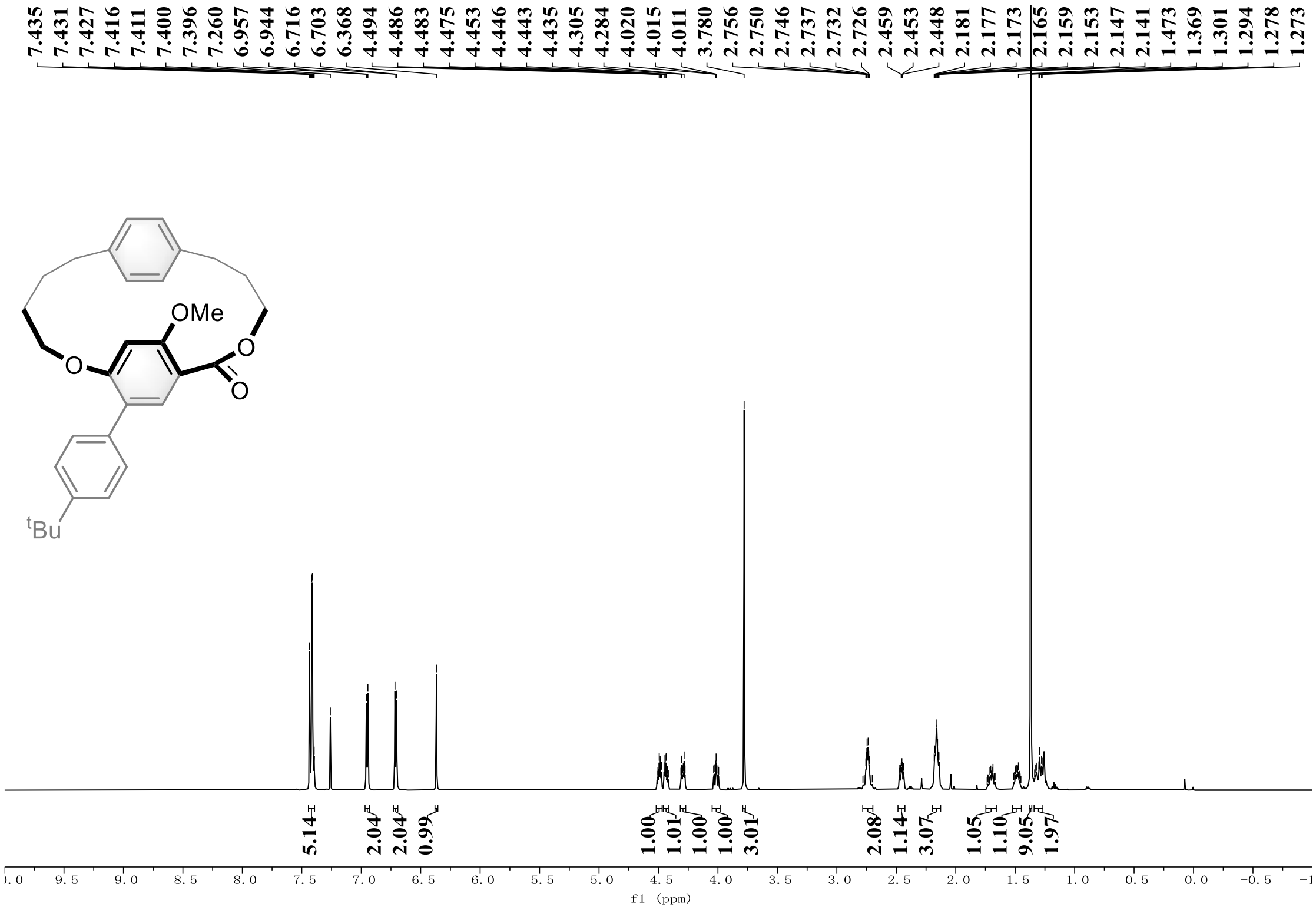

27, ¹³C NMR (151 MHz, $CDCl_3$)

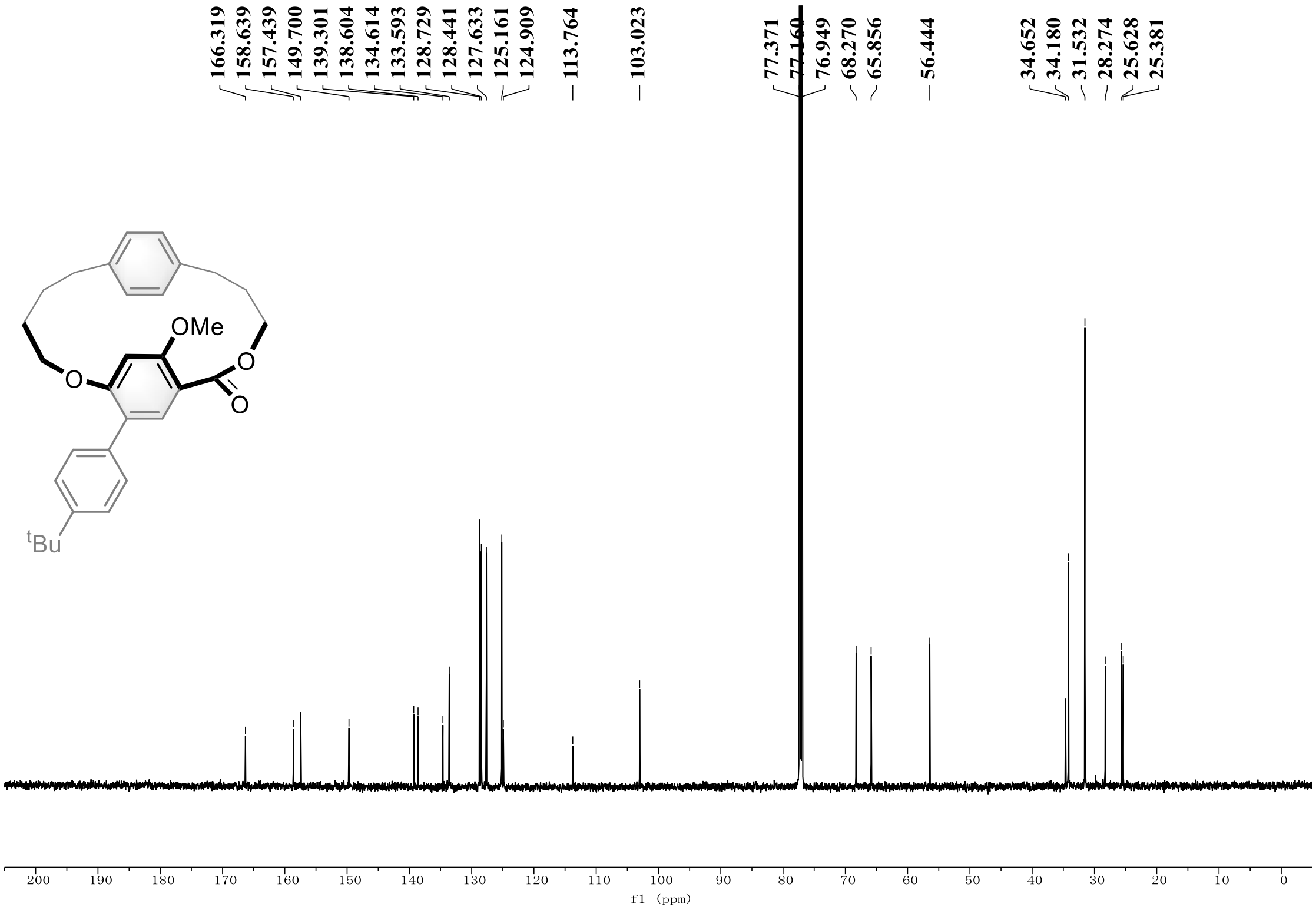

28, ^{1}H NMR (400 MHz, $CDCl_3$)

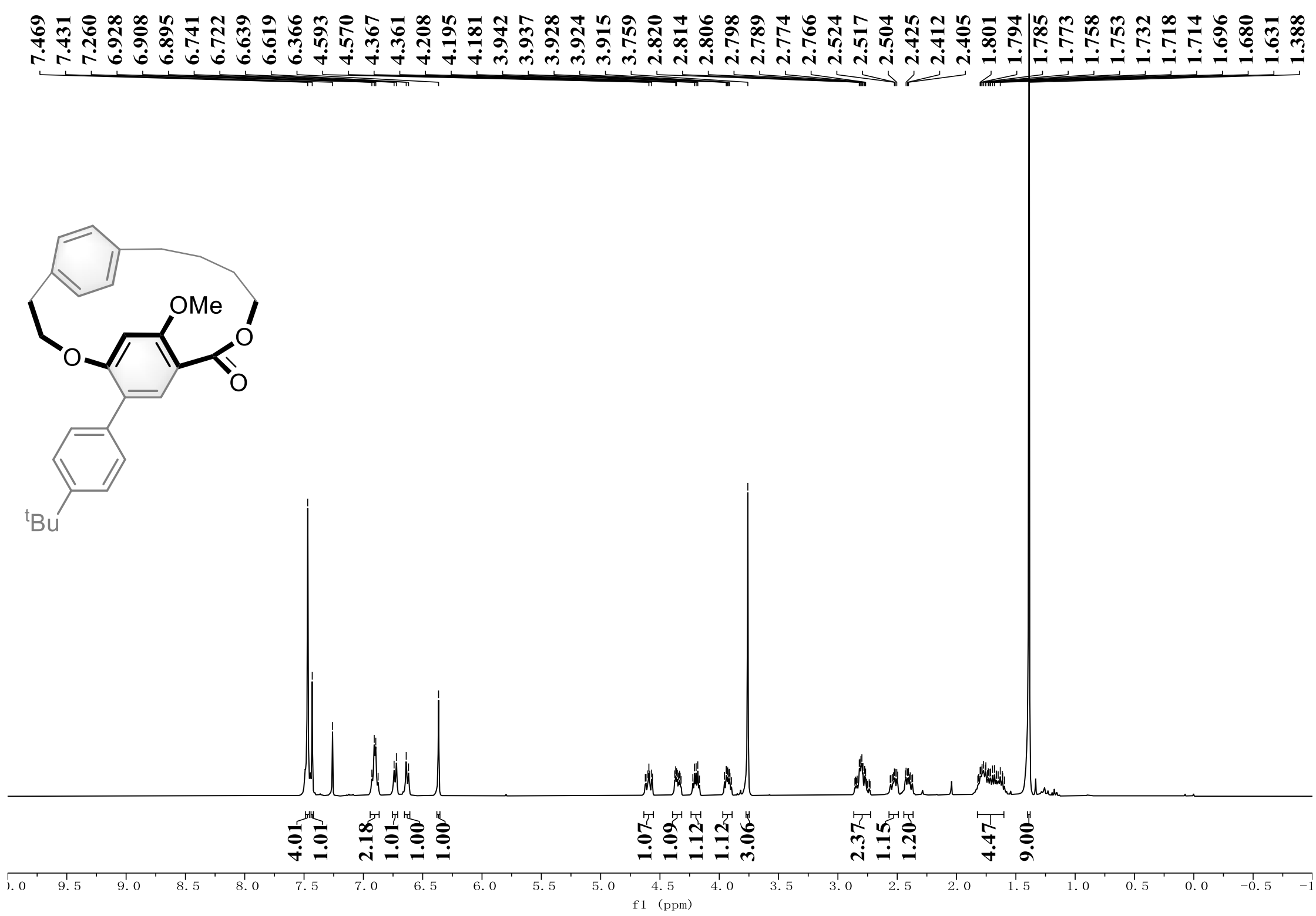

28, ^{13}C NMR (101 MHz, $CDCl_3$)

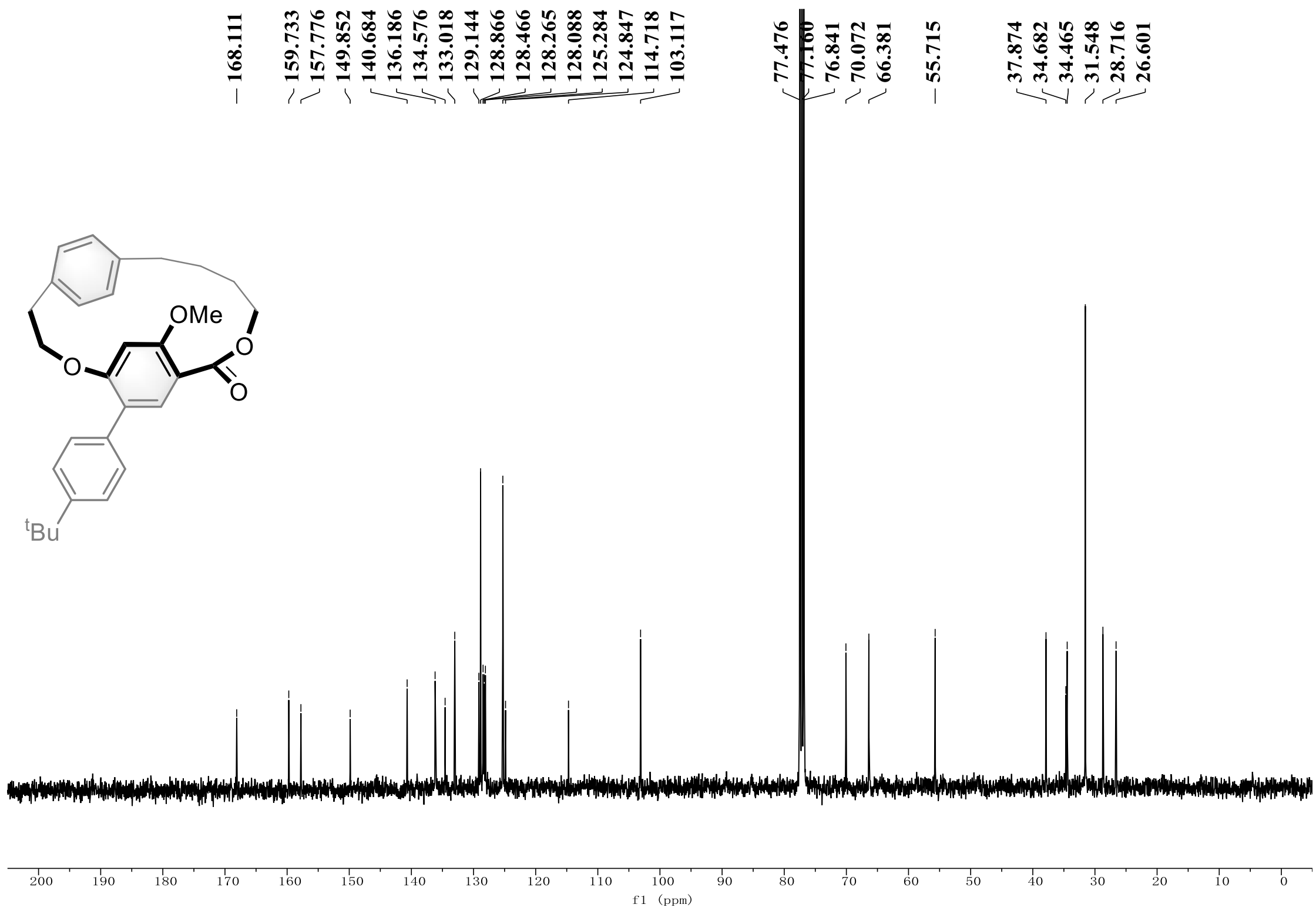

29, ^{1}H NMR (600 MHz, $CDCl_3$)

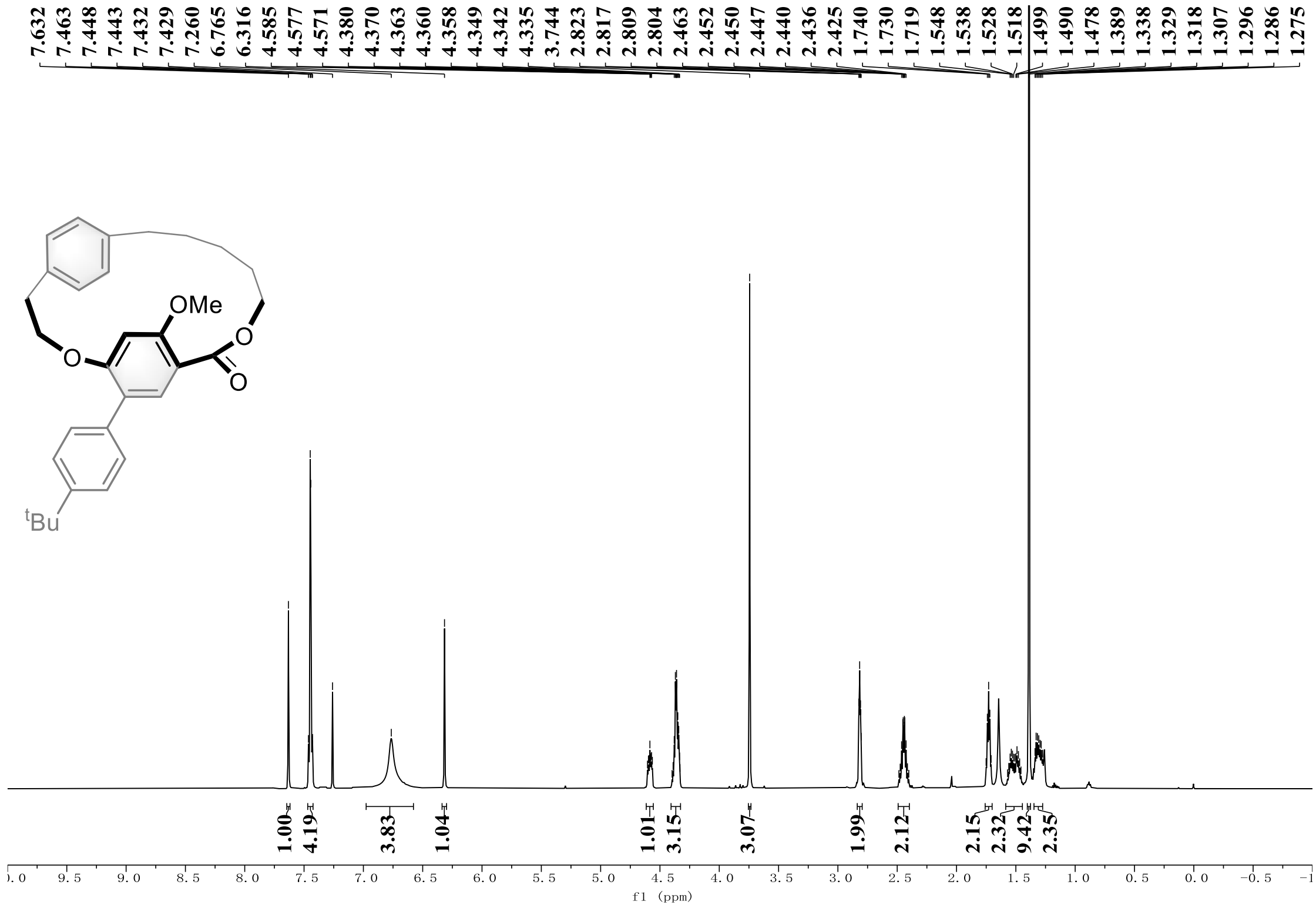

29, ^{13}C NMR (151 MHz, $CDCl_3$)

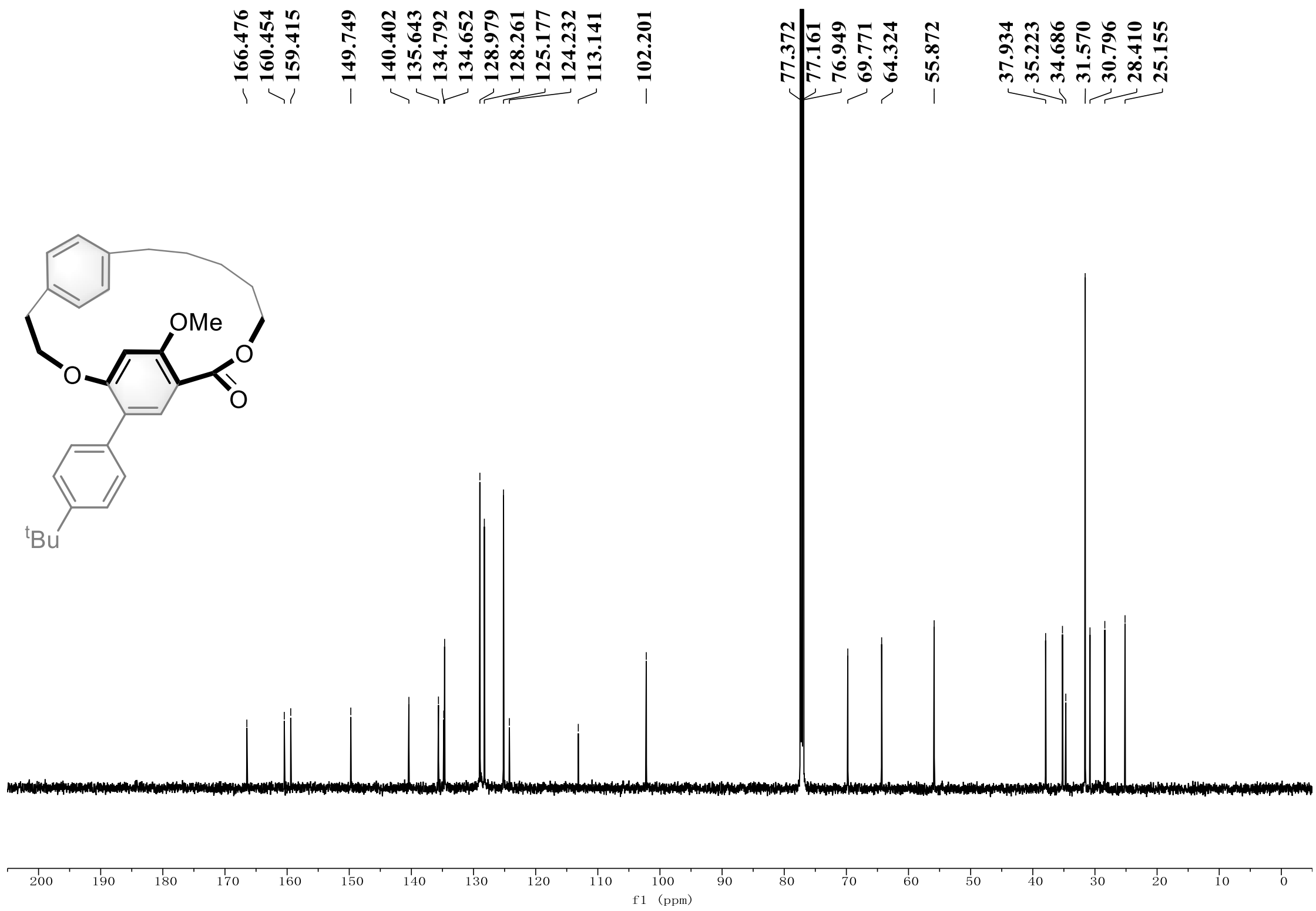

32, ^{1}H NMR (600 MHz, $CDCl_3$)

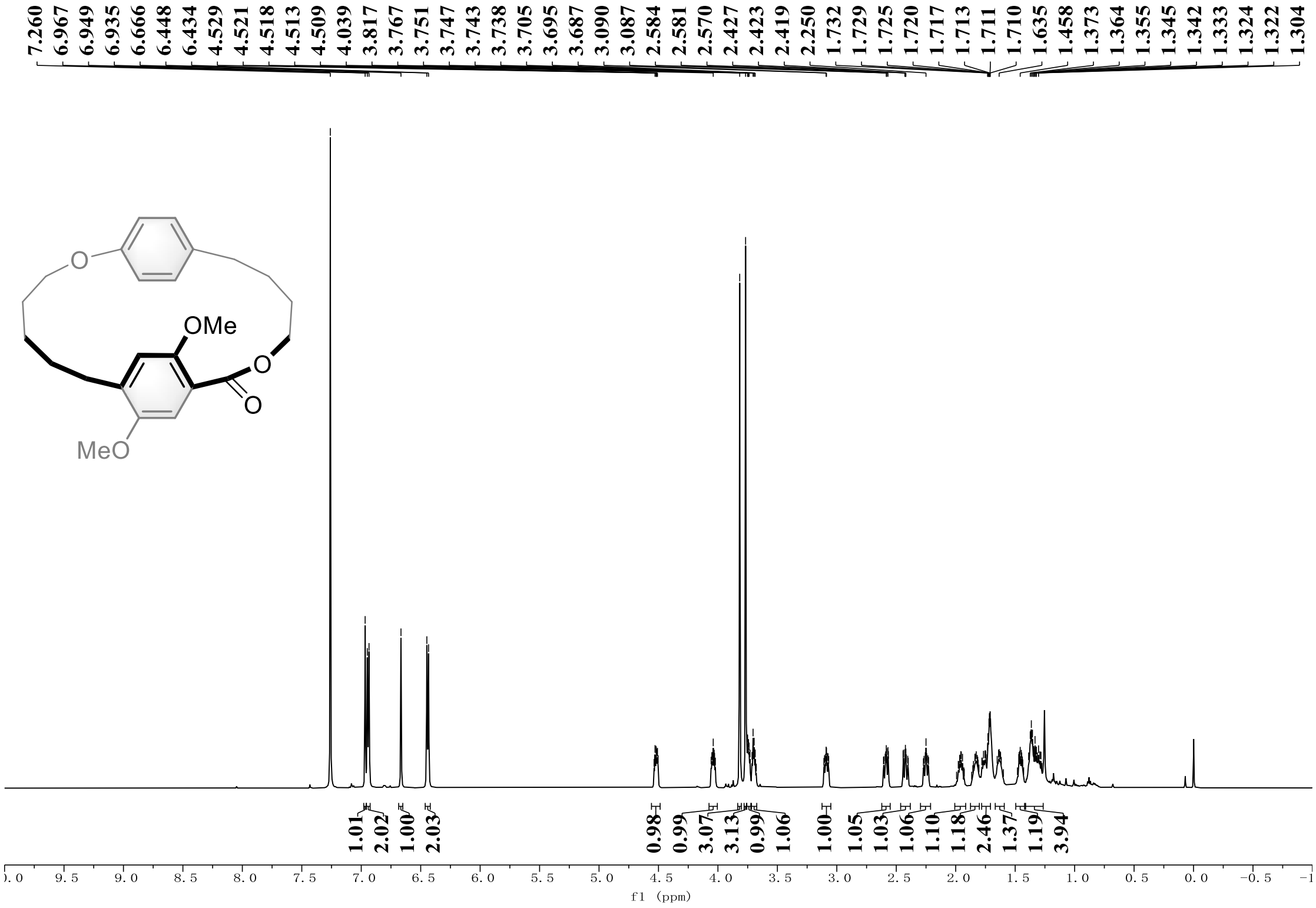

32, ^{13}C NMR (151 MHz, $CDCl_3$)

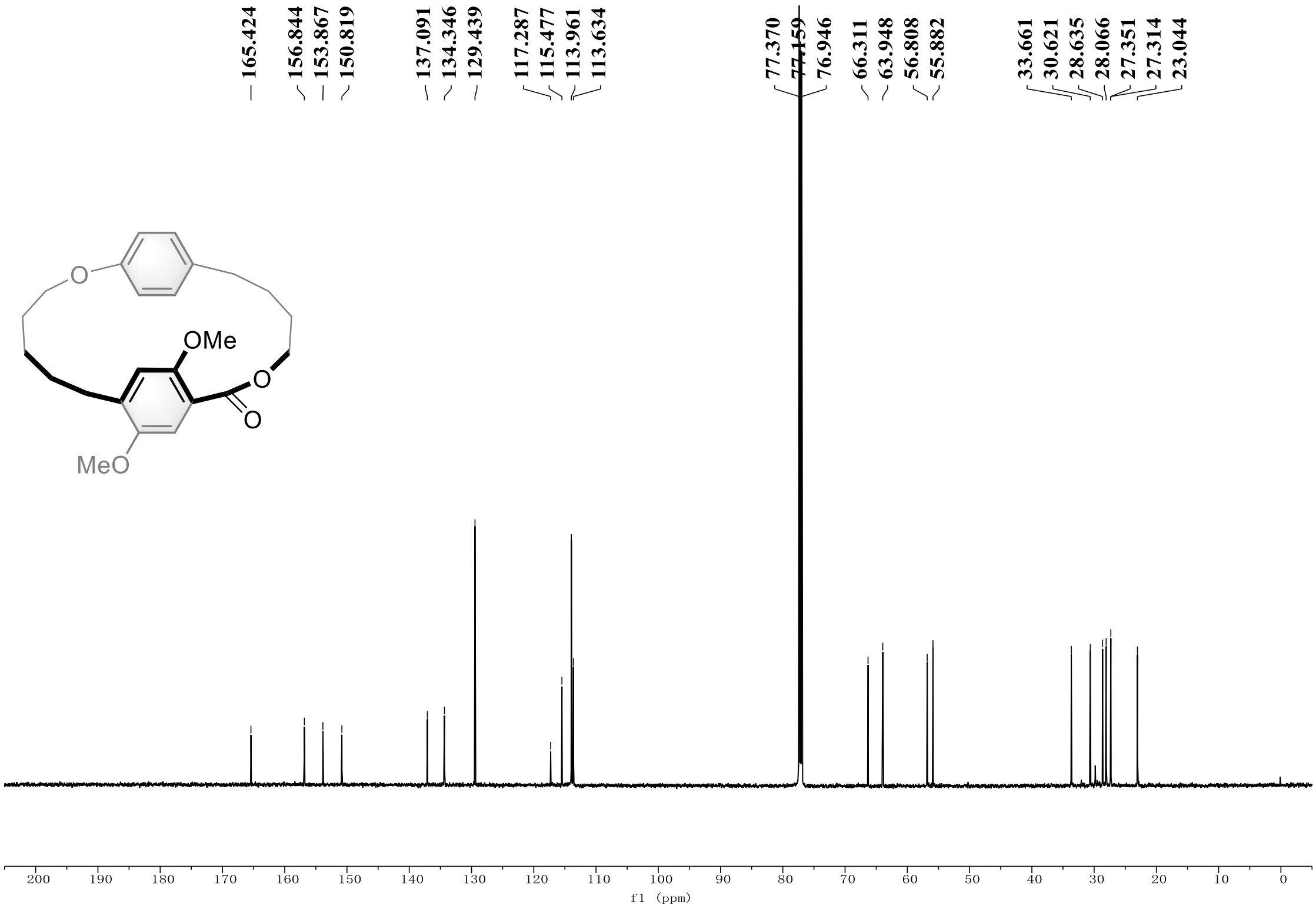

33, ^{1}H NMR (400 MHz, $CDCl_3$)

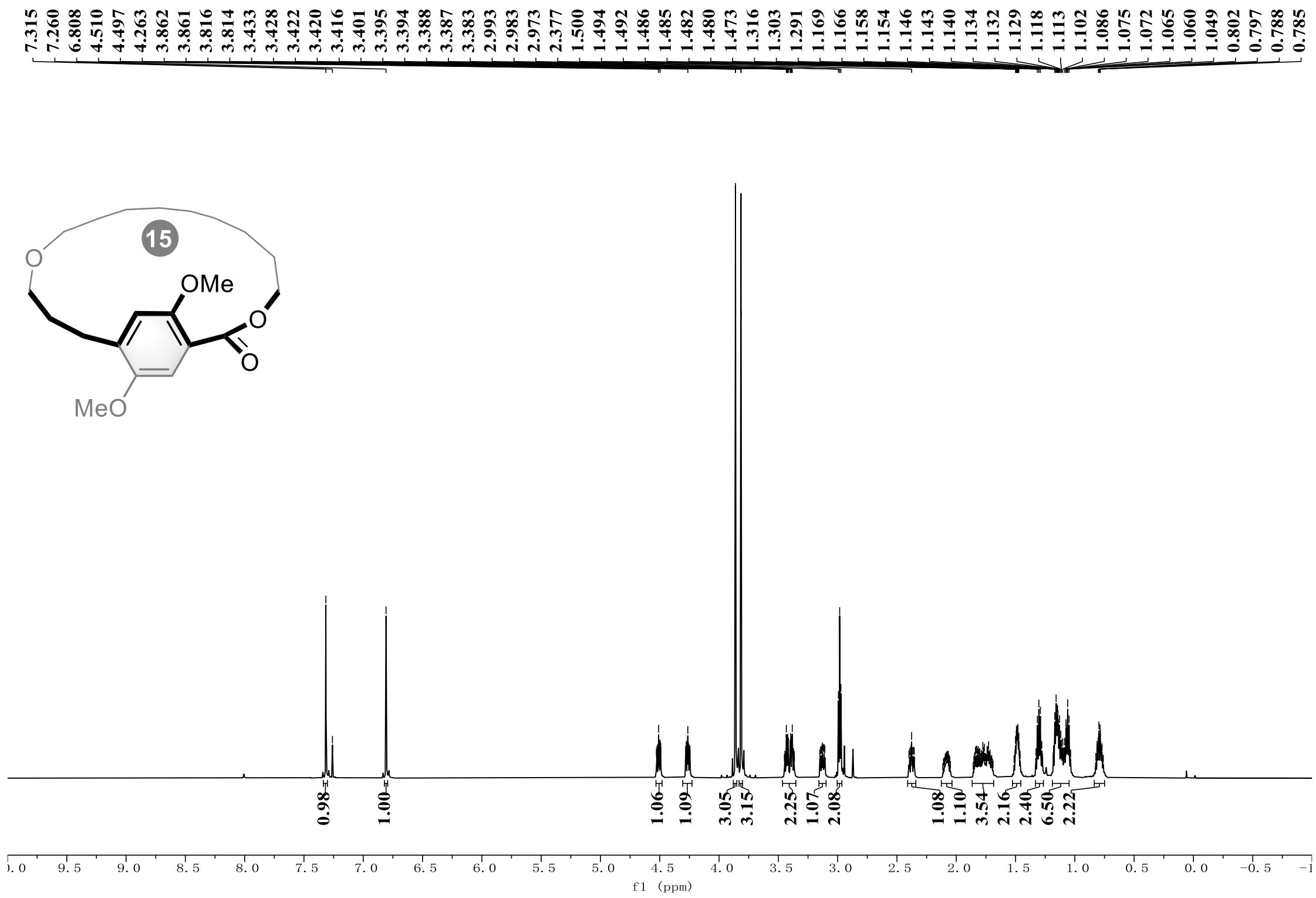

33, ^{13}C NMR (101 MHz, $CDCl_3$)

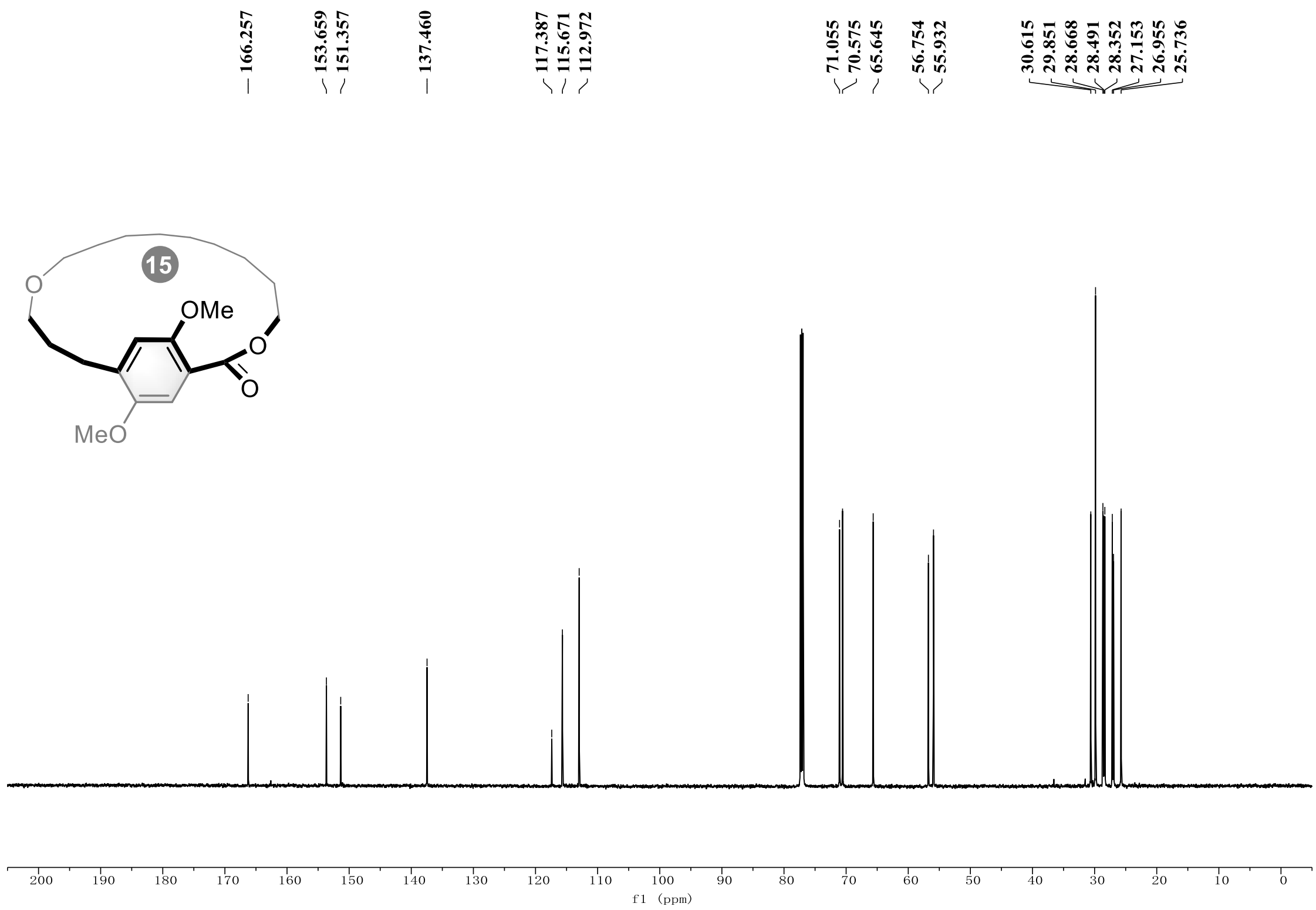

34, ^{1}H NMR (400 MHz, $CDCl_3$)

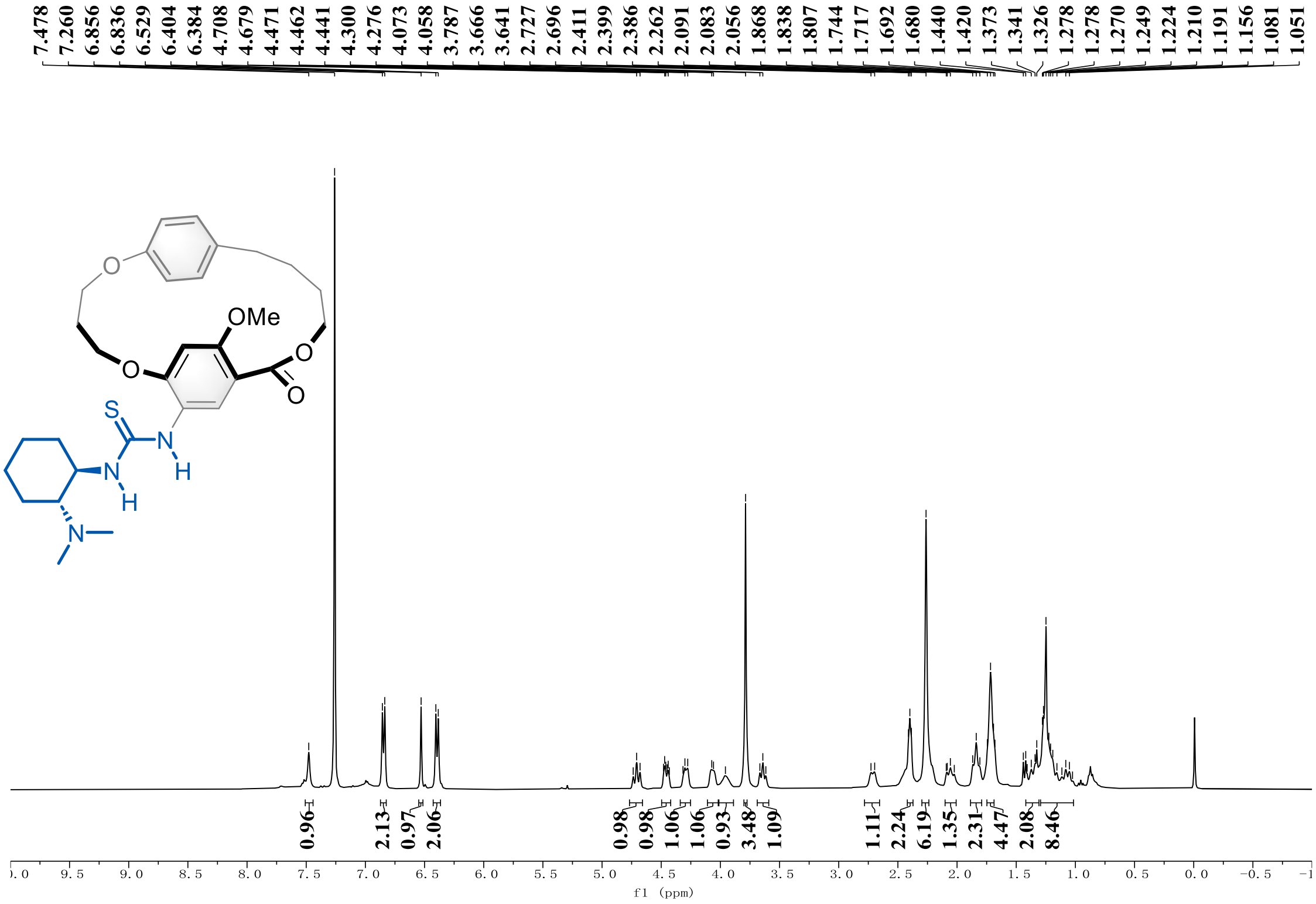

34, ^{13}C NMR (101 MHz, $CDCl_3$)

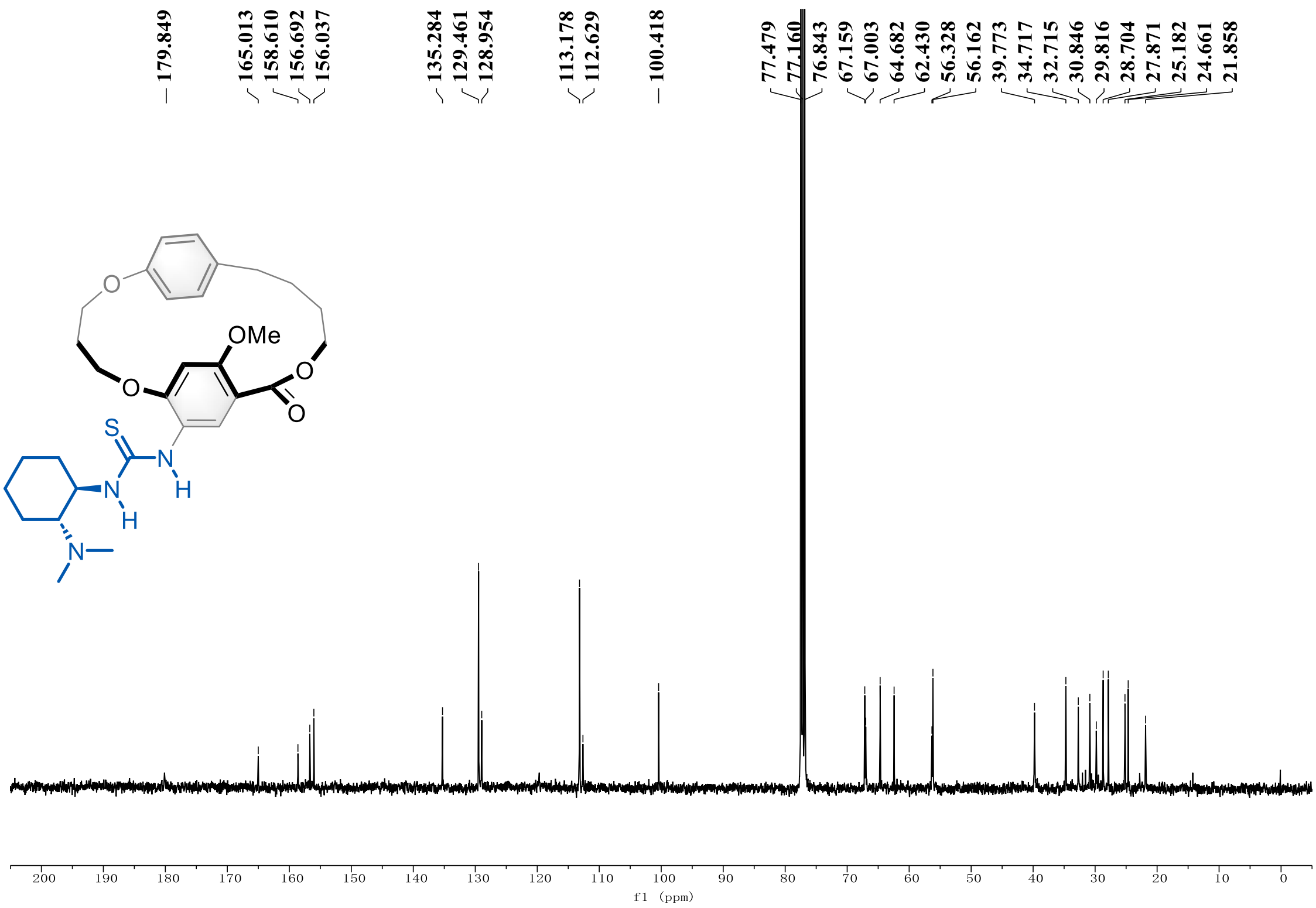

35, ^{1}H NMR (600 MHz, $CDCl_3$)

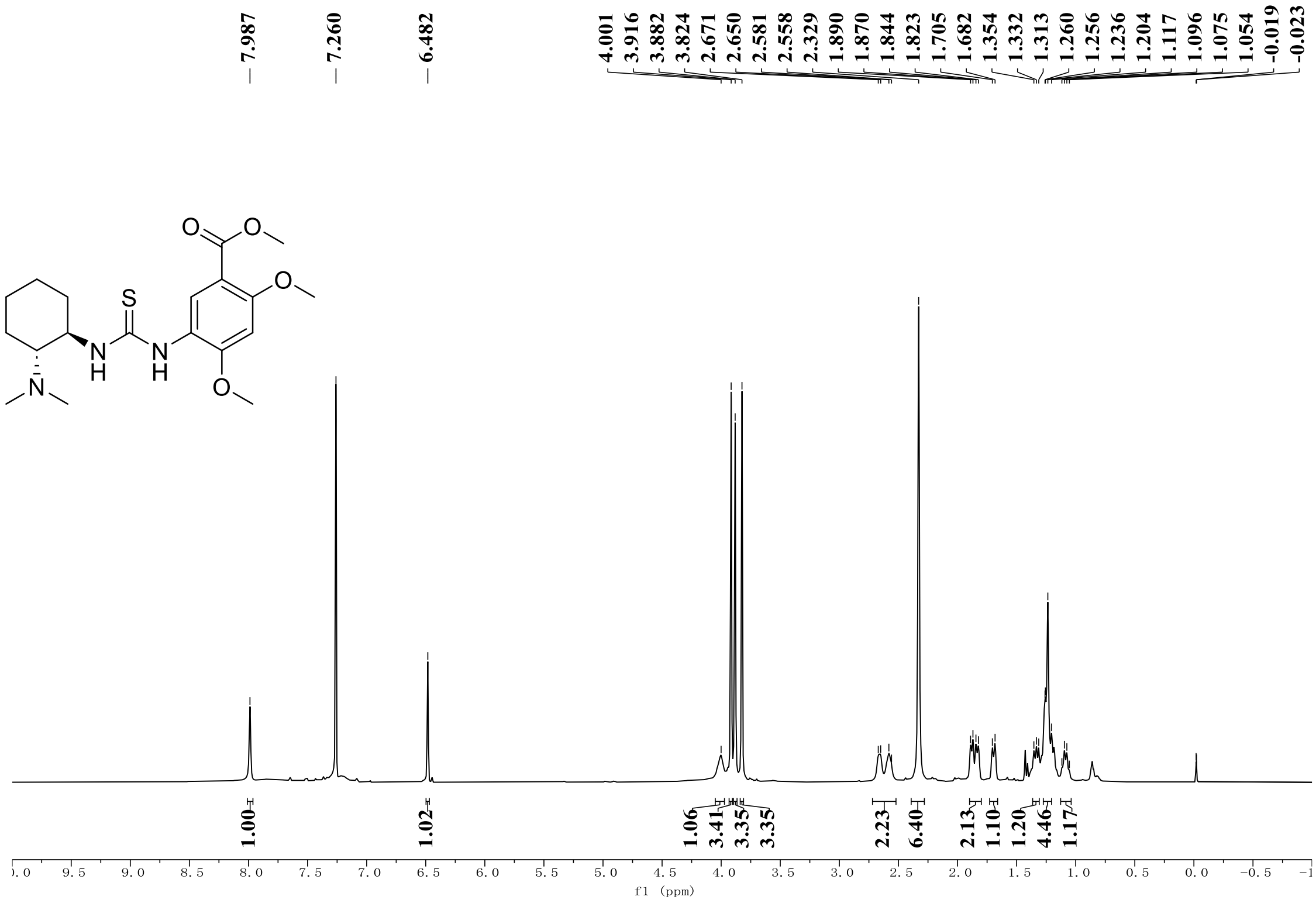

35, ^{13}C NMR (151 MHz, $CDCl_3$)

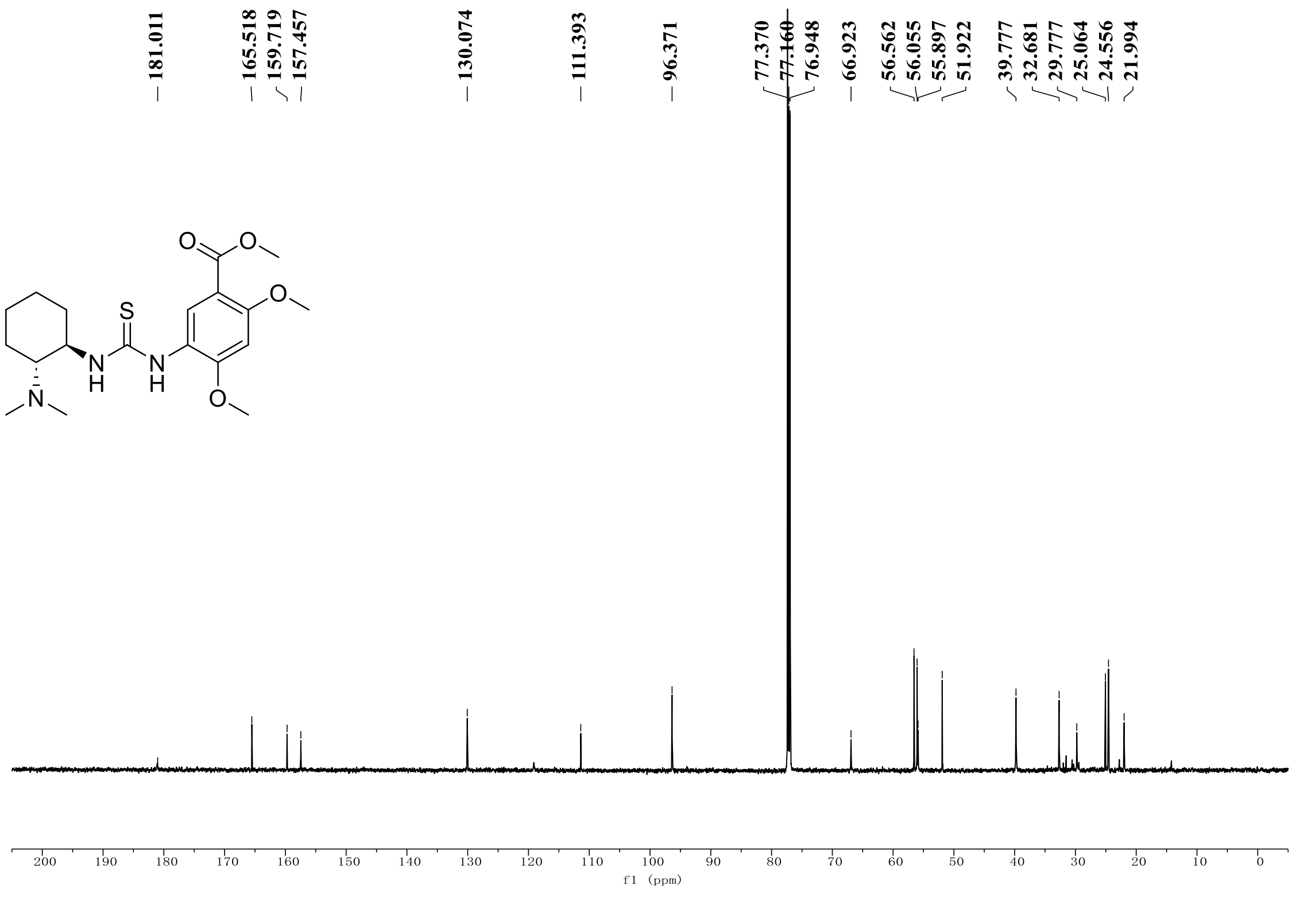

10. Copies of HPLC spectra

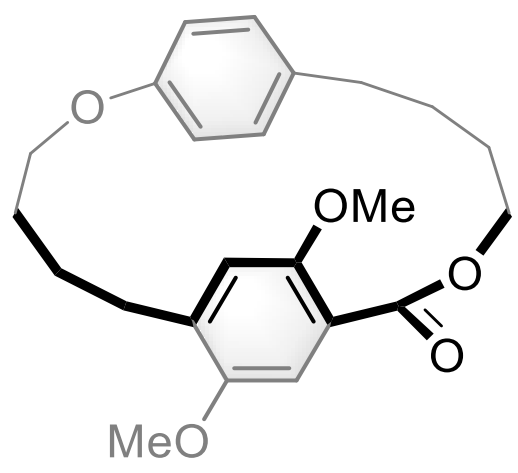

2

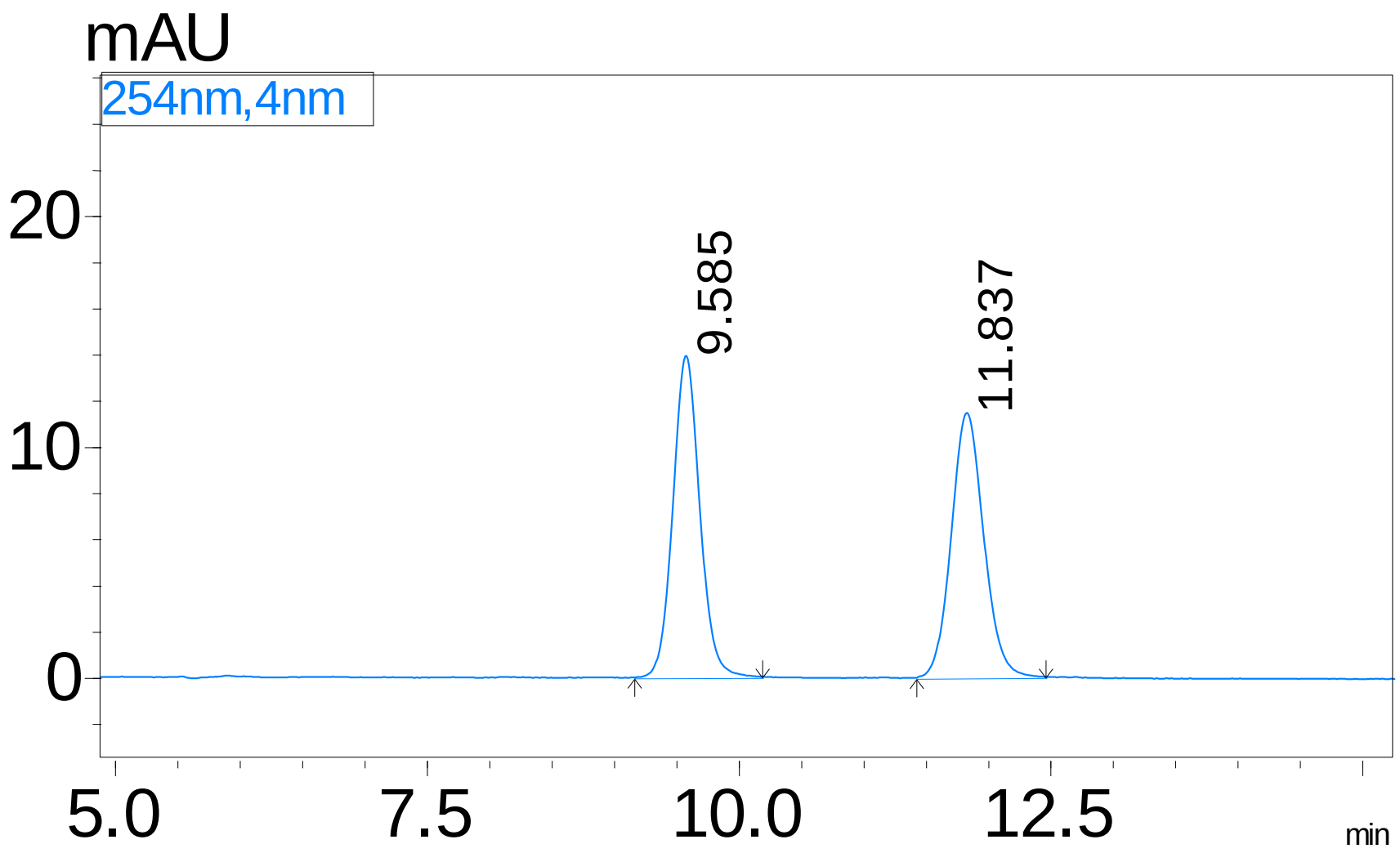

Peak	Time	Area	Height	Area%
1	9.585	208652	13918	50.198
2	11.837	207003	11458	49.802

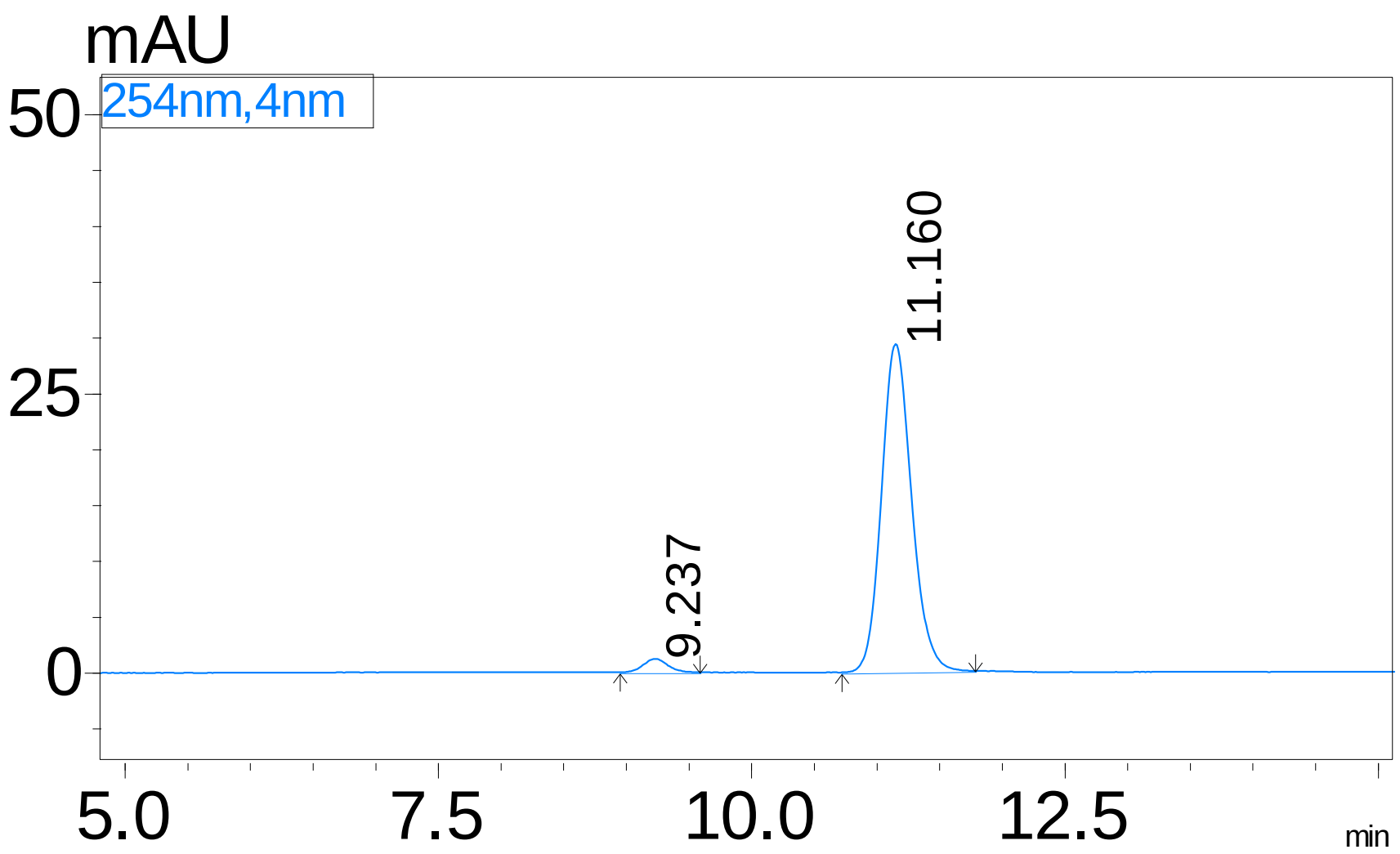

Peak	Time	Area	Height	Area%
1	9.237	16253	1197	3.216
2	11.160	489160	29339	96.784

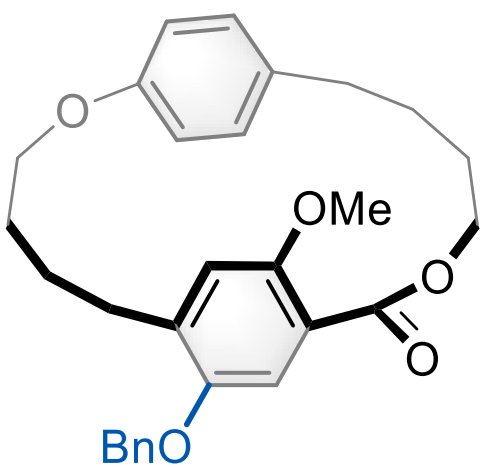

4

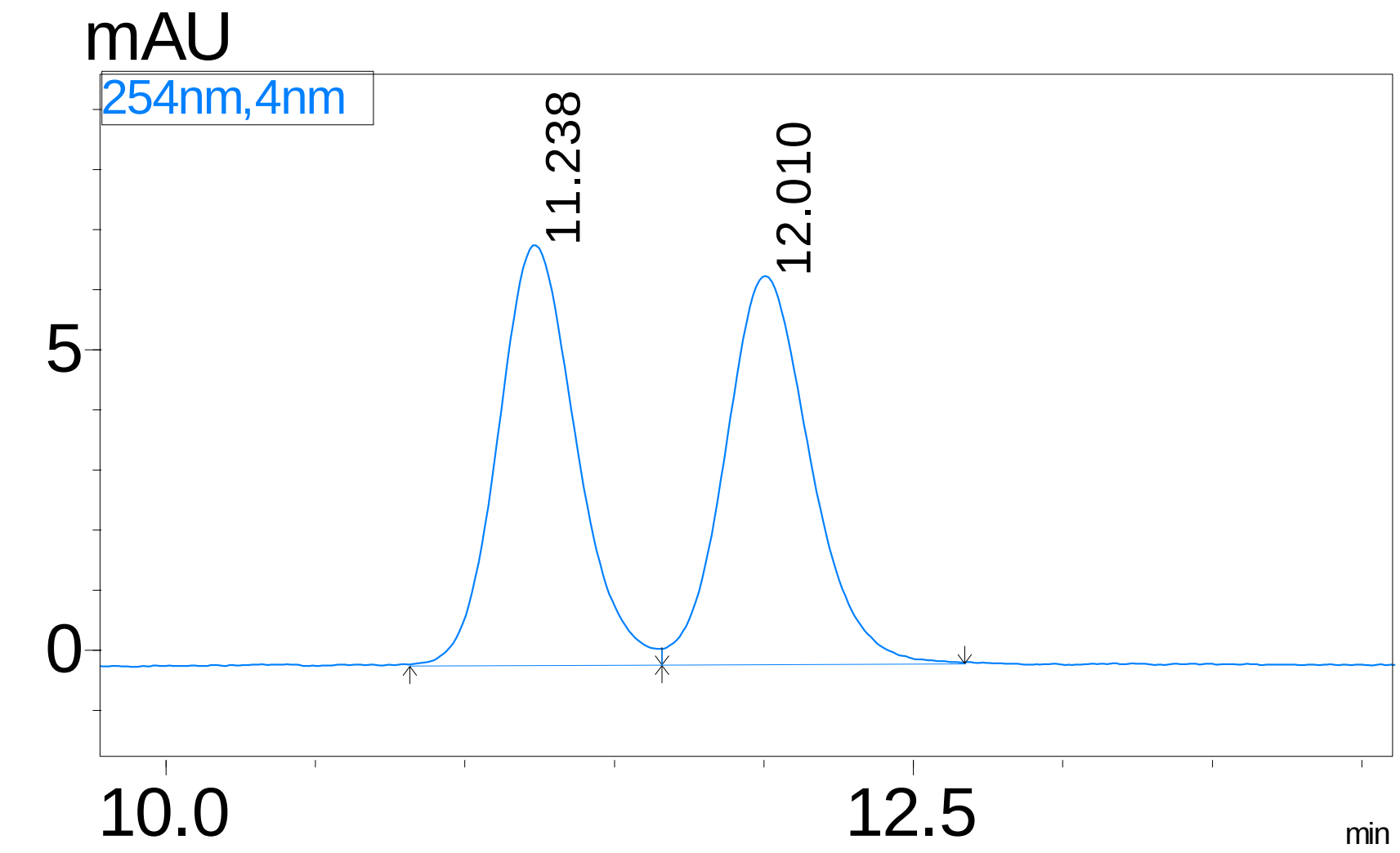

Peak	Time	Area	Height	Area%
1	11.238	126621	6970	49.915
2	12.010	127053	6446	50.085

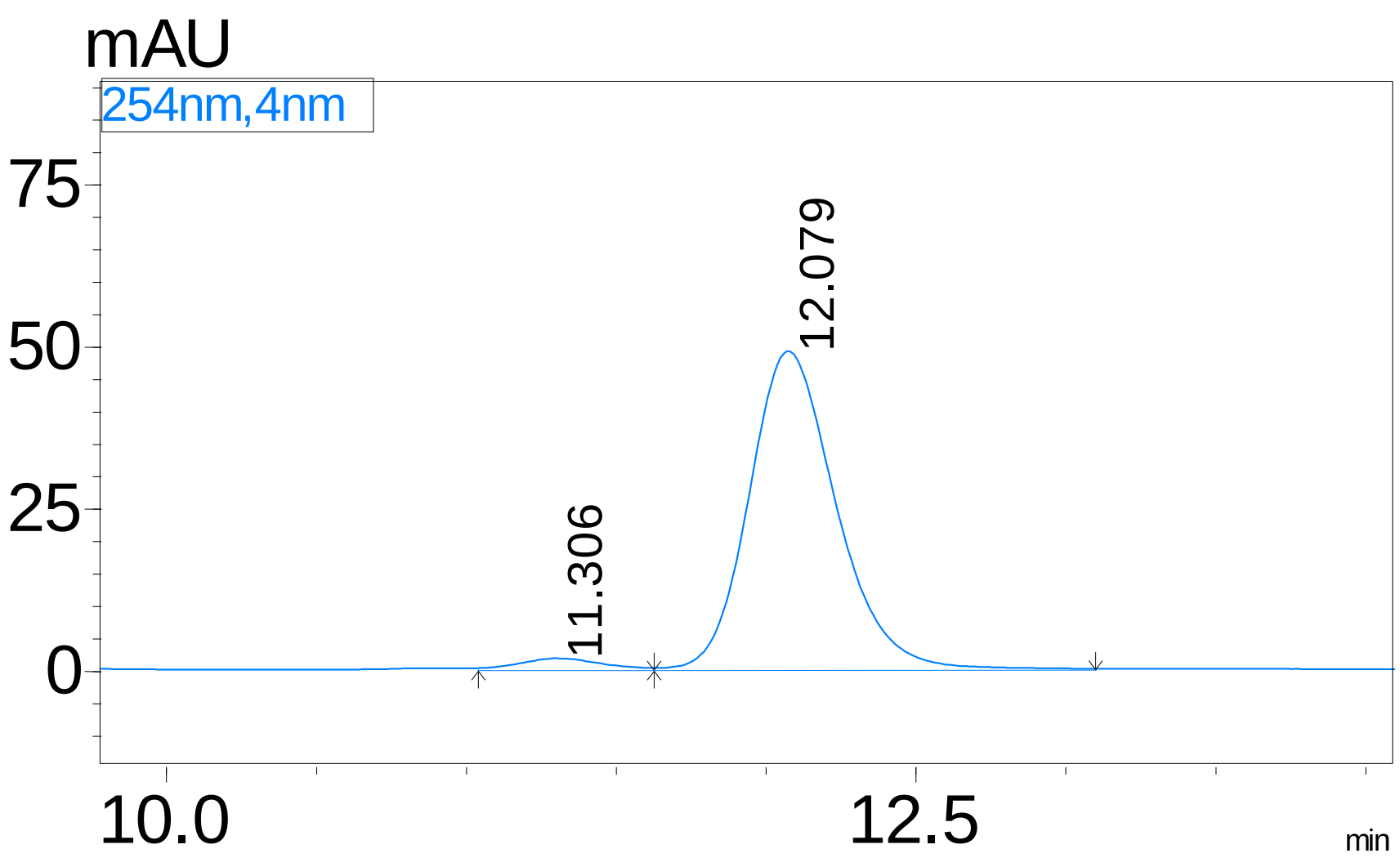

Peak	Time	Area	Height	Area%
1	11.306	29892	1654	2.955
2	12.079	981828	48981	97.045

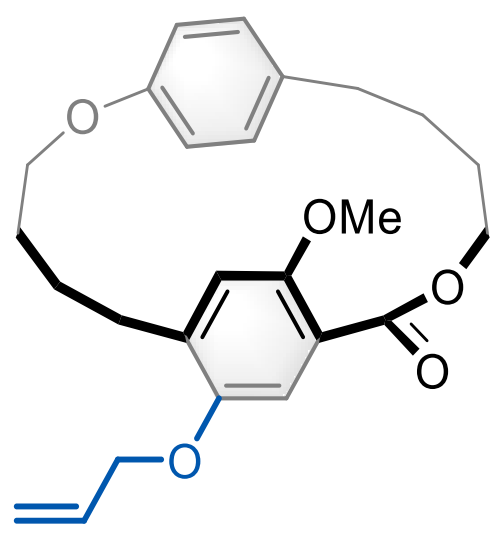

5

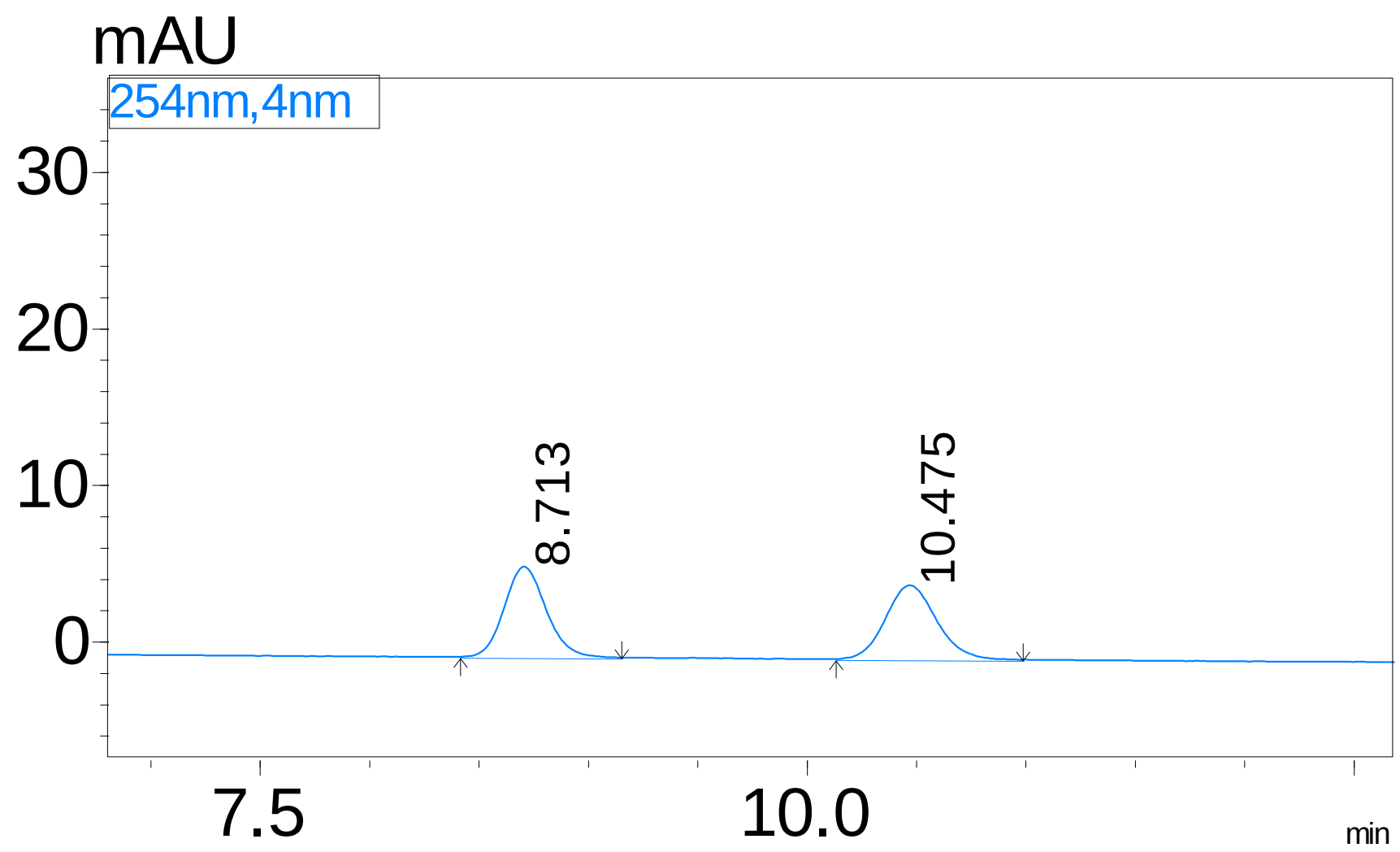

Peak	Time	Area	Height	Area%
1	8.713	76439	5779	49.998
2	10.475	76444	4735	50.002

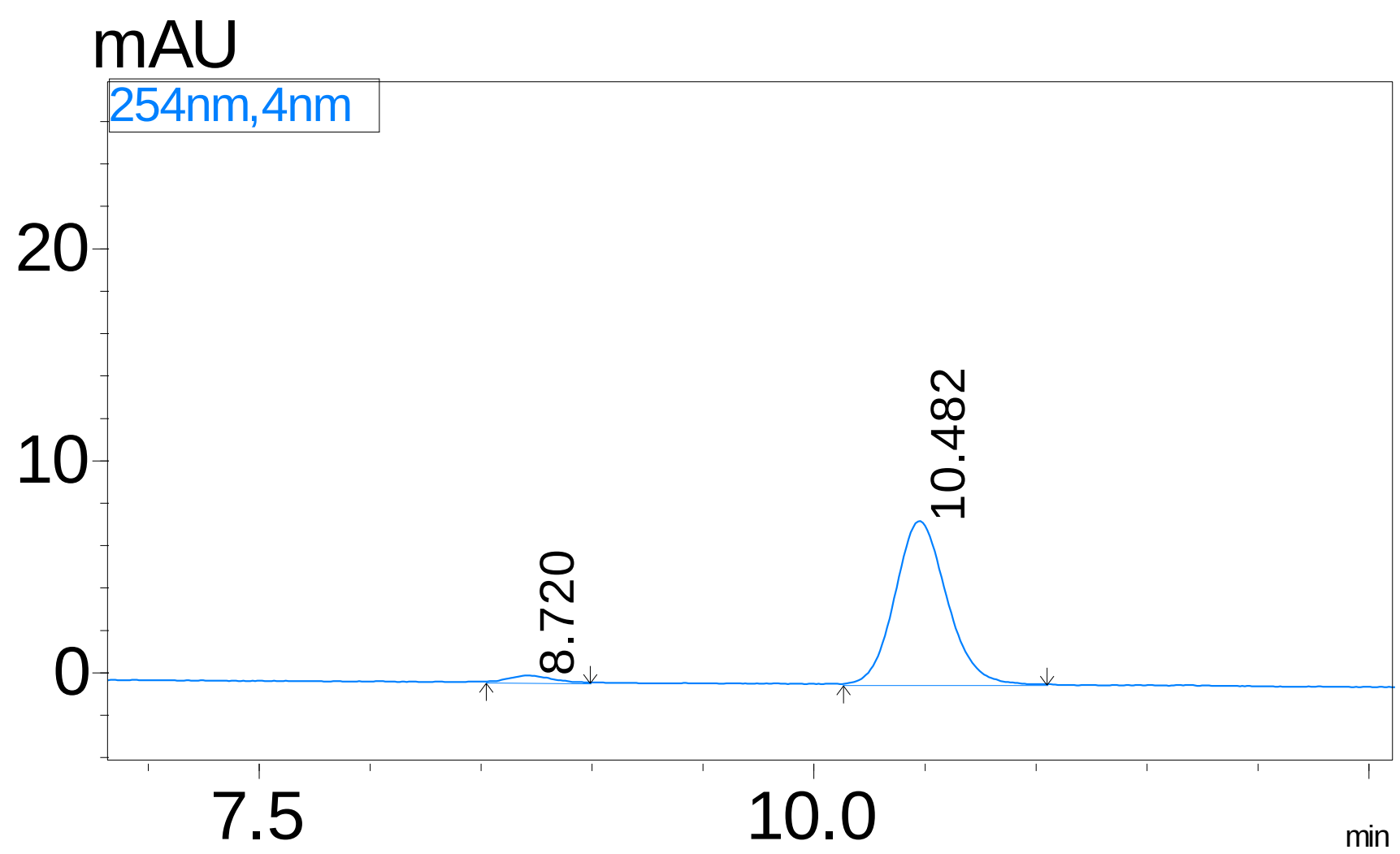

Peak	Time	Area	Height	Area%
1	8.720	3619	303	2.828
2	10.482	124370	7684	97.172

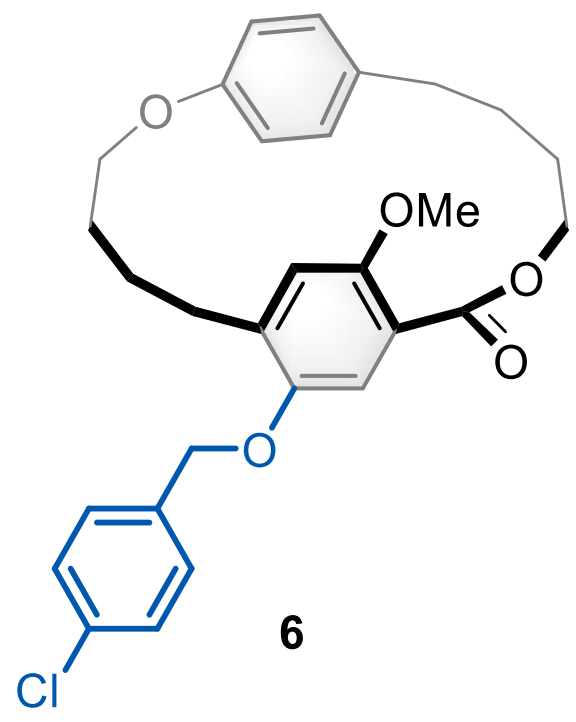

6

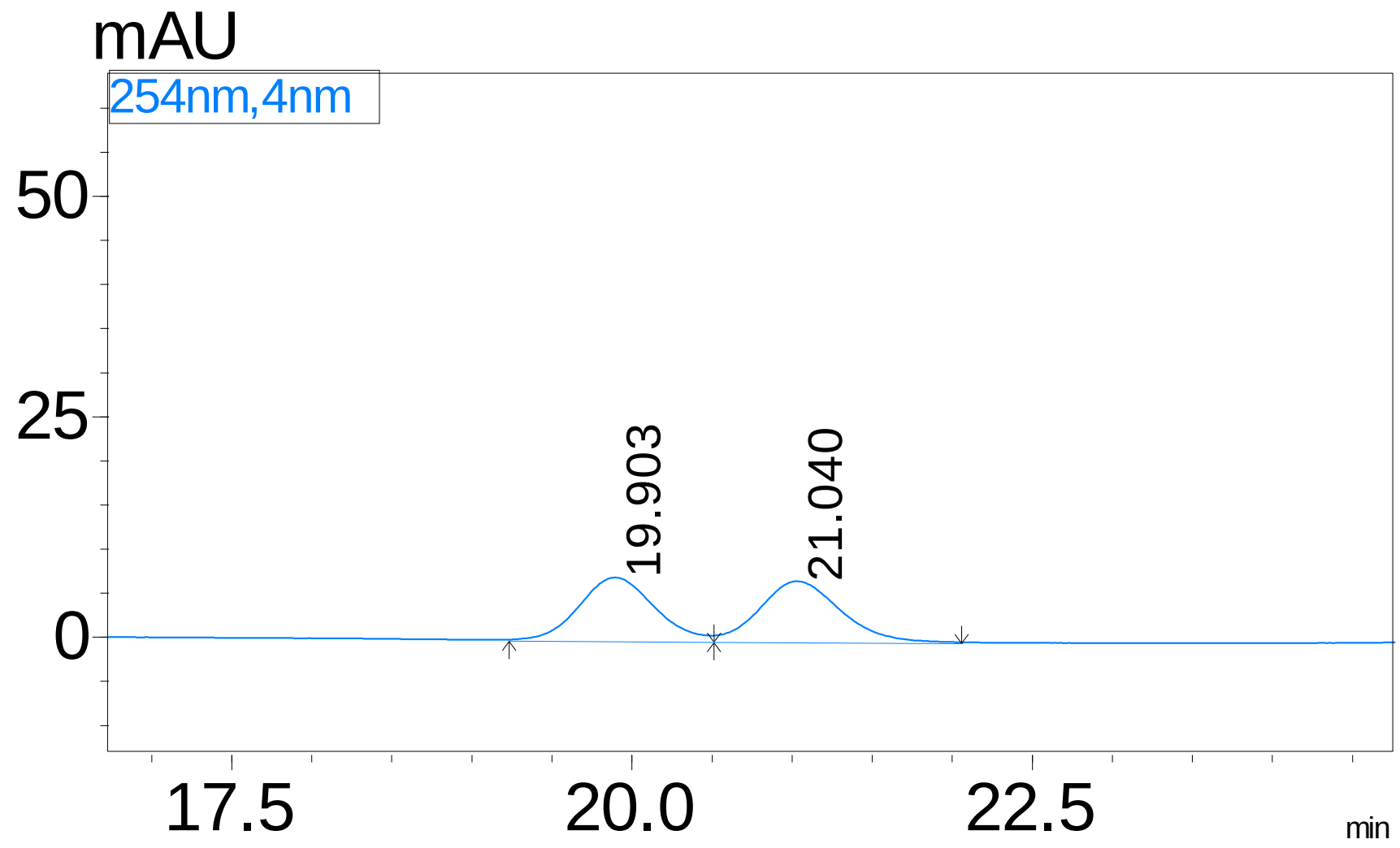

Peak	Time	Area	Height	Area%
1	19.903	229042	7114	49.458
2	21.040	234065	6796	50.542

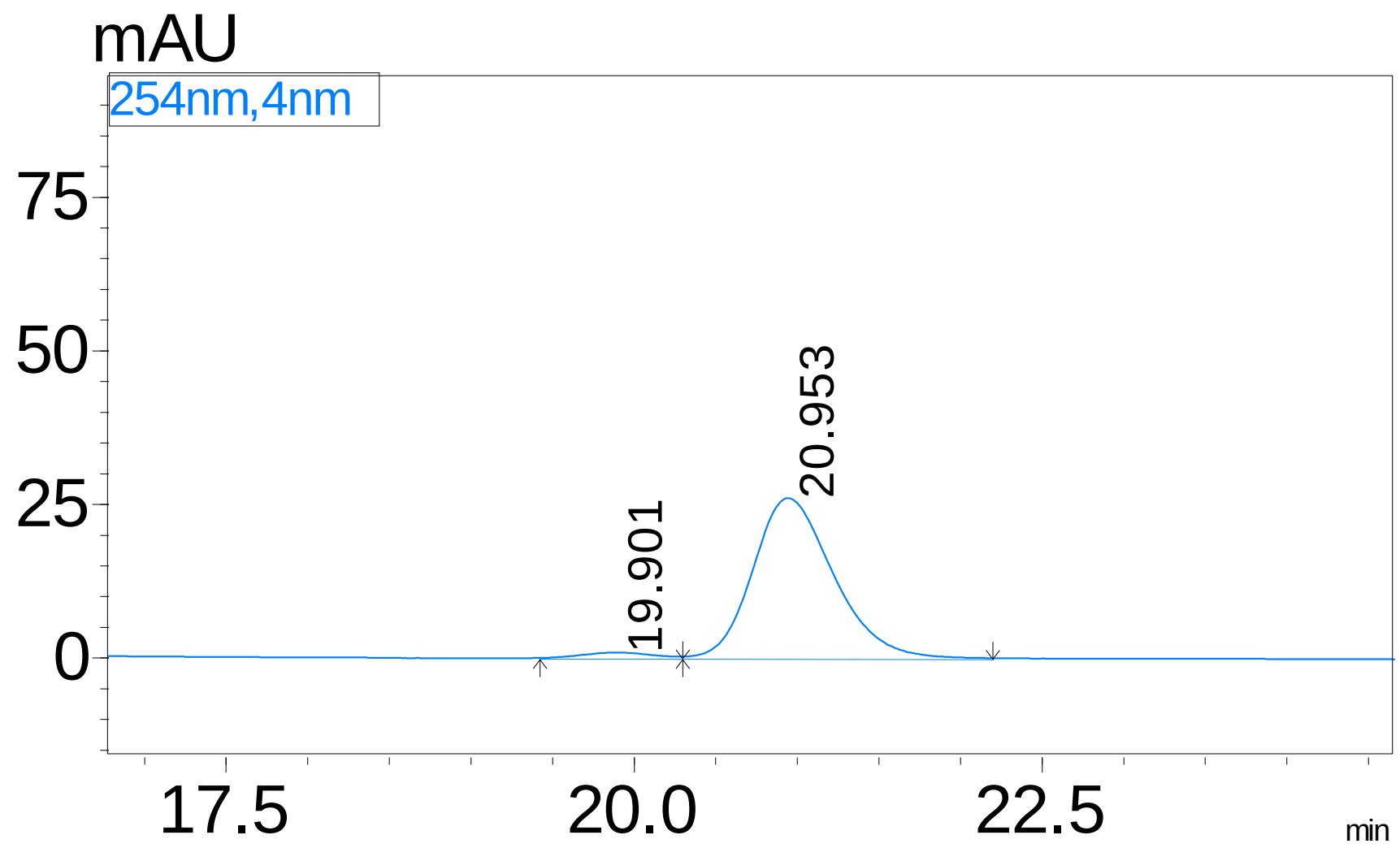

Peak	Time	Area	Height	Area%
1	19.901	25549	901	2.788
2	20.953	890991	26038	97.212

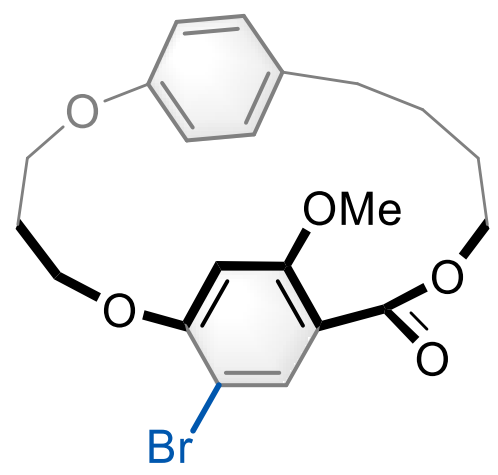

7

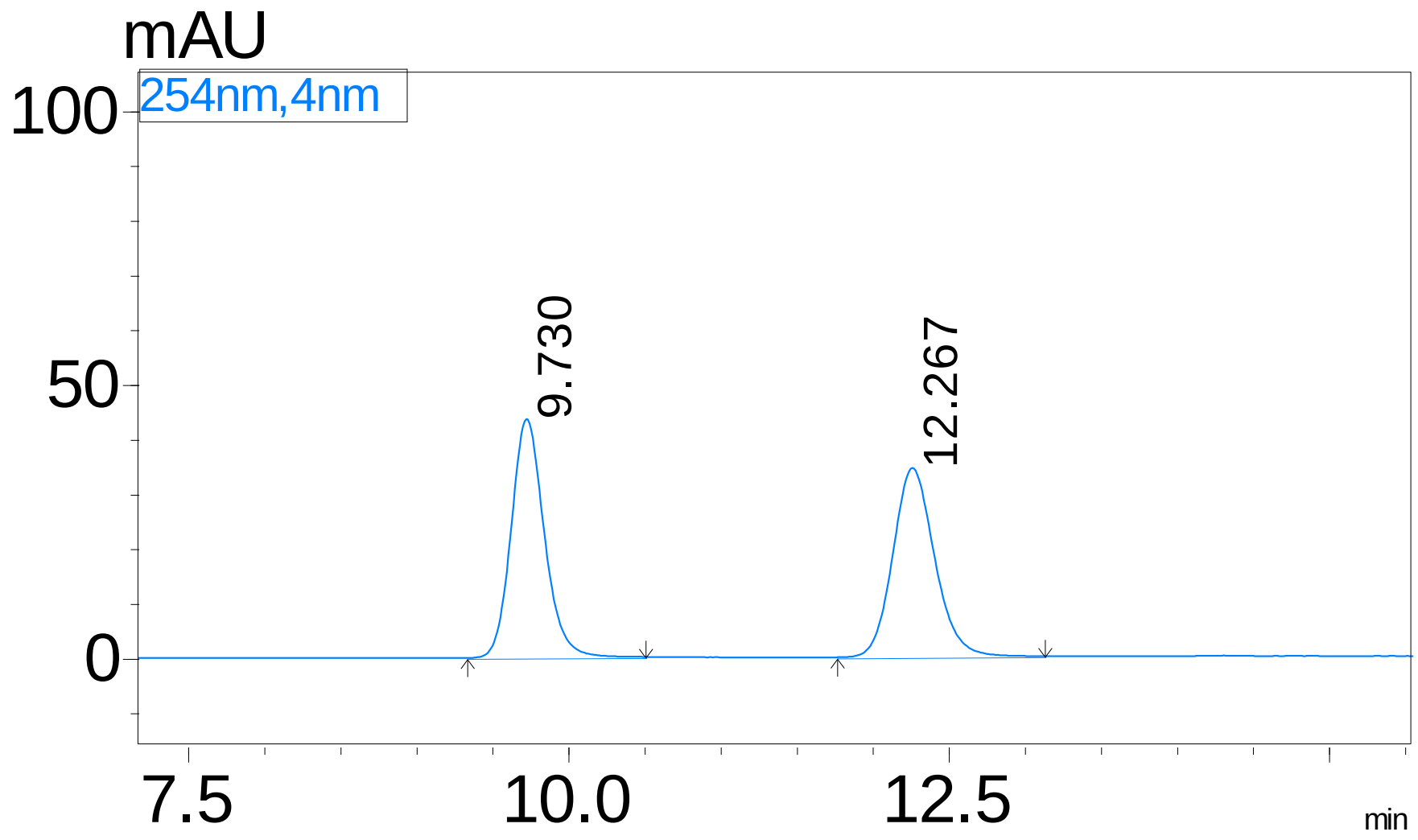

Peak	Time	Area	Height	Area%
1	9.730	658497	43610	50.015
2	12.267	658112	34506	49.985

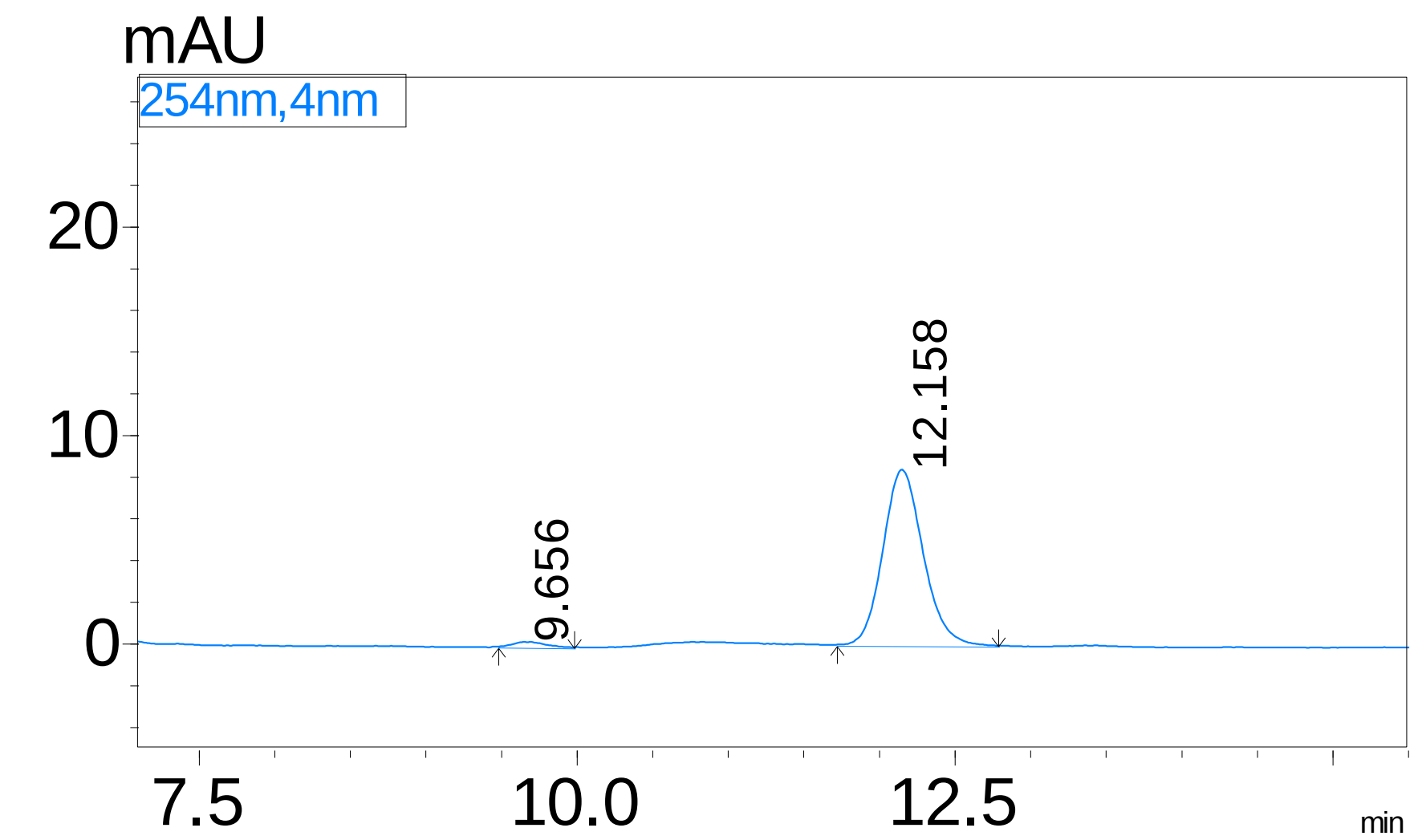

Peak	Time	Area	Height	Area%
1	9.656	3303	232	2.089
2	12.158	154789	8408	97.911

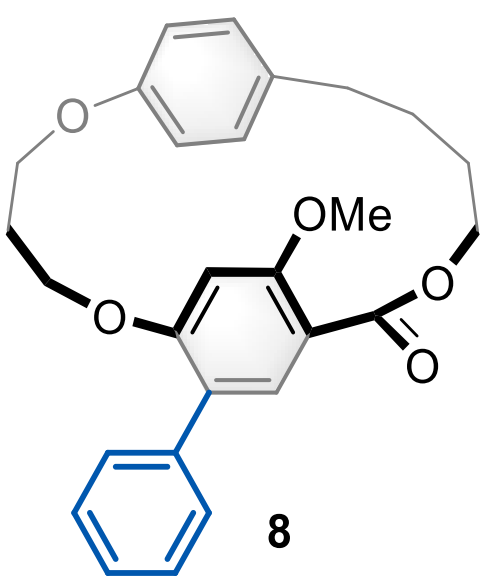

8

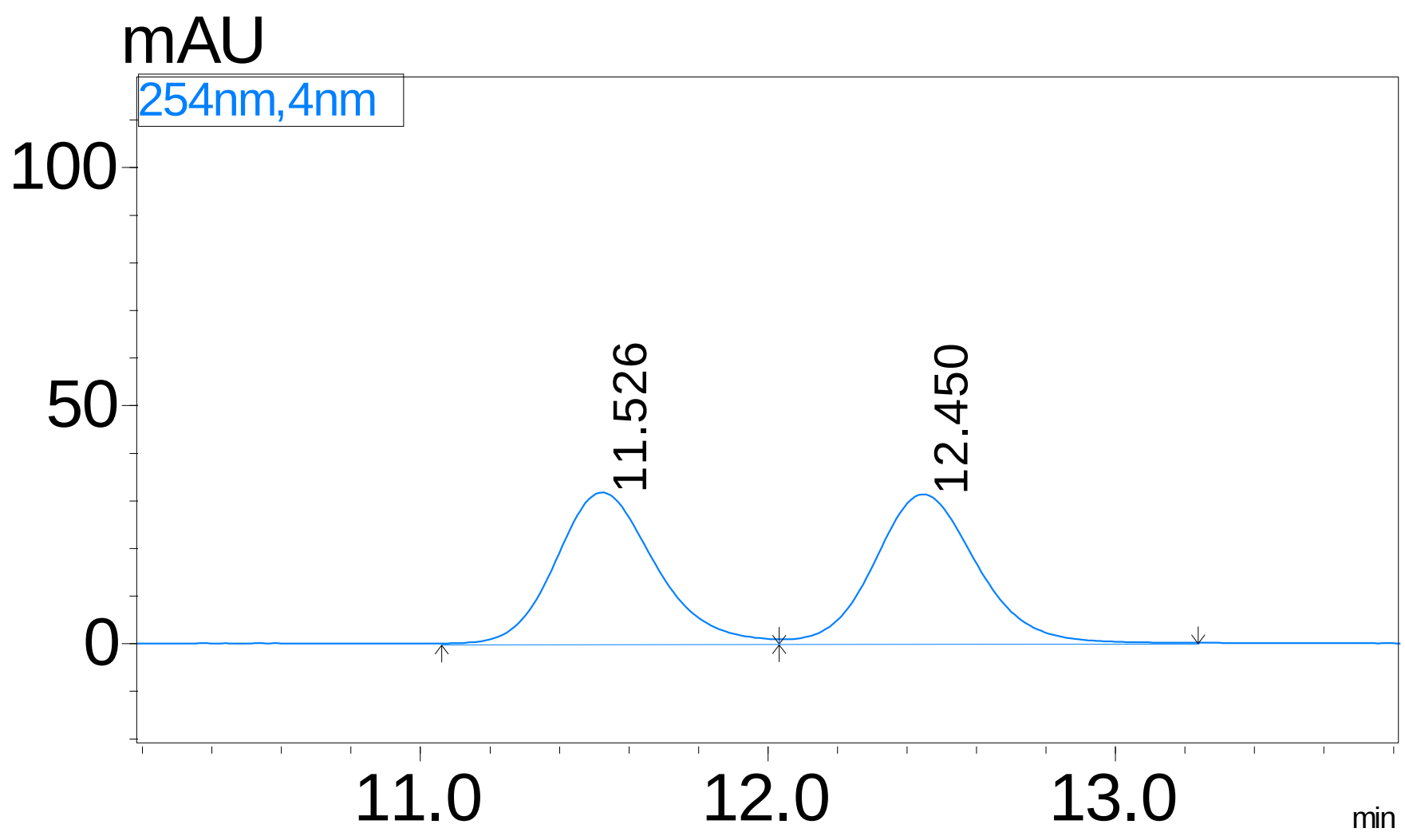

Peak	Time	Area	Height	Area%
1	11.526	630180	31662	49.615
2	12.450	639955	31242	50.385

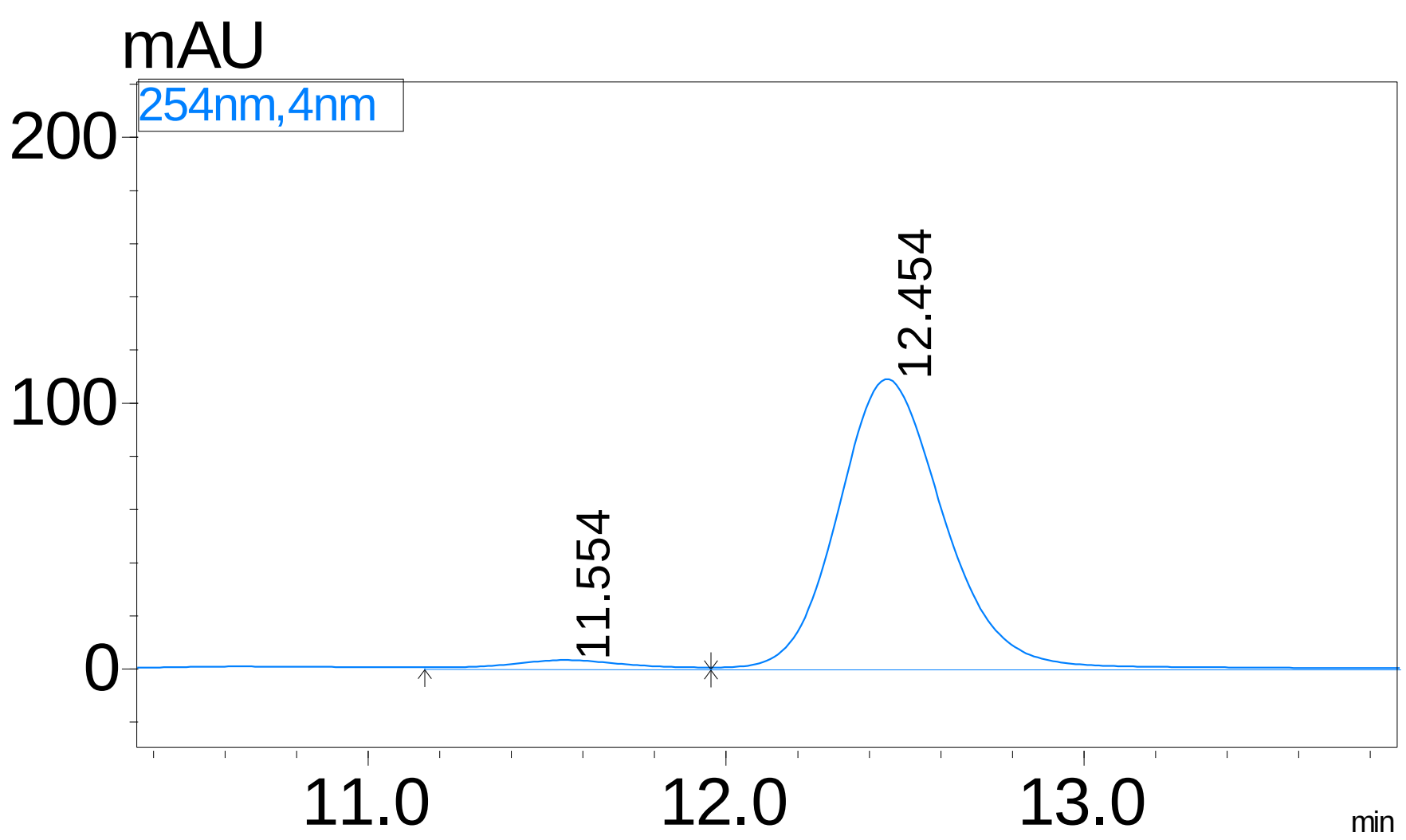

Peak	Time	Area	Height	Area%
1	11.554	66196	3088	2.873
2	12.454	2237721	108693	97.127

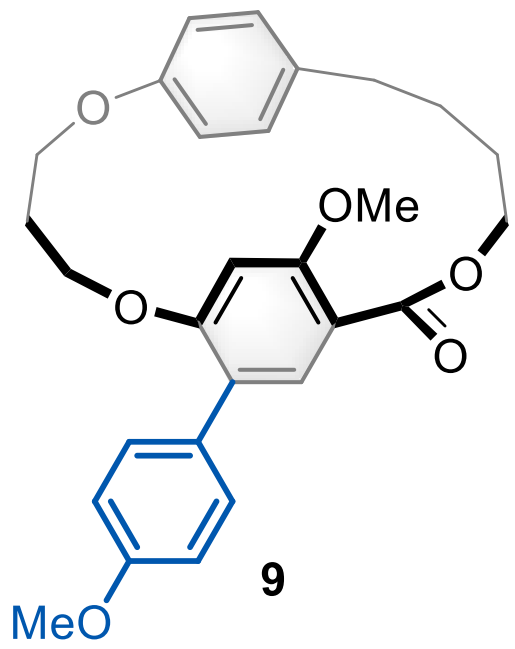

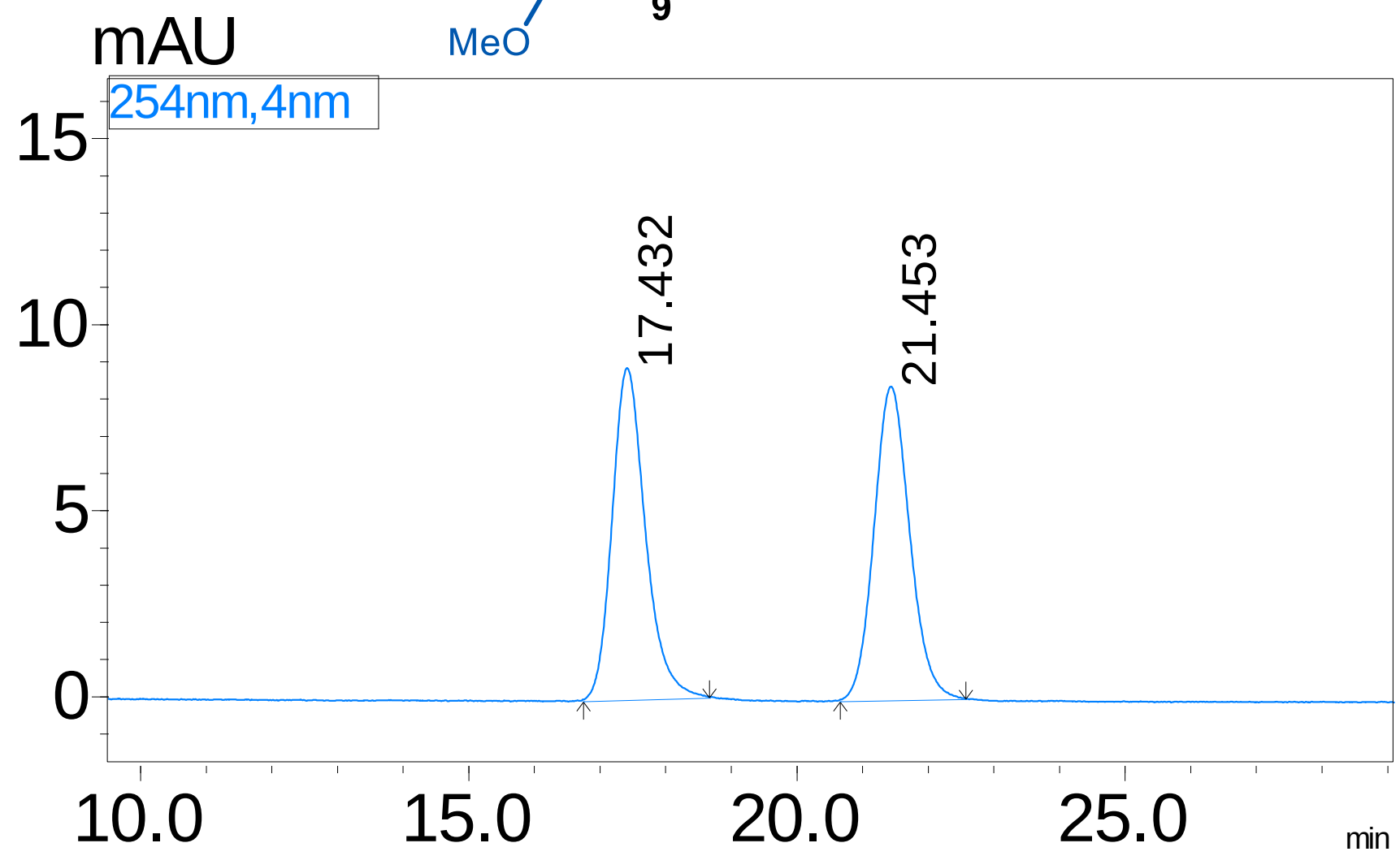

Peak	Time	Area	Height	Area%
1	17.432	312030	8895	49.610
2	21.453	316929	8415	50.390

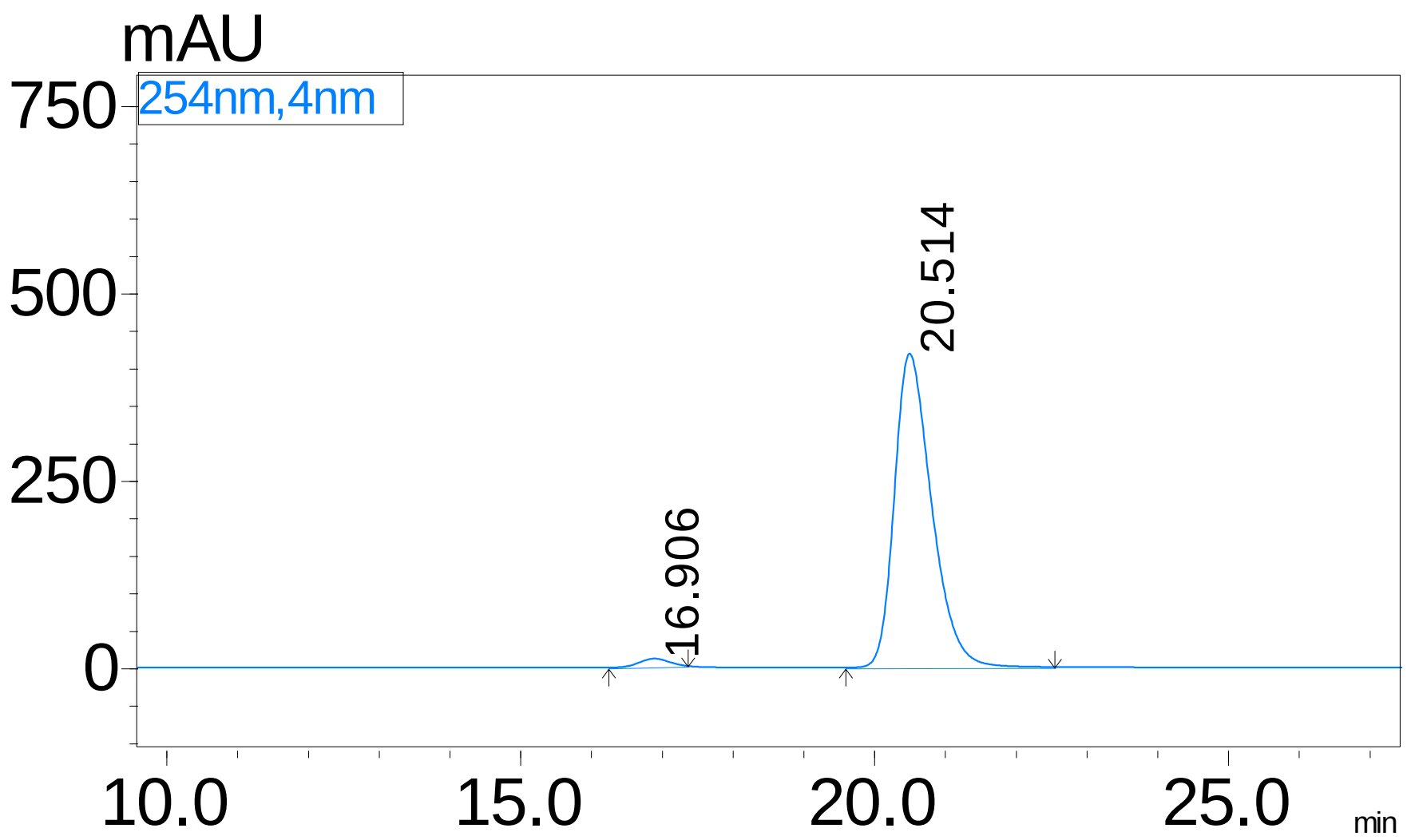

Peak	Time	Area	Height	Area%
1	16.906	297968	10594	1.916
2	20.514	15255353	418493	98.084

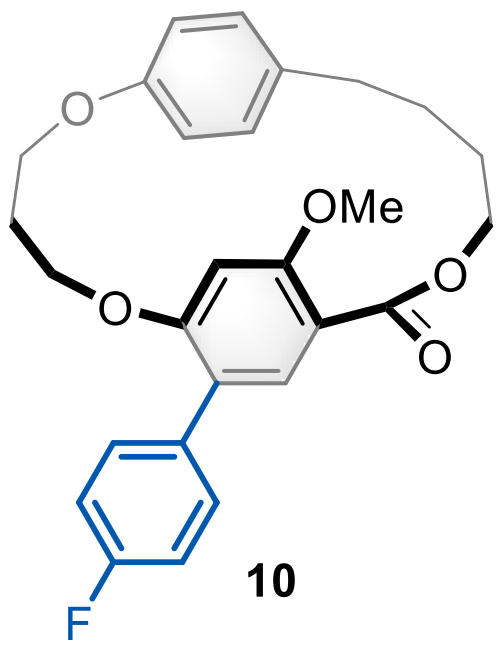

10

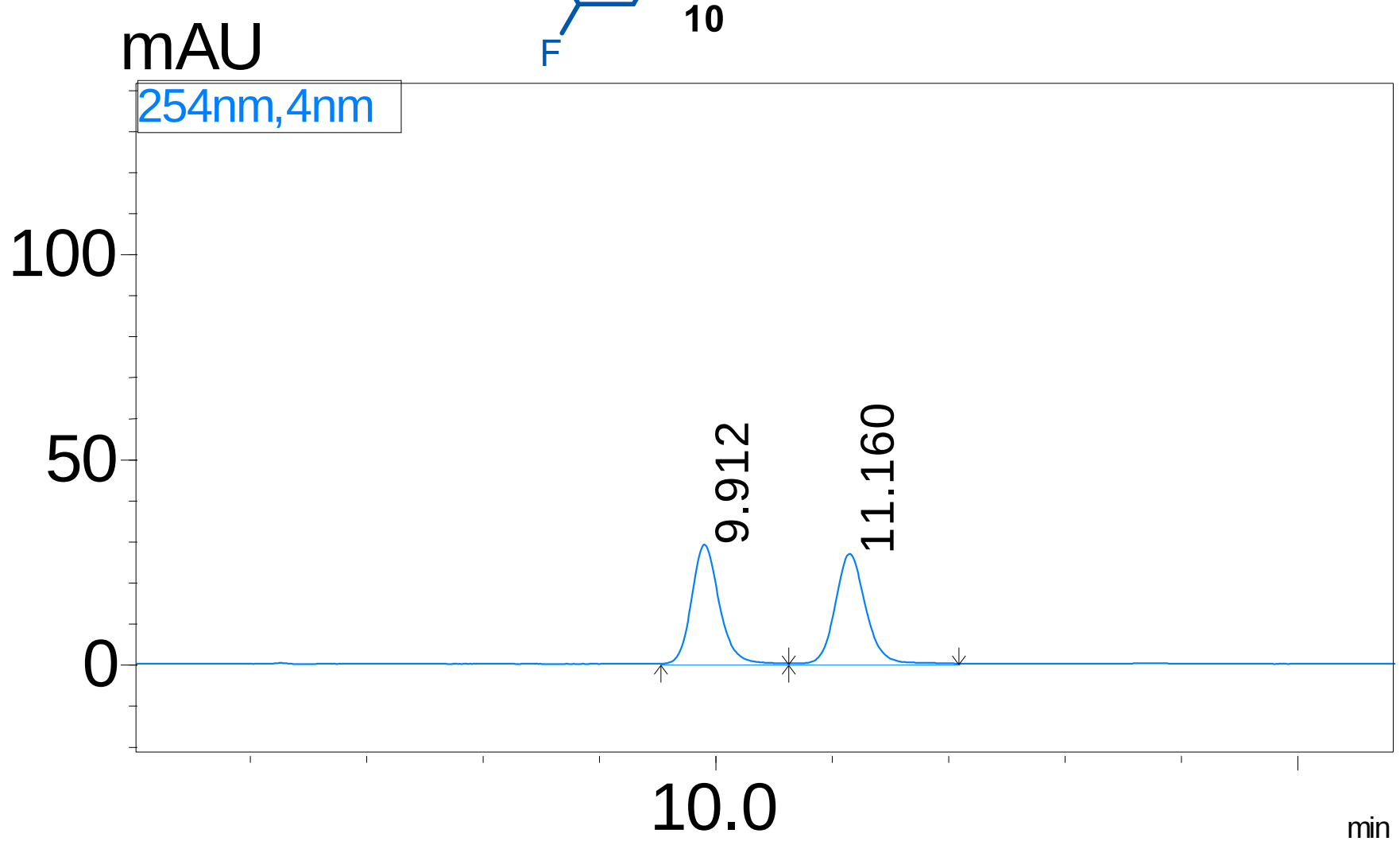

Peak	Time	Area	Height	Area%
1	9.912	476768	29036	49.759
2	11.160	481385	26728	50.241

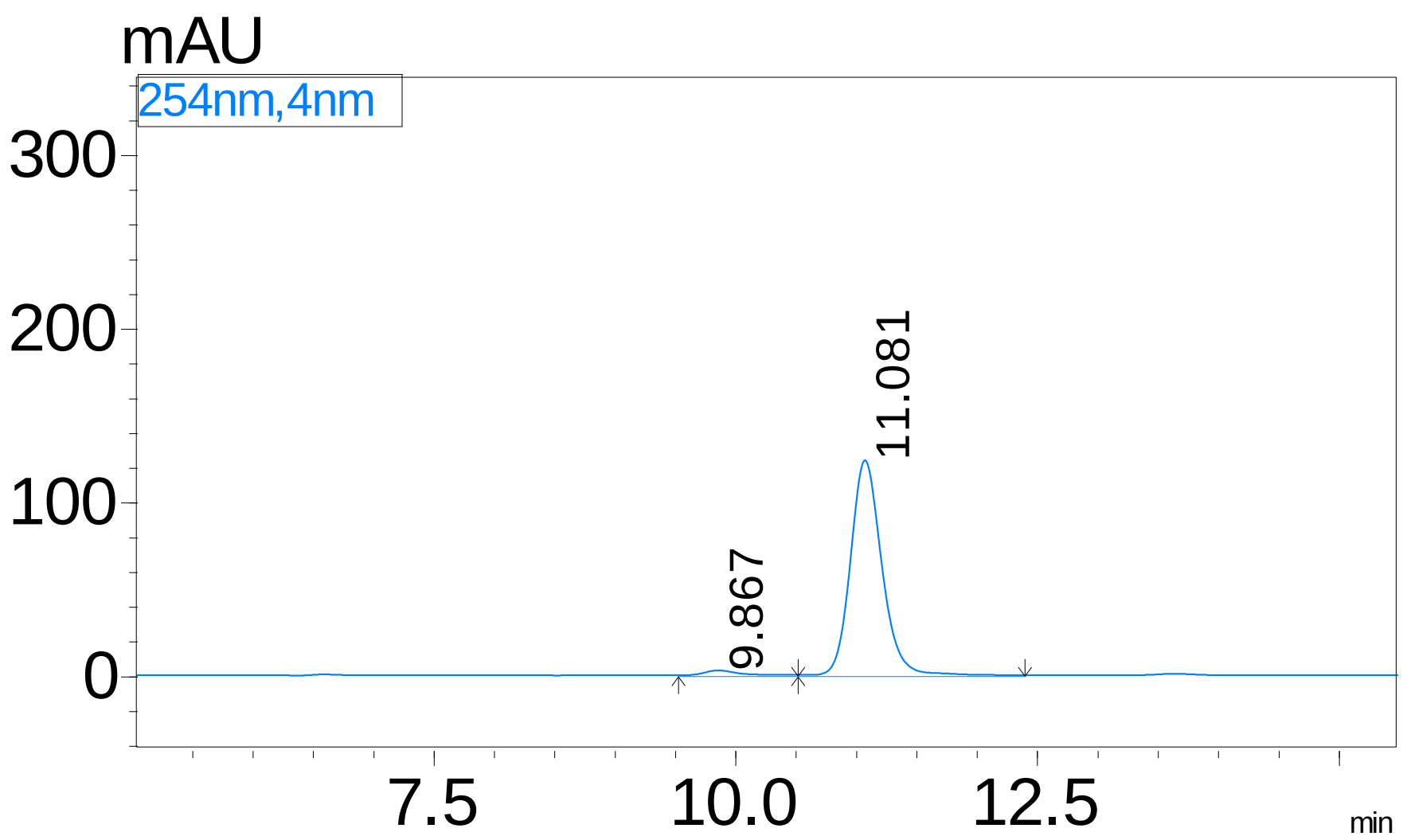

Peak	Time	Area	Height	Area%
1	9.867	55518	2838	2.426
2	11.081	2233423	123887	97.574

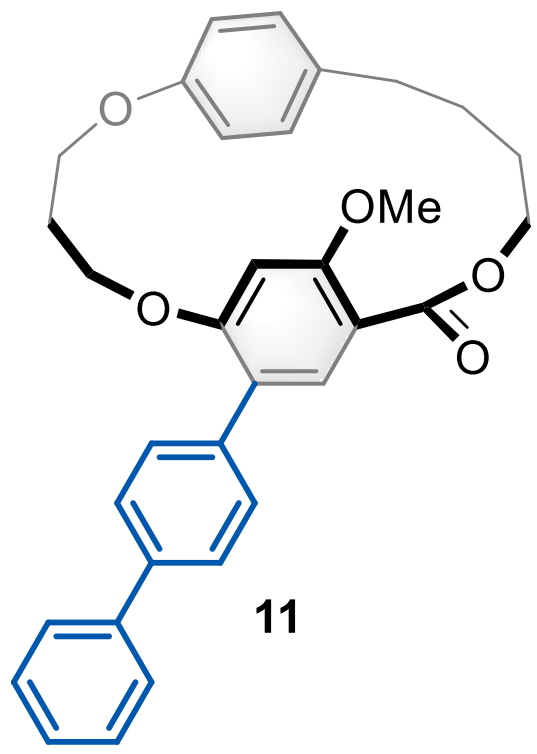

11

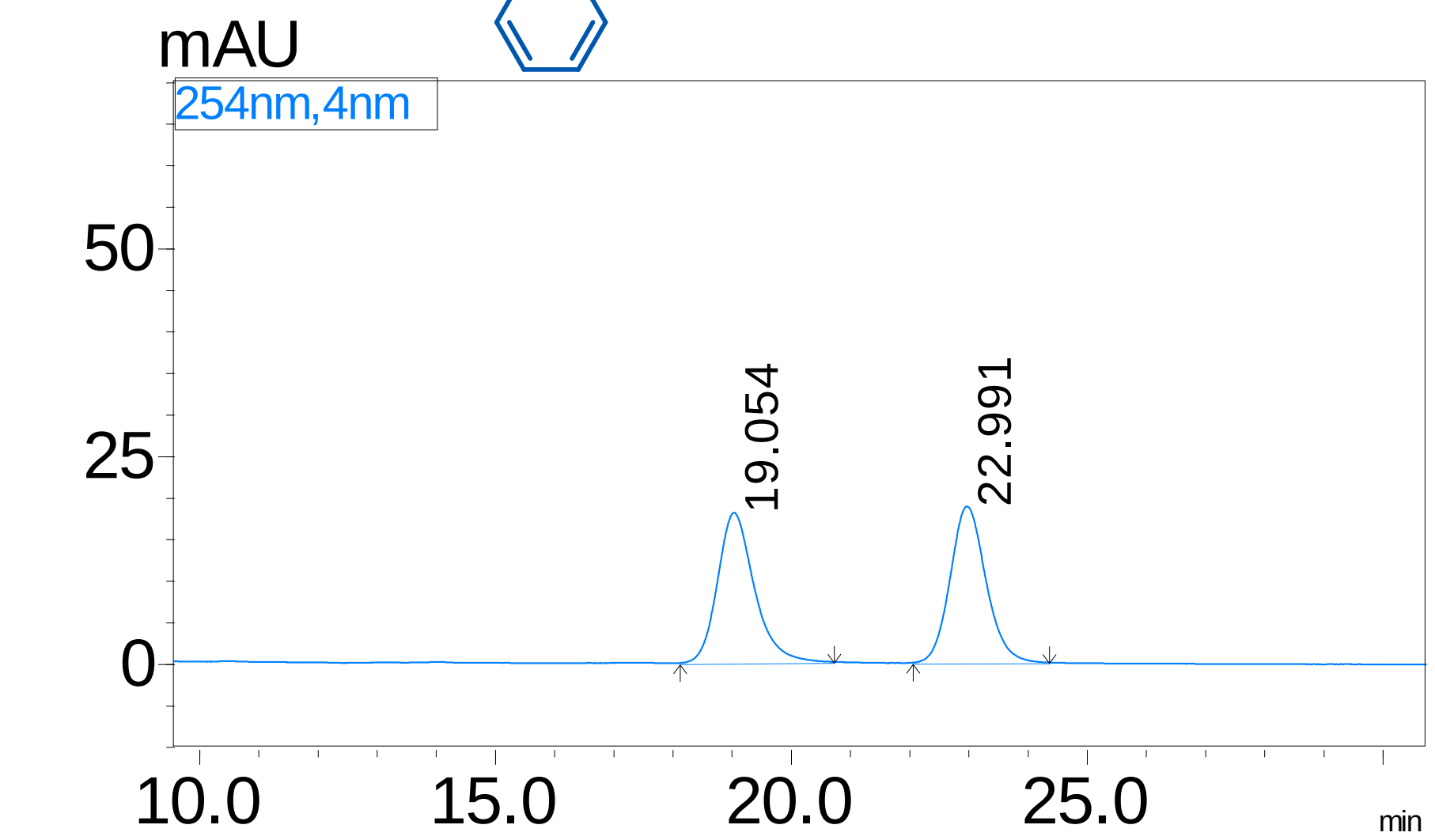

Peak	Time	Area	Height	Area%
1	19.054	777296	18043	49.646
2	22.991	788367	18801	50.354

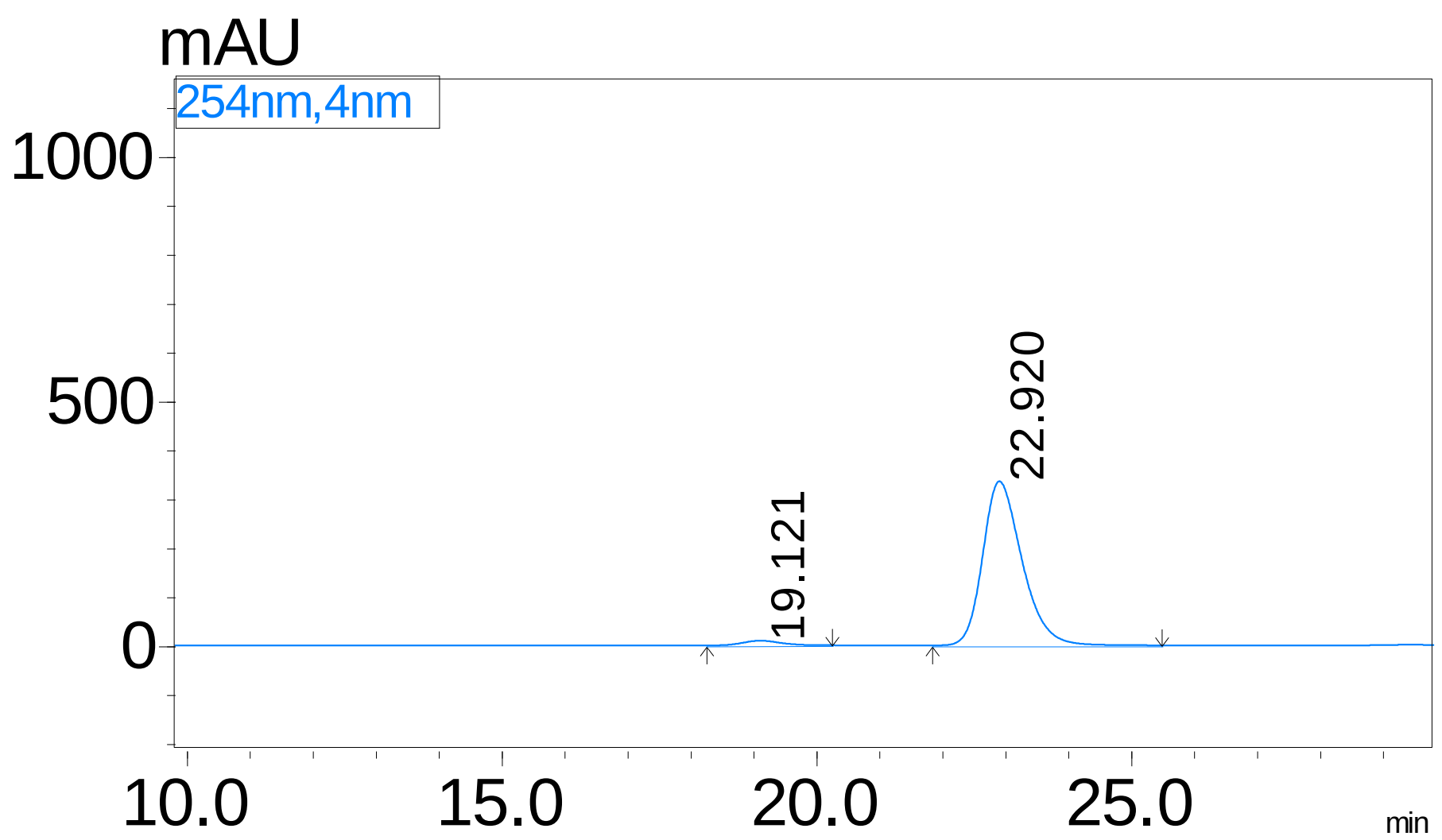

Peak	Time	Area	Height	Area%
1	19.121	398209	9519	2.663
2	22.920	14556147	335444	97.337

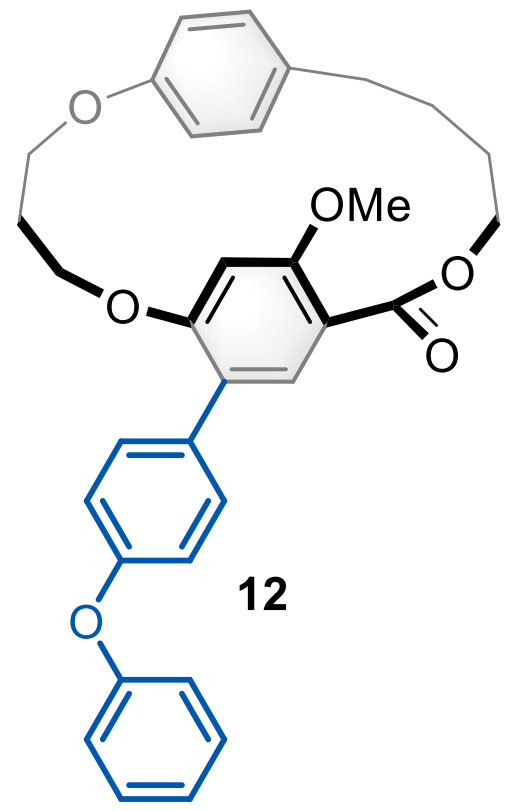

12

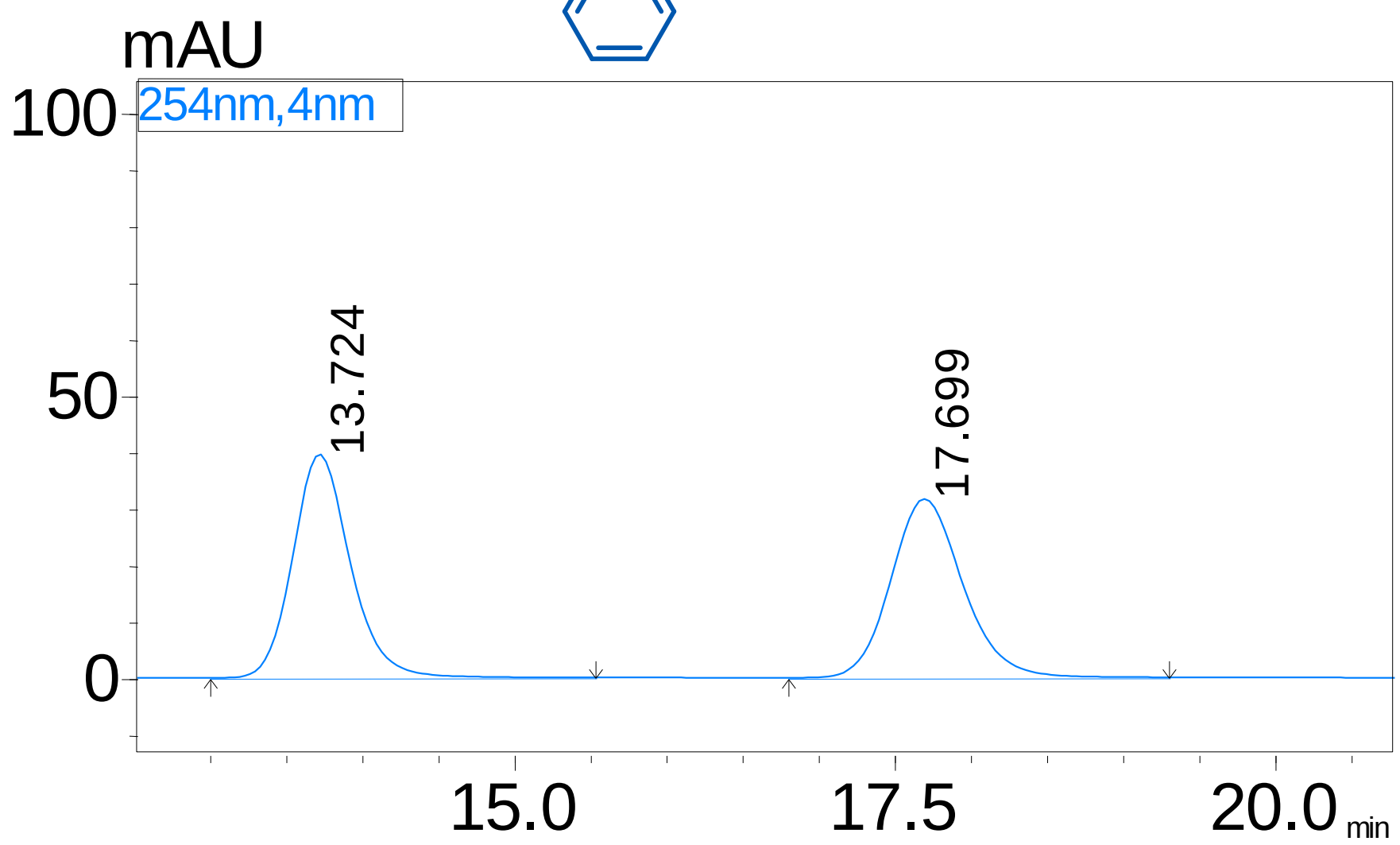

Peak	Time	Area	Height	Area%
1	13.724	1042641	39492	50.088
2	17.699	1038997	31631	49.912

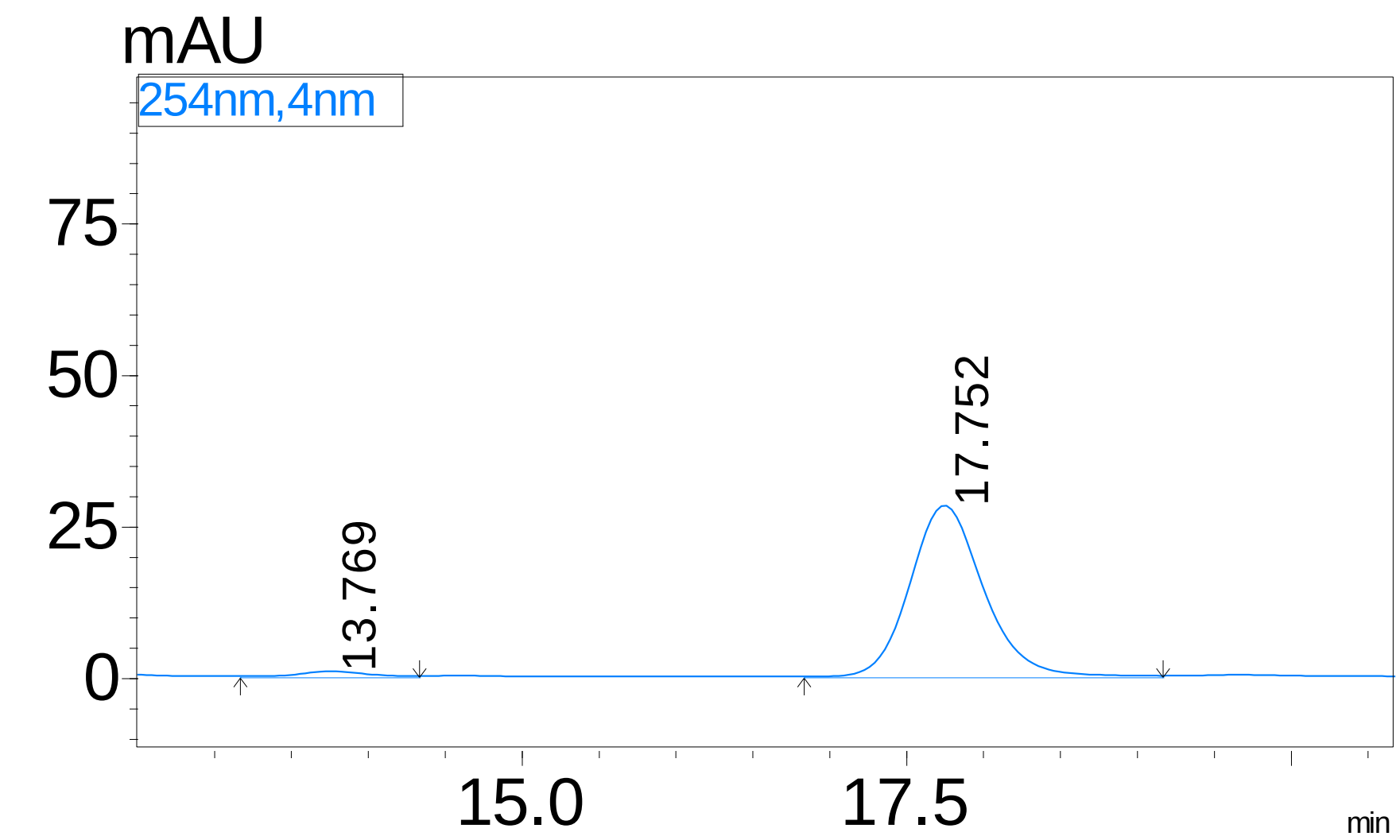

Peak	Time	Area	Height	Area%
1	13.769	24077	854	2.514
2	17.752	933698	28141	97.486

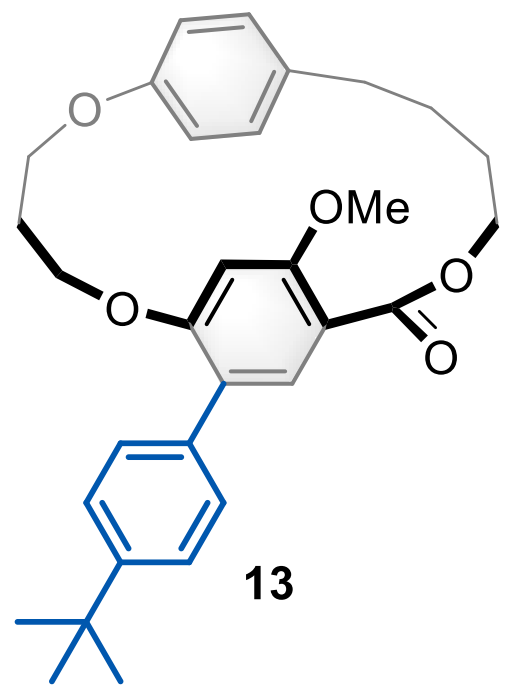

13

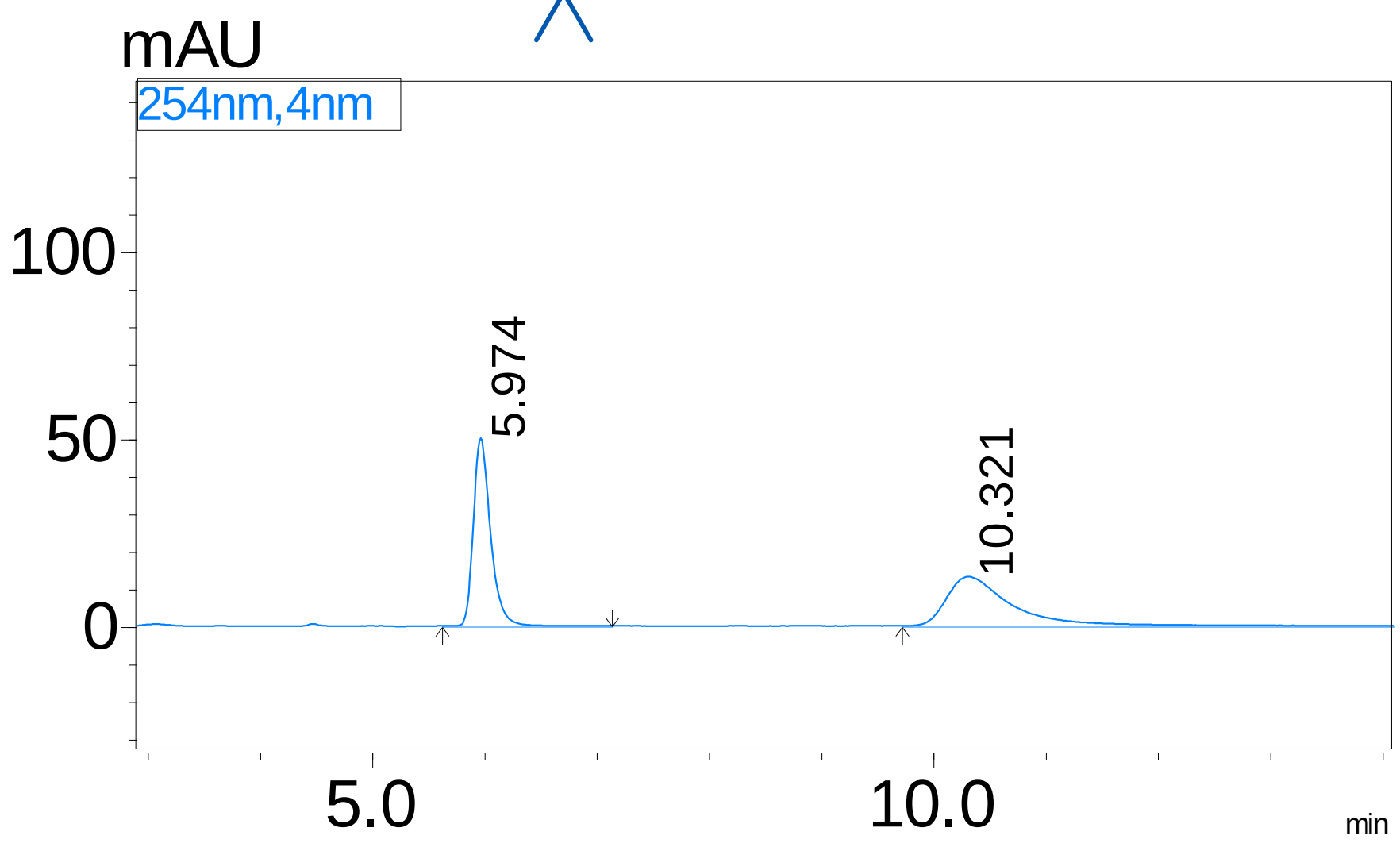

Peak	Time	Area	Height	Area%
1	5.974	529468	50044	49.882
2	10.321	531971	13112	50.118

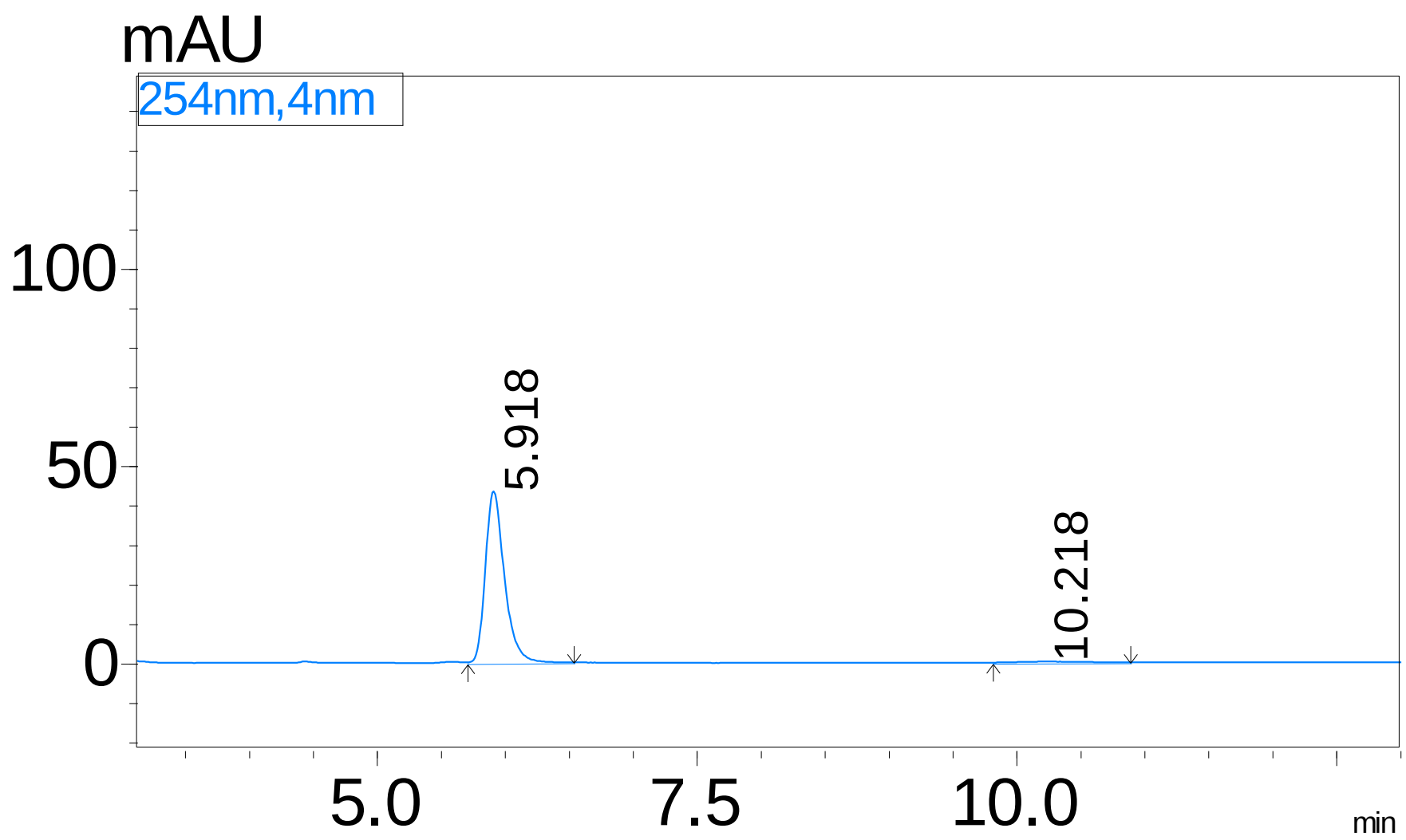

Peak	Time	Area	Height	Area%
1	5.918	450981	43509	98.358
2	10.218	7531	230	1.642

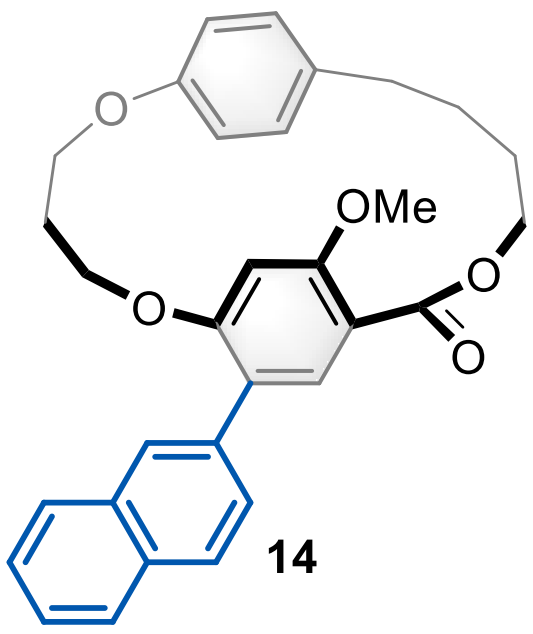

14

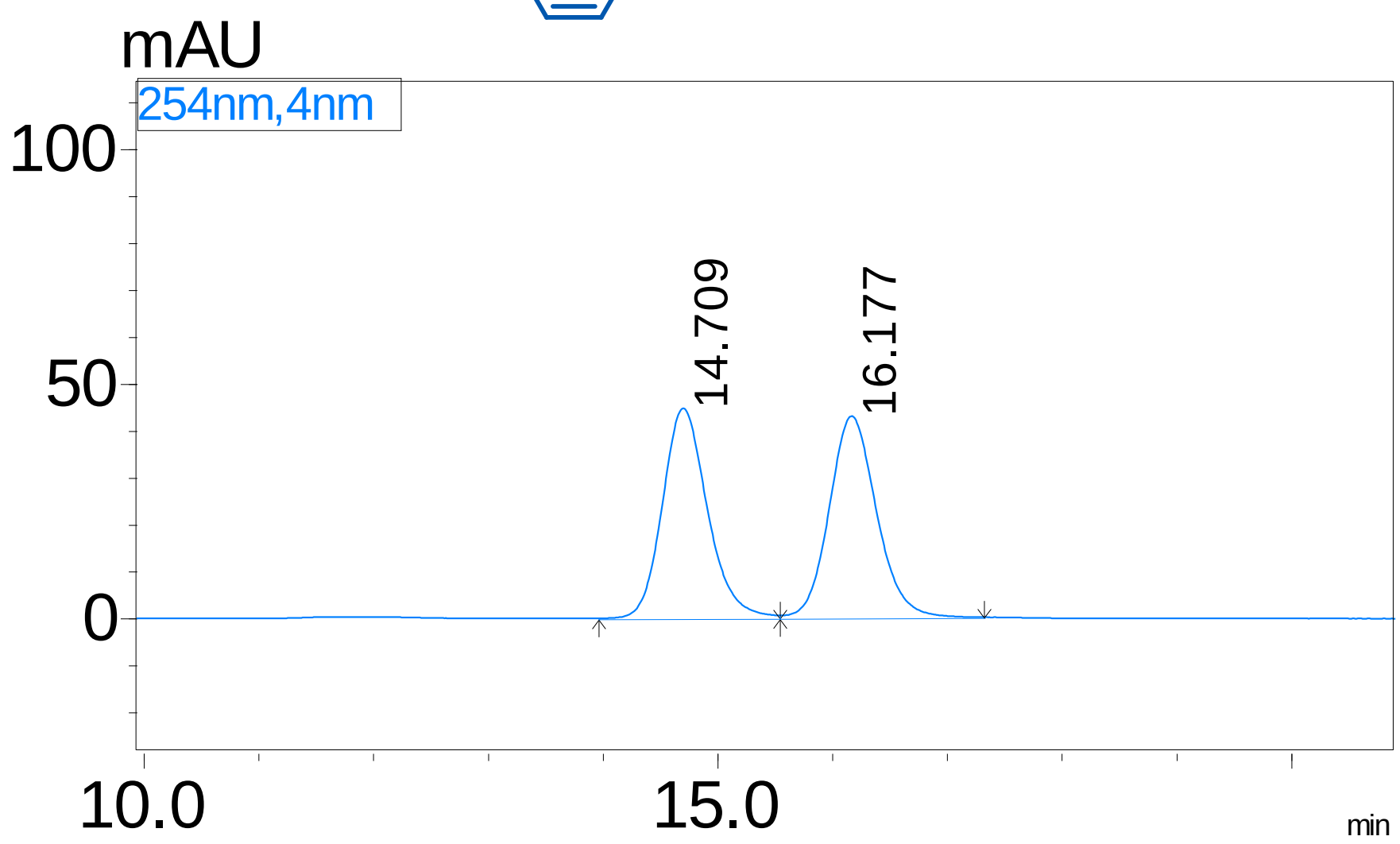

Peak	Time	Area	Height	Area%
1	16.906	297968	10594	1.916
2	20.514	15255353	418493	98.084

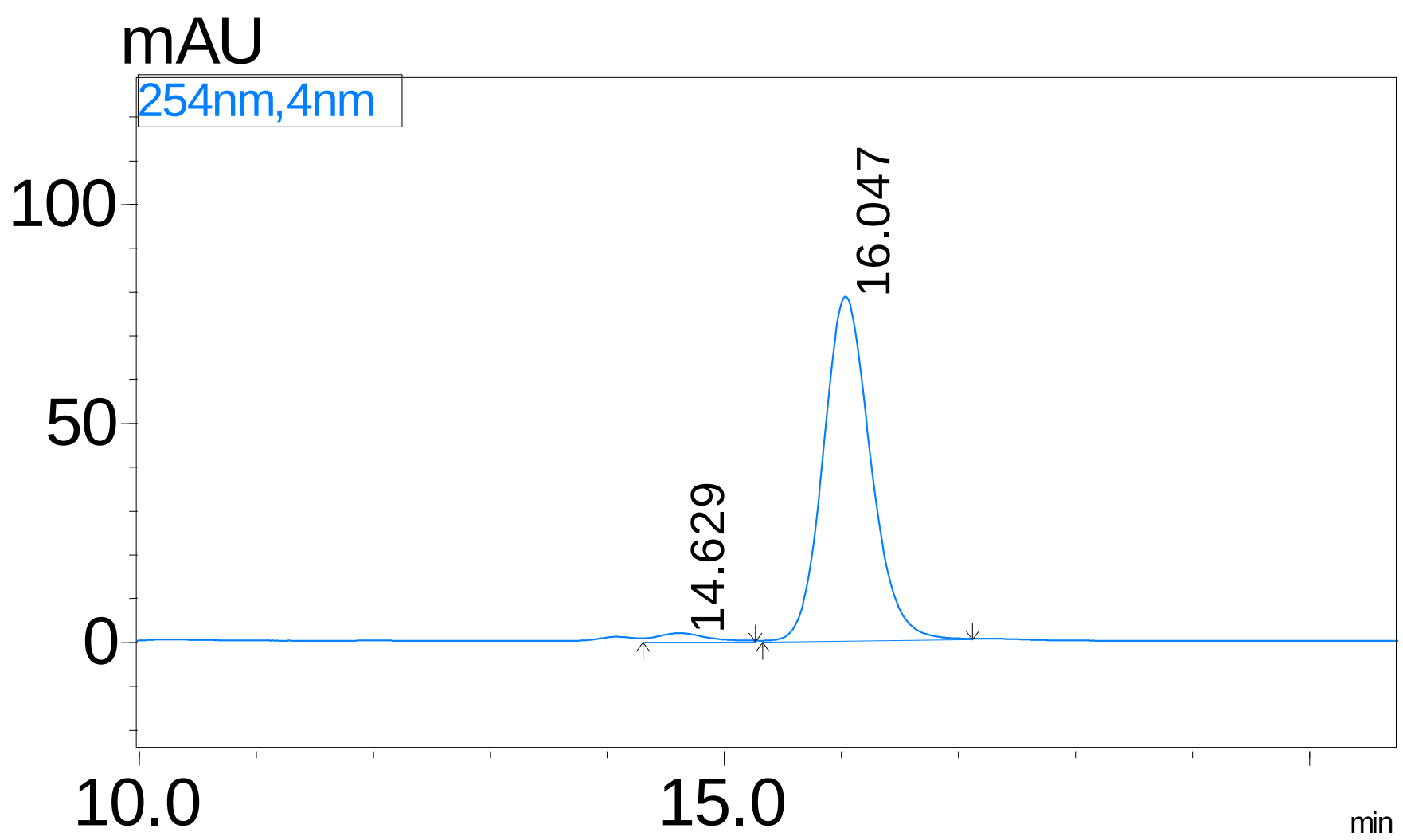

Peak	Time	Area	Height	Area%
1	14.629	47101	1766	2.099
2	16.047	2197071	78371	97.901

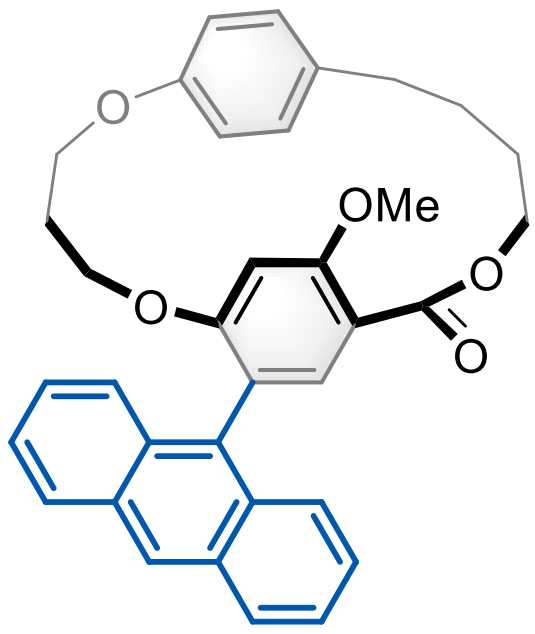

15

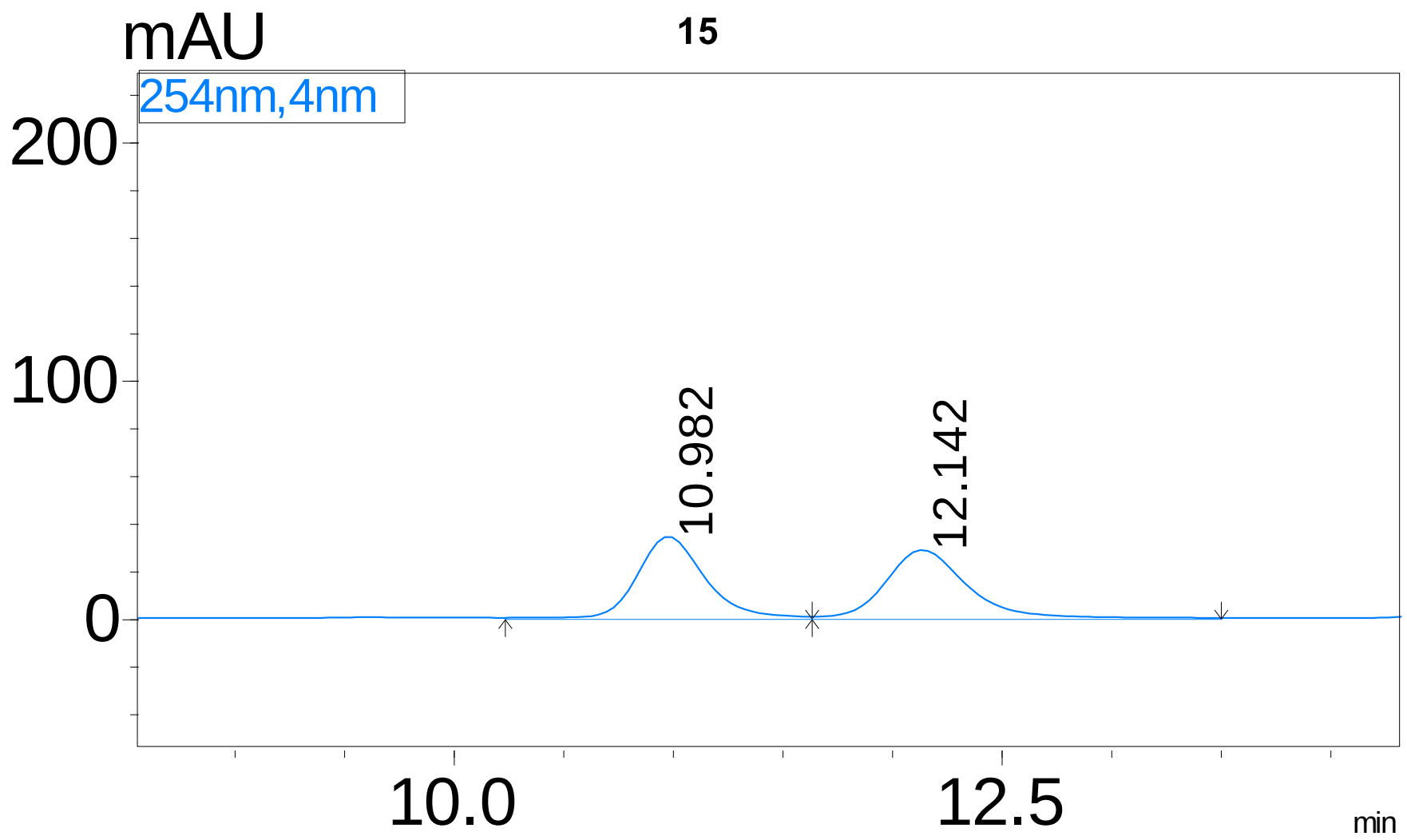

Peak	Time	Area	Height	Area%
1	10.982	715725	33945	49.943
2	12.142	717360	28509	50.057

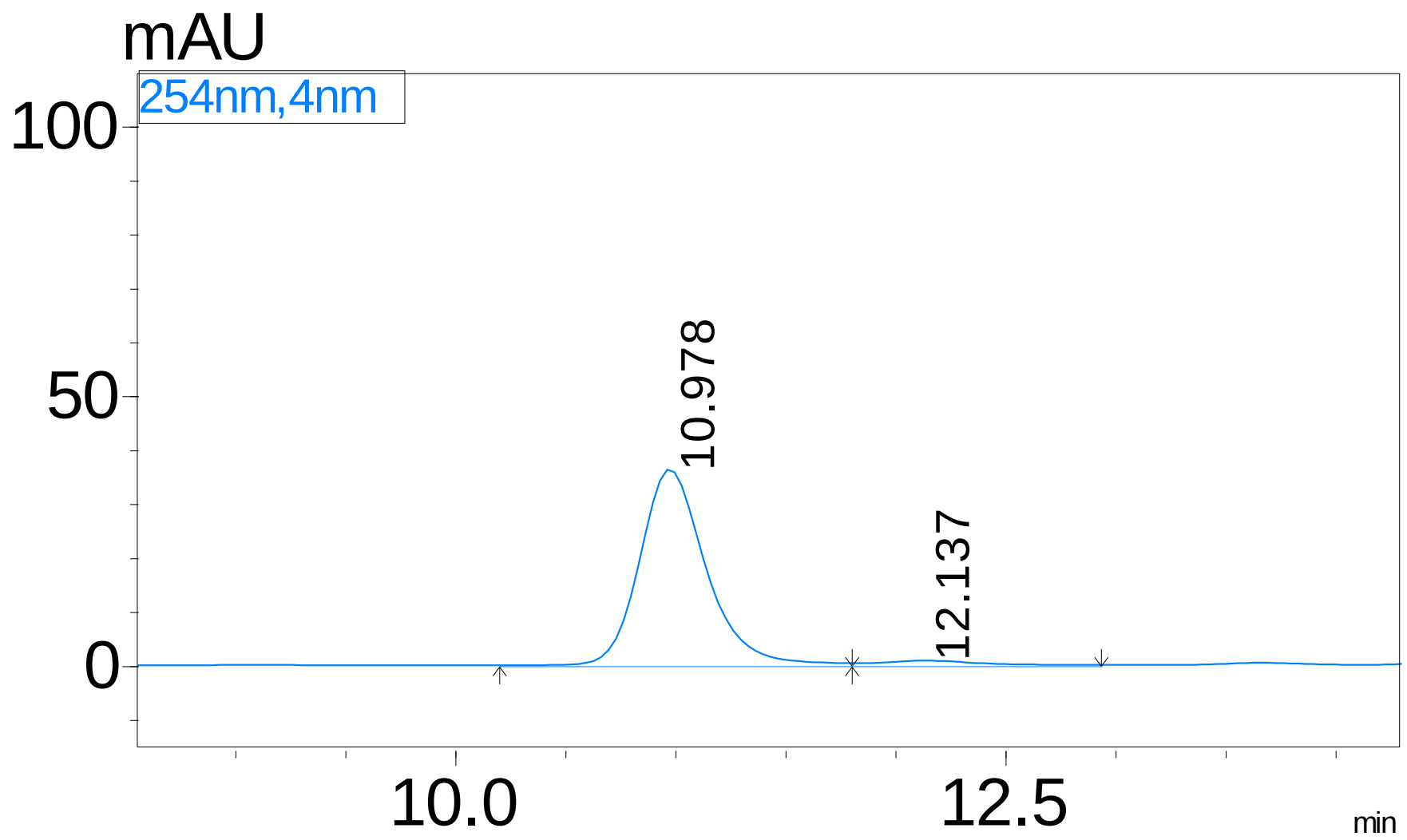

Peak	Time	Area	Height	Area%
1	10.978	760170	36220	96.806
2	12.137	25079	857	3.194

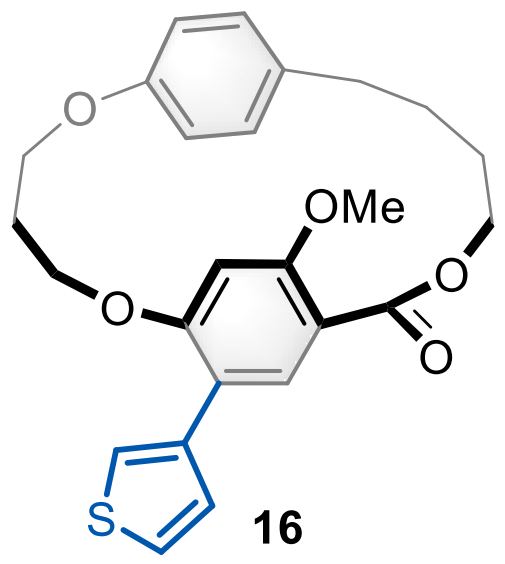

16

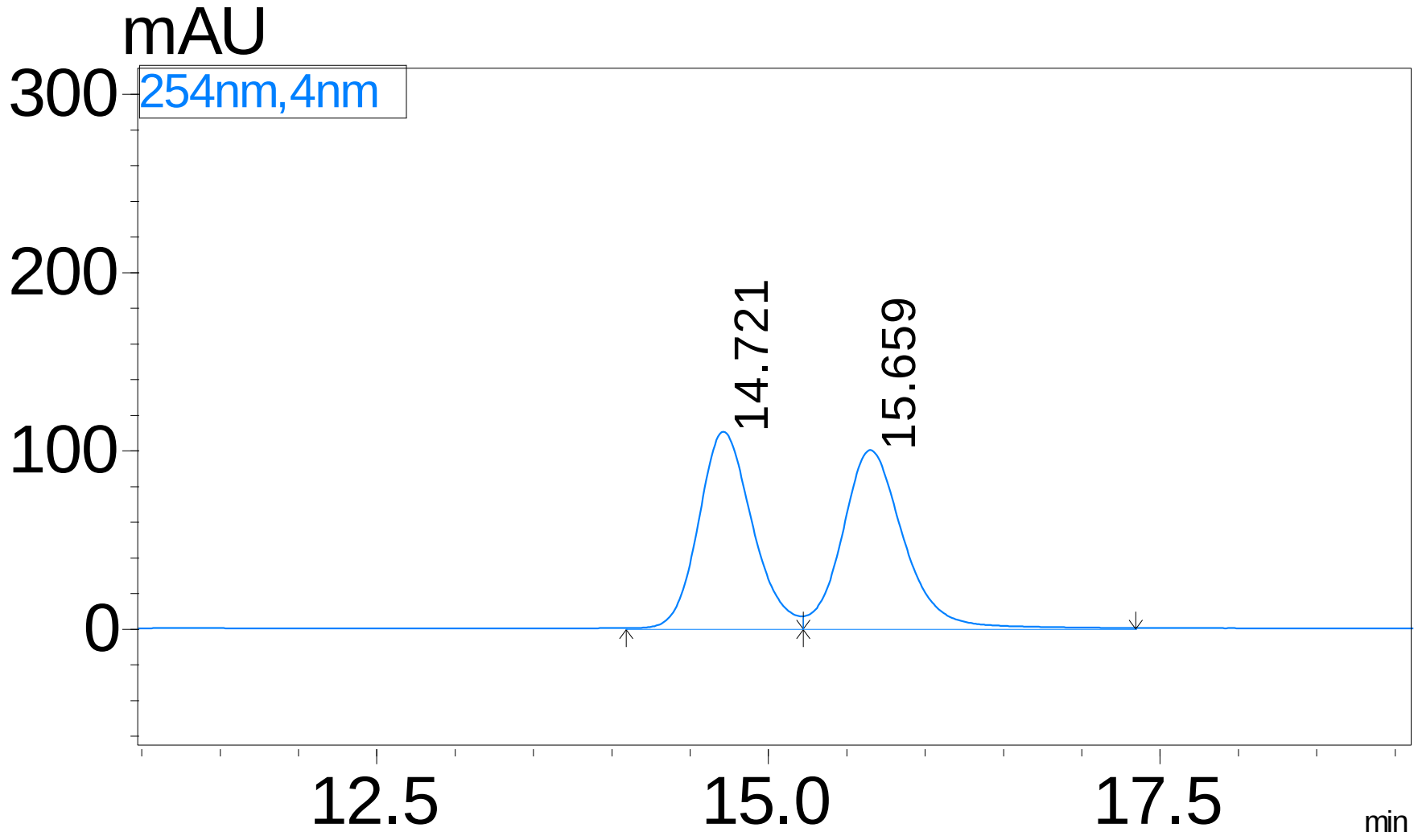

Peak	Time	Area	Height	Area%
1	14.721	2615691	110224	49.319
2	15.659	2687929	99862	50.681

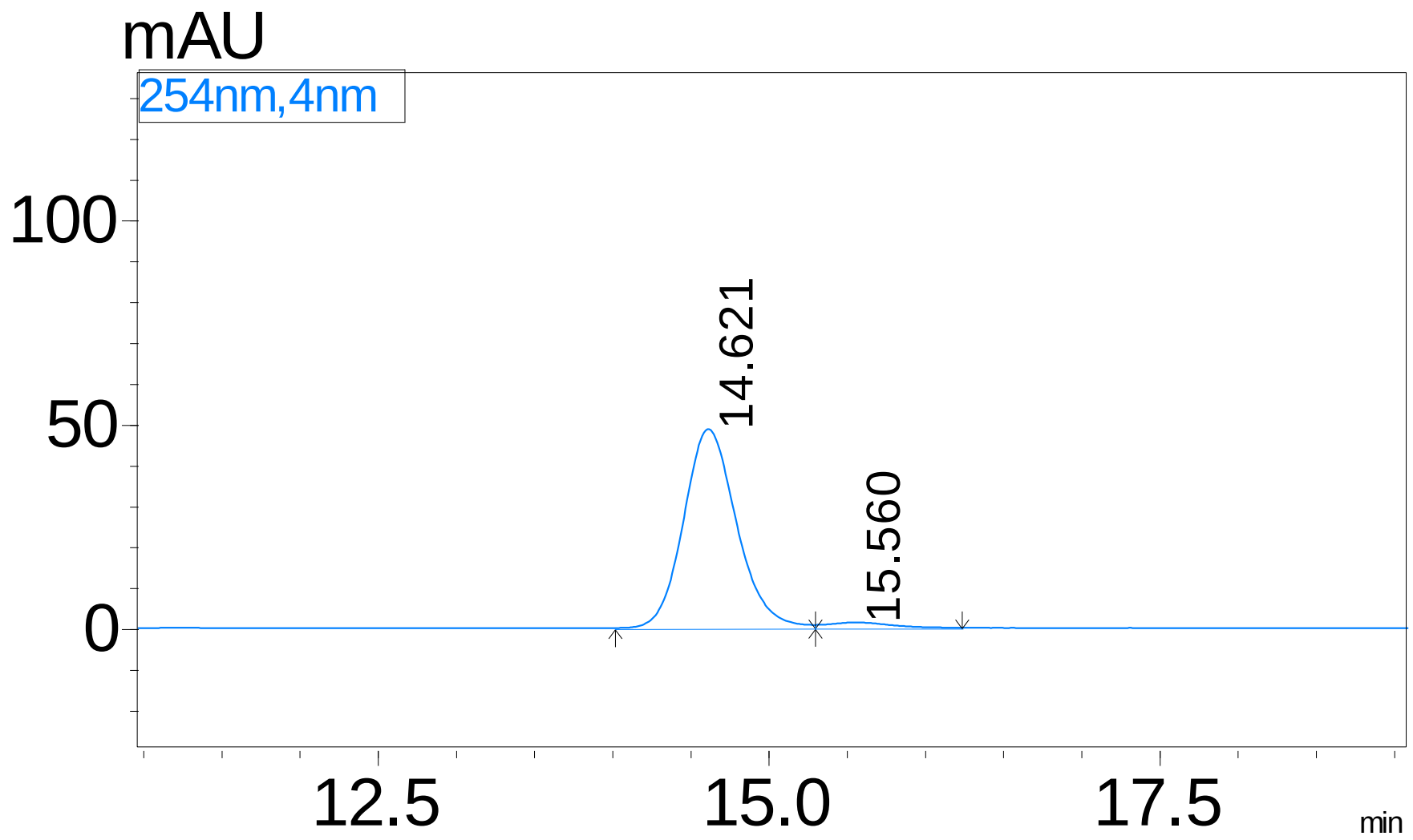

Peak	Time	Area	Height	Area%
1	14.621	1147645	48685	97.005
2	15.560	35438	1309	2.995

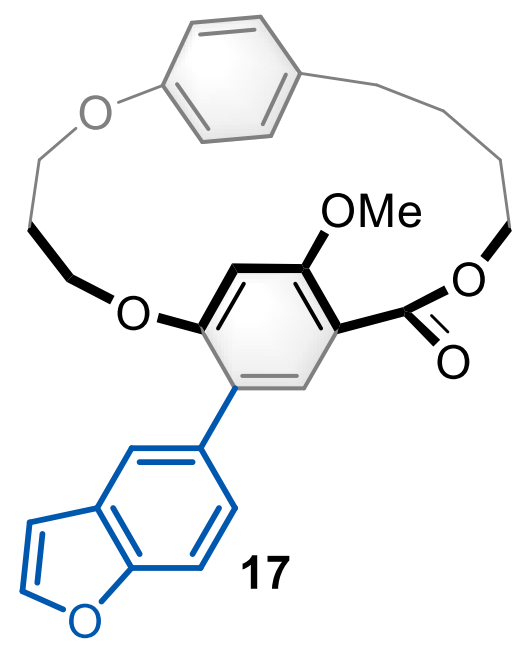

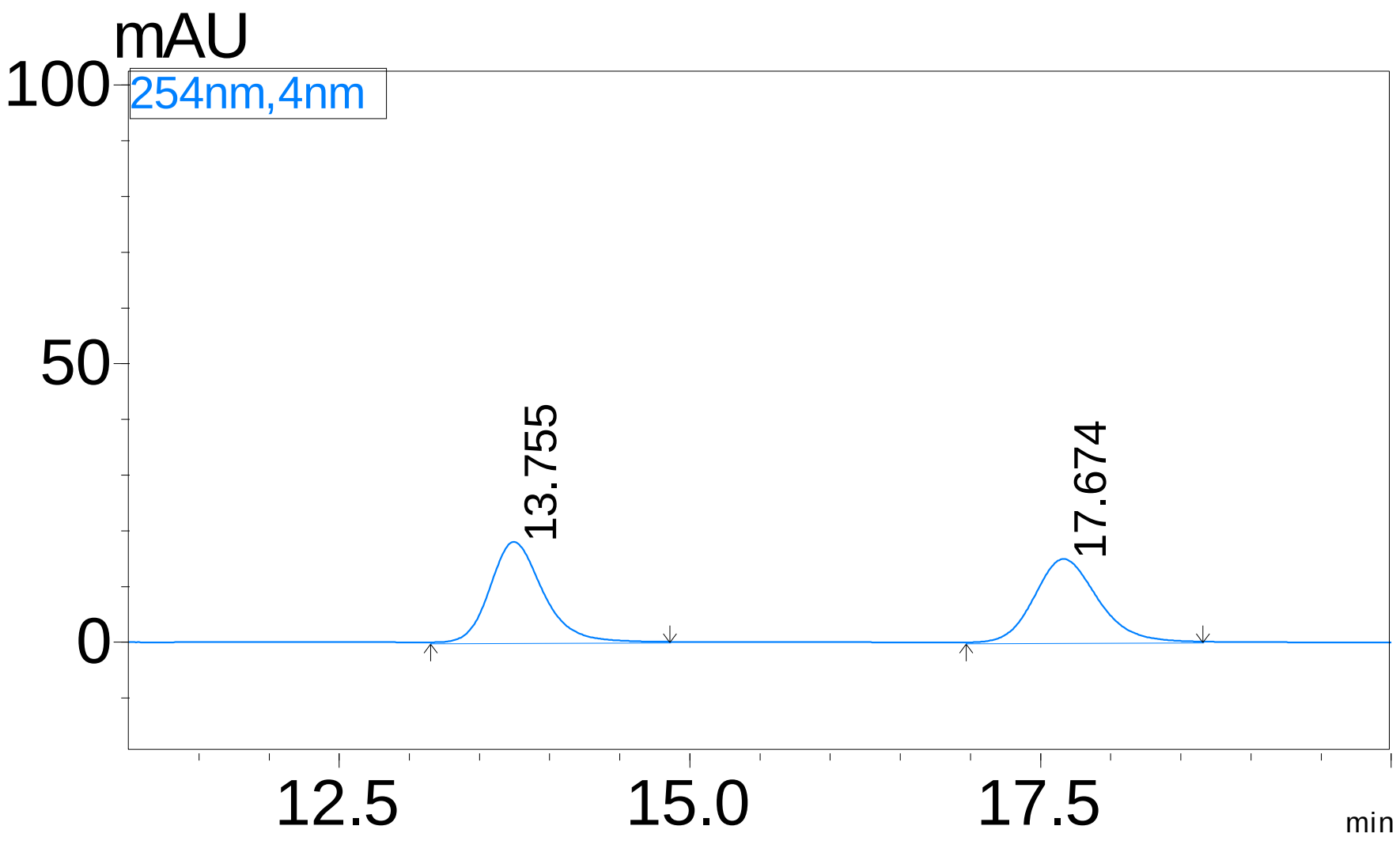

Peak	Time	Area	Height	Area%
1	13.755	468548	17963	50.120
2	17.674	466301	14917	49.880

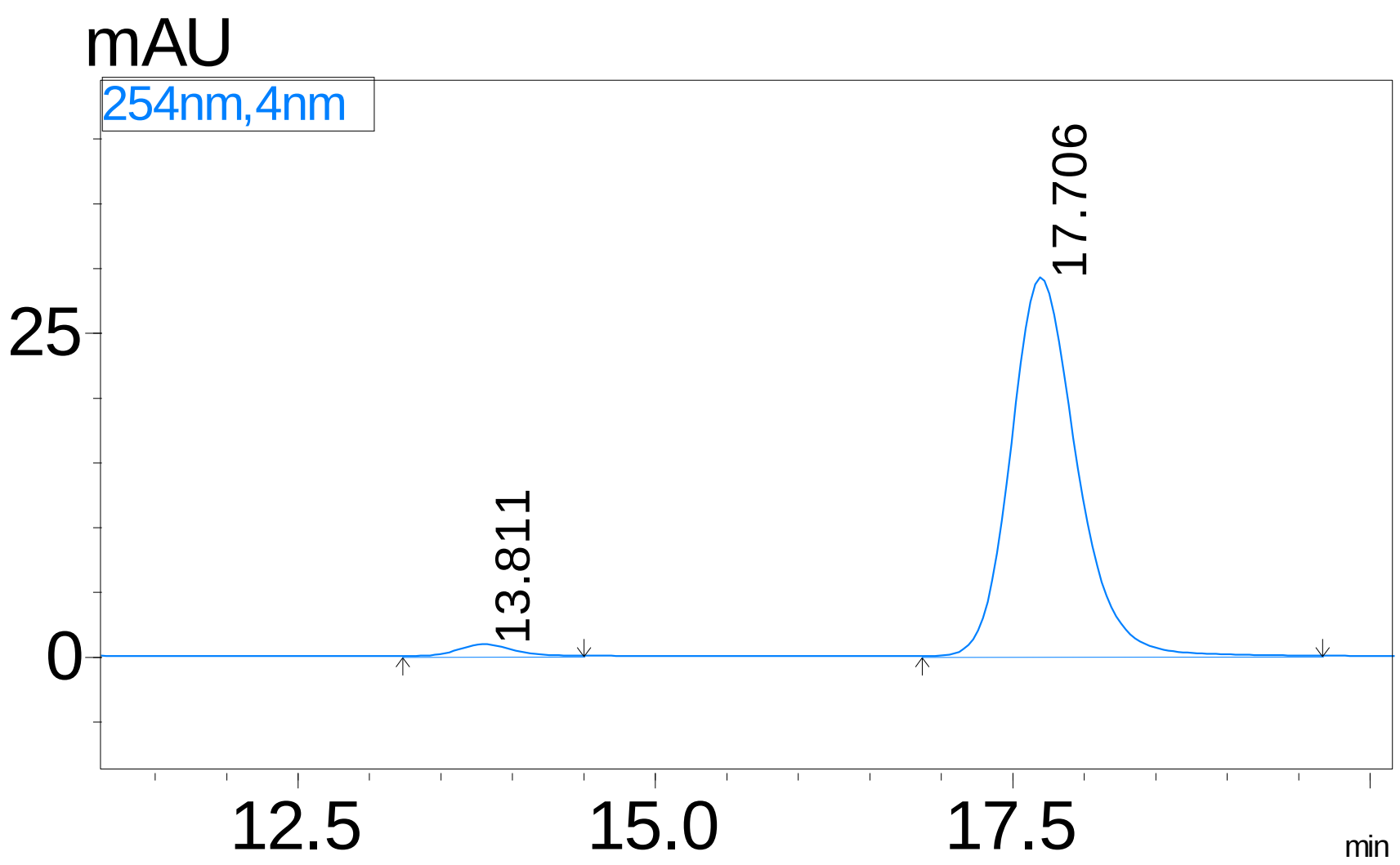

Peak	Time	Area	Height	Area%
1	13.811	23100	898	2.398
2	17.706	940270	29221	97.602

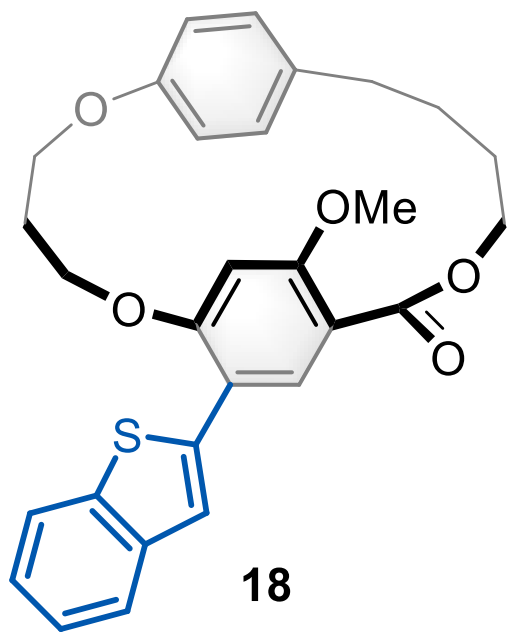

18

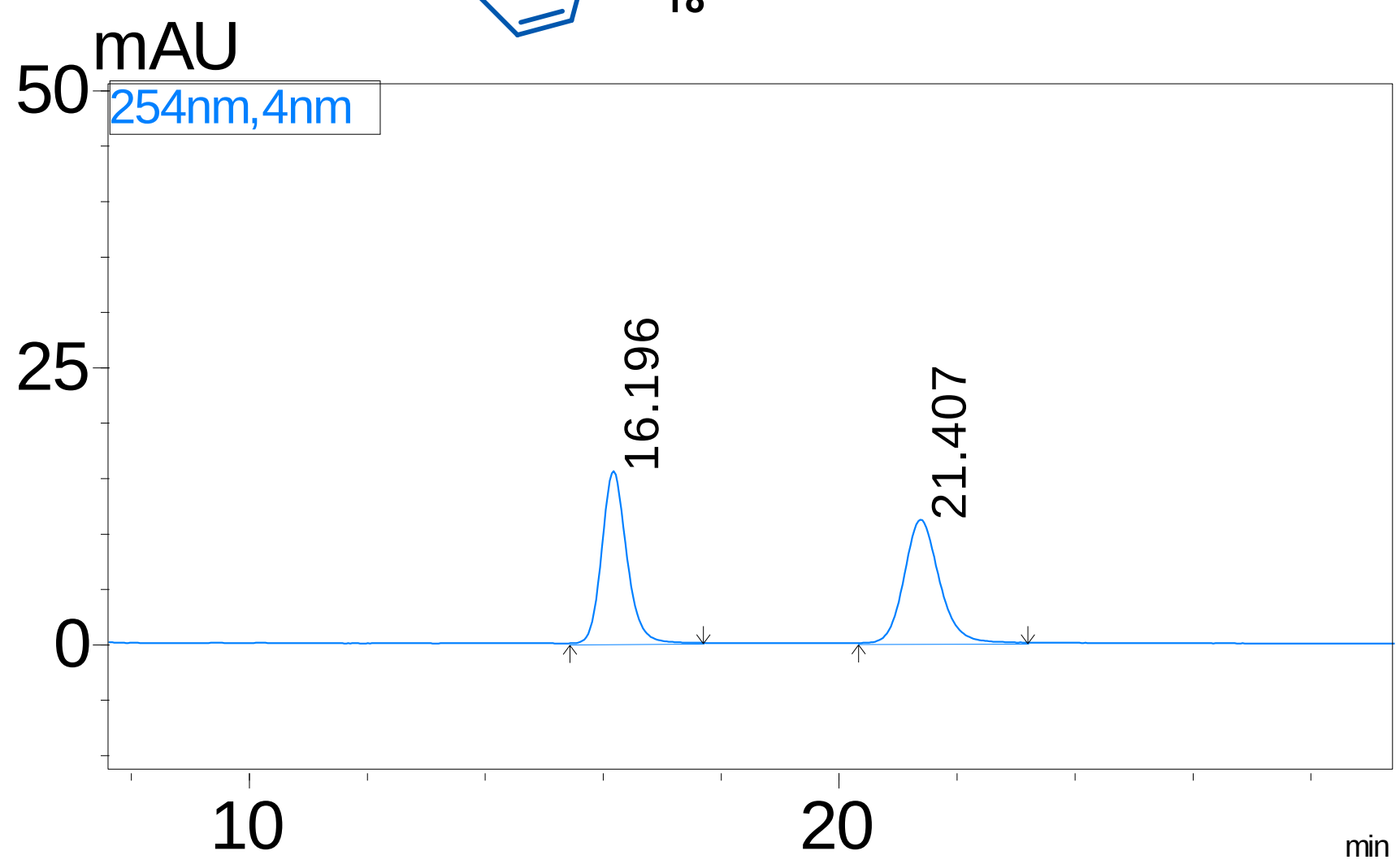

Peak	Time	Area	Height	Area%
1	16.196	458757	15516	50.066
2	21.407	457544	11115	49.934

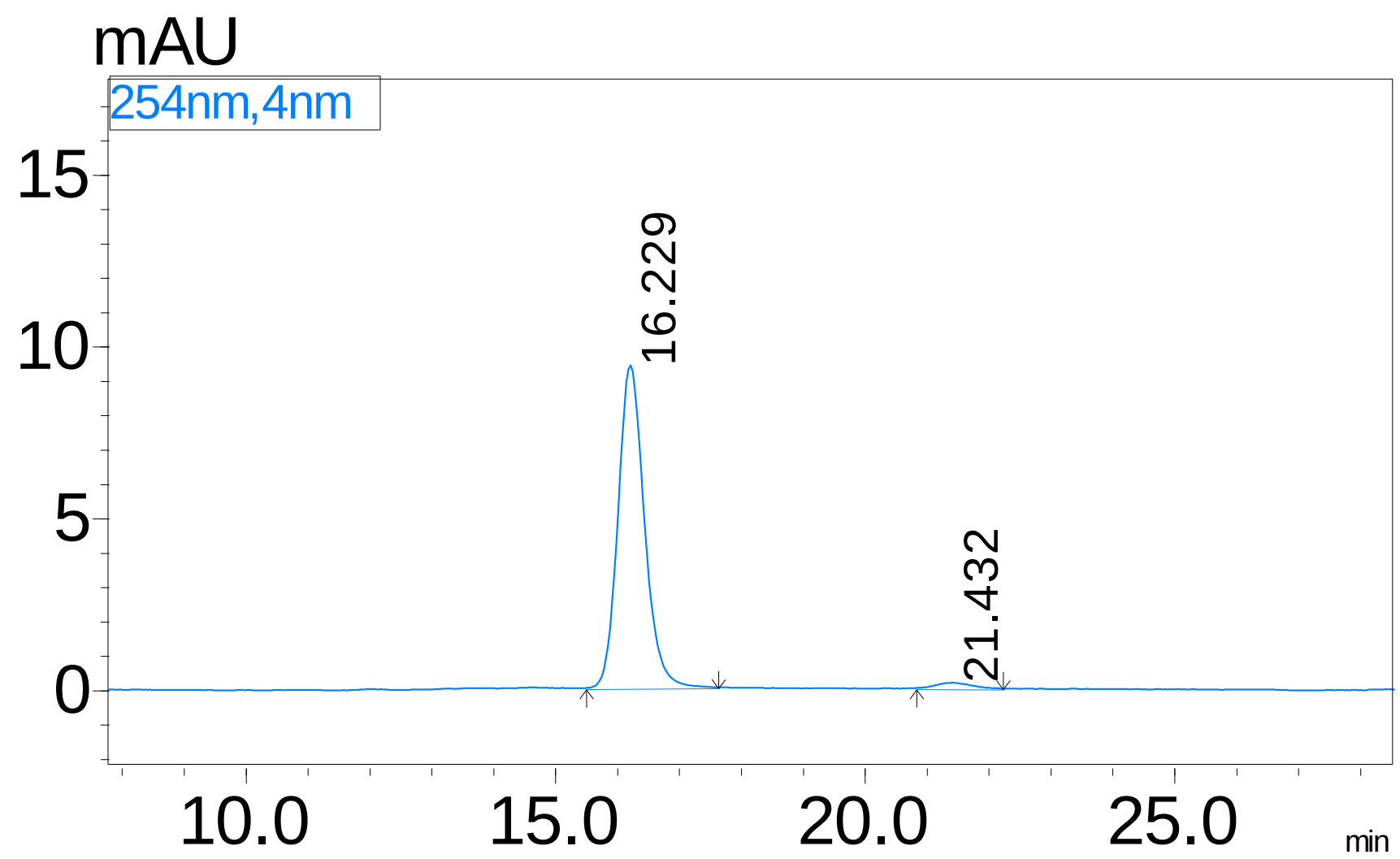

Peak	Time	Area	Height	Area%
1	16.229	277982	9386	97.798
2	21.432	6260	165	2.202

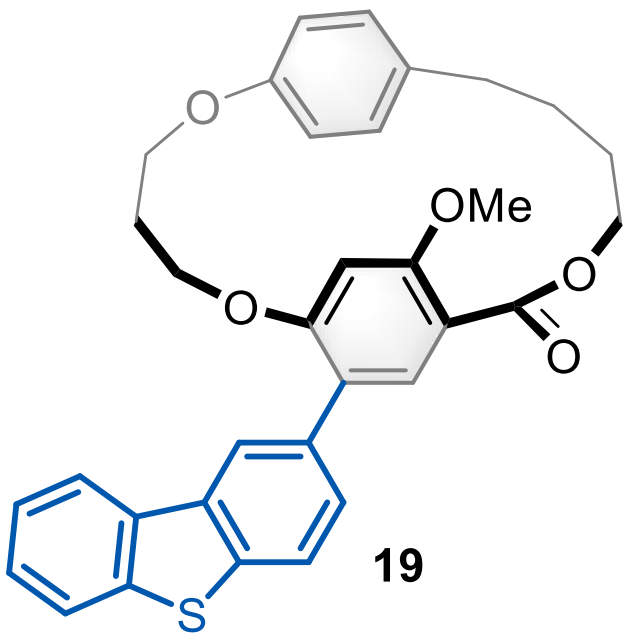

19

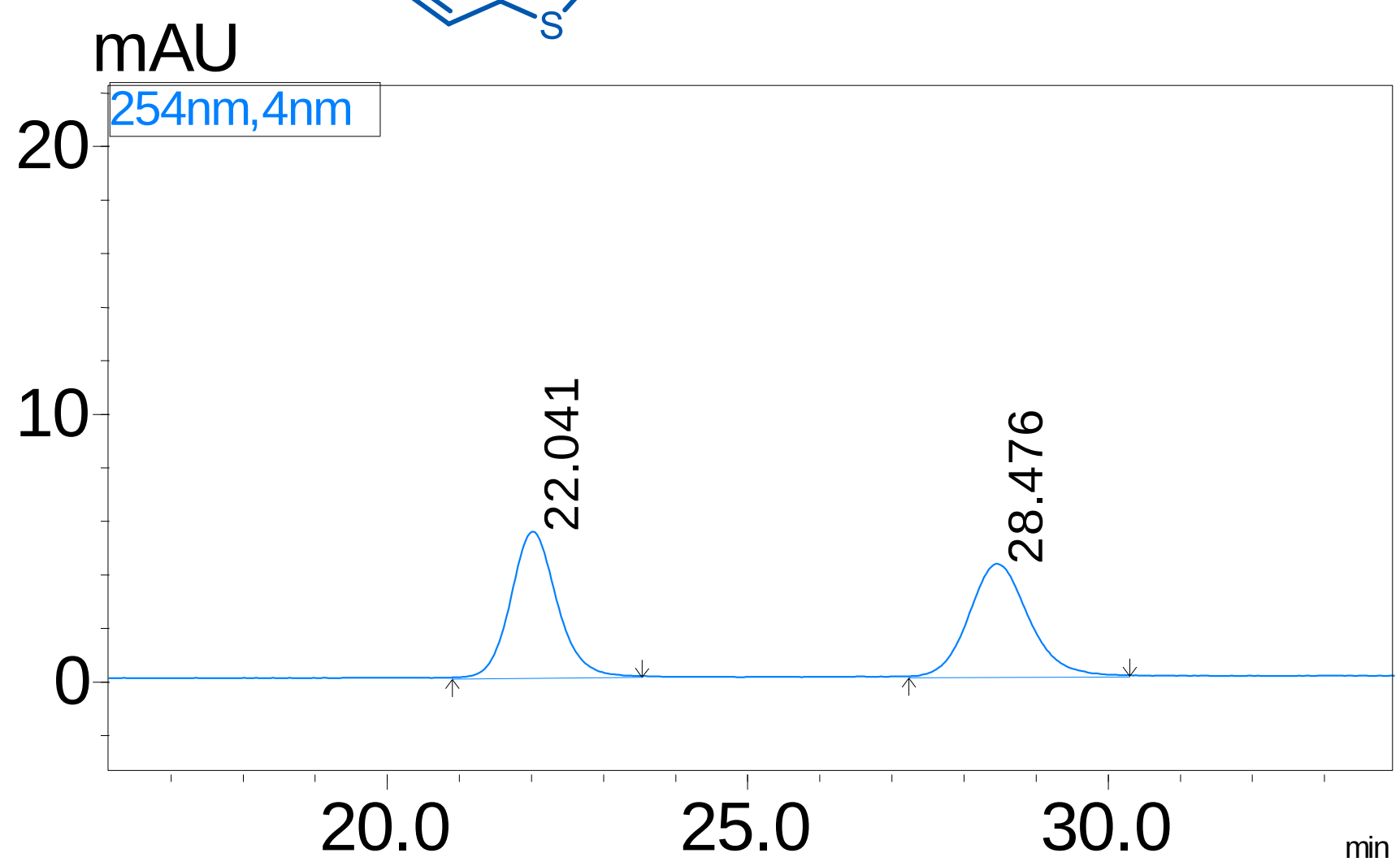

Peak	Time	Area	Height	Area%
1	22.041	241090	5430	50.146
2	28.476	239687	4190	49.854

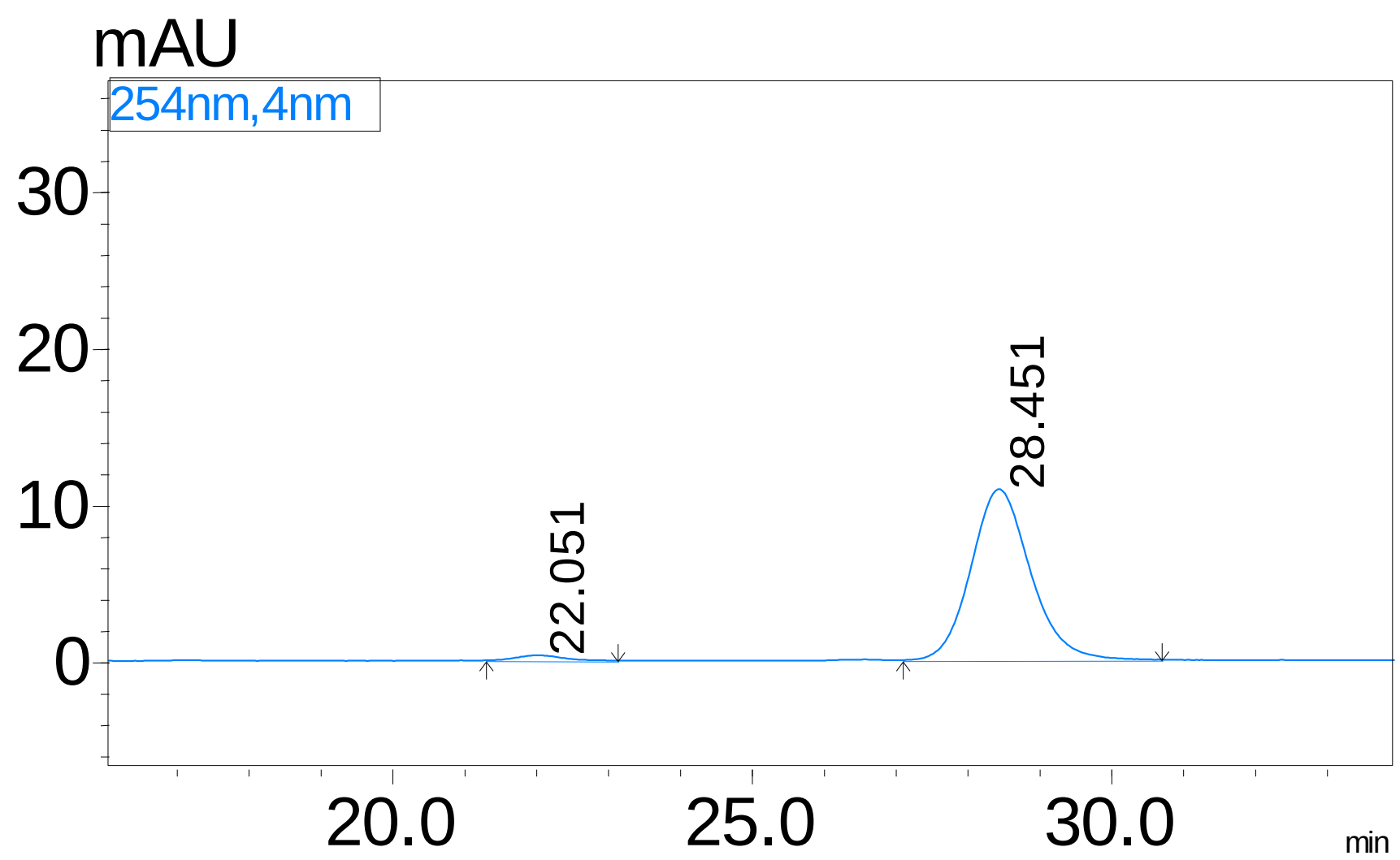

Peak	Time	Area	Height	Area%
1	22.051	14050	330	2.203
2	28.451	623800	10885	97.797

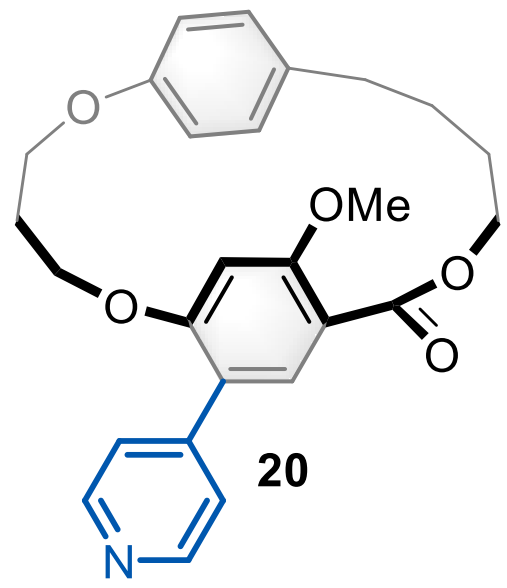

20

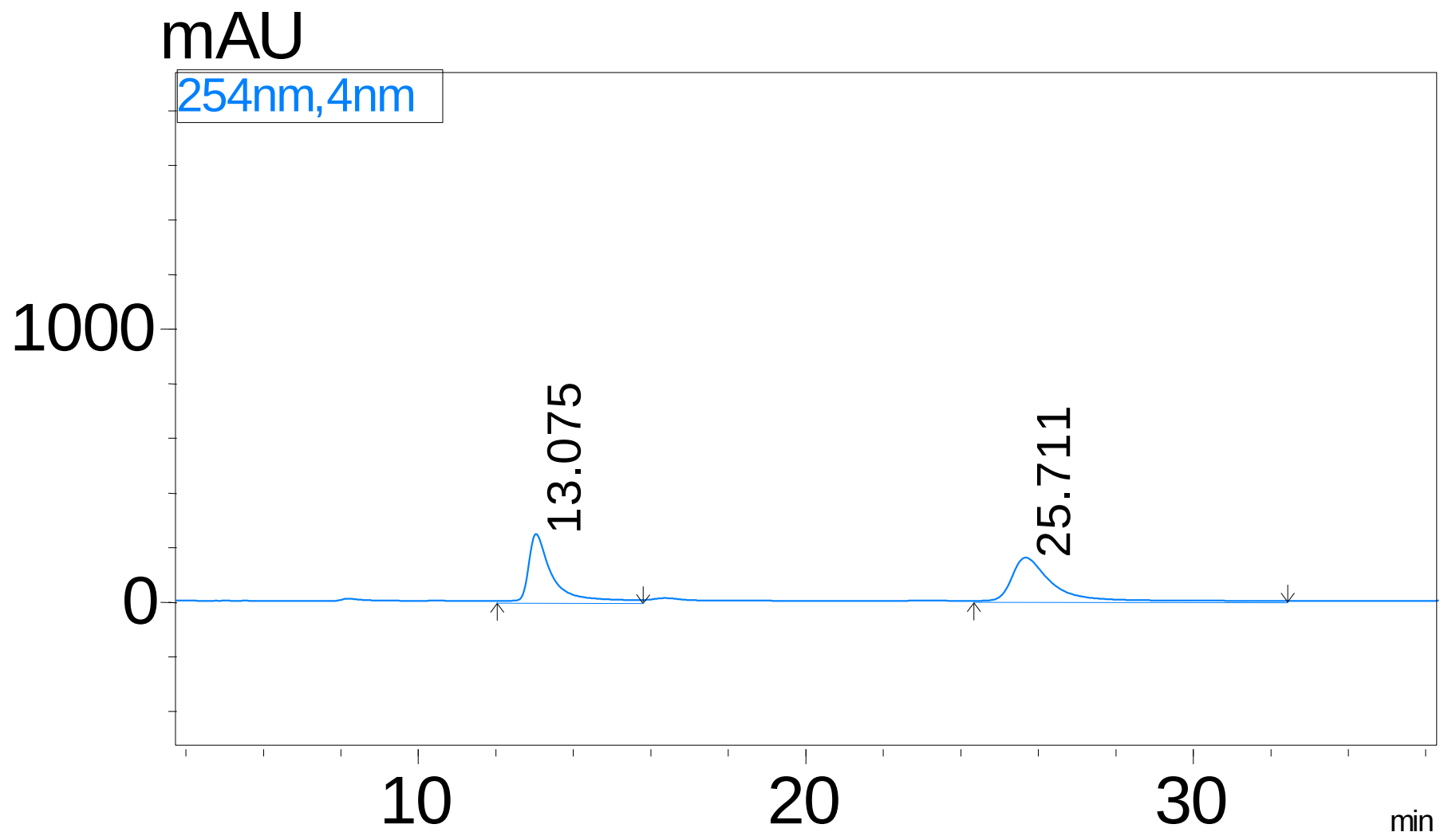

Peak	Time	Area	Height	Area%
1	13.075	10799064	249741	49.927
2	25.711	10830749	157807	50.073

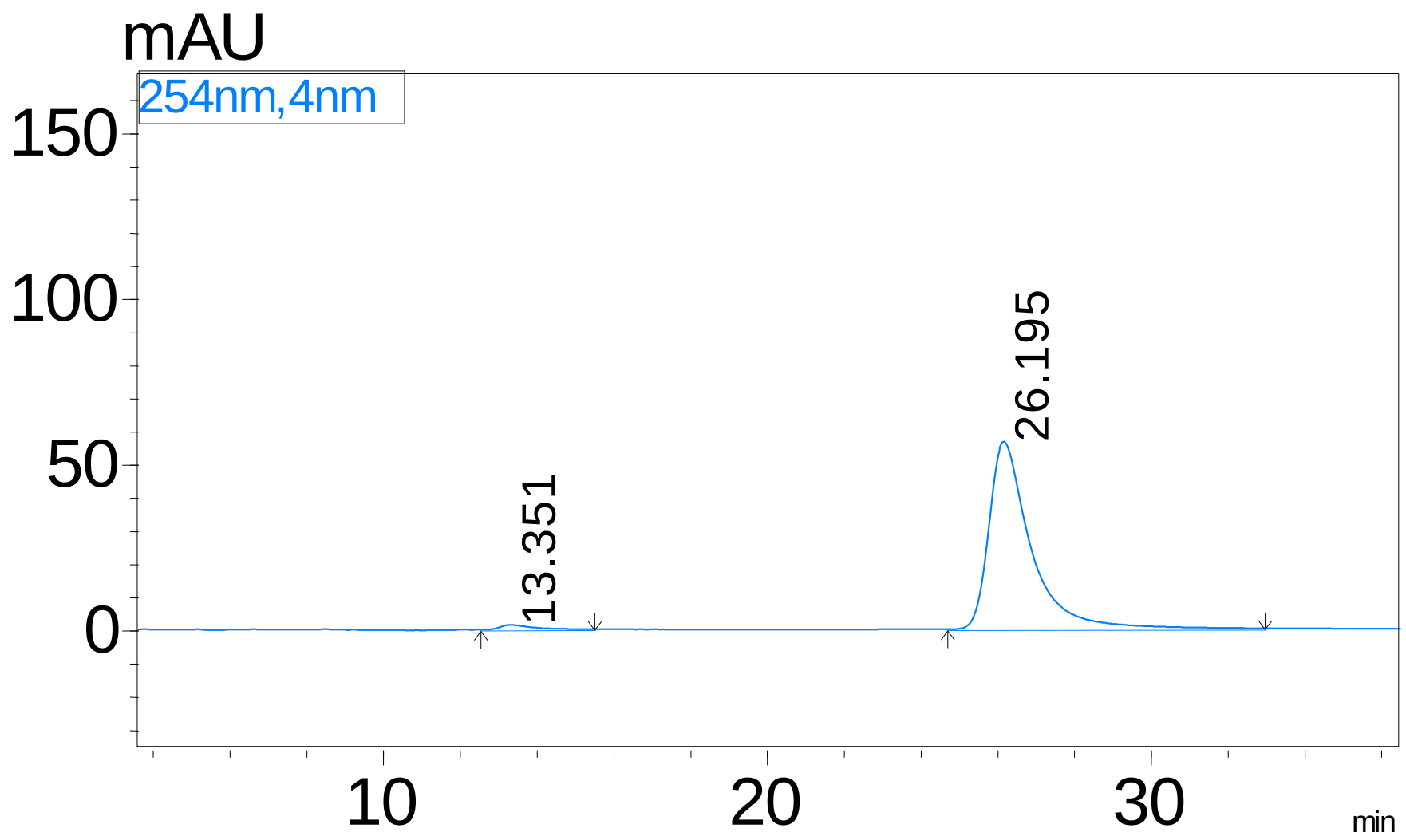

Peak	Time	Area	Height	Area%
1	13.351	84068	1449	1.923
2	26.195	4287102	56620	98.077

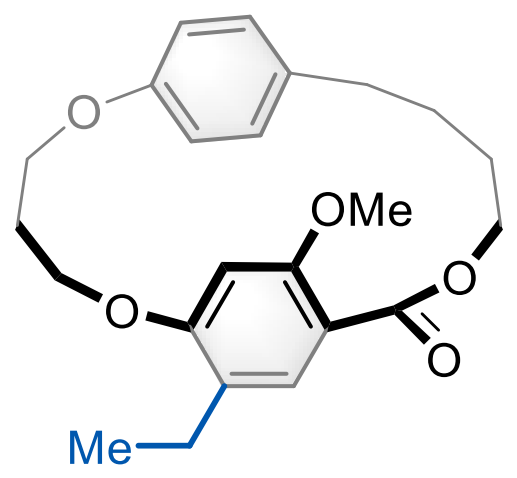

21

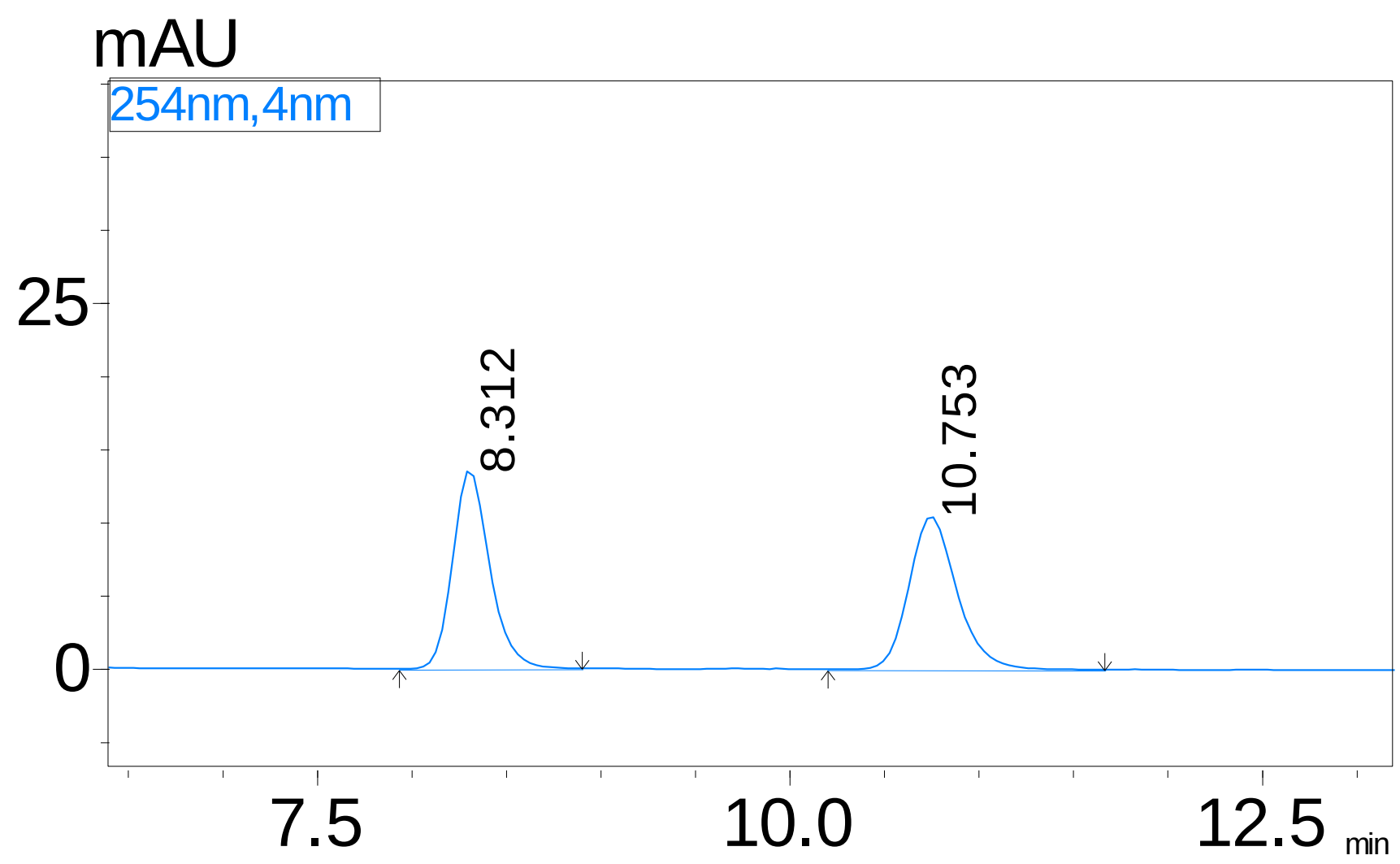

Peak	Time	Area	Height	Area%
1	8.312	185092	13470	50.139
2	10.753	184070	10385	49.861

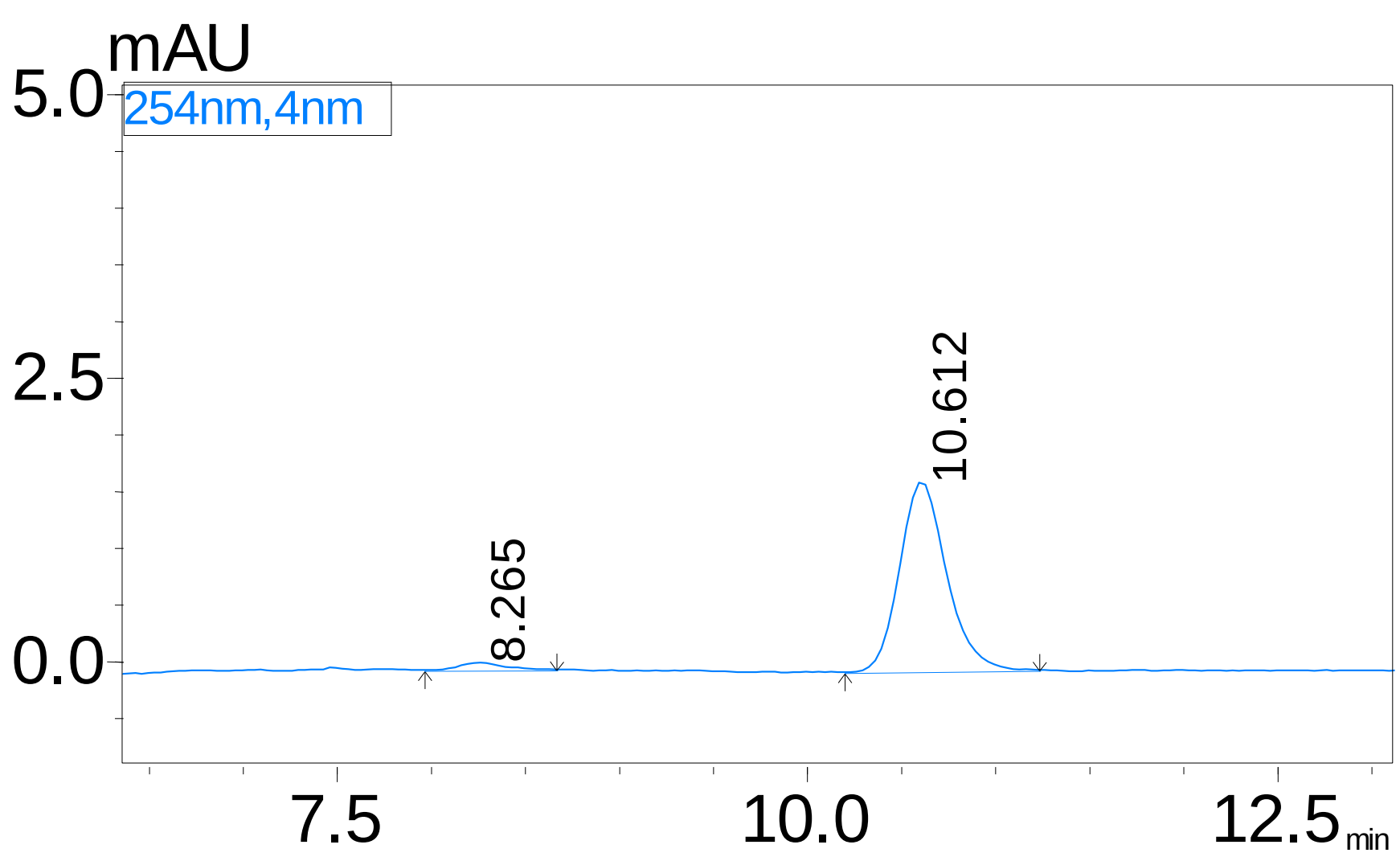

Peak	Time	Area	Height	Area%
1	8.265	938	60	3.136
2	10.612	28973	1666	96.864

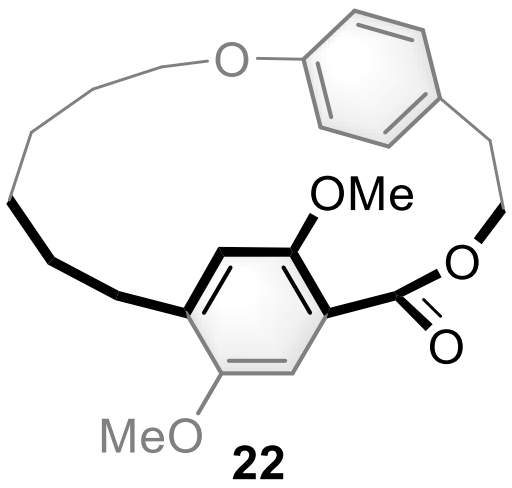

22

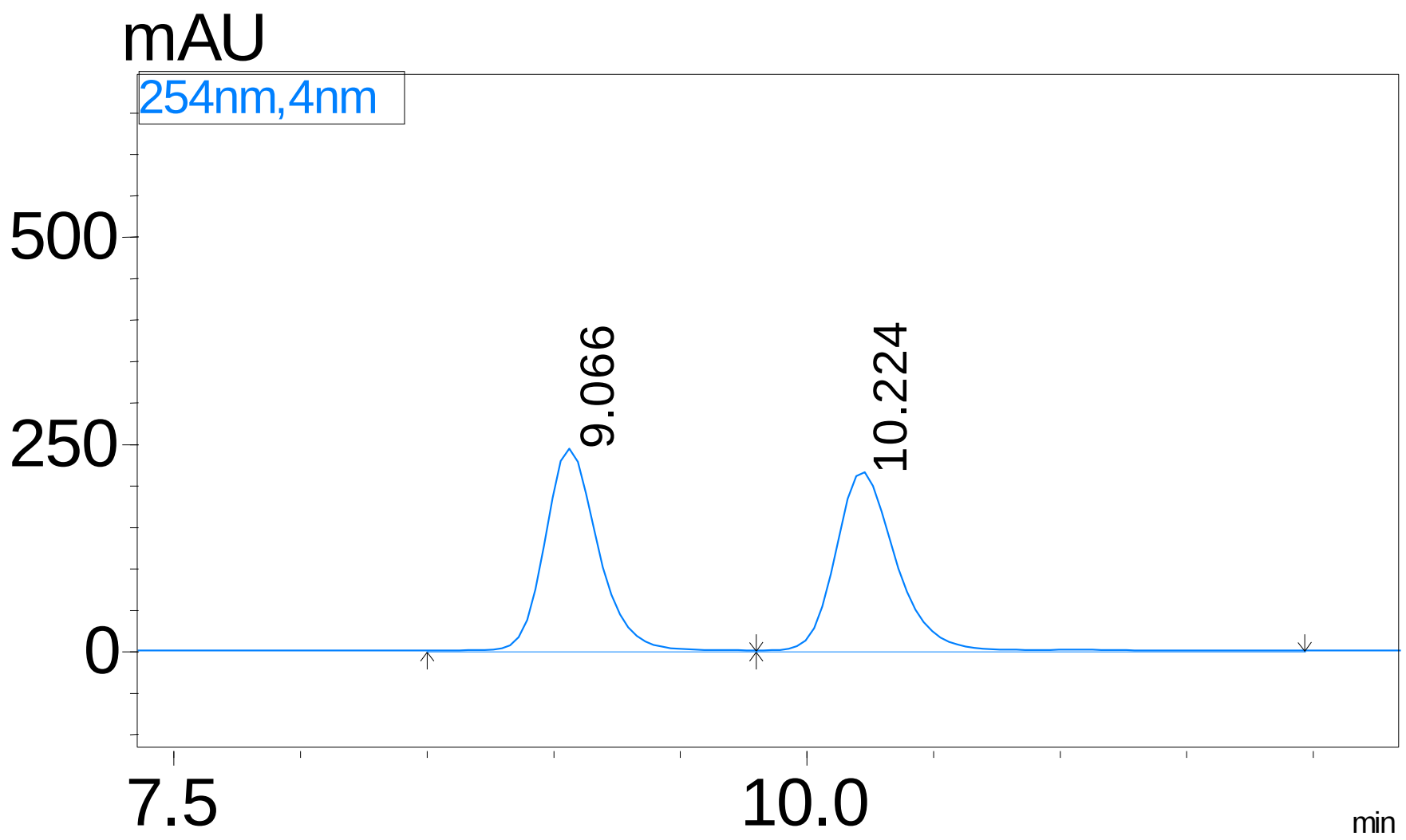

Peak	Time	Area	Height	Area%
1	9.066	3549969	243627	49.915
2	10.224	3561994	215048	50.085

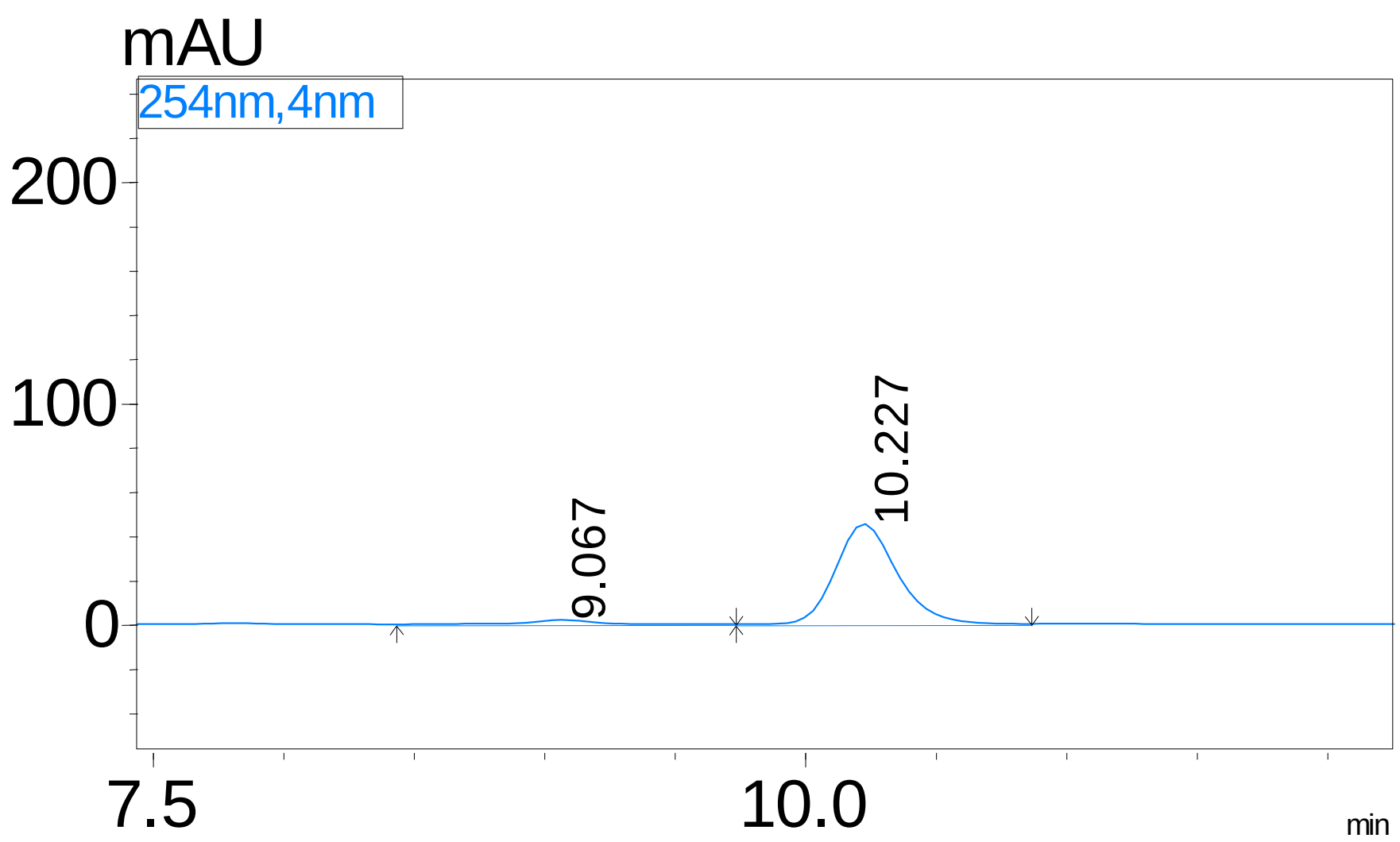

Peak	Time	Area	Height	Area%
1	9.067	39156	2074	4.987
2	10.227	745924	45287	95.013

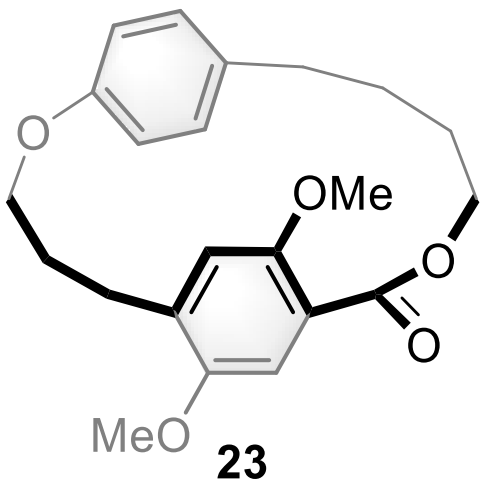

23

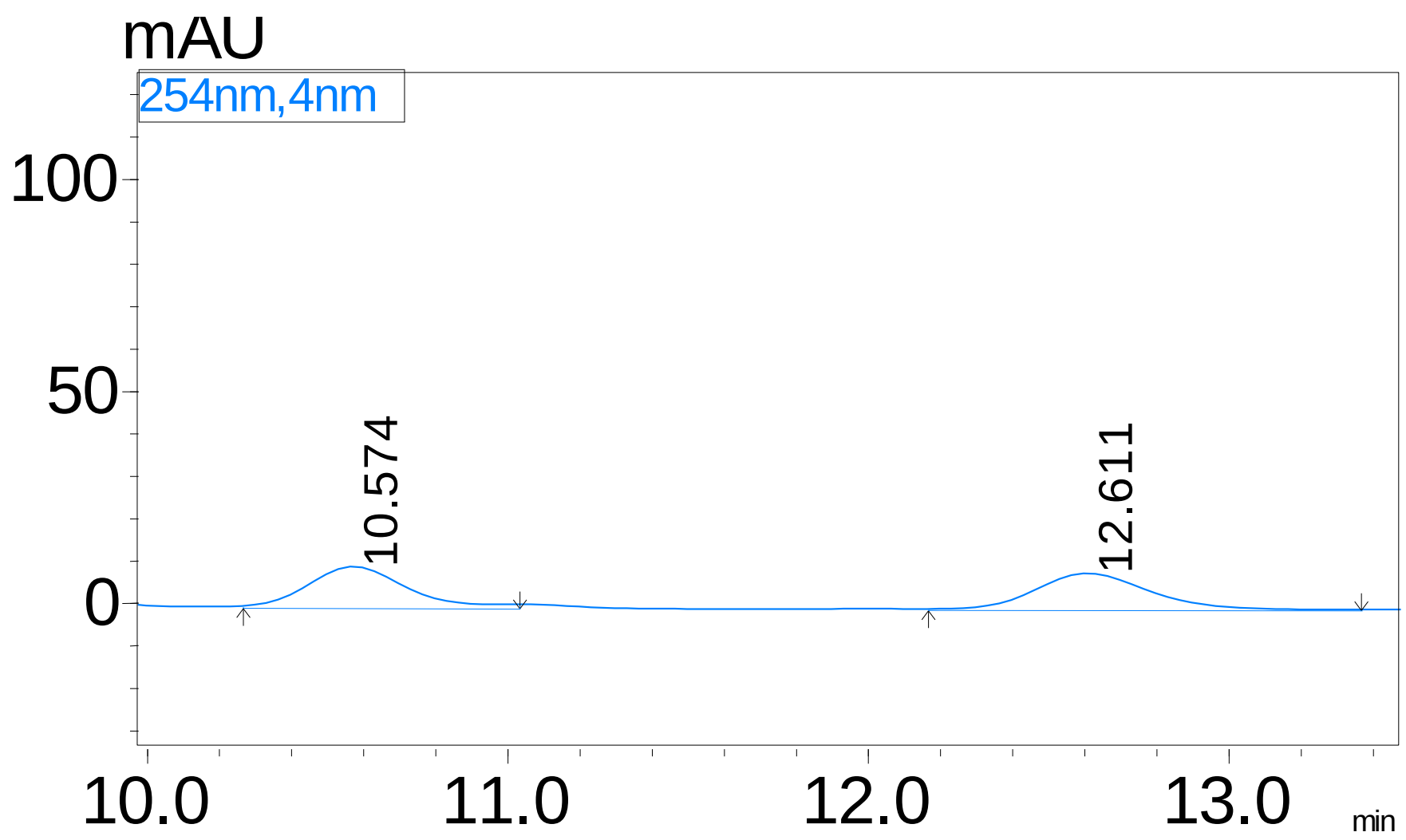

Peak	Time	Area	Height	Area%
1	10.574	180933	9601	50.205
2	12.611	179455	8443	49.795

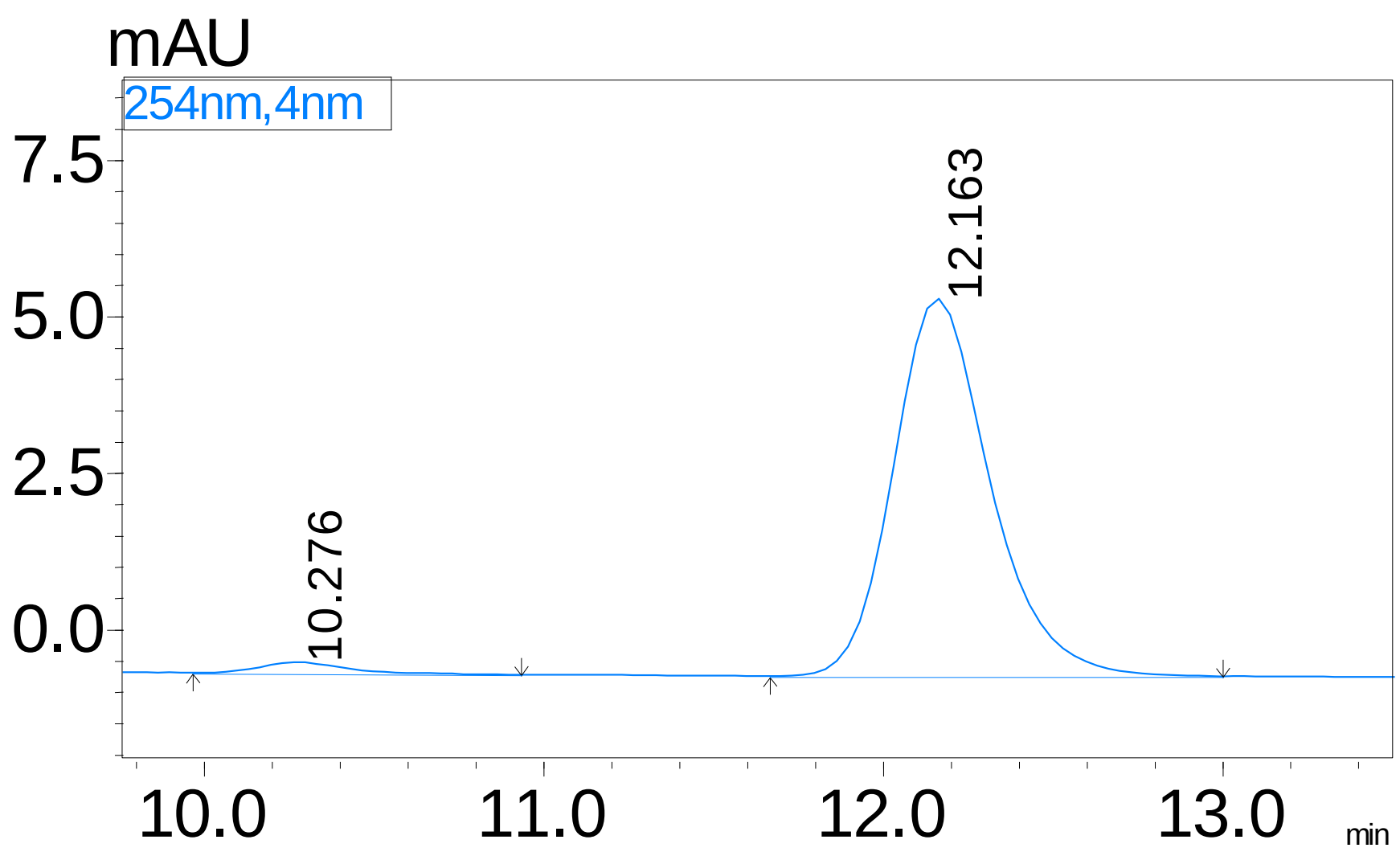

Peak	Time	Area	Height	Area%
1	10.276	3095	179	2.514
2	12.163	120034	6031	97.486

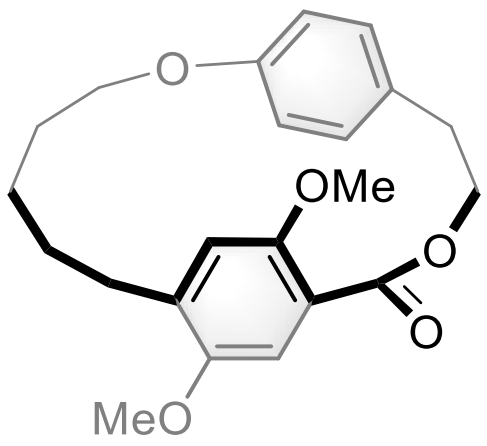

24

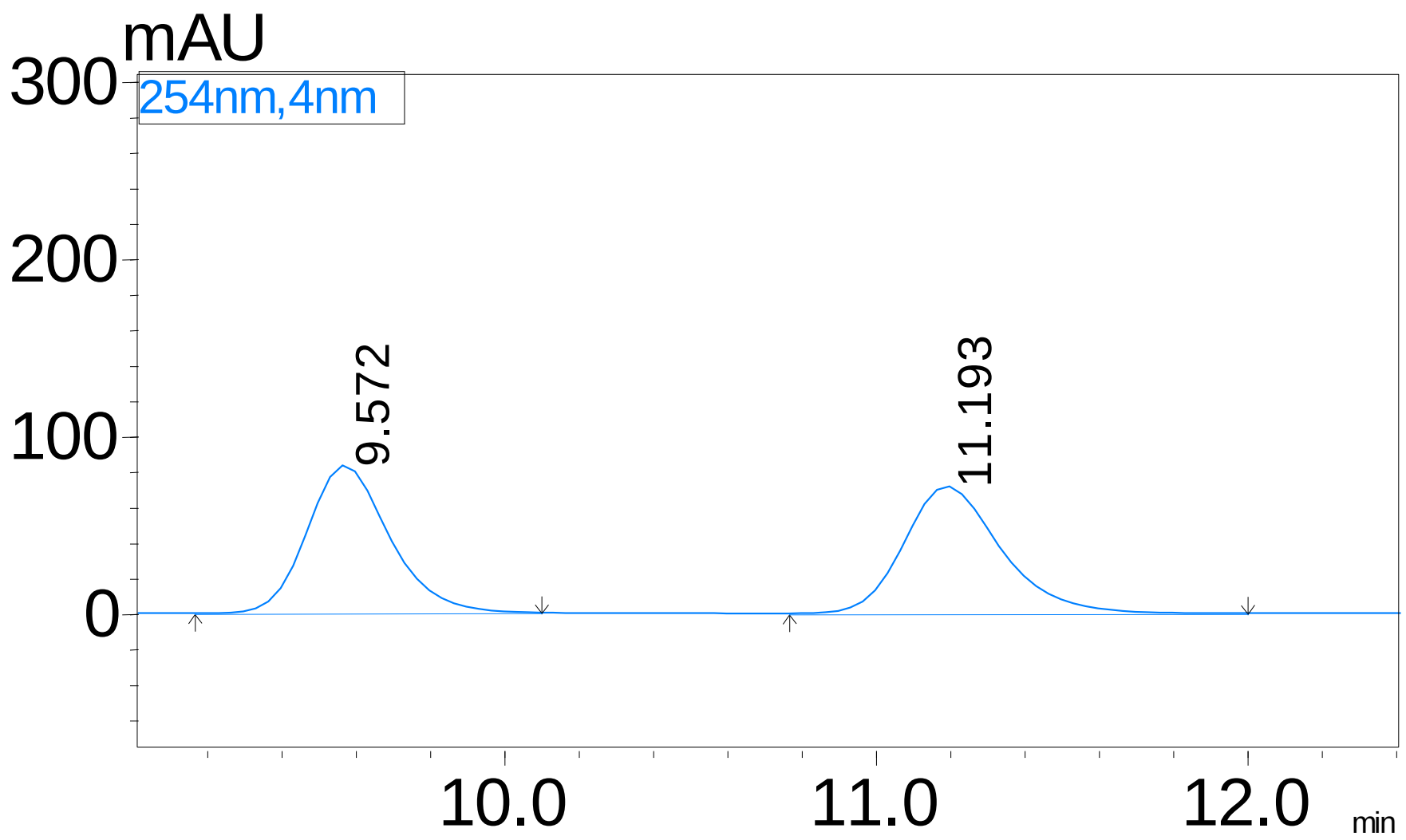

Peak	Time	Area	Height	Area%
1	9.572	1285677	83140	49.488
2	11.193	1312267	71603	50.512

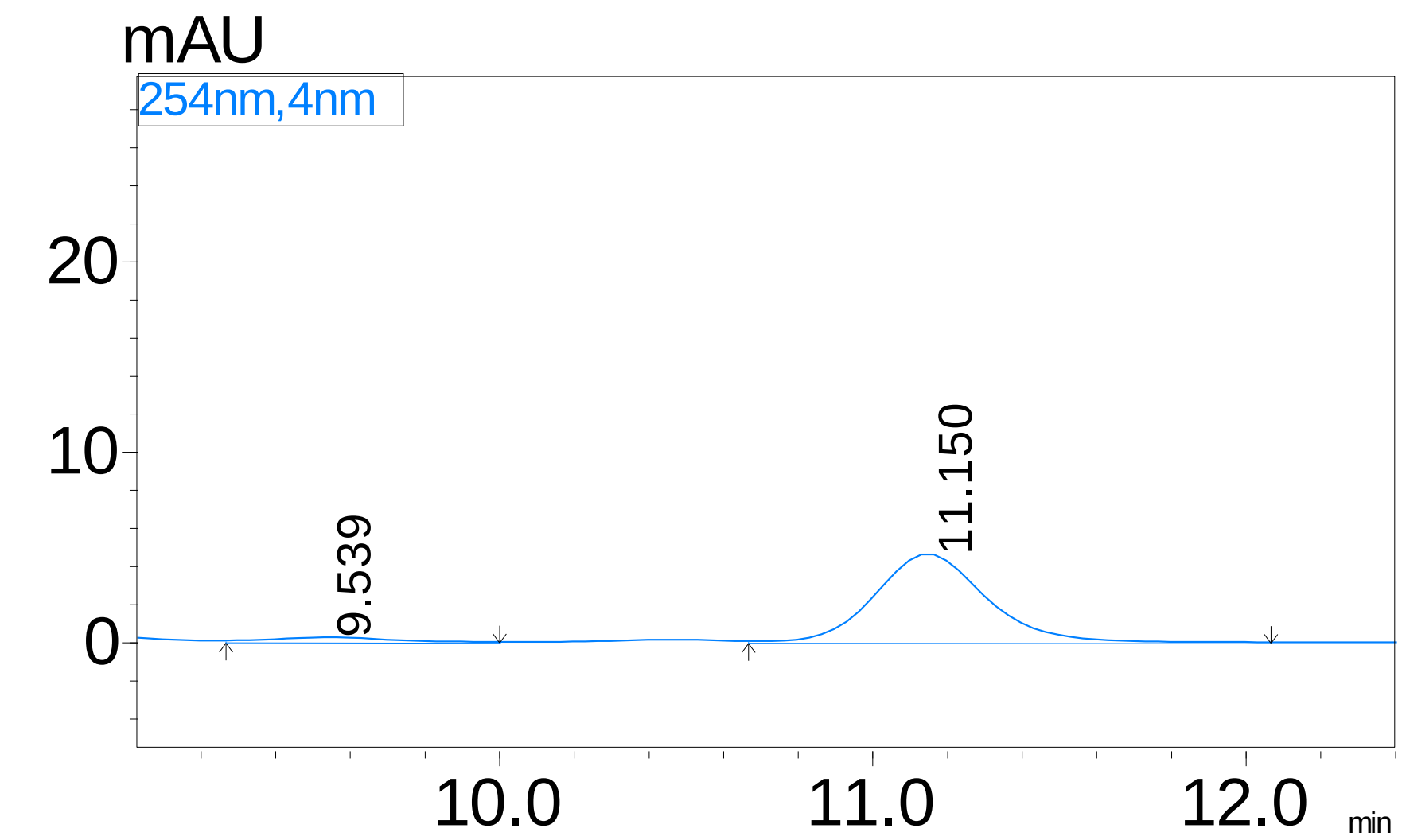

Peak	Time	Area	Height	Area%
1	9.539	4593	243	4.614
2	11.150	94948	4602	95.386

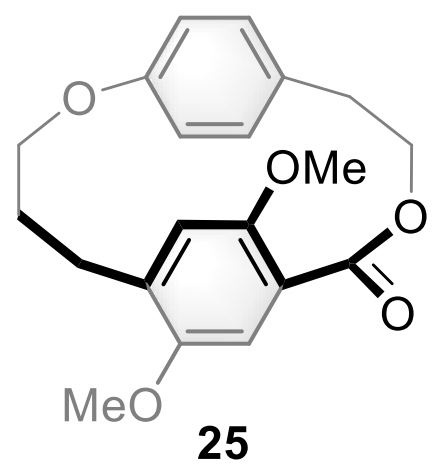

25

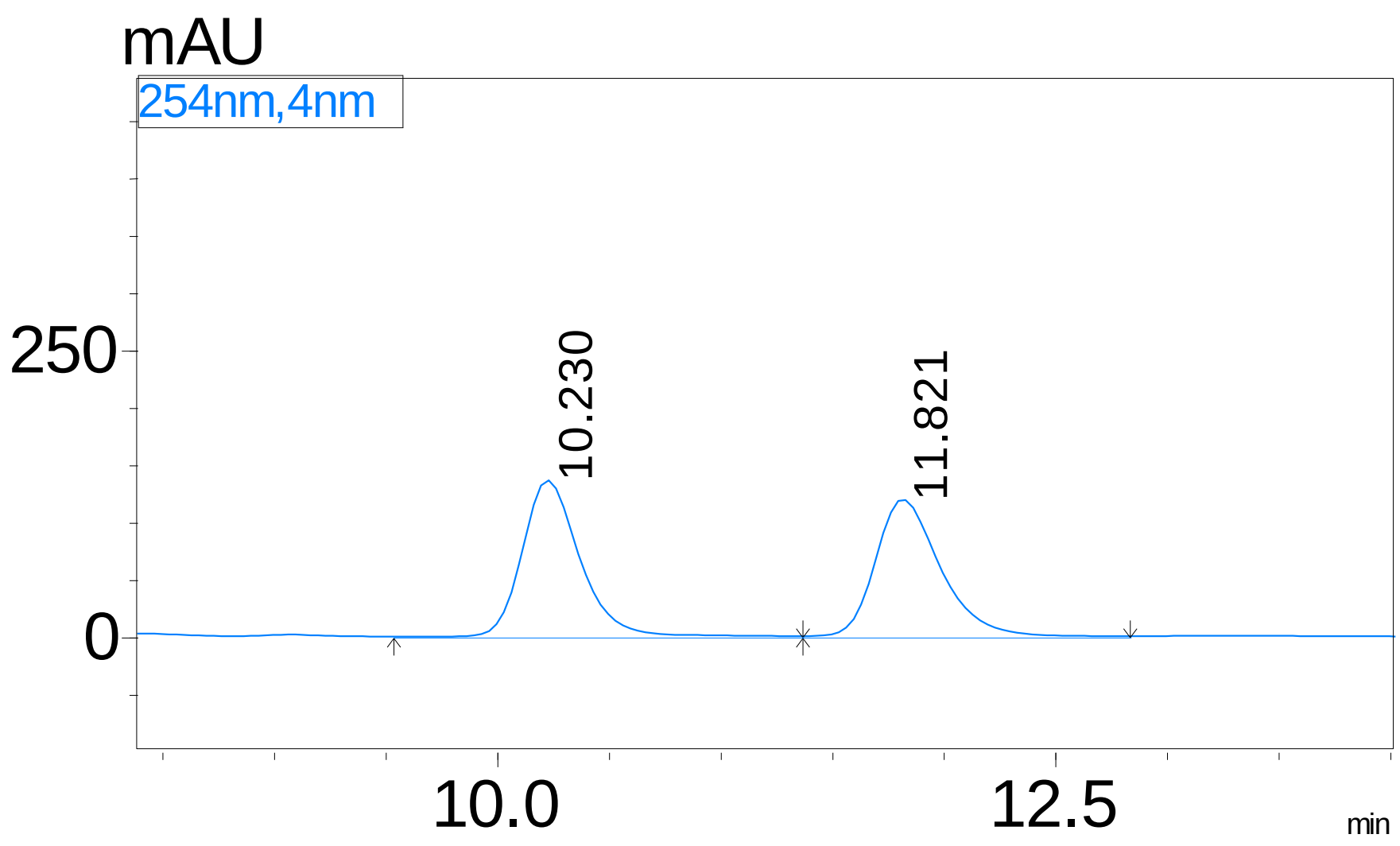

Peak	Time	Area	Height	Area%
1	10.230	2446273	136334	50.259
2	11.821	2421098	119177	49.741

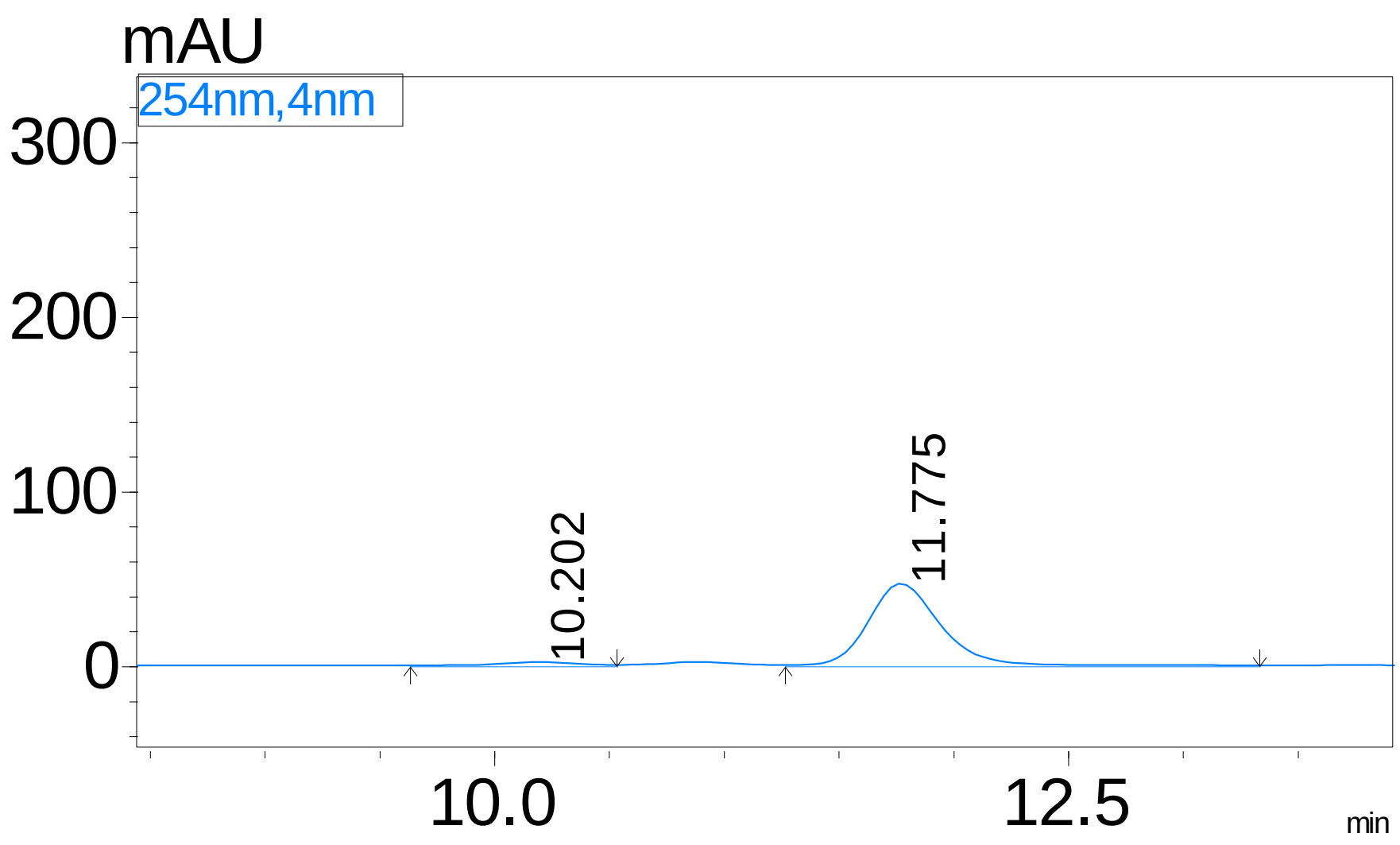

Peak	Time	Area	Height	Area%
1	10.202	38663	1920	3.727
2	11.775	998766	46793	96.273

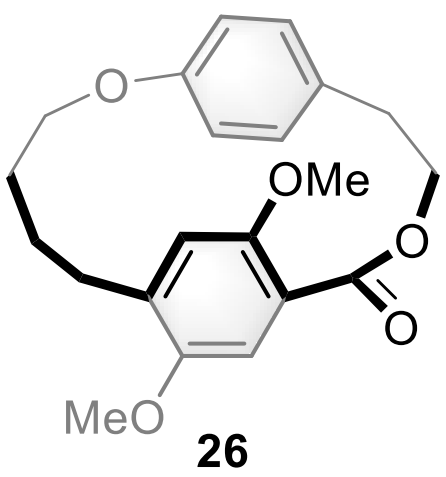

26

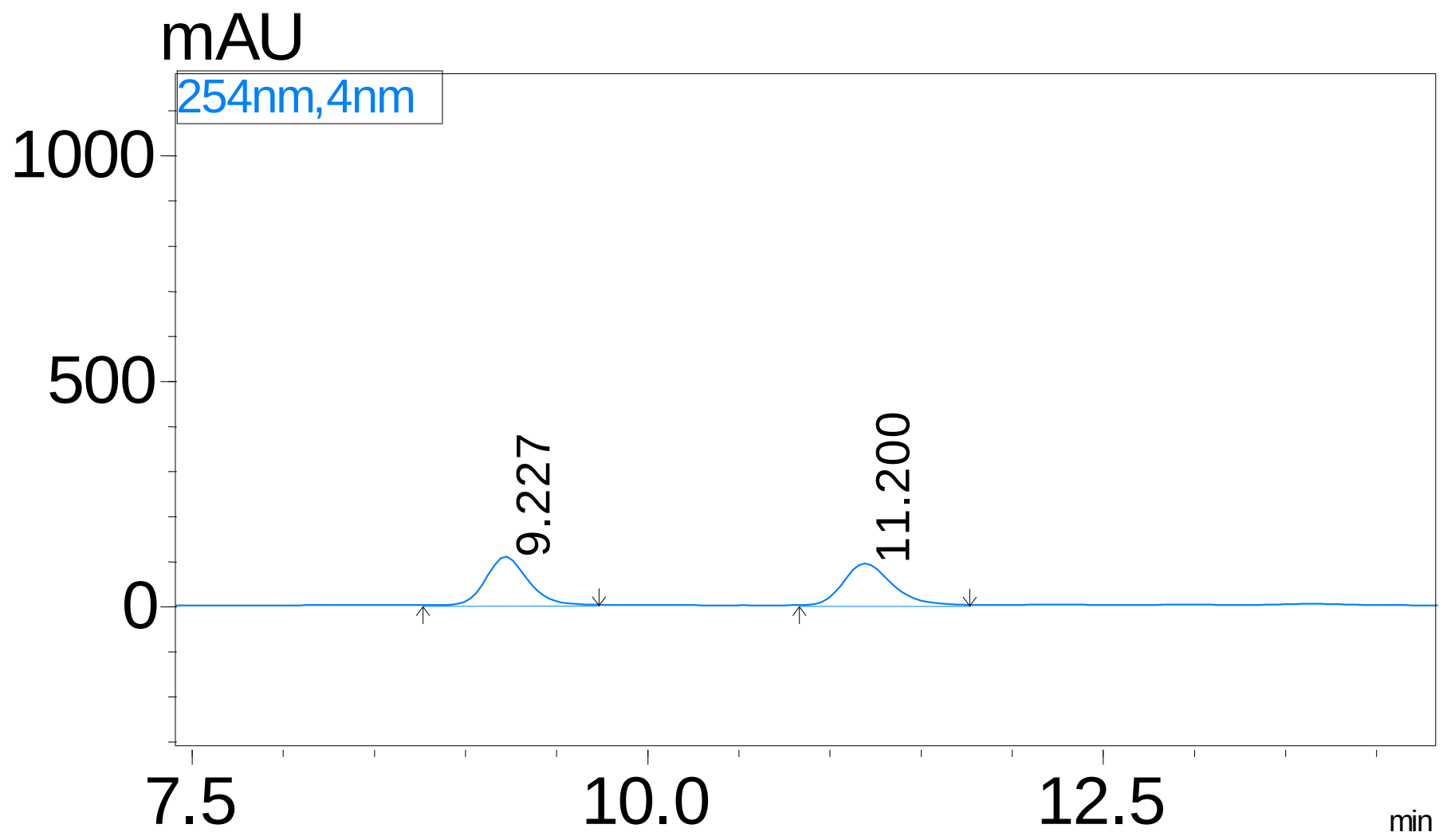

Peak	Time	Area	Height	Area%
1	9.227	1694241	107151	50.066
2	11.200	1689797	92678	49.934

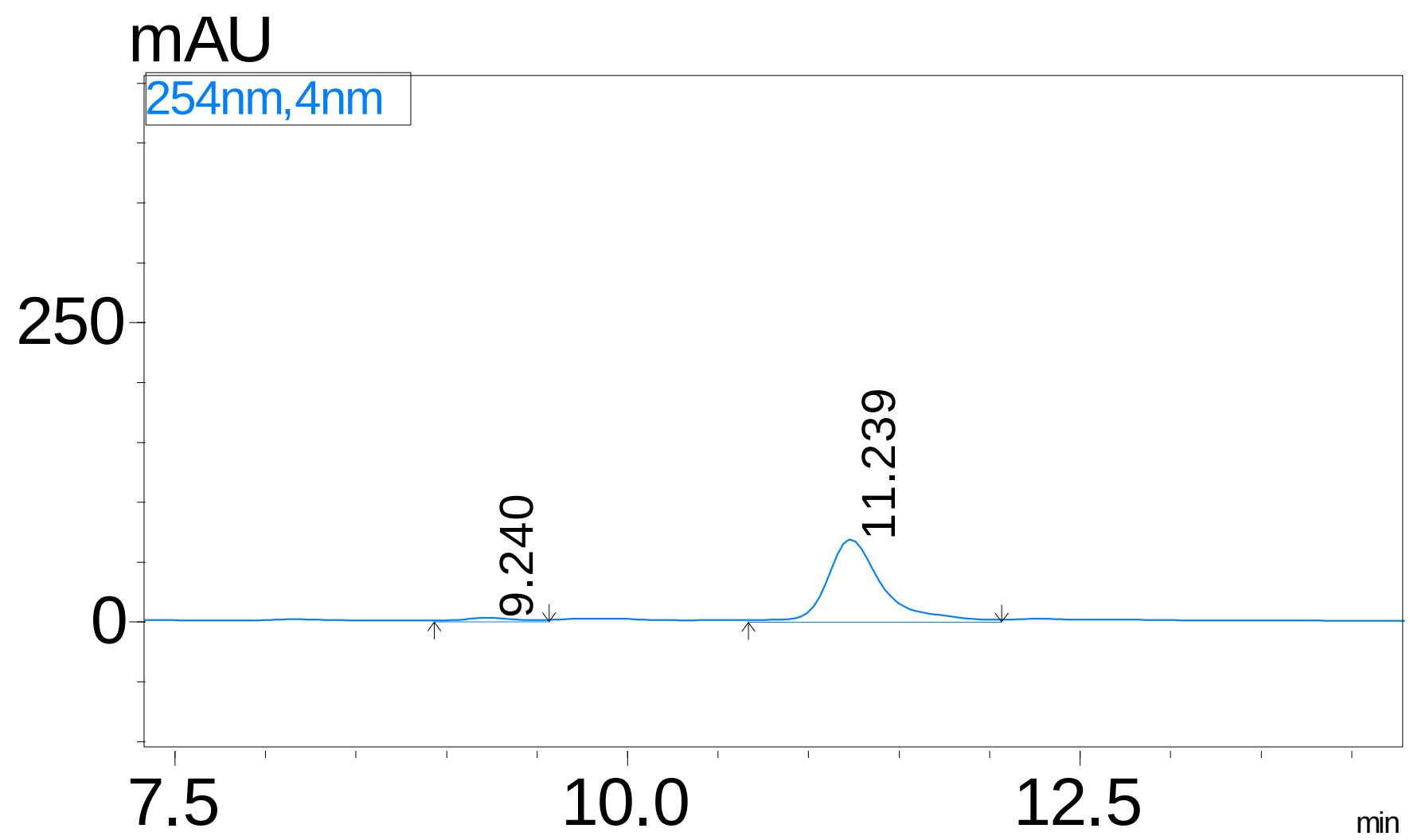

Peak	Time	Area	Height	Area%
1	9.240	42849	2436	2.980
2	11.239	1395073	68045	97.020

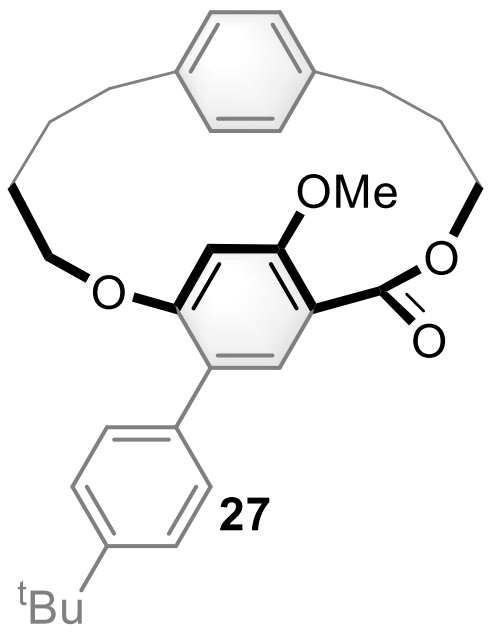

27

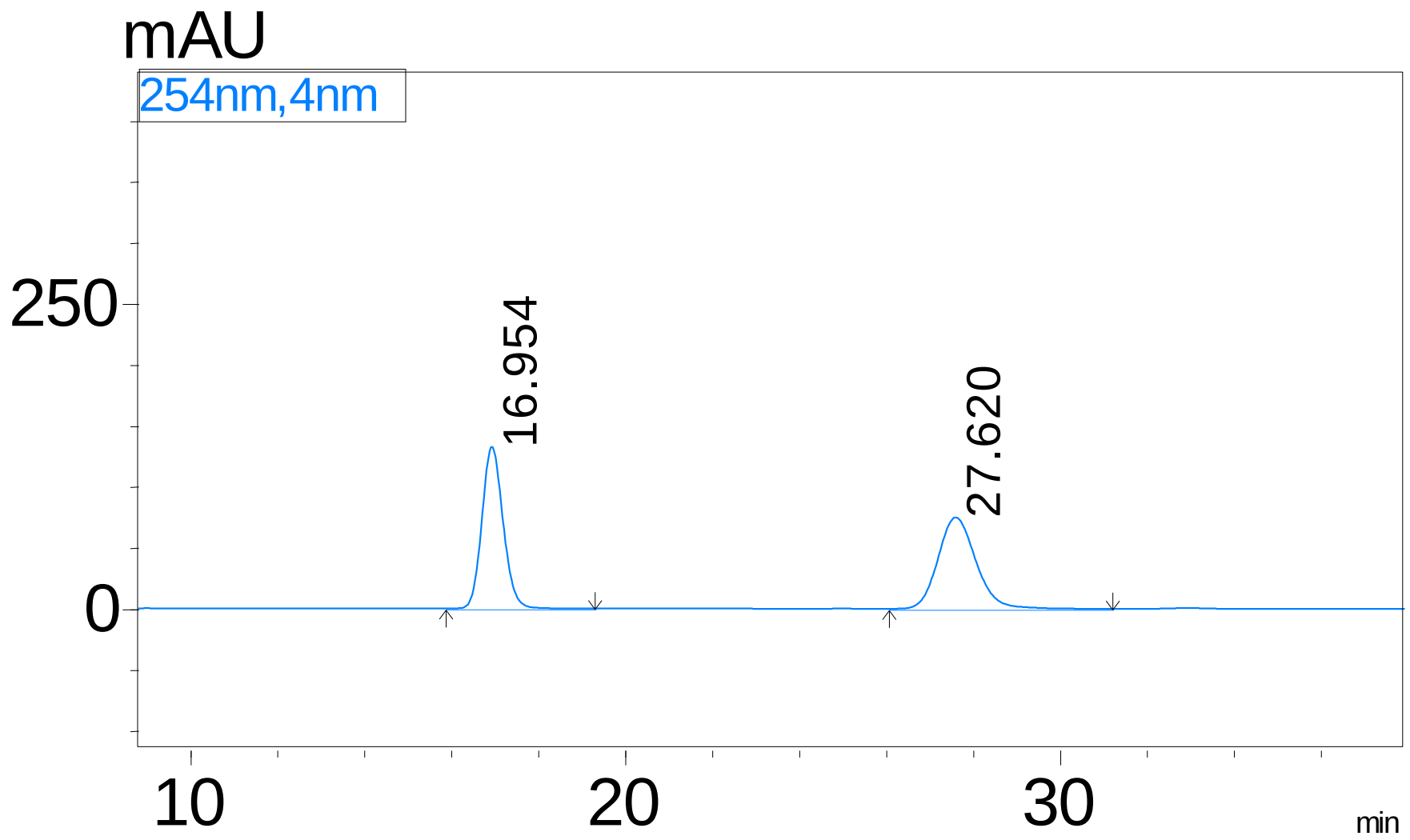

Peak	Time	Area	Height	Area%
1	16.954	4491338	132393	49.834
2	27.620	4521341	74917	50.166

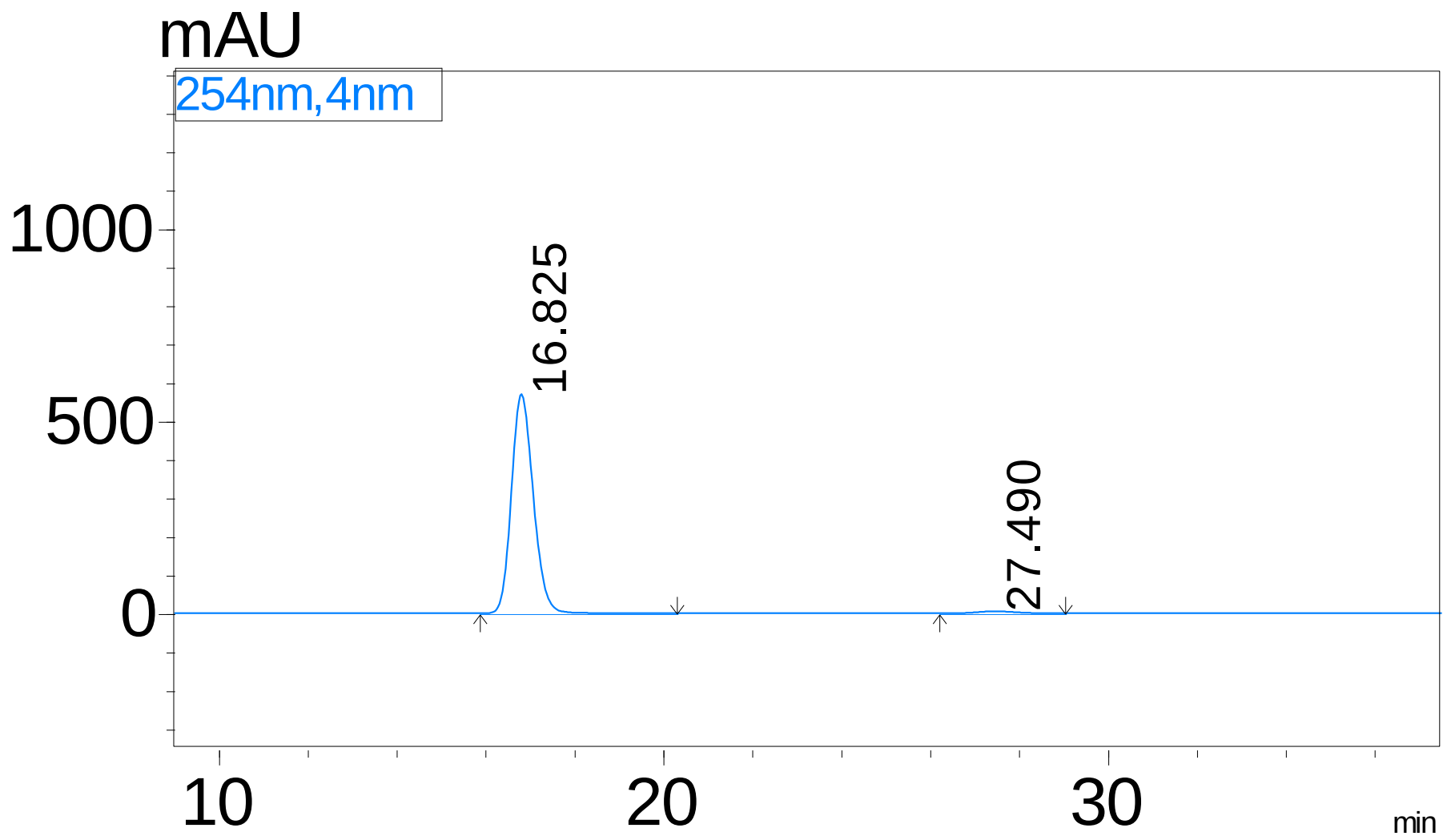

Peak	Time	Area	Height	Area%
1	16.825	19436484	569243	98.540
2	27.490	287913	4976	1.460

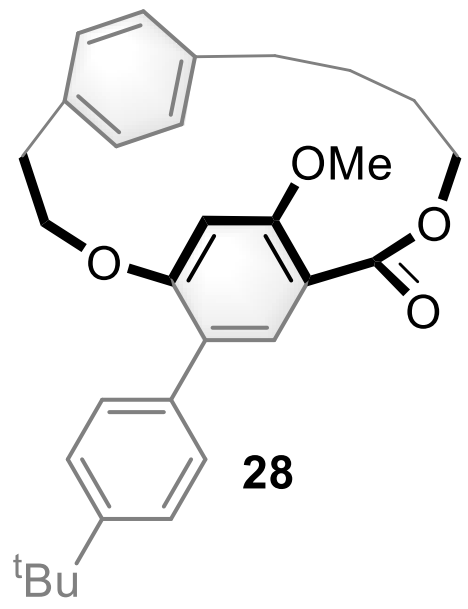

28

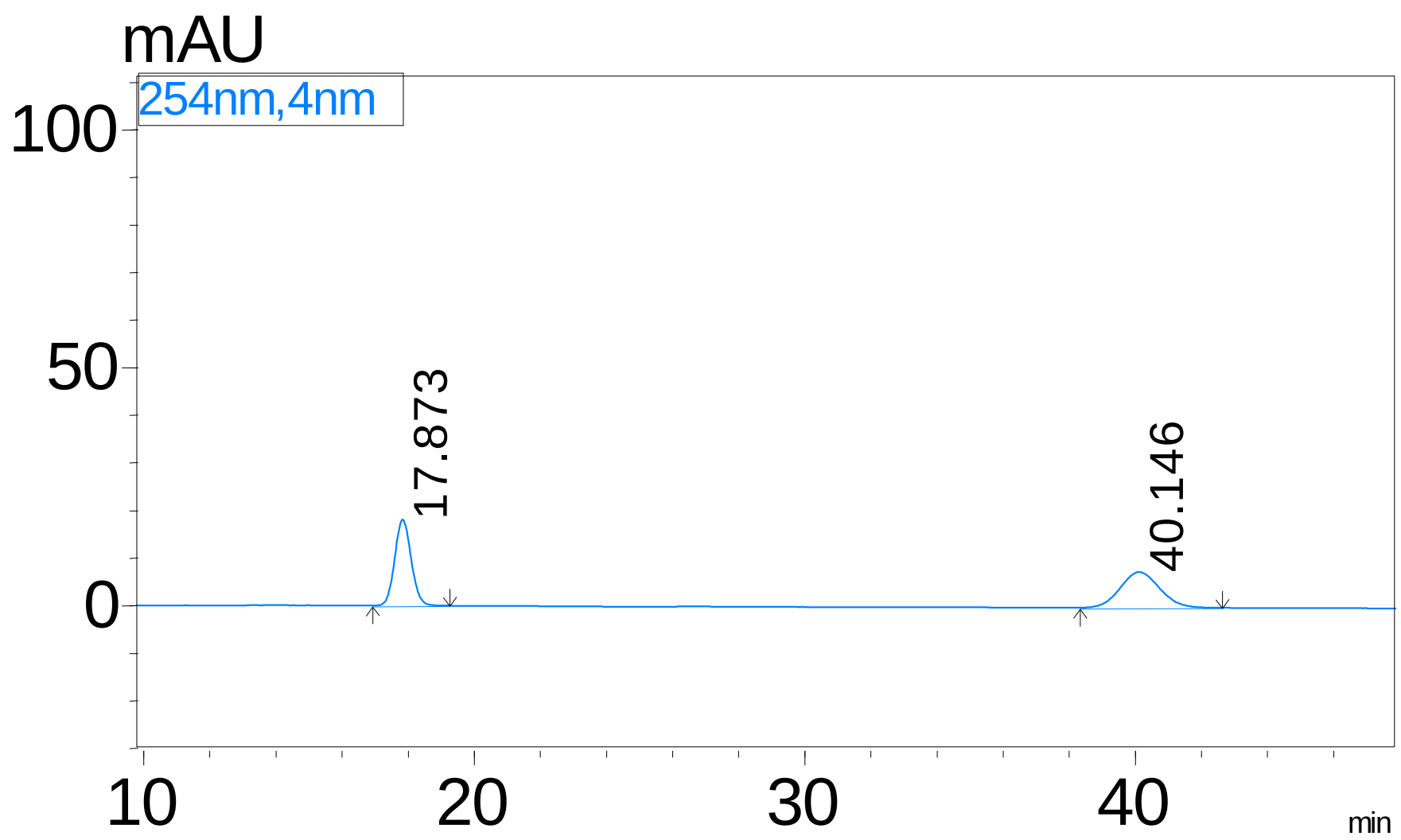

Peak	Time	Area	Height	Area%
1	17.873	624243	18092	50.172
2	40.146	619959	7479	49.828

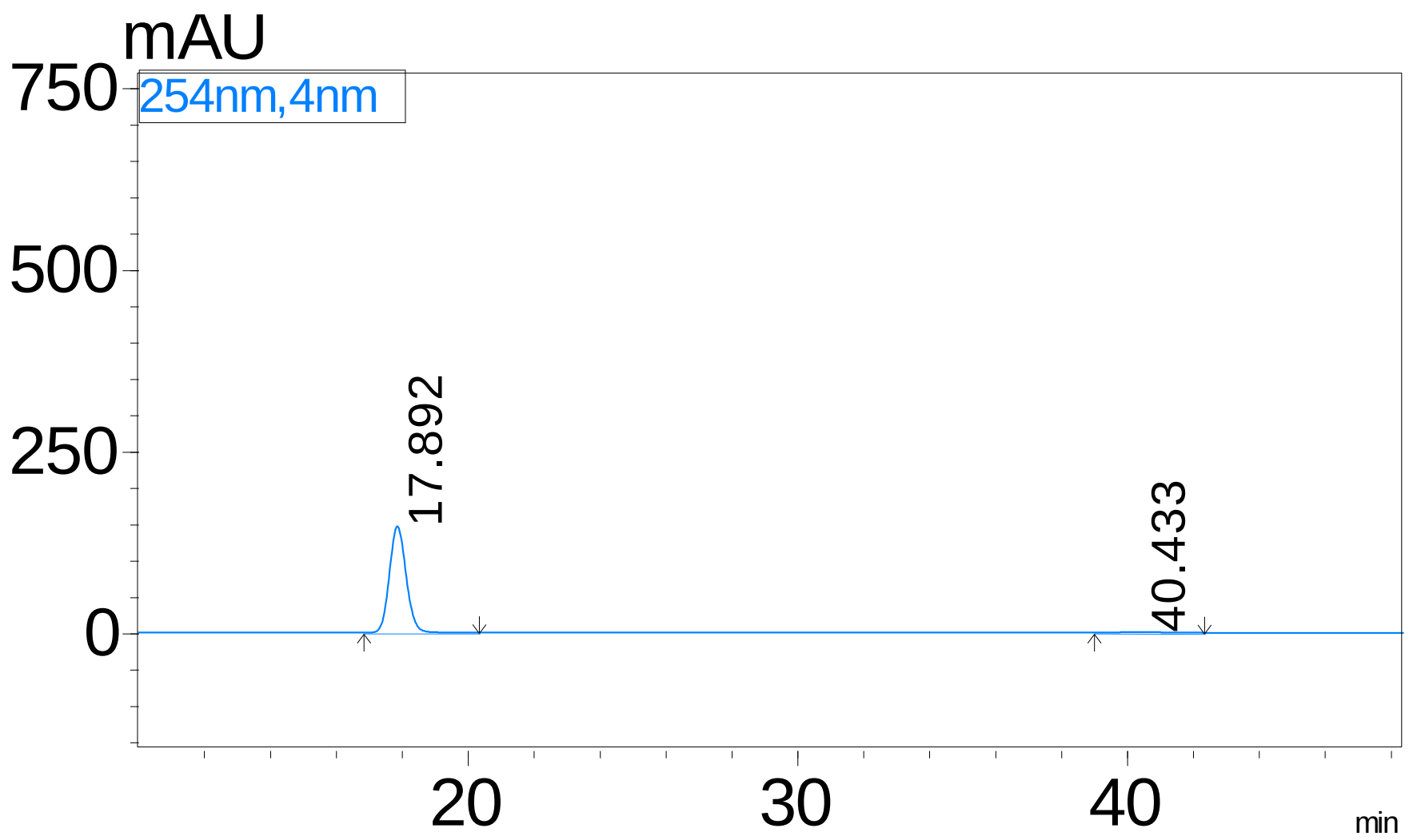

Peak	Time	Area	Height	Area%
1	17.892	5143855	146533	98.339
2	40.433	86889	1055	1.661

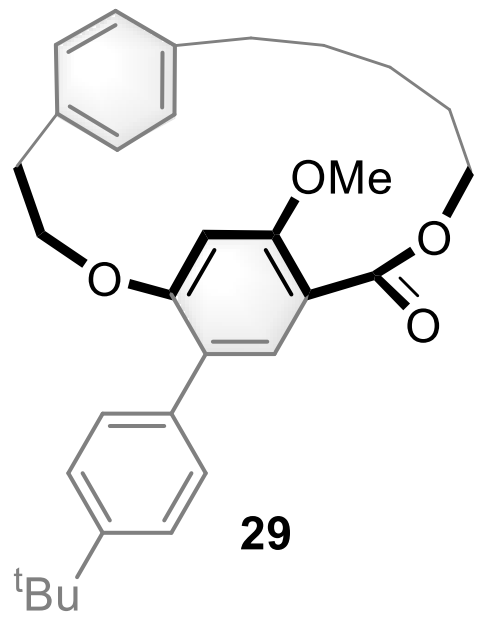

29

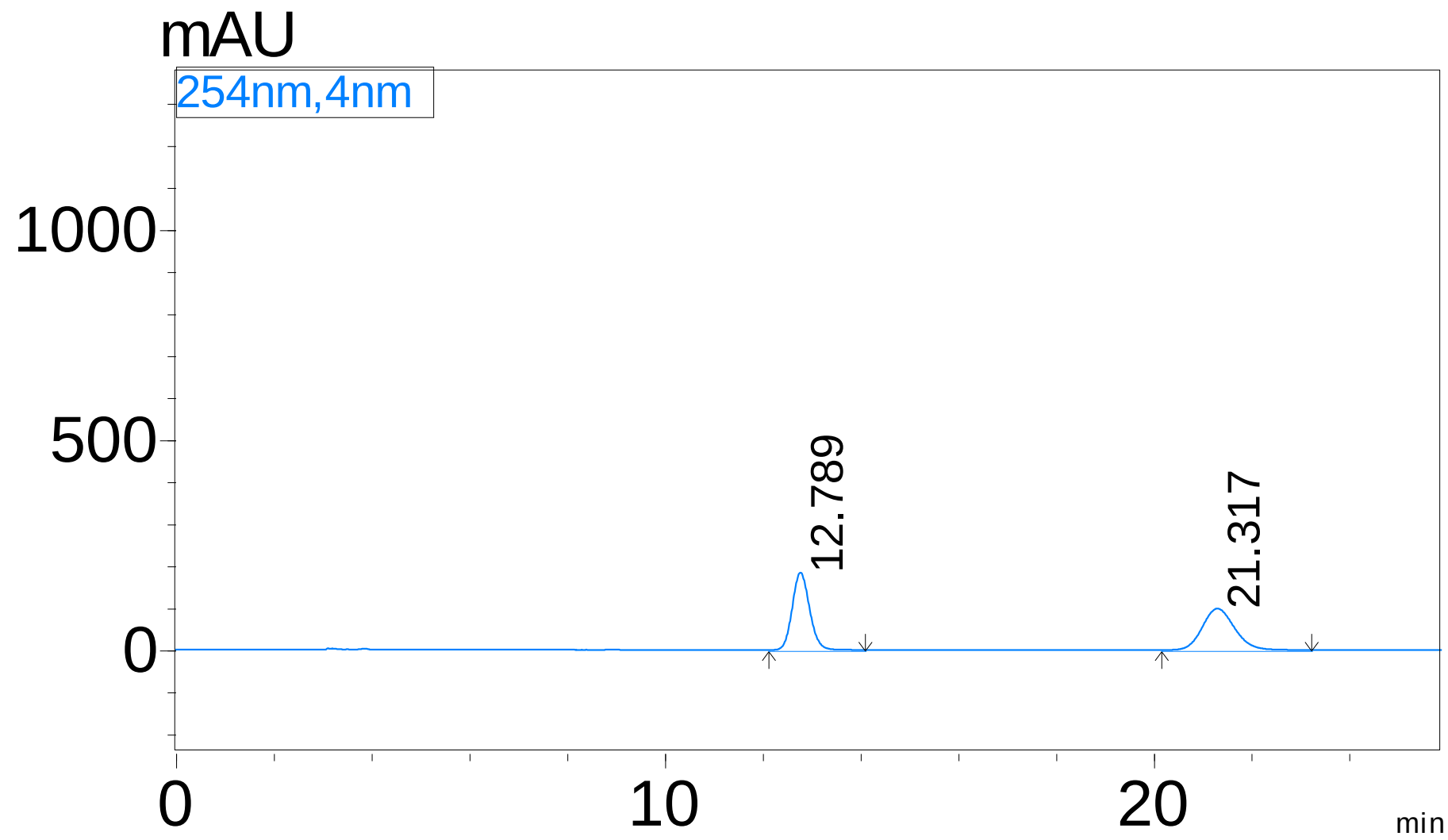

Peak	Time	Area	Height	Area%
1	12.789	4517587	183989	50.093
2	21.317	4500788	98435	49.907

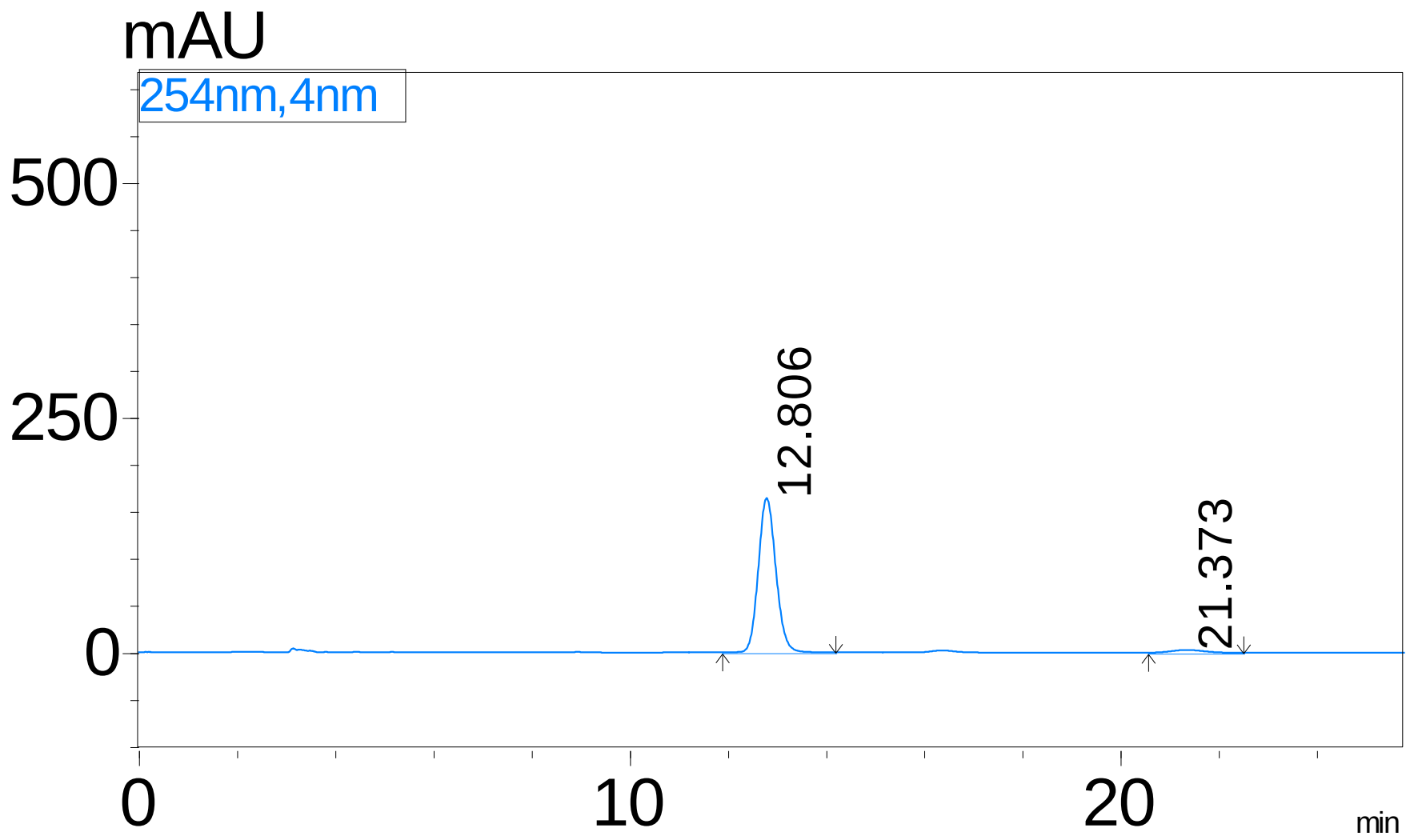

Peak	Time	Area	Height	Area%
1	12.806	4076182	164228	96.865
2	21.373	131918	2935	3.135

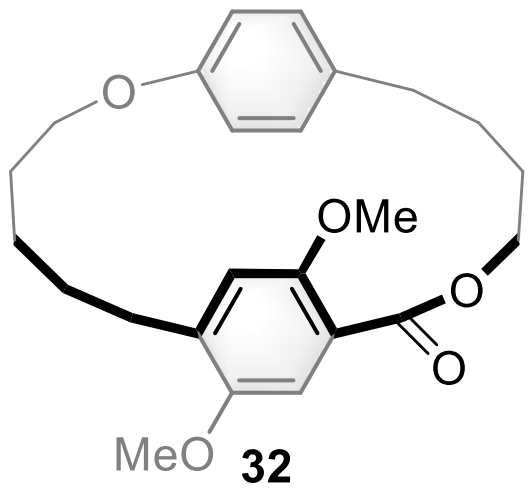

32

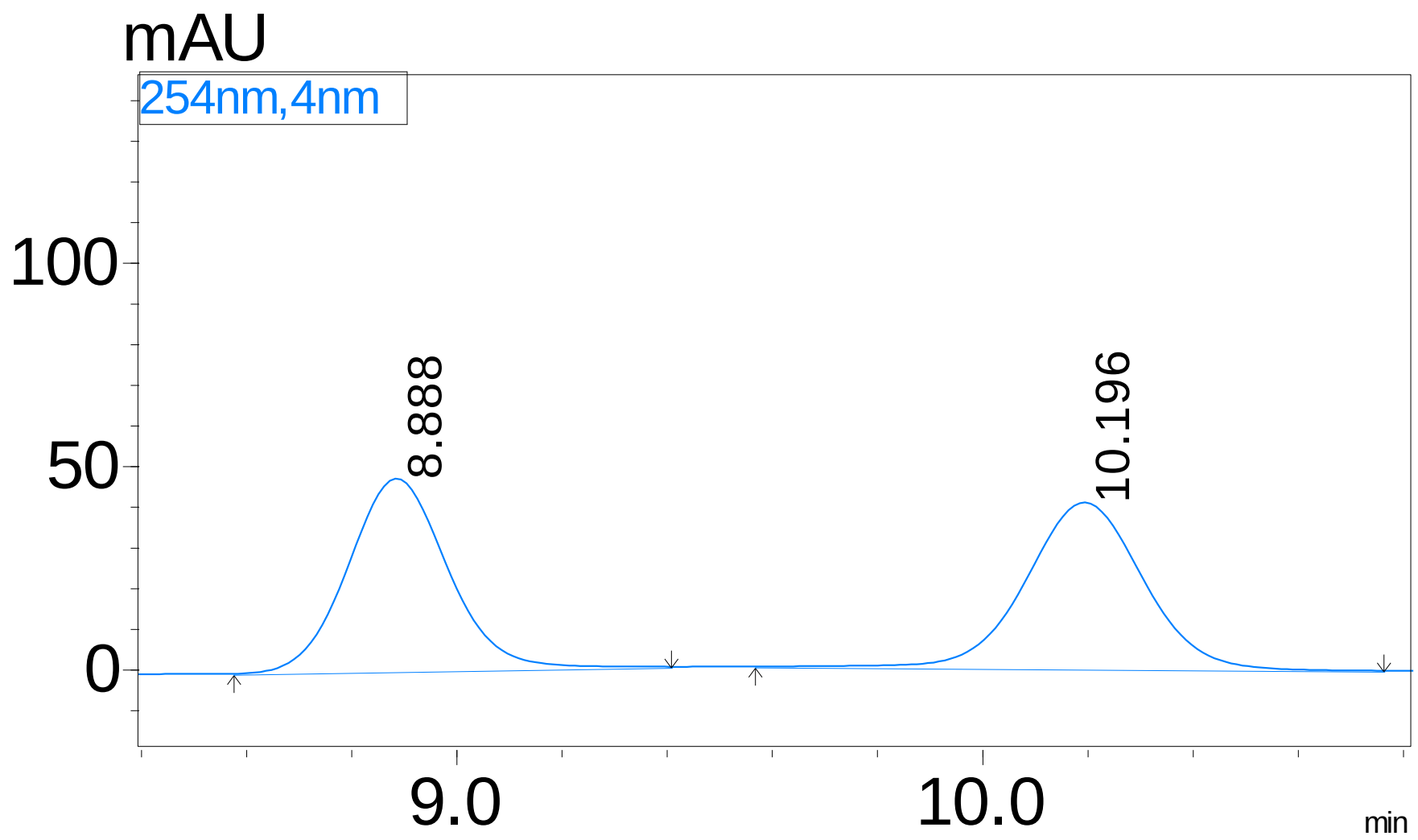

Peak	Time	Area	Height	Area%
1	8.888	626094	47330	49.720
2	10.196	633153	40856	50.280

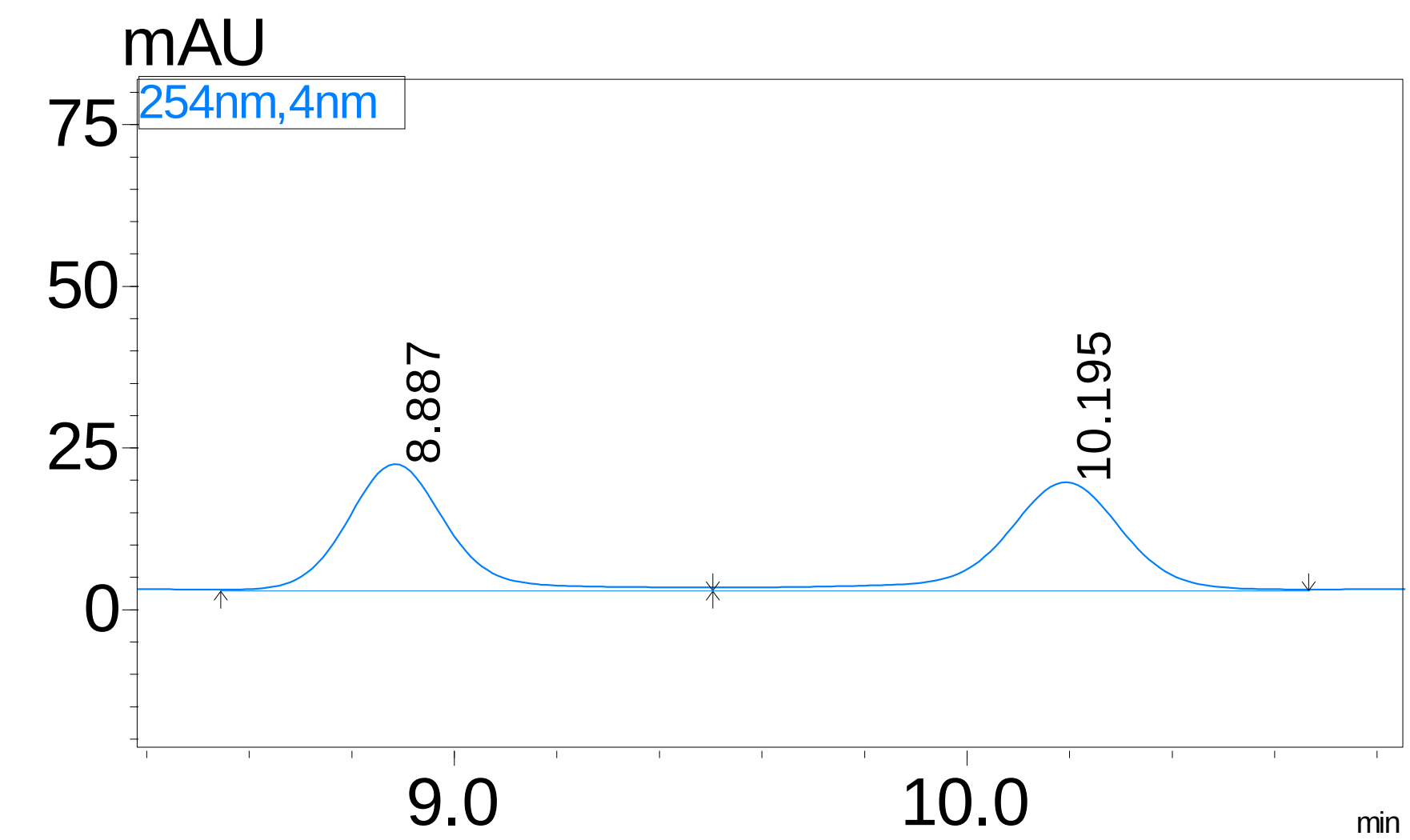

Peak	Time	Area	Height	Area%
1	8.887	267267	19420	49.781
2	10.195	269614	16593	50.219

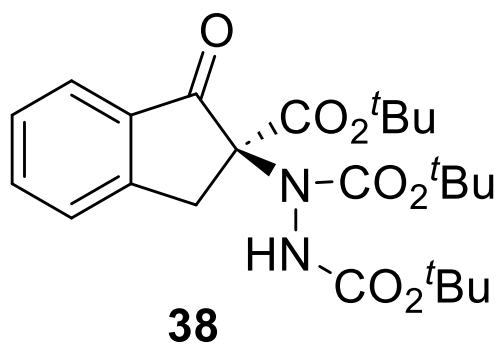

38

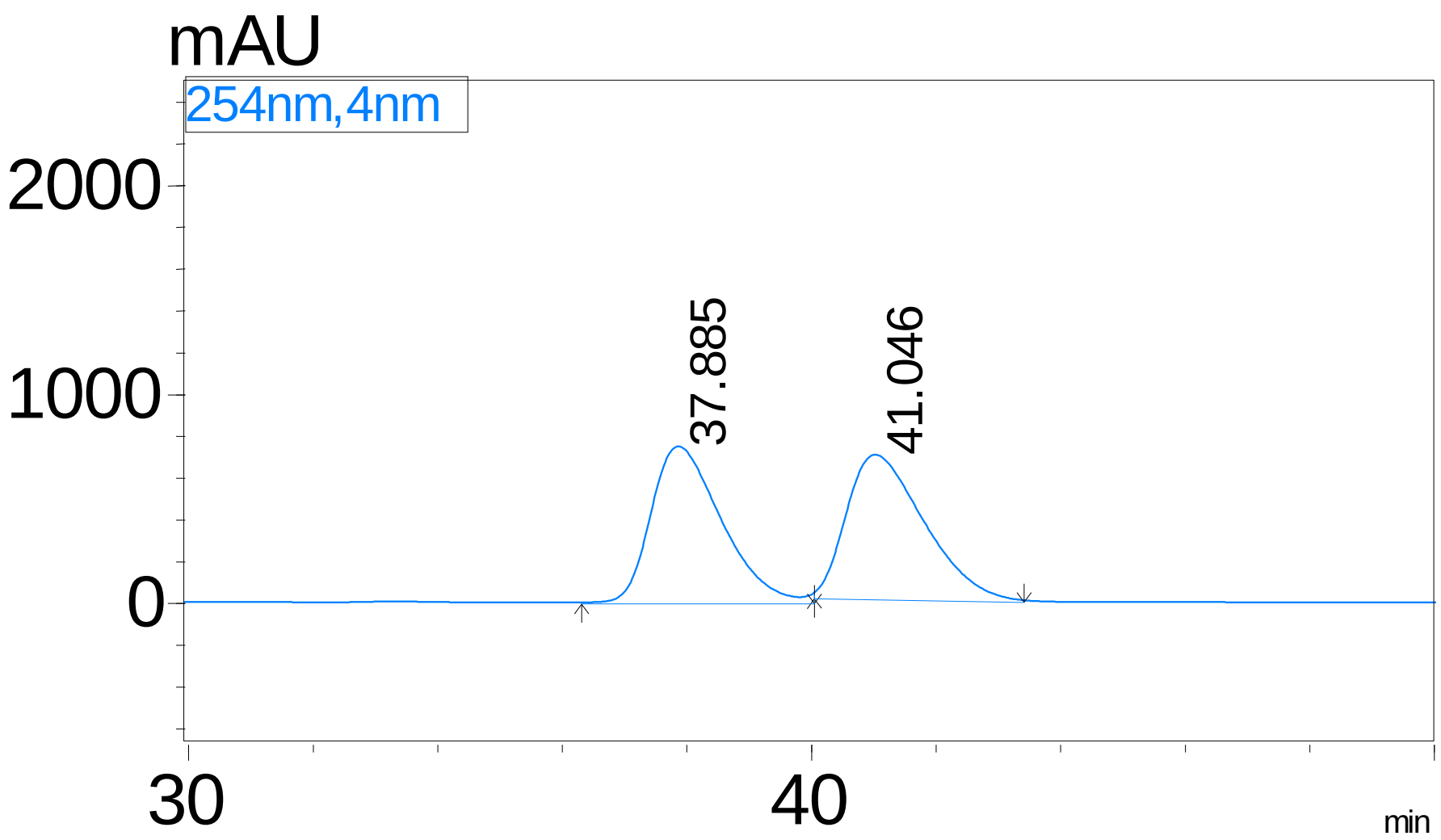

Peak	Time	Area	Height	Area%
1	37.885	58497431	746606	49.658
2	41.046	59303876	687551	50.342

34 was used:

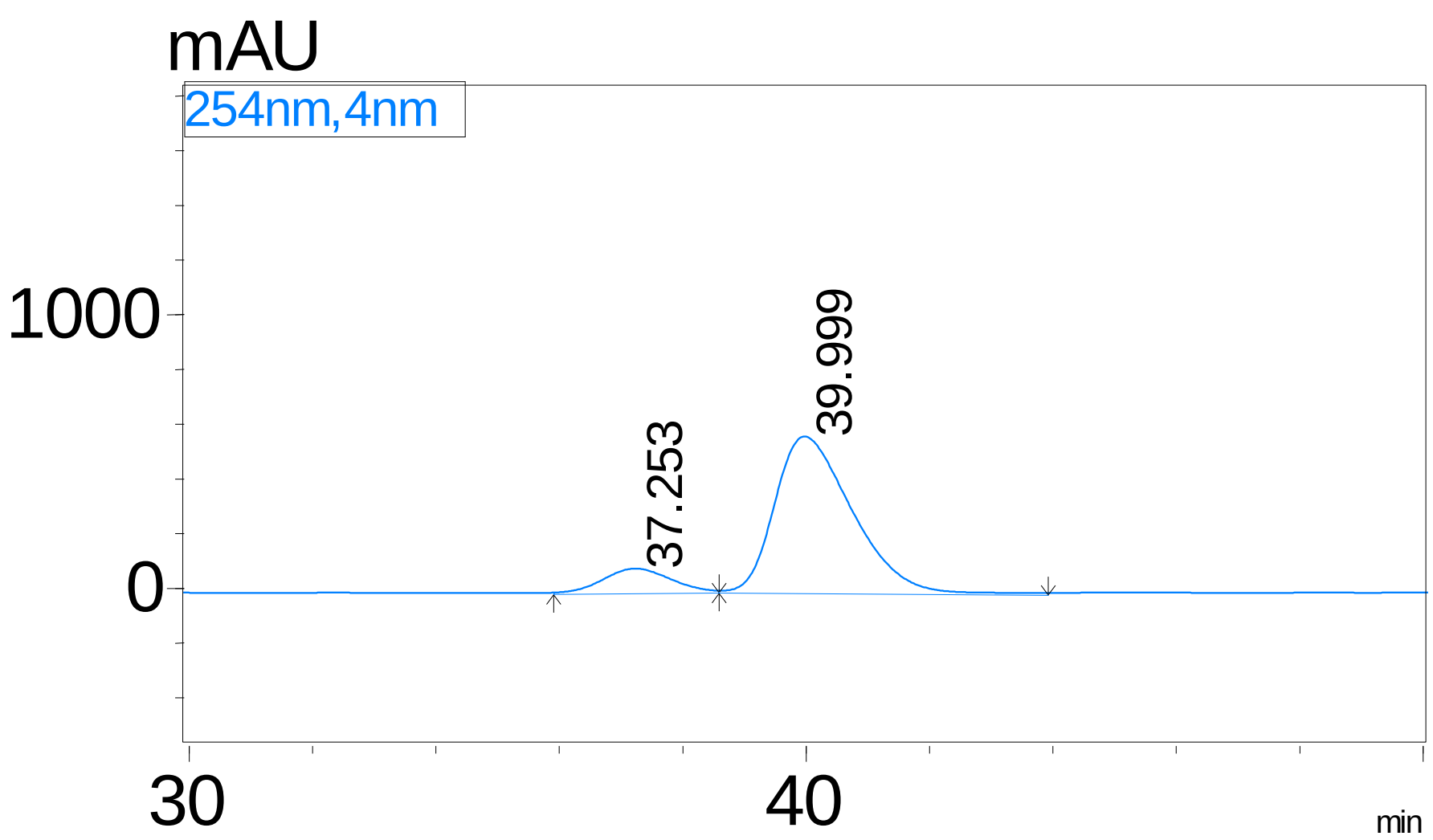

Peak	Time	Area	Height	Area%
1	37.253	6325680	85914	11.395
2	39.999	49185957	568736	88.605

35 was used:

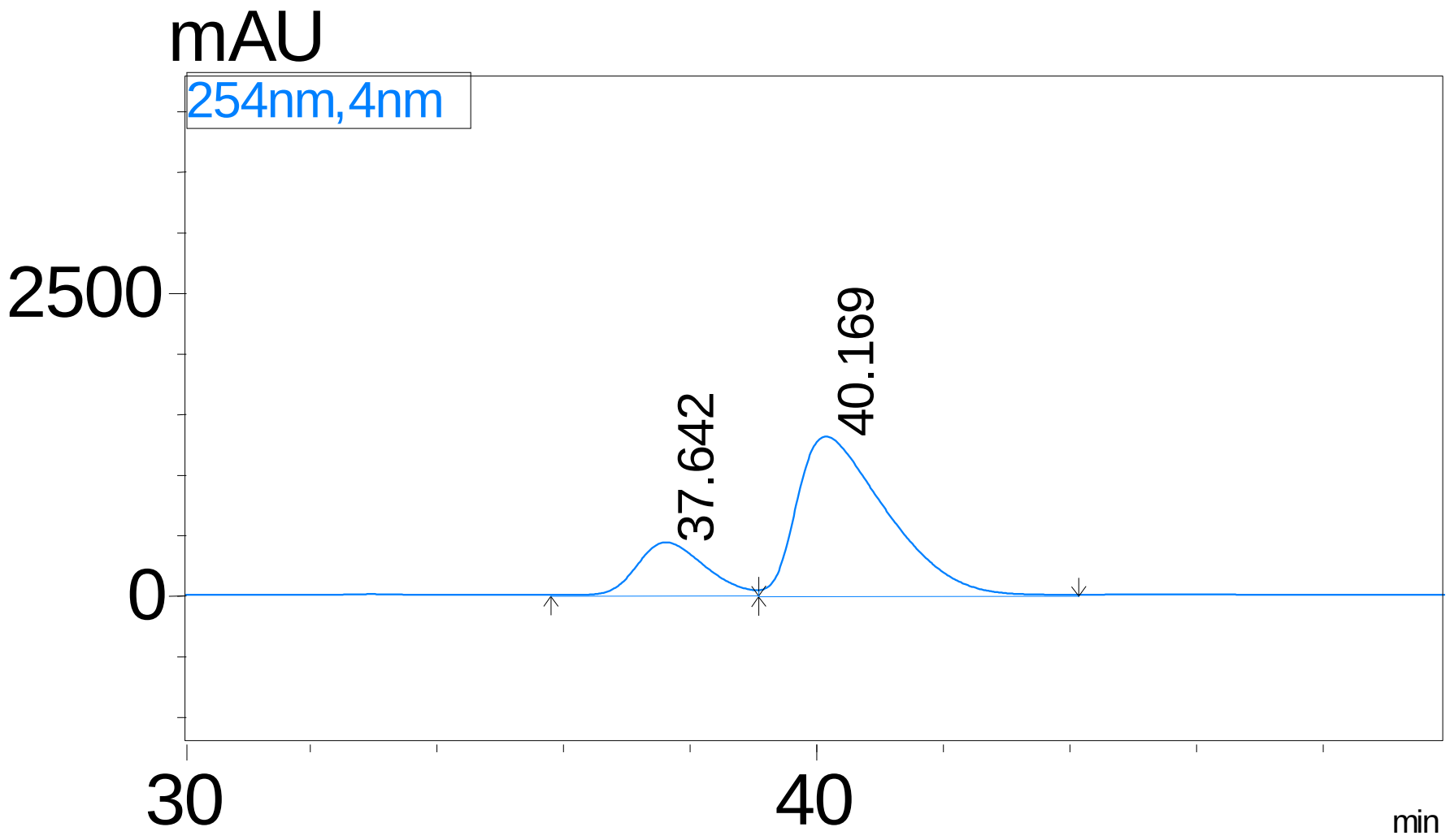

Peak	Time	Area	Height	Area%
1	37.642	32319546	434655	20.117
2	40.169	128335886	1310372	79.883
